# N-Directed fluorination of unactivated Csp^3^–H bonds†

**DOI:** 10.1039/c9sc04055b

**Published:** 2019-12-16

**Authors:** Emily N. Pinter, Jenna E. Bingham, Deyaa I. AbuSalim, Silas P. Cook

**Affiliations:** Department of Chemistry, Indiana University 800 East Kirkwood Avenue Bloomington IN 47405-7102 USA sicook@indiana.edu

## Abstract

Site-selective fluorination of aliphatic C–H bonds remains synthetically challenging. While directed C–H fluorination represents the most promising approach, the limited work conducted to date has enabled just a few functional groups as the arbiters of direction. Leveraging insights gained from both computations and experimentation, we enabled the use of the ubiquitous amine functional group as a handle for the directed C–H fluorination of Csp^3^–H bonds. By converting primary amines to adamantoyl-based fluoroamides, site-selective C–H fluorination proceeds under the influence of a simple iron catalyst in 20 minutes. Computational studies revealed a unique reaction coordinate for the catalytic process and offer an explanation for the high site selectivity.

## Abstract 2

By converting primary amines to adamantoyl-based fluoroamides, site-selective C–H fluorination proceeds under the influence of a simple iron catalyst in 20 minutes.

## Graphical abstract

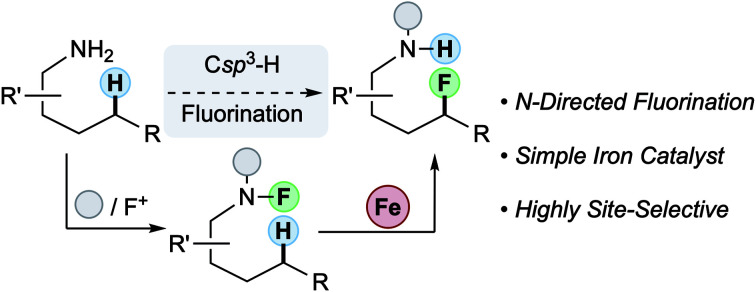

Due to the pervasiveness of fluorine atoms in industrially relevant small molecules, all practicing organic chemists appreciate the importance of this element. As a result of its unusual size and electronegativity, fluorine imparts unique physicochemical properties to pendant organic molecules.^1^ For example, the strong C–F bond can prevent biological oxidation pathways, thereby thwarting rapid clearance and potentially improving pharmacokinetics of molecules.^2^ Moreover, the installation of fluorine or trifluoromethyl groups, with their strong inductive effects,^2^ can have a profound effect on the p*K*_a_ of nearby hydrogen atoms.^3^ These attributes, among others, have solidified the importance of fluorinated molecules in the medicinal,^1–4^ material,^5^ and agrochemical^6^ industries. Yet, the same unique properties that make fluorine atoms attractive chemical modifiers also make their installation difficult. Consequently, new methods for site-selective fluorine incorporation remain highly desirable.^7^

Methods to construct Csp^2^–F bonds traditionally make use of the Balz–Schiemann fluorodediazonization^8^ and halogen exchange (“Halex” process).^9^ Advances in transition metal-mediated fluorination have broadened access to Csp^2^–F-containing molecules,^10^ but methods to access aliphatic fluorides remain limited. Conventional methods to make Csp^3^–F bonds—such as nucleophilic displacement of alkyl halides^11^ and deoxyfluorination^12^—can have limited functional group compatibility and unwanted side reactions. A more efficient route to form aliphatic C–F bonds would target the direct fluorination of Csp^3^–H bonds (Scheme 1).^13^

**Scheme 1:**
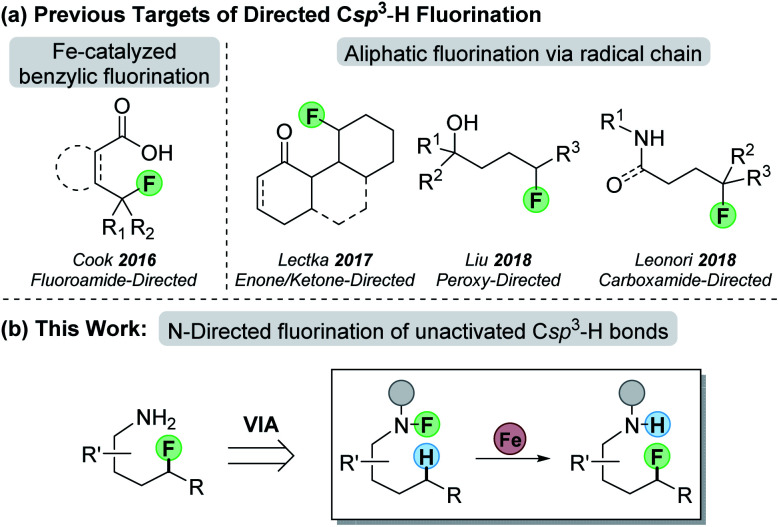
(a) Previous work on functional-group directed Csp^3^–H fluorination; (b) our approach to N-directed fluorination.

Recent efforts with palladium catalysis employ conventional C–H-metallation strategies to target Csp^3^–H bonds for fluorination.^14^ Alternatively, radical H-atom abstraction can remove the transition metal from the C–H-cleavage step, thereby offering a promising approach for Csp^3^–H-bond functionalization.^15^ With *undirected* C–H fluorination,^16^ however, selectivity remains a challenge in molecules without strength-differentiated Csp^3^–H bonds.^17^ To overcome this, our group pioneered the *directed* fluorination of benzylic Csp^3^–H bonds through an iron-catalyzed process that involves 1,5 hydrogen-atom transfer (HAT) to cleave the desired Csp^3^–H bond.^18^ Since this work, other groups have demonstrated directed Csp^3^–H fluorination based on radical propagation that proceeds through an interrupted Hofmann–Löffler–Freytag (HLF)^19^ reaction (Scheme 1a). These examples employ various radical precursors such as enones,^20^ ketones,^21^ hydroperoxides,^22^ and carboxamides^23^ to direct fluorination to specific Csp^3^–H bonds. Since amines are ubiquitous in natural products and drugs, we sought to use amines as the building block of our directing group to achieve fluorination of unactivated Csp^3^–H bonds (Scheme 1b). By using amines as the starting point, one could use the approach in straightforward synthetic planning for the late-stage functionalization of remote C–H bonds.

In the design phase of the project, we needed to devise a synthetically tractable N–F system that would enable 1,5-HAT and allow for fluorine transfer (Scheme 1b). To begin, we decided to examine common amine activating groups that would support 1,5-HAT while avoiding undesired radical reactions. The chosen activating group would provide the ideal steric and electronic properties to enable both N–F synthesis and N–F scission for 1,5-HAT. We first examined common acyl groups (*e.g.*, acetyl-, benzoyl, and tosyl-based amides), but these proved unsatisfactory. For example, fluoroamide synthesis was either not achieved or low yielding, and the desired fluorine transfer proceeded with significant side reactions or returned starting material. We then turned our attention to more sterically hindered amides—which allow for higher yielding fluoroamide synthesis. For fluorine transfer, we hypothesized that the increased steric bulk could slow intermolecular H-atom transfer, thereby leading more efficient intramolecular 1,5-HAT. To that end, we were delighted that pivaloyl-based fluoroamide **1a** proceeded in 64% yield to form product **2a** (Scheme 2a). Interestingly, 7% of **1a** underwent fluorination at the *tert*-butyl group of the pivaloyl—presumably through a 1,4-HAT reaction (**2aa**, Scheme 2a).^24^ The problem is further exacerbated when the pivaloyl group is homologated by one methylene—providing only 7% yield of desired **2b** with 32% of the fluorination taking place on the iso-pentyl group (**2bb**, Scheme 2a). In an attempt to “tie back” the pivaloyl group and prevent the undesired fluorination, we employed a cyclopropylmethyl-based fluoroamide but observed no improvement.

**Scheme 2:**
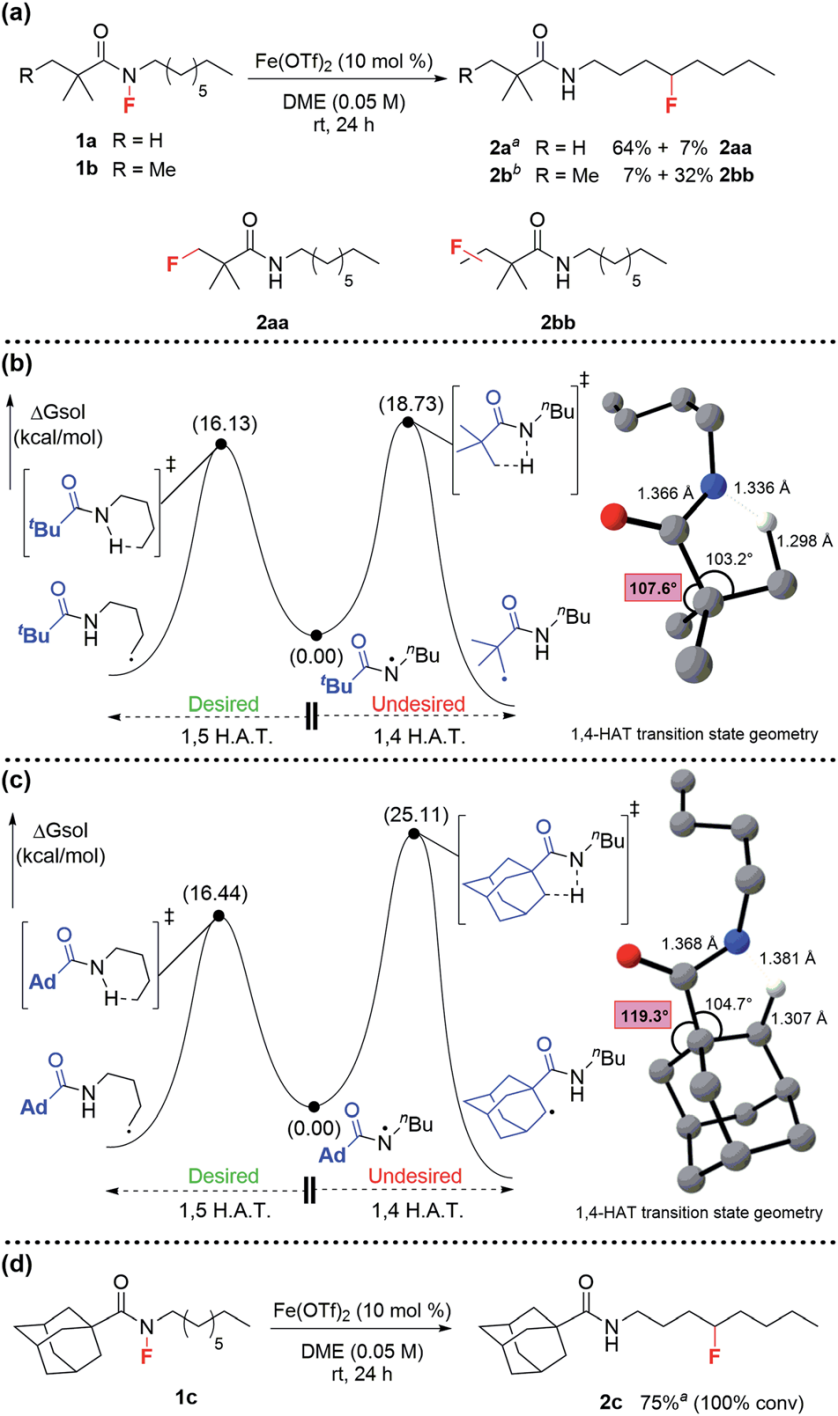
(a) The targeted 1,5-fluorination of unactivated aliphatic C–H bonds results in partial fluorination of the amine activating group; (b) DFT studies (uM06/cc-pVTZ(-f)-LACV3P**//uM06/LACVP** level of theory) identified the competing pathways responsible for alternate fluorination; (c) DFT (uM06/cc-pVTZ(-f)-LACV3P**//uM06/LACVP** level of theory) evaluation of adamantoylamides revealed higher transition state energy for 1,4-HAT due to restricted vibrational scissoring (d) adamantoyl-activated octylamine shows no fluorination of the activating group. ^*a* 1^H-NMR yield using 1,3,5-trimethoxybenzene as an internal standard. ^*b* 19^F-NMR yield using 4-fluorotoluene as an internal standard.

At this point, **1a** proved most promising for efficient fluorine transfer, as well as being the most synthetically accessible fluoroamide. The increased steric hindrance minimizes *N*-sulfonylation during fluorination with NFSI, a problem that plagued the synthesis of our previously targeted fluoroamides.^18^ Therefore, to further investigate how to improve fluorine transfer from **1a**, we decided to model H-abstraction computationally.

We hypothesized that the fluorinated side product **2aa** was formed after 1,4-HAT. Since 1,4-HAT is rare,^24^ we employed DFT (see ESI† for details) to calculate the 5-membered and 6-memebered transition-states for 1,4- and 1,5-HAT, respectively. Surprisingly, we found that the barrier for 1,4 C–H abstraction in **1a** was 18.7 kcal mol^−1^, which was only 2.6 kcal mol^−1^ higher in energy than the barrier calculated for 1,5 C–H abstraction in the same system (Scheme 2b). This suggested that both processes were competing at room temperature. We attributed the comparable barriers to the flexibility of the *tert*-butyl group, which undergoes vibrational scissoring to accommodate the C–H abstraction. The transition state distortion is modest and allows the molecule to maintain bond angles close to the ideal 109.5° (Scheme 2b). Based on this insight, we sought to limit the scissoring of the *tert*-butyl group and prevent the 1,4-HAT that leads to the undesired side product. After investigating several possible candidates, the underutilized adamantoyl group appeared promising. To evaluate the rigidity of adamantane, we calculated the barriers for 1,4- and 1,5-HAT for the adamantoyl-capped octylamine **1c** (Scheme 2c). As expected, the barriers for 1,4- and 1,5-HAT differed significantly—with 1,4 C–H abstraction proceeding with a barrier of 25.1 kcal mol^−1^ and the 1,5-HAT barely changed at 16.4 kcal mol^−1^—an 8.7 kcal mol^−1^ difference. Consequently, we synthesized **1c** and subjected it to the reaction conditions. Excitingly, the adamantoyl-capped system produced desired product **2c** in 75% yield with no fluorination of the adamantyl group (Scheme 2d).

Using the newly devised adamantoyl-based fluoroamides, the reaction conditions were optimized. While a range of metal salts, ligands, and radical initiators were evaluated, Fe(OTf)_2_ proved unique in catalyzing fluorine transfer with fluoroamides.^18^ Catalyst loading of 10 mol% allowed convenient setup and minor deviations above or below this loading had little effect on yield (see ESI†). Increasing the temperature to 40 °C produced a slight increase in yield (entry 2, Table 1). Likewise, raising the temperature to 80 °C resulted in *full conversion of the starting material in 20 minutes with 81% yield of the desired product* (entry 3, Table 1). It should be noted that fluorine transfer occurs efficiently at a variety of temperatures with adjustments in reaction time (see ESI†). Increasing the reaction concentration or changing the solvent resulted in decreased yield (entries 4 and 5, Table 1). Furthermore, the absence of Fe(OTf)_2_ leads to no reaction and quantitative recovery of starting material, attesting to the stability of fluoroamides and the effectiveness of Fe(OTf)_2_ (entry 6, Table 1).

**Table 1:** Optimization of pertinent reaction parameters

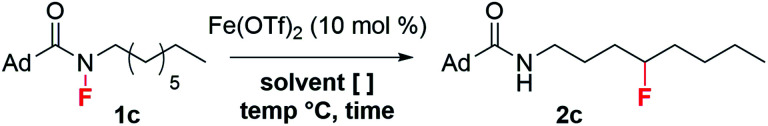					
Entry	Solvent	Temp (°C)	Conc (M)	Time	Yield^a^ (%)
1^b^	DME	rt	0.05	15 h	75
2	DME	40	0.05	18 h	79
**3**	**DME**	**80**	**0.05**	**20 min**	**81**
4	DME	80	0.1	20 min	73
5	THE	80	0.05	20 min	38
6^c^	DME	80	0.05	20 min	0

a Determined by ^1^H-NMR with 1,3,5-trimethoxybenzene as an internal standard.

b Reaction ran inside of glovebox.

c Reaction ran without Fe(OTf)_2_.

With the optimized conditions established, we evaluated the substrate scope of the reaction (Table 2). The reaction proved quite general for the fluorination of primary and secondary Csp^3^–H bonds (**2c–l**, Table 2), while tertiary Csp^3^–H abstraction led to greater side reactions and lower yields (**2m**). While all reactions resulted in complete consumption of the fluoroamide, only a singly fluorinated product is produced with the parent amide being the major side product (see ESI†). The reaction proved selective for δ-fluorination even in the presence of tertiary Csp^3^–H bonds (*e.g.*, **2h**, **2j**, and **2k**), thereby demonstrating selectivity counter to C–H-bond strength. Interestingly, transannular fluorine transfer occurs with complete regioselectivity to produce **2l** as the sole product. Additionally, benzylic C–H bonds can be fluorinated under these conditions (**2n**). The reaction also exhibits good functional group compatibility, allowing access to a variety of fluorinated motifs. In particular, the reaction proceeds in the presence of either free or protected alcohols (**2o** and **2p**). Moreover, esters and halides are both tolerated to give fluorinated products **2q** and **2r** in good yield. Notably, the reaction provides access to fluorohydrin **2s**—highlighting the unique ability of this methodology to access both fluorohydrins and γ-fluoroalcohols such as **2o**. In addition to these examples, terminal alkene **1t** works quite well giving **2t** in 67% yield. Furthermore, alkene functionalizations of **2t** would provide access to a diverse range of fluorinated motifs. To target difluoromethylene units with this methodology, fluoroamide **1u** was prepared and subjected to the reaction conditions. Pleasingly, **2u** was observed in 20% yield.

**Table 2:** Substrate scope for fluorine transfer

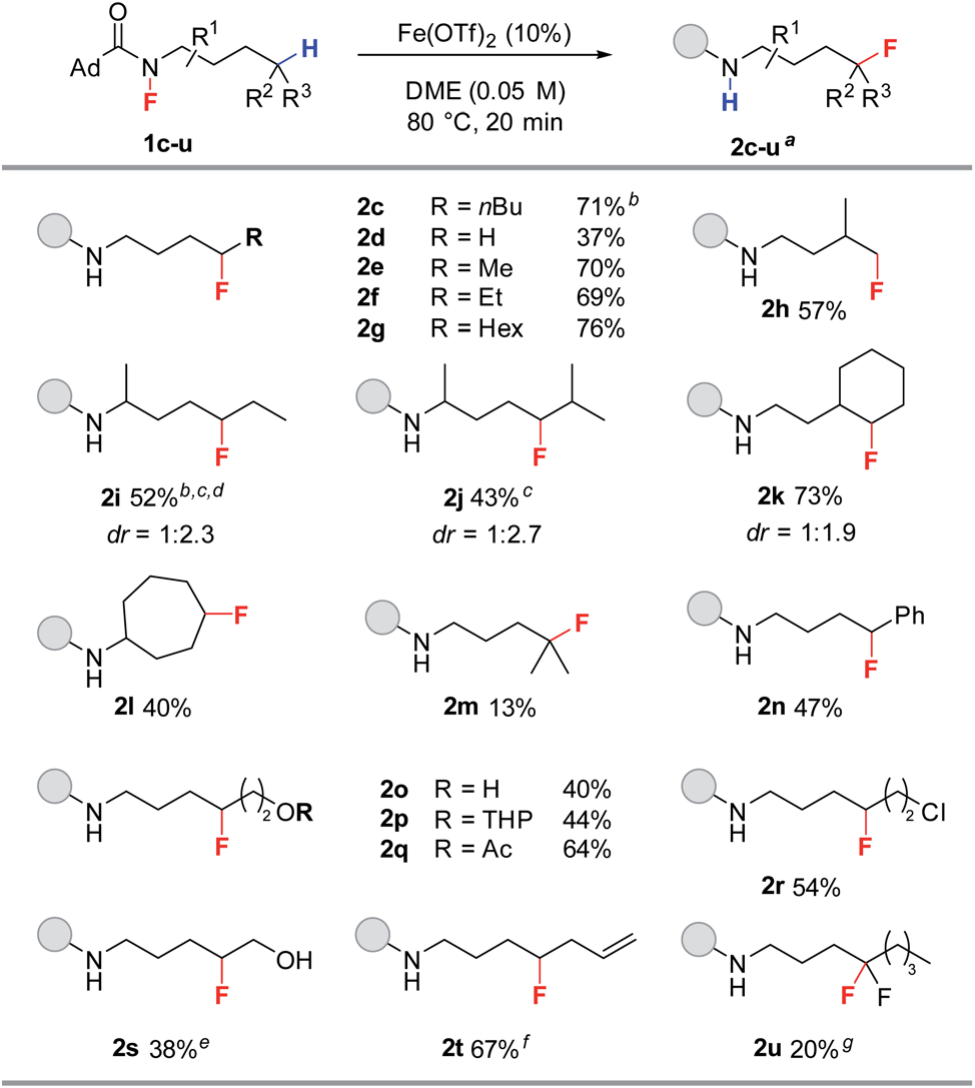

a Isolated yields. All reactions were run on 0.3 mmol scale unless otherwise noted.

b Yield reported as an average of two trials.

c 35 min reaction time.

d dr = 1 : 3.2 when ran at room temperature for 24 h.

e 0.25 mmol scale.

f 0.18 mmol scale.

g 0.1 mmol scale, yield determined by ^19^F-NMR with 4-fluorotoluene as an internal standard.

While exploring the substrate scope, we were surprised to discover that the fluoroamide N–F bond is unusually stable to a variety of common reactions. For example, fluoroamide **1o** was carried through an Appel reaction, PCC oxidation, and Wittig reaction with minimal loss of the fluoroamide. With such robustness, it becomes obvious that fluoroamides could act as secondary amide protecting group—being installed and carried through a multi-step synthesis until fluorine transfer is desired. Moreover, the greater rigidity of adamantoyl-based amides relative to pivalamides offers greater stability to acid and base hydrolysis—another feature of this system. Fortunately, the amide can be cleaved using conditions reported by Charette *et al.* with no evidence of elimination or loss of the alkyl fluoride (see ESI†).^25^

To evaluate the differences between C–H bonds, we calculated the hypothesized minima and maxima en route to C–F bond formation for primary, secondary, and tertiary substrates (Fig. 1). To begin, we defined the start of the pathway with the fluoroamides as octahedral, high-spin Fe(OTf)_2_-DME complex (**I**).^18^ Ligand dissociation results in the loss of DME to form **II** which is 7.2 kcal mol^−1^ higher in energy relative to **I**. This ligand loss opens a coordination site that allows Fe to enter the catalytic cycle *via* F-abstraction from the fluoroamides. This proceeds with a barrier (**II-TS**) of ∼25 kcal mol^−1^ for all systems to form the corresponding N-based radical (**III**). This new N-based radical is generally about −10 kcal mol^−1^ from the starting materials. The 1,5-HAT proceeds through a six-membered transition state (**III-TS**) with 16.4, 12.6, and 9.7 kcal mol^−1^ barriers for primary, secondary, and tertiary substrates, respectively. This abstraction forms the corresponding C-based radicals (**IV**) that were −15.0, −19.9 and −22.4 kcal mol^−1^ relative to the starting materials for primary, secondary, and tertiary substrates, respectively. A barrierless transition allows for the abstraction of fluorine from Fe(iii)-fluoride to simultaneously furnish the products (**V**) and regenerate catalyst **II**. Interestingly, this transition seems to proceed with an intermolecular electron-transfer from the alkyl radicals to the Fe(iii) center. The overall process is highly exergonic at −53.7, −58.6, and −61.9 kcal mol^−1^ for primary, secondary, and tertiary substrates, respectively. We attribute the low yields for the tertiary example to rapid oxidation of the carbon radical, likely by Fe(iii), that forms a tertiary carbocation and leads to unwanted side reactions. The turnover-limiting step is the N–F abstraction by Fe (**II-TS**).

**Fig. 1:**
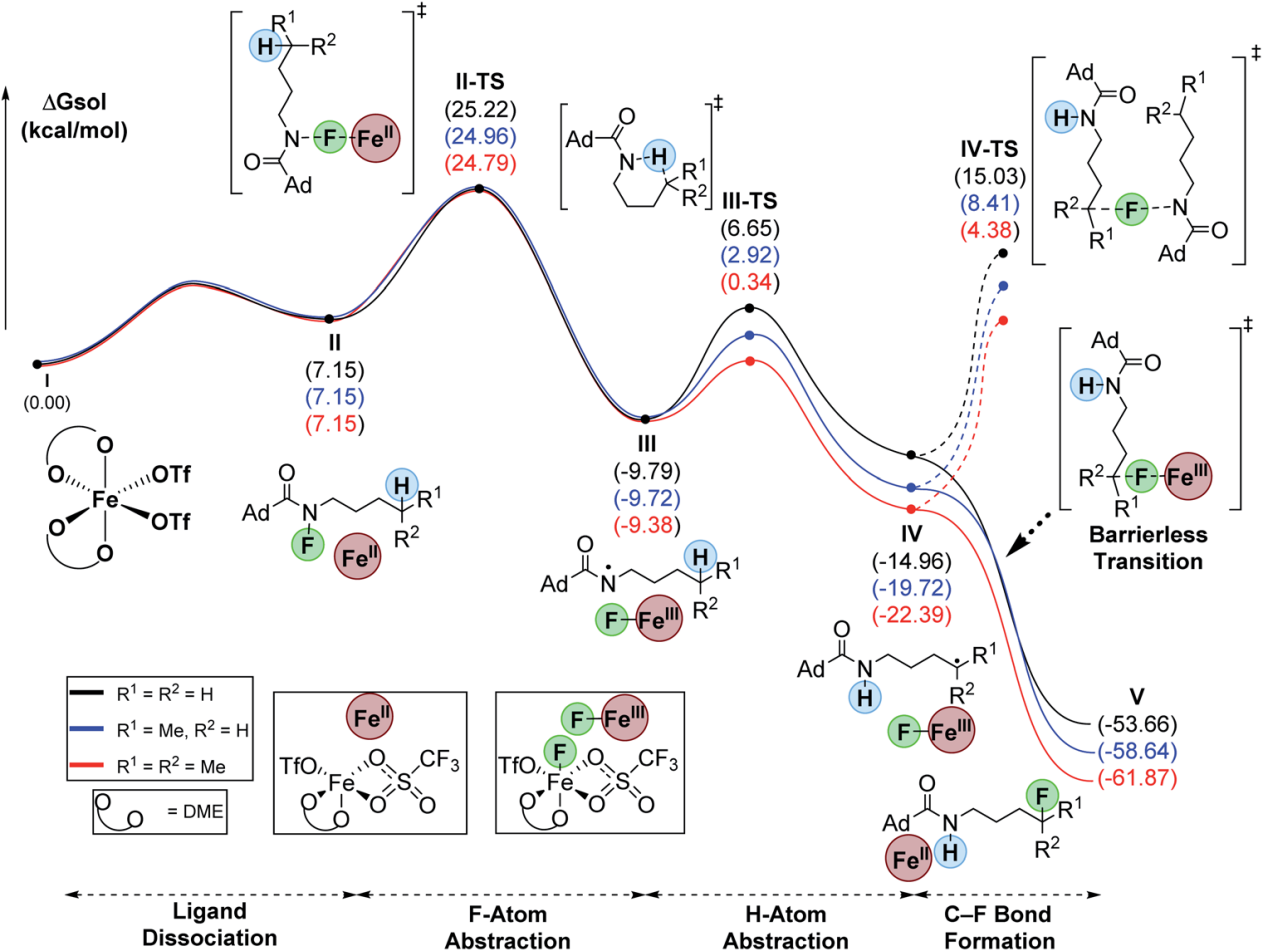
Computed relative Gibb's free energies for intermediates and transition-states along the reaction pathway (uM06/cc-pVTZ(-f)-LACV3P**//uM06/LACVP** level of theory).

An alternative pathway, related to the classic HLF reaction,^19*a*,*b*^ would involve radical chain propagation. Although unlikely, we also evaluated this pathway computationally (Fig. 1). Consistent with our previous report,^18^ this process proceeds with an unfavorably high barrier of 30.0, 28.1, and 26.8 kcal mol^−1^ for primary, secondary, and tertiary substrates, respectively. Hence, this process cannot compete with the barrierless delivery of fluorine from the Fe(iii) fluoride species.

In conclusion, we leveraged critical computational insights to enable the use of simple amines as a building block for the directed fluorination of C–H bonds. The reaction targets unactivated Csp^3^–H bonds site selectively regardless of bond strength. The reaction proceeds under mild iron catalysis that allows broad functional-group compatibility and provides access to unique fluorinated motifs. Moreover, we identified fluoroamides as surprisingly stable functional groups with likely implications for biology and materials. Mechanistic evaluation of fluorine transfer with DFT provided a detailed reaction coordinate that explains the observed reactivity. The overall reaction and mechanistic insights should provide chemists a more predictable approach to site-selective fluorination of C–H bonds.

## Conflicts of interest

The authors declare no conflicts of interest.

## Supplementary Material

SC-011-C9SC04055B-s001
