# Supplementary material for: N-Directed fluorination of unactivated Csp3–H bonds
Source: Chem Sci. 2019 Dec 16;11(4):1102–6. doi: 10.1039/c9sc04055b (PMC8146735; doi:10.1039/c9sc04055b)
Supplement: SC-011-C9SC04055B-s001 [file SC-011-C9SC04055B-s001.pdf]

Electronic Supplementary Material (ESI) for Chemical Science.
This journal is © The Royal Society of Chemistry 2019

N-Directed Fluorination of Unactivated C*sp*^{3}–H Bonds

Emily N. Pinter, Jenna E. Bingham, Deyaa. I. AbuSalim, and Silas P. Cook*

Department of Chemistry, Indiana University,

800 East Kirkwood Avenue, Bloomington, IN 47405-7102

Supporting Information

Table of Contents

General Methods. 2

Reaction Optimization 3

Experimental Procedures and Characterizations 6

Analysis of Crude Fluorine Transfer Reactions 42

Computational Details 44

- **I. Computational Methods** 44
- **II. Calculated 3D Geometries of the Fe Complexes Used in the Reaction Coordinate** 45
- **III. Calculated Energy Components** 48
- **IV. Cartesian Coordinates of the Minimized Geometries** 54

References 143

NMR Spectra 144

General Methods.

All the reactions were carried out in flame-dried glassware fitted with rubber septa under a nitrogen atmosphere unless otherwise stated. Analytical grade solvents and commercially available reagents were purchased from commercial sources and used directly without further purification unless otherwise stated. $Fe(OTf)_2$ was purchased from Strem Chemicals and stored under an inert atmosphere. THF, MeOH, and DME were purified according to the Grubbs procedure.[1] Thin-layer chromatography (TLC) was carried out on Merck 60 F254 precoated, glass silica gel plates which were visualized with either ultraviolet light or stained with $KMnO_4$. Flash chromatography was performed using ZEOprep 60 ECO 40-63 μm silica gel. Automated column chromatography was performed on a Biotage Isolera One using Biotage Snap Ultra cartridges (25g or 50g SiO_2) or a Yamazen Universal Premium column (55g SiO_2) collecting with full spectrum analysis between wavelengths 200-400nm and monitoring wavelengths 254nm and 280nm. ^{1}H-NMR and ^{13}C-NMR spectra were recorded at room temperature using a Varian I400 or VXR400 (^{1}H-NMR at 400 MHz and ^{13}C-NMR at 100 MHz) and Varian I500 (^{1}H-NMR at 500 MHz and ^{13}C-NMR at 125 MHz). ^{19}F-NMR spectra were recorded at room temperature using a Varian VXR400 (^{19}F-NMR at 376 MHz). Chemical shifts are reported in ppm with reference to solvent signals [^{1}H-NMR: $CDCl_3$ (7.26 ppm), CD_2Cl_2 (5.32 ppm); ^{13}C-NMR: $CDCl_3$ (77.16 ppm)]. ^{19}F-NMR was internally referenced to a fluorobenzene standard (-112.96 ppm in $CDCl_3$).[2] Signal patterns are indicated as s, singlet; d, doublet; t, triplet; q, quartet; and m, multiplet. Infrared spectra (IR) were obtained on a Bruker Tensor II FTIR Spectrometer analyzed as a thin film and recorded in wavenumbers (cm^{-1}). High Resolution Mass (HRMS) analysis was obtained using Electron Impact Ionization (EI) and reported as m/z (relative intensity) for the molecular ion [M], with Electrospray Ionization (ESI), or with Atmospheric Pressure Chemical Ionization (APCI) and reporting the molecular ion [M+H], [M+Na] or a suitable fragment ion.

Reaction Optimization

Table SI-1. *Optimization of temperature and concentration*

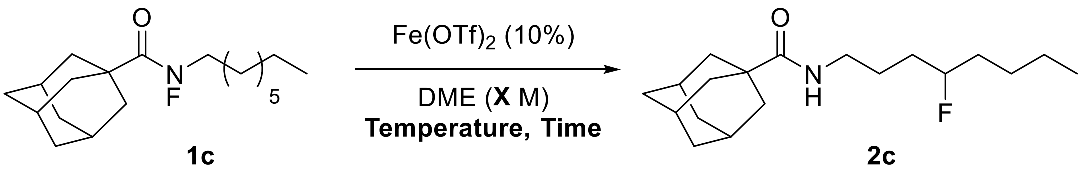

Entry	Temp (°C)	Time	Conc (M)	Yield (%)[a]
1[b]	rt	15 h	0.05	75
2	40	18 h	0.05	79
3	60	1 h	0.05	80
**4**	**80**	**20 min**	**0.05**	**81**
5	90	20 min	0.05	78
6	130	5 min	0.05	63
7	80	20 min	0.025	80
8	80	20 min	0.1	73
9	80	20 min	0.3	71

[a]Determined by ^{1}H NMR with 1,3,5-trimethoxybenzene as an internal standard. [b]Reaction ran inside glovebox.

Procedure: To a flame-dried vial with a stir bar was added fluoroamide (40.23 mg, 0.13 mmol, 1 equiv). The contents were evacuated and backfilled with N_2. The vial was capped and brought into a N_2 atmosphere glovebox. Anhydrous DME followed by anhydrous $Fe(OTf)_2$ (4.6 mg, 10 mol %) was added. The vial was capped, sealed with PTFE tape, and left to stir in the glovebox at room temperature or removed from the glovebox and stirred in an oil bath at the desired temperature until full consumption of starting material. The reaction was quenched with EtOAc (2 mL) and filtered through a 1-inch silica plug with ~15 mL EtOAc. The solution was concentrated by rotary evaporation and then analyzed by ^{1}H-NMR using 1,3,5-trimethoxybenzene as an internal standard.

Table SI-2. *Optimization of catalyst loading*

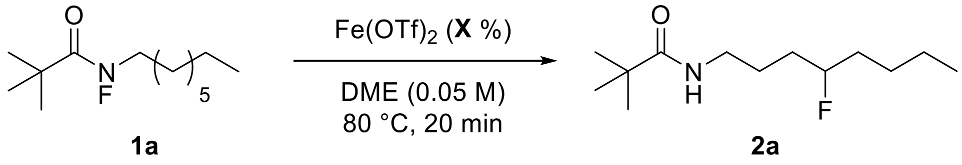

Entry	$Fe(OTf)_2$ (mol %)	Yield (%)[a]	Conversion (%)
1[b]	5	58	92
**2**	**10**	**62**	**100**
3	20	54	100
4	30	56	100

[a]Determined by ^{1}H-NMR with 1,3,5-trimethoxybenzene as an internal standard. [b]Reaction ran for 30 minutes.

Procedure: To a flame-dried vial with a stir bar was added fluoroamide (30.08 mg, 0.13 mmol, 1 equiv). The contents were evacuated and backfilled with N_2. The vial was capped and brought into a N_2 atmosphere glovebox. Anhydrous DME followed by anhydrous $Fe(OTf)_2$ (5-30 mol %) were added. The vial was capped, sealed with PTFE tape, and removed from the glovebox and stirred in an oil bath at 80 °C for 20 minutes. The reaction was quenched with EtOAc (2 mL) and filtered through a 1-inch silica plug with ~15 mL EtOAc. The solution was concentrated by rotary evaporation and then analyzed by ^{1}H-NMR using 1,3,5-trimethoxybenzene as an internal standard.

Table SI-3. *Optimization of solvent*

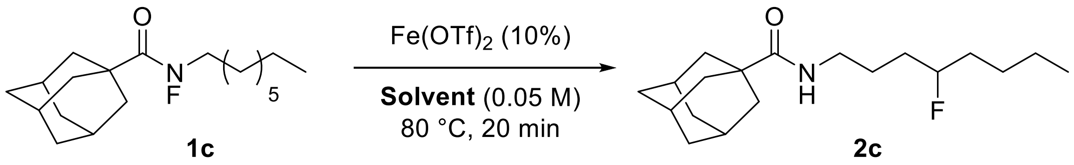

Entry	Solvent	Yield (%)[a]	Conversion (%)
1	2-MeTHF	8	28
2	EtOAc	9	20
3	THP	8	36
4	THF	38	65
**5**	**DME**	**81**	**100**
6	TBME	2	2

[a]Determined by ^{1}H NMR with 1,3,5-trimethoxybenzene as an internal standard.

Procedure: To a flame-dried vial with a stir bar was added fluoroamide (40.23 mg, 0.13 mmol, 1 equiv). The contents were evacuated and backfilled with N_2. Anhydrous solvent (2.6 mL, 0.05 M) was added to the fluoroamide by syringe. The vial was capped and brought into a N_2 atmosphere glovebox. Anhydrous Fe(OTf)$_2$ (4.6 mg, 10 mol %) was added, the vial was capped, sealed with PTFE tape, and removed from the glovebox and stirred in an oil bath at 80 °C for 20 minutes. The reaction was quenched with EtOAc (2 mL) and filtered through a 1-inch silica plug with ~15 mL EtOAc. The solution was concentrated by rotary evaporation and then analyzed by ^{1}H-NMR using 1,3,5-trimethoxybenzene as an internal standard. **Note:** Solvents were stored on sieves and degassed prior to use.

Experimental Procedures and Characterizations

Synthesis of Amides **General Procedure A**

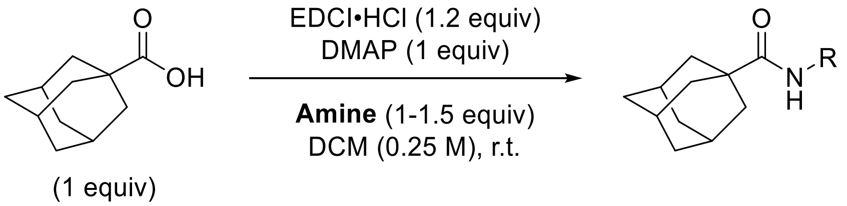

To a flame-dried round bottom flask equipped with a stir bar were added carboxylic acid (1 equiv), DMAP (1 equiv), EDCI•HCl (1.2 equiv), and DCM (0.25 M). The solution was stirred at room temperature until all solid dissolved. Amine (1-1.5 equiv) was added dropwise (or dropwise as a solution in 2 mL DCM) and the reaction was stirred at room temperature for 9-16 hours. The reaction was quenched with 1 M aqueous HCl (20 mL), diluted with DCM (75 mL) and water (50 mL), and extracted with DCM (3 x 75 mL). The combined organic layers were washed with brine, dried with $MgSO_4$, and concentrated by rotary evaporation. The crude amide was either quantitatively pure by NMR analysis or purified by silica flash column chromatography.

N-Octyl-2,2-dimethylpropanamide (SI-1)

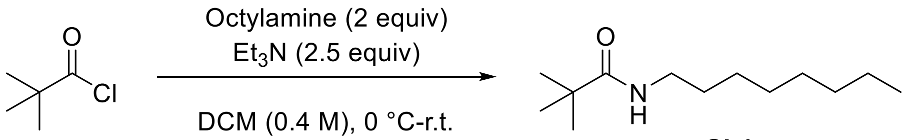

SI-1

A flame-dried round bottom flask was equipped with a stir bar, fitted with a rubber septum, evacuated and backfilled with N_2. To the flask were added DCM (30 mL) and pivaloyl chloride (1.5 mL, 12 mmol). Triethylamine (4.2 mL, 30 mmol), followed by octylamine (3.9 mL, 24 mmol) were added dropwise at room temperature. The reaction was stirred at room temperature for 3 hours. The reaction was quenched with 1 M aqueous HCl (20 mL), diluted with DCM (75 mL) and water (50 mL) and extracted with DCM (3 x 75 mL). The combined layers were washed with saturated $NaHCO_3$ (50 mL), brine (50 mL), dried with $MgSO_4$, and concentrated by rotary evaporation. No further purification was required and **SI-1** (2.080 g, 81% yield) was obtained as a pale yellow oil. ^{1}H and ^{13}C NMR spectra match those previously reported.[3]

<u>^{1}H-NMR</u> (400 MHz, $CDCl_3$): δ 5.61 (s, 1H), 3.26 – 3.15 (m, 2H) 1.52 – 1.42 (m, 2H), 1.32 – 1.21 (m, 10H), 1.18 (s, 9H), 0.87 (t, *J* = 7.1 Hz, 3H).
<u>^{13}C-NMR</u> (125 MHz, $CDCl_3$): δ 178.4, 39.7, 38.7, 31.9, 29.8, 29.4, 29.3, 27.8, 27.0, 22.8, 14.2.
<u>TLC:</u> R_f = 0.35, 20% EtOAc/hexanes (visualized with $KMnO_4$).

2,2-Dimethyl-*N*-octylbutanamide (SI-2)

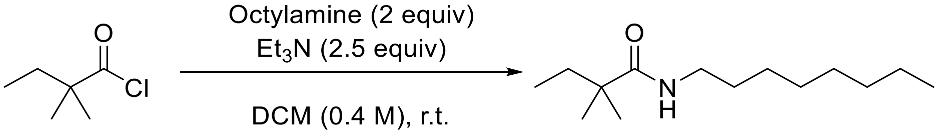

SI-2

To a flame-dried round bottom flask equipped with a stir bar and backfilled with N_2 were added DCM (24 mL) and 2,2-dimethylbutanoyl chloride (1.4 mL, 10 mmol). Triethylamine (3.5 mL, 25 mmol), followed by octylamine (3.3 mL, 20 mmol) were added dropwise at room temperature. The reaction was stirred at room temperature for 5 hours. The reaction was quenched with 1 M aqueous HCl (20 mL), diluted with DCM (75 mL) and water (50 mL) and extracted with DCM (3 x 75 mL). The combined layers were washed with $NaHCO_3$ then brine, dried with $MgSO_4$, and concentrated by rotary evaporation to give a crude orange oil. Purification by silica flash column chromatography (0-30% EtOAc/hexanes) provided **SI-2** (1.827 g, 80% yield**)** as a pale yellow oil.

^{1}H-NMR (400 MHz, $CDCl_3$): δ 5.64 (s, 1H), 3.25 – 3.15 (m, 2H), 1.55 – 1.40 (m, 4H), 1.33 – 1.17 (s, 10H), 1.11 (s, 6H), 0.89 – 0.77 (m, 6H).
^{13}C-NMR (125 MHz, $CDCl_3$): 177.5, 42.3, 39.5, 33.8, 31.7, 29.7, 29.3, 29.2, 26.9, 24.9, 22.6, 14.0, 9.1.
HRMS (ESI): Calcd. for $C_{14}H_{30}NO$ [M+H], 228.2322. Found: 228.2323.
FTIR (Film) cm^{-1}: 3341, 2925, 1635, 1535.
TLC: R_f = 0.26, 20% EtOAc/hexanes (visualized with $KMnO_4$).

N-Octyladamantane-1-carboxamide (SI-3)

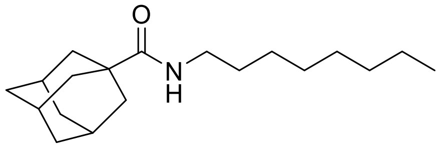

Octylamine (2.5 mL, 15 mmol) was subjected to General Procedure A to provide **SI-3** (3.691 g, 84% yield) as a white solid. The crude material was quantitatively pure by NMR analysis. ^{1}H and ^{13}C NMR spectra match those previously reported.[4]

^{1}H-NMR (500 MHz, $CDCl_3$): δ 5.56 (s, 1H), 3.25 – 3.17 (m, 2H), 2.06 – 2.00 (m, 3H), 1.87 – 1.80 (m, 6H), 1.76 – 1.65 (m, 6H), 1.51 – 1.42 (m, 2H), 1.33 – 1.19 (m, 10 H), 0.87 (t, J = 6.8 Hz, 3H).
^{13}C-NMR (125 MHz, $CDCl_3$): δ 177.9, 40.7, 39.5, 39.4, 36.7, 31.9, 29.8, 29.4, 29.3, 28.3, 27.0, 22.8, 14.2.
TLC: R_f = 0.56, 40% EtOAc/hexanes (visualized with $KMnO_4$).

N-Butyladamantane-1-carboxamide (SI-4)

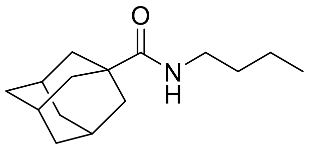

Butylamine (0.5 mL, 5 mmol) was subjected to General Procedure A to provide **SI-4** (1.086 g, 90% yield) as a white solid. The crude material was quantitatively pure by NMR analysis. ^{1}H and ^{13}C NMR spectra match those previously reported.[5]

^{1}H-NMR (500 MHz, $CDCl_3$): δ 5.55 (s, 1H), 3.26 – 3.19 (m, 2H), 2.06 – 2.00 (m, 3H), 1.87 – 1.80 (m, 6H), 1.77 – 1.65 (m, 6H), 1.51 – 1.42 (m, 2H), 1.38 – 1.28 (m, 2H), 0.92 (t, J = 7.4 Hz, 3H).
^{13}C-NMR (125 MHz, $CDCl_3$): δ 177.9, 40.7, 39.5, 39.1, 36.7, 31.9, 28.3, 20.2, 13.9.
TLC: R_f = 0.29, 20% EtOAc/hexanes (visualized with $KMnO_4$).

N-Pentyladamantane-1-carboxamide (SI-5)

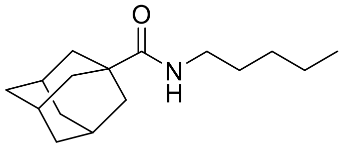

Amylamine (0.6 mL, 5 mmol) was subjected to General Procedure A to provide **SI-5** (1.206 g, 96% yield) as a white solid. The crude material was quantitatively pure by NMR analysis.

^{1}H-NMR (500 MHz, $CDCl_3$): δ 5.68 (s, 1H), 3.19 – 3.11 (m, 2H), 2.00 – 1.93 (m, 3H), 1.83 – 1.74 (m, 6H), 1.71 – 1.58 (m, 6H), 1.46 – 1.38 (m, 2H), 1.31 – 1.17 (m, 4H), 0.83 (t, J = 6.9 Hz, 3H).
^{13}C-NMR (125 MHz, $CDCl_3$): δ 177.8, 40.5, 39.3, 39.2, 36.6, 29.4, 29.1, 28.1, 22.4, 13.9.
HRMS (APCI): Calcd. for $C_{16}H_{28}NO$ [M+H], 250.2165. Found: 250.2168.
FTIR (Film) cm^{-1}: 3339, 2904, 2854, 1632, 1531.
TLC: R_f = 0.27, 20% EtOAc/hexanes (visualized with $KMnO_4$).

***N*-Hexyladamantane-1-carboxamide (SI-6)**

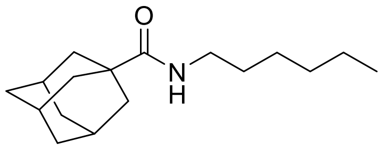

Hexylamine (2.0 mL, 15 mmol, 1.5 equiv) was subjected to General Procedure A to provide **SI-6** (2.063 g, 78% yield) as a white solid. The crude material was quantitatively pure by NMR analysis. ^{1}H and ^{13}C NMR spectra match those previously reported.[6]

^{1}H-NMR (500 MHz, $CDCl_3$): δ 5.55 (s, 1H), 3.25 – 3.19 (m, 2H), 2.06 – 2.01 (m, 3H), 1.86 – 1.81 (m, 6H), 1.77 – 1.66 (m, 6H), 1.52 – 1.42 (m, 2H), 1.35 – 1.23 (m, 6H), 0.88 (t, *J* = 7.2 Hz, 3H).
^{13}C-NMR (125 MHz, $CDCl_3$): δ 177.9, 40.7, 39.5, 39.4, 36.7, 31.6, 29.8, 28.3, 26.7, 22.7, 14.2.
TLC: R_f = 0.23, 20% EtOAc/hexanes (visualized with $KMnO_4$).

***N*-Decyladamantane-1-carboxamide (SI-7)**

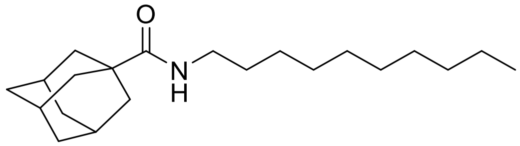

Decylamine (1.587 g, 10 mmol) was subjected to General Procedure A to provide **SI-7** (2.957 g, 93% yield) as a white solid. The crude material was quantitatively pure by NMR analysis.

^{1}H-NMR (500 MHz, $CDCl_3$): δ 5.55 (s, 1H), 3.25 – 3.17 (m, 2H), 2.07 – 1.99 (m, 3H), 1.87 – 1.80 (m, 6H), 1.77 – 1.65 (m, 6H), 1.52 – 1.42 (m, 2H), 1.35 – 1.18 (m, 14H), 0.87 (t, *J* = 6.9 Hz, 3H).
^{13}C-NMR (125 MHz, $CDCl_3$): δ 177.9, 40.7, 39.5, 39.4, 36.7, 32.0, 29.8, 29.7, 29.4, 28.3, 27.0, 22.8, 14.2.
HRMS (APCI): Calcd. for $C_{21}H_{38}NO$ [M+H], 320.2948. Found: 320.2952.
FTIR (Film) cm^{-1}: 3336, 2921, 2851, 1633, 1531.
TLC: R_f = 0.30, 20% EtOAc/hexanes (visualized with $KMnO_4$).

***N*-Isopentyladamantane-1-carboxamide (SI-8)**

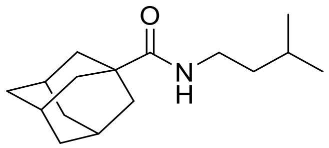

Isopentylamine (1.2 mL, 10 mmol) was subjected to General Procedure A to provide **SI-8** (2.123 g, 85% yield) as a white solid. The crude material was quantitatively pure by NMR analysis.

^{1}H-NMR (500 MHz, $CDCl_3$): δ 5.50 (s, 1H), 3.28 – 3.22 (m, 2H), 2.07 – 2.01 (m, 3H), 1.87 – 1.82 (m, 6H), 1.77 – 1.66 (m, 6H), 1.65 – 1.55 (m, 1H), 1.41 – 1.34 (m, 2H), 0.91 (d, *J* = 6.7 Hz, 6H).
^{13}C-NMR (125 MHz, $CDCl_3$): δ 177.9, 40.7, 39.5, 38.7, 37.7, 36.7, 28.3, 26.1, 22.6.
HRMS (APCI): Calcd. for $C_{16}H_{28}NO$ [M+H], 250.2165. Found: 250.2167.
FTIR (Film) cm^{-1}: 3328, 2904, 1629, 1540.
TLC: R_f = 0.20, 20% EtOAc/hexanes (visualized with $KMnO_4$).

***N*-(Heptan-2-yl)adamantane-1-carboxamide (SI-9)**

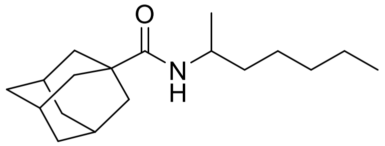

2-Aminoheptane (1.5 mL, 10 mmol) was subjected to General Procedure A to provide **SI-9** (2.742 g, 89% yield) as a white solid. The crude material was quantitatively pure by NMR analysis.

^{1}H-NMR (500 MHz, $CDCl_3$): δ 5.34 – 5.22 (m, 1H), 4.02 – 3.91 (m, 1H), 2.07 – 2.01 (m, 3H), 1.86 – 1.81 (m, 6H), 1.77 – 1.67 (m, 6H), 1.43 – 1.36 (m, 2H), 1.34 – 1.22 (m, 6H), 1.09 (d, *J* = 6.6 Hz, 3H), 0.88 (t, *J* = 6.8 Hz, 3H).
^{13}C-NMR (125 MHz, $CDCl_3$): δ 177.1, 44.7, 40.6, 39.4, 37.1, 36.7, 31.8, 28.3, 25.8, 22.6, 21.1, 14.1.
HRMS (APCI): Calcd. for $C_{18}H_{32}NO$ [M+H], 278.2478. Found: 278.2482.
FTIR (Film) cm^{-1}: 3310, 2900, 2850, 1628, 1546.
TLC: R_f = 0.43, 20% EtOAc/hexanes (visualized with $KMnO_4$).

***N*-(6-Methylheptan-2-yl)adamantane-1-carboxamide (SI-10)**

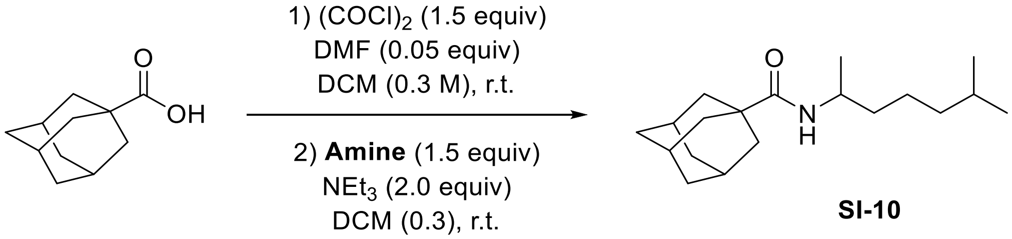

To a flame-dried round bottom flask equipped with a stir bar was added 1-adamantanecarboxylic acid (1812.4 mg, 10 mmol). The flask was fitted with a rubber septum and the contents were evacuated and backfilled with N_2. DCM (33 mL) was added, followed by DMF (0.38 mL, 0.5 mmol). Oxalyl chloride (1.3 mL, 15 mmol) was added dropwise at room temperature. The reaction was stirred until bubbling stopped (50 minutes). Volatile components were removed via rotary evaporation to give a crude yellow solid. The crude acid chloride was dissolved in DCM (33 mL) and 2-amino-6-methylheptane (2.5 mL, 15 mmol), followed by triethylamine (2.8 mL, 20 mmol) were added dropwise. The reaction was stirred at room temperature for 3 hours. The reaction was quenched with 1 M aqueous HCl (20 mL), diluted with DCM (75 mL) and water (50 mL) and extracted with DCM (3 x 75 mL). The combined layers were washed with brine, dried with $MgSO_4$, and concentrated by rotary evaporation. No further purification was required and **SI-10** (2.547 g, 87% yield) was obtained as a white solid.

^{1}H-NMR (500 MHz, $CDCl_3$): δ 5.36 – 5.22 (m, 1H), 4.02 – 3.90 (m, 1H), 2.07 – 2.00 (m, 3H), 1.87 – 1.80 (m, 6H), 1.77 – 1.66 (m, 6H), 1.56 – 1.46 (m, 1H), 1.42 – 1.34 (m, 2H), 1.32 – 1.24 (m, 2H), 1.22 – 1.06 (m, 5H), 0.86 (t, *J* = 6.6 Hz, 6H).
^{13}C-NMR (125 MHz, $CDCl_3$): δ 177.2, 44.8, 40.6, 39.5, 38.9, 37.4, 36.7, 28.4, 28.0, 23.9, 22.8, 22.7, 21.2.
HRMS (APCI): Calcd. for $C_{19}H_{34}NO$ [M+H], 292.2635. Found: 292.2638.
FTIR (Film) cm^{-1}: 3307, 2900, 2850, 1628, 1545.
TLC: R_f = 0.36, 20% EtOAc/hexanes (visualized with $KMnO_4$).

***N*-(2-Cyclohexylethyl)adamantane-1-carboxamide (SI-11)**

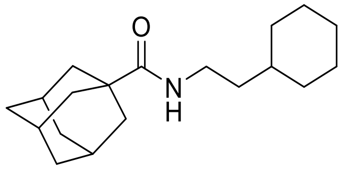

2-Cyclohexyl-ethylamine (1.3 mL, 10 mmol) was subjected to General Procedure A. Purification by silica flash column chromatography (10-40% EtOAc/hexanes) provided **SI-11** (1.864 g, 64% yield) as a white solid.

^{1}H-NMR (500 MHz, $CDCl_3$): δ 5.49 (s, 1H), 3.28 – 3.22 (m, 2H), 2.06 – 2.00 (m, 3H), 1.87 – 1.80 (m, 6H), 1.78 – 1.58 (m, 11H), 1.41 – 1.34 (m, 2H), 1.32 – 1.09 (m, 4H), 0.97 – 0.86 (m, 2H).
^{13}C-NMR (125 MHz, $CDCl_3$): δ 177.7, 40.5, 39.3, 37.2, 37.1, 36.6, 35.6, 33.2, 28.2, 26.5, 26.2.
HRMS (APCI): Calcd. for $C_{19}H_{32}NO$ [M+H], 290.2478. Found: 290.2482.
FTIR (Film) cm^{-1}: 3340, 2904, 2849, 1632, 1532.
TLC: R_f = 0.26, 20% EtOAc/hexanes (visualized with $KMnO_4$).

***N*-Cycloheptyladamantane-1-carboxamide (SI-12)**

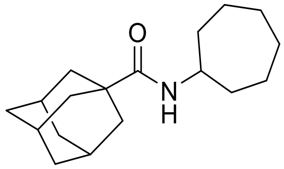

Cycloheptylamine (1.149 g, 10 mmol) was subjected to General Procedure A to provide **SI-12** (2.507 g, 91% yield) as a white solid. The crude material was quantitatively pure by NMR analysis.

^{1}H-NMR (400 MHz, $CDCl_3$): δ 5.48 (s, 1H), 3.98 – 3.87 (m, 1H), 2.07 – 2.00 (m, 3H), 1.95 – 1.79 (m, 8H), 1.77 – 1.34 (m, 16H).
^{13}C-NMR (125 MHz, $CDCl_3$): δ 176.8, 49.9, 40.5, 39.4, 36.7, 35.3, 28.3, 28.1, 24.4.
HRMS (APCI): Calcd. for $C_{18}H_{30}NO$ [M+H], 276.2322. Found: 276.2325.
FTIR (Film) cm^{-1}: 3305, 2902, 1626, 1544.
TLC: R_f = 0.35, 20% EtOAc/hexanes (visualized with $KMnO_4$).

N-(4-Methylpentyl)adamantane-1-carboxamide (SI-13)

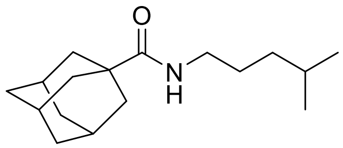

4-Methylpentan-1-amine (920.1 mg, 9 mmol) was subjected to General Procedure A to provide **SI-13** (2.133 g, 90% yield) as a white solid. The crude material was quantitatively pure by NMR analysis.

^{1}H-NMR (400 MHz, $CDCl_3$): δ 5.58 (s, 1H), 3.24 – 3.14 (m, 2H), 2.07 – 1.97 (m, 3H), 1.88 – 1.79 (m, 6H), 1.77 – 1.63 (m, 6H), 1.59 – 1.40 (m, 3H), 1.21 – 1.12 (m, 2H), 0.86 (d, J = 6.6 Hz, 6H).
^{13}C-NMR (125 MHz, $CDCl_3$): δ 177.8, 40.6, 39.6, 39.4, 36.6, 36.2, 28.2, 27.8, 27.6, 22.6.
HRMS (APCI): Calcd. for $C_{17}H_{30}NO$ [M+H], 264.2322. Found: 264.2326.
FTIR (Film) cm^{-1}: 3338, 2902, 1630, 1451, 1283.
TLC: R_f = 0.24, 20% EtOAc/hexanes (visualized with $KMnO_4$).

N-(4-Phenylbutyl)adamantane-1-carboxamide (SI-14)

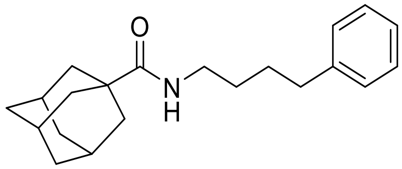

4-Phenylbutylamine (1.6 mL, 10 mmol) was subjected to General Procedure A to provide **SI-14** (2.764 g, 88% yield) as a white solid. The crude material was quantitatively pure by NMR analysis. ^{1}H and ^{13}C NMR spectra match those previously reported.[7]

^{1}H-NMR (500 MHz, $CDCl_3$): 7.31 – 7.24 (m, 2H), 7.21 – 7.15 (m, 3H), 5.52 (s, 1H), 3.30 – 3.22 (m, 2H), 2.63 (t, J = 7.6 Hz, 2H). 2.06 – 2.00 (m, 3H), 1.86 – 1.80 (m, 6H), 1.77 – 1.61 (m, 8H), 1.56 – 1.48 (m, 2H).
^{13}C-NMR (125 MHz, $CDCl_3$): 177.9, 142.2, 128.4, 128.3, 125.8, 40.6, 39.3, 39.1, 36.6, 35.5, 29.3, 28.6, 28.2.
TLC: R_f = 0.22, 20% EtOAc/hexanes.

N-(6-Hydroxyhexyl)adamantane-1-carboxamide (SI-15)

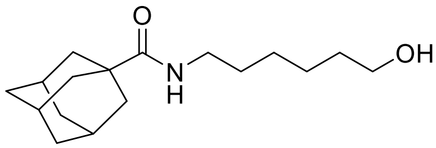

6-Amino-1-hexanol (3.568 g, 30 mmol) was subjected to General Procedure A to provide **SI-15** (7.338 g, 87% yield) as a white solid. The crude material was quantitatively pure by NMR analysis. ^{1}H and ^{13}C NMR spectra match those previously reported.[8]

^{1}H-NMR (500 MHz, $CDCl_3$): δ 5.58 (s, 1H), 3.63 (t, *J* = 6.6 Hz, 2H), 3.27 – 3.21 (m, 2H), 2.06 – 2.00 (m, 3H), 1.85 – 1.81 (m, 6H), 1.77 – 1.66 (m, 6H), 1.65 – 1.45 (m, 5H), 1.43 – 1.28 (m, 4H).
^{13}C-NMR (125 MHz, $CDCl_3$): δ 178.2, 62.6, 40.7, 39.4, 39.1, 36.7, 32.7, 29.8, 28.3, 26.5, 25.3.
TLC: R_f = 0.33, 30% acetone/hexanes (visualized with $KMnO_4$).

N-(6-((Tetrahydro-2H-pyran-2-yl)oxy)hexyl)adamantane-1-carboxamide (SI-16)

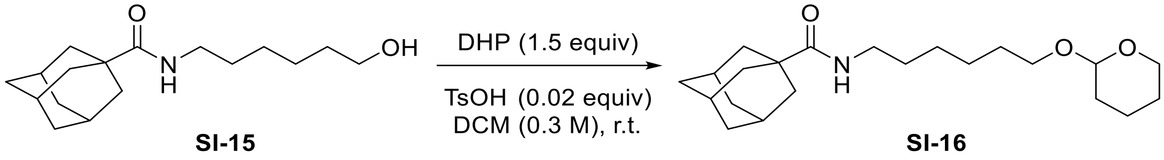

To a flame-dried round bottom flask equipped with a stir bar were added *N*-(6-hydroxyhexyl)adamantane-1-carboxamide **SI-15** (2.336 g, 8.4 mmol) and DCM (30 mL). 3,4-Dihydro-2H-pyran (1.2 mL,12.5 mmol), followed by *p*-toluenesulfonic acid monohydrate (28.1 mg, 0.16 mmol) were added and the reaction stirred at room temperature for 16 hours. The reaction was quenched with saturated aqueous $NaHCO_3$ (10 mL), diluted with DCM (75 mL) and water (50 mL), and extracted with DCM (3 x 75 mL). The combined organic layers were washed with brine, dried with $MgSO_4$, and concentrated by rotary evaporation to yield a crude orange oil. Purification by silica flash column chromatography (10-60% acetone/hexanes) provided **SI-16** (2.847 g, 93% yield**)** as a sticky white solid.

^{1}H-NMR (500 MHz, $CDCl_3$): δ 5.55 (s, 1H), 4.57 – 4.53 (m, 1H), 3.89 – 3.81 (m, 1H), 3.72 (dt, *J* = 9.6, 6.8 Hz, 1H), 3.52 – 3.46 (m, 1H), 3.37 (dt, *J* = 9.6, 6.7 Hz, 1H), 3.24 – 3.19 (m, 2H), 2.06 – 1.99 (m, 3H), 1.87 – 1.65 (m, 14H), 1.63 – 1.44 (m, 8H), 1.42 – 1.28 (m, 4H).
^{13}C-NMR (125 MHz, $CDCl_3$): δ 177.9, 99.0, 67.6, 62.5, 40.7, 39.5, 39.4, 36.7, 30.9, 29.8, 29.7, 28.3, 26.9, 26.1, 25.6, 19.9.
HRMS (APCI): Calcd. for $C_{22}H_{38}NO_3$ [M+H], 364.2846. Found: 364.2847.
FTIR (Film) cm^{-1}: 3344, 2903, 2850, 1632, 1529, 1451, 1031.
TLC: R_f = 0.54, 30% acetone/hexanes (visualized with $KMnO_4$).

N-(5-Hydroxypentyl)adamantane-1-carboxamide (SI-17)

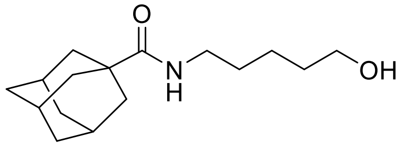

5-Amino-1-pentanol (1.059 g, 10 mmol) was subjected to General Procedure A to provide **SI-17** (2.504 g, 94% yield) as a white solid. The crude material was quantitatively pure by NMR analysis. ^{1}H and ^{13}C NMR spectra match those previously reported.[9]

<u>^{1}H-NMR</u> (500 MHz, $CDCl_3$): δ 5.62 (s, 1H), 3.64 (t, J = 6.4 Hz, 2H), 3.27 – 3.21 (m, 2H), 2.06 – 2.01 (m, 3H), 1.87 – 1.80 (m, 6H), 1.78 – 1.48 (m, 11H), 1.43 – 1.34 (m, 2H).
<u>^{13}C-NMR</u> (125 MHz, $CDCl_3$): δ 178.2, 62.5, 40.7, 39.4, 39.2, 36.6, 32.3, 29.5, 28.2, 23.1.
<u>TLC:</u> R_f = 0.13, 30% acetone/hexanes (visualized with $KMnO_4$).

N-(5-((Tetrahydro-2H-pyran-2-yl)oxy)pentyl)adamantane-1-carboxamide (SI-18)

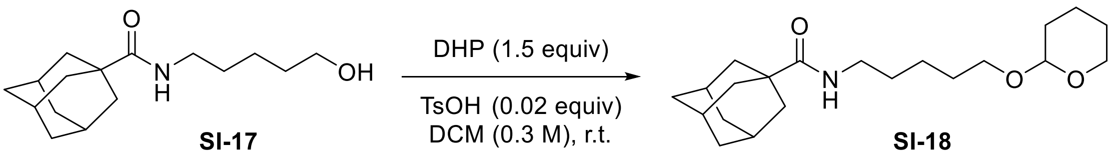

To a flame-dried round bottom flask equipped with a stir bar were added N-(5-hydroxypentyl)adamantane-1-carboxamide **SI-17** (723.5 mg, 2.7 mmol) and DCM (10 mL). 3,4-Dihydro-2H-pyran (0.4 mL,4 mmol), followed by *p*-toluenesulfonic acid monohydrate (10 mg, 0.05 mmol) were added and the reaction stirred at room temperature for 16 hours. The reaction was quenched with saturated aqueous $NaHCO_3$ (5 mL), diluted with DCM (50 mL) and water (25 mL), and extracted with DCM (3 x 50 mL). The combined organic layers were washed with brine, dried with $MgSO_4$, and concentrated by rotary evaporation to yield a crude orange oil. Purification by silica flash column chromatography (10-60% acetone/hexanes) provided **SI-18** (840 mg, 88% yield**)** as a sticky white solid.

<u>^{1}H-NMR</u> (500 MHz, $CDCl_3$): δ 5.57 (s, 1H), 4.58 – 4.53 (m, 1H), 3.89 – 3.82 (m, 1H), 3.73 (dt, J = 9.7, 6.7 Hz, 1H), 3.53 – 3.46 (m, 1H), 3.38 (dt, J = 9.6, 6.4 Hz, 1H), 3.27 – 3.21 (m, 2H), 2.06 – 2.00 (m, 3H), 1.89 – 1.77 (m, 7H), 1.76 – 1.65 (m, 7H), 1.64 – 1.47 (m, 8H), 1.43 – 1.35 (m, 2H).
<u>^{13}C-NMR</u> (125 MHz, $CDCl_3$): δ 177.9, 99.1, 67.5, 62.6, 40.7, 39.5, 39.3, 36.7, 30.9, 29.6, 29.5, 28.3, 25.6, 23.8, 19.9.
<u>HRMS</u> (APCI): Calcd. for $C_{21}H_{36}NO_3$ [M+H], 350.2690. Found: 350.2689.
<u>FTIR</u> (Film) cm^{-1}: 3337, 2904, 2850, 1633, 1529, 1032.
<u>TLC:</u> R_f = 0.41, 30% acetone/hexanes (visualized with $KMnO_4$).

Synthesis of N-fluoroamides General Procedure B

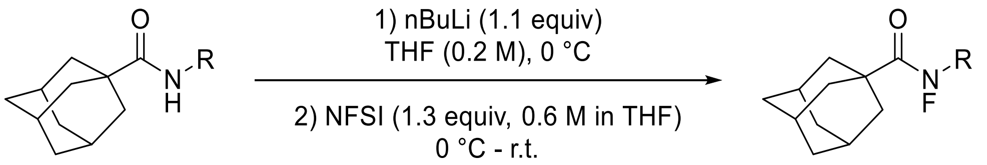

Fluorinations were typically conducted on 5-10 mmol scale.

To a flame-dried round-bottom flask with a stir bar was added amide (1.0 equiv). The contents were evacuated and backfilled with N_2. Anhydrous THF (0.2 M) was added and the solution stirred until all solid dissolved. The solution was cooled to 0 °C on an ice bath for 10 minutes. *n*-Butyllithium (1.1 equiv, 2.1-2.5 M in hexanes) was added dropwise, and the reaction stirred at 0 °C for 1 hour. *N*-Fluorobenzenesulfonimide (NFSI) (1.3 equiv) in THF (0.6 M) was added dropwise via syringe pump over 1 hour at 0 °C. The reaction stirred for 14 h, slowly warming to room temperature as the ice bath melted. The reaction was quenched with 1 M aqueous HCl (20 mL), diluted with DCM (75 mL) and water (50 mL), and extracted with DCM (3 x 75 mL). The combined organic layers were washed with brine, dried with $MgSO_4$, and concentrated by rotary evaporation. The crude mixture was purified by silica flash column chromatography.

_N_-Fluoro-_N_-octylpivalamide (1a)

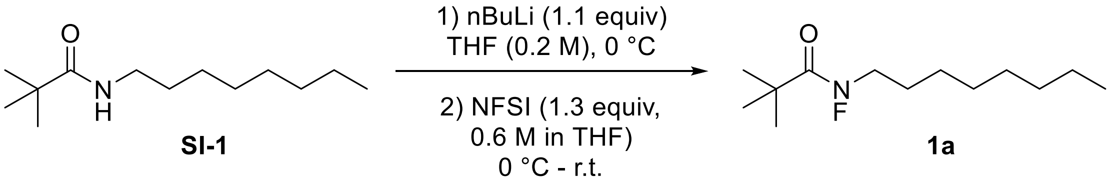

N-Octyl-2,2-dimethylpropanamide **SI-1** (2.012 g, 9.4 mmol) was subjected to General Procedure B. Purification by silica flash column chromatography (0-25% EtOAc/hexanes) provided **1a** (652.2 mg, 30% yield) as a yellow oil.

<u>^{1}H-NMR</u> (500 MHz, $CDCl_3$): δ 3.79 (dt, *J* = 33.0, 7.2 Hz, 2H), 1.70 – 1.61 (m, 2H), 1.37 – 1.21 (m, 19H), 0.87 (t, *J* = 6.9 Hz, 3H).
<u>^{13}C-NMR</u> (125 MHz, $CDCl_3$): δ 181.2 (d, *J* = 2.5 Hz), 51.1 (d, *J* = 11.3 Hz), 39.9 (d, *J* = 2.9 Hz), 31.9, 29.4, 29.3, 26.7, 26.6 (d, *J* = 5.6 Hz), 26.4 (d, *J* = 2.6 Hz), 22.8, 14.2.
<u>^{19}F-NMR</u> (376 MHz, $CDCl_3$): δ -67.6 (t, *J* = 33.0 Hz).
<u>HRMS</u> (ESI): Calcd. for $C_{13}H_{26}FNONa$ [M+Na], 254.1896. Found: 254.1891.
<u>FTIR</u> (Film) cm^{-1}: 2926, 2872, 1682, 1462.
<u>TLC:</u> R_f = 0.67, 20% EtOAc/hexanes.

N-Fluoro-2,2-dimethyl-N-octylbutanamide (1b)

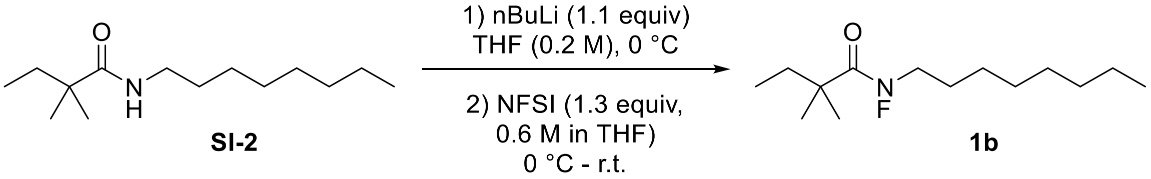

2,2-Dimethyl-*N*-octylbutanamide **SI-2** (1.743 g, 7.6 mmol) was subjected to General Procedure B. Purification by silica flash column chromatography (0-30% acetone/hexanes) provided **1b** (447.3 mg, 23% yield) as a yellow oil.

^{1}H-NMR (500 MHz, $CDCl_3$): δ 3.79 (dt, *J* = 33.2, 7.2 Hz, 2H), 1.70 – 1.59 (m, 4H), 1.38 – 1.18 (m, 16H), 0.90 – 0.83 (m, 6H).
^{13}C-NMR (125 MHz, $CDCl_3$): δ 180.6 (d, *J* = 2.7 Hz). 51.1 (d, *J* = 11.2 Hz), 44.1 (d, *J* = 3.3 Hz), 32.0 (d, *J* = 6.6 Hz), 31.9, 29.3, 26.7, 26.4 (d, *J* = 2.4 Hz), 24.7, 24.6, 22.8, 14.2, 9.4.
^{19}F-NMR (376 MHz, $CDCl_3$): δ -68.5 (t, *J* = 33.2 Hz).
HRMS (APCI): Calcd. for $C_{14}H_{29}FNO$ [M+H], 246.2228. Found: 246.2229.
TLC: FTIR (Film) cm^{-1}: 2926, 1681, 1461, 1171.
TLC: R_f = 20% EtOAc/hexanes.

N-Fluoro-N-octyladamantane-1-carboxamide (1c)

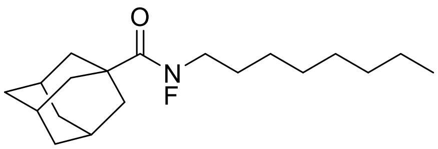

N-Octyladamantane-1-carboxamide **SI-3** (3.691 g, 12.6 mmol) was subjected to General Procedure B. Purification by silica flash column chromatography (0-30% EtOAc/hexanes) provided **1c** (1.186 g, 30% yield) as a yellow oil.

^{1}H-NMR (400 MHz, $CDCl_3$): δ 3.77 (dt, *J* = 33.3, 7.4 Hz, 2H), 2.07 – 1.95 (m, 9H), 1.77 – 1.69 (m, 6H), 1.67 – 1.59 (m, 2H), 1.37 – 1.20 (m, 10H), 0.87 (t, *J* = 7.3 Hz, 3H).
^{13}C-NMR (125 MHz, $CDCl_3$): δ 180.7 (d, *J* = 3.5 Hz), 51.2 (d, *J* = 11.1 Hz), 42.7 (d, *J* = 3.3 Hz), 37.6 (d, *J* = 5.6 Hz), 36.8, 31.9, 29.3, 29.2, 28.3, 26.7, 26.4 (d, *J* = 2.7 Hz), 22.8, 14.2.
^{19}F-NMR (376 MHz, $CDCl_3$): δ -70.5 (t, *J* = 33.2 Hz).
HRMS (EI): Calcd. for $C_{19}H_{31}FNO$ [M-H], 308.2390. Found: 308.2384.
FTIR (Film) cm^{-1}: 2906, 2851, 1676, 1454.
TLC: R_f = 0.62, 20% EtOAc/hexanes.

_N_-Butyl-_N_-fluoroadamantane-1-carboxamide (1d)

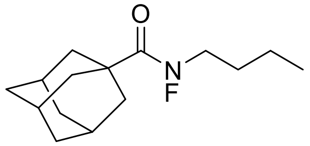

N-Butyladamantane-1-carboxamide **SI-4** (1.056 g, 4.5 mmol) was subjected to General Procedure B. Purification by silica flash column chromatography (0-25% EtOAc/hexanes) provided **1d** (315.3 mg, 27% yield) as a yellow oil.

^{1}H-NMR (500 MHz, $CDCl_3$): δ 3.79 (dt, *J* = 33.3, 7.1 Hz, 2H), 2.06 – 1.96 (m, 9H), 1.76 – 1.69 (m, 6H), 1.67 – 1.58 (m, 2H), 1.40 – 1.31 (m, 2H), 0.93 (t, *J* = 7.4 Hz, 3H).
^{13}C-NMR (125 MHz, $CDCl_3$): δ 180.7 (d, *J* = 3.6 Hz), 50.9 (d, *J* = 11.1 Hz), 42.7 (d, *J* = 3.5 Hz), 37.6 (d, *J* = 5.2 Hz), 36.8, 28.5 (d, *J* = 2.7 Hz), 28.3, 19.9, 13.8.
^{19}F-NMR (376 MHz, $CDCl_3$): δ -70.5 (t, *J* = 33.4 Hz).
HRMS (APCI): Calcd. for $C_{15}H_{25}FNO$ [M+H], 254.1915. Found: 254.1916.
FTIR (Film) cm^{-1}: 2905, 2850, 1674, 1454, 1220.
TLC: R_f = 0.57, 20% EtOAc/hexanes.

_N_-Fluoro-_N_-pentyladamantane-1-carboxamide (1e)

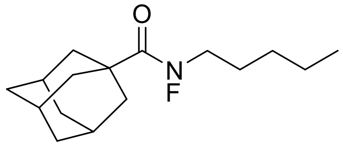

N-Pentyladamantane-1-carboxamide **SI-5** (1.206 g, 4.8 mmol) was subjected to General Procedure B. Purification by silica flash column chromatography (0-25% EtOAc/hexanes) provided **1e** (582.0 mg, 45% yield) as a yellow oil.

^{1}H-NMR (500 MHz, $CDCl_3$): δ 3.78 (dt, *J* = 33.1, 7.2 Hz, 2H), 2.05 – 1.97 (m, 9H). 1.76 – 1.69 (m, 6H), 1.68 – 1.58 (m, 2H), 1.39 – 1.25 (m, 4H), 0.90 (t, *J* = 7.2 Hz, 3H).
^{13}C-NMR (125 MHz, $CDCl_3$): δ 180.7 (d, *J* =3.3 Hz), 51.2 (d, *J* = 11.2 Hz), 42.7 (d, *J* = 3.4 Hz), 37.6 (d, *J* = 5.4 Hz), 36.8, 28.9, 28.3, 26.1 (d, *J* = 2.8 Hz), 22.4, 14.1.
^{19}F-NMR (376 MHz, $CDCl_3$): δ -70.5 (t, *J* = 33.3 Hz).
HRMS (APCI): Calcd. for $C_{16}H_{27}FNO$ [M+H], 268.2071. Found: 268.2072.
FTIR (Film) cm^{-1}: 2905, 2857, 1674, 1454.
TLC: R_f = 0.65, 20% EtOAc/hexanes.

_N_-Fluoro-_N_-hexyladamantane-1-carboxamide (1f)

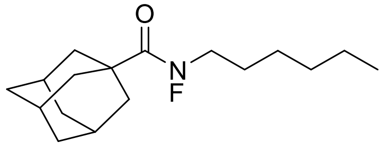

N-Hexyladamantane-1-carboxamide **SI-6** (2.063 g, 7.8 mmol) was subjected to General Procedure B. Purification by silica flash column chromatography (0-30% EtOAc/hexanes) provided **1f** (923.4 mg, 42% yield) as a yellow oil.

^{1}H-NMR (500 MHz, $CDCl_3$): δ 3.77 (dt, *J* = 33.2, 7.4 Hz, 2H), 2.05 – 1.96 (m, 9H), 1.75 – 1.69 (m, 6H), 1.67 – 1.59 (m, 2H), 1.36 – 1.25 (m, 6H), 0.88 (t, *J* = 7.2 Hz, 3H).
^{13}C-NMR (125 MHz, $CDCl_3$): δ 180.7 (d, *J* = 3.4 Hz), 51.2 (d, *J* = 11.5 Hz), 42.7 (d, *J* = 3.5 Hz), 37.6 (d, *J* = 5.4 Hz), 36.8, 31.5, 28.3, 26.4, 26.3, 22.6, 14.1.
^{19}F-NMR (376 MHz, $CDCl_3$): δ -70.5 (t, J = 33.2 Hz).
HRMS (APCI): Calcd. for $C_{17}H_{29}FNO$ [M+H], 282.2228. Found: 282.2230.
FTIR (Film) cm^{-1}: 2905, 2851, 1674, 1454, 1251.
TLC: R_f = 0.58, 20% EtOAc/hexanes.

_N_-Decyl-_N_-fluoroadamantane-1-carboxamide (1g)

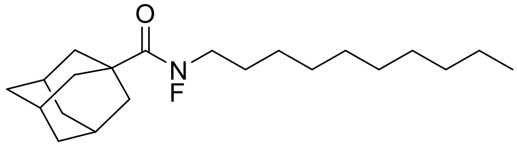

N-Decyladamantane-1-carboxamide **SI-7** (2.957 g, 9.3 mmol) was subjected to General Procedure B. Purification by silica flash column chromatography (0-30% EtOAc/hexanes) followed by a second column (0-25% EtOAc/hexanes) provided **1g** (1.092 g, 34% yield) as a yellow oil.

^{1}H-NMR (500 MHz, $CDCl_3$): δ 3.78 (dt, *J* = 33.1, 7.2 Hz, 2H), 2.07 – 1.95 (m, 9H), 1.77 – 1.69 (m, 6H), 1.68 – 1.59 (m, 2H), 1.37 – 1.19 (m, 14H), 0.88 (t, *J* = 7.1 Hz, 3H).
^{13}C-NMR (125 MHz, $CDCl_3$): δ 180.7 (d, *J* = 3.5 Hz), 51.2 (d, *J* = 11.2 Hz), 42.7 (d, *J* = 3.3 Hz), 37.6 (d, *J* = 5.4 Hz), 36.8, 32.0, 29.7, 29.6, 29.4, 29.3, 28.3, 26.7, 26.4 (d, *J* = 2.8 Hz), 22.8, 14.2.
^{19}F-NMR (376 MHz, $CDCl_3$): δ -70.5 (t, *J* = 33.1 Hz).
HRMS (APCI): Calcd. for $C_{21}H_{37}FNO$ [M+H], 338.2854. Found: 338.2856.
FTIR (Film) cm^{-1}: 2906, 2851, 1676, 1454.
TLC: R_f = 0.69, 20% EtOAc/hexanes.

_N_-Fluoro-_N_-isopentyladamantane-1-carboxamide (1h)

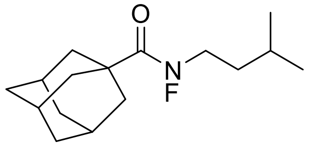

N-Isopentyladamantane-1-carboxamide **SI-8** (2.054 g, 8.2 mmol) was subjected to General Procedure B. Purification by silica flash column chromatography (0-25% EtOAc/hexanes) provided **1h** (915.4 mg, 41% yield) as a yellow oil.

^{1}H-NMR (500 MHz, $CDCl_3$): δ 3.80 (dt, *J* = 33.2, 7.4 Hz, 2H), 2.05 – 1.96 (m, 9H), 1.76 – 1.68 (m, 6H), 1.67 – 1.58 (m, 1H), 1.57 – 1.50 (m, 2H), 0.92 (d, *J* = 6.5 Hz, 6H).
^{13}C-NMR (125 MHz, $CDCl_3$): δ 180.8 (d, *J* = 3.5 Hz), 49.7 (d, *J* = 11.1 Hz), 42.7 (d, *J* = 3.5 Hz), 37.6 (d, *J* = 5.5 Hz), 36.8, 35.1 (d, *J* = 2.6 Hz), 28.3, 25.9, 22.5.
^{19}F-NMR (376 MHz, $CDCl_3$): δ -70.6 (t, *J* = 33.1 Hz).
HRMS (APCI): Calcd. for $C_{16}H_{27}FNO$ [M+H], 268.2071. Found: 268.2702.
FTIR (Film) cm^{-1}: 2905, 2850, 1675, 1454, 1222.
TLC: R_f = 0.55, 20% EtOAc/hexanes.

_N_-Fluoro-_N_-(heptan-2-yl)adamantane-1-carboxamide (1i)

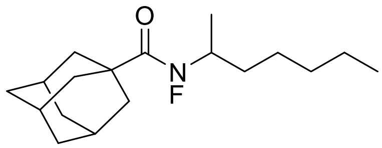

N-(Heptan-2-yl)adamantane-1-carboxamide **SI-9** (2.472 g, 9 mmol) was subjected to General Procedure B. Purification by silica flash column chromatography (0-30% EtOAc/hexanes) provided **1i** (988.2 mg, 37% yield) as a yellow oil.

^{1}H-NMR (400 MHz, $CDCl_3$): δ 4.59 – 4.36 (m, 1H), 2.06 – 1.93 (m, 9H), 1.77 – 1.60 (m, 7H), 1.52 – 1.42 (m, 1H), 1.37 – 1.23 (m, 6H), 1.19 (d, *J* = 6.7 Hz, 3H), 0.87 (t, *J* = 7.1 Hz, 3H).
^{13}C-NMR (125 MHz, $CDCl_3$): δ 180.5 (d, *J* = 3.4 Hz), 56.1 (d, *J* = 12.2 Hz), 42.7 (d, *J* = 3.4 Hz), 37.6 (d, *J* = 5.6 Hz), 36.8, 32.9 (d, *J* = 3.5 Hz), 31.6, 28.3, 26.1, 22.7, 16.9 (d, *J* = 5.8 Hz), 14.1.
^{19}F-NMR (376 MHz, $CDCl_3$): δ -96.9 (d, *J* = 42.2 Hz).
HRMS (APCI): Calcd. for $C_{18}H_{31}FNO$ [M+H], 296.2384. Found: 296.2386.
FTIR (Film) cm^{-1}: 2905, 2851, 1673, 1454.
TLC: R_f = 0.70, 20% EtOAc/hexanes.

_N_-Fluoro-_N_-(6-methylheptan-2-yl)adamantane-1-carboxamide (1j)

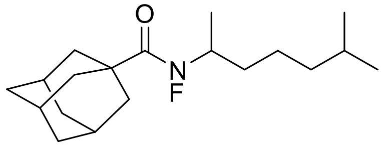

N-(6-Methylheptan-2-yl)adamantane-1-carboxamide **SI-10** (2.274 g, 7.8 mmol) was subjected to General Procedure B. Purification by silica flash column chromatography (0-30% EtOAc/hexanes) provided **1j** (1.150 g, 47% yield) as a yellow oil.

^{1}H-NMR (500 MHz, $CDCl_3$): δ 4.57 – 4.39 (m, 1H), 2.06 – 1.94 (m, 9H), 1.78 – 1.60 (m, 7H), 1.56 – 1.41 (m, 2H), 1.38 – 1.25 (m, 2H), 1.23 – 1.12 (m, 5H), 0.86 (d, *J* = 6.6 Hz, 6H).
^{13}C-NMR (125 MHz, $CDCl_3$): δ 180.5 (d, *J* = 3.4 Hz), 56.1 (d, *J* = 12.1 Hz), 42.8 (d, *J* = 3.4 Hz), 38.8, 37.6 (d, *J* = 5.6 Hz), 36.8, 33.2 (d, *J* = 3.5 Hz), 28.3, 28.0, 24.2, 22.7, 22.6, 17.0 (d, *J* = 5.8 Hz).
^{19}F-NMR (376 MHz, $CDCl_3$): δ -96.9 (d, *J* = 42.3 Hz).
HRMS (APCI): Calcd. for $C_{19}H_{33}FNO$ [M+H], 310.2541. Found: 310.2542.
FTIR (Film) cm^{-1}: 2905, 2850, 1673, 1454.
TLC: R_f = 0.60, 20% EtOAc/hexanes.

_N_-(2-Cyclohexylethyl)-_N_-fluoroadamantane-1-carboxamide (1k)

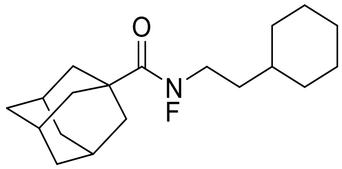

N-(2-Cyclohexylethyl)adamantane-1-carboxamide **SI-11** (1.864 g, 6.4 mmol) was subjected to General Procedure B. Purification by silica flash column chromatography (0-25% EtOAc/hexanes) provided **1k** (779.4 mg, 39% yield) as a yellow oil.

^{1}H-NMR (500 MHz, $CDCl_3$): δ 3.81 (dt, *J* = 33.2, 7.4 Hz, 2H), 2.06 – 1.96 (m, 9H), 1.79 – 1.61 (m, 11H), 1.57 – 1.51 (m, 2H), 1.35 – 1.09 (m, 4H), 0.98 – 0.87 (m, 2H).
^{13}C-NMR (125 MHz, $CDCl_3$): δ 180.8 (d, *J* = 3.6 Hz), 49.2 (d, *J* = 11.2 Hz), 42.6 (d, *J* = 3.4 Hz), 37.6 (d, *J* = 5.3 Hz), 36.7, 35.3, 33.6 (d, *J* = 2.5 Hz), 33.2, 28.2, 26.6, 26.3.
^{19}F-NMR (376 MHz, $CDCl_3$): δ -71.0 (t, *J* = 33.2 Hz).
HRMS (APCI): Calcd. for $C_{19}H_{31}FNO$ [M+H], 308.2384. Found: 308.2385.
FTIR (Film) cm^{-1}: 2905, 2849, 1675, 1448.
TLC: R_f = 0.62, 20% EtOAc/hexanes.

N-Cycloheptyl-N-fluoroadamantane-1-carboxamide (1l)

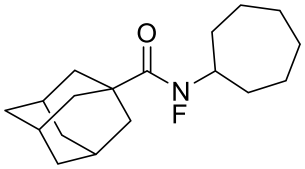

N-Cycloheptyladamantane-1-carboxamide **SI-12** (2.260 g, 8.2 mmol) was subjected to General Procedure B. Purification by silica flash column chromatography (0-30% EtOAc/hexanes) provided **1l** (944.8 mg, 39% yield) as a pale yellow solid.

^{1}H-NMR (500 MHz, $CDCl_3$): δ 4.51 – 4.34 (m, 1H), 2.05 – 1.95 (m, 9H), 1.93 – 1.66 (m, 12H), 1.63 – 1.41 (m, 6H).
^{13}C-NMR (125 MHz, $CDCl_3$): δ 180.7 (d, *J* = 3.0 Hz), 60.5 (d, *J* = 11.8 Hz), 42.7 (d, *J* = 3.1 Hz), 37.5 (d, *J* = 5.7 Hz), 36.8, 31.2 (d, *J* = 3.9 Hz), 28.4, 28.3, 25.1.
^{19}F-NMR (376 MHz, $CDCl_3$): δ -91.1 (d, *J* = 45.0 Hz).
HRMS (APCI): Calcd. for $C_{18}H_{29}FNO$ [M+H], 294.2228. Found: 294.2230.
FTIR (Film) cm^{-1}: 2906, 2851, 1673, 1454.
TLC: R_f = 0.60, 20% EtOAc/hexanes.

N-Fluoro-N-(4-methylpentyl)adamantane-1-carboxamide (1m)

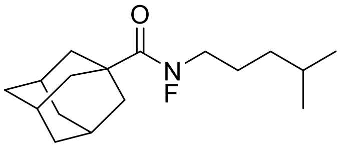

N-(4-Methylpentyl)adamantane-1-carboxamide **SI-13** (2.031 g, 7.7 mmol) was subjected to General Procedure B. Purification by silica flash column chromatography (0-25% EtOAc/hexanes) provided **1m** (731.6 mg, 33% yield) as a yellow oil.

^{1}H-NMR (500 MHz, $CDCl_3$): δ 3.76 (dt, *J* = 33.1, 7.2 Hz, 2H), 2.07 – 1.95 (m, 9H), 1.81 – 1.49 (m, 9H), 1.25 – 1.16 (m, 2H), 0.88 (d, *J* = 6.6 Hz, 6H).
^{13}C-NMR (125 MHz, $CDCl_3$): δ 180.7 (d, *J* = 3.7 Hz), 51.4 (d, *J* = 11.2 Hz), 42.6 (d, *J* = 3.5 Hz), 37.6 (d, *J* = 5.5 Hz), 36.7, 35.8, 28.2, 27.8, 24.2 (d, *J* = 2.8 Hz), 22.6.
^{19}F-NMR (376 MHz, $CDCl_3$): δ -70.4 (t, *J* = 33.1 Hz).
HRMS (APCI): Calcd. for $C_{17}H_{29}FNO$ [M+H], 282.2228. Found: 282.2229.
FTIR (Film) cm^{-1}: 2904, 2850, 1673, 1454.
TLC: R_f = 0.64, 20% EtOAc/hexanes.

_N_-Fluoro-_N_-(4-phenylbutyl)adamantane-1-carboxamide (1n)

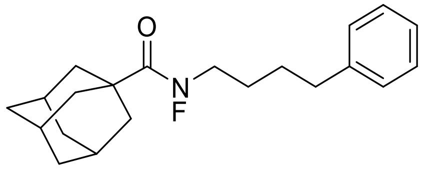

N-(4-Phenylbutyl)adamantane-1-carboxamide **SI-14** (2.764 g, 8.9 mmol) was subjected to General Procedure B. Purification by silica flash column chromatography (0-30% EtOAc/hexanes) provided **1n** (1.005 g, 34% yield) as a dark yellow oil.

^{1}H-NMR (500 MHz, CD_2Cl_2): δ 7.30 – 7.24 (m, 2H), 7.20 – 7.15 (m, 3H), 3.80 (dt, *J* = 33.6, 6.7 Hz, 2H), 2.64 (t, *J* = 7.2 Hz, 2H), 2.00 – 1.95 (m, 9H), 1.77 – 1.62 (m, 10H).
^{13}C-NMR (125 MHz, $CDCl_3$): δ 180.7 (d, *J* = 3.7 Hz), 142.1, 128.5, 128.4, 125.9, 50.9 (d, *J* = 11.1 Hz), 42.7 (d, *J* = 3.3 Hz), 37.6 (d, *J* = 5.4 Hz), 36.7, 35.5, 28.5, 28.2, 26.1 (d, *J* = 2.6 Hz).
^{19}F-NMR (376 MHz, $CDCl_3$): δ -70.2 (t, *J* = 33.1 Hz).
HRMS (APCI): Calcd. for $C_{21}H_{29}FNO$ [M+H], 330.2228. Found: 330.2229.
FTIR (Film) cm^{-1}: 2904, 2850, 1671, 1453, 697.
TLC: R_f = 0.61, 20% EtOAc/hexanes.

_N_-Fluoro-_N_-(6-hydroxyhexyl)adamantane-1-carboxamide (1o)

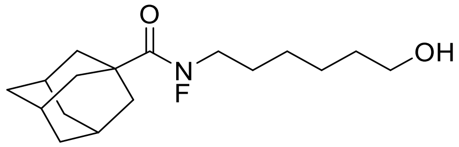

N-(6-((Tetrahydro-2H-pyran-2-yl)oxy)hexyl)adamantane-1-carboxamide **SI-16** (2.834 g, 7.8 mmol) was subjected to General Procedure B. Purification by silica flash column chromatography (0-30% acetone/hexanes) provided *N*-fluoro-*N*-(6-((tetrahydro-2H-pyran-2-yl)oxy)hexyl)adamantane-1-carboxamide **1p**, which was transferred to a flame-dried round bottom flask equipped with a stir bar and dissolved in MeOH (300 mL). *p*-Toluenesulfonic acid monohydrate (30.2 mg, 0.16 mmol) was added and the reaction was stirred at room temperature for 14 hours. The reaction was quenched with saturated aqueous $NaHCO_3$ (10 mL), diluted with DCM (75 mL) and water (50 mL), and extracted with DCM (3 x 75 mL). The combined organic layers were washed with brine, dried with $MgSO_4$, and concentrated by rotary evaporation to yield a crude yellow oil. Purification by silica flash column chromatography (10-50% acetone/hexanes) provided **1o** (477 mg, 20% yield over 2 steps**)** as a yellow oil.

^{1}H-NMR (500 MHz, $CDCl_3$): δ 3.79 (dt, *J* = 33.0, 7.1 Hz, 2H), 3.63 (t, *J* = 6.5 Hz, 2H), 2.06 – 1.96 (m, 9H), 1.77 – 1.63 (m, 8H), 1.61 – 1.53 (m, 2H), 1.44 – 1.31 (m, 5H).

^{13}C-NMR (125 MHz, $CDCl_3$): δ 180.7 (d, *J* = 3.6 Hz), 62.9, 50.9 (d, *J* = 11.3 Hz), 42.7 (d, *J* = 3.3 Hz), 37.6 (d, *J* = 5.4 Hz), 36.8, 32.7, 28.2, 26.4, 26.3, 25.4.
^{19}F-NMR (376 MHz, $CDCl_3$): δ -70.4 (t, *J* = 33.0 Hz).
HRMS (APCI): Calcd. for $C_{17}H_{27}FNO_2$ [M+H], 298.2177. Found: 298.2179.
FTIR (Film) cm^{-1}: 3368, 2904, 2851, 1671, 1454, 1345, 1054.
TLC: R_f = 0.46, 20% acetone/hexanes.

N-Fluoro-N-(6-((tetrahydro-2H-pyran-2-yl)oxy)hexyl)adamantane-1-carboxamide (1p)

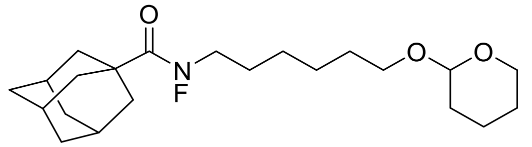

N-(6-((Tetrahydro-2H-pyran-2-yl)oxy)hexyl)adamantane-1-carboxamide **SI-16** (2.847 g, 7.8 mmol) was subjected to General Procedure B. Purification by silica flash column chromatography (0-30% acetone/hexanes) provided **1p** (966.4 mg, 32% yield) as a yellow oil.

^{1}H-NMR (500 MHz, $CDCl_3$): δ 4.58 – 4.54 (m, 1H), 3.90 – 3.79 (m, 2H), 3.77 – 3.69 (m, 2H), 3.54 – 3.46 (m, 1H), 3.41 – 3.34 (m, 1H), 2.05 – 1.96 (m, 10H), 1.89 – 1.47 (m, 15H), 1.44 – 1.31 (m, 4H).
^{13}C-NMR (125 MHz, $CDCl_3$): δ 180.8 (d, *J* = 3.6 Hz), 99.0, 67.6, 62.5, 51.1 (d, *J* = 11.2 Hz), 42.7 (d, *J* = 3.4 Hz), 37.6 (d, *J* = 5.4 Hz), 36.8, 30.9, 29.8, 28.3, 26.6, 26.4 (d, *J* = 2.7 Hz), 26.0, 25.7, 19.9.
^{19}F-NMR (376 MHz, $CDCl_3$): δ -70.4 (t, *J* = 33.2 Hz).
HRMS (APCI): Calcd. for $C_{22}H_{37}FNO_3$ [M+H], 382.2752. Found: 382.2752.
FTIR (Film) cm^{-1}: 2905, 2850, 1673, 1453, 1120, 1077, 1023.
TLC: R_f = 0.46, 20% acetone/hexanes.

6-(N-Fluoroadamantane-1-carboxamido)hexyl acetate (1q)

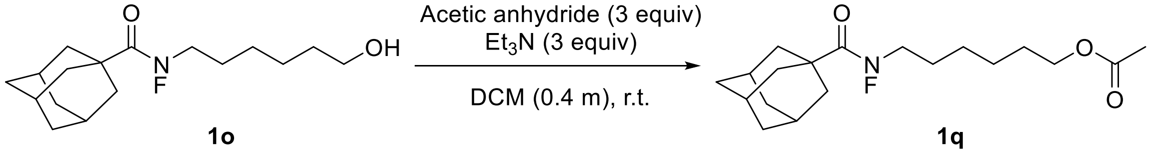

To a flame-dried round bottom flask equipped with a stir bar and backfilled with N_2 were added *N*-fluoro-*N*-(6-hydroxyhexyl)adamantane-1-carboxamide **1o** (137.5 mg, 0.5 mmol) and DCM (5 mL). Acetic anhydride (0.14 mL, 1.4 mmol) followed by triethylamine (0.2 mL, 1.4 mmol) were added dropwise at room temperature. The reaction was stirred at room temperature for 14 hours. The reaction was quenched with $NaHCO_3$ (5 mL), diluted with DCM (30 mL) and water (20 mL) and extracted with DCM (3 x 30 mL). The combined

layers were washed with 1 M aqueous HCl then brine, dried with $MgSO_4$, and concentrated by rotary evaporation to give a crude oil. Purification by silica flash column chromatography (0-30% EtOAc/hexanes) provided **1q** (137.9 mg, 87% yield**)** as a yellow oil.

^{1}H-NMR (500 MHz, $CDCl_3$): δ 4.03 (t, J = 6.7 Hz, 2H), 3.77 (dt, J = 33.1, 7.1 Hz, 2H), 2.06 – 1.93 (m, 12H), 1.75 – 1.57 (m, 10H), 1.40 – 1.31 (m, 4H).
^{13}C-NMR (125 MHz, $CDCl_3$): δ 180.8 (d, J = 3.6 Hz), 171.3, 64.5, 51.0 (d, J = 11.1 Hz), 42.6 (d, J = 3.3 Hz), 37.6 (d, J = 5.4 Hz), 36.7, 28.6, 28.2, 26.3, 26.2 (d, J = 2.8 Hz), 25.7, 21.1.
^{19}F-NMR (376 MHz, $CDCl_3$): δ -70.3 (t, J = 33.0 Hz).
HRMS (APCI): Calcd. for $C_{19}H_{31}FNO_3$ [M+H], 340.2282. Found: 340.2284.
FTIR (Film) cm^{-1}: 2906, 2853, 1738, 1675, 1454, 1364, 1236.
TLC: R_f = 0.48, 20% EtOAc/hexanes.

***N*-(6-Chlorohexyl)-*N*-fluoroadamantane-1-carboxamide (1r)**

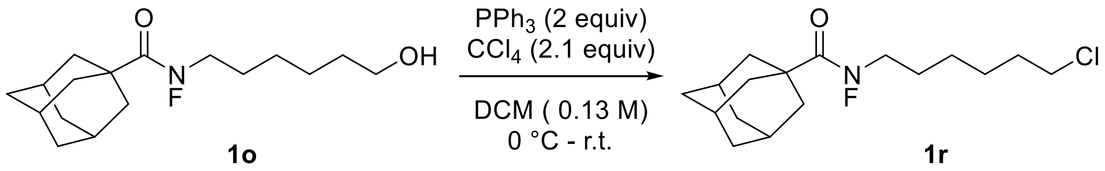

To a flame-dried round bottom flask equipped with a stir bar was added triphenylphosphine (347 mg, 1.3 mmol). The contents were evacuated and backfilled with N_2. DCM (4 mL) followed by CCl_4 (0.15 mL, 1.4 mmol) were added and the mixture placed in an ice bath and stirred at 0 °C for 10 minutes. *N*-Fluoro-*N*-(6-hydroxyhexyl)adamantane-1-carboxamide **1o** (197.5 mg, 0.66 mmol) in DCM (1 mL) was added dropwise at 0 °C. The reaction was removed from the ice bath, warmed to room temperature, and stirred for 14 h. The reaction was concentrated under reduced pressure and diluted with 25% EtOAc/hexanes (10 mL), filtered through a plug of silica, and the filtrate concentrated under reduced pressure. Purification by silica flash column chromatography (10-50% acetone/hexanes) provided **1r** (156.1 mg, 74% yield**)** as a pale yellow oil.

^{1}H-NMR (500 MHz, $CDCl_3$): δ 3.79 (dt, J = 33.0, 7.1 Hz, 2H), 3.52 (t, J = 6.7 Hz, 2H), 2.07 – 1.93 (m, 9H), 1.82 – 1.62 (m, 10H), 1.51 – 1.42 (m, 2H), 1.40 – 1.32 (m, 2H).
^{13}C-NMR (125 MHz, $CDCl_3$): δ 180.8 (d, J = 3.5 Hz), 51.0 (d, J = 11.1 Hz), 45.0, 42.7 (d, J = 3.5 Hz), 37.6 (d, J = 5.3 Hz), 36.7, 32.6, 28.2, 26.6, 26.3 (d, J = 2.8 Hz), 26.0.
^{19}F-NMR (376 MHz, $CDCl_3$): δ -70.3 (t, J = 33.2 Hz).
HRMS (APCI): Calcd. for $C_{17}H_{28}ClFNO$ [M+H], 316.1838. Found: 316.1840.
FTIR (Film) cm^{-1}: 2905, 2852, 1673, 1454.
TLC: R_f = 0.62, 30% acetone/hexanes.

N-Fluoro-*N*-(5-hydroxypentyl)adamantane-1-carboxamide (1s)

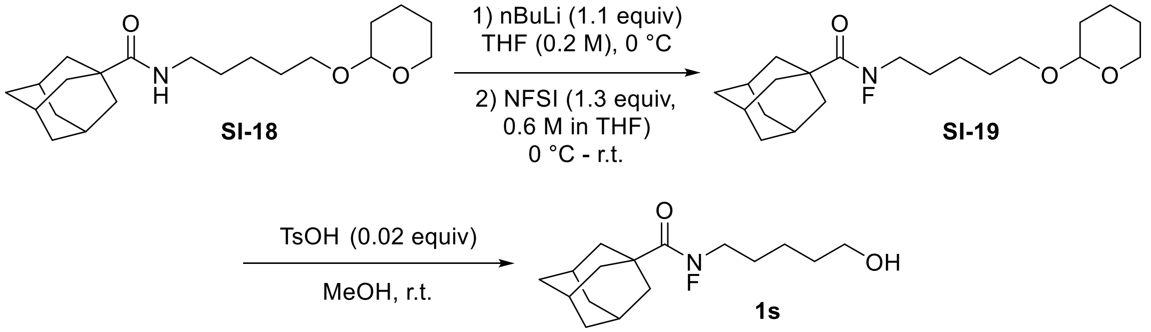

N-(5-((Tetrahydro-2H-pyran-2-yl)oxy)pentyl)adamantane-1-carboxamide **SI-18** (840 mg, 2.4 mmol) was subjected to General Procedure B. Purification by silica flash column chromatography (0-30% acetone/hexanes) provided the desired fluoramide **SI-19** with a THP protected alcohol. To remove the THP, **SI-19** was transferred to a flame-dried round bottom flask equipped with a stir bar and dissolved in MeOH (200 mL). *p*-Toluenesulfonic acid monohydrate (29.4 mg, 0.16 mmol) was added and the reaction stirred at room temperature for 14 hours. The reaction was quenched with saturated aqueous $NaHCO_3$ (10 mL), diluted with DCM (75 mL) and water (50 mL), and extracted with DCM (3 x 75 mL). The combined organic layers were washed with brine, dried with $MgSO_4$, and concentrated by rotary evaporation to yield a crude yellow oil. Purification by silica flash column chromatography (10-50% acetone/hexanes) provided **1s** (119.6 mg, 17% yield over 2 steps**)** as a yellow oil.

^{1}H-NMR (500 MHz, $CDCl_3$): δ 3.80 (dt, *J* = 33.0, 7.1 Hz, 2H), 3.63 (t, *J* = 6.5 Hz, 2H), 2.05 – 1.94 (m, 9H), 1.78 – 1.64 (m, 8H), 1.63 – 1.51 (m, 3H), 1.45 – 1.37 (m, 2H).
^{13}C-NMR (125 MHz, $CDCl_3$): δ 180.7 (d, *J* = 3.8 Hz), 62.7, 50.9 (d, *J* = 11.1 Hz), 42.7 (d, *J* = 3.3 Hz), 37.6 (d, *J* = 5.3 Hz), 36.7, 32.3, 28.2, 26.2 (d, *J* = 2.7 Hz), 22.9.
^{19}F-NMR (376 MHz, $CDCl_3$): δ -70.1 (t, *J* = 32.9 Hz).
HRMS (APCI): Calcd. for $C_{16}H_{27}FNO_2$ [M+H], 284.2020. Found: 284.2022.
FTIR (Film) cm^{-1}: 3368, 2904, 2850, 1670, 1453, 1345, 1055.
TLC: R_f = 0.36, 30% acetone/hexanes.

N-Fluoro-N-(6-oxohexyl)adamantane-1-carboxamide (SI-20)

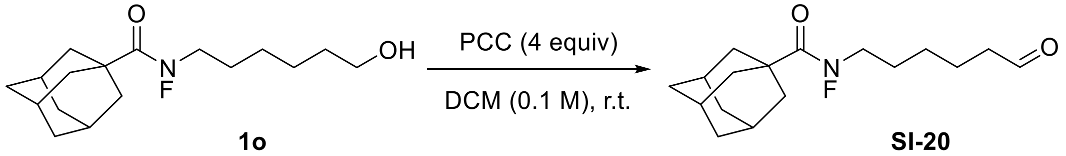

To a flame-dried round bottom flask equipped with a stir bar was added *N*-fluoro-*N*-(6-hydroxyhexyl)adamantane-1-carboxamide **1o** (255.5 mg, 0.9 mmol) as a solution in DCM (1 mL). DCM was added (9 mL), and the solution stirred. PCC (870 mg, 3.4 mmol) was added in one portion, and the reaction was stirred at room temperature for 5 hours. Silica was added until the mixture became a slurry and then filtered through a plug of silica, eluting with Et_2O (150 mL). The filtrate was concentrated under reduced pressure. No further purification was required and **SI-20** (228.9 mg, 90% yield) was obtained as a pale yellow oil.

^{1}H-NMR (500 MHz, $CDCl_3$): δ 9.77 – 9.75 (m, 1H), 3.79 (dt, *J* = 33.0, 7.0 Hz, 2H), 2.44 (td, *J* = 7.3, 1.7 Hz, 2H), 2.06 – 1.95 (m, 9H), 1.78 – 1.61 (m, 10H), 1.44 – 1.33 (m, 2H).
^{13}C-NMR (125 MHz, $CDCl_3$): δ 202.4, 180.8 (d, *J* = 3.6 Hz), 50.8 (d, *J* = 11.1 Hz), 43.8, 42.7 (d, *J* = 3.4 Hz), 37.6 (d, *J* = 5.3 Hz), 36.7, 28.2, 26.3, 26.2, 21.2.
^{19}F-NMR (376 MHz, $CDCl_3$): δ -70.1 (t, *J* = 33.0 Hz).
HRMS (APCI): Calcd. for $C_{17}H_{28}NO_2$ [M+H], 296.2020. Found: 296.2021.
FTIR (Film) cm^{-1}: 2905, 2851, 1724, 1672, 1454.
TLC: R_f = 0.48, 30% acetone/hexanes.

N-Fluoro-N-(hept-6-en-1-yl)adamantane-1-carboxamide (1t)

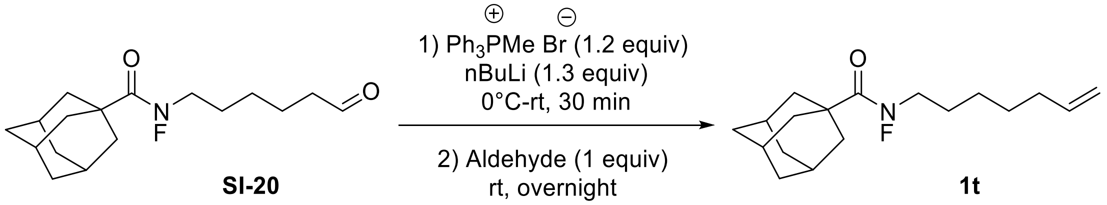

To a flame-dried round bottom flask equipped with a stir bar was added methyltriphenylphosphonium bromide (283.5 mg, 0.8 mmol). The contents were evacuated and backfilled with N_2. THF (5 mL) was added and the solution stirred at 0 °C for 10 minutes. *n*BuLi (0.4 mL, 0.85 mmol, 2.2 M in hexanes) was added dropwise at 0 °C. The reaction was removed from the ice bath and allowed to warm to room temperature over 30 minutes. *N*-Fluoro-*N*-(6-oxohexyl)adamantane-1-carboxamide **SI-20** (192.5 mg, 0.65 mmol) in THF (6.5 mL) was added dropwise at room temperature and the reaction was stirred for 16 hours. The reaction was quenched with saturated aqueous NH_4Cl (2 mL), diluted with DCM (30 mL) and water (20 mL) and extracted with DCM (3 x 30 mL). The

combined layers were washed with brine, dried with $MgSO_4$, and concentrated by rotary evaporation to give a crude orange oil. Purification by silica flash column chromatography (0-45% EtOAc/hexanes) provided **1t** (68 mg, 36% yield**)** as a pale yellow oil.

^{1}H-NMR (500 MHz, $CDCl_3$): δ 5.85 – 5.73 (m, 1H), 5.05 – 4.90 (m, 2H), 3.78 (dt, *J* = 33.1, 7.2 Hz, 2H), 2.11 – 1.94 (m, 11H), 1.80 – 1.61 (m, 8H), 1.48 – 1.22 (m, 4H).
^{13}C-NMR (125 MHz, $CDCl_3$): δ 180.8 (d, *J* = 3.4 Hz), 138.8, 114.7, 51.1 (d, *J* = 11.2 Hz), 42.7 (d, *J* = 3.3 Hz), 37.6 (d, *J* = 5.3 Hz), 36.8, 33.7, 28.6, 28.3, 26.3 (d, *J* = 2.7 Hz), 26.2.
^{19}F-NMR (376 MHz, $CDCl_3$): δ -70.4 (t, *J* = 33.1 Hz).
HRMS (APCI): Calcd. for $C_{18}H_{29}FNO$ [M+H], 294.2228. Found: 294.2229.
FTIR (Film) cm^{-1}: 2906, 2851, 1675, 1641, 1454, 1345, 909.
TLC: R_f = 0.77, 30% acetone/hexanes.

***N*-Fluoro-*N*-(4-fluorooctyl)adamantane-1-carboxamide (1u)**

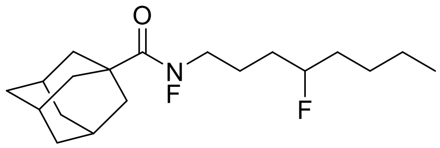

N-(4-Fluorooctyl)adamantane-1-carboxamide **2c** (571.8 mg, 1.8 mmol) was subjected to General Procedure B. Purification by silica flash column chromatography (0-25% EtOAc/hexanes) followed by a second column (0-25% Et_2O/hexanes) provided **1u** (152.1 mg, 25% yield) as a pale yellow oil.

^{1}H-NMR (500 MHz, $CDCl_3$): δ 4.56 – 4.40 (m, 1H), 3.92 – 3.74 (m, 2H), 2.05 – 1.97 (m, 9H), 1.91 – 1.81 (m, 1H), 1.79 – 1.27 (m, 15H), 0.91 (t, *J* = 7.1 Hz, 3H).
^{13}C-NMR (125 MHz, $CDCl_3$): δ 180.9 (d, *J* = 3.7 Hz), 94.0 (d, *J* = 167.7 Hz), 50.9 (d, *J* = 11.0 Hz), 42.7 (d, *J* = 3.4 Hz), 37.6 (d, *J* = 5.4 Hz), 36.8, 35.0 (d, *J* = 20.7 Hz), 32.3 (d, *J* = 21.4 Hz), 28.1, 27.4 (d, *J* = 4.4 Hz), 22.7, 22.4 (m), 14.1.
^{19}F-NMR (376 MHz, $CDCl_3$): δ -70.2 (t, *J* = 33.0 Hz), -180.7 (dtt, *J* = 47.7, 29.4, 17.5 Hz).
HRMS (ESI): Calcd. for $C_{19}H_{32}F_2NO$ [M+H], 328.2446. Found: 328.2445.
FTIR (Film) cm^{-1}: 2907, 2853, 1675, 1454.
TLC: R_f = 0.56, 20% Et_2O/hexanes.

Iron-catalyzed fluorine-transfer reaction General Procedure C

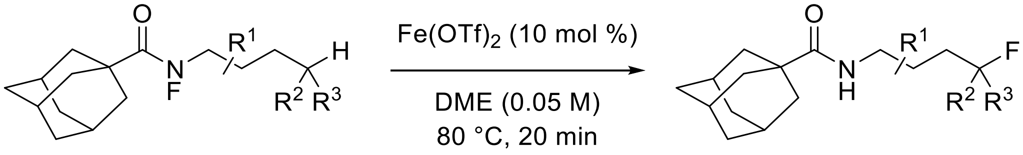

Catalytic reactions were typically run on 0.1-0.13 mmol scale for optimization, and 0.3 mmol scale for isolated yields.

To a flame-dried vial with a stir bar was added fluoroamide (1 equiv). The contents were evacuated and backfilled with N_2. The vial was capped and brought into a N_2 atmosphere glovebox. Anhydrous DME (0.05 M), followed by anhydrous $Fe(OTf)_2$ (10 mol %) were added. The vial was capped, sealed with PTFE tape, removed from the glovebox, and stirred in an oil bath at 80 °C for 20 minutes. The reaction was quenched with EtOAc (2 mL) and filtered through a 2-inch silica plug with ~120 mL EtOAc. The solution was concentrated by rotary evaporation and the crude mixture purified by silica flash column chromatography (typically with acetone/hexanes).

Note: $Fe(OTf)_2$ should be weighed in a glovebox for use. The reaction is air and moisture sensitive. Anhydrous DME was dispensed under argon atmosphere, degassed, and kept under inert atmosphere before use. Comparable yields were also obtained when adding anhydrous DME via syringe to the fluoroamide outside of the glovebox before $Fe(OTf)_2$ addition, or adding a solution of fluoroamide in anhydrous DME to a septum-capped vial containing $Fe(OTf)_2$ retrieved from the glovebox.

N-(4-Fluorooctyl)pivalamide (2a)

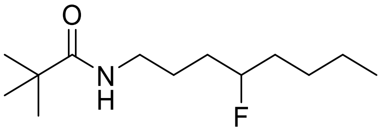

To a flame-dried vial with a stir bar was added *N*-fluoro-*N*-octylpivalamide **1a** (30.2 mg, 0.13 mmol). The contents were evacuated and backfilled with N_2. Anhydrous DME (2.6 mL) was added via syringe and the vial was capped, sealed with PTFE tape, and brought into a N_2 atmosphere glovebox. $Fe(OTf)_2$ (4.6 mg) was added, the vial capped, sealed with PTFE tape, and stirred in the glovebox at room temperature for 24 hours. The reaction was removed from the glovebox, quenched with EtOAc (0.5 mL), filtered through a 1-inch silica plug with ~20 mL EtOAc, and concentrated by rotary evaporation to give a crude yellow oil. ^{1}H-NMR analysis using 1,3,5-trimethoxybenzene as an internal standard gave **2a** in 64% yield.

<u>^{1}H-NMR</u> (500 MHz, $CDCl_3$): δ 5.71 (s, 1H), 4.57 – 4.36 (m, 1H), 3.31 – 3.20 (m, 2H), 1.72 – 1.12 (m, 19H), 0.89 (t, *J* = 6.7 Hz, 3H).
<u>^{13}C-NMR</u> (125 MHz, $CDCl_3$): δ 178.5, 94.3 (d, *J* = 167.0 Hz), 39.4, 38.8, 35.0 (d, *J* = 20.7 Hz), 32.5 (d, *J* = 21.2 Hz), 27.7, 27.4 (d, *J* = 3.4 Hz), 25.6 (d, *J* = 3.7 Hz), 22.7, 14.1.
<u>^{19}F-NMR</u> (376 MHz, $CDCl_3$): δ -180.1 (m).
<u>HRMS</u> (APCI): Calcd. for $C_{13}H_{27}FNO$ [M+H], 232.2071. Found: 232.2070.
<u>FTIR</u> (Film) cm^{-1}: 3345, 2933, 1636, 1532.
<u>TLC:</u> R_f = 37, 30% acetone/hexanes (visualized with $KMnO_4$).

_N_-(4-Fluorooctyl)-2,2-dimethylbutanamide (2b)
4-Fluoro-2,2-dimethyl-_N_-octylbutanamide (2bb)

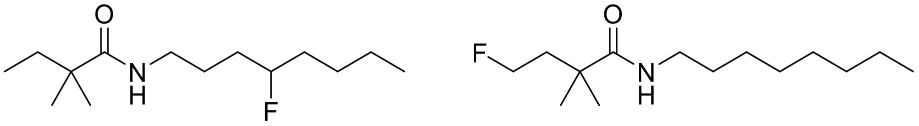

2b **2bb**

To a flame-dried vial with a stir bar was added *N*-fluoro-2,2-dimethyl-*N*-octylbutanamide **1b** (32.4 mg, 0.13 mmol). The contents were evacuated and backfilled with N_2. The vial was capped and brought into a N_2 atmosphere glovebox. Anhydrous DME (2.6 mL), followed by anhydrous $Fe(OTf)_2$ (4.6 mg) were added. The vial was capped, sealed with PTFE tape, and stirred in the glovebox at room temperature for 24 hours. The reaction was removed from the glovebox, quenched with EtOAc (0.5 mL), filtered through a 1-inch silica plug with ~20 mL EtOAc, and concentrated by rotary evaporation to give a crude yellow oil. ^{19}F-NMR analysis using 4-fluorotoluene as an internal standard gave **2b** (7% yield) and **2bb** (26% yield) as an inseparable mixture, and 6% of other fluorinated products.

<u>^{1}H-NMR</u> (500 MHz, $CDCl_3$): δ 5.77 – 5.61 (m, 2H), 4.58 – 4.39 (m, 3H), 3.32 – 3.17 (m, 4H), 2.00 – 1.89 (m, 2H), 1.73 – 1.09 (m, 36H), 0.94 – 0.79 (m, 9H).
<u>^{13}C-NMR</u> (125 MHz, $CDCl_3$): δ 177.8, 176.9, 94.4 (d, *J* = 166.9 Hz), 82.1 (d, *J* = 163.9 Hz), 42.5, 41.1 (d, *J* = 3.1 Hz), 40.9 (d, *J* = 18.7 Hz), 39.8, 39.4, 36.8, 35.0 (d, *J* = 20.7 Hz), 34.0, 32.6 (d, *J* = 21.1 Hz), 31.9, 29.7, 29.4 (d, *J* = 6.0 Hz), 27.4 (d, *J* = 4.7 Hz), 27.0, 26.0, 25.7 (d, *J* = 3.7 Hz), 25.1, 22.8, 22.7, 14.2, 14.1, 9.3.
<u>^{19}F-NMR</u> (376 MHz, $CDCl_3$): δ **2b** -180.1 (m). δ **2bb** -216.7 (tt, *J* = 47.5, 26.5 Hz).
<u>HRMS</u> (APCI): Calcd. for $C_{14}H_{29}FNO$ [M+H], 246.2228. Found: 246.2228.
<u>FTIR</u> (Film) cm^{-1}: 3345, 2927, 2856, 1636, 1535.
<u>TLC:</u> R_f = 0.46, 30% acetone/hexanes (visualized with $KMnO_4$).

N-(4-Fluorooctyl)adamantane-1-carboxamide (2c)

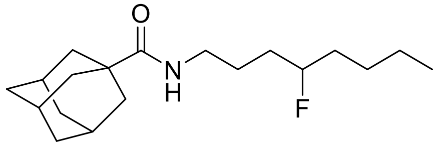

N-Fluoro-*N*-octyladamantane-1-carboxamide **1c** (92.9 mg, 0.3 mmol) was subjected to General Procedure C. Purification by silica flash column chromatography (10-50% Et_2O/hexanes) provided **2c** (66.4 mg, 71% yield) as a white solid.

^{1}H-NMR (500 MHz, $CDCl_3$): δ 5.62 (s, 1H), 4.58 – 4.39 (m, 1H), 3.33 – 3.21 (m, 2H), 2.09 – 2.00 (m, 3H), 1.91 – 1.81 (m, 6H), 1.79 – 1.18 (m, 16H), 0.91 (t, *J* = 7.1 Hz, 3H).
^{13}C-NMR (125 MHz, $CDCl_3$): δ 178.0, 94.3 (d, *J* = 167.1 Hz), 40.6, 39.4, 39.0, 36.6, 34.9 (d, *J* = 20.8 Hz), 32.5 (d, *J* = 21.1 Hz), 28.3, 27.3 (d, *J* = 4.7 Hz), 25.5 (d, *J* = 3.8 Hz), 22.6, 14.0.
^{19}F-NMR (376 MHz, $CDCl_3$): δ -180.1 (m).
HRMS (APCI): Calcd. for $C_{19}H_{33}FNO$ [M+H], 310.2541. Found: 310.2543.
FTIR (Film) cm^{-1}: 3342, 2904, 2850, 1631, 1526, 1451.
TLC: R_f = 0.36, 30% acetone/hexanes (visualized with $KMnO_4$).

N-(4-Fluorobutyl)adamantane-1-carboxamide (2d)

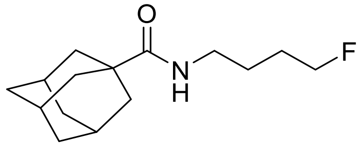

N-Butyl-*N*-fluoroadamantane-1-carboxamide **1d** (75.9 mg, 0.3 mmol) was subjected to General Procedure C. Purification by silica flash column chromatography (0-20% acetone/hexanes) provided **2d** (28.3 mg, 37% yield) as a white solid.

^{1}H-NMR (500 MHz, $CDCl_3$): δ 5.68 (s, 1H), 4.45 (dt, *J* = 47.2, 5.8 Hz, 2H), 3.31 – 3.24 (m, 2H), 2.06 – 1.99 (m, 3H), 1.85 – 1.80 (m, 6H), 1.76 – 1.57 (m, 10H).
^{13}C-NMR (125 MHz, $CDCl_3$): δ 178.1, 83.9 (d, *J* = 164.7 Hz), 40.7, 39.4, 38.8, 36.7, 28.3, 27.9 (d, *J* = 19.8 Hz), 25.8 (d, *J* = 4.6 Hz).
^{19}F-NMR (376 MHz, $CDCl_3$): δ -218.1 (tt, *J* = 47.3, 25.7 Hz).
HRMS (APCI): Calcd. for $C_{15}H_{25}FNO$ [M+H], 254.1915. Found: 254.1917.
FTIR (Film) cm^{-1}: 3344, 2903, 2850, 1633, 1528, 1451.
TLC: R_f = 0.40, 30% acetone/hexanes (visualized with $KMnO_4$).

N-(4-Fluoropentyl)adamantane-1-carboxamide (2e)

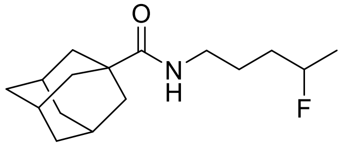

N-Fluoro-*N*-pentyladamantane-1-carboxamide **1e** (80.98 mg, 0.3 mmol) was subjected to General Procedure C. Purification by silica flash column chromatography (0-20% acetone/hexanes) provided **2e** (56.3 mg, 70% yield) as a white solid.

^{1}H-NMR (500 MHz, $CDCl_3$): δ 5.66 (s, 1H), 4.76 – 4.56 (m, 1H), 3.32 – 3.20 (m, 2H), 2.06 – 1.99 (m, 3H), 1.87 – 1.80 (m, 6H), 1.76 – 1.48 (m, 10H), 1.31 (dd, *J* = 23.9, 6.2 Hz, 3H).
^{13}C-NMR (125 MHz, $CDCl_3$): δ 178.1, 90.8 (d, *J* = 164.5 Hz), 40.7, 39.4, 39.0, 36.7, 34.3 (d, *J* = 21.0 Hz), 28.3, 25.5 (d, *J* = 4.1 Hz), 21.1 (d, *J* = 22.9 Hz).
^{19}F-NMR (376 MHz, $CDCl_3$): δ -172.5 (m).
HRMS (APCI): Calcd. for $C_{16}H_{27}FNO$ [M+H], 268.2071. Found: 268.2073.
FTIR (Film) cm^{-1}: 3342, 2904, 2850, 1633, 1529, 1451.
TLC: R_f = 0.44, 30% acetone/hexane (visualized with $KMnO_4$).

N-(4-Fluorohexyl)adamantane-1-carboxamide (2f)

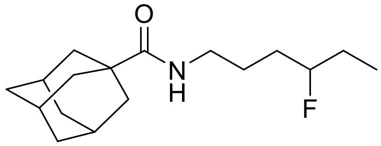

N-Fluoro-*N*-hexyladamantane-1-carboxamide **1f** (84.4 mg, 0.3 mmol) was subjected to General Procedure C. Purification by silica flash column chromatography (0-20% acetone/hexanes) provided **2f** (58.3 mg, 69% yield) as a white solid.

^{1}H-NMR (500 MHz, $CDCl_3$): δ 5.63 (s, 1H), 4.51 – 4.33 (m, 1H), 3.34 – 3.22 (m, 2H), 2.08 – 2.00 (m, 3H), 1.87 – 1.82 (m, 6H), 1.78 – 1.49 (m, 12H), 0.96 (t, *J* = 7.5 Hz. 3H).
^{13}C-NMR (125 MHz, $CDCl_3$): δ 178.1, 95.5 (d, *J* = 167.4 Hz), 40.7, 39.4, 39.1, 36.7, 32.0 (d, *J* = 21.3 Hz), 28.3, 28.2 (d, *J* = 20.9 Hz), 25.6 (d, *J* = 3.8 Hz), 9.5 (d, *J* = 5.8 Hz).
^{19}F-NMR (376 MHz, $CDCl_3$): δ -181.2 (m).
HRMS (APCI): Calcd. for $C_{17}H_{29}FNO$ [M+H], 282.2228. Found: 282.2229.
FTIR (Film) cm^{-1}: 3335, 2906, 2850, 1633, 1528, 1451.
TLC: R_f = 0.32, 30% acetone/hexanes (visualized with $KMnO_4$).

N-(4-Fluorodecyl)adamantane-1-carboxamide (2g)

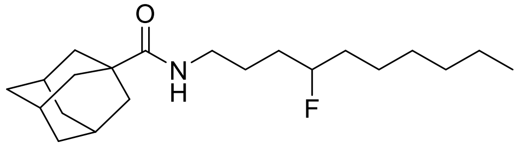

N-Decyl-*N*-fluoroadamantane-1-carboxamide **1g** (101.5 mg, 0.3 mmol) was subjected to General Procedure C. Purification by silica flash column chromatography (10-45% Et_2O/hexanes) provided **2g** (77.01 mg, 76% yield) as a white solid.

<u>^{1}H-NMR</u> (500 MHz, $CDCl_3$): δ 5.62 (s, 1H), 4.58 – 4.38 (m, 1H), 3.34 – 3.20 (m, 2H), 2.07 – 2.00 (m, 3H), 1.87 – 1.81 (m, 6H), 1.78 – 1.21 (m, 20H), 0.88 (t, *J* = 7.1 Hz, 3H).
<u>^{13}C-NMR</u> (125 MHz, $CDCl_3$): δ 178.0, 94.3 (d, *J* = 167.2 Hz), 40.7, 39.4, 39.0, 36.6, 35.2 (d, *J* = 20.8 Hz), 32.5 (d, *J* = 21.3 Hz), 31.8, 29.2, 28.2, 25.5 (d, *J* = 3.8 Hz), 25.1 (d, *J* = 4.7 Hz), 22.6, 14.1.
<u>^{19}F-NMR</u> (376 MHz, $CDCl_3$): δ -180.1 (m).
<u>HRMS</u> (APCI): Calcd. for $C_{21}H_{37}FNO$ [M+H], 338.2854. Found: 338.2857.
<u>FTIR</u> (Film) cm^{-1}: 3341, 2905, 2851, 1632, 1528, 1452.
<u>TLC:</u> R_f = 0.40, 30% acetone/hexanes (visualized with $KMnO_4$).

N-(4-Fluoro-3-methylbutyl)adamantane-1-carboxamide (2h)

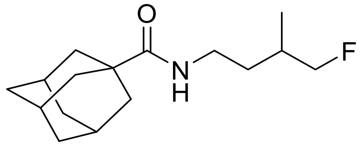

N-Fluoro-*N*-isopentyladamantane-1-carboxamide **1h** (81.4 mg, 0.3 mmol) was subjected to General Procedure C. Purification by silica flash column chromatography (0-20% acetone/hexanes) provided **2h** (46.5 mg, 57% yield) as a white solid.

<u>^{1}H-NMR</u> (500 MHz, $CDCl_3$): δ 5.62 (s, 1H), 4.39 – 4.14 (m, 2H), 3.42 – 3.22 (m, 2H), 2.08 – 1.98 (m, 3H), 1.95 – 1.79 (m, 7H), 1.77 – 1.57 (m, 7H), 1.47 – 1.35 (m, 1H), 0.97 (dd, *J* = 6.8, 1.2 Hz, 3H).
<u>^{13}C-NMR</u> (125 MHz, $CDCl_3$): δ 178.1, 88.2 (d, *J* = 168.9 Hz), 40.7, 39.4, 37.2, 36.7, 32.8 (d, *J* = 4.4 Hz), 32.2 (d, *J* = 18.3 Hz), 28.3, 15.9 (d, *J* = 7.4 Hz).
<u>^{19}F-NMR</u> (376 MHz, $CDCl_3$): δ -221.5 (td, *J* = 47.6, 19.8 Hz).
<u>HRMS</u> (APCI): Calcd. for $C_{16}H_{27}FNO$ [M+H], 268.2071. Found: 268.2073.
<u>FTIR</u> (Film) cm^{-1}: 3339, 2905, 2850, 1632, 1530.
<u>TLC:</u> R_f = 0.38, 30% acetone/hexanes (visualized with $KMnO_4$).

N-(5-Fluoroheptan-2-yl)adamantane-1-carboxamide (2i)

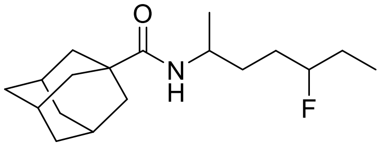

N-Fluoro-*N*-(heptan-2-yl)adamantane-1-carboxamide **1i** (88.9 mg, 0.3 mmol) was subjected to General Procedure C (modification: ran at 80 °C for 35 minutes). Purification by silica flash column chromatography (0-20% acetone/hexanes) provided **2i** (46.1 mg, 52% yield, d.r. = 1:2.3) as a white solid.

^{1}H-NMR (400 MHz, $CDCl_3$): δ 5.42 – 5.25 (m, 1H), 4.49 – 4.29 (m, 1H), 4.08 – 3.92 (m, 1H), 2.07 – 1.99 (m, 3H), 1.86 – 1.79 (m, 6 H), 1.78 – 1.41 (m, 12H), 1.14 – 1.09 (m, 3H), 0.98 – 0.92 (m, 3H).
^{13}C-NMR (125 MHz, $CDCl_3$): δ (major) 177.4, 95.7 (d, *J* = 168.2 Hz), 44.8, 40.6, 39.5, 36.7, 32.7 (d, *J* = 3.9 Hz), 31.5 (d, *J* = 21.3 Hz), 28.3, 28.2 (d, *J* = 21.5 Hz), 21.2, 9.5 (d, *J* = 5.9 Hz). δ (minor) 177.4, 95.4 (d, *J* = 167.4 Hz), 44.3, 40.7, 39.5, 36.7, 32.8 (d, *J* = 3.9 Hz), 31.3 (d, *J* = 21.2 Hz), 28.3, 28.3 (d, *J* = 21.4 Hz), 21.5, 9.5 (d, *J* = 6.0 Hz).
^{19}F-NMR (376 MHz, $CDCl_3$): δ (major) -181.4 (m). δ (minor) -181.9 (m).
HRMS (APCI): Calcd. for $C_{18}H_{31}FNO$ [M+H], 296.2384. Found: 296.2386.
FTIR (Film) cm^{-1}: 3307, 2901, 1629, 1542.
TLC: R_f = 0.40, 30% acetone/hexanes (visualized with $KMnO_4$).

N-(5-Fluoro-6-methylheptan-2-yl)adamantane-1-carboxamide (2j)

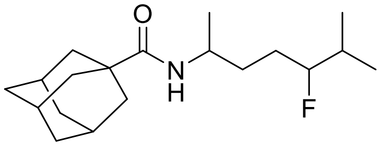

N-Fluoro-*N*-(6-methylheptan-2-yl)adamantane-1-carboxamide **1j** (92.7 mg, 0.3 mmol) was subjected to General Procedure C (modification: ran at 80 °C for 35 minutes). Purification by silica flash column chromatography (0-20% acetone/hexanes) provided **2j** (39.8 mg, 43% yield, d.r. = 1:2.7) as a white solid.

^{1}H-NMR (500 MHz, $CDCl_3$): δ 5.43 – 5.24 (m, 1H), 4.28 – 4.08 (m, 1H), 4.07 – 3.92 (m, 1H), 2.08 – 1.99 (m, 3H), 1.87 – 1.41 (m, 17H), 1.14 – 1.10 (m, 3H), 0.96-0.87 (m, 6H).
^{13}C-NMR (125 MHz, $CDCl_3$): δ (major) 177.3, 98.8 (d, *J* = 170.8 Hz), 44.9, 40.6, 39.5, 36.7, 33.0, 32.4 (d, *J* = 20.2 Hz), 29.1 (d, *J* = 21.7 Hz), 28.3, 21.2, 18.5 (d, *J* = 6.5 Hz), 17.4 (d, *J* = 6.2 Hz). δ (minor) 177.4, 98.4 (d, *J* = 170.5 Hz), 44.3, 40.7, 39.5, 36.7, 33.1, 32.5 (d, *J* = 20. Hz), 28.7 (d, *J* = 21.8 Hz), 28.3, 21.6, 18.4 (d, *J* = 5.6 Hz), 17.3 (d, *J* = 6.5 Hz).

^{19}F-NMR (376 MHz, $CDCl_3$): δ (major) -186.6 (m). δ (minor) -187.3 (m).
HRMS (APCI): Calcd. for $C_{19}H_{33}FNO$ [M+H], 310.2541. Found: 310.2542.
FTIR (Film) cm^{-1}: 3304, 2900, 1628, 1547.
TLC: R_f = 0.46, 30% acetone/hexanes (visualized with $KMnO_4$).

N-(2-(2-Fluorocyclohexyl)ethyl)adamantane-1-carboxamide (2k)

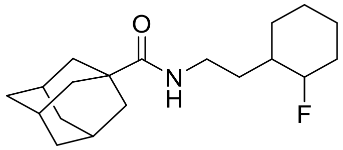

N-(2-Cyclohexylethyl)-*N*-fluoroadamantane-1-carboxamide **1k** (92.7 mg, 0.3 mmol) was subjected to General Procedure C. Purification by silica flash column chromatography (0-20% acetone/hexanes) provided **2k** (67.9 mg, 73% yield, d.r. = 1:1.9) as a white solid.

^{1}H-NMR (500 MHz, $CDCl_3$): δ 5.73 – 5.57 (m, 1H), 4.75 – 4.60 (m, 1H minor product), 4.11 (dtd, *J* = 49.2, 10.0, 4.5 Hz, 1H major product), 3.39 – 3.16 (m, 2H), 2.11 – 1.92 (m, 4H), 1.89 – 1.08 (m, 21H), 1.02 – 0.91 (m, 1H).
^{13}C-NMR (125 MHz, $CDCl_3$): δ (major) 177.9, 96.5 (d, *J* = 173.4 Hz), 41.4 (d, *J* = 16.5 Hz), 40.6, 39.4, 37.3, 36.7, 32.7 (d, *J* = 2.1 Hz), 32.5 (d, *J* = 18.6 Hz), 30.4 (d, *J* = 8.6 Hz), 28.3, 25.0, 24.1. δ (minor) 178.0, 91.5 (d, *J* = 171.0 Hz), 40.6, 39.4, 38.7 (d, *J* = 19.9 Hz), 37.2, 36.7, 32.1, 31.0 (d, *J* = 21.3 Hz), 28.3, 27.0 (d, *J* = 2.2 Hz), 25.0, 20.4 (d, *J* = 2.1 Hz).
^{19}F-NMR (376 MHz, $CDCl_3$): δ -172.1 (m).
HRMS (APCI): Calcd. for $C_{19}H_{31}FNO$ [M+H], 308.2384. Found: 308.2386.
FTIR (Film) cm^{-1}: 3342, 2904, 2850, 1631, 1528, 1450.
TLC: R_f = 0.41, 30% acetone/hexanes (visualized with $KMnO_4$).

N-(4-Fluorocycloheptyl)adamantane-1-carboxamide (2l)

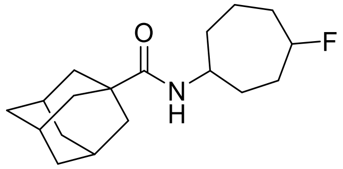

N-Cycloheptyl-*N*-fluoroadamantane-1-carboxamide **1l** (87.8 mg, 0.3 mmol) was subjected to General Procedure C. Purification by silica flash column chromatography (0-20% acetone/hexanes) provided **2l** (35.8 mg, 41% yield) as a white solid.

^{1}H-NMR (500 MHz, $CDCl_3$): δ 5.48 – 5.39 (m, 1H), 4.80 – 4.62 (m, 1H), 3.98 – 3.89 (m, 1H), 2.10 – 1.62 (m, 22H), 1.56 – 1.47 (m, 1H), 1.32 – 1.22 (m, 2H).

13C-NMR (125 MHz, $CDCl_3$): δ 177.0, 93.6 (d, *J* = 167.0 Hz), 49.8, 40.6, 39.4, 36.7, 35.3, 34.6 (d, *J* = 22.1 Hz), 30.4 (d, *J* = 22.5 Hz), 28.8 (d, *J* = 11.1 Hz), 28.3, 18.6 (d, *J* = 6.9 Hz).
19F-NMR (376 MHz, $CDCl_3$): δ -163.7 (m).
HRMS (APCI): Calcd. for $C_{18}H_{29}FNO$ [M+H], 294.2228. Found: 294.2230.
FTIR (Film) cm^{-1}: 3307, 2902, 2850, 1626, 1543.
TLC: R_f = 0.48, 30% acetone/hexanes (visualized with $KMnO_4$).

N-(4-Fluoro-4-methylpentyl)adamantane-1-carboxamide (2m)

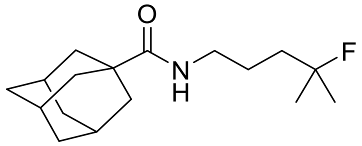

N-Fluoro-*N*-(4-methylpentyl)adamantane-1-carboxamide **1m** (84.6 mg, 0.3 mmol) was subjected to General Procedure C. Purification by silica flash column chromatography (0-20% acetone/hexanes) provided **2m** (10.9 mg, 13% yield) as a white solid.

1H-NMR (500 MHz, $CDCl_3$): δ 5.66 (s, 1H), 3.32 – 3.18 (m, 2H), 2.08 – 2.00 (m, 3H), 1.88 – 1.81 (m, 6H), 1.77 – 1.54 (m, 10H), 1.34 (d, *J* = 21.45 Hz, 6H).
13C-NMR (125 MHz, $CDCl_3$): δ 178.1, 95.6 (d, *J* = 165.1 Hz), 40.7, 39.5, 39.4, 38.6 (d, *J* = 23.1 Hz), 36.7, 28.3, 26.8 (d, *J* = 24.8 Hz), 24.4 (d, *J* = 4.6 Hz).
19F-NMR (376 MHz, $CDCl_3$): δ -138.0 (m).
HRMS (APCI): Calcd. for $C_{17}H_{29}FNO$ [M+H], 282.2228. Found: 282.2227.
FTIR (Film) cm^{-1}: 3356, 2905, 2850, 1632, 1532, 1432.
TLC: R_f = 0.40, 30% acetone/hexanes (visualized with $KMnO_4$).

N-(4-Fluoro-4-phenylbutyl)adamantane-1-carboxamide (2n)

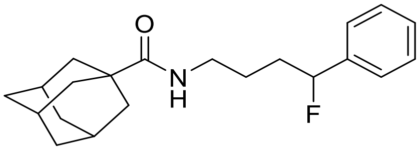

N-Fluoro-*N*-(4-phenylbutyl)adamantane-1-carboxamide **1n** (98.8 mg, 0.3 mmol) was subjected to General Procedure C. Purification by silica flash column chromatography (10-50% Et_2O/hexanes) followed by a second column (0-20% acetone/hexanes) provided **2n** (46.6 mg, 47% yield) as a pale yellow solid.

1H-NMR (400 MHz, CD_2Cl_2): δ 7.42 – 7.27 (m, 5H), 5.61 (s, 1H), 5.46 (ddd, *J* = 47.9, 8.2, 4.7 Hz, 1H), 3.31 – 3.16 (m, 2H), 2.06 – 1.49 (m, 19H).

^{13}C-NMR (125 MHz, $CDCl_3$): δ 178.1, 140.2 (d, *J* = 20.0 Hz), 128.6, 128.4, 125.6 (d, *J* = 6.9 Hz), 94.3 (d, *J* = 170.08 Hz), 40.7, 39.4, 38.9, 36.7, 34.6 (d, *J* = 23.9 Hz), 28.3, 25.5 (d, *J* = 3.7 Hz).
^{19}F-NMR (376 MHz, $CDCl_3$): δ -175.2 (ddd, *J* = 47.7, 29.4, 17.5 Hz).
HRMS (ESI): Calcd. for $C_{21}H_{28}FNONa$ [M+Na], 352.2047. Found: 352.2046.
FTIR (Film) cm^{-1}: 3345, 2905, 2850, 1635, 1527, 1452, 699.
TLC: R_f 0.38, 30% acetone/hexanes.

N-(4-Fluoro-6-hydroxyhexyl)adamantane-1-carboxamide (2o)

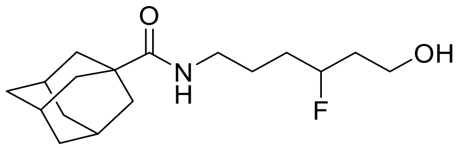

N-Fluoro-*N*-(6-hydroxyhexyl)adamantane-1-carboxamide **1o** (90.5 mg, 0.3 mmol) was subjected to General Procedure C. Purification by silica flash column chromatography (10-60% acetone/hexanes) provided **2o** (35.7 mg, 40% yield) as a white solid.

^{1}H-NMR (500 MHz, $CDCl_3$): δ 5.75 – 5.68 (m, 1H), 4.84 – 4.66 (m, 1H), 3.82 – 3.71 (m, 2H), 3.38 – 3.17 (m, 2H), 2.32 (s, 1H), 2.08 – 1.97 (m, 3H), 1.96 – 1.51 (m, 18H).
^{13}C-NMR (125 MHz, $CDCl_3$): δ 178.4, 92.0 (d, *J* = 165.9 Hz), 59.1 (d, *J* = 5.5 Hz), 40.7, 39.4, 38.7, 38.1 (d, *J* = 20.2 Hz), 36.6, 32.3 (d, *J* = 21.0 Hz), 28.2, 25.6 (d, *J* = 3.5 Hz).
^{19}F-NMR (376 MHz, $CDCl_3$): δ -182.4 (m).
HRMS (APCI): Calcd. for $C_{17}H_{29}FNO_2$ [M+H], 298.2177. Found: 298.2180.
FTIR (Film) cm^{-1}: 3348, 2905, 1635, 1534.
TLC: R_f = 0.26, 40% acetone/hexanes (visualized with $KMnO_4$).

N-(4-Fluoro-6-((tetrahydro-2H-pyran-2-yl)oxy)hexyl)adamantane-1-carboxamide (2p)

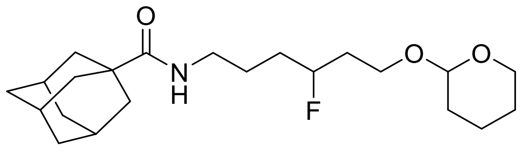

N-Fluoro-*N*-(6-((tetrahydro-2H-pyran-2-yl)oxy)hexyl)adamantane-1-carboxamide **1p** (114.8 mg, 0.3 mmol) was subjected to General Procedure C. Purification by silica flash column chromatography (0-20% acetone/hexanes) provided **2p** (50.0 mg, 44% yield, mixture of diastereomers) as a pale yellow oil.

^{1}H-NMR (500 MHz, $CDCl_3$): δ 5.78 – 5.61 (m, 1H), 4.84 – 4.51 (m, 2H), 3.90 – 3.67 (m, 2H), 3.56 – 3.40 (m, 2H), 3.30 – 3.16 (m, 2H), 2.05 – 1.97 (m, 3H), 1.91 – 1.44 (m, 24H).

^{13}C-NMR (125 MHz, $CDCl_3$): δ 178.0, 99.5, 98.8, 91.6 (d, *J* = 167.7 Hz), 91.5 (d, *J* = 167.1 Hz), 63.6 (d, *J* = 5.6 Hz), 63.3 (d, *J* = 5.5 Hz), 62.6, 62.3, 40.9, 39.6, 39.2, 36.8, 35.7 (d, *J* = 21.1 Hz), 35.6 (d, *J* = 21.3 Hz), 32.8 (d, *J* = 21.1 Hz), 32.8 (d, *J* = 20.8 Hz), 30.9, 28.4, 25.7 – 25.5 (m), 19.9, 19.7.
^{19}F-NMR (376 MHz, $CDCl_3$): δ -183.0 (m).
HRMS (APCI): Calcd. for $C_{22}H_{37}FNO_3$ [M+H], 382.2752. Found: 382.2753.
FTIR (Film) cm^{-1}: 3359, 2905, 2850, 1635, 1529, 1033.
TLC: R_f = 0.36, 30% acetone/hexanes (visualized with $KMnO_4$).

6-(Adamantane-1-carboxamido)-3-fluorohexyl acetate (2q)

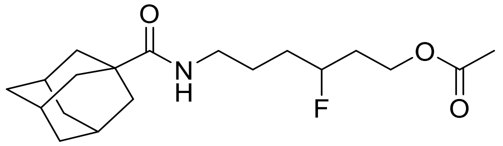

6-(*N*-Fluoroadamantane-1-carboxamido)hexyl acetate **1o** (102.2 mg, 0.3 mmol) was subjected to General Procedure C. Purification by silica flash column chromatography (0-25% acetone/hexanes) provided **2q** (65.1 mg, 64% yield) as a white solid.

^{1}H-NMR (500 MHz, $CDCl_3$): δ 5.64 (s, 1H), 4.71 – 4.53 (m, 1H), 4.25 – 4.13 (m, 2H), 3.32 – 3.23 (m, 2H), 2.07 – 2.01 (m, 6H), 2.00 – 1.80 (m, 8H), 1.78 – 1.52 (m, 10H).
^{13}C-NMR (125 MHz, $CDCl_3$): δ 178.0, 171.0, 90.8 (d, *J* = 168.5 Hz), 60.6 (d, *J* = 5.0 Hz), 40.6, 39.3, 38.9, 36.6, 34.3 (d, *J* = 21.0 Hz), 32.5 (d, *J* = 20.9 Hz), 28.2, 25.4 (d, *J* = 3.8 Hz), 21.0.
^{19}F-NMR (376 MHz, $CDCl_3$): δ -183.5 (m).
HRMS (APCI): Calcd. for $C_{19}H_{31}FNO_3$ [M+H], 340.2282. Found: 340.2285.
FTIR (Film) cm^{-1}: 3340, 2905, 2850, 1739, 1636, 1530, 1247.
TLC: R_f = 0.30, 30% acetone/hexanes (visualized with $KMnO_4$).

***N*-(6-Chloro-4-fluorohexyl)adamantane-1-carboxamide (2r)**

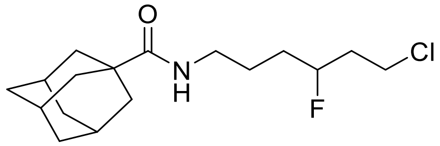

N-(6-chlorohexyl)-*N*-fluoroadamantane-1-carboxamide **1o** (94.3 mg, 0.3 mmol) was subjected to General Procedure C. Purification by silica flash column chromatography (0-20% acetone/hexanes) provided **2r** (51.1 mg, 54% yield) as a white solid.

^{1}H-NMR (500 MHz, $CDCl_3$): δ 5.71 (s, 1H), 4.77 – 4.60 (m, 1H), 3.66 – 3.58 (m, 2H), 3.30 – 3.21 (m, 2H), 2.15 – 1.77 (m, 11H), 1.76 – 1.50 (m, 10H).

^{13}C-NMR (125 MHz, $CDCl_3$): δ 178.1, 90.8 (d, *J* = 168.7 Hz), 40.7, 40.6 (d, *J* = 4.7 Hz), 39.4, 38.9, 38.2 (d, *J* = 21.2 Hz), 36.6, 32.3 (d, *J* = 20.7 Hz), 28.2, 25.5 (d, *J* = 3.8 Hz).
^{19}F-NMR (376 MHz, $CDCl_3$): δ -184.8 (m).
HRMS (APCI): Calcd. for $C_{17}H_{28}FNOCl$ [M+H], 316.1838. Found: 316.1841
FTIR (Film) cm^{-1}: 3344, 2903, 2849, 1630, 1524, 1450, 1283, 661.
TLC: R_f = 0.39, 30% acetone/hexanes (visualized with $KMnO_4$).

N-(4-Fluoro-5-hydroxypentyl)adamantane-1-carboxamide (2s)

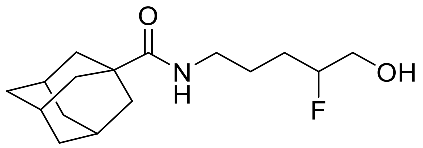

N-Fluoro-*N*-(5-hydroxypentyl)adamantane-1-carboxamide **1s** (71.1 mg, 0.25 mmol) was subjected to General Procedure C. Purification by silica flash column chromatography (10-60% acetone/hexanes) provided **2s** (26.8 mg, 38% yield) as a white solid.

^{1}H-NMR (500 MHz, $CDCl_3$): δ 5.72 (s, 1H), 4.67 – 4.49 (m, 1H), 3.78 – 3.60 (m, 2H), 3.37 – 3.20 (m, 2H), 2.50 (s, 1H), 2.07 – 1.99 (m, 3H), 1.85 – 1.79 (m, 6H), 1.77 – 1.49 (m, 10H).
^{13}C-NMR (125 MHz, $CDCl_3$): δ 178.4, 94.3 (d, *J* = 168.8 Hz), 64.8 (d, *J* = 22.3 Hz), 40.7, 39.4, 38.9, 36.6, 28.3 (d, *J* = 20.6 Hz), 28.2, 25.4 (d, *J* = 4.1 Hz).
^{19}F-NMR (376 MHz, $CDCl_3$): δ -189.4 (m).
HRMS (APCI): Calcd. for $C_{16}H_{27}FNO_2$ [M+H], 284.2020. Found: 284.2022.
FTIR (Film) cm^{-1}: 3343, 2905, 2850, 1629, 1533, 1451.
TLC: R_f = 0.23, 40% acetone/hexane (visualized with $KMnO_4$).

N-(4-Fluorohept-6-en-1-yl)adamantane-1-carboxamide (2t)

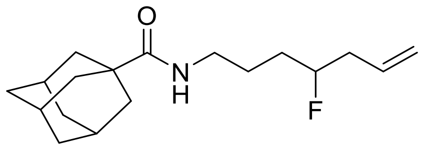

N-Fluoro-*N*-(hept-6-en-1-yl)adamantane-1-carboxamide **1t** (52.4 mg, 0.18 mmol) was subjected to General Procedure C. Purification by silica flash column chromatography (0-20% acetone/hexanes) provided **2t** (35.3 mg, 67% yield) as a white solid.

^{1}H-NMR (500 MHz, $CDCl_3$): δ 5.85 – 5.75 (m, 1H), 5.64 (s, 1H), 5.16 – 5.07 (m, 2H), 4.64 – 4.45 (m, 1H), 3.32 – 3.21 (m, 2H), 2.47 – 2.27 (m, 2H), 2.07 – 2.00 (m, 3H), 1.88 – 1.81 (m, 6H), 1.77 – 1.56 (m, 10H).

^{13}C-NMR (125 MHz, $CDCl_3$): δ 178.1, 133.1 (d, J = 6.3 Hz), 118.1, 93.2 (d, J = 169.7 Hz), 40.7, 39.6 (d, J = 21.6 Hz), 39.4, 39.0, 36.6, 31.9 (d, J = 20.9 Hz), 28.3, 25.5 (d, J = 3.7 Hz).
^{19}F-NMR (376 MHz, $CDCl_3$): δ -179.9 (m).
HRMS (APCI): Calcd. for $C_{18}H_{29}FNO$ [M+H], 294.2228. Found: 294.2230.
FTIR (Film) cm^{-1}: 3346, 2905, 2850, 1635, 1528.
TLC: R_f = 0.36, 30% acetone/hexanes (visualized with $KMnO_4$).

_N_-(4,4-Difluorooctyl)adamantane-1-carboxamide (2u)

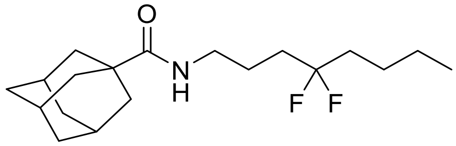

To a flame-dried vial with a stir bar was added *N*-(4-fluorooctyl)adamantane-1-carboxamide **1u** (34.2 mg, 0.1 mmol). The contents were evacuated and backfilled with N_2. The vial was capped and brought into a N_2 atmosphere glovebox. Anhydrous DME (2.0 mL), followed by anhydrous $Fe(OTf)_2$ (3.6 mg) were added. The vial was capped, sealed with PTFE tape, removed from the glovebox, and stirred in an oil bath at 80 °C for 20 minutes. The reaction was quenched with EtOAc (0.5 mL), filtered through a 1-inch silica plug with ~20 mL EtOAc, and concentrated by rotary evaporation to give a crude yellow oil. ^{19}F-NMR analysis using 4-fluorotoluene as an internal standard gave **2u** in 20% yield.

^{1}H-NMR (500 MHz, $CDCl_3$): δ 5.64 (s, 1H), 3.33 – 3.23 (m, 2H), 2.07 – 2.01 (m, 3H), 1.89 – 1.51 (m, 18H), 1.47 – 1.29 (m, 4H), 0.92 (t, J = 7.2 Hz, 3H).
^{13}C-NMR (125 MHz, $CDCl_3$): δ 178.2, 125.3 (t, J = 240.2 Hz), 40.8, 39.5, 38.9, 36.7, 36.4 (t, J = 25.3 Hz), 33.7 (t, J = 25.9 Hz), 28.3, 24.6 (t, J = 4.6 Hz), 22.9 (t, J = 4.1 Hz), 22.6, 14.0.
^{19}F-NMR (376 MHz, $CDCl_3$): δ -97.7 – -97.9 (m, 2F).
HRMS (APCI): Calcd. for $C_{19}H_{32}F_2NO$ [M+H], 328.2446. Found: 328.2445.
FTIR (Film) cm^{-1}: 3353, 2906, 2851, 1633, 1530, 1453.
TLC: R_f = 0.37, 30% acetone/hexanes (visualized with $KMnO_4$).

4-Fluorooctan-1-amine (SI-21)

Amine deprotection via triflic anhydride activation of amide.[10]

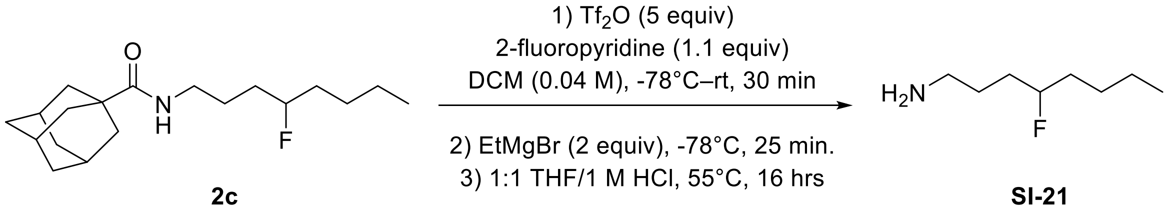

To a flame-dried round bottom flask equipped with a stir bar was added *N*-(4-fluorooctyl)adamantane-1-carboxamide **2c** (65.8 mg, 0.21 mmol). The contents were evacuated and backfilled with N_2. Anhydrous DCM (5.3 mL, 0.04 M) was added and the solution cooled to -78°C using an acetone/dry ice bath. Trifluoromethanesulfonic anhydride (0.17 mL, 1.1 mmol) was added dropwise at -78°C and stirred for 10 minutes. The solution was warmed to room temperature using a water bath and stirred for 20 minutes at room temperature. The reaction was cooled to -78°C using an acetone/dry ice bath and a solution of EtMgBr in Et_2O (0.19 mL, 0.4 mmol) was added. The reaction was left to stir at -78°C for 25 minutes, then quenched with 3 mL of THF and 3 mL of 1 M aqueous HCl. The reaction was warmed to 55°C in an oil bath for 16 hours (Note: the flask was ventilated for 3 hours to allow for DCM evaporation). The reaction mixture was analyzed by ^{19}F-NMR using 2,2,2-trifluoroethanol as an internal standard to give **SI-21** in 75% yield.

^{1}H-NMR (400 MHz, $CDCl_3$): δ 4.62 – 4.36 (m, 1H), 3.12 – 2.73 (m, 4H), 1.78 – 1.13 (m, 10H), 0.92 (m, 3H).
^{13}C-NMR (100 MHz, $CDCl_3$): δ 94.1 (d, *J* = 167.2 Hz), 40.6, 34.9 (d, *J* = 20.1 Hz), 32.3 (d, *J* = 21.7 Hz), 27.4 (d, *J* = 4.5 Hz), 25.6 (d, *J* = 4.9 Hz), 22.7, 14.1.
^{19}F-NMR (376 MHz, $CDCl_3$): δ -180.3 (m) (chemical shift not referenced)
HRMS (APCI): Calcd. for $C_8H_{19}FN$ [M+H], 148.1496. Found: 148.1495.
FTIR (Film) cm^{-1}: 3367, 3188, 2924, 2855, 1259, 1049.

Analysis of Crude Fluorine Transfer Reactions

Fluorine transfer reactions were analyzed before purification to determine conversion and distribution of products. Full conversion of fluoroamide starting material was obtained in all cases, and only one fluorinated product was observed via ^{19}F-NMR analysis. Side products observed in this reaction include the formation of alkene isomers on the aliphatic chain, as well as the corresponding unfluorinated amide.

To demonstrate this, the crude ^{19}F-NMR spectra of substrates **2d** and **2e** are included below.

Crude ^{19}F-NMR of **2d** (^{19}F-NMR chemical shift not referenced).

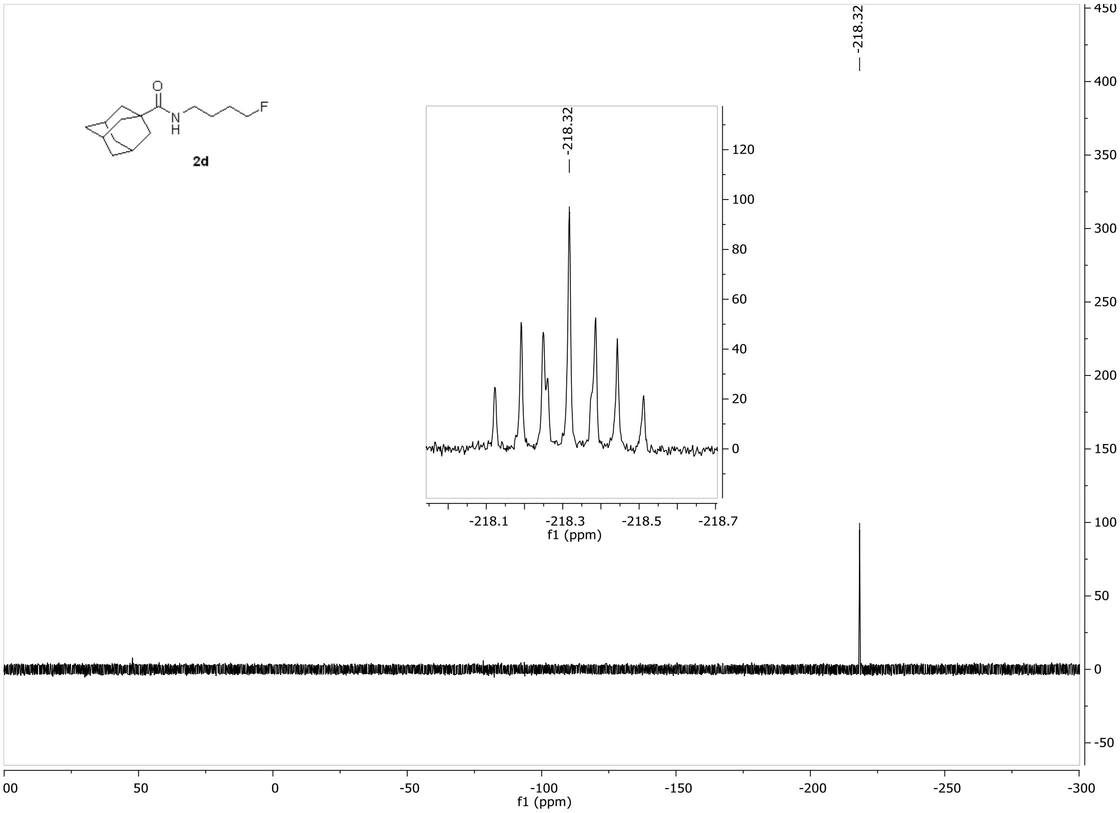

Crude ^{19}F-NMR of **2e** (^{19}F-NMR chemical shift not referenced).

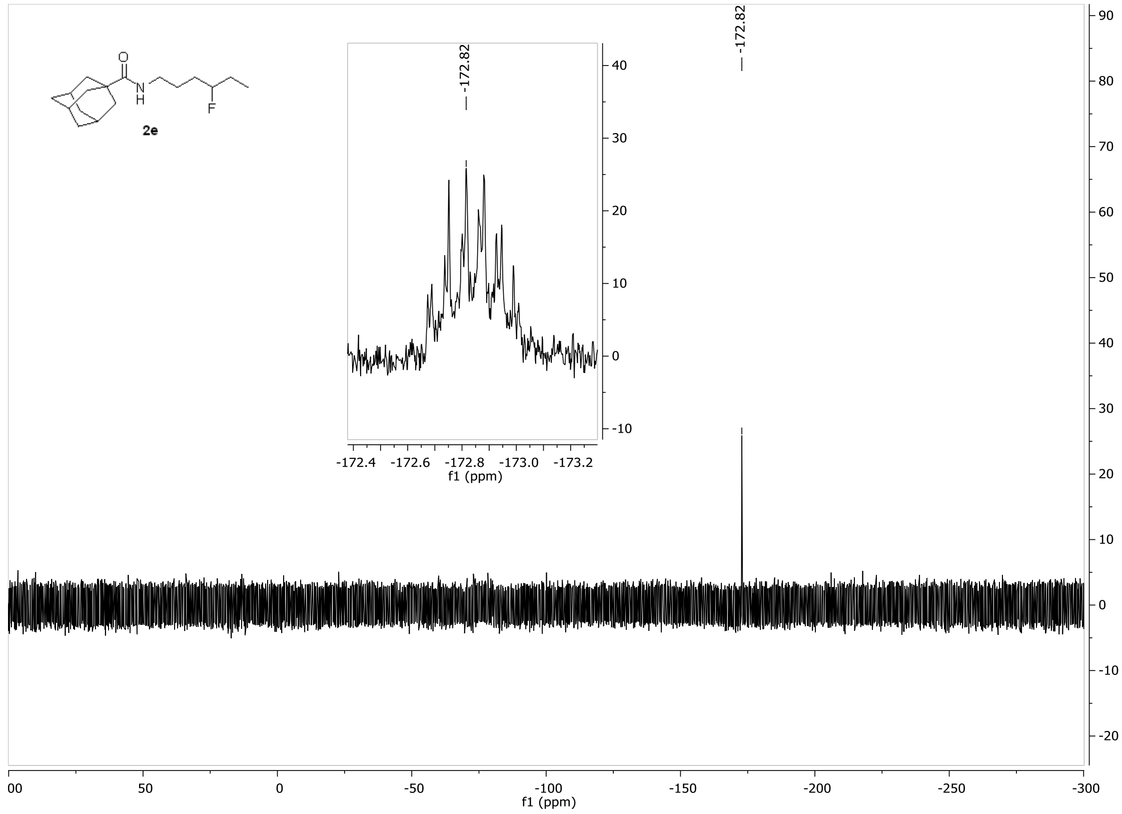

Computational Details

I. Computational Methods

All calculations were performed by using DFT, as implemented in the Jaguar 9.1 suite of ab initio quantum chemistry programs.[11] Geometry optimizations were performed with the M06 functional[12] using the 6-31G** basis set. Fe was represented by using the Los Alamos LACVP[13] basis set that included relativistic core potentials. More accurate single-point energies were computed from the optimized geometries by using Dunning's correlation-consistent triple-ζ basis set, cc-pVTZ(-f),[14] which included a double set of polarization functions. Cu was represented by using a modified version of LACVP, designated as LACV3P, in which the exponents were decontracted to match the effective core potential with triple-ζ quality. Vibrational frequencies were computed at theM06/6-31G** level of theory to derive the zero-point energy (ZPE) and vibrational entropy corrections from unscaled frequencies. Entropy herein referred specifically to the vibrational/rotational/translational entropy of the solutes because the continuum model included the entropy of the solvent implicitly. All intermediates were confirmed as local minima on the potential energy surface with zero imaginary frequencies. Transition states were confirmed to possess only one imaginary frequency. Solvation energies were evaluated by using a self-consistent reaction field (SCRF)[15] approach based on accurate numerical solutions of the linearized Poisson–Boltzmann equation. Solvation calculations were carried out on the optimized gas-phase geometries by using a dielectric constant of $\varepsilon = 7.2$ for dimethoxyethane. The change in solution-phase free energy, ΔG(sol), was calculated from Equations (S1)–(S5).

$$G(sol) = G(gas) + \Delta G(solv) \quad \text{(S1)}$$
$$G(gas) = H(gas) - TS(gas) \quad \text{(S2)}$$
$$H(gas) = E(SCF) + ZPE \quad \text{(S3)}$$
$$\Delta E(SCF) = \Sigma E(SCF) \text{ for products} - \Sigma E(SCF) \text{ for reactants} \quad \text{(S4)}$$
$$\Delta G(sol) = \Sigma G(sol) \text{ for products} - \Sigma G(sol) \text{ for reactants} \quad \text{(S5)}$$

II. Calculated 3D Geometries of the Fe Complexes Used in the Reaction Coordinate

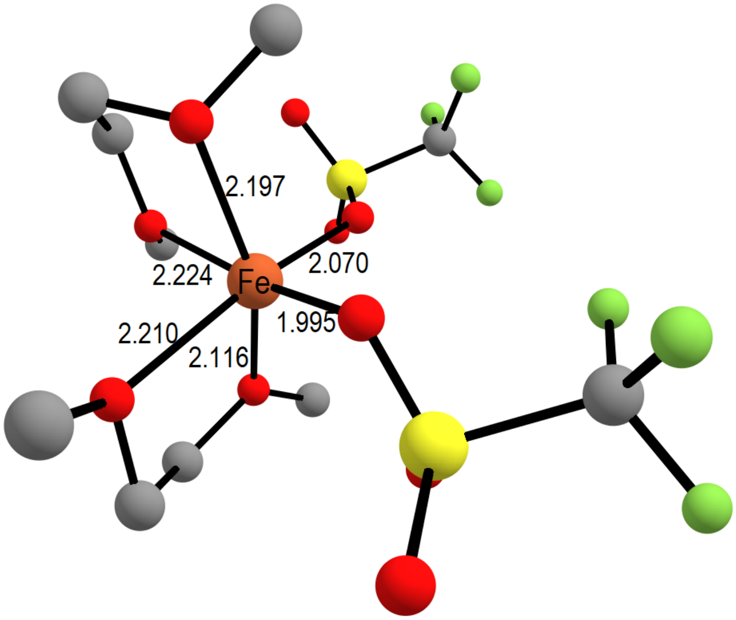

Figure SI-1. 3D-Geometry of the most stable isomer of the octahedral $Fe(OTf)_2(DME)_2$ complex, **I**. Hydrogens removed for the purpose of clarity. Bond lengths measured in angstroms.

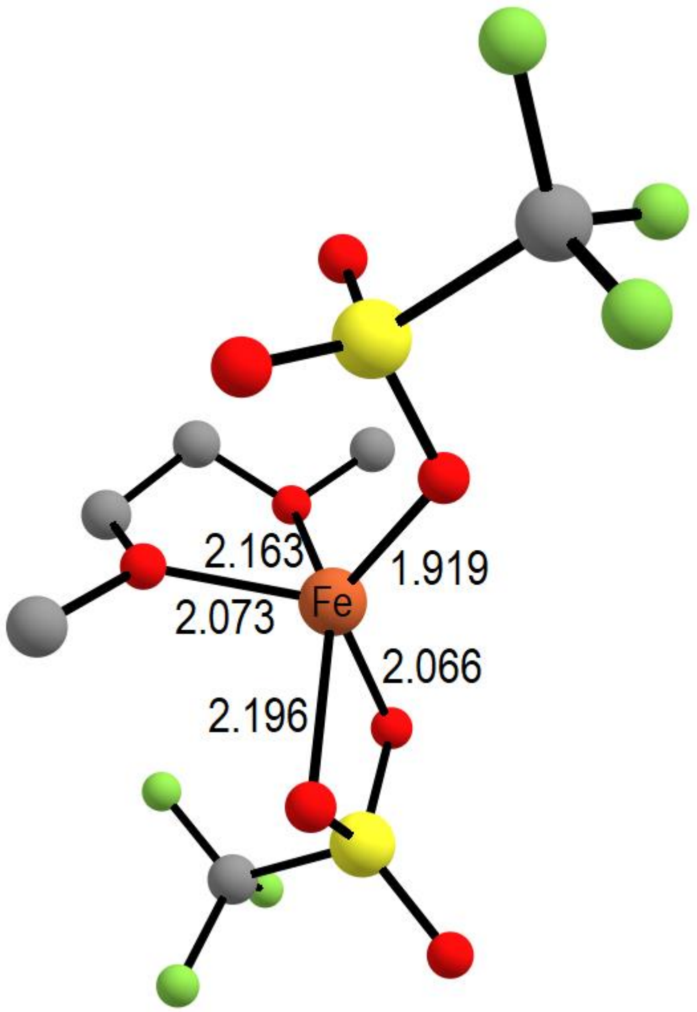

Figure SI-2. 3D-Geometry of the most stable isomer of $Fe(OTf)_2(DME)$ complex, **II**. Hydrogens removed for the purpose of clarity. Bond lengths measured in angstroms.

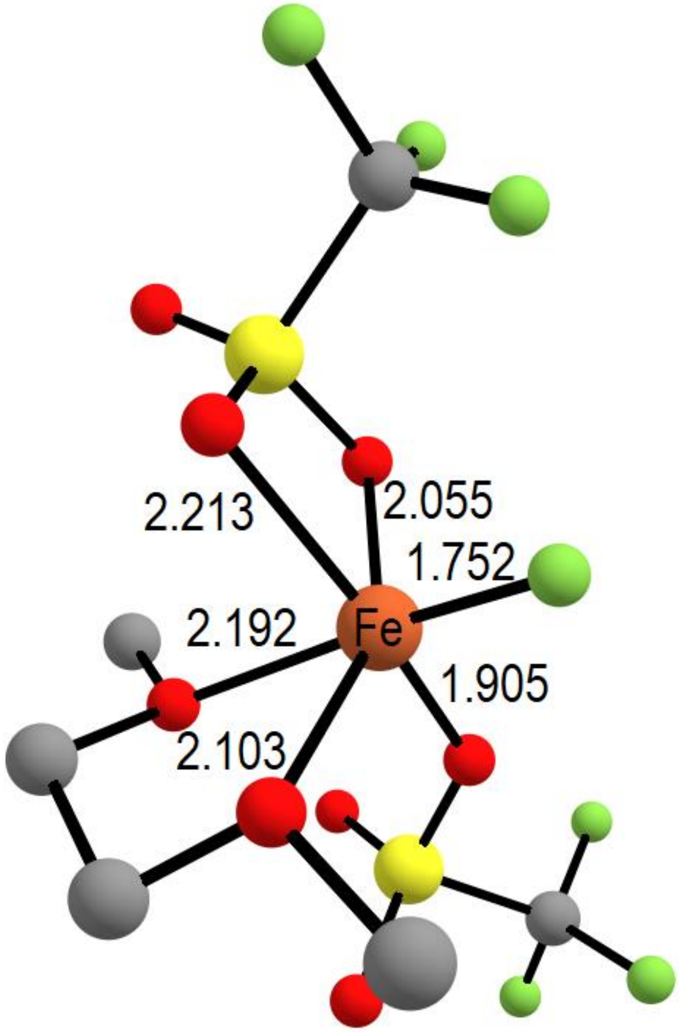

Figure SI-3. 3D-Geometry of the most stable isomer of F–Fe(OTf)$_2$(DME) complex, **Fe^{III}F**. Hydrogens removed for the purpose of clarity. Bond lengths measured in angstroms.

III. Calculated Energy Components

Table SI-4. Calculated energy components for the Fe species used in this study.

Molecule	E(SCF)/(eV) cc-pVTZ(-f)/LACV3P**	ZPE/(kcal/mol) 6-31G**/LACVP**	S(gas)/(cal/mol) 6-31G**/LACVP**	G(solv)/(kcal/mol) 6-31G**/LACVP**
**I, $Fe(OTf)_2(DME)_2$**	-72491.656	219.719	211.306	-17.83
**II, $Fe(OTf)_2(DME)$, Isomer 1**	-64088.664	128.008	175.229	-14.14
**II, $Fe(OTf)_2(DME)$, Isomer 2**	-64088.75	128.392	185.057	-14.37
**F–$Fe(OTf)_2(DME)$, Isomer 1**	-66805.836	129.998	181.721	-13.84
**F–$Fe(OTf)_2(DME)$, Isomer 2**	-66806.031	130.177	187.007	-12.58

Table SI-5. Calculated energy components for the 14 conformers for all the minima from substrate **1d** in the reaction coordinate.

Molecule	E(SCF)/(eV) cc-pVTZ(-f)/LACV3P**	ZPE/(kcal/mol) 6-31G**/LACVP**	S(gas)/(cal/mol) 6-31G**/LACVP**	G(solv)/(kcal/mol) 6-31G**/LACVP**
1d**, Conf. 1**	-22191.551	234.445	128.712	-4.47
1d**, Conf. 2**	-22191.58	234.47	128.427	-4.3
1d**, Conf. 3**	-22191.533	234.26	127.315	-4.56
1d**, Conf. 4**	-22191.566	234.452	129.626	-4.23
1d**, Conf. 5**	-22191.549	234.618	127.827	-4.29
1d**, Conf. 6**	-22191.523	234.265	131.304	-4.23
1d**, Conf. 7**	-22191.559	234.481	130.316	-4.31
1d**, Conf. 8**	-22191.588	234.74	127.646	-3.94
1d**, Conf. 9**	-22191.551	234.539	129.236	-4.24
1d, **Conf. 10**	-22191.547	234.526	130.278	-4.32
1d**, Conf. 11**	-22191.496	234.251	131.582	-4.34
1d**, Conf. 12**	-22191.516	234.406	130.798	-4.29
1d**, Conf. 13**	-22191.512	234.597	128.569	-4.14
1d**, Conf. 14**	-22191.461	234.453	127.66	-4.51
III (1°)**, Conf. 1**	-19474.965	231.171	127.91	-4.9
III (1°)**, Conf. 2**	-19474.965	231.168	125.867	-4.98

III (1°), **Conf. 3**	-19474.967	230.954	130.078	-4.9
III (1°), **Conf. 4**	-19474.945	230.945	130.019	-4.75
III (1°), **Conf. 5**	-19474.941	231.389	127.078	-4.66
III (1°), **Conf. 6**	-19474.93	230.675	131.282	-4.97
III (1°), **Conf. 7**	-19474.969	231.161	128.47	-4.34
III (1°), **Conf. 8**	-19474.932	231.259	121.67	-4.4
III (1°), **Conf. 9**	-19474.938	231.12	129.265	-4.95
III (1°), **Conf. 10**	-19474.92	231.035	130.383	-4.99
III (1°), **Conf. 11**	-19474.912	230.722	131.056	-4.96
III (1°), **Conf. 12**	-19474.914	230.757	131.248	-5
III (1°), **Conf. 13**	-19474.941	231.125	127.138	-4.72
III (1°), **Conf. 14**	-19474.861	230.968	115.884	-3.91
IV (1°), **Conf. 1**	-19475.086	230.511	130.441	-6.77
IV (1°), **Conf. 2**	-19475.098	230.347	133.296	-6.7
IV (1°), **Conf. 3**	-19475.098	230.317	127.976	-6.71
IV (1°), **Conf. 4**	-19475.066	230.455	132.478	-6.94
IV (1°), **Conf. 5**	-19475.105	230.618	123.718	-6.45
IV (1°), **Conf. 6**	-19475.006	230.224	127.456	-6.98
IV (1°), **Conf. 7**	-19475.129	230.83	122.306	-6.4
IV (1°), **Conf. 8**	-19475.121	230.385	121.6	-6.46
IV (1°), **Conf. 9**	-19475.098	230.62	130.076	-6.58
IV (1°), **Conf. 10**	-19475.096	230.603	131.079	-6.91
IV (1°), **Conf. 11**	-19475.094	230.604	130.634	-6.92
IV (1°), **Conf. 12**	-19475.035	230.419	129.453	-7.07
IV (1°), **Conf. 13**	-19475.186	231.129	125.38	-6.21
IV (1°), **Conf. 14**	-19475.121	230.801	128.357	-6.36
2d**, Conf. 1**	-22193.736	235.862	133.073	-7.99
2d**, Conf. 2**	-22193.727	235.797	133.722	-8.14

2d**, Conf. 3**	-22193.736	235.758	131.442	-8.18
2d**, Conf. 4**	-22193.715	235.793	134.894	-8.2
2d**, Conf. 5**	-22193.689	235.643	132.769	-8.02
2d**, Conf. 6**	-22193.641	235.336	128.277	-8.4
2d**, Conf. 7**	-22193.758	235.973	123.931	-7.41
2d**, Conf. 8**	-22193.756	236.082	123.017	-7.53
2d**, Conf. 9**	-22193.691	235.804	135.622	-8.16
2d**, Conf. 10**	-22193.684	235.81	134.99	-8.32
2d**, Conf. 11**	-22193.613	235.377	129.198	-8.45
2d**, Conf. 12**	-22193.633	235.487	130.107	-8.44
2d**, Conf. 13**	-22193.684	235.844	131.624	-7.91
2d**, Conf. 14**	-22193.672	235.554	131.111	-8.09

Table SI-6. Calculated energy components for the 14 conformers for all the minima from substrate **1e** in the reaction coordinate.

Molecule	E(SCF)/(eV) cc-pVTZ(-f)/LACV3P**	ZPE/(kcal/mol) 6-31G**/LACVP**	S(gas)/(cal/mol) 6-31G**/LACVP**	G(solv)/(kcal/mol) 6-31G**/LACVP**
1e**, Conf. 1**	-23260.764	252.17	136.249	-4.39
1e**, Conf. 2**	-23260.785	252.259	135.747	-4.27
1e**, Conf. 3**	-23260.742	251.991	137.326	-4.52
1e**, Conf. 4**	-23260.779	252.184	136.821	-4.22
1e**, Conf. 5**	-23260.758	252.374	135.67	-4.31
1e**, Conf. 6**	-23260.734	252.026	138.662	-4.19
1e**, Conf. 7**	-23260.828	252.619	132.232	-3.81
1e**, Conf. 8**	-23260.826	252.533	132.469	-3.83
1e**, Conf. 9**	-23260.764	252.278	136.647	-4.3
1e**, Conf. 10**	-23260.754	252.233	131.257	-4.4
1e**, Conf. 11**	-23260.707	252.005	139.297	-4.41
1e**, Conf. 12**	-23260.721	252.166	137.748	-4.34
1e**, Conf. 13**	-23260.727	252.266	136.444	-4.34
1e**, Conf. 14**	-23260.674	252.188	134.957	-4.68
III (2°)**, Conf. 1**	-20544.176	248.896	135.637	-4.93
III (2°)**, Conf. 2**	-20544.195	249.047	131.614	-5.01
III (2°)**, Conf. 3**	-20544.174	248.692	137.271	-4.89
III (2°)**, Conf. 4**	-20544.158	248.696	137.596	-4.87
III (2°)**, Conf. 5**	-20544.146	249.091	135.526	-4.72

III (2°)**, Conf. 6**	-20544.141	248.377	138.639	-4.91
III (2°)**, Conf. 7**	-20544.182	248.866	136.958	-4.36
III (2°)**, Conf. 8**	-20544.184	249.221	132.223	-4.73
III (2°)**, Conf. 9**	-20544.145	248.854	136.98	-5
III (2°)**, Conf. 10**	-20544.127	248.773	139.005	-4.99
III (2°)**, Conf. 11**	-20544.121	248.455	138.527	-5.11
III (2°)**, Conf. 12**	-20544.119	248.504	138.643	-5.08
III (2°)**, Conf. 13**	-20544.152	248.775	134.914	-4.88
III (2°)**, Conf. 14**	-20544.098	248.959	136.414	-4.27
IV (2°)**, Conf. 1**	-20544.457	248.278	133.917	-6.68
IV (2°)**, Conf. 2**	-20544.461	246.221	130.086	-6.6
IV (2°)**, Conf. 3**	-20544.475	248.439	134.072	-6.66
IV (2°)**, Conf. 4**	-20544.439	248.258	142.212	-6.87
IV (2°)**, Conf. 5**	-20544.502	248.486	132.56	-6.22
IV (2°)**, Conf. 6**	-20544.367	245.899	130.968	-6.85
IV (2°)**, Conf. 7**	-20544.561	247.975	128.244	-5.95
IV (2°)**, Conf. 8**	-20544.549	247.295	126.673	-5.84
IV (2°)**, Conf. 9**	-20544.467	246.12	130.867	-6.62
IV (2°)**, Conf. 10**	-20544.447	246.179	131.582	-6.88
IV (2°)**, Conf. 11**	-20544.447	246.447	130.502	-6.87
IV (2°)**, Conf. 12**	-20544.402	248.279	134.825	-6.99
IV (2°)**, Conf. 13**	-20544.576	248.83	133.53	-6.23
IV (2°)**, Conf. 14**	-20544.5	248.667	137.787	-6.18
2e**, Conf. 1**	-23263.154	253.174	138.735	-7.99
2e**, Conf. 2**	-23263.18	253.195	138.801	-7.91
2e**, Conf. 3**	-23263.189	253.214	132.156	-7.79
2e**, Conf. 4**	-23263.117	253.108	140.965	-8.39
2e**, Conf. 5**	-23263.139	253.255	137.454	-7.74
2e**, Conf. 6**	-23263.088	252.865	138.415	-8.13

2e, **Conf. 7**	-23263.254	253.411	135.518	-6.84
2e, **Conf. 8**	-23263.227	253.512	133.554	-7.02
2e, **Conf. 9**	-23263.084	253.189	141.469	-8.48
2e, **Conf. 10**	-23263.123	253.185	133.677	-8.02
2e, **Conf. 11**	-23263.068	252.923	136.91	-8.1
2e, **Conf. 12**	-23263.115	253.198	139.672	-8.05
2e, **Conf. 13**	-23263.223	253.669	127.535	-6.94
2e, **Conf. 14**	-23263.096	253.148	134.054	-8

Table SI-7. Calculated energy components for the 14 conformers for all the minima from substrate **1m** in the reaction coordinate.

Molecule	E(SCF)/(eV) cc-pVTZ(-f)/LACV3P**	ZPE/(kcal/mol) 6-31G**/LACVP**	S(gas)/(cal/mol) 6-31G**/LACVP**	G(solv)/(kcal/mol) 6-31G**/LACVP**
1m, **Conf. 1**	-24330.012	269.584	135.497	-4.57
1m, **Conf. 2**	-24330.045	269.74	142.912	-4.46
1m, **Conf. 3**	-24329.992	269.496	144.806	-4.77
1m, **Conf. 4**	-24330.033	269.744	144.797	-4.53
1m, **Conf. 5**	-24330.027	269.929	141.938	-4.43
1m, **Conf. 6**	-24329.992	269.582	144.817	-4.49
1m, **Conf. 7**	-24330.051	269.821	141.806	-4.43
1m, **Conf. 8**	-24330.09	270.032	139.448	-3.98
1m, **Conf. 9**	-24330.059	269.967	141.774	-4.1
1m, **Conf. 10**	-24330.031	269.904	135.763	-4.47
1m, **Conf. 11**	-24329.982	269.625	144.106	-4.48
1m, **Conf. 12**	-24329.994	269.792	143.461	-4.45
1m, **Conf. 13**	-24330.029	269.88	140.821	-4.38
1m, **Conf. 14**	-24329.99	269.746	141.069	-4.6
III (3°), **Conf. 1**	-21613.445	266.516	140.549	-5.04
III (3°), **Conf. 2**	-21613.473	266.517	138.231	-5.14
III (3°), **Conf. 3**	-21613.426	266.236	143.831	-5.03
III (3°), **Conf. 4**	-21613.412	266.256	143.859	-5.15
III (3°), **Conf. 5**	-21613.42	266.617	138.755	-4.78
III (3°), **Conf. 6**	-21613.395	265.942	145.548	-5.2
III (3°), **Conf. 7**	-21613.451	266.443	142.335	-4.56
III (3°), **Conf. 8**	-21613.469	266.754	137.758	-4.78

III (3°), **Conf. 9**	-21613.441	266.537	140.466	-4.82
III (3°), **Conf. 10**	-21613.4	266.448	142.367	-5.14
III (3°), **Conf. 11**	-21613.391	266.152	142.323	-5.2
III (3°), **Conf. 12**	-21613.396	266.159	144.112	-5.18
III (3°), **Conf. 13**	-21613.436	266.262	141.993	-4.6
III (3°), **Conf. 14**	-21613.33	266.482	133.559	-4.45
IV (3°), **Conf. 1**	-21613.834	266.179	148.122	-6.72
IV (3°), **Conf. 2**	-21613.84	266.201	147.099	-6.72
IV (3°), **Conf. 3**	-21613.84	266.201	147.099	-6.72
IV (3°), **Conf. 4**	-21613.814	266.261	141.913	-6.97
IV (3°), **Conf. 5**	-21613.93	266.37	136.213	-6.02
IV (3°), **Conf. 6**	-21613.756	265.912	137.696	-7
IV (3°), **Conf. 7**	-21613.758	265.909	137.597	-7
IV (3°), **Conf. 8**	-21613.926	264.964	132.814	-5.7
IV (3°), **Conf. 9**	-21613.896	265.895	136.923	-6.34
IV (3°), **Conf. 10**	-21613.855	265.261	135.333	-6.85
IV (3°), **Conf. 11**	-21613.855	266.11	148.528	-6.88
IV (3°), **Conf. 12**	-21613.863	266.128	142.748	-6.5
IV (3°), **Conf. 13**	-21613.994	266.636	137.598	-5.82
IV (3°), **Conf. 14**	-21613.938	266.316	140.26	-6.05
2m, **Conf. 1**	-24332.551	270.599	142.212	-8.08
2m, **Conf. 2**	-24332.586	270.439	137.727	-7.86
2m, **Conf. 3**	-24332.588	270.475	145.827	-7.9
2m, **Conf. 4**	-24332.52	270.351	147.228	-8.61
2m, **Conf. 5**	-24332.633	270.795	140.914	-6.9
2m, **Conf. 6**	-24332.49	270.096	140.878	-8.33
2m, **Conf. 7**	-24332.502	270.639	135.95	-7.05
2m, **Conf. 8**	-24332.492	270.711	138.939	-8.39
2m, **Conf. 9**	-24332.564	270.703	142.568	-7.57
2m, **Conf. 10**	-24332.453	270.638	138.194	-7.91

2m, **Conf. 11**	-24332.441	270.138	142.586	-8.5
2m, **Conf. 12**	-24332.447	270.269	142.01	-8.56
2m, **Conf. 13**	-24332.594	271.091	137.351	-6.49
2m, **Conf. 14**	-24332.604	270.724	140.725	-6.93

Table SI-8. Calculated energy components for the transition-states investigated in this study.

Molecule	E(SCF)/(eV) cc-pVTZ(-f)/LACV3P**	ZPE/(kcal/mol) 6-31G**/LACVP**	S(gas)/(cal/mol) 6-31G**/LACVP**	G(solv)/(kcal/mol) 6-31G**/LACVP**
**Piv, 1,4-TS**	-13156.574	158.334	116.511	-5.24
**Piv, 1,5-TS**	-13156.725	158.667	112.208	-5.98
**Ad, 1,4-TS**	-19473.758	228.063	125.733	-6.07
1° N-F Abstraction **(II-TS)**	-86280.406	361.719	259.826	-13.78
2° N-F Abstraction (I**I-TS**)	-87349.633	379.42	266.665	-13.69
3° N-F Abstraction (I**I-TS**)	-88418.898	396.896	274.96	-13.74
1° C-H Abstraction **(III-TS**)	-19474.209	228.273	121.001	-5.97
2° C-H Abstraction **(III-TS**)	-20543.572	245.968	127.877	-6.19
3° C-H Abstraction **(III-TS**)	-21612.951	263.36	134.978	-6.29
1° C-H Chain Transfer **(IV-TS**)	-41666.113	465.625	198.326	-12.61
2° C-H Chain Transfer **(IV-TS**)	-43804.805	500.779	217.768	-11.09
3° C-H Chain Transfer **(IV-TS**)	-45931.453	-45943.699	219.301	-11.08

IV. Cartesian Coordinates of the Minimized Geometries

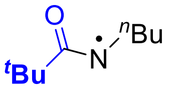

C	-2.083809178	-1.063956140	-1.324390983
H	-1.778888615	-1.456214343	-2.301376504
H	-2.072008778	-1.898887960	-0.616232680
C	-1.172905267	1.210212866	-1.871220650
H	-0.864548926	0.873697605	-2.869569385
H	-0.502316870	2.019926242	-1.557051439
C	-1.598613615	0.569253237	0.511931006
H	-1.584422790	-0.238514288	1.253858367

H	-0.943819474	1.373256538	0.864740112
C	-1.163422980	0.057288015	-0.865271596
C	0.257214879	-0.471820612	-0.747378583
O	0.558983046	-1.656759377	-0.766154837
N	1.222519550	0.489076606	-0.496182794
C	2.498072549	0.338030909	-1.135318583
H	2.540901658	-0.584087293	-1.738001266
H	2.606953899	1.195961580	-1.822246473
C	3.625484768	0.365035673	-0.107943707
H	3.568898487	1.304747109	0.460670583
H	4.588309304	0.370823539	-0.640218294
C	3.558400987	-0.821365388	0.842411571
H	2.585851170	-0.809477003	1.355949879
H	3.570242822	-1.753948320	0.258977552
C	4.689357554	-0.814340456	1.855553873
H	4.668875486	0.099039107	2.463696512
H	4.629724299	-1.668626239	2.538150777
H	5.667541238	-0.854001440	1.359051797
H	-2.624202006	0.956231960	0.453501043
H	-3.112165453	-0.691190755	-1.408973877
H	-2.185131951	1.625155520	-1.956483747

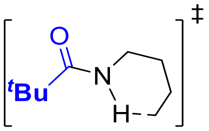

C	-0.950507867	-0.039645423	-1.060593253
C	0.473315265	-0.350860710	-1.526115599
O	0.671492652	-0.912700274	-2.597528547
N	1.522288278	0.167821171	-0.799096321
C	2.857297056	-0.246032238	-1.191816026
H	2.874079946	-1.303342062	-1.502624011
H	3.144338927	0.338611381	-2.077980931
C	3.839095073	0.004857495	-0.056885260
H	3.868687308	1.080063310	0.173134080
H	4.846760647	-0.287780828	-0.373492410
C	3.410644276	-0.774939850	1.187798338
H	3.393573525	-1.848158453	0.950776134
H	4.151360888	-0.642228829	1.990748779
C	2.048184460	-0.320252459	1.633995402
H	1.492557949	-0.024444335	0.509299353
H	1.418582028	-1.057572621	2.137054227
H	2.024429269	0.653762268	2.134338019
C	-1.811216068	-1.282662610	-1.277832609
H	-1.728811197	-1.632163092	-2.311181631
H	-1.502757378	-2.101938383	-0.615987397
H	-2.863241935	-1.054890024	-1.063852671
C	-1.441665107	1.088182666	-1.977118615
H	-0.835542953	1.993285857	-1.843861751
H	-1.382329555	0.783824009	-3.027548086
H	-2.484833769	1.336540758	-1.741798486
C	-1.052677424	0.416065815	0.390183099
H	-2.094488152	0.675312472	0.617173898
H	-0.754698081	-0.374626080	1.090491172

H	-0.437433588	1.302970084	0.583202991

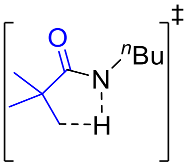

C	-1.572960509	-1.304892689	-0.205059146
H	-1.263126484	-2.214836883	-0.732435794
H	-1.152463309	-1.336146533	0.807719511
C	-1.678840310	-0.026680192	-2.362213220
H	-1.339091442	-0.901448725	-2.929122564
H	-1.357903561	0.869334752	-2.906680847
C	-1.312713872	1.203858542	-0.168819652
H	-1.934956558	1.139760064	0.727542177
H	-0.086919594	1.278899444	0.252405891
C	-1.090518065	-0.065534730	-0.958347500
C	0.444527876	-0.152554559	-1.061498455
O	1.066562623	-0.828828140	-1.861337709
N	1.014785290	0.596426339	-0.072243869
C	2.370000100	1.092219473	-0.138190138
H	2.740035816	0.987192492	-1.170607649
H	2.346571728	2.167407714	0.092548844
C	3.297825606	0.374383935	0.833424107
H	2.924594050	0.515660739	1.859166702
H	4.286385139	0.857378382	0.791811381
C	3.437096652	-1.111431328	0.537715828
H	2.447735586	-1.585022259	0.603811378
H	3.756780822	-1.246043922	-0.506010983
C	4.409672635	-1.795488741	1.483303229
H	4.088773047	-1.685860198	2.527292842
H	4.496657860	-2.867023586	1.274448022
H	5.413912897	-1.359456672	1.404228394
H	-1.499225617	2.105248176	-0.758599613
H	-2.667160787	-1.305285976	-0.126254257
H	-2.774434255	-0.032423720	-2.324401029

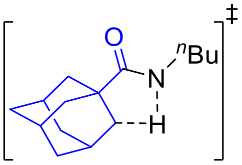

C	-3.754917500	0.165697935	-2.259132740
C	-2.772359392	1.338919062	-2.309716837
C	-2.958147674	2.246981710	-1.090268476
C	-2.719020144	1.446958033	0.191326650
C	-3.709388085	0.251002115	0.228853858
C	-3.504094422	-0.660208540	-0.993128454
H	-3.632418890	-0.463660495	-3.152866389
H	-4.790487451	0.537365411	-2.266142104
H	-2.933522242	1.919604838	-3.228856408
H	-3.974812088	2.666370917	-1.077336343
H	-2.260722706	3.095681696	-1.144162283

H	-2.876656317	2.077178109	1.076875073
H	-4.740245413	0.636489535	0.235860391
H	-3.572766800	-0.317761096	1.159759888
H	-4.224580730	-1.488734980	-0.944880314
C	-2.073704171	-1.231905049	-1.018584401
H	-1.910403402	-1.857641410	-1.908551736
H	-1.894984268	-1.865538405	-0.137695887
C	-1.348232215	0.795217075	-2.292691944
H	-1.145573523	0.161712382	-3.168027849
H	-0.624496383	1.624763267	-2.321964499
C	-1.366657004	0.782294494	0.216589402
H	-1.223426202	0.192015545	1.134086016
H	-0.158330853	1.279966807	0.211613627
C	-1.109944354	-0.048198454	-1.019850512
C	0.384327501	-0.306932534	-1.006299111
O	0.988782101	-1.114809863	-1.694501652
N	0.977264165	0.583332802	-0.152740006
C	2.321747644	1.078943599	-0.326213855
H	2.663397470	0.821571585	-1.342760180
H	2.292166704	2.176891391	-0.264694578
C	3.288698519	0.526134007	0.712785841
H	2.932465669	0.800919065	1.717503401
H	4.263804320	1.020996725	0.582406139
C	3.460293324	-0.982890425	0.622914755
H	2.481212305	-1.463518910	0.752578586
H	3.783273574	-1.251253965	-0.393544308
C	4.446493220	-1.512314708	1.650409822
H	4.118719031	-1.276196330	2.671142722
H	4.561432840	-2.599653552	1.583280650
H	5.440169453	-1.064991326	1.516703844

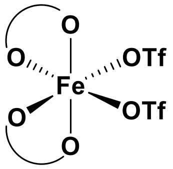

Fe	-0.141871369	-0.116932389	0.033930676
O	-0.481714051	-0.039271795	-2.006666927
S	0.458483354	0.114840629	-3.166041474
O	0.902517779	1.490211772	-3.369477140
O	1.490415699	-0.927487209	-3.214627271
C	-0.663055779	-0.288913293	-4.556978483
F	-1.678905097	0.557392429	-4.590039512
F	-1.126467549	-1.523771312	-4.403134547
F	0.003475383	-0.216244953	-5.698585351
O	-2.028202595	-0.638329711	0.420508551
S	-3.097245963	0.367147904	0.807875842
O	-3.491780645	0.235392307	2.206599463
O	-2.812298572	1.709224347	0.310830761
C	-4.508422524	-0.248424338	-0.187890607
F	-4.798666401	-1.492615258	0.161570097
F	-4.198758458	-0.215682140	-1.473244466
F	-5.560725963	0.524190202	0.030013002
O	0.353863883	-2.257167759	0.050124582

C	1.720662053	-2.436930256	0.392153541
H	1.796718299	-2.227099749	1.467196905
H	2.025237189	-3.478570351	0.217049219
O	2.071984541	-0.176065752	-0.169621671
C	2.597328339	-1.485179591	-0.380786437
H	2.592952218	-1.697125675	-1.459260394
H	3.631016360	-1.533461584	-0.003737156
O	0.161368069	0.132986454	2.208407624
C	-0.179167351	1.464352542	2.581996077
H	-1.275127032	1.572561305	2.597102007
H	0.208891875	1.680601846	3.589630415
C	0.439276559	2.404372930	1.577199099
H	0.053356473	3.418172656	1.748883017
H	1.537209910	2.424682556	1.672645245
O	0.104352502	1.971993723	0.267288305
C	-0.072826188	-2.995205419	-1.101623775
H	0.510244178	-2.719722662	-1.989431048
H	0.015069497	-4.069084474	-0.893494245
H	-1.119584956	-2.732177517	-1.266839307
C	2.867699741	0.826487477	-0.799179113
H	3.849973421	0.875712671	-0.310107984
H	2.984021762	0.609151333	-1.867562389
H	2.342578903	1.777424062	-0.685421771
C	-0.416437773	-0.837714403	3.081141965
H	-1.499318901	-0.681539266	3.166141663
H	-0.236003435	-1.818514096	2.633424498
H	0.060272779	-0.777229075	4.068511103
C	-0.244789042	3.006124211	-0.657012468
H	0.574302519	3.735579062	-0.719108137
H	-0.380681329	2.530263711	-1.629944601
H	-1.175736083	3.483318140	-0.333730452

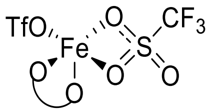

Fe	-0.385667873	0.354233315	0.484538882
O	1.124609585	0.169300986	-0.685022746
S	2.449717931	0.809420851	-0.249648046
O	2.452506849	2.245625288	-0.511646450
O	2.876187564	0.336974260	1.057964903
C	3.571771306	0.053724138	-1.489011394
F	3.189386443	0.398954093	-2.706064521
F	3.546734703	-1.260852818	-1.370210374
F	4.798660982	0.495821927	-1.269090780
O	-0.946718019	-1.540232814	1.443217769
S	-2.345369747	-1.424686425	0.924437062
O	-3.010793894	-2.597504999	0.415740565
O	-2.336207639	-0.182755011	0.064789277
C	-3.294940217	-0.866067492	2.395595363
F	-3.306615999	-1.819941001	3.306624398
F	-4.529095962	-0.563634849	2.044894660
F	-2.709070558	0.217684920	2.909637166
O	0.156615636	1.167726428	2.312177970

C	-0.191594307	2.522949503	2.560572527
H	-1.202397937	2.560770660	2.995868526
H	0.520356921	2.967126802	3.270157421
C	-0.120327281	3.237802179	1.235441372
H	-0.560165963	4.242705830	1.315806788
H	0.920865041	3.319521659	0.887126789
O	-0.865406106	2.455839273	0.303843997
C	0.548518457	0.416597954	3.457961871
H	-0.255663739	0.421569512	4.204834636
H	0.734276643	-0.604370115	3.121134535
H	1.468435110	0.840743993	3.875816496
C	-0.853132365	3.001775089	-1.014796575
H	0.176713575	3.051673803	-1.392206500
H	-1.454857814	2.334532013	-1.636823315
H	-1.304254429	4.002166963	-1.005961489

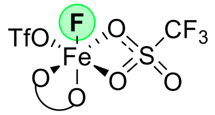

Fe	-0.515001310	0.323890893	-0.209577968
O	1.312444273	0.179512710	-0.727847240
S	2.594125794	0.739122031	-0.075737811
O	2.582824397	2.196696698	-0.079457650
O	2.959492184	0.023564266	1.131586821
C	3.796911194	0.238736031	-1.369929766
F	3.479137857	0.809483334	-2.518856106
F	3.787755328	-1.073378819	-1.506347449
F	4.999832868	0.639319828	-0.995123703
O	-0.860325138	-1.543490508	0.575356338
S	-2.212958456	-1.244231593	1.193382328
O	-2.419978130	-1.687494351	2.552487186
O	-2.438051664	0.203047514	0.878829853
C	-3.402708572	-2.172623881	0.138593766
F	-3.178730788	-3.463027670	0.292143001
F	-3.243822047	-1.831081572	-1.121554169
F	-4.625710629	-1.876212423	0.536602490
O	0.174181732	1.005153765	1.757102165
C	-0.440661139	2.238824102	2.108700557
H	-1.501857822	2.062795465	2.340287623
H	0.059758732	2.667798160	2.987586716
C	-0.260011722	3.141310147	0.919140196
H	-0.849702124	4.062433759	1.023352066
H	0.801914393	3.384467964	0.778047969
O	-0.718099675	2.417155368	-0.226293117
C	0.410246926	0.134676360	2.868784839
H	-0.532896163	-0.088350512	3.384723242
H	0.852147741	-0.778917459	2.469479791
H	1.121985999	0.614506630	3.549651449
C	-0.401713274	3.072503817	-1.462877994
H	0.687408796	3.157498705	-1.563746251
H	-0.814996543	2.450503045	-2.257225826
H	-0.869542743	4.063686986	-1.471234755
F	-1.249183580	0.219790864	-1.796412933

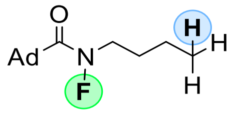

Conformer 1

C	-2.258064819	1.460864238	-2.251262634
C	-2.346037504	0.453066238	-1.100681744
C	-2.851765254	-0.888738742	-1.634151369
C	-1.884464660	-1.407084401	-2.701727933
C	-1.812367258	-0.403459837	-3.855393162
C	-1.300467477	0.938215025	-3.327274339
H	-1.908514133	2.433876587	-1.874436358
H	-3.256412280	1.623386428	-2.684137227
H	-3.033615368	0.830456677	-0.330209439
H	-3.859453502	-0.769677610	-2.059968519
H	-2.932950523	-1.615582009	-0.812115005
H	-2.235171496	-2.378676623	-3.077638094
H	-2.806105943	-0.276603508	-4.310887035
H	-1.142462757	-0.779711284	-4.643012277
H	-1.240660998	1.663976545	-4.150870495
C	0.095906868	0.759524579	-2.722743958
H	0.483616064	1.726022115	-2.382542828
H	0.793280178	0.379662615	-3.485068831
C	-0.956987288	0.275185971	-0.481161741
H	-0.606965435	1.230884988	-0.071340845
H	-0.995916743	-0.446728238	0.350666903
C	-0.495009486	-1.583473757	-2.086784996
H	0.210518356	-1.979689755	-2.830961322
H	-0.522897316	-2.316064751	-1.269211024
C	0.031017370	-0.240678465	-1.551663889
C	1.388467984	-0.495226705	-0.914311566
O	1.851757258	-1.609126095	-0.736704446
N	2.197648290	0.576759667	-0.558183775
F	1.563885732	1.813328545	-0.421372612
C	3.243910554	0.445199373	0.445650303
H	3.910914719	-0.335623028	0.068605456
H	3.795208770	1.393160174	0.428526471
C	2.751816705	0.101671259	1.843910841
H	3.637659357	-0.026726639	2.484592175
H	2.256656677	-0.879778206	1.810767150
C	1.823127013	1.134306206	2.470122139
H	2.289825522	2.129689730	2.417236964
H	0.899628985	1.207740213	1.879695443
C	1.486142896	0.788478970	3.910971186
H	2.390227515	0.754704742	4.532162662
H	0.802978905	1.518714528	4.357514156
H	1.008601981	-0.197380951	3.977120122

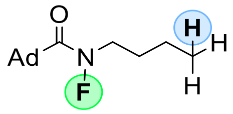

Conformer 2

C	-3.181302028	-1.424128342	0.634409433
C	-2.039695125	-1.848814291	-0.291611715
C	-2.545290379	-1.898329163	-1.737519639
C	-3.061137195	-0.516346646	-2.152549105
C	-4.199909141	-0.095056666	-1.221187636
C	-3.681748623	-0.038379715	0.218213822
H	-2.833934532	-1.403205033	1.678372071
H	-4.001810920	-2.155673735	0.582357773
H	-1.668502105	-2.840698474	0.004142530
H	-3.350235263	-2.643076336	-1.828166592
H	-1.734879678	-2.216530589	-2.410741198
H	-3.423438706	-0.554408055	-3.190023500
H	-5.034251223	-0.808804071	-1.296690316
H	-4.589711808	0.889082226	-1.522051524
H	-4.490622727	0.280568937	0.891244675
C	-2.534235692	0.968949128	0.309988948
H	-2.162200693	1.040540723	1.340991095
H	-2.881031721	1.975957784	0.038568348
C	-0.889787457	-0.841631410	-0.189609826
H	-0.524633319	-0.789833183	0.848667785
H	-0.050552211	-1.164686981	-0.817106640
C	-1.917403961	0.498893560	-2.066293935
H	-2.273663498	1.498379696	-2.360472431
H	-1.117347174	0.222487498	-2.762289044
C	-1.380277352	0.558479009	-0.621444464
C	-0.272703146	1.593860861	-0.466603343
O	-0.307667610	2.493278420	0.355983352
N	0.794295929	1.586595553	-1.359564804
F	1.005239844	0.408273597	-2.071971012
C	2.065191573	2.235762165	-1.094505268
H	2.463163132	2.594876593	-2.051052876
H	1.795978066	3.106279620	-0.487441391
C	3.074978075	1.350889912	-0.379595063
H	3.987435567	1.947156204	-0.230016797
H	3.361211342	0.517451176	-1.036910958
C	2.589916740	0.808275141	0.956898394
H	2.229294190	1.637200733	1.582907414
H	1.718179038	0.157243108	0.789195125
C	3.672873528	0.026822696	1.680948850
H	4.539735493	0.663043130	1.900127378
H	3.313956033	-0.383736581	2.630558646
H	4.029451728	-0.811834540	1.069317128

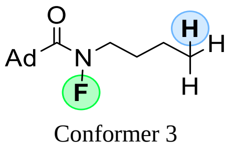

Conformer 3

C	-3.782441097	0.741945954	-2.147858978
C	-2.776263417	1.836675232	-1.785342314
C	-2.950445547	2.224191990	-0.313450521
C	-2.724132601	0.997154704	0.575689371
C	-3.730677516	-0.094576287	0.206120424
C	-3.540101288	-0.483173869	-1.262432830
H	-3.677387296	0.468779683	-3.208494288
H	-4.809644661	1.112254906	-2.010554774
H	-2.941834726	2.718304073	-2.420878202
H	-3.961299618	2.626128288	-0.148364538
H	-2.239404742	3.020410120	-0.046137549
H	-2.851684344	1.278172841	1.630722322
H	-4.757378633	0.264402157	0.374014013
H	-3.586999928	-0.973871946	0.851802804
H	-4.249689922	-1.278826734	-1.529651813
C	-2.114345135	-0.994125911	-1.476648178
H	-1.964250543	-1.299750665	-2.521032553
H	-1.927837994	-1.888025177	-0.865025514
C	-1.351257384	1.322835404	-2.010114652
H	-1.214592968	1.032072479	-3.063036618
H	-0.626065769	2.116026114	-1.793250590
C	-1.298535756	0.477884435	0.368758830
H	-1.120767234	-0.409177461	0.997019091
H	-0.573970767	1.241569804	0.673436460
C	-1.091831145	0.095396517	-1.111406505
C	0.297926872	-0.477715392	-1.353289091
O	0.499325967	-1.580750790	-1.832866884
N	1.396886560	0.243581628	-0.937087461
F	1.228712430	1.583852412	-0.612440166
C	2.782337678	-0.040461378	-1.222141001
H	2.785956333	-1.091926252	-1.530282712
H	3.113787455	0.563175467	-2.079901092
C	3.667384503	0.189242329	-0.006829265
H	3.704689268	1.264034209	0.222656121
H	4.693374491	-0.101249549	-0.275609743
C	3.211502558	-0.582796946	1.223333667
H	2.208258218	-0.237039419	1.511900670
H	3.099314710	-1.646560588	0.964456816
C	4.172005605	-0.423530059	2.390085854
H	4.278612386	0.631885287	2.670962152
H	3.830488300	-0.969055037	3.276063305
H	5.172290199	-0.796347917	2.135628841

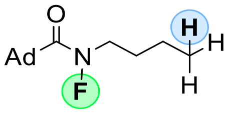

Conformer 4

C	-3.389135409	-2.005161533	-0.452319753
C	-2.622506141	-1.101838981	-1.424353951
C	-3.406111951	0.193999731	-1.644676933
C	-3.584301594	0.913381484	-0.304981293
C	-4.364507666	0.013122928	0.655858556
C	-3.582767065	-1.281709889	0.884945945
H	-2.838787779	-2.945253077	-0.296890591
H	-4.367127555	-2.271274311	-0.880386371
H	-2.486046840	-1.622380144	-2.383181293
H	-4.388308486	-0.028907774	-2.087911138
H	-2.871760538	0.842735960	-2.354779028
H	-4.133112503	1.853236730	-0.460335519
H	-5.358626815	-0.212665929	0.241402160

H	-4.525419889	0.531451664	1.613067973
H	-4.134901538	-1.931839089	1.578676075
C	-2.213625618	-0.960073329	1.492278897
H	-1.668425730	-1.886635352	1.698991944
H	-2.338032630	-0.434284903	2.451352014
C	-1.245976044	-0.777699338	-0.836650811
H	-0.658799844	-1.695704802	-0.714793388
H	-0.685862951	-0.117207050	-1.518012803
C	-2.212116774	1.231410109	0.290259432
H	-2.318899937	1.771039828	1.242296226
H	-1.643943862	1.893720528	-0.376222627
C	-1.415245034	-0.063733768	0.524594415
C	-0.045461462	0.321885433	1.055760142
O	0.366775777	1.467881960	1.079843426
N	0.790339111	-0.658055529	1.602965223
F	0.491702939	-1.972407703	1.221141073
C	2.233182991	-0.492529442	1.543775221
H	2.426086438	0.502305989	1.957527706
H	2.669494050	-1.231995537	2.226504145
C	2.801927073	-0.620726113	0.140359966
H	2.327587110	0.134825328	-0.504296061
H	2.533244122	-1.604281893	-0.271807656
C	4.313041636	-0.442402866	0.123621974
H	4.565718463	0.541053885	0.547875421
H	4.774832528	-1.185238890	0.792094919
C	4.896984576	-0.569048548	-1.272960682
H	4.469856759	0.183792141	-1.947114854
H	5.984222581	-0.438398087	-1.275049199
H	4.678969750	-1.554617299	-1.703231714

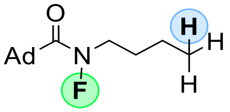

Conformer 5

C	-3.285127700	0.717532435	-0.131794660
C	-3.140147192	0.308186797	1.337699802
C	-3.894724950	-1.001195460	1.578689509
C	-3.310056150	-2.094965816	0.681357907
C	-3.467600873	-1.689374183	-0.786505338
C	-2.713526621	-0.381144854	-1.034183830
H	-2.759297162	1.667115766	-0.313111310
H	-4.345649666	0.885851234	-0.371864974
H	-3.551285263	1.098020176	1.982610469
H	-4.965577737	-0.865826999	1.364159128
H	-3.812504081	-1.295419713	2.635797630
H	-3.836336438	-3.043097317	0.861708314
H	-4.532476828	-1.563802755	-1.033942215
H	-3.075376125	-2.481364985	-1.441999336
H	-2.817384477	-0.085285032	-2.087931200
C	-1.226886659	-0.572195778	-0.718140924
H	-0.679082564	0.354765565	-0.928846132
H	-0.798027339	-1.361312983	-1.356728221
C	-1.656252580	0.120888406	1.669538671
H	-1.119034186	1.067016009	1.537366274
H	-1.537216603	-0.176208098	2.722932886
C	-1.826349967	-2.281414731	1.001965359
H	-1.392421212	-3.077651084	0.382293508
H	-1.692106223	-2.597777435	2.046046974
C	-1.054682496	-0.972373492	0.764111797
C	0.411508919	-1.238834066	1.072784978
O	0.858092727	-2.345248193	1.326804210
N	1.305580494	-0.179835425	1.149354797
F	0.909851532	1.013598028	0.540814662
C	2.742257521	-0.368801326	1.003459472
H	3.194835142	0.605119391	1.221802495
H	3.031626492	-1.051058411	1.808505831
C	3.178546770	-0.926911321	-0.345052659
H	2.764564547	-1.938739281	-0.441714835
H	4.272824380	-1.040216006	-0.313673922
C	2.790938283	-0.083253197	-1.555101396
H	3.108663396	-0.618215872	-2.460059626
H	1.695394372	-0.011825561	-1.620628839
C	3.397688941	1.311379146	-1.549390166
H	4.487420132	1.267254260	-1.422539675
H	3.195420265	1.839722615	-2.486939887
H	2.990973068	1.925392845	-0.738039569

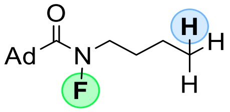

Conformer 6

C	-4.437669335	-0.840826806	1.786217170
C	-3.283608763	-1.683072183	1.238042879
C	-3.523809857	-1.975143743	-0.246884230
C	-3.619836679	-0.658404860	-1.024553347
C	-4.772990687	0.180570746	-0.469837177
C	-4.516443992	0.476400873	1.009958341
H	-4.283074836	-0.640312052	2.857074273
H	-5.385383510	-1.392810349	1.694823836
H	-3.216366474	-2.628797142	1.794592257
H	-4.451942436	-2.553155644	-0.370410332
H	-2.704516351	-2.590986801	-0.647702203
H	-3.793580267	-0.870750031	-2.089143669
H	-5.725562851	-0.357315387	-0.590555854
H	-4.860227401	1.121339579	-1.034074568
H	-5.332344555	1.094181667	1.411282898
C	-3.198154966	1.237628262	1.159599943
H	-3.007467504	1.478461729	2.214047433
H	-3.244696122	2.198793264	0.628091533
C	-1.965645671	-0.919510263	1.399050524
H	-1.785617966	-0.693878223	2.461893490
H	-1.128959455	-1.536042564	1.050114968
C	-2.303541394	0.111318788	-0.882181660
H	-2.358715337	1.062702135	-1.433776209
H	-1.482349972	-0.467960835	-1.318600303
C	-2.029704876	0.404620330	0.606500720

C	-0.751953180	1.207972769	0.812541438
O	-0.712367493	2.254890523	1.434107103
N	0.424792359	0.785265059	0.202086259
F	0.467074695	-0.547073135	-0.203722693
C	1.740541386	1.177114039	0.665298995
H	1.589207542	2.138944429	1.166092102
H	2.086092115	0.453756280	1.421802336
C	2.740515980	1.289296723	-0.470102038
H	2.358060212	2.003033856	-1.214157537
H	2.820020245	0.319008215	-0.981534233
C	4.110480133	1.728039212	0.025937087
H	4.018213868	2.699650846	0.534279363
H	4.465354923	1.020450093	0.790593023
C	5.128713753	1.826843189	-1.098072274
H	4.805230270	2.547409283	-1.859412716
H	6.109893678	2.147574731	-0.732310585
H	5.258947679	0.858693628	-1.597339121

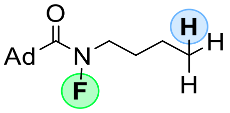

Conformer 7

C	-2.631291930	-2.405676314	-0.415303722
C	-3.010351718	-1.350750460	-1.459909866
C	-4.087608779	-0.427879866	-0.885897891
C	-3.546628168	0.264389406	0.367923082
C	-3.183054065	-0.792006164	1.414952836
C	-2.103909971	-1.717553137	0.848152361
H	-1.866236548	-3.083356027	-0.823240758
H	-3.508854259	-3.021905870	-0.168768202
H	-3.390036938	-1.847767206	-2.364066666
H	-4.990311834	-1.007414295	-0.640146893
H	-4.380536820	0.322169248	-1.635752014
H	-4.310433929	0.941882101	0.775052533
H	-4.074295084	-1.375493736	1.691150117
H	-2.819267275	-0.305608890	2.332403439
H	-1.834699185	-2.476642049	1.596430684
C	-0.856054865	-0.903501783	0.495281286
H	-0.067112857	-1.568697467	0.124452907
H	-0.462306467	-0.397033044	1.391120353
C	-1.770558067	-0.530233734	-1.828453047
H	-1.012700818	-1.178624488	-2.282490324
H	-2.032454287	0.238144233	-2.572083251
C	-2.301143627	1.076519389	0.007810463
H	-1.910513289	1.598702672	0.892056498
H	-2.545398717	1.856549234	-0.726381564
C	-1.208416644	0.158763627	-0.567185249
C	0.002730585	1.024576012	-0.886613668
O	0.036018245	2.232835390	-0.709533300
N	1.160279624	0.417645310	-1.318499283
F	1.128980630	-0.920884910	-1.681667641
C	2.368960193	1.051435229	-1.785334465
H	2.413549231	0.991274349	-2.882206969
H	2.246113641	2.105055563	-1.514565240
C	3.609772437	0.437837932	-1.149397652
H	4.474725934	1.045032072	-1.452848524
H	3.770204103	-0.564218261	-1.568112082
C	3.535472568	0.354632168	0.371096478
H	2.735026087	-0.346078820	0.651432543
H	4.469395592	-0.089640591	0.740643107
C	3.299781233	1.699862830	1.041486173
H	2.311574959	2.111802704	0.800676442
H	3.362104182	1.617675760	2.131645455
H	4.050285625	2.435744089	0.722555202

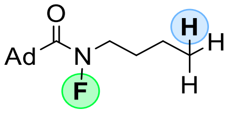

Conformer 8

C	-2.889346628	0.158304006	-2.108226082
C	-3.269074803	0.749174390	-0.748077550
C	-4.053661270	-0.289632674	0.057024498
C	-3.177432029	-1.524119133	0.277509128
C	-2.781629057	-2.118373714	-1.078211589
C	-2.011287667	-1.076820966	-1.896177243
H	-2.350851064	0.906595209	-2.708843000
H	-3.795652684	-0.114153396	-2.669842285
H	-3.885979851	1.647019271	-0.894542538
H	-4.973967060	-0.568567407	-0.478176448
H	-4.359718836	0.133992419	1.025242513
H	-3.730893235	-2.273782379	0.860788010
H	-3.681167562	-2.434928038	-1.626914708
H	-2.162152408	-3.015830499	-0.930673031
H	-1.729666685	-1.505190333	-2.868669238
C	-0.738180654	-0.682456164	-1.142030037
H	-0.178422674	0.075524792	-1.713334933
H	-0.085017968	-1.555305454	-1.024100061
C	-1.999625751	1.136797182	0.012088461
H	-1.434493578	1.898833620	-0.540955134
H	-2.252570225	1.586116653	0.982859200
C	-1.913497828	-1.132892117	1.048910254
H	-1.307685750	-2.022928480	1.248593047
H	-2.183712386	-0.697379294	2.023211221
C	-1.107920859	-0.096675334	0.238800291
C	0.146223573	0.370497897	0.966214791
O	0.394139751	1.546753251	1.177937581
N	1.023598659	-0.567038177	1.491900124
F	0.902755018	-1.871770369	1.020983042
C	2.407006121	-0.296467364	1.837648475
H	2.418384806	0.771049741	2.076454618
H	2.642716402	-0.848782779	2.756003421
C	3.396935349	-0.652771292	0.735340025
H	3.383081278	-1.740214357	0.590861027
H	4.400214447	-0.404782698	1.112190211
C	3.160380083	0.049266684	-0.596019329
H	3.874393144	-0.355937560	-1.325716655

H	2.164343206	-0.223099167	-0.979140878
C	3.298190680	1.563110621	-0.527351933
H	4.266331970	1.848613931	-0.094576275
H	3.239319660	2.012063669	-1.524664752
H	2.507861755	2.014379336	0.083416981

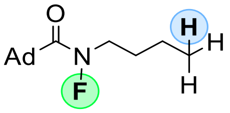

Conformer 9

C	-3.586779857	-0.836758989	1.722493589
C	-2.755380967	-1.715293104	0.785169606
C	-3.571896368	-2.040295826	-0.470262902
C	-3.964063060	-0.742410558	-1.185163660
C	-4.793278764	0.130231749	-0.240810340
C	-3.962803286	0.460575726	1.001739893
H	-3.014870033	-0.610971732	2.634962376
H	-4.495276896	-1.372787453	2.035820724
H	-2.477792288	-2.647799208	1.297384841
H	-4.475066486	-2.604611663	-0.193881623
H	-2.985706806	-2.681106754	-1.146036742
H	-4.551105435	-0.980262676	-2.083794138
H	-5.716236281	-0.394780512	0.048263022
H	-5.096104206	1.056955365	-0.750910533
H	-4.545647691	1.102766337	1.677426356
C	-2.690317602	1.201092717	0.587495443
H	-2.091151196	1.465180003	1.468929129
H	-2.939128485	2.149067077	0.089182428
C	-1.478165800	-0.973221813	0.381117765
H	-0.884795355	-0.719360898	1.274222241
H	-0.854425498	-1.611309143	-0.256250907
C	-2.696596049	0.007937450	-1.607035739
H	-2.963316769	0.947119327	-2.115642168
H	-2.124392848	-0.594866479	-2.319404430
C	-1.847335383	0.331875681	-0.361823830
C	-0.572296469	1.093443924	-0.681929746
O	-0.241071187	2.116209822	-0.109304756
N	0.265515394	0.651689940	-1.712165192
F	0.084908140	-0.683676209	-2.094280202
C	1.694119987	0.912553402	-1.641513412
H	2.110944387	0.710584601	-2.636630697
H	1.772896238	1.987057162	-1.452971853
C	2.394691055	0.098292339	-0.563321346
H	2.144054574	-0.960407775	-0.708543044
H	1.987275717	0.395431646	0.415189264
C	3.907419174	0.282597738	-0.574224620
H	4.349605403	-0.389966236	0.172503622
H	4.306681238	-0.046091260	-1.546124776
C	4.345198493	1.712675896	-0.293228644
H	3.901171622	2.083144028	0.639698778
H	5.433391488	1.787514266	-0.197460554
H	4.043595592	2.399944956	-1.092867912

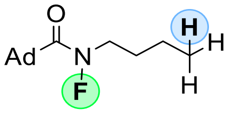

Conformer 10

C	-3.391719822	0.477870490	-1.781914679
C	-3.614602450	0.811065231	-0.304159216
C	-4.171498443	-0.419041346	0.416782159
C	-3.163736199	-1.565538309	0.310853258
C	-2.922946963	-1.900102082	-1.165267057
C	-2.382721911	-0.666957579	-1.896830657
H	-3.020044826	1.363929034	-2.318194761
H	-4.343905812	0.191386150	-2.253331838
H	-4.325070531	1.645426026	-0.216965082
H	-5.132795107	-0.717011603	-0.028455246
H	-4.365288439	-0.182762414	1.473876691
H	-3.553208389	-2.452564644	0.830756840
H	-3.862002685	-2.231403787	-1.633379851
H	-2.208966190	-2.733104777	-1.251821426
H	-2.213611105	-0.909944406	-2.955752934
C	-1.052131430	-0.246004219	-1.266712425
H	-0.657503801	0.650199609	-1.772261303
H	-0.307494555	-1.042364850	-1.384353030
C	-2.286144382	1.221668840	0.332913842
H	-1.880903309	2.116615797	-0.157664900
H	-2.429962400	1.488499092	1.389750872
C	-1.838489890	-1.154378967	0.959794747
H	-1.130347645	-1.988866599	0.926092864
H	-1.996561308	-0.904354729	2.020259976
C	-1.264402399	0.076407694	0.230569758
C	0.045439677	0.567787444	0.824747896
O	0.246947582	1.729161888	1.134538703
N	1.074193019	-0.340121687	1.086509757
F	0.991320553	-1.557786803	0.403244693
C	2.459071692	0.099498069	1.078445072
H	2.477837460	0.995944742	1.706952092
H	3.044327783	-0.679795000	1.579400038
C	2.977897403	0.407216688	-0.319254941
H	2.275040269	1.105768857	-0.795557212
H	2.968042407	-0.515071810	-0.917739079
C	4.377310413	1.010843418	-0.311322011
H	4.647937463	1.283729681	-1.339749345
H	4.360693630	1.954041574	0.255140218
C	5.439430386	0.083702719	0.262564162
H	5.431933407	-0.887438900	-0.249287157
H	6.442828603	0.508322556	0.153112680
H	5.285685000	-0.107157981	1.331580780

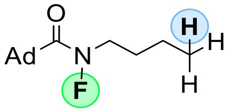

Conformer 11

C	-4.260809412	-0.978501240	-1.309449997

C	-4.167778180	-0.167699336	-0.014128112
C	-4.217486125	-1.117968891	1.185007339
C	-3.032907064	-2.083677465	1.113605117
C	-3.108880796	-2.892836920	-0.185363303
C	-3.076639177	-1.944319849	-1.388427373
H	-4.256631410	-0.304372853	-2.179161331
H	-5.207882227	-1.538327236	-1.339792008
H	-5.006327942	0.541054795	0.039784257
H	-5.164489881	-1.678747744	1.186821487
H	-4.180294055	-0.544899319	2.123740972
H	-3.057370472	-2.766182896	1.975196127
H	-4.032878332	-3.490150720	-0.202770303
H	-2.267087029	-3.599724183	-0.239221503
H	-3.132818288	-2.526336623	-2.319484406
C	-1.764577557	-1.154196643	-1.377409291
H	-1.732361150	-0.461310735	-2.232879114
H	-0.913757888	-1.837965923	-1.479441424
C	-2.853396015	0.615431063	0.005597861
H	-2.809155070	1.319244407	-0.836417433
H	-2.779371278	1.223101835	0.918652429
C	-1.720073421	-1.295704151	1.144330166
H	-0.869729670	-1.986365147	1.139555081
H	-1.653518484	-0.705535354	2.071537677
C	-1.653895872	-0.344131776	-0.067113825
C	-0.385726630	0.500588697	-0.069456146
O	-0.389935378	1.716455767	-0.144705406
N	0.845636845	-0.121335284	0.103713212
F	0.893245931	-1.488945757	-0.152730230
C	2.102980060	0.479400432	-0.296138356
H	2.303764432	0.214113289	-1.345583807
H	1.929244485	1.559753937	-0.247904752
C	3.242723722	0.055877158	0.614623461
H	3.397649987	-1.028198860	0.513506185
H	2.934171923	0.232434753	1.653710229
C	4.542351348	0.795405864	0.318577871
H	4.387984042	1.874814259	0.466468257
H	5.292162034	0.493768758	1.061426845
C	5.086383786	0.542145602	-1.081032207
H	4.433490881	0.958065471	-1.857728239
H	6.074027688	0.996029732	-1.213876422
H	5.185899366	-0.533758006	-1.274884697

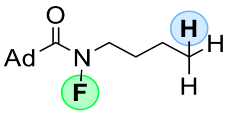

Conformer 12

C	-4.796659177	-0.040523504	-0.037827968
C	-4.181227775	-0.914855110	-1.133266881
C	-4.193709250	-0.150887142	-2.460109922
C	-3.364130559	1.126961150	-2.316576099
C	-3.963263010	2.004841395	-1.212774294
C	-3.968496462	1.237228483	0.113308874
H	-4.820149536	-0.589354165	0.915656634
H	-5.837799269	0.210018416	-0.291597444
H	-4.760890648	-1.843105225	-1.235608817
H	-5.227084895	0.098963578	-2.744362453
H	-3.781381583	-0.780084358	-3.263113744
H	-3.363056875	1.678564918	-3.267549190
H	-4.989191738	2.297883878	-1.481533503
H	-3.379705419	2.932173285	-1.109473675
H	-4.400115862	1.868562113	0.903079753
C	-2.529944177	0.879269943	0.496979581
H	-2.517944336	0.323109562	1.447163116
H	-1.945763090	1.793026931	0.650392876
C	-2.740030011	-1.267719620	-0.759814569
H	-2.712801992	-1.837908678	0.179623300
H	-2.288924299	-1.913803200	-1.524918286
C	-1.921029047	0.766197691	-1.952282131
H	-1.310005563	1.674448615	-1.893074202
H	-1.481796780	0.126098018	-2.733470878
C	-1.895718949	0.008682979	-0.606541734
C	-0.472922988	-0.417118649	-0.263596245
O	-0.103173924	-1.577804832	-0.262264633
N	0.445443369	0.542899040	0.147807033
F	0.142158061	1.865960205	-0.168651181
C	1.879976685	0.356659896	0.077879656
H	2.023024409	-0.727748188	0.116058033
H	2.241713604	0.712093622	-0.901474697
C	2.598889526	1.073214416	1.208287989
H	2.280534979	0.633354317	2.164617150
H	2.272738803	2.121516734	1.219871025
C	4.115065582	1.003301617	1.072835973
H	4.417863475	1.484865069	0.130496141
H	4.567757908	1.601760092	1.874018327
C	4.664800713	-0.415411737	1.123287027
H	4.344189612	-1.011467353	0.260342394
H	5.759886736	-0.420534272	1.129751800
H	4.324267881	-0.936620496	2.027210398

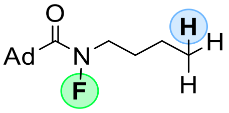

Conformer 13

C	-3.604090486	-0.753067254	-0.527221395
C	-3.649108093	0.426834820	0.447721978
C	-3.667907709	-0.100808737	1.885076304
C	-2.396806970	-0.912997657	2.142310575
C	-2.334561978	-2.089676176	1.163133846
C	-2.332633541	-1.567603736	-0.277488467
H	-3.621017247	-0.388124485	-1.565225015
H	-4.492673834	-1.388723067	-0.394456535
H	-4.551283425	1.027052991	0.261793183
H	-4.557034276	-0.728832870	2.047364481
H	-3.731129767	0.736998565	2.595854398
H	-2.398630887	-1.292003418	3.174547974
H	-3.197042073	-2.754741213	1.320530441
H	-1.429449247	-2.687945413	1.348899962

H	-2.289740250	-2.415218035	-0.976635233
C	-1.099815659	-0.685267078	-0.493210679
H	-1.082518242	-0.294005451	-1.523253864
H	-0.185980665	-1.275661595	-0.356129201
C	-2.415716595	1.308074524	0.241384951
H	-2.396686248	1.714453964	-0.779279633
H	-2.440889053	2.174559920	0.916253835
C	-1.165530736	-0.023772749	1.944266436
H	-0.254184646	-0.588315684	2.171855978
H	-1.201589911	0.832403183	2.636195653
C	-1.130335490	0.500632871	0.493201719
C	0.053740601	1.429275450	0.263076793
O	-0.062191635	2.611091291	-0.025531127
N	1.332873696	0.901487730	0.269032464
F	1.516306933	-0.364976431	0.808090319
C	2.562532903	1.659954950	0.373100578
H	2.263965534	2.703914726	0.229469147
H	2.937899048	1.547581275	1.399999827
C	3.601986524	1.218386783	-0.647943687
H	3.625026523	0.118144577	-0.661760533
H	4.588086703	1.539635928	-0.287471879
C	3.380991596	1.760588905	-2.056904913
H	3.463636201	2.858034199	-2.031700246
H	4.208417575	1.413521706	-2.690607627
C	2.055764984	1.369317657	-2.695941579
H	1.208713559	1.868486606	-2.210302521
H	2.036495592	1.649918178	-3.754792970
H	1.885744812	0.286439884	-2.631226360

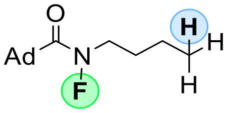

Conformer 14

C	-2.978356047	-1.652876167	-0.373838266
C	-2.633276558	-1.178263767	-1.787983287
C	-3.324643424	0.160843903	-2.058459199
C	-2.832880235	1.199054800	-1.047451763
C	-3.165203427	0.726974703	0.372125115
C	-2.485781020	-0.619860355	0.642058467
H	-2.510020620	-2.629031824	-0.177515432
H	-4.065530963	-1.791458158	-0.274362884
H	-2.971604919	-1.923957101	-2.521456431
H	-4.416511773	0.046677750	-1.982035644
H	-3.107094433	0.498671869	-3.082788479
H	-3.321886350	2.165077823	-1.238799691
H	-4.254756779	0.628029793	0.489492317
H	-2.828907885	1.473661984	1.107292216
H	-2.725368648	-0.956489438	1.660964485
C	-0.967875644	-0.455292955	0.518357898
H	-0.463672484	-1.415733247	0.709627138
H	-0.607855589	0.256525088	1.271632048
C	-1.118227900	-1.006875682	-1.913944081
H	-0.603876100	-1.962812382	-1.744199660
H	-0.846308808	-0.688583370	-2.929777681
C	-1.317177550	1.374238282	-1.183661575
H	-0.964548441	2.146618945	-0.490251418
H	-1.064158089	1.708994155	-2.201634113
C	-0.608859490	0.032335493	-0.901193369
C	0.898625523	0.149188719	-1.078750727
O	1.543553383	-0.491724466	-1.896673403
N	1.613152714	1.011325888	-0.287270021
F	0.968639266	1.789278405	0.659493234
C	3.041570555	1.252964221	-0.362431763
H	3.287761345	1.349047274	-1.426636741
H	3.200755787	2.229564607	0.111051376
C	3.908350825	0.173487321	0.271731803
H	4.939281205	0.361983464	-0.058928808
H	3.620206366	-0.793617330	-0.164329448
C	3.875598227	0.103655252	1.794497737
H	4.685247456	-0.563595270	2.120386815
H	4.121292537	1.093806517	2.209669129
C	2.559586593	-0.388773917	2.380214662
H	2.254442120	-1.335037254	1.913497353
H	2.646970432	-0.562647258	3.458248437
H	1.750289132	0.332085478	2.226988559

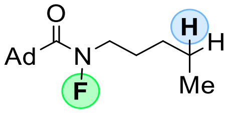

Conformer 1

C	-2.250419811	1.465281809	-2.303428477
C	-2.359579870	0.478884938	-1.135867195
C	-2.865464144	-0.869636262	-1.652308000
C	-1.885638354	-1.412679353	-2.695904965
C	-1.791104072	-0.430994318	-3.866704792
C	-1.280611061	0.918085208	-3.355966519
H	-1.900862035	2.443392650	-1.940006755
H	-3.241826235	1.624672622	-2.753255920
H	-3.055731353	0.874110228	-0.382205092
H	-3.865979255	-0.752802245	-2.095374154
H	-2.963103760	-1.581150800	-0.818665360
H	-2.236174005	-2.389255062	-3.058993520
H	-2.777182486	-0.308322519	-4.339702029
H	-1.111137663	-0.825168545	-4.636825174
H	-1.205325911	1.627994300	-4.192081402
C	0.105683458	0.744230042	-2.728183859
H	0.493073290	1.714697585	-2.398227163
H	0.812666171	0.348172703	-3.473397959
C	-0.980539158	0.303955231	-0.492978687
H	-0.631481036	1.263903316	-0.092403210
H	-1.036819195	-0.404644565	0.349171721
C	-0.506212307	-1.584859315	-2.058349844
H	0.207299204	-1.998619902	-2.785084960
H	-0.549380716	-2.302262876	-1.227885525
C	0.019562474	-0.235211474	-1.540503200
C	1.372902594	-0.484316200	-0.892054475
O	1.837686490	-1.596528419	-0.706017233

N	2.182676894	0.592081737	-0.550489402
F	1.545354576	1.826672621	-0.410003221
C	3.243927657	0.468634435	0.437748756
H	3.919632271	-0.298270378	0.047245495
H	3.778250462	1.426192815	0.418085750
C	2.777248738	0.109724134	1.839967512
H	3.674191761	0.000972907	2.468694531
H	2.305615688	-0.882921661	1.808414172
C	1.831569294	1.117190734	2.478437768
H	2.272382095	2.126682984	2.431223375
H	0.897396215	1.175573960	1.899207650
C	1.506583732	0.767894361	3.923849743
H	1.077868041	-0.245168293	3.961864970
C	0.551043900	1.760058891	4.565844516
H	-0.398270931	1.804135730	4.017210591
H	0.322435414	1.496833802	5.604178005
H	0.975084959	2.772237189	4.564899554
H	2.441035514	0.718228656	4.503625058

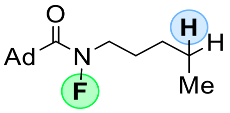

Conformer 2

C	-3.161604475	-1.402177127	0.662450860
C	-2.013016592	-1.832119275	-0.252960418
C	-2.513462312	-1.909245485	-1.699188110
C	-3.040458798	-0.538960834	-2.138010075
C	-4.186014649	-0.112778930	-1.217559594
C	-3.673032975	-0.027924387	0.222271367
H	-2.817874445	-1.361319925	1.706982288
H	-3.975428470	-2.141694146	0.619692112
H	-1.635245116	-2.815828931	0.060659860
H	-3.311154659	-2.662727777	-1.780026758
H	-1.698048489	-2.230478689	-2.364766379
H	-3.399490715	-0.597414251	-3.175473198
H	-5.014139700	-0.834505987	-1.284137069
H	-4.582704881	0.862953553	-1.536026786
H	-4.486934636	0.294266107	0.887206706
C	-2.534749601	0.990626130	0.301552285
H	-2.167164345	1.082469077	1.332470144
H	-2.889648361	1.989739292	0.011915296
C	-0.872391038	-0.813411741	-0.163822661
H	-0.509472336	-0.742162408	0.874749552
H	-0.028694582	-1.138699899	-0.785016948
C	-1.906610522	0.487805634	-2.064282472
H	-2.271043049	1.479365288	-2.374305172
H	-1.102634715	0.208250694	-2.753858245
C	-1.374100120	0.574957806	-0.619172749
C	-0.274703243	1.620265492	-0.475192228
O	-0.317043901	2.527589778	0.337708487
N	0.794159904	1.610717988	-1.365004804
F	1.016280337	0.417838086	-2.052107102
C	2.064418511	2.257368676	-1.094110731

H	2.466340801	2.623992997	-2.046377616
H	1.797250634	3.124016148	-0.480949605
C	3.070462702	1.364463898	-0.384285321
H	3.973260172	1.966059676	-0.203486488
H	3.375604238	0.551094222	-1.057624503
C	2.564361769	0.781132190	0.924856107
H	2.194738254	1.586932776	1.579122042
H	1.693851524	0.134917752	0.725665844
C	3.619074979	-0.039840139	1.652575021
H	3.975515391	-0.837393111	0.981946943
C	3.100883839	-0.632583656	2.950607976
H	2.221141651	-1.264862753	2.769703305
H	3.853641438	-1.246801676	3.455469948
H	2.791718000	0.155492295	3.648509291
H	4.505941092	0.586214060	1.850401072

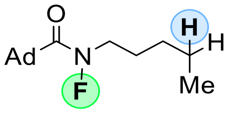

Conformer 3

C	-3.774530880	0.731824921	-2.194574100
C	-2.774586234	1.829006190	-1.823402917
C	-2.975001467	2.229257491	-0.357858409
C	-2.764751756	1.009435155	0.545610088
C	-3.764670718	-0.085307148	0.167132100
C	-3.548294335	-0.485790459	-1.294593927
H	-3.650833385	0.449705308	-3.250931429
H	-4.803828189	1.103649103	-2.078794089
H	-2.927244742	2.705502492	-2.469432765
H	-3.988536916	2.632763494	-0.214363699
H	-2.268664579	3.027642040	-0.084290372
H	-2.910636074	1.299215229	1.596106258
H	-4.794018264	0.275329738	0.313857833
H	-3.633165694	-0.959437326	0.822439356
H	-4.252954756	-1.283954367	-1.568089492
C	-2.118961844	-0.999155866	-1.478410673
H	-1.949826308	-1.313532334	-2.517252425
H	-1.943871428	-1.888038782	-0.856522495
C	-1.345733021	1.312976408	-2.018435309
H	-1.191037346	1.013373607	-3.066666155
H	-0.625152721	2.108632505	-1.796733092
C	-1.335336287	0.488798792	0.369053630
H	-1.168488493	-0.392186315	1.008906052
H	-0.616313456	1.255929227	0.679561711
C	-1.102616099	0.093219935	-1.104047111
C	0.292007014	-0.482601425	-1.313781882
O	0.501747202	-1.594100800	-1.771323740
N	1.386270206	0.250154024	-0.898206123
F	1.210781405	1.591145317	-0.586444163
C	2.771923370	-0.024540145	-1.197994891
H	2.775964665	-1.073983164	-1.510800230
H	3.089680985	0.587289548	-2.055045175
C	3.667322762	0.206990325	0.008526343
H	3.678081801	1.278983604	0.253608113

H	4.696568095	-0.050467511	-0.280436206
C	3.252607404	-0.594244279	1.232664708
H	2.240554438	-0.288596698	1.543438042
H	3.173270282	-1.661621240	0.968303585
C	4.210463659	-0.421834650	2.402278069
H	4.284885856	0.648753862	2.650268492
C	3.789022874	-1.215916241	3.626795715
H	2.791190975	-0.913880479	3.970014294
H	3.745378160	-2.289929263	3.404598854
H	4.480824884	-1.078882478	4.464410094
H	5.225945917	-0.718355731	2.091508269

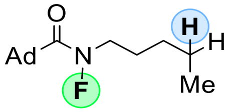

Conformer 4

C	-3.360816833	-2.026703048	-0.441708887
C	-2.583273940	-1.138986264	-1.419248421
C	-3.369214449	0.148616328	-1.676992263
C	-3.571653208	0.892968113	-0.354297603
C	-4.364296584	0.008186127	0.611061253
C	-3.579576013	-1.278075342	0.877672212
H	-2.808197014	-2.960582516	-0.258701459
H	-4.330424144	-2.306592997	-0.880062085
H	-2.429726746	-1.677356650	-2.365514761
H	-4.343116963	-0.087388338	-2.131681565
H	-2.825969797	0.785884550	-2.390815725
H	-4.121091834	1.827484946	-0.536713597
H	-5.350139860	-0.230604798	0.184259381
H	-4.543070009	0.544047436	1.555335750
H	-4.139400588	-1.917457274	1.575100708
C	-2.221620027	-0.938152241	1.500091776
H	-1.676606866	-1.858264248	1.734817884
H	-2.363647548	-0.393697492	2.446268241
C	-1.217631354	-0.796919559	-0.816896113
H	-0.628883593	-1.710101617	-0.668721755
H	-0.649623901	-0.147209613	-1.502115655
C	-2.210004169	1.228210288	0.256259242
H	-2.333699082	1.785383732	1.196036482
H	-1.634563362	1.880202389	-0.414252001
C	-1.411604168	-0.058269009	0.527606933
C	-0.050225914	0.343530865	1.069818182
O	0.356520425	1.491717491	1.078543886
N	0.787727834	-0.621141140	1.638025116
F	0.490483319	-1.945688438	1.292962118
C	2.230972876	-0.453624612	1.569334708
H	2.430051866	0.541851039	1.978342901
H	2.670712064	-1.192041030	2.250890831
C	2.784085781	-0.590940937	0.160623199
H	2.318184298	0.175452096	-0.477172398
H	2.488936174	-1.568744532	-0.247412810
C	4.296818986	-0.445609374	0.119670758
H	4.585066121	0.534819761	0.532877877
H	4.760005702	-1.197253672	0.779969093
C	4.866487965	-0.590469304	-1.283878585
H	4.585525561	-1.575522373	-1.686456516
C	6.377100669	-0.423836785	-1.322682994
H	6.872868949	-1.169466742	-0.688214315
H	6.776193150	-0.533702263	-2.336690050
H	6.673351978	0.566613896	-0.954546281
H	4.389348041	0.150125934	-1.942639394

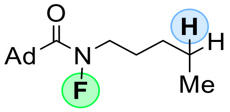

Conformer 5

C	-3.295298729	0.679183144	-0.169928935
C	-3.147208318	0.310422040	1.310103116
C	-3.899452870	-0.992941993	1.587999545
C	-3.314754985	-2.110047652	0.719949491
C	-3.474503573	-1.745050208	-0.758196276
C	-2.722800705	-0.442946883	-1.042632941
H	-2.771476886	1.624282873	-0.378128289
H	-4.356492581	0.838738955	-0.412884560
H	-3.558475608	1.117036547	1.933766487
H	-4.970850280	-0.865115084	1.371552880
H	-3.815430190	-1.258160617	2.652627851
H	-3.839934715	-3.053347573	0.926809474
H	-4.539927190	-1.627942644	-1.007464369
H	-3.082260681	-2.554399196	-1.392102585
H	-2.828691281	-0.176169535	-2.103885621
C	-1.235394106	-0.622283844	-0.723536001
H	-0.689850752	0.300304242	-0.958755616
H	-0.804925771	-1.426831496	-1.341365824
C	-1.662613504	0.134346283	1.644967442
H	-1.126971198	1.077164833	1.486029380
H	-1.542047353	-0.133383758	2.705984131
C	-1.830514306	-2.286258075	1.043129245
H	-1.396892852	-3.098390847	0.444350051
H	-1.694944695	-2.574159986	2.095290640
C	-1.060909031	-0.982628440	0.768886835
C	0.406184324	-1.240872436	1.079227880
O	0.854080167	-2.343236538	1.346885701
N	1.298659471	-0.176784135	1.144484168
F	0.901606013	1.002432300	0.507861977
C	2.731912261	-0.372681837	0.983067675
H	3.196076170	0.591768785	1.218350963
H	3.018227825	-1.075084425	1.771521038
C	3.154587985	-0.907556225	-0.378968736
H	2.711822238	-1.904405404	-0.502285825
H	4.244751879	-1.055180374	-0.347189906
C	2.799079025	-0.023048663	-1.568676718
H	3.114449440	-0.538021841	-2.488530237
H	1.705625200	0.081049436	-1.646988132
C	3.434676265	1.361039486	-1.534257690
H	3.036905453	1.929466218	-0.682288683
C	3.188802732	2.139117572	-2.817498095
H	3.616487154	1.618791553	-3.683906031

H	2.113941158	2.255626173	-3.005110461
H	3.628745557	3.141582260	-2.781268298
H	4.516761735	1.258081720	-1.357920173

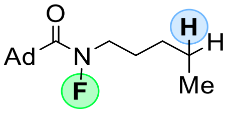

Conformer 6

C	-4.429542304	-0.846451585	1.783099943
C	-3.258108059	-1.675882007	1.253332897
C	-3.487307187	-1.996045467	-0.227434670
C	-3.605605490	-0.693586952	-1.026002263
C	-4.776486812	0.132058827	-0.489272910
C	-4.531098288	0.456565416	0.986239262
H	-4.283870243	-0.626490564	2.851323961
H	-5.366307166	-1.417378331	1.696646304
H	-3.175765022	-2.611288442	1.825237596
H	-4.403736038	-2.593616920	-0.344410954
H	-2.655185130	-2.602459849	-0.615897417
H	-3.770250561	-0.926018589	-2.087847380
H	-5.718086331	-0.425924374	-0.603787595
H	-4.879955446	1.061502093	-1.069113863
H	-5.360724639	1.063916806	1.375210824
C	-3.228446766	1.245166891	1.129610635
H	-3.047060288	1.505974873	2.180831136
H	-3.290748368	2.196820900	0.582819117
C	-1.955424489	-0.885390351	1.407832083
H	-1.784692133	-0.639229935	2.467574157
H	-1.105120049	-1.489486587	1.070785411
C	-2.304799223	0.103048234	-0.890768262
H	-2.375639572	1.044562141	-1.457090609
H	-1.471304348	-0.467088701	-1.314652120
C	-2.041650811	0.425627409	0.594027449
C	-0.779387238	1.252517696	0.796960895
O	-0.755401699	2.294138477	1.427326374
N	0.402106868	0.862805665	0.163571869
F	0.468517165	-0.481393613	-0.211772979
C	1.709357934	1.269093324	0.643787018
H	1.553460951	2.255641519	1.091909595
H	2.030992551	0.583889128	1.445109218
C	2.731048907	1.314103764	-0.475275938
H	2.381856747	2.012160996	-1.249849468
H	2.789338788	0.325068942	-0.951808544
C	4.105679527	1.727354616	0.025504596
H	4.043326072	2.716489574	0.507963581
H	4.437937645	1.031448810	0.813616795
C	5.148140006	1.769807114	-1.082154018
H	5.203875445	0.780667786	-1.561256596
C	6.520830707	2.181198431	-0.578448262
H	6.883965094	1.485313479	0.188446477
H	7.262796388	2.204678532	-1.383499279
H	6.491018155	3.179742608	-0.124543356
H	4.812006645	2.463566223	-1.867495147

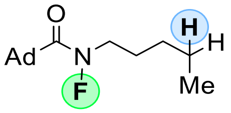

Conformer 7

C	-2.602999748	-2.351113441	-0.438039256
C	-3.034974090	-1.191564669	-1.341708185
C	-3.980811896	-0.267540717	-0.571893113
C	-3.257537191	0.284075056	0.659267632
C	-2.841348493	-0.876061676	1.566816643
C	-1.892250648	-1.801795980	0.803168604
H	-1.933409362	-3.029283336	-0.988326302
H	-3.481652842	-2.940686089	-0.136395689
H	-3.545135205	-1.588192993	-2.231179598
H	-4.883457784	-0.818371092	-0.267025294
H	-4.311010959	0.559020433	-1.218887479
H	-3.925721416	0.962118433	1.209044733
H	-3.728892944	-1.435652997	1.898792875
H	-2.347265770	-0.490709106	2.471437964
H	-1.582661430	-2.634314747	1.451060328
C	-0.646328528	-1.021472091	0.373099489
H	0.060090310	-1.689786068	-0.133873993
H	-0.134233185	-0.613165959	1.259777748
C	-1.796767897	-0.409513423	-1.790070376
H	-1.137141608	-1.057418643	-2.377694162
H	-2.092188572	0.429174838	-2.439245430
C	-2.015112712	1.060269347	0.219363982
H	-1.493949385	1.482147022	1.089819753
H	-2.296582796	1.913773274	-0.413257837
C	-1.052787062	0.143990014	-0.557043199
C	0.161542480	0.978785594	-0.948604654
O	0.326256824	2.128998837	-0.576634860
N	1.088827885	0.460758643	-1.846937772
F	1.073057114	-0.923132644	-2.015423509
C	2.454092748	0.942770053	-1.957684105
H	2.768710769	0.810466139	-2.999699795
H	2.378271794	2.017302630	-1.767169681
C	3.433875389	0.255168105	-1.010602371
H	4.420716134	0.713025052	-1.173501923
H	3.528933468	-0.796125366	-1.309806634
C	3.055711236	0.342761348	0.461543764
H	2.044113716	-0.073639058	0.601915301
H	3.717263514	-0.317342803	1.041495881
C	3.108835162	1.746737232	1.050187550
H	2.550554406	2.442548305	0.410317427
C	2.513343641	1.790490086	2.447120910
H	1.444453061	1.538292186	2.409584619
H	3.002083807	1.070424613	3.116639278
H	2.600635312	2.783049876	2.901199462
H	4.153316285	2.093792361	1.063606250

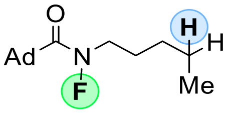

Conformer 8

C	-2.814341560	0.090902527	-2.173983573
C	-3.178812606	0.771912309	-0.852327202
C	-4.013280339	-0.186664593	0.001080835
C	-3.193903274	-1.444781166	0.295922991
C	-2.813415398	-2.128710900	-1.021464195
C	-1.991321894	-1.166322851	-1.885184445
H	-2.239833306	0.781114622	-2.809869689
H	-3.727032443	-0.174169541	-2.728693204
H	-3.755275806	1.685852569	-1.053964851
H	-4.941639800	-0.454003428	-0.525894693
H	-4.306487299	0.302031801	0.942362352
H	-3.784351449	-2.136884936	0.913287114
H	-3.721233952	-2.435280316	-1.562189536
H	-2.234966116	-3.042744401	-0.818966938
H	-1.718870302	-1.658333401	-2.829825394
C	-0.708702315	-0.783897107	-1.140052932
H	-0.113738253	-0.083292526	-1.748656868
H	-0.092289871	-1.675136104	-0.970945917
C	-1.900681549	1.147112585	-0.100600267
H	-1.297821039	1.852162775	-0.689367751
H	-2.142322245	1.660925522	0.840414659
C	-1.920120297	-1.067178585	1.057933986
H	-1.351743728	-1.969657057	1.308094389
H	-2.178129048	-0.571319836	2.006454828
C	-1.064989737	-0.108632506	0.203315913
C	0.193946350	0.343346749	0.934501344
O	0.473849780	1.517597075	1.115959135
N	1.031237207	-0.606264965	1.504181968
F	0.885557796	-1.915755481	1.052506602
C	2.418616360	-0.363768753	1.851671592
H	2.443238965	0.687084219	2.154726066
H	2.652106656	-0.971096302	2.734357067
C	3.397866243	-0.661771407	0.719919350
H	3.388527371	-1.741624018	0.525449550
H	4.406116491	-0.422423430	1.088371678
C	3.118544410	0.099847779	-0.568606384
H	3.761476678	-0.299123991	-1.366867470
H	2.087932891	-0.112750329	-0.898793014
C	3.316818470	1.607521538	-0.472740920
H	2.775126578	2.002993438	0.396356442
C	2.815896929	2.319274190	-1.719184598
H	3.288853069	1.924398047	-2.627874817
H	1.730689189	2.181323910	-1.821194259
H	3.007824137	3.396723530	-1.682825409
H	4.383789059	1.820927748	-0.308136102

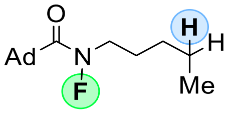

Conformer 9

C	-3.605321759	-0.811096224	1.724673437
C	-2.776711685	-1.704784940	0.799070177
C	-3.589091591	-2.032021399	-0.458389466
C	-3.964147902	-0.736249350	-1.185892719
C	-4.789894035	0.152707356	-0.253483401
C	-3.963668003	0.484558481	0.991657095
H	-3.036562236	-0.584165680	2.638867240
H	-4.521058184	-1.335741514	2.036404000
H	-2.511318060	-2.635761005	1.320273975
H	-4.499458045	-2.585148521	-0.182736467
H	-3.005419976	-2.683971905	-1.125710797
H	-4.548883976	-0.975415651	-2.085602881
H	-5.720136558	-0.360225510	0.034042696
H	-5.079498093	1.078388440	-0.773135302
H	-4.544244019	1.137854418	1.658485928
C	-2.681298062	1.208928603	0.578939237
H	-2.084252101	1.473359341	1.461854055
H	-2.917517155	2.155685769	0.072358998
C	-1.490162408	-0.978193718	0.396334376
H	-0.898998440	-0.723742942	1.290858222
H	-0.869225407	-1.627873296	-0.231808771
C	-2.687009926	-0.002114322	-1.606342184
H	-2.940922124	0.935003615	-2.125223969
H	-2.116006818	-0.617066471	-2.309871814
C	-1.842191714	0.324265478	-0.358796798
C	-0.561426866	1.074578657	-0.684408688
O	-0.225216770	2.102857726	-0.124317106
N	0.272071846	0.618796953	-1.710263687
F	0.086578741	-0.718507021	-2.080281369
C	1.700864562	0.879283951	-1.648136439
H	2.115994029	0.645568400	-2.636901021
H	1.784976458	1.958607380	-1.492692200
C	2.400564551	0.095373724	-0.547099940
H	2.155877313	-0.968310304	-0.666876340
H	1.986586828	0.414286374	0.421509121
C	3.910658363	0.289708086	-0.553895342
H	4.357627067	-0.350911032	0.220683666
H	4.326979395	-0.066290838	-1.510985377
C	4.353771555	1.730103612	-0.322403843
H	4.013304837	2.365393695	-1.153827402
C	5.860775677	1.853417234	-0.172540545
H	6.219464572	1.269339889	0.684534193
H	6.379019624	1.472544160	-1.062038477
H	6.177539677	2.891400877	-0.024833223
H	3.848411740	2.126363140	0.573611870

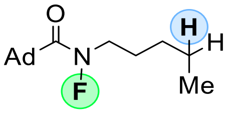

Conformer 10

C	-3.373205223	0.472943624	-1.787945356
C	-3.619929896	0.786308994	-0.309823496
C	-4.166741553	-0.460907728	0.388936775
C	-3.140216609	-1.590516544	0.282096938
C	-2.875734933	-1.904986399	-1.194379508
C	-2.345542678	-0.654713914	-1.904213705
H	-3.008916380	1.370634564	-2.309694802
H	-4.314931085	0.177623010	-2.274721562
H	-4.344795564	1.608116099	-0.222763908
H	-5.117822402	-0.767725366	-0.072013747
H	-4.377161000	-0.240887809	1.446303349
H	-3.522473677	-2.489103753	0.787129370
H	-3.803552938	-2.245096119	-1.678288046
H	-2.147666149	-2.725660489	-1.280803266
H	-2.160091089	-0.881754012	-2.963891489
C	-1.029845509	-0.219812966	-1.252866073
H	-0.642817211	0.687870640	-1.743534714
H	-0.271415414	-1.002966956	-1.369895661
C	-2.305820594	1.210452331	0.348278508
H	-1.909047637	2.117147321	-0.127524469
H	-2.466359674	1.462799069	1.406253074
C	-1.830086485	-1.165975039	0.952572582
H	-1.108503995	-1.988623016	0.918638111
H	-2.005749188	-0.930652580	2.013535650
C	-1.265600316	0.082243551	0.245217083
C	0.030286959	0.583410314	0.860334288
O	0.215790936	1.741720308	1.189190163
N	1.067517030	-0.315400782	1.125592104
F	1.009569823	-1.521432229	0.415508110
C	2.447471607	0.143201932	1.115711343
H	2.477244346	1.001216477	1.795123358
H	3.044759015	-0.660724084	1.560355578
C	2.938508779	0.528003595	-0.272293788
H	2.258547509	1.291856272	-0.674718576
H	2.864105322	-0.348676272	-0.932298241
C	4.366313027	1.058264037	-0.267673021
H	4.610177189	1.437505878	-1.271026649
H	4.433590994	1.928741515	0.404976274
C	5.415472483	0.029072276	0.135641374
H	5.236563509	-0.312283096	1.165796308
C	6.828327583	0.580315269	0.029148238
H	6.965894067	1.438584352	0.698534021
H	7.581879238	-0.169372185	0.292205060
H	7.042323110	0.924913882	-0.990422347
H	5.308885590	-0.862463105	-0.501198530

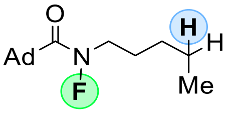

Conformer 11

C	-4.207833733	-1.013147577	-1.376061706
C	-4.173160114	-0.204688771	-0.076300227
C	-4.243929747	-1.159145200	1.118329642
C	-3.039341176	-2.101904233	1.083964367
C	-3.057757842	-2.908806612	-0.218667154
C	-3.003878949	-1.956612237	-1.418010484
H	-4.187796683	-0.336533794	-2.243554268
H	-5.142683148	-1.590327721	-1.438752467
H	-5.026835579	0.487341446	-0.048586861
H	-5.179372340	-1.738030618	1.086817832
H	-4.249573395	-0.588273176	2.059007729
H	-3.079224653	-2.787358649	1.942612242
H	-3.969533106	-3.522714261	-0.267775158
H	-2.201809458	-3.599928510	-0.246596420
H	-3.018231423	-2.537016005	-2.351699965
C	-1.707987221	-1.142134819	-1.362220648
H	-1.661255366	-0.445213527	-2.213682220
H	-0.840621059	-1.808388767	-1.436531801
C	-2.876046577	0.603456258	-0.011534969
H	-2.817526501	1.310152612	-0.850093818
H	-2.843950375	1.210110830	0.904673618
C	-1.742771822	-1.290032565	1.159785473
H	-0.879726973	-1.964063799	1.179826471
H	-1.717539909	-0.701277436	2.089749449
C	-1.655924836	-0.333253603	-0.046465938
C	-0.404560721	0.533401431	-0.015046395
O	-0.428282864	1.747975598	-0.104538986
N	0.835431583	-0.064965453	0.207041256
F	0.901991752	-1.434389843	-0.059336765
C	2.080091591	0.544746284	-0.223388155
H	2.256301276	0.278445882	-1.276973321
H	1.900944904	1.624108568	-0.175543746
C	3.242261332	0.131157545	0.661604124
H	3.365472791	-0.959903939	0.603680608
H	2.979826933	0.361093326	1.702717809
C	4.546825970	0.819394885	0.280499226
H	4.422553412	1.912577954	0.348469609
H	5.314874073	0.560446991	1.023975411
C	5.064500696	0.458691162	-1.107492505
H	4.359187873	0.804673091	-1.877346928
C	6.435599509	1.055106793	-1.380946477
H	7.174262927	0.695696356	-0.653551643
H	6.802049288	0.797803304	-2.380550313
H	6.409209190	2.149661663	-1.308181833
H	5.103128320	-0.636644438	-1.201873799

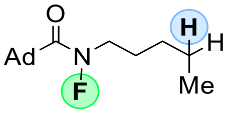

Conformer 12

C	-4.796777623	-0.054549145	-0.056712807
C	-4.173278322	-0.915405267	-1.158177324
C	-4.178617510	-0.138225201	-2.477153236
C	-3.353799891	1.140425531	-2.314784465
C	-3.963304749	2.004674386	-1.205878365
C	-3.973240852	1.223839318	0.112659197
H	-4.824545058	-0.614109851	0.890397661
H	-5.837111603	0.196093502	-0.313567662
H	-4.749962723	-1.844145739	-1.273817857
H	-5.210642218	0.111693555	-2.766059505
H	-3.759426091	-0.758705269	-3.283438214
H	-3.347884913	1.702235860	-3.259880591
H	-4.988923401	2.295523574	-1.478154059
H	-3.384780199	2.933597887	-1.089920579
H	-4.411578176	1.846051733	0.906083689
C	-2.535511959	0.867199289	0.502187547
H	-2.527055396	0.301939408	1.446940400
H	-1.954374631	1.781280959	0.666342412
C	-2.734231715	-1.268260406	-0.779667947
H	-2.711008696	-1.848947875	0.153392850
H	-2.276078800	-1.904896822	-1.548440199
C	-1.911562954	0.781466335	-1.944313956
H	-1.304002922	1.690752727	-1.869372660
H	-1.464607744	0.151002617	-2.728934127
C	-1.893635152	0.008526732	-0.606511466
C	-0.473442500	-0.417417892	-0.261081660
O	-0.098132801	-1.576352249	-0.281674362
N	0.436302585	0.539138340	0.183719850
F	0.127362820	1.865426951	-0.119412138
C	1.871489893	0.359279053	0.099652186
H	2.016404559	-0.724814915	0.133178151
H	2.224409606	0.717122786	-0.882209736
C	2.596990050	1.073916500	1.226124315
H	2.288866838	0.629460078	2.183630137
H	2.268226367	2.120983352	1.245779201
C	4.110554847	1.005099431	1.075074876
H	4.411899816	1.499269837	0.136651659
H	4.579248256	1.588151183	1.881734726
C	4.676403428	-0.410740111	1.093927687
H	4.304237378	-0.971988637	0.223985798
C	6.195718690	-0.424946679	1.099756178
H	6.591527855	0.069954443	1.995098253
H	6.598547806	-1.442950750	1.079491449
H	6.598213169	0.107042751	0.228090752
H	4.289370815	-0.949133186	1.975773163

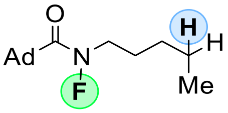

Conformer 13

C	-3.570790022	-0.726438897	-0.579201468
C	-3.638969324	0.416244536	0.437582414
C	-3.663054954	-0.164471898	1.853861721
C	-2.383844822	-0.969665340	2.092567693
C	-2.299253591	-2.109732293	1.072055887
C	-2.291747597	-1.534448533	-0.348239875
H	-3.582728711	-0.323399399	-1.602965084
H	-4.452632459	-1.377075339	-0.478635474
H	-4.547528113	1.010752378	0.265964089
H	-4.545510144	-0.808893048	1.984836274
H	-3.743120320	0.645790422	2.594067756
H	-2.390570782	-1.385649405	3.110276044
H	-3.155239881	-2.789641493	1.197451979
H	-1.388624048	-2.703480797	1.243545074
H	-2.233219771	-2.354585934	-1.078147610
C	-1.069212700	-0.628534541	-0.521347483
H	-1.050420964	-0.200348715	-1.536203344
H	-0.148709973	-1.211450773	-0.398689530
C	-2.415130055	1.319833112	0.274993902
H	-2.392320063	1.763933765	-0.729772992
H	-2.456764532	2.160380499	0.981331677
C	-1.162567438	-0.057790184	1.938475044
H	-0.246020564	-0.618689442	2.153153421
H	-1.216133784	0.771305683	2.661132371
C	-1.121745170	0.520475857	0.507990281
C	0.051108540	1.472002469	0.315911594
O	-0.077878174	2.659115175	0.060265094
N	1.334798696	0.962515067	0.314503329
F	1.535987503	-0.317051047	0.813730916
C	2.561380017	1.727217159	0.392792669
H	2.257075966	2.767430801	0.231269878
H	2.952169229	1.640239147	1.416696188
C	3.593099395	1.270092054	-0.628825688
H	3.648800104	0.171964493	-0.593265904
H	4.576029522	1.633972242	-0.300902200
C	3.334699226	1.738665885	-2.056548934
H	3.378623291	2.839578987	-2.092155616
H	4.164272126	1.387979837	-2.689833416
C	2.017833832	1.278418352	-2.673601598
H	1.176846945	1.764028048	-2.158453759
C	1.949238638	1.591458719	-4.158709451
H	2.751228516	1.083812592	-4.710174929
H	0.995269608	1.280534812	-4.598086267
H	2.059919189	2.668407934	-4.338804996
H	1.892332203	0.196312336	-2.506809546

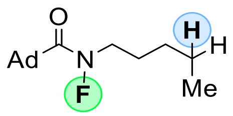

Conformer 14

C	-2.959272619	-1.645528709	-0.323794736
C	-2.624617224	-1.191098085	-1.747123508
C	-3.318914851	0.143689224	-2.031364257
C	-2.819730599	1.196560176	-1.039319080
C	-3.140980629	0.745002756	0.389133792
C	-2.459777481	-0.597576627	0.673072269
H	-2.488403365	-2.618178999	-0.116387468
H	-4.045585766	-1.783607879	-0.214359908
H	-2.967799475	-1.947313677	-2.467311950
H	-4.410084139	0.030038024	-1.944412269
H	-3.109879981	0.467023455	-3.062147406
H	-3.310425623	2.159655467	-1.240688660
H	-4.229589201	0.648162925	0.516507053
H	-2.798678992	1.502012584	1.110893984
H	-2.691430407	-0.919377701	1.698571222
C	-0.943497219	-0.434486667	0.535668497
H	-0.437720808	-1.391866712	0.736739905
H	-0.578232493	0.288361747	1.276013124
C	-1.110682765	-1.020943803	-1.887028031
H	-0.594481483	-1.974066200	-1.707374147
H	-0.846703183	-0.716867865	-2.909297303
C	-1.305380604	1.370259509	-1.189097005
H	-0.948258236	2.153058843	-0.509341751
H	-1.059918738	1.690467852	-2.213520500
C	-0.594793727	0.032647286	-0.893207162
C	0.910938720	0.149213250	-1.081756493
O	1.553475301	-0.507233174	-1.889177829
N	1.625354208	1.029680107	-0.311603282
F	0.980965082	1.823501953	0.621822194
C	3.054530799	1.266349748	-0.384191310
H	3.307755290	1.350067727	-1.447915879
H	3.213495293	2.247335773	0.079576548
C	3.912652664	0.190445186	0.267991543
H	4.951915331	0.393248138	-0.025996323
H	3.649648958	-0.774696846	-0.187322500
C	3.827905234	0.101436658	1.786684790
H	4.636062210	-0.558431171	2.138468347
H	4.039022051	1.089375661	2.229181290
C	2.505478229	-0.419622776	2.338481603
H	1.705324867	0.303566985	2.134783033
C	2.578470809	-0.691369712	3.830964939
H	1.625611985	-1.057603025	4.227578022
H	3.345676151	-1.442301177	4.059423873
H	2.839201275	0.219864955	4.385130894
H	2.226520547	-1.339092428	1.797878478

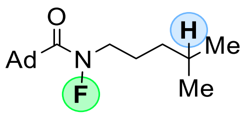

Conformer 1

C	-2.246567051	1.481160269	-2.222234699
C	-2.313437278	0.528041663	-1.024063237
C	-2.848814147	-0.830600065	-1.480561165
C	-1.916648651	-1.409801867	-2.547914567
C	-1.863518081	-0.461634379	-3.748560486
C	-1.323650018	0.897231131	-3.297266756
H	-1.876462035	2.466350852	-1.900673587
H	-3.254488682	1.634915166	-2.635752039
H	-2.975024957	0.949263609	-0.253496205
H	-3.866267026	-0.719241239	-1.884666013
H	-2.915680404	-1.517567263	-0.623610332
H	-2.288057997	-2.393387974	-2.868798527
H	-2.867192072	-0.344517355	-4.184421540
H	-1.218150719	-0.882361691	-4.534238452
H	-1.277872898	1.582787003	-4.155502234
C	0.086545371	0.730062621	-2.722362835
H	0.493909375	1.706077887	-2.435667728
H	0.759282364	0.308119000	-3.484790021
C	-0.909891410	0.361850660	-0.433728900
H	-0.539351831	1.330736669	-0.077662570
H	-0.935320861	-0.320558992	0.431843857
C	-0.513534394	-1.574711201	-1.962740783
H	0.166712896	-2.014668563	-2.705740417
H	-0.527414930	-2.267974415	-1.111050942
C	0.043059115	-0.215507360	-1.505456724
C	1.419801872	-0.461454710	-0.905617195
O	1.880348886	-1.573491920	-0.709193668
N	2.254209374	0.612463118	-0.616633302
F	1.637869808	1.860501088	-0.501519692
C	3.329891343	0.498494752	0.359959077
H	4.010705046	-0.259843753	-0.038857492
H	3.852636826	1.461783793	0.334677024
C	2.885948724	0.129097317	1.768707285
H	3.795046879	0.033136418	2.379223297
H	2.429109172	-0.870704499	1.736831702
C	1.923395896	1.120921065	2.405610126
H	2.401308099	2.113855431	2.480653444
H	1.056367977	1.260642591	1.743671417
C	1.410727089	0.701011405	3.785254675
H	0.947044700	-0.295253034	3.679753301
C	0.343719073	1.678175329	4.263260573
H	-0.485251643	1.748627199	3.548613841
H	-0.071928150	1.383214395	5.234384895
H	0.769392282	2.685303673	4.378750939
C	2.531963902	0.601544458	4.812665398
H	2.139772039	0.352096901	5.806027779
H	3.272029140	-0.163607561	4.552147507
H	3.059544499	1.562579362	4.897932934

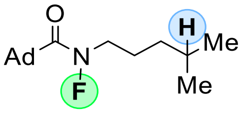

Conformer 2

C	-3.112810235	-1.410698678	0.713671362
C	-1.967024975	-1.833902707	-0.208025431
C	-2.478743759	-1.933152613	-1.648927854
C	-3.028912401	-0.574972538	-2.096910974
C	-4.172180490	-0.155787978	-1.170321887
C	-3.647657386	-0.048573395	0.263860485
H	-2.759596612	-1.353657389	1.754284372
H	-3.916309623	-2.162145919	0.686737416
H	-1.572044042	-2.808677572	0.112296382
H	-3.266358457	-2.698560684	-1.715251333
H	-1.664399697	-2.249732418	-2.318041725
H	-3.396345645	-0.649222017	-3.130421549
H	-4.990257246	-0.890099394	-1.221955807
H	-4.585806090	0.810811315	-1.495050033
H	-4.460090789	0.269011394	0.932841717
C	-2.523370272	0.986884468	0.322157710
H	-2.147613825	1.094629587	1.348590634
H	-2.895067527	1.977894969	0.025741278
C	-0.840582452	-0.798377382	-0.140015021
H	-0.469178548	-0.711529220	0.894330192
H	0.001787119	-1.118586276	-0.765401567
C	-1.909289640	0.468796206	-2.044172890
H	-2.290800138	1.451599523	-2.361508729
H	-1.107399551	0.193459908	-2.737913465
C	-1.365091361	0.578734286	-0.604922558
C	-0.280898074	1.643073927	-0.480945905
O	-0.329995278	2.554899585	0.326341880
N	0.777495180	1.648353894	-1.384741503
F	1.013918852	0.454672704	-2.066156049
C	2.039112456	2.319702150	-1.128972974
H	2.419986998	2.696041635	-2.085974480
H	1.761731718	3.180227447	-0.511559727
C	3.079155867	1.450581434	-0.434662278
H	3.964691895	2.080818415	-0.274085618
H	3.394037665	0.644962075	-1.113172074
C	2.598370610	0.851377250	0.877675753
H	2.261418391	1.652561536	1.557394184
H	1.708749314	0.233210658	0.677804636
C	3.629812255	-0.018480196	1.594502609
H	3.987607105	-0.777193923	0.876790381
C	2.976259094	-0.737271202	2.767764145
H	2.129648411	-1.354587713	2.441541600
H	3.687527092	-1.387612043	3.290555626
H	2.593313618	-0.010166437	3.497825085
C	4.827603222	0.791895760	2.073636299
H	4.500242491	1.586028652	2.759751531
H	5.541252084	0.160148457	2.615734901
H	5.371706057	1.267004264	1.249161925

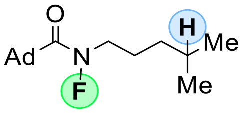

Conformer 3

C	-3.787365878	0.757060695	-2.101030720
C	-2.771287141	1.844257105	-1.744017915
C	-2.921366955	2.218933828	-0.265713820
C	-2.686515895	0.982464473	0.608536642
C	-3.702403474	-0.102275924	0.243996809
C	-3.535892197	-0.477387240	-1.231042077
H	-3.700161638	0.493701050	-3.165842686
H	-4.810777390	1.130117427	-1.944114245
H	-2.942160456	2.732635265	-2.368896668
H	-3.927810190	2.623618451	-0.081322530
H	-2.202826725	3.009616335	-0.001663468
H	-2.796484915	1.253914171	1.668257532
H	-4.725023625	0.258897413	0.431053428
H	-3.552404978	-0.988268699	0.879121826
H	-4.252282425	-1.268250522	-1.495062245
C	-2.115369387	-0.992012384	-1.471491695
H	-1.981889370	-1.288399918	-2.520822161
H	-1.922991437	-1.892342342	-0.871477738
C	-1.351697027	1.327016176	-1.995679008
H	-1.232961529	1.045647039	-3.053592580
H	-0.620947122	2.116071247	-1.783932509
C	-1.266053442	0.460224912	0.375234275
H	-1.081661479	-0.432954234	0.993174073
H	-0.534046730	1.219060068	0.675621549
C	-1.083393816	0.090430241	-1.111737726
C	0.301687393	-0.486296510	-1.372927659
O	0.494277921	-1.591363917	-1.853221319
N	1.408473701	0.234909007	-0.973303445
F	1.249371413	1.572621155	-0.639398373
C	2.788794724	-0.052794448	-1.282050042
H	2.780381652	-1.101113197	-1.599083306
H	3.110199897	0.558797350	-2.137888641
C	3.693830807	0.162456608	-0.078443663
H	3.714857258	1.233106007	0.173585771
H	4.715239478	-0.102670182	-0.383292811
C	3.263733672	-0.642508534	1.138517768
H	2.217803553	-0.392267799	1.375458237
H	3.266634758	-1.718468050	0.889852823
C	4.115057682	-0.414500550	2.386890058
H	4.088667081	0.664793685	2.617409341
C	3.519581666	-1.173336292	3.565697514
H	2.478174610	-0.882794508	3.749585190
H	3.532822221	-2.255553750	3.371384922
H	4.086740441	-0.997047419	4.487392609
C	5.567484903	-0.825031110	2.175831869
H	6.146516430	-0.718685249	3.101097247
H	5.626292075	-1.879507572	1.869050092
H	6.067035812	-0.225016563	1.406529192

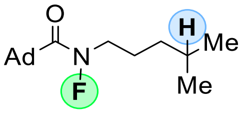

Conformer 4

C	-3.361922247	-2.020339052	-0.474775499
C	-2.608234250	-1.086376581	-1.427550884
C	-3.414981346	0.198550343	-1.628780890
C	-3.612614193	0.890979000	-0.277441325
C	-4.381960903	-0.039957126	0.663073755
C	-3.576112364	-1.323548835	0.873477562
H	-2.794133836	-2.952164039	-0.332131477
H	-4.332393626	-2.298148486	-0.912589179
H	-2.458395528	-1.587215635	-2.394800311
H	-4.390715213	-0.034757855	-2.080937542
H	-2.889164916	0.869367755	-2.324698418
H	-4.176981807	1.823610526	-0.419446553
H	-5.369128342	-0.277039869	0.238371209
H	-4.557451768	0.458120339	1.628431319
H	-4.118595988	-1.996180909	1.553129899
C	-2.215858616	-0.986957590	1.492788634
H	-1.655889738	-1.907129221	1.687961575
H	-2.354477305	-0.479350513	2.459757745
C	-1.240654110	-0.746740188	-0.828353461
H	-0.637002790	-1.656253996	-0.720629159
H	-0.690133000	-0.064005850	-1.495522242
C	-2.248743587	1.223248995	0.330015272
H	-2.369490577	1.743286638	1.291258009
H	-1.690520491	1.907921489	-0.322123896
C	-1.429315905	-0.060785999	0.544488259
C	-0.066007767	0.339219150	1.082958400
O	0.325799404	1.491743756	1.128920542
N	0.791863076	-0.635641595	1.600948259
F	0.509700836	-1.948104972	1.202586933
C	2.231448476	-0.445863404	1.528180427
H	2.418521824	0.539793333	1.965638593
H	2.687546924	-1.197857918	2.183685251
C	2.776745090	-0.529192126	0.111538695
H	2.274401512	0.237730253	-0.495379054
H	2.511988490	-1.506452471	-0.319545843
C	4.284108151	-0.335675996	0.080273286
H	4.530012315	0.689111505	0.409867399
H	4.751034876	-1.009368135	0.817681927
C	4.932583939	-0.587308383	-1.281016679
H	4.703467164	-1.628750724	-1.568935885
C	6.443397145	-0.433896676	-1.178437994
H	6.869980939	-1.098670280	-0.417552388
H	6.936113028	-0.651294215	-2.133027373
H	6.705346864	0.597919417	-0.900437844
C	4.382895564	0.338179582	-2.356225548
H	4.881631213	0.161936994	-3.317247653
H	3.307062174	0.203722208	-2.514656823
H	4.552972502	1.389566118	-2.081140158

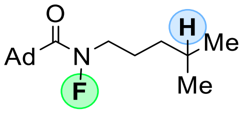

Conformer 5

C	-3.208096865	0.750697278	-0.215392923
C	-3.111496150	0.349161521	1.260404433
C	-3.891662228	-0.947388433	1.488115639
C	-3.297613429	-2.055840857	0.615248651
C	-3.407412808	-1.658332788	-0.859118198
C	-2.627713062	-0.362832317	-1.093807193
H	-2.662297741	1.690610533	-0.387862182
H	-4.258522780	0.933523999	-0.487010890
H	-3.529076011	1.149421371	1.888025348
H	-4.953872731	-0.798320476	1.241934533
H	-3.844146759	-1.235337869	2.549095501
H	-3.842983003	-2.994738637	0.786804235
H	-4.462976465	-1.519400000	-1.137438544
H	-3.008403793	-2.460485430	-1.497987973
H	-2.697268708	-0.072886759	-2.152016949
C	-1.153685648	-0.572657503	-0.734639206
H	-0.586423826	0.344577857	-0.936769090
H	-0.718939914	-1.372933975	-1.355271648
C	-1.640944038	0.142308744	1.636459510
H	-1.087022093	1.079777465	1.514743034
H	-1.557316367	-0.149835768	2.694593412
C	-1.826618266	-2.262040448	0.979016981
H	-1.387360712	-3.068612785	0.376727154
H	-1.727257331	-2.573430680	2.028587990
C	-1.029294279	-0.965485892	0.755396686
C	0.423712756	-1.253124348	1.104788080
O	0.847983598	-2.366756706	1.363999774
N	1.329200929	-0.204540947	1.215544783
F	0.961273253	0.992067461	0.593563925
C	2.765734140	-0.416197732	1.091906612
H	3.230659153	0.549229736	1.323377676
H	3.027960782	-1.106369637	1.899567445
C	3.220334585	-0.980737259	-0.248655472
H	2.813006329	-1.995682662	-0.341544640
H	4.312834556	-1.096748663	-0.205489586
C	2.820006344	-0.159897261	-1.469971420
H	3.189727948	-0.675157399	-2.371211911
H	1.722539607	-0.159685833	-1.560038306
C	3.319039328	1.284735288	-1.507735605
H	2.934955889	1.810640814	-0.620944448
C	2.757874623	1.985243129	-2.738230969
H	3.102982083	1.489252650	-3.656413694
H	1.660353097	1.964889220	-2.742639503
H	3.075836885	3.033368821	-2.786468386
C	4.841112239	1.359670618	-1.498727938
H	5.278088491	0.947466433	-0.580749344
H	5.257705896	0.796046834	-2.346340131
H	5.187148336	2.395918548	-1.589321300

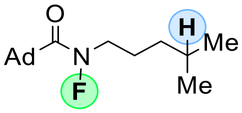

Conformer 6

C	-4.399415765	-0.912062891	1.811141433
C	-3.236094847	-1.717942207	1.229945407
C	-3.495862941	-1.990511390	-0.255363465
C	-3.635307837	-0.663306594	-1.008411398
C	-4.797849253	0.139089109	-0.420758048
C	-4.523028186	0.415320604	1.059221225
H	-4.232084266	-0.725584738	2.882599417
H	-5.335429335	-1.484536252	1.726511951
H	-3.137638857	-2.671091923	1.768968600
H	-4.412897183	-2.587199321	-0.372139421
H	-2.670552615	-2.580858587	-0.681185755
H	-3.821450410	-0.861600652	-2.073634428
H	-5.739724294	-0.418621461	-0.534394289
H	-4.916707123	1.087015988	-0.966818312
H	-5.346859615	1.005506235	1.485052208
C	-3.221211772	1.205530544	1.201620790
H	-3.019289280	1.432594700	2.256999841
H	-3.298771877	2.174404788	0.688224783
C	-1.933536824	-0.926700117	1.382534892
H	-1.741113836	-0.715537034	2.446067073
H	-1.088395084	-1.515358907	1.007532695
C	-2.335067769	0.134423868	-0.874144358
H	-2.421284776	1.093695381	-1.407647002
H	-1.507560550	-0.417757240	-1.332729423
C	-2.042436677	0.409714758	0.614990549
C	-0.781209010	1.239311764	0.813250180
O	-0.752457474	2.268962927	1.463009670
N	0.391830842	0.869087647	0.155008447
F	0.458931680	-0.461689507	-0.263066843
C	1.704301353	1.273690746	0.619264806
H	1.546579311	2.240470349	1.108533456
H	2.052007173	0.562571170	1.386098266
C	2.701447088	1.377753805	-0.518300406
H	2.301329586	2.073432898	-1.268328946
H	2.791525913	0.399453591	-1.013561910
C	4.063929477	1.836959659	-0.021180377
H	3.986336521	2.868023959	0.365778415
H	4.363530035	1.215946672	0.839028210
C	5.173232734	1.787595097	-1.069978340
H	5.261768107	0.742574202	-1.413600383
C	6.499009911	2.204975017	-0.448919540
H	6.756076889	1.581057754	0.415799021
H	7.320696381	2.137161740	-1.171575976
H	6.447894516	3.247307002	-0.103011854
C	4.855586297	2.662656568	-2.276160329
H	5.688147499	2.668565703	-2.989796579
H	3.962671566	2.325525945	-2.814548453
H	4.683751997	3.701709999	-1.959522241

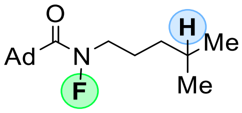

Conformer 7

C	-2.564620181	-2.373415307	-0.270680519
C	-2.989055165	-1.368183002	-1.346862396
C	-4.044127231	-0.420562166	-0.772092374
C	-3.453400511	0.328987324	0.425000142
C	-3.044946638	-0.677276135	1.504350294
C	-1.987706053	-1.627357790	0.937401028
H	-1.815128366	-3.068902945	-0.677798230
H	-3.429715826	-2.978232857	0.039900231
H	-3.403745433	-1.906713819	-2.211063598
H	-4.934382334	-0.988667734	-0.462524729
H	-4.369845411	0.294012028	-1.542840627
H	-4.201142474	1.024634333	0.831633822
H	-3.922982977	-1.247665739	1.843118274
H	-2.645339699	-0.148800810	2.382826660
H	-1.685663100	-2.350939435	1.708085227
C	-0.757324788	-0.829424791	0.496532787
H	0.017005399	-1.511511049	0.124940479
H	-0.327697162	-0.280891551	1.350935032
C	-1.767152951	-0.564930897	-1.803535250
H	-1.027762270	-1.233799130	-2.258426066
H	-2.060810365	0.167870990	-2.570967729
C	-2.225818188	1.124247595	-0.022749068
H	-1.799652327	1.686372708	0.819459147
H	-2.501161524	1.869876457	-0.781363410
C	-1.156216771	0.181702846	-0.599323792
C	0.041754506	1.032027328	-0.998297123
O	0.087708101	2.245203619	-0.852883212
N	1.168162752	0.409146770	-1.480154856
F	1.140809751	-0.947586983	-1.754047665
C	2.409061058	1.006941507	-1.905691851
H	2.510947235	0.901409281	-2.994946373
H	2.282707490	2.071059810	-1.683150341
C	3.606796862	0.401802628	-1.180911478
H	4.496329338	0.986710762	-1.451409614
H	3.781233769	-0.611076101	-1.566589790
C	3.434443131	0.335296502	0.332635817
H	2.628109900	-0.378785087	0.564051587
H	4.350473444	-0.091492870	0.772110827
C	3.135635739	1.662032391	1.030841917
H	2.187949963	2.066472044	0.638838206
C	2.956059864	1.423455692	2.524885958
H	2.168693044	0.685686310	2.724913106
H	3.885336546	1.039771487	2.970707279
H	2.693700798	2.348834879	3.049450503
C	4.238268869	2.689491000	0.788121560
H	5.211644077	2.293056283	1.111908161
H	4.330712529	2.967182191	-0.268636454
H	4.050608836	3.611348081	1.351195660

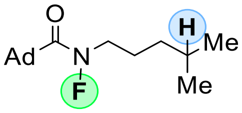

Conformer 8

C	-2.781258348	0.101571690	-2.195295359
C	-3.166177225	0.774819966	-0.875553500
C	-4.011912168	-0.189340029	-0.039613056
C	-3.196126079	-1.448786447	0.260040606
C	-2.794982602	-2.124417598	-1.055466156
C	-1.960854225	-1.155999079	-1.900862162
H	-2.198091614	0.796123654	-2.818494944
H	-3.685064353	-0.161548471	-2.765245824
H	-3.740933682	1.689089122	-1.080487725
H	-4.932314578	-0.454424084	-0.581443259
H	-4.319115669	0.293897587	0.900030042
H	-3.795407997	-2.144778251	0.864397695
H	-3.694364295	-2.428362215	-1.611590467
H	-2.218863187	-3.039221666	-0.849891278
H	-1.673142857	-1.642369390	-2.843907074
C	-0.690140721	-0.776329580	-1.133856529
H	-0.087231754	-0.071556476	-1.729657777
H	-0.074980141	-1.667565072	-0.961570922
C	-1.900049399	1.147188563	-0.102518165
H	-1.288000045	1.855542348	-0.678142765
H	-2.156127745	1.656026960	0.837195332
C	-1.934622702	-1.074319101	1.043448062
H	-1.368104852	-1.977459681	1.296585225
H	-2.207511374	-0.584603945	1.991012120
C	-1.067940274	-0.109418237	0.207332525
C	0.174548734	0.339440919	0.967803970
O	0.438051638	1.512467052	1.180303512
N	1.015834783	-0.612272375	1.524503472
F	0.867206914	-1.920568748	1.073751108
C	2.399770005	-0.376021832	1.890238528
H	2.427344162	0.676108188	2.190640683
H	2.618933430	-0.982538575	2.777090013
C	3.387379989	-0.681767789	0.768637081
H	3.346391684	-1.756382565	0.550899810
H	4.397631699	-0.490416793	1.155852733
C	3.142217895	0.104238887	-0.513541780
H	3.839963748	-0.255385535	-1.287093289
H	2.136879083	-0.142707051	-0.893271345
C	3.280560808	1.624528401	-0.410035812
H	2.618936923	1.985129729	0.391319518
C	2.806025281	2.267205362	-1.705689719
H	3.387333889	1.900085792	-2.564103828
H	1.747940168	2.038117051	-1.891544961
H	2.909091521	3.358242170	-1.674417478
C	4.711400343	2.038521736	-0.090443801
H	5.050692747	1.651794318	0.878650747
H	5.405078074	1.666963167	-0.858985083
H	4.808393587	3.129916709	-0.056602565

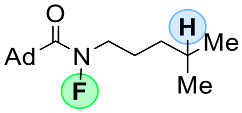

Conformer 9

C	-3.601692513	-0.829381868	1.716744350
C	-2.796689652	-1.719995405	0.767531639
C	-3.621303982	-2.001445278	-0.493062645
C	-3.972220034	-0.681893804	-1.189131697
C	-4.774312558	0.203607565	-0.233135461
C	-3.935134509	0.489968224	1.014647540
H	-3.024403652	-0.635504335	2.633177012
H	-4.527326063	-1.340952330	2.020828945
H	-2.549049538	-2.667828811	1.266610463
H	-4.541838219	-2.541656848	-0.225799580
H	-3.054315788	-2.649937817	-1.177906443
H	-4.565839187	-0.888219468	-2.091152830
H	-5.714023128	-0.295445616	0.047908092
H	-5.046383404	1.146519182	-0.730833945
H	-4.498301261	1.140563997	1.698821327
C	-2.639166930	1.195820784	0.611802910
H	-2.032699682	1.427425174	1.497427722
H	-2.857299249	2.158463230	0.127370490
C	-1.496507297	-1.012237941	0.375416442
H	-0.896918634	-0.789657480	1.272731848
H	-0.891269061	-1.660537635	-0.269447424
C	-2.681710096	0.033997398	-1.599387719
H	-2.917957683	0.987973320	-2.095394322
H	-2.127759361	-0.576599887	-2.319793632
C	-1.824083230	0.313845457	-0.349420148
C	-0.526521676	1.038233785	-0.664226194
O	-0.157360957	2.039444436	-0.074787323
N	0.283604906	0.589596962	-1.710549170
F	0.073199060	-0.738674269	-2.098398159
C	1.716396094	0.827930039	-1.661291915
H	2.120330647	0.594938870	-2.654613741
H	1.816546382	1.905736649	-1.505044633
C	2.413814288	0.032368245	-0.568134377
H	2.231214305	-1.036519947	-0.740769451
H	1.943608654	0.279484181	0.394767258
C	3.912788585	0.299759899	-0.507525343
H	4.345769563	-0.299537803	0.309368851
H	4.388477881	-0.064751814	-1.432420687
C	4.312756445	1.764446737	-0.292481792
H	4.014361801	2.344093272	-1.182813110
C	5.826629788	1.866986119	-0.158475191
H	6.169332291	1.327824807	0.736308138
H	6.339427336	1.429619739	-1.023693795
H	6.155458701	2.909043358	-0.062761783
C	3.623552176	2.368042153	0.926271672
H	2.535051370	2.425603624	0.810753713
H	3.834999837	1.764754539	1.821477916
H	3.988708714	3.383506638	1.121669177

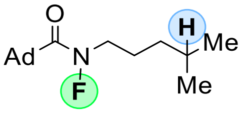

Conformer 10

C	-3.390213952	0.464322194	-1.786166162
C	-3.606178650	0.827695964	-0.314456845
C	-4.171981080	-0.383719918	0.430843406
C	-3.175626364	-1.541619284	0.342611975
C	-2.942759570	-1.906660036	-1.127413639
C	-2.392313609	-0.692003385	-1.882446353
H	-3.011926927	1.336629952	-2.339933997
H	-4.346279646	0.177825505	-2.249621077
H	-4.309190088	1.669583123	-0.241206753
H	-5.137938390	-0.680402302	-0.005017963
H	-4.359181335	-0.126337958	1.484185968
H	-3.571453281	-2.414762252	0.880779125
H	-3.886438815	-2.237048202	-1.586714374
H	-2.237062783	-2.747958916	-1.199865275
H	-2.228505865	-0.955773974	-2.937127439
C	-1.055985442	-0.271769034	-1.263653981
H	-0.652663700	0.609767035	-1.787506929
H	-0.319698449	-1.078144952	-1.366375796
C	-2.272170206	1.238510556	0.310973873
H	-1.860528118	2.120359097	-0.197566855
H	-2.410357141	1.526362117	1.363079841
C	-1.844640417	-1.130342158	0.979211904
H	-1.143614942	-1.971282068	0.958247344
H	-1.996650002	-0.860446262	2.035634896
C	-1.261393754	0.082146401	0.227193657
C	0.055512314	0.569873504	0.807812325
O	0.273188249	1.733236659	1.097135739
N	1.073341326	-0.345799002	1.087959191
F	0.979658108	-1.568554431	0.411369261
C	2.463244788	0.079842974	1.052754132
H	2.517329327	0.962699395	1.698283438
H	3.045844512	-0.719144959	1.526096375
C	2.952405352	0.397049092	-0.352486189
H	2.265332878	1.131922165	-0.794821028
H	2.882530397	-0.509233729	-0.969705517
C	4.367413994	0.961353502	-0.373800336
H	4.650723763	1.168424957	-1.418341465
H	4.370915495	1.936771788	0.138129015
C	5.452788747	0.079211840	0.247663237
H	5.235346124	-0.043143559	1.322225217
C	6.804524844	0.769649187	0.124187167
H	7.599994255	0.184752052	0.600803219
H	7.074828543	0.896424596	-0.933837037
H	6.793871748	1.764718963	0.585121943
C	5.502171128	-1.301666953	-0.393030951
H	6.325415617	-1.898156283	0.017381779
H	4.576646000	-1.870457176	-0.242575736
H	5.665034771	-1.213397591	-1.476930177

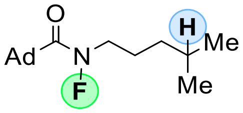

Conformer 11

C	-4.219356188	-0.999692356	-1.362815739
C	-4.161584974	-0.153559600	-0.088160099
C	-4.250622273	-1.069237696	1.135258313
C	-3.069114634	-2.041474353	1.126241953
C	-3.111009550	-2.886298853	-0.151644946
C	-3.037911895	-1.971991795	-1.379144575
H	-4.186010379	-0.349925616	-2.250206955
H	-5.167778659	-1.556189684	-1.406097659
H	-4.998610004	0.559033485	-0.079209507
H	-5.199681742	-1.626262055	1.124349648
H	-4.239259310	-0.469764109	2.057983886
H	-3.122616375	-2.699641722	2.005268917
H	-4.037583809	-3.479019027	-0.180075127
H	-2.272228165	-3.598595318	-0.160906161
H	-3.068797324	-2.579725451	-2.294867231
C	-1.722786514	-1.187225452	-1.350749420
H	-1.662211445	-0.517771029	-2.223142249
H	-0.871837163	-1.875578994	-1.406743124
C	-2.845707802	0.625160443	-0.051082791
H	-2.772838957	1.304448578	-0.910981366
H	-2.796435781	1.258593524	0.846015454
C	-1.753379669	-1.258924839	1.174424039
H	-0.906339847	-1.952732346	1.211622366
H	-1.710653347	-0.643982212	2.086652308
C	-1.648053009	-0.340592186	-0.060025569
C	-0.377779750	0.498632384	-0.052326931
O	-0.374458960	1.709938980	-0.181402234
N	0.847717745	-0.118031098	0.196576988
F	0.888280904	-1.497649970	-0.016631455
C	2.107178471	0.450646168	-0.245086772
H	2.281829182	0.149993555	-1.290206541
H	1.952025711	1.534939487	-0.231863862
C	3.256505667	0.037945705	0.656433055
H	3.374852943	-1.053474019	0.616096655
H	2.982772229	0.277099184	1.692581865
C	4.563271907	0.734791446	0.297278580
H	4.451861003	1.817556436	0.467217038
H	5.346681338	0.398495442	0.995330721
C	5.072288865	0.513602986	-1.129902379
H	4.342771117	0.948477266	-1.833803047
C	6.395124615	1.244408151	-1.322074208
H	7.166196233	0.822305527	-0.661791618
H	6.759125072	1.153708217	-2.352173729
H	6.305469291	2.312420434	-1.088650782
C	5.230750902	-0.967581284	-1.454738038
H	5.894679112	-1.449935242	-0.722955418
H	4.276181231	-1.506954406	-1.439862685
H	5.673603186	-1.110955049	-2.447650762

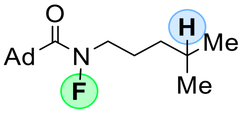

Conformer 12

C	-4.823288344	-0.007695992	-0.111208377
C	-4.182653414	-0.898194499	-1.178797653
C	-4.146834691	-0.148183143	-2.513037426
C	-3.311515785	1.124208429	-2.356438320
C	-3.936962452	2.017575841	-1.280271421
C	-3.989025832	1.264304327	0.053002713
H	-4.881834108	-0.547053926	0.846143087
H	-5.853689995	0.249667001	-0.399613353
H	-4.767340784	-1.822452145	-1.289834166
H	-5.168544270	0.107273258	-2.832238479
H	-3.715332211	-0.789435924	-3.296197418
H	-3.276231153	1.666603233	-3.312187980
H	-4.951866242	2.315316194	-1.583444119
H	-3.349972755	2.941759479	-1.168916512
H	-4.439324200	1.907780032	0.822417123
C	-2.565662169	0.899647233	0.485112487
H	-2.587360722	0.353415735	1.440781918
H	-1.978513659	1.810270995	0.645778981
C	-2.757241050	-1.259291113	-0.757572755
H	-2.763633922	-1.820298978	0.187732886
H	-2.287935214	-1.916608296	-1.501725915
C	-1.883279663	0.756808615	-1.943708968
H	-1.267113268	1.660662100	-1.872554207
H	-1.424785623	0.105258722	-2.704059133
C	-1.906756316	0.011851087	-0.590219849
C	-0.498986544	-0.421443388	-0.205501205
O	-0.136259985	-1.584458925	-0.193112792
N	0.414724084	0.534374301	0.233161722
F	0.126116620	1.857563540	-0.101720952
C	1.849050021	0.337700473	0.169884700
H	1.981790611	-0.748038450	0.211494059
H	2.217098306	0.683969591	-0.810536484
C	2.569909179	1.052751495	1.299040949
H	2.244062265	0.626933928	2.258171886
H	2.254115227	2.104051110	1.305734018
C	4.084112557	0.979286355	1.152385947
H	4.381687017	1.512527278	0.234804822
H	4.552573657	1.528987071	1.985004503
C	4.680806238	-0.430322627	1.112294288
H	4.275464240	-0.953814466	0.228800337
C	6.191871563	-0.341982037	0.950394519
H	6.642099671	0.149685487	1.824604893
H	6.647022373	-1.335669221	0.860647805
H	6.471490674	0.238360047	0.062864842
C	4.325792404	-1.234936696	2.355940973
H	3.250351980	-1.428782516	2.443422950
H	4.834629416	-2.206322321	2.353167282
H	4.642976978	-0.697530642	3.261953203

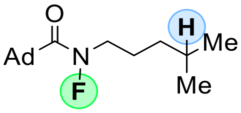

Conformer 13

C	-3.624111540	-0.751984812	-0.518008367
C	-3.668016923	0.429054312	0.454872254
C	-3.683765072	-0.096713518	1.892365399
C	-2.412810785	-0.908673786	2.150355711
C	-2.352796197	-2.087425182	1.172958405
C	-2.352734176	-1.565707654	-0.267988246
H	-3.641444956	-0.388311503	-1.556396100
H	-4.512573639	-1.387515830	-0.384240760
H	-4.571046899	1.027956051	0.269973391
H	-4.572757138	-0.724064090	2.057424122
H	-3.746282471	0.742518786	2.601345643
H	-2.415499874	-1.285314987	3.183255691
H	-3.215896309	-2.751020753	1.332167398
H	-1.447954836	-2.685974030	1.357639677
H	-2.309933342	-2.413213808	-0.966987060
C	-1.120696542	-0.682618611	-0.485270093
H	-1.105025131	-0.294836616	-1.516512529
H	-0.209167820	-1.275884989	-0.347352640
C	-2.435255711	1.310439801	0.247140579
H	-2.416100676	1.715453647	-0.774234173
H	-2.460568649	2.177757699	0.920900799
C	-1.182400121	-0.018279852	1.951183500
H	-0.269716912	-0.581366943	2.177388957
H	-1.219402827	0.838042865	2.642499026
C	-1.148862824	0.505317616	0.499371485
C	0.029919551	1.442601672	0.275646845
O	-0.093817171	2.627069365	0.009258394
N	1.314657980	0.927655523	0.267105759
F	1.510401066	-0.344382100	0.794082506
C	2.532965012	1.703582836	0.385741251
H	2.221036304	2.741210245	0.221382833
H	2.892519463	1.614085181	1.420897540
C	3.604638064	1.275487419	-0.605545430
H	3.714794486	0.183169585	-0.557118817
H	4.561666601	1.685062935	-0.255044242
C	3.370700021	1.741349440	-2.039501140
H	3.371758745	2.843344949	-2.055798644
H	4.235384897	1.431325492	-2.649886928
C	2.101148442	1.247720218	-2.738538587
H	1.226774390	1.643247805	-2.197128770
C	2.061606913	1.800749177	-4.157107919
H	2.909637505	1.420089784	-4.744662451
H	1.141517610	1.506518447	-4.676096483
H	2.116175590	2.896066932	-4.165782658
C	2.017892910	-0.273010399	-2.760860521
H	1.144911306	-0.610562788	-3.333527294
H	2.910809941	-0.700498270	-3.241066048
H	1.938247869	-0.702044964	-1.755193097

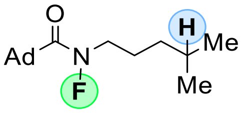

Conformer 14

C	-3.000788175	-1.622213906	-0.346617265
C	-2.649665360	-1.163474051	-1.764610627
C	-3.329674790	0.178926976	-2.047794808
C	-2.830403059	1.221675410	-1.045290083
C	-3.167577530	0.765603691	0.377793377
C	-2.500302486	-0.584138151	0.660088440
H	-2.540989648	-2.600350863	-0.140455508
H	-4.089285648	-1.750383146	-0.247376500
H	-2.993069707	-1.912829929	-2.491805354
H	-4.422539431	0.074464888	-1.971575530
H	-3.108310014	0.506319529	-3.074723199
H	-3.310467135	2.190377895	-1.245411630
H	-4.258046751	0.677898226	0.495769740
H	-2.824747176	1.515943315	1.106284939
H	-2.743366565	-0.909362443	1.681830767
C	-0.981341599	-0.434887551	0.536251990
H	-0.487382212	-1.398782892	0.734176891
H	-0.614783024	0.279860377	1.284397505
C	-1.133109625	-1.006062290	-1.890498799
H	-0.626337533	-1.964300392	-1.710372956
H	-0.857619885	-0.699660526	-2.909038190
C	-1.313586229	1.382808740	-1.180472175
H	-0.955637453	2.158555451	-0.492921940
H	-1.056317833	1.707478464	-2.200656395
C	-0.616539452	0.038082730	-0.887069775
C	0.891507531	0.143965512	-1.065905664
O	1.533442945	-0.513779107	-1.873584687
N	1.612617145	1.003735022	-0.278388785
F	0.969237961	1.803606122	0.650301748
C	3.033624969	1.278268665	-0.387785616
H	3.258140460	1.361806945	-1.457995950
H	3.175281649	2.265861386	0.067411119
C	3.938927120	0.232218624	0.247189397
H	4.970158422	0.507486193	-0.015416447
H	3.751644866	-0.729039091	-0.248944017
C	3.839309452	0.097578902	1.762284309
H	4.679121666	-0.529305050	2.105740341
H	3.998035285	1.086612314	2.224121995
C	2.552533443	-0.508047839	2.328860778
H	1.715138986	0.166744786	2.098835016
C	2.669281834	-0.608804895	3.844931242
H	1.747600610	-0.998754741	4.293285514
H	3.486185500	-1.289366474	4.125369954
H	2.879112975	0.366480154	4.301049429
C	2.255233228	-1.878513738	1.731737510
H	3.109390591	-2.556094966	1.877215507
H	1.386869894	-2.337559446	2.222148201
H	2.044529902	-1.837018663	0.655726558

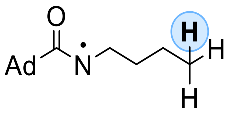

Conformer 1

C	-2.210785583	1.441257380	-2.135705047
C	-2.135792263	0.551242224	-0.891304050
C	-2.699560864	-0.834132834	-1.220567894
C	-1.883352072	-1.466517462	-2.351535282
C	-1.959690032	-0.574248406	-3.594878260
C	-1.394570584	0.810895340	-3.268152337
H	-1.825623017	2.446116981	-1.905840150
H	-3.258645156	1.561906399	-2.448810042
H	-2.717301916	1.004925595	-0.076151112
H	-3.755632493	-0.748883706	-1.517866238
H	-2.668729822	-1.476899127	-0.327661873
H	-2.286184313	-2.462004718	-2.585804495
H	-3.003221440	-0.484018579	-3.932143088
H	-1.392127759	-1.028106826	-4.420881931
H	-1.442785518	1.451828612	-4.160119665
C	0.066338801	0.676130418	-2.829954634
H	0.497288586	1.662485950	-2.604582022
H	0.670866860	0.229342765	-3.633897482
C	-0.674235114	0.419039697	-0.452460765
H	-0.260668909	1.409282845	-0.207486353
H	-0.606995260	-0.198796010	0.459398118
C	-0.423568127	-1.602943669	-1.913607472
H	0.182755250	-2.066720560	-2.702679760
H	-0.343453254	-2.258505632	-1.032346671
C	0.153439029	-0.222766272	-1.577237346
C	1.615832186	-0.352671621	-1.202457115
O	2.320914448	-1.285736052	-1.558853071
N	2.156557218	0.745480361	-0.538691822
C	3.118301820	0.434571731	0.483391839
H	3.760935590	-0.375589144	0.102436153
H	3.733550672	1.321538469	0.681476607
C	2.443822306	-0.033720634	1.777754101
H	3.225076805	-0.385608160	2.466846365
H	1.815208747	-0.911161593	1.553494228
C	1.617257387	1.053753873	2.449488719
H	2.277324585	1.894417657	2.711000732
H	0.890275843	1.456031567	1.729382877
C	0.898625252	0.546674452	3.687667365
H	1.607972834	0.135350963	4.417324801
H	0.332800482	1.340794665	4.186118533
H	0.192633991	-0.254739674	3.432644672

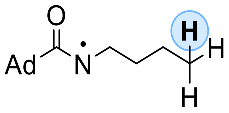

Conformer 2

C	-2.568411634	-1.598877595	0.679945964
C	-1.714157539	-1.844840318	-0.567187268
C	-2.599871398	-1.775756594	-1.814891937

C	-3.253895953	-0.393501486	-1.903966096
C	-4.106944694	-0.151459239	-0.655792041
C	-3.221795502	-0.216241043	0.592172156
H	-1.944484328	-1.665198388	1.584248016
H	-3.341569558	-2.377175796	0.765866277
H	-1.240564350	-2.834929713	-0.503991039
H	-3.373682263	-2.556746292	-1.770173215
H	-1.998996889	-1.968787526	-2.716264082
H	-3.885727852	-0.339794251	-2.802072564
H	-4.904299957	-0.907162641	-0.591773510
H	-4.598435196	0.830787698	-0.719000635
H	-3.833430547	-0.038868779	1.488087543
C	-2.137014653	0.859595896	0.503091118
H	-1.506445114	0.841387537	1.406475300
H	-2.578636238	1.863379813	0.449426444
C	-0.624395506	-0.771879542	-0.657069373
H	0.032979948	-0.833499576	0.226552349
H	0.009378816	-0.942064389	-1.540828019
C	-2.165995738	0.679451267	-1.993814608
H	-2.613373560	1.682274382	-2.065535405
H	-1.547743925	0.531479835	-2.891066151
C	-1.267618096	0.622326840	-0.737047769
C	-0.234465384	1.724546673	-0.870622614
O	-0.403137572	2.864141043	-0.455752536
N	0.869782651	1.399491004	-1.639763897
C	2.145788452	1.892312330	-1.207117395
H	2.810791019	1.958378073	-2.075701686
H	2.013524887	2.899837755	-0.776510600
C	2.762743373	0.968145960	-0.144781742
H	3.834382223	1.203257145	-0.076145611
H	2.698548243	-0.072667263	-0.498573320
C	2.129094956	1.094100776	1.234010649
H	2.265672826	2.121604060	1.601016719
H	1.040759704	0.949423933	1.169125032
C	2.710372141	0.093540149	2.219259180
H	3.797422314	0.211855373	2.311967102
H	2.277416947	0.206125085	3.218617848
H	2.521229328	-0.936320679	1.888152795

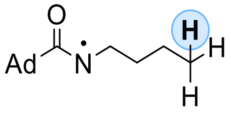

Conformer 3

C	-3.521508061	0.597618604	-2.522426610
C	-2.592413761	1.728069287	-2.068612677
C	-3.076297578	2.275686067	-0.722411220
C	-3.071962332	1.155876827	0.322788306
C	-4.002310532	0.028549746	-0.134289614
C	-3.517238343	-0.523865956	-1.478229530
H	-3.193179578	0.206916759	-3.497468785
H	-4.542946544	0.983695324	-2.658710233
H	-2.592214427	2.532635906	-2.817818775
H	-4.091090937	2.687828538	-0.827465697
H	-2.426637463	3.101253588	-0.394731870
H	-3.415455652	1.551452744	1.289320760
H	-5.031842465	0.405445130	-0.229104691
H	-4.021396664	-0.773904413	0.618330277
H	-4.182532880	-1.335210554	-1.805754513
C	-2.096230385	-1.069238628	-1.319542394
H	-1.734728558	-1.493331360	-2.267896237
H	-2.069084541	-1.886173258	-0.585262909
C	-1.169156667	1.186079061	-1.910881734
H	-0.796980203	0.804207429	-2.875243939
H	-0.485704344	1.989417698	-1.593420769
C	-1.648468197	0.614560069	0.481552521
H	-1.624416082	-0.184394932	1.238486792
H	-0.966815053	1.406215749	0.822752620
C	-1.155607868	0.051759682	-0.867434612
C	0.253414738	-0.473685939	-0.719837364
O	0.561838488	-1.656690988	-0.753637364
N	1.213154411	0.487408628	-0.441204285
C	2.481205870	0.369658613	-1.103688764
H	2.524675426	-0.537036699	-1.729275573
H	2.565186539	1.245476054	-1.771534513
C	3.623594391	0.381379522	-0.093706880
H	3.562593460	1.301192420	0.506016681
H	4.578295181	0.416750239	-0.638903357
C	3.584456537	-0.836228424	0.818599385
H	2.618084570	-0.853199460	1.343694691
H	3.599053255	-1.748787248	0.204525939
C	4.728266080	-0.849468643	1.816598824
H	4.707019694	0.043913438	2.453699254
H	4.685573464	-1.725533963	2.472407532
H	5.700344813	-0.863854493	1.306798067

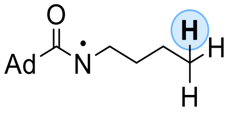

Conformer 4

C	-3.370027073	-1.984887064	-0.376796825
C	-2.422873300	-1.259478850	-1.338073776
C	-3.062443597	0.058141316	-1.787172656
C	-3.329760276	0.942420478	-0.565176830
C	-4.278815675	0.214685416	0.392179081
C	-3.639975786	-1.101636889	0.845257330
H	-2.928256561	-2.941981607	-0.061152360
H	-4.316275180	-2.219253290	-0.886971715
H	-2.225727678	-1.894239819	-2.213714635
H	-4.003392931	-0.143003460	-2.321112016
H	-2.397722840	0.579537597	-2.492599593
H	-3.785386729	1.889398752	-0.887642413
H	-5.238975360	0.015309091	-0.107066271
H	-4.494788497	0.849441643	1.264456175
H	-4.316458423	-1.623799008	1.536999032
C	-2.319525302	-0.810298079	1.563680000
H	-1.850778857	-1.742319950	1.910120709
H	-2.494280251	-0.181928194	2.450445196
C	-1.100908866	-0.965173597	-0.621593287

H	-0.617189711	-1.902197231	-0.305479445
H	-0.404656828	-0.455129575	-1.307569429
C	-2.011957981	1.236949016	0.153869632
H	-2.176314587	1.881961920	1.027435277
H	-1.322486626	1.781694540	-0.509373976
C	-1.359100882	-0.073540980	0.605297304
C	-0.070161772	0.212710356	1.346609456
O	0.216596381	1.301857805	1.821668609
N	0.731029109	-0.903370269	1.565032935
C	2.142966486	-0.689000313	1.458290064
H	2.390274860	0.232748164	2.014149970
H	2.676300900	-1.531176233	1.917108889
C	2.594823618	-0.506325708	0.004728836
H	2.100023011	0.385228277	-0.411581021
H	2.254280736	-1.365145427	-0.593219436
C	4.105589704	-0.361894182	-0.103914689
H	4.432947656	0.485929681	0.515948401
H	4.586402052	-1.253788330	0.324694026
C	4.563900254	-0.164948098	-1.539269030
H	4.117948804	0.737721383	-1.975054094
H	5.652159697	-0.064255561	-1.608628665
H	4.267595843	-1.013796657	-2.168240383

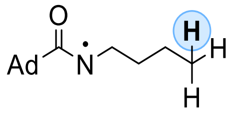

Conformer 5

C	-3.216767994	0.686687621	-0.167847257
C	-3.192421416	0.211087880	1.288155700
C	-3.891344421	-1.147812061	1.392276713
C	-3.166375185	-2.167750552	0.508428082
C	-3.192113193	-1.689989997	-0.946477287
C	-2.492895701	-0.331921562	-1.053890764
H	-2.733882073	1.671847962	-0.252287801
H	-4.256592402	0.807734193	-0.506710486
H	-3.708173993	0.943723059	1.925396996
H	-4.942010830	-1.054793936	1.078628561
H	-3.896085324	-1.491059647	2.437560102
H	-3.664725144	-3.144412122	0.585635079
H	-4.231027126	-1.607166597	-1.299666618
H	-2.690794595	-2.425150820	-1.594294936
H	-2.505366288	0.011191882	-2.098134907
C	-1.040640752	-0.466302540	-0.586874952
H	-0.521771916	0.500608811	-0.671033309
H	-0.502033650	-1.183435258	-1.228807806
C	-1.740897185	0.076589872	1.755845273
H	-1.223510260	1.045559332	1.701610738
H	-1.700605556	-0.259467942	2.803103310
C	-1.716116343	-2.303603065	0.977171240
H	-1.182022509	-3.048609247	0.366742808
H	-1.668936087	-2.658107762	2.015019159
C	-1.001715846	-0.951324464	0.870680705
C	0.415113564	-1.071782279	1.393914777
O	0.750145975	-1.902221646	2.228378846

N	1.290139978	-0.073759354	0.982217339
C	2.657833549	-0.469322856	0.804131205
H	3.305535726	0.405534253	0.939652750
H	2.906360073	-1.222771682	1.568520748
C	2.893174747	-1.090508209	-0.581095249
H	2.313653045	-2.023573687	-0.646831280
H	3.954020713	-1.375652669	-0.630421546
C	2.550951968	-0.175041775	-1.752284784
H	2.811492065	-0.696652435	-2.683261002
H	1.463818358	-0.015455781	-1.788068599
C	3.256571903	1.171043127	-1.701868628
H	4.340765595	1.049049812	-1.575867389
H	3.092050246	1.741412933	-2.621950661
H	2.890717776	1.783901581	-0.869150872

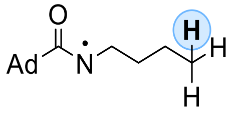

Conformer 6

C	-3.946397663	-1.043411096	2.018009049
C	-2.818192766	-1.684986426	1.205275182
C	-3.318751794	-1.991472191	-0.209572117
C	-3.769587334	-0.695352900	-0.889608206
C	-4.899177573	-0.059004043	-0.074243666
C	-4.398542040	0.253454000	1.339402826
H	-3.602004029	-0.833531717	3.042024983
H	-4.791982431	-1.742680431	2.101020559
H	-2.492359328	-2.615109728	1.692474582
H	-4.155965281	-2.704123722	-0.165401938
H	-2.520736927	-2.469276266	-0.797683911
H	-4.124491777	-0.915805988	-1.906529947
H	-5.760960763	-0.741930028	-0.028552671
H	-5.244860479	0.863429744	-0.564473410
H	-5.207206600	0.713656690	1.924641485
C	-3.218625341	1.224262935	1.257625580
H	-2.857562237	1.481233509	2.264560989
H	-3.518598290	2.169800006	0.785833939
C	-1.634510820	-0.717506142	1.123544243
H	-1.248500901	-0.501593016	2.133226309
H	-0.809976196	-1.168746526	0.549564255
C	-2.586047274	0.273453704	-0.971787741
H	-2.888865093	1.208695941	-1.467089537
H	-1.769388685	-0.160205866	-1.566012142
C	-2.079402934	0.593820024	0.449904887
C	-0.909119233	1.549379004	0.363901325
O	-0.961199127	2.733429655	0.657004604
N	0.246729179	0.997240360	-0.175851485
C	1.467807254	1.266380124	0.521657437
H	1.395038979	2.250355604	1.019531648
H	1.553801774	0.525727222	1.343962010
C	2.688407945	1.178779959	-0.375407855
H	2.589099421	1.917571003	-1.183925955
H	2.709549260	0.192922247	-0.862534542
C	3.980368697	1.415715489	0.392520040

H	3.930882633	2.394241511	0.894059821
H	4.066407144	0.670496016	1.198555915
C	5.207594077	1.354646410	-0.501232627
H	5.155727170	2.113412236	-1.291947592
H	6.132184346	1.523009298	0.061028855
H	5.288845941	0.376612847	-0.991874410

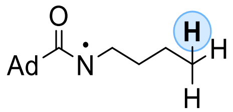

Conformer 7

C	-2.811839536	-2.351675964	-0.746701931
C	-2.869845753	-1.164184726	-1.712588264
C	-3.970507032	-0.196400759	-1.267284439
C	-3.671283023	0.307608789	0.148469785
C	-3.614700766	-0.882896241	1.111163959
C	-2.511590468	-1.849103591	0.668914868
H	-2.036050880	-3.062746597	-1.068267148
H	-3.769704448	-2.892874136	-0.758621729
H	-3.081073019	-1.524229466	-2.729607464
H	-4.947210957	-0.702761240	-1.290104968
H	-4.032113666	0.652165975	-1.965352562
H	-4.460747762	1.003027992	0.466662063
H	-4.584879534	-1.401937677	1.127680504
H	-3.420489870	-0.530745442	2.135300590
H	-2.464786098	-2.701540135	1.361604892
C	-1.162266842	-1.124984423	0.676420690
H	-0.353612546	-1.805002872	0.373405381
H	-0.925229536	-0.766400816	1.689803675
C	-1.521794558	-0.438717338	-1.705874073
H	-0.719022588	-1.117162817	-2.033925489
H	-1.540762362	0.408026818	-2.411068378
C	-2.323938130	1.034321316	0.160261568
H	-2.093200274	1.417124398	1.163869131
H	-2.349540575	1.908941424	-0.506856880
C	-1.214219738	0.079078503	-0.287613778
C	0.123140776	0.785084693	-0.282178804
O	0.335177253	1.855755682	0.270732172
N	1.157006612	0.082785117	-0.880120880
C	2.076734830	0.821280277	-1.695573233
H	1.871562726	0.523792513	-2.741504002
H	1.890741064	1.904252760	-1.626802137
C	3.521390178	0.471621289	-1.355402181
H	4.178481735	1.006287898	-2.057368741
H	3.668254797	-0.602517524	-1.529231702
C	3.896249061	0.821920425	0.081247238
H	3.241237074	0.259864891	0.763861515
H	4.916655354	0.463081710	0.272775394
C	3.802463506	2.308845353	0.390607872
H	2.762821766	2.655116797	0.366804669
H	4.196457563	2.530705581	1.387898374
H	4.379672978	2.901170343	-0.332669425

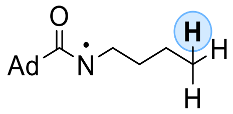

Conformer 8

C	-2.704560584	0.060889543	-2.168455697
C	-3.141577425	0.771649715	-0.884252658
C	-4.009598112	-0.176944069	-0.051404142
C	-3.203218588	-1.428874370	0.305918702
C	-2.761631547	-2.137441539	-0.977729169
C	-1.896699136	-1.191361882	-1.814865108
H	-2.098193483	0.738592425	-2.788359057
H	-3.586596422	-0.216630236	-2.765047505
H	-3.718334119	1.671922170	-1.139343426
H	-4.911097145	-0.458221597	-0.616405326
H	-4.348612415	0.328498818	0.865224379
H	-3.820784743	-2.108796422	0.910182873
H	-3.642461773	-2.452612718	-1.557043902
H	-2.195411580	-3.048157395	-0.731314198
H	-1.576341862	-1.698878757	-2.735936980
C	-0.659287587	-0.780335596	-1.012505586
H	-0.041624854	-0.097790418	-1.618027871
H	-0.037451669	-1.656147757	-0.770565487
C	-1.907951394	1.175776923	-0.073174481
H	-1.281907583	1.879442028	-0.642590693
H	-2.200284916	1.697960957	0.848064976
C	-1.965654765	-1.028986239	1.113182654
H	-1.378369988	-1.915608920	1.389445796
H	-2.263096208	-0.528744430	2.047887644
C	-1.087007555	-0.066722232	0.283923473
C	0.119194441	0.315444508	1.117672162
O	0.259707519	1.412888251	1.642755716
N	1.001058339	-0.734466929	1.363976010
C	2.363771744	-0.398075905	1.648313872
H	2.457241591	0.652654818	1.960078776
H	2.713334117	-1.049493533	2.459777579
C	3.246549738	-0.666884454	0.413796569
H	3.092125749	-1.707983572	0.099509176
H	4.292198921	-0.583434116	0.743207672
C	3.004958311	0.275145537	-0.758637014
H	3.659764244	-0.037415879	-1.583787267
H	1.978712510	0.142913938	-1.131746150
C	3.252610330	1.740799173	-0.434584041
H	4.247641996	1.882896896	0.007964574
H	3.201233708	2.359639920	-1.336654934
H	2.512940939	2.132520293	0.273786499

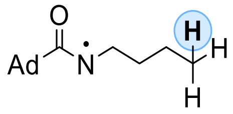

Conformer 9

C	-3.276211005	-0.924751134	1.752553308
C	-2.546441610	-1.737800596	0.679218811
C	-3.499524782	-2.014266392	-0.487984955

C	-3.981038743	-0.688164357	-1.084967797
C	-4.710518992	0.120760021	-0.008361630
C	-3.755697049	0.402163753	1.156055871
H	-2.605295398	-0.735555181	2.604270004
H	-4.132868515	-1.496653407	2.139770749
H	-2.196997130	-2.688569437	1.106602751
H	-4.360580728	-2.603468100	-0.138614304
H	-2.990569380	-2.612966047	-1.258298592
H	-4.661733620	-0.886637269	-1.925266912
H	-5.589251683	-0.436197293	0.350174830
H	-5.079194172	1.066805397	-0.431899746
H	-4.276148582	0.987036622	1.927703444
C	-2.553751429	1.199200493	0.646036946
H	-1.865490159	1.432717337	1.472662263
H	-2.871111355	2.162540917	0.224807443
C	-1.340789560	-0.941747527	0.170630668
H	-0.639347287	-0.748116880	0.998989374
H	-0.792877231	-1.517258599	-0.590787942
C	-2.776444736	0.107243600	-1.596559354
H	-3.104190169	1.061679160	-2.036176951
H	-2.247632231	-0.451106342	-2.382114624
C	-1.811256741	0.395668787	-0.426996398
C	-0.636231210	1.190925297	-0.955523536
O	-0.495959699	2.394661887	-0.791235185
N	0.235188579	0.468516373	-1.764445380
C	1.628116762	0.751339157	-1.590910732
H	2.188148010	0.392500213	-2.464727055
H	1.745619231	1.845085413	-1.511532942
C	2.169474513	0.096261298	-0.311457213
H	1.871351339	-0.961357895	-0.305679216
H	1.680863771	0.571442440	0.553714861
C	3.683267018	0.212898521	-0.187660307
H	4.000149372	-0.323414850	0.716936944
H	4.156922589	-0.312419947	-1.030496972
C	4.178046984	1.649755465	-0.129501393
H	3.664604427	2.210754374	0.661985170
H	5.253056868	1.694317289	0.073936624
H	4.004466011	2.181173667	-1.072851418

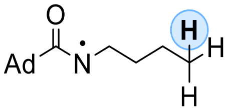

Conformer 10

C	-2.979575363	0.450622965	-1.974718541
C	-3.376945685	0.930466767	-0.575276598
C	-4.139358585	-0.183590790	0.148805720
C	-3.246392494	-1.422279475	0.268892891
C	-2.847868279	-1.900679887	-1.130300993
C	-2.086216258	-0.788357707	-1.858332103
H	-2.447630801	1.250783906	-2.511372666
H	-3.878891275	0.212551241	-2.562340869
H	-4.015558401	1.821341954	-0.657986992
H	-5.057515786	-0.433435895	-0.403772846
H	-4.447464653	0.157966095	1.148222259
H	-3.789332457	-2.221992755	0.792719281
H	-3.746192979	-2.177286636	-1.702220708
H	-2.221642540	-2.802527529	-1.057212460
H	-1.796091190	-1.132960388	-2.861078990
C	-0.825554894	-0.433596836	-1.063864280
H	-0.261715852	0.357122843	-1.585435661
H	-0.161069950	-1.307480272	-0.984677992
C	-2.120430540	1.284064108	0.221715220
H	-1.567781930	2.099991526	-0.269293775
H	-2.379766991	1.644960535	1.225898741
C	-1.985856902	-1.071159225	1.064780517
H	-1.336483969	-1.951604385	1.174137653
H	-2.251411647	-0.730929166	2.077371798
C	-1.214255926	0.053962630	0.341947894
C	0.012089523	0.391561007	1.163874665
O	0.076427527	1.339245127	1.932841543
N	1.043272699	-0.538627304	1.078607229
C	2.367882270	0.006695680	1.027374133
H	2.438779419	0.810396047	1.781473407
H	3.090179341	-0.779858376	1.273642005
C	2.669876944	0.615047373	-0.350065941
H	1.906590141	1.378969236	-0.559228416
H	2.557767317	-0.165632696	-1.117452809
C	4.059068835	1.237620320	-0.434945264
H	4.150600135	1.744646372	-1.404872780
H	4.148738567	2.026280650	0.326735743
C	5.194978063	0.236602637	-0.281401971
H	5.087010110	-0.592727049	-0.992450370
H	6.166759417	0.706420484	-0.465740953
H	5.231680421	-0.195726446	0.725406229

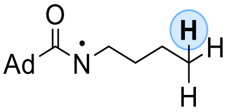

Conformer 11

C	-3.860084159	-0.993079866	-1.634838071
C	-4.098335163	-0.189162806	-0.352460288
C	-4.363396863	-1.151040455	0.810052505
C	-3.154185439	-2.071277551	1.000750558
C	-2.916741635	-2.874010982	-0.282190581
C	-2.651644222	-1.915898075	-1.447897499
H	-3.687255049	-0.311103679	-2.481153433
H	-4.752707370	-1.588464432	-1.879358270
H	-4.964631073	0.473582171	-0.488331794
H	-5.263936917	-1.750463662	0.608133223
H	-4.555037792	-0.583111646	1.732857329
H	-3.340080354	-2.759095523	1.838016661
H	-3.794823232	-3.499561694	-0.502145980
H	-2.061825530	-3.554219544	-0.150253603
H	-2.477985571	-2.491557607	-2.368276101
C	-1.410301766	-1.074970054	-1.137488610
H	-1.191645918	-0.392824762	-1.975446398
H	-0.530393383	-1.724148180	-1.008065879
C	-2.860189577	0.654906987	-0.040912052

H	-2.663436256	1.370314705	-0.853027967
H	-3.011374806	1.253534149	0.867938032
C	-1.913579013	-1.229368080	1.312173865
H	-1.035773132	-1.872345534	1.467089219
H	-2.064448658	-0.654236453	2.238557338
C	-1.643105579	-0.255224596	0.147600058
C	-0.412771563	0.574428632	0.446915780
O	-0.430046983	1.765594309	0.714265640
N	0.773588091	-0.150458534	0.481844547
C	1.905708993	0.435488647	-0.168209234
H	1.871953356	0.110402596	-1.228286726
H	1.798333972	1.535269562	-0.188406401
C	3.217744796	0.010075758	0.471375827
H	3.321175524	-1.081423766	0.381689395
H	3.158903806	0.227572243	1.545890284
C	4.428030339	0.707997303	-0.136789653
H	4.327035261	1.795172202	0.001217833
H	5.323000539	0.416350648	0.428497034
C	4.635818923	0.396067159	-1.611760762
H	3.825117825	0.794107005	-2.234457310
H	5.569416436	0.829125674	-1.986119015
H	4.680322287	-0.687609523	-1.782250386

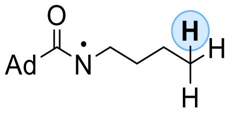

Conformer 12

C	-4.869926695	-0.022549460	-0.437440041
C	-4.069044593	-0.864018798	-1.436093388
C	-3.760433933	-0.022723768	-2.678951806
C	-2.944602127	1.210244322	-2.277524067
C	-3.744773684	2.047815262	-1.274907066
C	-4.053080981	1.207784915	-0.031717908
H	-5.114016494	-0.623467205	0.451425439
H	-5.824611207	0.291046886	-0.886055050
H	-4.653238047	-1.748861063	-1.725597422
H	-4.696202733	0.289298995	-3.166739805
H	-3.201165947	-0.623653529	-3.411972666
H	-2.720355432	1.813565015	-3.168782878
H	-4.681900031	2.392906612	-1.736774778
H	-3.174943875	2.946080939	-0.993108541
H	-4.623969023	1.810743934	0.688892510
C	-2.741507252	0.760145878	0.619715534
H	-2.946882925	0.163202849	1.521456240
H	-2.145547552	1.628865548	0.932935805
C	-2.758749748	-1.314083730	-0.785550668
H	-2.952483418	-1.932890331	0.101439522
H	-2.176327641	-1.939910040	-1.477717245
C	-1.632351324	0.764301375	-1.627466385
H	-1.030270941	1.640432864	-1.340053211
H	-1.032507126	0.176543419	-2.341596065
C	-1.931613446	-0.090623803	-0.379904429
C	-0.628188943	-0.527957049	0.253224185
O	-0.368756087	-1.674836925	0.580250711
N	0.257003350	0.510725609	0.522955544
C	1.625553059	0.297393878	0.159797315
H	1.850656047	-0.781066996	0.221730275
H	1.730060364	0.561882182	-0.914791332
C	2.579229322	1.144259422	0.985602465
H	2.524778630	0.813536759	2.033290114
H	2.217935790	2.181127130	0.970012409
C	4.019476057	1.077755988	0.492416471
H	4.064774843	1.436737819	-0.547682788
H	4.625737913	1.781389082	1.077953510
C	4.631357442	-0.313242405	0.582121850
H	4.133871686	-1.025938448	-0.086349665
H	5.692427956	-0.302963990	0.311804185
H	4.551503223	-0.711967867	1.601672417

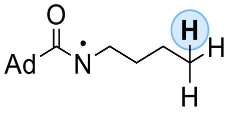

Conformer 13

C	-3.890443462	-0.740844605	-0.084174407
C	-3.682760344	0.461259790	0.842799886
C	-3.336370181	-0.035808419	2.250085163
C	-2.053721369	-0.870826218	2.200451242
C	-2.258701657	-2.068442185	1.267615672
C	-2.606499830	-1.573791375	-0.139825308
H	-4.161683270	-0.396859544	-1.093379890
H	-4.724313109	-1.359733663	0.280106759
H	-4.602323999	1.062367190	0.879767790
H	-4.162712290	-0.641429298	2.651509260
H	-3.204716078	0.818738427	2.931056326
H	-1.801376099	-1.226553873	3.209591220
H	-3.066311293	-2.709505696	1.651529917
H	-1.346970533	-2.683998977	1.237541316
H	-2.750168261	-2.434184793	-0.808909737
C	-1.458389461	-0.712436347	-0.673244854
H	-1.685009688	-0.351602229	-1.688137124
H	-0.530036720	-1.297988622	-0.734037671
C	-2.537297742	1.324355852	0.307763455
H	-2.766165679	1.703395983	-0.697228386
H	-2.386902653	2.207207550	0.947003782
C	-0.905389372	-0.009445946	1.668532093
H	0.027489934	-0.593761733	1.638921636
H	-0.729272122	0.848485093	2.338123438
C	-1.245541172	0.500538471	0.257128634
C	-0.108747064	1.337385734	-0.290399710
O	-0.258517950	2.372799691	-0.923492815
N	1.149246047	0.777751986	-0.123439100
C	2.188658043	1.622621303	0.391126555
H	1.924726201	2.688198134	0.264700245
H	2.219086004	1.431030167	1.480748164
C	3.553559146	1.312664905	-0.213063297
H	3.668298847	0.219864645	-0.257136479
H	4.328825056	1.685184405	0.470389789
C	3.762175098	1.920137021	-1.596532166

H	3.718182973	3.016964461	-1.513389425
H	4.783327584	1.685380157	-1.927769682
C	2.758324045	1.450601839	-2.638908014
H	1.747306759	1.809145276	-2.411294985
H	3.021028066	1.821565249	-3.635812607
H	2.720757183	0.354815410	-2.686401671

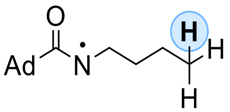

Conformer 14

C	-2.957252008	-1.662027349	-0.413782005
C	-2.629929464	-1.148274375	-1.819150322
C	-3.287188797	0.219531344	-2.028077020
C	-2.756602860	1.208339724	-0.985821766
C	-3.082672310	0.692502481	0.419103119
C	-2.425098291	-0.675107767	0.629381009
H	-2.506457716	-2.654169060	-0.260420608
H	-4.045069636	-1.781564900	-0.298192162
H	-3.007665366	-1.857617823	-2.569217265
H	-4.380672590	0.131372604	-1.941069521
H	-3.074192688	0.589251068	-3.042536436
H	-3.222674749	2.192641986	-1.136427801
H	-4.172549311	0.609276136	0.545642094
H	-2.723374780	1.405373778	1.176356360
H	-2.652844667	-1.043701323	1.639841085
C	-0.907086636	-0.536272864	0.479460788
H	-0.417168002	-1.509199002	0.646482249
H	-0.508640433	0.162378812	1.228658401
C	-1.113672046	-1.014063117	-1.970915623
H	-0.621883772	-1.989343694	-1.844140660
H	-0.852683495	-0.667571094	-2.981477853
C	-1.238695046	1.346058233	-1.137331397
H	-0.838649857	2.061889851	-0.405371381
H	-0.990426495	1.730719645	-2.139201815
C	-0.565542964	-0.027451895	-0.935549755
C	0.933165777	0.119243656	-1.089589215
O	1.583133302	-0.506171911	-1.919187641
N	1.514216227	1.035205751	-0.197826324
C	2.915859608	1.240669060	-0.399624445
H	3.161236862	1.242277502	-1.472248923
H	3.187656677	2.207658628	0.046154218
C	3.783007410	0.139673749	0.258300776
H	4.802953133	0.290969780	-0.119075299
H	3.449623229	-0.835027484	-0.122884746
C	3.794051082	0.151461661	1.779374132
H	4.566596115	-0.554551021	2.116662858
H	4.119152619	1.141895765	2.132462579
C	2.463404430	-0.214333393	2.415951953
H	2.102460967	-1.180662608	2.037407904
H	2.546024022	-0.293271876	3.505481699
H	1.695243491	0.533586739	2.185821522

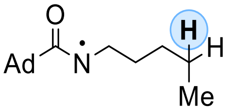

Conformer 1

C	-2.196381456	1.452059079	-2.235161464
C	-2.115004214	0.666971777	-0.921908168
C	-2.695077608	-0.735495565	-1.128042730
C	-1.896292201	-1.466419379	-2.211576976
C	-1.979870117	-0.680172015	-3.523908207
C	-1.399067744	0.722415391	-3.320907342
H	-1.799680835	2.468552522	-2.093036558
H	-3.246517277	1.556855278	-2.546253210
H	-2.682322306	1.192971584	-0.140786451
H	-3.753615238	-0.665674099	-1.420369799
H	-2.658730439	-1.301772526	-0.184981645
H	-2.309172675	-2.474438061	-2.358563707
H	-3.026823151	-0.609254571	-3.855123586
H	-1.426250269	-1.207084524	-4.315303215
H	-1.453360604	1.286934167	-4.262771194
C	0.065527561	0.611104793	-2.889763797
H	0.507205359	1.608860735	-2.751085635
H	0.658306010	0.093840323	-3.659359334
C	-0.649291939	0.556956476	-0.489917801
H	-0.224598008	1.559084022	-0.327683413
H	-0.577150953	0.013058984	0.467777701
C	-0.432230927	-1.579767867	-1.781097715
H	0.160410442	-2.113833381	-2.535246157
H	-0.348052012	-2.158682713	-0.847656444
C	0.160452477	-0.181780869	-1.567807650
C	1.627510257	-0.295734847	-1.205708146
O	2.334168465	-1.227951870	-1.563548579
N	2.168554280	0.805364225	-0.553702362
C	3.137939049	0.507972784	0.464006003
H	3.813168869	-0.264044540	0.059418347
H	3.719193880	1.411932764	0.685502085
C	2.482103726	-0.029746449	1.740585012
H	3.273519584	-0.381745554	2.417700561
H	1.884397722	-0.918593706	1.479423631
C	1.614422061	0.999486518	2.449750459
H	2.241138014	1.846069229	2.773805909
H	0.886652804	1.423346934	1.739597697
C	0.874451484	0.419983647	3.645563599
H	1.601393479	-0.016236071	4.347941980
H	0.247621461	-0.419541826	3.305289714
C	0.014657646	1.451421250	4.357955191
H	-0.737046033	1.872497583	3.678009268
H	-0.515834864	1.022598583	5.214815498
H	0.623580292	2.285990202	4.727615102

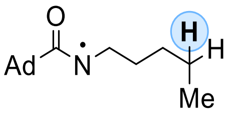

Conformer 2

C	-2.196381456	1.452059079	-2.235161464
C	-2.115004214	0.666971777	-0.921908168
C	-2.695077608	-0.735495565	-1.128042730
C	-1.896292201	-1.466419379	-2.211576976
C	-1.979870117	-0.680172015	-3.523908207
C	-1.399067744	0.722415391	-3.320907342
H	-1.799680835	2.468552522	-2.093036558
H	-3.246517277	1.556855278	-2.546253210
H	-2.682322306	1.192971584	-0.140786451
H	-3.753615238	-0.665674099	-1.420369799
H	-2.658730439	-1.301772526	-0.184981645
H	-2.309172675	-2.474438061	-2.358563707
H	-3.026823151	-0.609254571	-3.855123586
H	-1.426250269	-1.207084524	-4.315303215
H	-1.453360604	1.286934167	-4.262771194
C	0.065527561	0.611104793	-2.889763797
H	0.507205359	1.608860735	-2.751085635
H	0.658306010	0.093840323	-3.659359334
C	-0.649291939	0.556956476	-0.489917801
H	-0.224598008	1.559084022	-0.327683413
H	-0.577150953	0.013058984	0.467777701
C	-0.432230927	-1.579767867	-1.781097715
H	0.160410442	-2.113833381	-2.535246157
H	-0.348052012	-2.158682713	-0.847656444
C	0.160452477	-0.181780869	-1.567807650
C	1.627510257	-0.295734847	-1.205708146
O	2.334168465	-1.227951870	-1.563548579
N	2.168554280	0.805364225	-0.553702362
C	3.137939049	0.507972784	0.464006003
H	3.813168869	-0.264044540	0.059418347
H	3.719193880	1.411932764	0.685502085
C	2.482103726	-0.029746449	1.740585012
H	3.273519584	-0.381745554	2.417700561
H	1.884397722	-0.918593706	1.479423631
C	1.614422061	0.999486518	2.449750459
H	2.241138014	1.846069229	2.773805909
H	0.886652804	1.423346934	1.739597697
C	0.874451484	0.419983647	3.645563599
H	1.601393479	-0.016236071	4.347941980
H	0.247621461	-0.419541826	3.305289714
C	0.014657646	1.451421250	4.357955191
H	-0.737046033	1.872497583	3.678009268
H	-0.515834864	1.022598583	5.214815498
H	0.623580292	2.285990202	4.727615102

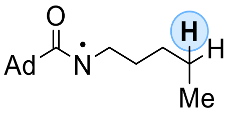

Conformer 3

C	-3.509434461	0.641666372	-2.539684441
C	-2.578797055	1.755809163	-2.050560637
C	-3.073086651	2.277578627	-0.697665261
C	-3.085850416	1.134038500	0.321961115
C	-4.018280015	0.023066296	-0.170237995
C	-3.522588225	-0.502856380	-1.520998887
H	-3.172505557	0.271224775	-3.519690989
H	-4.526998132	1.036871244	-2.678307818
H	-2.565629040	2.576696285	-2.781743478
H	-4.084141263	2.698164806	-0.804567517
H	-2.421308608	3.091206219	-0.345403484
H	-3.436423944	1.509302411	1.293994753
H	-5.044322620	0.408715350	-0.267647174
H	-4.050680658	-0.795865852	0.563958725
H	-4.189374108	-1.302490160	-1.873384444
C	-2.106630681	-1.060998099	-1.359765967
H	-1.738334592	-1.466688332	-2.313782277
H	-2.091822950	-1.893617871	-0.643093635
C	-1.160900426	1.200955795	-1.891645628
H	-0.782607886	0.838901714	-2.861267485
H	-0.475020227	1.992212198	-1.550210212
C	-1.667251426	0.579902399	0.482102592
H	-1.654767932	-0.236528009	1.220515089
H	-0.984130340	1.359463672	0.847578748
C	-1.163733160	0.043926238	-0.874291539
C	0.240986710	-0.493442405	-0.718209413
O	0.534948924	-1.680986369	-0.731115263
N	1.209190977	0.461766067	-0.454667300
C	2.477859347	0.318676813	-1.108937830
H	2.518918915	-0.604134431	-1.710855720
H	2.568732815	1.175815204	-1.800021849
C	3.622387520	0.358682428	-0.101723741
H	3.561423392	1.295483617	0.471323055
H	4.574163999	0.382400705	-0.652997961
C	3.595543707	-0.831812343	0.843481396
H	2.634144481	-0.843462756	1.381628250
H	3.613246349	-1.766265603	0.259312804
C	4.744475270	-0.822227038	1.840852747
H	4.705007132	0.109658285	2.425777321
H	5.699267912	-0.786717187	1.292839292
C	4.721595118	-2.024008812	2.771116998
H	3.779267617	-2.068916934	3.331649368
H	4.806513751	-2.960917195	2.206489524
H	5.540286543	-1.998665587	3.498221059

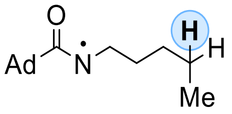

Conformer 4

C	-3.357163108	-1.961833433	-0.432207485
C	-2.381749688	-1.227874226	-1.357774664
C	-2.997317953	0.104816306	-1.794705302
C	-3.282883497	0.968832908	-0.562615705
C	-4.259360829	0.232469066	0.360008346

C	-3.644473675	-1.099290238	0.800948136
H	-2.932319476	-2.929665974	-0.126191549
H	-4.293974106	-2.176162371	-0.968035419
H	-2.172385810	-1.847470900	-2.241430705
H	-3.927873040	-0.075856576	-2.353630169
H	-2.311693291	0.632202918	-2.475239040
H	-3.722506411	1.926025556	-0.876863572
H	-5.210655288	0.053188588	-0.163333505
H	-4.487543724	0.852737759	1.239671705
H	-4.340778629	-1.627528369	1.467947197
C	-2.336679805	-0.835028825	1.552206842
H	-1.884441898	-1.778591868	1.889529933
H	-2.522997842	-0.222695679	2.447829546
C	-1.072915985	-0.961504954	-0.608019362
H	-0.606739715	-1.909717806	-0.298849790
H	-0.356934321	-0.448378997	-1.270827147
C	-1.977522785	1.236263617	0.189503180
H	-2.153201531	1.865998514	1.071932109
H	-1.268182933	1.785361390	-0.448818205
C	-1.348289019	-0.089348966	0.629372961
C	-0.073848855	0.171820668	1.405391524
O	0.197941707	1.243092756	1.927951919
N	0.731722776	-0.944893702	1.594713554
C	2.142866876	-0.723499253	1.478942887
H	2.392826219	0.188928736	2.048548980
H	2.683985777	-1.571471261	1.917288534
C	2.570757916	-0.516139721	0.021805788
H	2.060367292	0.377073920	-0.372375723
H	2.224525816	-1.368820904	-0.581726850
C	4.076409785	-0.357171273	-0.117958506
H	4.417233037	0.484724106	0.505880592
H	4.581041800	-1.251513814	0.281508820
C	4.511156244	-0.131613400	-1.559238032
H	4.006398679	0.763946578	-1.950929935
H	4.158100585	-0.969246035	-2.179651028
C	6.017267011	0.019625835	-1.700084985
H	6.385792920	0.863231468	-1.103047962
H	6.538274297	-0.880163971	-1.349345383
H	6.316464152	0.192693752	-2.739625060

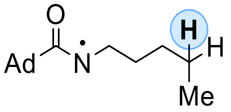

Conformer 5

C	-3.205523116	0.640109345	-0.221820957
C	-3.180184211	0.221349823	1.251278180
C	-3.892505616	-1.125038647	1.409822215
C	-3.180750866	-2.185004166	0.563313464
C	-3.207588055	-1.764638560	-0.909359001
C	-2.495243461	-0.418515120	-1.071101645
H	-2.713547208	1.616508434	-0.346201488
H	-4.245517636	0.758387086	-0.560908351
H	-3.685837483	0.983475280	1.861446504
H	-4.943255375	-1.033811331	1.096020265
H	-3.897019916	-1.427669679	2.467568342
H	-3.688233635	-3.152891931	0.680843422
H	-4.247074895	-1.684749580	-1.261462924
H	-2.716032109	-2.529380560	-1.529704284
H	-2.508430266	-0.115255400	-2.127656949
C	-1.042178867	-0.547222903	-0.603758275
H	-0.518102474	0.413343718	-0.723676295
H	-0.509530523	-1.289774180	-1.221079321
C	-1.728208337	0.090855022	1.718781182
H	-1.201754694	1.051884229	1.626314167
H	-1.688138351	-0.204818027	2.778192457
C	-1.730217959	-2.316983193	1.031889733
H	-1.206181905	-3.091061610	0.450073963
H	-1.683134875	-2.631405430	2.082690411
C	-1.002363584	-0.977514669	0.871204245
C	0.416431021	-1.092571836	1.389268341
O	0.759419761	-1.922389197	2.220992242
N	1.285534762	-0.089602974	0.975620462
C	2.654323890	-0.479395265	0.794619731
H	3.300369105	0.391773075	0.958884285
H	2.900944194	-1.256234184	1.535593819
C	2.897288297	-1.055477949	-0.609488394
H	2.312313823	-1.981922725	-0.710033837
H	3.956185304	-1.347713106	-0.657864781
C	2.574390248	-0.100942035	-1.753051448
H	2.835483618	-0.594000597	-2.701848155
H	1.488774200	0.078106186	-1.795091845
C	3.293224310	1.239841182	-1.674340480
H	4.369684931	1.069131223	-1.512878963
H	2.936018023	1.795588505	-0.795108029
C	3.080546417	2.078273990	-2.924062641
H	3.466986856	1.567278387	-3.815048153
H	2.012311023	2.265142380	-3.093694124
H	3.579329742	3.050797070	-2.856276203

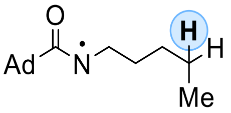

Conformer 6

C	-3.997922609	-1.019531140	1.999457326
C	-2.849482309	-1.674964038	1.226345357
C	-3.310301348	-1.998009802	-0.198324196
C	-3.736732911	-0.708145698	-0.906505678
C	-4.885915028	-0.057160211	-0.131183687
C	-4.424164066	0.271068131	1.292236804
H	-3.681316000	-0.798807856	3.030111867
H	-4.849133920	-1.713604928	2.066478382
H	-2.539707009	-2.599621822	1.734051845
H	-4.150875575	-2.707349471	-0.169125586
H	-2.496990145	-2.485223766	-0.757045056
H	-4.063426031	-0.939270621	-1.930429343
H	-5.751418916	-0.736108019	-0.101182216
H	-5.213858858	0.860457940	-0.642156684
H	-5.247130110	0.741993967	1.848255193

C	-3.238621217	1.237184584	1.232138504
H	-2.904728362	1.505318049	2.245578707
H	-3.522573449	2.177969161	0.741042113
C	-1.661171929	-0.711746214	1.166294772
H	-1.302756706	-0.485010289	2.183839215
H	-0.821780597	-1.172279861	0.621870646
C	-2.548433641	0.255809460	-0.966988040
H	-2.833868866	1.185606801	-1.482448366
H	-1.716709545	-0.187362325	-1.532333577
C	-2.080522661	0.593519200	0.464165300
C	-0.901759781	1.542213432	0.398249621
O	-0.954314301	2.726071336	0.694184505
N	0.257077886	0.984230108	-0.126641223
C	1.481266325	1.282610474	0.552162120
H	1.409704059	2.288212818	1.005796552
H	1.571476906	0.584587990	1.410608482
C	2.693746866	1.151622214	-0.350744248
H	2.580166694	1.845337935	-1.196410867
H	2.709918580	0.141247144	-0.785135277
C	3.997707508	1.430907785	0.380320902
H	3.968163876	2.441930515	0.818952464
H	4.100361039	0.739613293	1.233744576
C	5.219887939	1.305843166	-0.517653845
H	5.111223611	1.990015555	-1.372615820
H	5.250816552	0.292316158	-0.945451316
C	6.517471909	1.599906305	0.216451668
H	6.661183196	0.908882094	1.056853536
H	7.389194413	1.509383265	-0.440408266
H	6.515140197	2.617472968	0.627130619

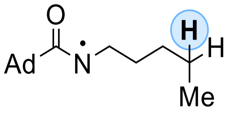

Conformer 7

C	-2.836540815	-2.333482398	-0.765159473
C	-2.887905385	-1.135609375	-1.718599509
C	-3.974983932	-0.160032816	-1.256785448
C	-3.662401572	0.325381147	0.162445836
C	-3.612896569	-0.875655009	1.111948659
C	-2.523109479	-1.849222511	0.654184675
H	-2.070185010	-3.049302064	-1.098717723
H	-3.800113800	-2.864494238	-0.777399255
H	-3.109310493	-1.483112479	-2.737799106
H	-4.957539993	-0.654819237	-1.279824653
H	-4.030501730	0.696731591	-1.945324085
H	-4.442255671	1.025687255	0.493357266
H	-4.588454724	-1.384604341	1.129136248
H	-3.409345665	-0.536309579	2.138627182
H	-2.482249059	-2.709203637	1.337787186
C	-1.166465416	-1.139719362	0.661817387
H	-0.367044776	-1.825864723	0.348292220
H	-0.919179128	-0.792178097	1.676610405
C	-1.531285599	-0.425656479	-1.712196177
H	-0.738615844	-1.110397453	-2.052339239
H	-1.544635861	0.427721228	-2.409398201
C	-2.307487262	1.036935879	0.173474290
H	-2.065409072	1.405919174	1.179463488
H	-2.327077923	1.918621231	-0.484477145
C	-1.210980206	0.073699081	-0.291166426
C	0.134662509	0.762553646	-0.279381131
O	0.362565152	1.822116033	0.289854544
N	1.160883872	0.054809336	-0.879151289
C	2.088810186	0.779129313	-1.699456832
H	1.896592512	0.461870449	-2.740627263
H	1.902766374	1.863932005	-1.652417875
C	3.528940904	0.436000244	-1.329011871
H	4.199072315	0.945702330	-2.037262227
H	3.673845907	-0.643441292	-1.468781531
C	3.883221267	0.826378235	0.100777858
H	3.194446078	0.314988945	0.792834943
H	4.888400200	0.444508641	0.335917292
C	3.841056804	2.325922024	0.370513367
H	2.814072532	2.692779975	0.235691748
H	4.463944270	2.846636699	-0.374779195
C	4.313074014	2.663030356	1.774748190
H	3.677892883	2.174145753	2.524412070
H	5.341719317	2.319161665	1.944299944
H	4.285604110	3.740359918	1.970915992

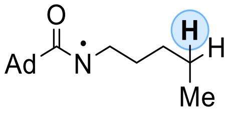

Conformer 8

C	-2.473972075	0.307896870	-2.180060167
C	-2.942682960	0.950088287	-0.871121846
C	-3.912054663	0.001752487	-0.158322633
C	-3.208384741	-1.323601098	0.146631631
C	-2.736980430	-1.963480101	-1.162476504
C	-1.767323880	-1.016544911	-1.875973274
H	-1.789202497	0.987928189	-2.710148669
H	-3.333172848	0.133880274	-2.845137531
H	-3.449136966	1.901601071	-1.086107529
H	-4.795226467	-0.178632496	-0.789467157
H	-4.271752204	0.461279073	0.774375782
H	-3.902468984	-2.002422805	0.662646911
H	-3.600790951	-2.175869487	-1.810055118
H	-2.243633643	-2.925430383	-0.957411299
H	-1.422452529	-1.477046724	-2.812591188
C	-0.558430921	-0.749546358	-0.974028770
H	0.142664433	-0.072369305	-1.488442543
H	-0.015137750	-1.683313983	-0.766285531
C	-1.737107074	1.213922887	0.033902877
H	-1.032407533	1.908322911	-0.453530657
H	-2.047082302	1.688537546	0.973873575
C	-2.000840119	-1.062926690	1.049900845
H	-1.484109705	-2.004052729	1.289982069
H	-2.317764271	-0.609414234	2.001152430

C	-1.016253081	-0.104561042	0.342362893
C	0.126072497	0.149362168	1.303615934
O	0.095234331	1.036845879	2.149102979
N	1.151692570	-0.782477224	1.295356694
C	2.453561977	-0.325794211	1.691044434
H	2.429532606	0.725503398	2.013978799
H	2.778951648	-0.939125166	2.544295782
C	3.468558229	-0.531131515	0.556369028
H	3.601122051	-1.607108965	0.385602276
H	4.431272417	-0.143271851	0.919108175
C	3.086713556	0.149128204	-0.752820464
H	3.910837466	0.020977996	-1.470453558
H	2.226887034	-0.377952365	-1.193122267
C	2.757248734	1.631514002	-0.635989361
H	3.606307952	2.163022357	-0.179335665
H	1.907749790	1.782984354	0.047654565
C	2.420932640	2.246839282	-1.985622479
H	3.253518047	2.138445613	-2.692184547
H	1.546612628	1.754271037	-2.434087323
H	2.191001913	3.313921716	-1.901299498

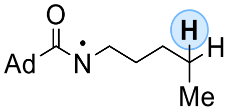

Conformer 9

C	-3.279540861	-0.908938527	1.757343360
C	-2.564754123	-1.734443514	0.682999853
C	-3.526207886	-2.005151107	-0.478353817
C	-3.994396105	-0.675951863	-1.079111214
C	-4.707794108	0.146751682	-0.002450902
C	-3.745870531	0.421465024	1.157679091
H	-2.601949903	-0.724663326	2.604790127
H	-4.141099226	-1.469203959	2.150733128
H	-2.224766389	-2.687740078	1.112152909
H	-4.392121905	-2.583204317	-0.122338261
H	-3.027220794	-2.613176058	-1.247886124
H	-4.681669814	-0.869508352	-1.915084268
H	-5.592979111	-0.396500487	0.361344846
H	-5.065554184	1.096183947	-0.428113942
H	-4.257364716	1.015029625	1.928651363
C	-2.536673426	1.202935054	0.639058202
H	-1.841290799	1.431826537	1.460973827
H	-2.844601834	2.168309579	0.215275098
C	-1.352196352	-0.955037097	0.165861823
H	-0.643501645	-0.769598081	0.989737769
H	-0.816776200	-1.540204865	-0.597865035
C	-2.782544168	0.102342028	-1.598454478
H	-3.099778253	1.058068016	-2.043150470
H	-2.261870291	-0.465641061	-2.382357032
C	-1.809667380	0.386596505	-0.433622873
C	-0.626856062	1.164675449	-0.971893363
O	-0.481331923	2.370650289	-0.829880363
N	0.245552547	0.421652915	-1.758972133
C	1.637535515	0.708773539	-1.589153790
H	2.202469691	0.325846773	-2.449511431
H	1.754237875	1.805014609	-1.542414772
C	2.177220994	0.094795169	-0.288755512
H	1.888280253	-0.964727716	-0.253875600
H	1.679799376	0.589069497	0.560520885
C	3.687898226	0.229237700	-0.159895984
H	4.008898519	-0.275675466	0.763717987
H	4.178321839	-0.314645681	-0.983630990
C	4.187366980	1.668849344	-0.137768131
H	3.617314708	2.236784628	0.614013006
H	3.973297049	2.152789455	-1.101937021
C	5.676614058	1.755360556	0.155322961
H	5.910290693	1.331444641	1.139893982
H	6.257035538	1.193798741	-0.587723371
H	6.037981750	2.788950102	0.144840208

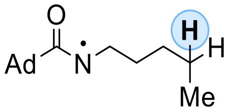

Conformer 10

C	-2.991339243	0.452292774	-1.964057927
C	-3.402986172	0.902274439	-0.558815672
C	-4.138740380	-0.241962507	0.146116633
C	-3.215963535	-1.460043746	0.248793147
C	-2.802941886	-1.908282146	-1.156753427
C	-2.068139154	-0.766166015	-1.865813117
H	-2.478596562	1.273208437	-2.488107008
H	-3.883615626	0.201071783	-2.557111915
H	-4.063786914	1.777805922	-0.630348728
H	-5.049647619	-0.505828171	-0.412062915
H	-4.456916454	0.076170569	1.150131306
H	-3.739337136	-2.280725198	0.759911895
H	-3.692058284	-2.199725134	-1.735751378
H	-2.153658951	-2.794594369	-1.095219372
H	-1.768578672	-1.087592676	-2.873530470
C	-0.817868940	-0.391925140	-1.064346296
H	-0.271900144	0.418769997	-1.574536635
H	-0.133012081	-1.251287010	-0.995915177
C	-2.155739815	1.275677677	0.245471341
H	-1.622762922	2.112366407	-0.232012232
H	-2.424694624	1.614082785	1.255027696
C	-1.965966871	-1.088717079	1.051085873
H	-1.294482403	-1.953755217	1.147740669
H	-2.240137719	-0.771507664	2.068892868
C	-1.220951608	0.066600323	0.347713569
C	-0.001284181	0.418161306	1.173974363
O	0.047202220	1.361882969	1.949915720
N	1.041699917	-0.495650064	1.086145083
C	2.363056641	0.057851125	1.052881328
H	2.426451709	0.843885830	1.826206707
H	3.087695752	-0.730050794	1.288696109
C	2.671129712	0.698425265	-0.307810291
H	1.939943784	1.502206921	-0.477898778
H	2.510204629	-0.049301933	-1.099118734

C	4.087100447	1.252686780	-0.397232913
H	4.179366572	1.834763978	-1.326382601
H	4.255348459	1.969329316	0.422624404
C	5.179932034	0.190602158	-0.375867534
H	4.975253444	-0.552441578	-1.162021088
H	5.153468163	-0.361736295	0.574609262
C	6.562571700	0.789360624	-0.572897103
H	6.788733098	1.521891623	0.212308026
H	7.347863991	0.026515242	-0.551887172
H	6.633034305	1.312959135	-1.534863893

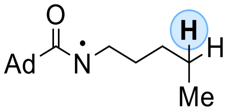

Conformer 11

C	-3.810054081	-1.014164221	-1.667814438
C	-4.090385719	-0.219934353	-0.387857041
C	-4.359724365	-1.191354972	0.765655572
C	-3.135707228	-2.086734347	0.979464850
C	-2.855990005	-2.880285298	-0.300955925
C	-2.587751864	-1.913192843	-1.458369029
H	-3.632995145	-0.325922246	-2.508194097
H	-4.685015250	-1.626489862	-1.932967233
H	-4.966981739	0.425698125	-0.539350971
H	-5.242766856	-1.808897496	0.542169344
H	-4.583454917	-0.630863835	1.685663524
H	-3.324112351	-2.781044537	1.810709701
H	-3.716037583	-3.523155008	-0.541089585
H	-1.989783550	-3.542834813	-0.153760287
H	-2.382937511	-2.482117378	-2.376506126
C	-1.371240381	-1.047143503	-1.120992451
H	-1.151415279	-0.358043217	-1.952938298
H	-0.479784941	-1.677988359	-0.977471639
C	-2.876655717	0.648536124	-0.048064420
H	-2.678345152	1.371112335	-0.853609324
H	-3.058040257	1.239672486	0.859981105
C	-1.918645072	-1.220948290	1.317668643
H	-1.031563909	-1.847044290	1.487738990
H	-2.097188934	-0.651041722	2.242366576
C	-1.645181748	-0.237200720	0.160909494
C	-0.434530873	0.610382914	0.491632821
O	-0.478683361	1.797231931	0.777257511
N	0.760702962	-0.095198311	0.532887418
C	1.884327848	0.497292157	-0.124400664
H	1.838151426	0.178962972	-1.185729446
H	1.776089806	1.597598650	-0.140208860
C	3.203487242	0.068106871	0.497535607
H	3.283713890	-1.027501866	0.441112151
H	3.173487039	0.320206600	1.565412115
C	4.413668375	0.716880315	-0.160386693
H	4.334885979	1.814285061	-0.085183070
H	5.316561441	0.444684842	0.406697843
C	4.618850334	0.324301972	-1.619830601
H	3.755938776	0.654707742	-2.218366540
H	4.621433964	-0.775331396	-1.691646247
C	5.896302097	0.899092914	-2.203541059
H	6.774778044	0.554372409	-1.642848607
H	6.036980840	0.615382227	-3.251429741
H	5.894273185	1.995400231	-2.154169349

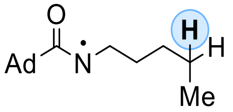

Conformer 12

C	-4.865810613	-0.071622710	-0.443455469
C	-4.049418110	-0.861886143	-1.470992776
C	-3.758939803	0.027337109	-2.684404055
C	-2.967670882	1.261537666	-2.240551526
C	-3.783188202	2.048152519	-1.209535350
C	-4.073265750	1.159967400	0.004699496
H	-5.097233659	-0.708153770	0.423683910
H	-5.826841718	0.238158964	-0.880951666
H	-4.615890180	-1.748014512	-1.790416026
H	-4.701102901	0.337095047	-3.161032858
H	-3.188171896	-0.536488154	-3.437737881
H	-2.756142641	1.899974688	-3.110157601
H	-4.727074633	2.389981508	-1.659860704
H	-3.231111386	2.947342375	-0.897173410
H	-4.654767029	1.726076222	0.746304639
C	-2.751352390	0.717033020	0.638553609
H	-2.940245096	0.085124789	1.519911239
H	-2.172779903	1.587027711	0.979642031
C	-2.730419316	-1.308107064	-0.836421343
H	-2.910937500	-1.959681970	0.029380653
H	-2.136715059	-1.898709852	-1.549983296
C	-1.645734455	0.818488827	-1.607825821
H	-1.058715913	1.695278542	-1.292292329
H	-1.037175695	0.267466309	-2.343519097
C	-1.926180685	-0.083447229	-0.390678878
C	-0.619034193	-0.515999772	0.238974384
O	-0.365965847	-1.661738914	0.578860729
N	0.267298409	0.522068594	0.496436158
C	1.635925151	0.293885308	0.145301208
H	1.855318211	-0.782427965	0.258621168
H	1.747054975	0.500697273	-0.940535674
C	2.590824048	1.173109227	0.934509032
H	2.522046952	0.898904339	1.997486857
H	2.242903699	2.212037016	0.861836317
C	4.033244031	1.064600250	0.459613231
H	4.099763847	1.379205381	-0.595741248
H	4.651695362	1.780467154	1.021245707
C	4.637408734	-0.329121802	0.604159243
H	4.090437202	-1.041552360	-0.031238938
H	4.487143712	-0.678521813	1.637865245
C	6.112219245	-0.357594099	0.246157836
H	6.688674192	0.314537240	0.894464929
H	6.542263480	-1.360512255	0.340559967
H	6.271748790	-0.025525882	-0.787707194

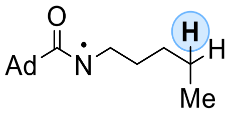

Conformer 13

C	-3.891067893	-0.746176779	-0.081838406
C	-3.684902644	0.441265440	0.863922024
C	-3.324476090	-0.076744318	2.260215413
C	-2.036275818	-0.902315677	2.186921058
C	-2.241076299	-2.086455630	1.236579595
C	-2.602455837	-1.570042568	-0.159832996
H	-4.170358758	-0.386483813	-1.083422151
H	-4.718960008	-1.376162152	0.276660620
H	-4.608163597	1.035188276	0.917914978
H	-4.143881356	-0.694255885	2.657715269
H	-3.192939826	0.767531091	2.953873898
H	-1.774093399	-1.272464203	3.188240981
H	-3.042188650	-2.738153689	1.616013214
H	-1.325833396	-2.695558796	1.188993533
H	-2.745270320	-2.419491886	-0.842882615
C	-1.464597335	-0.691790398	-0.686999893
H	-1.701391594	-0.316447073	-1.694318232
H	-0.531928314	-1.268536702	-0.764574422
C	-2.549680994	1.321251024	0.334710388
H	-2.788776234	1.715678735	-0.662064347
H	-2.400671953	2.194547808	0.987470469
C	-0.898164508	-0.023698566	1.660754918
H	0.038804836	-0.599978028	1.612554765
H	-0.723212668	0.823404134	2.344260559
C	-1.253404994	0.508368085	0.260846269
C	-0.127069623	1.362484263	-0.283512391
O	-0.291399566	2.401099403	-0.906457033
N	1.140075316	0.818180325	-0.123106064
C	2.166800382	1.681382352	0.384533926
H	1.892316098	2.740475363	0.230640282
H	2.184705289	1.520750284	1.480413392
C	3.542010311	1.370994740	-0.194402342
H	3.674605365	0.279322754	-0.197560546
H	4.304361947	1.780034481	0.482305714
C	3.758034630	1.932342295	-1.595670105
H	3.688730187	3.032189786	-1.560542013
H	4.790024911	1.709414744	-1.908210410
C	2.793468524	1.409012830	-2.652617247
H	1.770644032	1.730427377	-2.409119051
H	2.781411236	0.308814436	-2.621358156
C	3.161068616	1.893074349	-4.047610091
H	4.172363590	1.570678535	-4.327818587
H	2.469891277	1.513700287	-4.807622548
H	3.141324842	2.989143089	-4.102099863

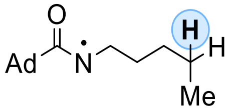

Conformer 14

C	-3.182845815	-1.458776386	-0.395086359
C	-2.667780098	-1.145957122	-1.803370785
C	-3.178263485	0.231450909	-2.238646006
C	-2.674996759	1.298613867	-1.261907735
C	-3.186691353	0.982043892	0.146540410
C	-2.677774816	-0.394907353	0.583793976
H	-2.839131006	-2.455687216	-0.080743861
H	-4.283114295	-1.481559755	-0.390819609
H	-3.026740199	-1.911246142	-2.506062540
H	-4.278393910	0.236380313	-2.265719203
H	-2.830766839	0.458325945	-3.257968591
H	-3.038262549	2.288279939	-1.573947105
H	-4.286817488	0.997540571	0.158110207
H	-2.846618441	1.753041739	0.854130695
H	-3.040643939	-0.619248609	1.597045904
C	-1.146966281	-0.387523528	0.591191968
H	-0.757903606	-1.365307636	0.917247368
H	-0.767666183	0.365197746	1.296983506
C	-1.137490096	-1.145734282	-1.795581874
H	-0.747200926	-2.129015841	-1.498691410
H	-0.744199775	-0.948261320	-2.803897536
C	-1.143625166	1.303833442	-1.256588362
H	-0.763355937	2.073812810	-0.568088334
H	-0.761049112	1.547116334	-2.260540945
C	-0.619629102	-0.079662790	-0.825776737
C	0.892434114	-0.074362695	-0.807565702
O	1.586617102	-0.945586306	-1.319031917
N	1.464636514	0.945259264	-0.063298101
C	2.767363241	1.386834254	-0.459114358
H	2.915307024	1.333645793	-1.549084387
H	2.877249413	2.429098619	-0.125957584
C	3.885989327	0.552668470	0.208374317
H	4.828072071	1.033985044	-0.087340408
H	3.872386914	-0.444993949	-0.248282446
C	3.811186939	0.438460609	1.724837699
H	4.813010927	0.172433958	2.097261701
H	3.582463270	1.422901438	2.165750353
C	2.819322102	-0.597868590	2.243728138
H	2.985069774	-1.549951482	1.716622867
H	1.796953115	-0.284334926	1.991358764
C	2.940546008	-0.804275445	3.744239529
H	2.219839045	-1.541351497	4.114244641
H	3.944583774	-1.154275379	4.016891714
H	2.763169194	0.133497308	4.286482366

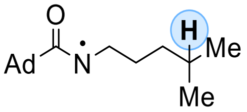

Conformer 1

C	-2.197789016	1.455969593	-2.133514758
C	-2.041554775	0.737299033	-0.789565618
C	-2.626524886	-0.674379150	-0.891985692
C	-1.884851819	-1.457793367	-1.979248531

C	-2.042136675	-0.737817275	-3.322300136
C	-1.457177273	0.674045355	-3.223011090
H	-1.798436129	2.478913517	-2.063673290
H	-3.263633648	1.543324012	-2.392094576
H	-2.565836521	1.301059204	-0.004382078
H	-3.699734619	-0.621156224	-1.128988068
H	-2.536125818	-1.191937394	0.075126763
H	-2.301080940	-2.472371376	-2.053647484
H	-3.105784248	-0.685252225	-3.599338727
H	-1.530456812	-1.303369043	-4.115287604
H	-1.565657908	1.189703803	-4.187890209
C	0.029472001	0.585755690	-2.868435105
H	0.474486921	1.589813047	-2.803687475
H	0.581331286	0.031415792	-3.642516329
C	-0.554375967	0.650216933	-0.434249903
H	-0.126548841	1.660135762	-0.343264278
H	-0.431232736	0.153816379	0.543142363
C	-0.399123171	-1.548046665	-1.624864662
H	0.153364829	-2.119698340	-2.381853591
H	-0.261733969	-2.077396349	-0.668166948
C	0.199706333	-0.140026542	-1.516056148
C	1.686215373	-0.241363056	-1.233953522
O	2.377333114	-1.172061210	-1.623831422
N	2.258883812	0.862573721	-0.617025025
C	3.246564555	0.569962676	0.386456530
H	3.936295074	-0.174060430	-0.045670137
H	3.808991443	1.483213028	0.617849717
C	2.618741486	-0.013416211	1.657110908
H	3.425489708	-0.357130969	2.318225935
H	2.044735316	-0.913234270	1.377597123
C	1.716337078	0.983093477	2.375140086
H	2.332800934	1.753533561	2.869143065
H	1.111236721	1.520428232	1.629233078
C	0.778980955	0.353566104	3.402939453
H	0.195298461	-0.427825595	2.883104739
C	-0.193766151	1.396218175	3.936331624
H	-0.778063186	1.851151223	3.125690805
H	-0.896312880	0.961192582	4.656875225
H	0.348856688	2.203474664	4.448691534
C	1.543677813	-0.306011233	4.544861538
H	0.858775935	-0.725411674	5.291462991
H	2.190493665	-1.120323359	4.197601672
H	2.178940700	0.431705490	5.056259311

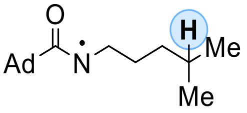

Conformer 2

C	-2.441520191	-1.499576152	0.856732195
C	-1.584389830	-1.804277009	-0.375298168
C	-2.476075841	-1.859421489	-1.618908559
C	-3.180288483	-0.511973596	-1.805014408
C	-4.038503520	-0.211674980	-0.572519423
C	-3.147331832	-0.152848985	0.672441045
H	-1.809943810	-1.474870103	1.758537400
H	-3.183287547	-2.298404786	1.006942610
H	-1.074103868	-2.768878360	-0.242324063
H	-3.220832241	-2.662339309	-1.512206606
H	-1.871444904	-2.096793441	-2.507129776
H	-3.816174018	-0.547328880	-2.701138214
H	-4.806207485	-0.990492994	-0.449917674
H	-4.566878402	0.744269765	-0.703736813
H	-3.762404437	0.066235819	1.556726441
C	-2.103740713	0.952890429	0.498521852
H	-1.469217824	1.025698069	1.397006508
H	-2.582667388	1.933025910	0.374851390
C	-0.535229315	-0.702144222	-0.548680472
H	0.122437130	-0.676481279	0.335732007
H	0.103314431	-0.912627166	-1.420031765
C	-2.132712395	0.590957532	-1.979208505
H	-2.617751233	1.568314337	-2.121785517
H	-1.511821960	0.399907846	-2.866830942
C	-1.229574169	0.658625505	-0.726707893
C	-0.240251996	1.789358694	-0.939960799
O	-0.450858273	2.945194230	-0.596845625
N	0.872736268	1.466411434	-1.698457657
C	2.139404871	1.996927724	-1.280373664
H	2.790691364	2.089636043	-2.156734800
H	1.983390056	2.996116734	-0.838636650
C	2.800800125	1.076609549	-0.239542778
H	3.863357369	1.348079544	-0.183573098
H	2.764722350	0.042480933	-0.617248451
C	2.160274668	1.152955258	1.139103694
H	2.415135222	2.115320831	1.613748281
H	1.063657269	1.160771814	1.043694695
C	2.545730704	0.007399634	2.073371250
H	2.296388292	-0.938847414	1.560283717
C	1.730463134	0.081948521	3.357220323
H	0.652462214	0.046750988	3.150602528
H	1.970493255	-0.744711225	4.036333580
H	1.934887771	1.020409042	3.891440320
C	4.037394292	0.002578729	2.383018616
H	4.293233599	-0.795085516	3.090645223
H	4.649246024	-0.145535531	1.485633571
H	4.335531523	0.957859806	2.838773571

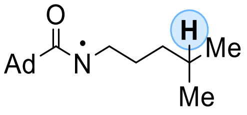

Conformer 3

C	-3.541434353	0.641155436	-2.474275649
C	-2.599760103	1.754103980	-2.003822226
C	-3.054169080	2.262648709	-0.632005212
C	-3.032156647	1.110340939	0.377417716
C	-3.975028395	0.000424534	-0.096264612
C	-3.519802485	-0.512299575	-1.465961441
H	-3.233537461	0.280513527	-3.467415018
H	-4.564042565	1.034059343	-2.578122133

H	-2.611506234	2.581353128	-2.727820972
H	-4.069384750	2.680628470	-0.704376565
H	-2.394539549	3.075477971	-0.292698385
H	-3.354503242	1.475651732	1.362960577
H	-5.004996962	0.382966302	-0.158666503
H	-3.982446168	-0.824902464	0.631465727
H	-4.194847767	-1.311022861	-1.804467925
C	-2.098025828	-1.067078931	-1.352622680
H	-1.757412404	-1.463428057	-2.320757121
H	-2.058986401	-1.905780536	-0.643975460
C	-1.175932983	1.202542657	-1.893171473
H	-0.826583523	0.850087528	-2.877057760
H	-0.482227199	1.992994395	-1.565934730
C	-1.607820697	0.559646299	0.489416335
H	-1.570364694	-0.263057440	1.220041947
H	-0.916122321	1.338183535	0.840606981
C	-1.144433568	0.036844188	-0.886336248
C	0.265283470	-0.497780378	-0.775680507
O	0.560929140	-1.684642322	-0.805521320
N	1.237808020	0.458151835	-0.529979839
C	2.496826797	0.313019971	-1.201853846
H	2.529538303	-0.612791546	-1.799501371
H	2.577771714	1.166797228	-1.898179575
C	3.656283765	0.357119236	-0.211004615
H	3.606992538	1.299467246	0.355122780
H	4.595484187	0.372011050	-0.781493101
C	3.619257226	-0.824683573	0.744869710
H	2.626024508	-0.858643447	1.219609178
H	3.701809483	-1.764256667	0.170394766
C	4.691323403	-0.801983342	1.831978175
H	4.562012638	0.130346534	2.410144112
C	4.499641282	-1.983417805	2.772495478
H	3.492135526	-1.997713344	3.205453465
H	4.638638924	-2.929793699	2.230383235
H	5.223011710	-1.964691546	3.596235766
C	6.100220868	-0.813978012	1.249994669
H	6.853658761	-0.892334616	2.043496867
H	6.231032284	-1.679636370	0.583815177
H	6.325304277	0.089696180	0.671824044

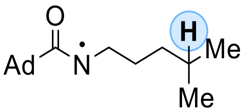

Conformer 4

C	-3.381346309	-1.980173173	-0.343080426
C	-2.401338063	-1.306455982	-1.308747656
C	-3.004418372	0.008808001	-1.810979158
C	-3.274227312	0.938689700	-0.624027178
C	-4.254964045	0.262557163	0.339166807
C	-3.653568942	-1.051930813	0.844908717
H	-2.966545237	-2.935902766	0.010904903
H	-4.322734600	-2.210667049	-0.863931670
H	-2.202613316	-1.973105316	-2.159994877
H	-3.939757078	-0.190140237	-2.355477393

H	-2.316860815	0.492839720	-2.521134590
H	-3.704345445	1.883409899	-0.985671147
H	-5.210728032	0.068273416	-0.170327665
H	-4.471480846	0.929399244	1.187090404
H	-4.353090000	-1.536893204	1.540683004
C	-2.339833073	-0.764706767	1.576962298
H	-1.896656823	-1.694674930	1.960762986
H	-2.515667730	-0.104865057	2.440316862
C	-1.086394793	-1.016831707	-0.578393856
H	-0.629531410	-1.953218387	-0.222816418
H	-0.366913663	-0.546006281	-1.268962756
C	-1.962426408	1.229327933	0.108025464
H	-2.126732142	1.906022926	0.957244924
H	-1.249796126	1.736130614	-0.560876464
C	-1.346830381	-0.079104159	0.613342871
C	-0.066852754	0.204914275	1.371663126
O	0.220192176	1.298159005	1.838003104
N	0.722894187	-0.912439221	1.615939774
C	2.136333245	-0.719086703	1.484370434
H	2.403539158	0.209481732	2.018514260
H	2.666675596	-1.558387523	1.951926119
C	2.559248413	-0.574321555	0.017874549
H	2.088066441	0.330991656	-0.397914887
H	2.162334550	-1.428326344	-0.549558661
C	4.070999250	-0.491148377	-0.124696586
H	4.460312764	0.255810397	0.585646035
H	4.520515235	-1.454319792	0.172293021
C	4.553841536	-0.130377098	-1.528633677
H	4.134270224	0.858155126	-1.782879975
C	6.073780847	-0.031774513	-1.551745960
H	6.446644240	0.689811481	-0.814995762
H	6.524514620	-1.008052919	-1.321400379
H	6.439663555	0.275380433	-2.538449487
C	4.077206915	-1.133162511	-2.572879261
H	4.466631550	-0.878793021	-3.565926475
H	4.429675674	-2.144737036	-2.324607218
H	2.984581766	-1.169210689	-2.651635789

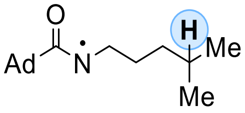

Conformer 5

C	-3.120853432	0.729659214	-0.257701769
C	-3.137638207	0.282116863	1.207297813
C	-3.884282393	-1.049962849	1.324404931
C	-3.178758198	-2.110781265	0.473646537
C	-3.163424226	-1.662205864	-0.990819079
C	-2.417028175	-0.330275844	-1.110923861
H	-2.602692512	1.695772105	-0.352657388
H	-4.149998189	0.879254817	-0.616818349
H	-3.638708732	1.044133532	1.821298172
H	-4.925331163	-0.928284485	0.989463582
H	-3.919283754	-1.372065118	2.375793860
H	-3.711149113	-3.068387368	0.561379109

H	-4.192677623	-1.551775660	-1.363961266
H	-2.675580602	-2.426212667	-1.615024846
H	-2.398654246	-0.007524138	-2.161596128
C	-0.978515468	-0.503507324	-0.614533729
H	-0.425942641	0.444825511	-0.707948447
H	-0.453083287	-1.250923452	-1.232632036
C	-1.699972643	0.108099128	1.703903136
H	-1.148906128	1.057664920	1.641029662
H	-1.690378605	-0.207843334	2.758176869
C	-1.742435053	-2.286205282	0.971353852
H	-1.223786419	-3.061538164	0.386330830
H	-1.726117759	-2.621233218	2.016703438
C	-0.980335073	-0.961621908	0.852545718
C	0.423435580	-1.117947059	1.401581781
O	0.727708463	-1.965657907	2.230554245
N	1.321206954	-0.127189666	1.023272848
C	2.694425708	-0.524138836	0.900969879
H	3.332326720	0.354406544	1.058939035
H	2.913818035	-1.273089116	1.678468265
C	2.990001436	-1.154919387	-0.469672649
H	2.444832834	-2.108239633	-0.535053185
H	4.058450566	-1.410579226	-0.486439634
C	2.625992149	-0.277454735	-1.663122743
H	2.976942666	-0.772846830	-2.583271225
H	1.530746469	-0.224986077	-1.747879984
C	3.177055387	1.148106136	-1.641059266
H	2.796573430	1.648992685	-0.735392271
C	2.658948483	1.915540109	-2.849439674
H	3.004191432	1.445787051	-3.781367031
H	1.562124906	1.933727767	-2.873174196
H	3.013231584	2.953208389	-2.850463499
C	4.699701758	1.168641018	-1.615445333
H	5.114880057	0.689045572	-0.720819498
H	5.101334676	0.639201055	-2.491886483
H	5.083448726	2.195426612	-1.645928765

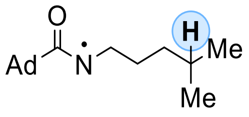

Conformer 6

C	-4.036673859	-0.974349154	2.004820107
C	-2.908500197	-1.660220090	1.227906303
C	-3.382414956	-1.970703416	-0.195552423
C	-3.776199563	-0.669735749	-0.902588364
C	-4.904732904	0.011730853	-0.123217476
C	-4.430925502	0.327175049	1.298887270
H	-3.711137653	-0.761954912	3.034494081
H	-4.905983408	-1.645371032	2.074781990
H	-2.621757952	-2.592676739	1.734834140
H	-4.241951069	-2.656919006	-0.164049351
H	-2.584410893	-2.479708424	-0.756831700
H	-4.113085321	-0.892038497	-1.925165612
H	-5.788483170	-0.643269227	-0.090984847
H	-5.209647417	0.938095135	-0.632788770
H	-5.239803901	0.819418982	1.857162982
C	-3.221182224	1.261958362	1.234281621
H	-2.876549416	1.521887747	2.246281769
H	-3.482186956	2.209583404	0.743563978
C	-1.695525672	-0.728054582	1.163862198
H	-1.328043233	-0.510338916	2.180137478
H	-0.870139053	-1.210382281	0.616898558
C	-2.563008882	0.262227319	-0.967401619
H	-2.824977019	1.199118463	-1.482560786
H	-1.744633392	-0.203177833	-1.534380912
C	-2.083262733	0.587559611	0.462696123
C	-0.882104561	1.506167793	0.391112793
O	-0.907149902	2.694747502	0.672035342
N	0.264452252	0.915200341	-0.122966410
C	1.495280560	1.205701925	0.545869042
H	1.440197058	2.217730083	0.986977040
H	1.582472762	0.515781046	1.411227256
C	2.700444783	1.046142355	-0.362987941
H	2.593387680	1.734218035	-1.214868159
H	2.687802332	0.031588617	-0.783762409
C	4.007048700	1.320904213	0.366877324
H	3.931845148	2.290176289	0.886818228
H	4.154202929	0.566711966	1.160553126
C	5.241210459	1.337044884	-0.530590056
H	5.073340179	2.089753256	-1.319511792
C	5.474156294	-0.012191038	-1.201477475
H	6.380805700	0.005926363	-1.817227071
H	5.601654978	-0.796422831	-0.441950908
H	4.642544506	-0.307737411	-1.851647945
C	6.474129125	1.746318059	0.267185409
H	6.677611886	1.013053281	1.060700482
H	7.363740854	1.796084785	-0.371029161
H	6.345162134	2.725735219	0.743917062

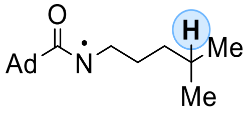

Conformer 7

C	-2.823417404	-2.343895414	-0.715604009
C	-2.877317799	-1.165523937	-1.693125264
C	-3.966019525	-0.182967646	-1.250411812
C	-3.653838295	0.331499109	0.158699217
C	-3.602816996	-0.850093801	1.132620894
C	-2.511194622	-1.830681932	0.693852861
H	-2.055299138	-3.064551025	-1.034486305
H	-3.785773407	-2.877148049	-0.717532409
H	-3.098548646	-1.533954705	-2.705004188
H	-4.947613372	-0.680030473	-1.263100083
H	-4.023538259	0.659642821	-1.956022205
H	-4.434468524	1.037556630	0.475284402
H	-4.577516057	-1.360157927	1.159935445
H	-3.399284861	-0.489794666	2.152155220
H	-2.468165955	-2.676765444	1.394483913
C	-1.155519725	-1.118923727	0.686399169

H	-0.355111083	-1.810131284	0.386724858
H	-0.908152253	-0.749899560	1.693623106
C	-1.522201295	-0.453241099	-1.701807856
H	-0.728551292	-1.143391618	-2.028836353
H	-1.537200808	0.385995039	-2.415936826
C	-2.299925810	1.045415121	0.154507156
H	-2.057693944	1.435291021	1.152568767
H	-2.321697719	1.913418356	-0.521329265
C	-1.202204882	0.074801433	-0.291413021
C	0.143016606	0.764971690	-0.293273534
O	0.372012019	1.831968519	0.261221376
N	1.167065091	0.049667215	-0.886861368
C	2.086008834	0.752292784	-1.736318573
H	1.884852866	0.403945212	-2.765779746
H	1.898679940	1.838570057	-1.721146239
C	3.530341623	0.422163321	-1.369571699
H	4.194980818	0.889442299	-2.109371188
H	3.670139696	-0.662986232	-1.462975240
C	3.894853392	0.858142616	0.045048896
H	3.243267387	0.319553542	0.751560246
H	4.923853188	0.528603327	0.264848625
C	3.796148559	2.357560757	0.336542361
H	2.748661705	2.666660399	0.197456285
C	4.158018348	2.612721363	1.793618880
H	3.511125409	2.039762283	2.468934590
H	5.198808798	2.318813466	1.994868857
H	4.057805588	3.673430962	2.053025297
C	4.686514487	3.178812293	-0.588169518
H	5.730340078	2.837168660	-0.522628195
H	4.381094855	3.110294946	-1.639470419
H	4.670027892	4.240735369	-0.313887699

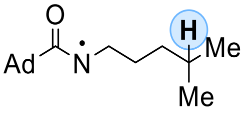

Conformer 8

C	-2.366248567	0.374558385	-2.184511616
C	-2.833019726	1.029231717	-0.880857037
C	-3.860666787	0.123803449	-0.193986171
C	-3.224521155	-1.234719752	0.117167689
C	-2.757778962	-1.888099574	-1.187378933
C	-1.730415554	-0.984109201	-1.875500846
H	-1.637195068	1.024918916	-2.693084861
H	-3.216936481	0.246948420	-2.870610182
H	-3.288930382	2.005224867	-1.099516025
H	-4.737541017	-0.011875410	-0.844766502
H	-4.217478785	0.595435774	0.733719673
H	-3.959189937	-1.883697316	0.614934543
H	-3.617609955	-2.055851855	-1.853100566
H	-2.313900444	-2.873304303	-0.979775256
H	-1.389028302	-1.455055676	-2.808289973
C	-0.529206226	-0.781754409	-0.947540747
H	0.216998499	-0.139216589	-1.442223767
H	-0.037728405	-1.742628642	-0.734454569

C	-1.634920896	1.230692472	0.048971937
H	-0.891604868	1.895524616	-0.421598803
H	-1.940017153	1.712724603	0.986796085
C	-2.023625489	-1.035253492	1.045507730
H	-1.556665168	-2.001449190	1.289324629
H	-2.336125855	-0.572288491	1.993368559
C	-0.981238773	-0.121887860	0.362615236
C	0.153971884	0.077924745	1.344768332
O	0.127468944	0.934128665	2.222371303
N	1.168235077	-0.863092963	1.315847093
C	2.475984017	-0.433322920	1.723090700
H	2.468459706	0.617862396	2.048879287
H	2.780437525	-1.052148107	2.580457866
C	3.492845689	-0.661574978	0.595670087
H	3.579948696	-1.739788033	0.409658221
H	4.470701277	-0.327743972	0.968796533
C	3.140356544	0.043853832	-0.709971712
H	3.973640484	-0.086940854	-1.419589486
H	2.278061087	-0.468405082	-1.162864594
C	2.830690949	1.537691537	-0.605146300
H	1.977575460	1.676269616	0.083343802
C	2.421737278	2.082045622	-1.968761019
H	3.242423214	1.970354920	-2.691565722
H	1.548919933	1.553887149	-2.375744454
H	2.171295915	3.148286604	-1.914927240
C	4.016496530	2.321962486	-0.056714827
H	4.294172470	2.010335660	0.956998484
H	4.896247562	2.182659065	-0.701500774
H	3.798868636	3.395912004	-0.021244125

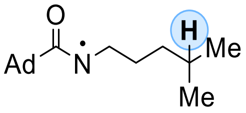

Conformer 9

C	-3.333807019	-0.967774396	1.717205539
C	-2.631555804	-1.774222288	0.620295680
C	-3.587629144	-1.971585007	-0.560257564
C	-4.009382115	-0.607164977	-1.114951342
C	-4.710490636	0.196302617	-0.015689149
C	-3.752949868	0.397714538	1.163205279
H	-2.661268421	-0.835819830	2.578335773
H	-4.216470494	-1.515757232	2.079863339
H	-2.325629409	-2.752806113	1.016923210
H	-4.474255370	-2.536296362	-0.235197295
H	-3.098551580	-2.564798793	-1.347501869
H	-4.692222734	-0.748558001	-1.964916973
H	-5.615378676	-0.333072733	0.318799193
H	-5.034560428	1.171403869	-0.409112680
H	-4.254292924	0.977676353	1.950998859
C	-2.514535933	1.160344562	0.686697695
H	-1.821985171	1.336774833	1.523889527
H	-2.788231855	2.150059667	0.296736746
C	-1.389801781	-1.013354320	0.145856080
H	-0.686253561	-0.878824521	0.983867803

H	-0.861540581	-1.585498582	-0.632526613
C	-2.768690710	0.152482397	-1.592491167
H	-3.051921661	1.133517542	-2.003875435
H	-2.256907588	-0.401528240	-2.392003357
C	-1.800181096	0.362903131	-0.408219060
C	-0.586510992	1.119795888	-0.906106989
O	-0.395047344	2.311691436	-0.703931730
N	0.258831580	0.376294694	-1.723292668
C	1.657283594	0.636853240	-1.566164839
H	2.211210375	0.216290744	-2.415464095
H	1.793125540	1.732229201	-1.563130923
C	2.195950601	0.068004901	-0.245275661
H	1.959665284	-1.003953748	-0.201918002
H	1.653320445	0.541077458	0.586969538
C	3.696903001	0.272340265	-0.088964394
H	4.010127502	-0.159013252	0.876165904
H	4.225080378	-0.306421451	-0.863344666
C	4.176953821	1.725025282	-0.144291981
H	3.976509372	2.118508852	-1.155995390
C	3.455585353	2.605923209	0.867786207
H	2.384399230	2.706150294	0.656619027
H	3.562730868	2.190920349	1.881066806
H	3.883077693	3.615529331	0.879048894
C	5.680963217	1.774086934	0.084775310
H	5.925729742	1.415454427	1.094840988
H	6.221538278	1.143360092	-0.631409209
H	6.068229601	2.795954822	-0.005666759

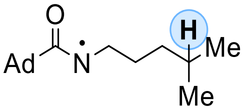

Conformer 10

C	-3.073110802	0.517097346	-1.897593806
C	-3.412349902	0.961189416	-0.471532274
C	-4.153670111	-0.169217529	0.249781868
C	-3.265824151	-1.416147022	0.299504171
C	-2.924085713	-1.857621415	-1.127252528
C	-2.183577371	-0.729139009	-1.851604726
H	-2.558294480	1.329004217	-2.433509428
H	-3.996474986	0.299612104	-2.455421702
H	-4.048340002	1.857122292	-0.505520990
H	-5.094596280	-0.397836129	-0.273096951
H	-4.420146839	0.145241868	1.269902583
H	-3.793302208	-2.227363296	0.821507142
H	-3.844563330	-2.113380573	-1.673155888
H	-2.301064449	-2.764339432	-1.103623559
H	-1.935209208	-1.046179152	-2.874522982
C	-0.890470344	-0.404285094	-1.097925244
H	-0.340725177	0.395583707	-1.620874072
H	-0.230422824	-1.284871609	-1.068003345
C	-2.121863497	1.286214706	0.284308455
H	-1.583035784	2.111604445	-0.206318826
H	-2.338106758	1.620607159	1.307749789
C	-1.973644731	-1.093296458	1.054537004
H	-1.326116968	-1.979736965	1.113840562
H	-2.196502612	-0.780634732	2.086158891
C	-1.221576668	0.047620941	0.334663839
C	0.039294735	0.349501849	1.116943250
O	0.145519722	1.279683466	1.903598953
N	1.048978718	-0.594670929	0.977784877
C	2.384117085	-0.080292220	0.906333122
H	2.494345298	0.702548386	1.678191453
H	3.091610367	-0.890283758	1.119761460
C	2.672936707	0.551126701	-0.463062708
H	1.913650806	1.324785187	-0.649397899
H	2.537233645	-0.210118395	-1.244835322
C	4.058383407	1.181427869	-0.548547458
H	4.180809705	1.620877155	-1.552200143
H	4.108839203	2.024333350	0.158546776
C	5.245883043	0.252441889	-0.286101760
H	5.174866333	-0.121091532	0.749140246
C	6.547622970	1.034553506	-0.407798562
H	6.577368446	1.882855582	0.286700537
H	7.418114581	0.399453061	-0.205262485
H	6.660136528	1.435820941	-1.425044920
C	5.257683824	-0.945753535	-1.227117847
H	6.142017986	-1.571323556	-1.057326099
H	4.376769441	-1.586882232	-1.103789951
H	5.282177324	-0.610514108	-2.273944590

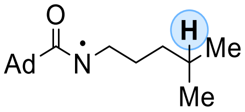

Conformer 11

C	-3.786156748	-1.045489515	-1.672186690
C	-4.068020944	-0.189767787	-0.433185948
C	-4.382108144	-1.103482137	0.755206351
C	-3.186506286	-2.020425281	1.029553483
C	-2.905923806	-2.875354621	-0.210475029
C	-2.592014966	-1.965907572	-1.402798842
H	-3.576832163	-0.398966270	-2.537929403
H	-4.672442666	-1.645825387	-1.927262277
H	-4.924765615	0.470641999	-0.628385475
H	-5.277509427	-1.706705791	0.542034036
H	-4.606749826	-0.498327511	1.646272633
H	-3.407998445	-2.672919009	1.886137753
H	-3.778705732	-3.505310092	-0.438707304
H	-2.060627096	-3.553373310	-0.019177904
H	-2.386905547	-2.578458902	-2.292353089
C	-1.359497155	-1.117070403	-1.080347377
H	-1.107309547	-0.470214646	-1.936650060
H	-0.487597222	-1.763551786	-0.893506813
C	-2.838448348	0.661194414	-0.108080629
H	-2.607323108	1.343292823	-0.939765171
H	-3.019808461	1.295298226	0.770601914
C	-1.953151721	-1.172310741	1.353036046
H	-1.085790573	-1.812790588	1.565667434
H	-2.133085513	-0.559018057	2.249344382

C	-1.634649886	-0.246105568	0.160776662
C	-0.409778846	0.585868559	0.476589867
O	-0.431481115	1.781002261	0.727557142
N	0.771695742	-0.140516665	0.543023734
C	1.905190897	0.416646185	-0.127553490
H	1.851655015	0.076886168	-1.182672728
H	1.816197195	1.518102643	-0.168080246
C	3.217270167	-0.022388225	0.500452474
H	3.283601927	-1.118708832	0.459316314
H	3.188900972	0.236994825	1.566856468
C	4.433049075	0.625734722	-0.149486951
H	4.396143555	1.713528960	0.023613723
H	5.341409954	0.270599514	0.363877777
C	4.604340950	0.373712166	-1.650639428
H	3.738311048	0.809668920	-2.178191998
C	4.662630556	-1.114317050	-1.971281735
H	5.476555232	-1.593096878	-1.407704076
H	3.732692481	-1.637056877	-1.717355411
H	4.855949109	-1.284750363	-3.037453174
C	5.858030160	1.078320265	-2.150644405
H	6.750589747	0.661573271	-1.662622401
H	5.986095880	0.958247307	-3.232880349
H	5.833317178	2.152777572	-1.930625288

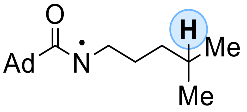

Conformer 12

C	-4.869751249	-0.023753203	-0.494709601
C	-4.039707123	-0.866598256	-1.468179215
C	-3.702559663	-0.030416039	-2.707002233
C	-2.903263082	1.208117584	-2.290351429
C	-3.732294087	2.047266022	-1.312935748
C	-4.068883503	1.211971366	-0.073420821
H	-5.134524120	-0.621912300	0.390074689
H	-5.813548738	0.283634813	-0.969784585
H	-4.612252356	-1.755639092	-1.768084851
H	-4.626470874	0.274694545	-3.220881710
H	-3.121111472	-0.632104735	-3.421918090
H	-2.658291666	1.808415072	-3.178173546
H	-4.658160279	2.386118162	-1.801333383
H	-3.173474590	2.949205528	-1.021083033
H	-4.660318917	1.815696470	0.629669906
C	-2.771638104	0.773791507	0.612688214
H	-2.994577329	0.180546048	1.512759511
H	-2.187960024	1.647373060	0.935262631
C	-2.745318757	-1.307990039	-0.781491245
H	-2.959279004	-1.922365841	0.103773368
H	-2.142688576	-1.935190679	-1.455096250
C	-1.606154651	0.770021894	-1.605436499
H	-1.013615954	1.649651344	-1.308173824
H	-0.987289782	0.181337205	-2.302371522
C	-1.933305993	-0.079279982	-0.361979991
C	-0.647072735	-0.506491269	0.312168556
O	-0.408516963	-1.645886303	0.682031741
N	0.241139917	0.530334175	0.568132813
C	1.612270210	0.294440797	0.231540069
H	1.823538984	-0.785424011	0.328361656
H	1.737492580	0.520403919	-0.849101136
C	2.564649998	1.152402407	1.046668432
H	2.487396946	0.857761836	2.102608826
H	2.218570624	2.193436486	0.998452677
C	4.007462257	1.065817064	0.564191227
H	4.071583707	1.481925357	-0.454939844
H	4.634341423	1.717335483	1.194054754
C	4.628074184	-0.334788142	0.558573550
H	4.066341444	-0.964491740	-0.152230587
C	6.070596305	-0.254935873	0.076226131
H	6.673436551	0.347996121	0.770456529
H	6.532841510	-1.247707637	0.014112078
H	6.140753781	0.212383134	-0.913353756
C	4.561901997	-0.986656540	1.934023386
H	3.532516505	-1.165084341	2.265911076
H	5.080480283	-1.952883872	1.939553304
H	5.047894236	-0.346310371	2.684381225

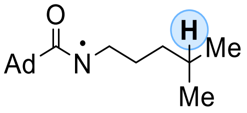

Conformer 13

C	-3.852040986	-0.748591075	-0.133037820
C	-3.685597886	0.429342048	0.832331730
C	-3.352312615	-0.101544740	2.230525749
C	-2.051606007	-0.908928605	2.178396798
C	-2.216735616	-2.083267226	1.208407077
C	-2.550635063	-1.553999338	-0.190141804
H	-4.112315224	-0.379992531	-1.136520520
H	-4.679351392	-1.394513434	0.197558742
H	-4.618137719	1.009901577	0.870977308
H	-4.172817012	-0.734674003	2.600351012
H	-3.248875261	0.735643098	2.937481196
H	-1.808946511	-1.288453661	3.181177591
H	-3.018221493	-2.750328674	1.559509059
H	-1.292474628	-2.679627620	1.176177626
H	-2.665631154	-2.396786325	-0.886627953
C	-1.412591343	-0.653633402	-0.678914695
H	-1.630513175	-0.267776635	-1.686664468
H	-0.469881322	-1.216648247	-0.742291719
C	-2.549969163	1.331166207	0.342106008
H	-2.770212330	1.734980881	-0.655315789
H	-2.428784946	2.198067901	1.009104907
C	-0.913037121	-0.008708521	1.691151013
H	0.032357691	-0.571727300	1.658231440
H	-0.766077364	0.831746365	2.389305785
C	-1.241423854	0.536954860	0.290074351
C	-0.113974650	1.409300465	-0.220165223
O	-0.276404806	2.453027184	-0.835568579
N	1.151344457	0.864807487	-0.050462790

C	2.199458608	1.736080217	0.390135757
H	1.934653908	2.788486060	0.182756029
H	2.238196010	1.637398727	1.492692291
C	3.554514105	1.367961823	-0.200632691
H	3.685381761	0.280078715	-0.117219975
H	4.338449961	1.821672520	0.420743119
C	3.742487720	1.832225483	-1.641695833
H	3.730401272	2.934308979	-1.658212493
H	4.752347915	1.539078716	-1.975678448
C	2.727608449	1.320819919	-2.669402683
H	1.725396884	1.670216605	-2.367734635
C	3.040906760	1.922612747	-4.032509837
H	4.027304069	1.585981071	-4.384140705
H	2.302163327	1.619946528	-4.784090108
H	3.055800628	3.018843001	-3.997431139
C	2.707517638	-0.199440655	-2.750760838
H	3.710816751	-0.586272578	-2.985587478
H	2.373445445	-0.656851211	-1.813116251
H	2.030595282	-0.541553537	-3.543994239

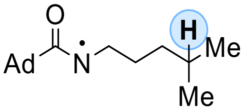

Conformer 14

C	-2.632738776	-1.879860083	-0.406113616
C	-2.512814488	-1.291601871	-1.815248260
C	-3.366129561	-0.023189369	-1.913141987
C	-2.877119251	1.004182326	-0.888267481
C	-2.995268747	0.415205961	0.520786143
C	-2.143350614	-0.854131653	0.620450309
H	-2.038909955	-2.803360113	-0.330382817
H	-3.678247229	-2.151804442	-0.196958112
H	-2.859891882	-2.027829148	-2.553976121
H	-4.424020196	-0.264117559	-1.729722255
H	-3.302541311	0.396544274	-2.928234340
H	-3.483912674	1.918199548	-0.959442311
H	-4.046801579	0.180647087	0.743961163
H	-2.663705929	1.152288917	1.267617936
H	-2.222284753	-1.274166769	1.633219984
C	-0.679592310	-0.503132366	0.336980160
H	-0.049611620	-1.404026739	0.427017601
H	-0.315351094	0.225839819	1.076845723
C	-1.051037355	-0.942278956	-2.101629279
H	-0.422008140	-1.844470141	-2.060577348
H	-0.936821073	-0.531427618	-3.113648417
C	-1.413700339	1.354404075	-1.170960787
H	-1.046967833	2.101367580	-0.452555488
H	-1.309090655	1.789718096	-2.176716574
C	-0.545133908	0.080712328	-1.079173632
C	0.890285663	0.456735899	-1.387752421
O	1.426428804	0.232729520	-2.464807006
N	1.526953475	1.218881308	-0.411516264
C	2.946168335	1.045658704	-0.301351135
H	3.333916088	1.004815059	-1.332313825

H	3.378518723	1.918064860	0.206349574
C	3.380794479	-0.247554346	0.415707807
H	4.365206551	-0.517257306	0.011902893
H	2.704066214	-1.063908494	0.117510333
C	3.501620172	-0.184664170	1.935278945
H	4.004680211	-1.112613315	2.248165969
H	4.192957751	0.629881746	2.212860709
C	2.214342114	-0.040014991	2.755023291
H	1.440603286	-0.671489801	2.284626689
C	2.447159754	-0.549205551	4.172587620
H	1.547468235	-0.444244597	4.790806744
H	2.744158271	-1.604869397	4.182741113
H	3.248636331	0.026727049	4.657411789
C	1.701979799	1.395286640	2.810012906
H	1.456497425	1.797518918	1.820431720
H	0.795522617	1.461485942	3.425510381
H	2.458114496	2.048756172	3.269260628

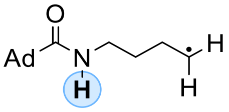

Conformer 1

C	-2.437892717	1.337443159	-2.003807118
C	-2.608819947	-0.013708306	-1.304777698
C	-2.812881052	-1.108506728	-2.356459245
C	-1.597256144	-1.163520936	-3.286290102
C	-1.426150820	0.189819015	-3.982111117
C	-1.220181955	1.283704376	-2.930237854
H	-2.311126664	2.136507399	-1.257532594
H	-3.340397867	1.581296939	-2.584116668
H	-3.480164769	0.023289673	-0.635596601
H	-3.724422044	-0.906208707	-2.938932150
H	-2.954395611	-2.081930075	-1.864130077
H	-1.742589420	-1.951032947	-4.039353970
H	-2.313229757	0.418748001	-4.591561087
H	-0.565511624	0.156805621	-4.667505773
H	-1.088732104	2.255682014	-3.426881911
C	0.033430784	0.969172226	-2.104939601
H	0.185006030	1.768403302	-1.359159908
H	0.913005689	0.956434038	-2.769930401
C	-1.356942766	-0.326367247	-0.484558850
H	-1.199587716	0.445132187	0.287706166
H	-1.454496080	-1.287636230	0.036585514
C	-0.344698246	-1.475111248	-2.465874367
H	0.537595816	-1.531339325	-3.123465985
H	-0.432377814	-2.446714416	-1.961878239
C	-0.119267755	-0.386894350	-1.400797924
C	1.060744152	-0.808125921	-0.530040608
O	1.042159034	-1.863396833	0.088522588
N	2.123429364	0.042635629	-0.459945676
C	3.208856704	-0.201234630	0.473751994
H	3.462434031	-1.265587548	0.422441038
H	4.081201520	0.370561766	0.130395078
C	2.846722773	0.180340808	1.902286506

H	3.701981592	-0.035545105	2.558076693
H	2.022605845	-0.465528179	2.235745580
C	2.443759768	1.643526312	2.050633926
H	3.242783736	2.294180779	1.653824925
H	1.571735322	1.835167994	1.391762298
C	2.124219005	2.017791318	3.448044068
H	2.067047034	3.058260054	3.754382002
H	1.763423696	1.266256097	4.146978001
H	2.084706312	0.927194375	-0.941107626

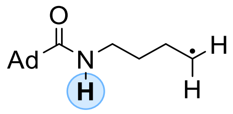

Conformer 2

C	-3.685003551	-1.160413976	0.451182060
C	-2.368307567	-1.789389615	-0.013759657
C	-2.435681407	-2.057642270	-1.519647179
C	-2.662942182	-0.738581095	-2.263509082
C	-3.980754001	-0.111443744	-1.799760087
C	-3.916075241	0.158236522	-0.293794648
H	-3.653556185	-0.979887380	1.535778463
H	-4.519355571	-1.852837714	0.262878425
H	-2.199766030	-2.733934693	0.523049912
H	-3.252213936	-2.760291912	-1.743638807
H	-1.502449516	-2.530299023	-1.862201283
H	-2.701900503	-0.926187162	-3.346132816
H	-4.817870273	-0.787437684	-2.030037599
H	-4.165714433	0.826740597	-2.344599488
H	-4.859215562	0.612328786	0.041839262
C	-2.761659346	1.117344636	0.001769879
H	-2.694977041	1.339565890	1.074280561
H	-2.920412215	2.076907485	-0.515618149
C	-1.213755091	-0.830716760	0.282559685
H	-1.134289807	-0.628201497	1.359261552
H	-0.256489170	-1.274933806	-0.035756916
C	-1.503923302	0.219987249	-1.963817005
H	-1.646333612	1.159618984	-2.522055388
H	-0.565835460	-0.241092727	-2.316482335
C	-1.425257924	0.505901042	-0.456855004
C	-0.301068800	1.443480698	-0.020353390
O	-0.201046196	1.797459712	1.146330712
N	0.587408680	1.838562313	-0.977992128
C	1.759174114	2.633754097	-0.658317013
H	1.874189740	3.417214226	-1.418426260
H	1.546046213	3.124559847	0.298142054
C	3.030712776	1.803275541	-0.557662514
H	3.876738580	2.481702551	-0.370953698
H	3.238402225	1.318173734	-1.524577524
C	2.980629614	0.744908753	0.538465129
H	2.720718342	1.236794995	1.495372992
H	2.133385244	0.061912865	0.354982874
C	4.240461151	-0.025616515	0.653315997
H	5.176692282	0.379391193	0.274946871
H	4.290027348	-0.943069068	1.231863016
H	0.488550098	1.487428838	-1.918359958

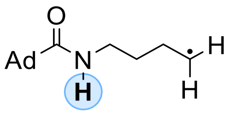

Conformer 3

C	-3.525165221	1.086460336	-2.179869763
C	-2.510619384	2.004282106	-1.493922810
C	-2.850715890	2.116612652	-0.004661034
C	-2.795373630	0.728949446	0.639798525
C	-3.808880348	-0.191334242	-0.047841042
C	-3.472920197	-0.302909571	-1.537810056
H	-3.304802088	1.013266934	-3.255790356
H	-4.535294866	1.512623710	-2.087287581
H	-2.537784681	3.000750620	-1.957490997
H	-3.854102072	2.550772031	0.118573986
H	-2.143575008	2.795593083	0.495625124
H	-3.033858279	0.808365699	1.710055345
H	-4.827091566	0.205732704	0.080696688
H	-3.789325730	-1.187744312	0.417605429
H	-4.198072523	-0.966121090	-2.030699325
C	-2.067830066	-0.883890159	-1.701123684
H	-1.818467170	-0.981633224	-2.769729360
H	-1.997724663	-1.887572278	-1.263193322
C	-1.100689787	1.420750173	-1.648274971
H	-0.833691515	1.370921248	-2.716425837
H	-0.386396815	2.101505975	-1.154295891
C	-1.390265145	0.145486998	0.478690596
H	-1.318680478	-0.848130175	0.941046687
H	-0.648014203	0.791357591	0.976594376
C	-1.030173787	0.023057805	-1.016952319
C	0.329557016	-0.667813036	-1.092841094
O	0.462926139	-1.822115953	-0.709674637
N	1.378283103	0.064970869	-1.568308227
C	2.737862799	-0.442908919	-1.545625439
H	2.655235494	-1.533375486	-1.475756192
H	3.226288201	-0.207410720	-2.500410181
C	3.551943708	0.107089148	-0.383806762
H	3.615298591	1.204264256	-0.461591852
H	4.584321422	-0.263363479	-0.472366775
C	2.992919507	-0.269015553	0.981842858
H	1.945949649	0.073670783	1.062074557
H	2.911173440	-1.370963231	1.044503062
C	3.793106412	0.275197816	2.103018204
H	3.438615730	0.213974734	3.127623305
H	4.801281202	0.650425831	1.941026924
H	1.231977275	1.031080005	-1.819721979

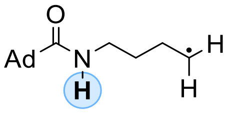

Conformer 4

C	-3.591596892	-1.986924387	-0.496224190
C	-3.189449318	-0.862159086	-1.454046063
C	-4.021385946	0.387925013	-1.150718551
C	-3.773472087	0.831322824	0.294442973
C	-4.175838682	-0.295915612	1.249583953
C	-3.341255266	-1.544053088	0.947830539
H	-3.014332135	-2.897292453	-0.718695641
H	-4.653907315	-2.239827277	-0.631103764
H	-3.363585616	-1.179940520	-2.491913665
H	-5.090618115	0.175782081	-1.301539871
H	-3.751566572	1.197477318	-1.844763026
H	-4.367489010	1.730255691	0.512837343
H	-5.246174480	-0.524686327	1.136859401
H	-4.022901123	0.019047951	2.293058914
H	-3.621242643	-2.352840718	1.638017652
C	-1.854147508	-1.219152922	1.134467086
H	-1.252314480	-2.123047849	0.942340628
H	-1.683007892	-0.915845117	2.180915460
C	-1.705473780	-0.542969770	-1.267233877
H	-1.093601960	-1.430220766	-1.496492324
H	-1.381752614	0.254098425	-1.949200280
C	-2.289301004	1.151950060	0.479513103
H	-2.095699835	1.487482658	1.511103658
H	-1.974393024	1.964376098	-0.188905590
C	-1.431990395	-0.093210979	0.179532110
C	0.031180227	0.331655148	0.273275437
O	0.496297185	1.155913663	-0.502535232
N	0.782250573	-0.215293985	1.270331431
C	2.178838993	0.139751156	1.432077602
H	2.277821460	1.204208211	1.184726922
H	2.446746888	0.018925322	2.490794788
C	3.110179697	-0.676746332	0.550433064
H	2.821172227	-0.518931717	-0.498114073
H	2.976604928	-1.748840135	0.758576133
C	4.570876350	-0.290623088	0.750876243
H	4.673269658	0.796641616	0.562766092
H	4.847483897	-0.415111669	1.811326157
C	5.498588724	-1.059935690	-0.111603958
H	5.165853295	-1.429896707	-1.078836247
H	6.559656325	-1.117568304	0.111597404
H	0.387576822	-0.929452758	1.861828739

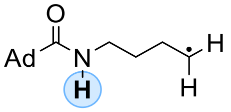

Conformer 5

C	-3.249802342	0.792630600	-0.058263130
C	-3.074459163	0.340097394	1.395138075
C	-3.854872894	-0.957875974	1.621072204
C	-3.324814105	-2.042113214	0.678606670
C	-3.504957152	-1.589824252	-0.773252655
C	-2.724838125	-0.292814638	-1.003881437
H	-2.710175287	1.737032114	-0.229026260
H	-4.312222755	0.989198119	-0.265707875
H	-3.448054430	1.121016324	2.073086352
H	-4.927352845	-0.788346070	1.441895685
H	-3.750785105	-1.283339025	2.666937910
H	-3.876823706	-2.977943607	0.845061463
H	-4.571199446	-1.430440237	-0.993713859
H	-3.147508999	-2.372267439	-1.459320003
H	-2.847293181	0.034278528	-2.046429439
C	-1.237509485	-0.531825434	-0.725323422
H	-0.673932180	0.393893249	-0.926489721
H	-0.837998588	-1.299510664	-1.406894628
C	-1.587718184	0.101182446	1.674672955
H	-1.032812656	1.043163828	1.543731202
H	-1.436394329	-0.210958105	2.719346101
C	-1.839674862	-2.278155297	0.956205587
H	-1.441323850	-3.069503788	0.306165255
H	-1.683894055	-2.625077104	1.986968878
C	-1.038621777	-0.989553570	0.732604590
C	0.438144326	-1.266190254	1.016312435
O	0.857754013	-2.364930545	1.349255921
N	1.272864559	-0.195474466	0.880575984
C	2.711313536	-0.333795531	1.005108303
H	3.118746364	0.669359350	1.184577428
H	2.919819293	-0.943788741	1.890892384
C	3.365388673	-0.958653539	-0.225368275
H	3.136817999	-2.031202158	-0.245298540
H	4.456336733	-0.865281119	-0.121056153
C	2.906301072	-0.312860245	-1.533776482
H	3.484834443	-0.752224494	-2.366110149
H	1.862053432	-0.597341379	-1.732978405
C	3.027891296	1.167025336	-1.533273191
H	3.913231461	1.645141365	-1.116154813
H	2.350027905	1.792733370	-2.108518173
H	0.916892080	0.672869839	0.506558504

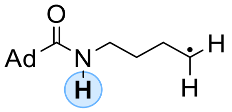

Conformer 6

C	-4.498693118	-0.862787645	1.610175323
C	-3.310633576	-1.699805861	1.127230938
C	-3.434399633	-1.934300997	-0.381798667
C	-3.446971176	-0.588038573	-1.113893078
C	-4.635630163	0.245081564	-0.628114638
C	-4.507371837	0.482787874	0.878751412
H	-4.429508404	-0.700618914	2.696152839
H	-5.439379520	-1.402591057	1.423883189
H	-3.298197068	-2.665736286	1.652309428
H	-4.358075817	-2.490181049	-0.601530930

H	-2.596227705	-2.552351429	-0.739141610
H	-3.529231176	-0.756842802	-2.197191881
H	-5.577424832	-0.277528593	-0.853530330
H	-4.665415730	1.207540859	-1.160638028
H	-5.355091563	1.087663632	1.230571154
C	-3.204254033	1.228142237	1.168091259
H	-3.095881644	1.431299271	2.241123877
H	-3.197679249	2.208001097	0.668947969
C	-2.006962550	-0.951980159	1.420247972
H	-1.894168421	-0.780803558	2.501753669
H	-1.145069833	-1.559190361	1.101657723
C	-2.140074456	0.157418943	-0.821873206
H	-2.115595751	1.117533906	-1.359774017
H	-1.300131168	-0.442683225	-1.206386021
C	-1.997818406	0.408200798	0.690453139
C	-0.716002872	1.160025784	1.054165864
O	-0.711193135	2.120056374	1.809708587
N	0.436129611	0.668113215	0.509925086
C	1.732394601	1.252024682	0.784240794
H	1.549656350	2.270931810	1.146359544
H	2.236480227	0.715710403	1.605632232
C	2.616977595	1.258659610	-0.448944511
H	2.123568333	1.831393990	-1.248320207
H	2.738813379	0.233291745	-0.833053522
C	3.993878093	1.851311819	-0.164203910
H	3.851224505	2.867416033	0.254029537
H	4.482784203	1.283480562	0.643034079
C	4.865228140	1.893069369	-1.362418668
H	4.432655602	2.075060053	-2.343850576
H	5.946448675	1.927456863	-1.274302681
H	0.395929889	-0.136526280	-0.098764092

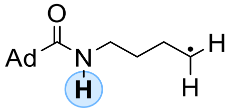

Conformer 7

C	-2.417860047	-2.381325154	0.002662268
C	-2.556373059	-1.615625431	-1.315964058
C	-3.787965268	-0.707410541	-1.252289848
C	-3.629449814	0.289633848	-0.100847742
C	-3.486335971	-0.475639609	1.218835623
C	-2.256201879	-1.386725186	1.155343264
H	-1.549048323	-3.055705521	-0.040036108
H	-3.305279909	-3.011120714	0.165438089
H	-2.661951200	-2.326165738	-2.148262077
H	-4.693715448	-1.315013251	-1.107049865
H	-3.911577812	-0.169343430	-2.204555079
H	-4.511045286	0.944813296	-0.054895069
H	-4.389506104	-1.075554775	1.407259822
H	-3.388461577	0.231819127	2.055372217
H	-2.151383774	-1.932884048	2.103597287
C	-1.007288365	-0.534892828	0.923650315
H	-0.108439590	-1.173815135	0.888414269
H	-0.858638567	0.182726185	1.740638042
C	-1.302815282	-0.761467308	-1.544383910
H	-0.420563941	-1.422289720	-1.604349452
H	-1.395684721	-0.233109631	-2.508166670
C	-2.379699667	1.141219274	-0.333808735
H	-2.243500951	1.874517624	0.472149164
H	-2.473657262	1.706804778	-1.274974284
C	-1.128270899	0.245791506	-0.399690578
C	0.099023661	1.149562306	-0.495893656
O	0.366750087	1.925417561	0.410441465
N	0.867494841	1.040392243	-1.618320413
C	2.161450926	1.684459107	-1.729714224
H	2.202526205	2.293020519	-2.643133939
H	2.231850623	2.362395928	-0.871434343
C	3.301824072	0.672205328	-1.706179251
H	4.253781264	1.222620845	-1.732734330
H	3.277143476	0.055523588	-2.618023642
C	3.262931248	-0.234189451	-0.470241666
H	2.355246423	-0.855406548	-0.504902310
H	4.107253713	-0.942379548	-0.537305658
C	3.300379999	0.532399846	0.800403169
H	2.594253045	0.360270207	1.605545261
H	4.054640452	1.303506340	0.947807896
H	0.622128754	0.347505289	-2.309748337

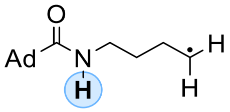

Conformer 8

C	-3.352239589	0.382244669	-1.773274679
C	-3.524656462	0.567415177	-0.262535442
C	-3.887615600	-0.775836958	0.377592152
C	-2.771030990	-1.788609984	0.108611809
C	-2.601011628	-1.973160402	-1.401688588
C	-2.235868835	-0.631946378	-2.043210456
H	-3.108342778	1.345596012	-2.244922117
H	-4.295049650	0.033009092	-2.220924359
H	-4.323000785	1.297711515	-0.068147628
H	-4.837765585	-1.149080927	-0.032918858
H	-4.032922722	-0.651685848	1.461549480
H	-3.023877460	-2.752268116	0.573757290
H	-3.532749431	-2.361908059	-1.839272951
H	-1.815879956	-2.717095449	-1.605965902
H	-2.108529109	-0.762419830	-3.127255203
C	-0.927441270	-0.117255492	-1.440686644
H	-0.638119786	0.843087833	-1.888114333
H	-0.107041482	-0.827299316	-1.639381836
C	-2.214733260	1.079960616	0.339071326
H	-1.932471428	2.048677650	-0.093391798
H	-2.328555984	1.232829359	1.424212690
C	-1.457715194	-1.273610630	0.708983043
H	-0.661902423	-2.015219882	0.524498156
H	-1.573265972	-1.167000439	1.800575467
C	-1.077330977	0.075412419	0.081535933
C	0.229083425	0.690030611	0.582451829

O	0.637081231	1.743981583	0.115612161
N	0.915190079	0.000808458	1.540921297
C	2.249948509	0.380960797	1.957751040
H	2.440475011	1.352440324	1.487999349
H	2.279314785	0.526289203	3.046452767
C	3.300265694	-0.640106645	1.530705326
H	3.176734195	-1.572283521	2.103197813
H	4.290506150	-0.244568285	1.800467075
C	3.259754845	-0.948691739	0.030945856
H	4.059944838	-1.677524117	-0.189093885
H	2.318719548	-1.466028355	-0.209357063
C	3.393498490	0.268445667	-0.809407173
H	4.138235056	1.022844876	-0.562113443
H	2.713496376	0.486984757	-1.626672835
H	0.535814914	-0.868474359	1.885217800

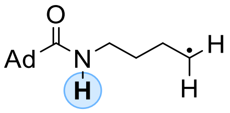

Conformer 9

C	-4.056837345	-0.464472701	1.576851967
C	-3.198818553	-1.575548440	0.963739728
C	-3.789978926	-1.989719833	-0.386752251
C	-3.812009801	-0.780847227	-1.326565521
C	-4.671850659	0.328028135	-0.712797558
C	-4.081828549	0.745022247	0.637516623
H	-3.650135177	-0.172158732	2.556207175
H	-5.080554352	-0.830036771	1.748723153
H	-3.177658444	-2.442079624	1.640090841
H	-4.810491438	-2.378397790	-0.252054015
H	-3.192360886	-2.801844641	-0.828201304
H	-4.227961428	-1.075832486	-2.300618885
H	-5.704691617	-0.028320951	-0.582191285
H	-4.714185379	1.193506567	-1.391426479
H	-4.695210400	1.543743750	1.078325457
C	-2.655423077	1.258970748	0.434306075
H	-2.209172621	1.573618841	1.387160147
H	-2.657027854	2.139338060	-0.228035171
C	-1.773265426	-1.061335775	0.758892632
H	-1.317100638	-0.765733680	1.712881156
H	-1.139073106	-1.854292880	0.330697344
C	-2.382824059	-0.263658414	-1.528809929
H	-2.404228943	0.594729678	-2.221067072
H	-1.770066821	-1.054973876	-1.991501644
C	-1.773599162	0.157176964	-0.183386110
C	-0.344820600	0.689291825	-0.251519826
O	0.243053380	1.041811070	0.763342945
N	0.243607504	0.761263001	-1.477810796
C	1.608438347	1.231534395	-1.611553528
H	1.750708401	1.608871683	-2.632751657
H	1.740343359	2.079623718	-0.926762969
C	2.639314442	0.159419285	-1.284711277
H	2.585869716	-0.649679612	-2.026543721
H	2.375725687	-0.278019242	-0.311537795
C	4.048150455	0.739716638	-1.212749677
H	4.769469450	-0.088871858	-1.089459658
H	4.315218632	1.202453098	-2.177542435
C	4.185848324	1.728105651	-0.113240431
H	3.548153709	1.654537738	0.766446051
H	4.951587483	2.497747176	-0.128907652
H	-0.252632987	0.440783983	-2.294097109

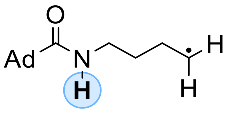

Conformer 10

C	-3.910232935	0.488600023	-1.276413738
C	-3.697185070	0.643968668	0.232689056
C	-3.909739173	-0.708359187	0.919013938
C	-2.910265679	-1.726931544	0.363207368
C	-3.125746230	-1.882750538	-1.145059942
C	-2.912849898	-0.531871044	-1.834204263
H	-3.775816467	1.458644068	-1.777337179
H	-4.940340903	0.159427629	-1.480476833
H	-4.410737420	1.377789470	0.633818518
H	-4.938169706	-1.062110544	0.751159637
H	-3.779062553	-0.605366699	2.006999582
H	-3.053712606	-2.696800129	0.860818459
H	-4.142680844	-2.253490494	-1.342767302
H	-2.426759700	-2.629442957	-1.552167380
H	-3.062146146	-0.641669347	-2.917849665
C	-1.487650169	-0.044685117	-1.569347433
H	-1.301263602	0.919713607	-2.060540705
H	-0.756919233	-0.761486195	-1.978153708
C	-2.271600824	1.131374061	0.496945934
H	-2.091407163	2.105482175	0.024009006
H	-2.107112221	1.262535020	1.578393152
C	-1.481205245	-1.235852743	0.626060804
H	-0.766553697	-1.981424467	0.238846162
H	-1.319231457	-1.147070478	1.713122197
C	-1.250074373	0.120193798	-0.055745509
C	0.141827130	0.719537037	0.125784732
O	0.425110553	1.804156561	-0.365407060
N	1.048331070	0.005437344	0.849639565
C	2.401233283	0.492816702	1.038475358
H	2.347879727	1.580014589	1.180352666
H	2.806808529	0.058352197	1.962388383
C	3.315703150	0.180971438	-0.137598425
H	2.885261083	0.633211715	-1.039890144
H	3.340841614	-0.907768615	-0.301439520
C	4.733241856	0.697603057	0.100516715
H	5.321306702	0.552485201	-0.823902651
H	4.705637560	1.786718747	0.255204127
C	5.396974529	0.034894322	1.251353765
H	5.287512863	-1.039358523	1.395014759
H	6.113935974	0.553536327	1.880573039
H	0.795390759	-0.896226248	1.222099914

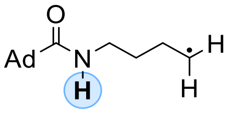

Conformer 11

C	-4.441608418	-0.367186220	-0.592964016
C	-3.749571254	-0.074856182	0.742084609
C	-3.670014922	-1.362658906	1.567017496
C	-2.870336308	-2.415970972	0.795038222
C	-3.564607539	-2.708574722	-0.538092104
C	-3.643765211	-1.422529047	-1.365438898
H	-4.518709096	0.555290409	-1.187085495
H	-5.467395140	-0.724029008	-0.415461022
H	-4.319753245	0.684722183	1.295844460
H	-4.681282063	-1.741268846	1.778348320
H	-3.192869718	-1.161542874	2.538312814
H	-2.803058057	-3.338976863	1.388824430
H	-4.574255857	-3.105849375	-0.355190624
H	-3.010140899	-3.481399216	-1.091876539
H	-4.137689401	-1.630083595	-2.325293247
C	-2.230934149	-0.899159064	-1.628569485
H	-2.252852759	0.018708716	-2.230443860
H	-1.648309369	-1.642763579	-2.195973427
C	-2.337175317	0.449675201	0.477190054
H	-2.363890210	1.378473781	-0.107855321
H	-1.829610024	0.679733203	1.427881877
C	-1.455262170	-1.887472366	0.530764076
H	-0.870901693	-2.655160028	-0.002619687
H	-0.957776554	-1.695943660	1.496585527
C	-1.515025782	-0.597251194	-0.299223645
C	-0.164566377	0.034240040	-0.625788667
O	-0.098276949	1.060978363	-1.288870997
N	0.953664099	-0.580986059	-0.148512582
C	2.268253110	-0.014224993	-0.377898875
H	3.013990333	-0.818614358	-0.315003906
H	2.292567718	0.375229054	-1.404118526
C	2.616591871	1.102384916	0.595750863
H	2.588131228	0.713039971	1.625378135
H	1.847453952	1.881266408	0.522800748
C	3.997016511	1.686146410	0.302516860
H	4.011669203	2.088386448	-0.722030352
H	4.158103373	2.562390419	0.956918391
C	5.090006042	0.697498564	0.481400655
H	5.052169144	-0.005846069	1.312173666
H	6.021729761	0.771111860	-0.071321797
H	0.868304140	-1.411445214	0.415840480

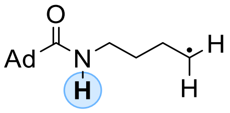

Conformer 12

C	-4.669306093	-0.088214136	0.090063015
C	-4.133978669	-0.938588642	-1.065694896
C	-4.242051258	-0.150927385	-2.373496342
C	-3.415640999	1.132411514	-2.262434472
C	-3.947628705	1.984462532	-1.105272816
C	-3.845155546	1.196984488	0.205113058
H	-4.616257477	-0.656610804	1.030651231
H	-5.728584778	0.158644517	-0.077421706
H	-4.719857053	-1.865500149	-1.143570117
H	-5.293403822	0.095958235	-2.585111862
H	-3.879269684	-0.762400922	-3.213456982
H	-3.483850811	1.700646332	-3.201234373
H	-4.994528230	2.264371164	-1.295203463
H	-3.373819910	2.921142264	-1.030676524
H	-4.224335666	1.809261890	1.035893908
C	-2.379841907	0.841330995	0.470925314
H	-2.284095830	0.281564873	1.414069535
H	-1.784317609	1.760498009	0.584894033
C	-2.669904114	-1.290022315	-0.801364802
H	-2.566010521	-1.875585747	0.121315064
H	-2.268106733	-1.919497376	-1.608434584
C	-1.948572798	0.780049385	-1.997023059
H	-1.365573217	1.713358637	-1.954029810
H	-1.537430709	0.187097029	-2.828410314
C	-1.822236194	-0.016256595	-0.683479712
C	-0.375894858	-0.412621516	-0.376263538
O	-0.062778809	-1.551280941	-0.064546999
N	0.539882296	0.600679995	-0.444768982
C	1.946838338	0.406052043	-0.165744713
H	2.090828149	-0.672151202	-0.030077527
H	2.547847317	0.705118793	-1.039567564
C	2.415854407	1.163022010	1.067437728
H	1.857986879	0.794875141	1.941226069
H	2.171622822	2.231307362	0.967540490
C	3.917288245	0.992539458	1.294141185
H	4.467690962	1.397189031	0.430262723
H	4.218572374	1.625311574	2.149180845
C	4.300799204	-0.422761298	1.522250111
H	5.267877088	-0.808931994	1.215692191
H	3.684913930	-1.057345287	2.156510181
H	0.229972370	1.524042441	-0.710493815

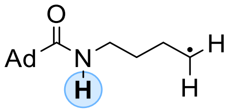

Conformer 13

C	-3.166885230	-0.874365356	-0.862200304
C	-3.303951482	0.485037757	-0.171029443
C	-3.751275302	0.277496532	1.279343480
C	-2.711223059	-0.567901242	2.021287785
C	-2.572437820	-1.926174016	1.327400469
C	-2.125458018	-1.717901988	-0.122999765
H	-2.866215013	-0.736138934	-1.911834950
H	-4.134835406	-1.397815016	-0.870560795
H	-4.046840829	1.096788395	-0.702502894
H	-4.731151973	-0.222938168	1.305080481

H	-3.871922126	1.249934876	1.779212813
H	-3.028844015	-0.716256521	3.063430786
H	-3.532827910	-2.462698603	1.351418416
H	-1.842105072	-2.552228767	1.862653159
H	-2.015639851	-2.692436624	-0.620106207
C	-0.772485452	-0.996858501	-0.132778986
H	-0.417397082	-0.866241049	-1.168895370
H	-0.041111377	-1.628334897	0.398862309
C	-1.953841533	1.202963423	-0.190510179
H	-1.622372031	1.364797257	-1.228504998
H	-2.021151362	2.191307169	0.280901414
C	-1.363013798	0.156040122	2.004024468
H	-0.605101863	-0.429771843	2.549098139
H	-1.436537468	1.133447628	2.501315211
C	-0.892276033	0.373457382	0.551111254
C	0.402388031	1.183140073	0.607372371
O	0.387048191	2.375834731	0.878475857
N	1.568978078	0.495767627	0.414651257
C	2.838554263	1.188244631	0.306940462
H	2.716557610	2.133129250	0.848238408
H	3.609523035	0.604847539	0.827762121
C	3.267765796	1.458948612	-1.130870516
H	3.426158392	0.503689703	-1.658520984
H	4.247966470	1.954337614	-1.101654535
C	2.276097610	2.315878545	-1.922589681
H	1.959189827	3.169951672	-1.307356879
H	2.810246197	2.751191861	-2.787586661
C	1.081774422	1.573020515	-2.401591043
H	0.155021075	2.098801243	-2.615817254
H	1.204662597	0.587239582	-2.850481173
H	1.514712321	-0.433810020	0.024178188

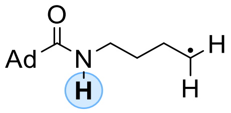

Conformer 14

C	-2.924101307	-1.680861115	-0.396806560
C	-2.668834862	-1.113950596	-1.796001843
C	-3.342528736	0.255494140	-1.921716151
C	-2.760785685	1.208246206	-0.873934937
C	-3.010424589	0.640309913	0.526828680
C	-2.341126622	-0.731775703	0.653167468
H	-2.463404600	-2.675667875	-0.302840272
H	-4.004076877	-1.809229510	-0.228582319
H	-3.079633193	-1.798258639	-2.551874369
H	-4.429683136	0.159727424	-1.780095872
H	-3.183803644	0.662528112	-2.931564567
H	-3.237625022	2.194984681	-0.962843617
H	-4.091661354	0.549465178	0.709380762
H	-2.613193888	1.326726516	1.290558103
H	-2.517574566	-1.137517424	1.659835188
C	-0.833292023	-0.583690006	0.429779945
H	-0.332163831	-1.559564554	0.529664203
H	-0.406881130	0.070262952	1.208458191
C	-1.162971112	-0.964401858	-2.017117405
H	-0.659323155	-1.938626682	-1.952420419
H	-0.951731103	-0.578849363	-3.024122571
C	-1.253326063	1.357784428	-1.096891417
H	-0.841696070	2.067405359	-0.362966011
H	-1.052109368	1.781804045	-2.092605154
C	-0.558726250	-0.013009305	-0.975716978
C	0.944997236	0.107126602	-1.226494181
O	1.518033945	-0.502174320	-2.117539492
N	1.616573148	0.960146137	-0.395684235
C	3.056219732	1.110391018	-0.480585354
H	3.321779168	0.961258237	-1.532130806
H	3.314969110	2.144797700	-0.212315780
C	3.835689285	0.132655950	0.391403181
H	4.899986172	0.207405679	0.128736082
H	3.520145517	-0.889691426	0.135873286
C	3.667653846	0.378768060	1.892383379
H	4.426784346	-0.221685645	2.427591078
H	3.918073031	1.426104596	2.121345263
C	2.314404505	0.048882663	2.408147962
H	1.870578910	0.598996742	3.234509805
H	1.868452771	-0.911648784	2.153440437
H	1.146035048	1.333238286	0.417050671

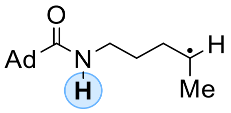

Conformer 1

C	-2.421163321	1.353484460	-1.993279784
C	-2.595706905	0.007957159	-1.283735694
C	-2.820105680	-1.092823242	-2.325385093
C	-1.613565974	-1.164475894	-3.267014529
C	-1.439013487	0.183149677	-3.973617046
C	-1.213137621	1.282622998	-2.931635032
H	-2.279449168	2.156019908	-1.253353378
H	-3.327478332	1.601257528	-2.565877091
H	-3.459512511	0.056943724	-0.605447083
H	-3.735707009	-0.886198931	-2.899955008
H	-2.964867355	-2.061344317	-1.824422463
H	-1.773432926	-1.955363942	-4.013562515
H	-2.330770537	0.415289836	-4.574977832
H	-0.586028418	0.138824314	-4.667828026
H	-1.077981992	2.249954173	-3.436282450
C	0.046153365	0.963246597	-2.116947943
H	0.208988574	1.765999796	-1.377028671
H	0.920224889	0.941052264	-2.788679355
C	-1.337492474	-0.310155660	-0.475082783
H	-1.165703457	0.465261168	0.290363537
H	-1.438260403	-1.266887378	0.053955389
C	-0.354144920	-1.481541916	-2.457855329
H	0.520749572	-1.548841959	-3.124102512
H	-0.444215764	-2.448843039	-1.946059869

C	-0.109344320	-0.387362343	-1.403003210
C	1.075577166	-0.810712257	-0.539774504
O	1.058659428	-1.867424150	0.076952546
N	2.137570210	0.040520833	-0.472542766
C	3.220866399	-0.195521168	0.466332923
H	3.481895134	-1.258209755	0.415930917
H	4.091388317	0.381518170	0.126784142
C	2.849297481	0.184488283	1.893757923
H	3.702582492	-0.025772449	2.553971235
H	2.028412783	-0.467693516	2.223099396
C	2.435987624	1.644635430	2.043553161
H	3.235843410	2.305577813	1.660131428
H	1.569323157	1.834939391	1.374527666
C	2.106352473	2.023346972	3.440395476
H	1.752580687	1.244096555	4.116862405
H	2.093386338	0.927778201	-0.948553947
C	1.982087705	3.441009034	3.859132863
H	0.956343036	3.829509077	3.734398008
H	2.238087997	3.580163532	4.916815418
H	2.638714964	4.089484720	3.264249781

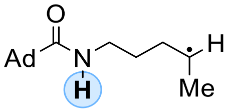

Conformer 2

C	-3.669610885	-1.164394427	0.458477891
C	-2.350171206	-1.787898713	-0.007743126
C	-2.419338642	-2.058725594	-1.513395213
C	-2.655186585	-0.742123568	-2.259216128
C	-3.975610420	-0.121627816	-1.794239523
C	-3.909269348	0.151347319	-0.288961388
H	-3.637082212	-0.981109440	1.542554940
H	-4.501207518	-1.860978110	0.273237326
H	-2.175279940	-2.730648840	0.530164656
H	-3.232719498	-2.765910164	-1.734721423
H	-1.484379680	-2.526793699	-1.857465500
H	-2.694867180	-0.931470034	-3.341471183
H	-4.809478452	-0.802611388	-2.021522654
H	-4.166964976	0.814627532	-2.340245870
H	-4.854450377	0.600573222	0.047658657
C	-2.760432591	1.118319730	0.001737277
H	-2.692882332	1.344648617	1.073560033
H	-2.926065194	2.074909105	-0.519094547
C	-1.201397140	-0.820773392	0.284444789
H	-1.121151933	-0.615622375	1.360490899
H	-0.241806937	-1.260527131	-0.034792530
C	-1.500856643	0.223482419	-1.963275897
H	-1.649002386	1.161059209	-2.523612135
H	-0.559658291	-0.233079287	-2.313779370
C	-1.421742703	0.512961332	-0.457635370
C	-0.301016430	1.455488337	-0.024579085
O	-0.207886855	1.816454662	1.140570514
N	0.590657582	1.846175627	-0.980255391
C	1.763827908	2.637819499	-0.656846388

H	1.883165229	3.423779100	-1.413746157
H	1.549506582	3.126300186	0.300674085
C	3.031436863	1.799945480	-0.552748218
H	3.880803466	2.471793475	-0.358261066
H	3.242056007	1.317843581	-1.520765504
C	2.965877232	0.735056384	0.535691079
H	2.704492958	1.223030165	1.495770917
H	2.112863235	0.059679635	0.341496623
C	4.210871733	-0.064610898	0.654226966
H	5.143099807	0.350609610	0.267344427
H	0.496396579	1.489740406	-1.919187790
C	4.267938821	-1.317776996	1.447547631
H	3.260191927	-1.672770901	1.695498430
H	4.784406072	-2.125681306	0.909267033
H	4.809378443	-1.189802417	2.398724948

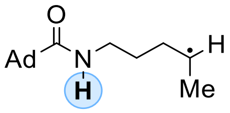

Conformer 3

C	-3.513535653	1.093319531	-2.199600809
C	-2.508279361	2.005981981	-1.491868235
C	-2.870712735	2.110613307	-0.007676851
C	-2.826381941	0.718308712	0.628363322
C	-3.829518126	-0.197159677	-0.080316270
C	-3.471254829	-0.300219137	-1.566338906
H	-3.276442667	1.028595271	-3.272431729
H	-4.525369152	1.518150918	-2.119146719
H	-2.528459913	3.005538291	-1.949359288
H	-3.874834560	2.546537599	0.103440945
H	-2.169285951	2.785053043	0.506851012
H	-3.080728465	0.790805196	1.695451994
H	-4.848989005	0.200485747	0.036341033
H	-3.817846770	-1.196182724	0.379813844
H	-4.188975119	-0.959611594	-2.074816183
C	-2.063675985	-0.880963859	-1.710738145
H	-1.796779464	-0.973319718	-2.775639255
H	-2.002442134	-1.887240819	-1.277001986
C	-1.096598033	1.422337325	-1.630087228
H	-0.816867187	1.375550011	-2.695106992
H	-0.385400763	2.097690060	-1.124721545
C	-1.418960906	0.136086144	0.485006313
H	-1.354697333	-0.860475514	0.942375564
H	-0.686007284	0.778549859	1.000809714
C	-1.035951793	0.021460958	-1.004337333
C	0.323837434	-0.671401902	-1.064944815
O	0.453423539	-1.822639687	-0.672657785
N	1.372487328	0.055224795	-1.548923101
C	2.731729981	-0.453213102	-1.534003658
H	2.649222894	-1.542888146	-1.449754941
H	3.208613849	-0.227828153	-2.496804620
C	3.558130069	0.107476076	-0.385282535
H	3.627475288	1.203543194	-0.475419349
H	4.587739030	-0.269125794	-0.477027135

C	3.006087053	-0.250122890	0.988967542
H	1.966249557	0.116537679	1.077795476
H	2.903312547	-1.351384076	1.057952902
C	3.817792677	0.287635562	2.109044855
H	4.832537110	0.634937608	1.909299006
H	1.225864169	1.019377638	-1.807233145
C	3.352344012	0.201407379	3.515216923
H	2.255573120	0.217990156	3.569108815
H	3.676213487	-0.730323848	4.010306811
H	3.734884630	1.027758342	4.126967982

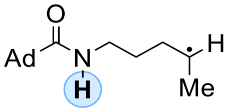

Conformer 4

C	-3.587419101	-1.989694556	-0.491326073
C	-3.183845508	-0.868126141	-1.452700941
C	-4.018689323	0.381953448	-1.156949493
C	-3.775068913	0.832916501	0.286623428
C	-4.177712336	-0.290750591	1.245974837
C	-3.340888922	-1.538960943	0.951231815
H	-3.008460512	-2.900342024	-0.707938210
H	-4.649275573	-2.244011543	-0.627517210
H	-3.355400528	-1.190408403	-2.489615251
H	-5.087264464	0.167379372	-1.309366131
H	-3.748493903	1.188815651	-1.853996343
H	-4.371069628	1.731928610	0.499481834
H	-5.247578417	-0.521507201	1.132645286
H	-4.026718879	0.028916906	2.288362662
H	-3.620793179	-2.344854600	1.644834129
C	-1.854714519	-1.210683236	1.139829253
H	-1.252458426	-2.115742789	0.955113643
H	-1.687117677	-0.902014100	2.185258761
C	-1.700467551	-0.545385335	-1.264138821
H	-1.086030682	-1.432321077	-1.487580448
H	-1.376892740	0.249133754	-1.948882063
C	-2.291735961	1.156571114	0.473593191
H	-2.101190119	1.496962532	1.504251966
H	-1.976783412	1.966953622	-0.197487248
C	-1.431029062	-0.088449752	0.181118982
C	0.032082177	0.337964257	0.277307418
O	0.495435733	1.169119119	-0.492063316
N	0.784411996	-0.218789677	1.267830979
C	2.182662224	0.130018226	1.432368385
H	2.285417891	1.196375232	1.195218087
H	2.449534908	-0.001029218	2.490151495
C	3.113775862	-0.681318484	0.545295065
H	2.828454465	-0.512016975	-0.502396303
H	2.975903439	-1.755106428	0.742092134
C	4.575825106	-0.304433870	0.754289687
H	4.684264555	0.786452479	0.583008535
H	4.851860624	-0.446365683	1.814857865
C	5.513070243	-1.070878075	-0.105081285
H	5.156604272	-1.422147002	-1.074265918
H	0.389746039	-0.940666046	1.850016444
C	6.965404547	-1.134492013	0.191539100
H	7.147506780	-1.190407133	1.273745267
H	7.441744314	-2.003033243	-0.277577417
H	7.508327469	-0.243512615	-0.169825504

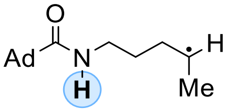

Conformer 5

C	-3.265501969	0.759033103	-0.092434780
C	-3.078774890	0.349798690	1.371949665
C	-3.859399345	-0.939198068	1.642769673
C	-3.338622986	-2.052342505	0.728782740
C	-3.531021926	-1.642960805	-0.734256538
C	-2.751238598	-0.354655478	-1.010002960
H	-2.725528466	1.696811255	-0.295884544
H	-4.329235532	0.952596470	-0.296481842
H	-3.445701467	1.151613547	2.028885706
H	-4.932877540	-0.772262777	1.466686558
H	-3.747865412	-1.233123174	2.697150789
H	-3.891257879	-2.981547920	0.927321333
H	-4.599015618	-1.488060055	-0.950032154
H	-3.180954533	-2.445878888	-1.400218984
H	-2.882461581	-0.058088442	-2.060670838
C	-1.262463006	-0.586553703	-0.736120956
H	-0.702204407	0.334607351	-0.966437442
H	-0.866460351	-1.372574167	-1.398385779
C	-1.590185349	0.116996754	1.647397801
H	-1.035855333	1.054241420	1.485189404
H	-1.432848903	-0.164211322	2.699853230
C	-1.851038473	-2.282899118	1.000650753
H	-1.458912009	-3.092478762	0.369883479
H	-1.687546369	-2.600268071	2.039765089
C	-1.050136310	-1.001341203	0.733928086
C	0.429823772	-1.272196740	1.005226119
O	0.855504623	-2.372285764	1.326570651
N	1.261532253	-0.198435281	0.872434070
C	2.701952522	-0.343692799	0.978092169
H	3.116723619	0.654478788	1.166855294
H	2.915331598	-0.967645146	1.853069238
C	3.338440551	-0.955112384	-0.267980689
H	3.079402507	-2.019972877	-0.314798607
H	4.431594860	-0.894681172	-0.162579822
C	2.899006105	-0.258318842	-1.556757748
H	3.442389838	-0.713286917	-2.405563603
H	1.836089100	-0.481365525	-1.747124569
C	3.084202037	1.217474314	-1.523560954
H	4.014117297	1.609778309	-1.105425705
H	0.902803673	0.670436477	0.502528088
C	2.207757253	2.148080403	-2.276480067
H	2.573861552	2.338459514	-3.300347321
H	1.192442396	1.740185984	-2.379932978
H	2.134774059	3.130324004	-1.791810087

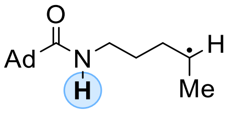

Conformer 6

C	-4.517549276	-0.831904465	1.602851767
C	-3.335630580	-1.687280526	1.138233934
C	-3.449317698	-1.935118980	-0.369370747
C	-3.438744519	-0.596345618	-1.114781688
C	-4.621472635	0.255685913	-0.647004472
C	-4.503127076	0.506627199	0.858599318
H	-4.455701390	-0.660423204	2.687832924
H	-5.462882057	-1.362585504	1.413634485
H	-3.339587562	-2.648337704	1.672448461
H	-4.378084247	-2.481485640	-0.591804168
H	-2.616138133	-2.567518783	-0.713283222
H	-3.514530099	-0.775633720	-2.196909615
H	-5.567555132	-0.258058813	-0.875098432
H	-4.635667841	1.213006359	-1.189306078
H	-5.346153178	1.125475041	1.197251722
C	-3.192693867	1.237938862	1.151993982
H	-3.091242949	1.449461132	2.224170700
H	-3.169637024	2.213254992	0.644644124
C	-2.024912592	-0.953242248	1.435022941
H	-1.919163657	-0.772950785	2.515791021
H	-1.168846353	-1.575637683	1.130332176
C	-2.124994011	0.136015140	-0.819488629
H	-2.084738723	1.090798480	-1.365835228
H	-1.289681700	-0.477783476	-1.192118662
C	-1.992281535	0.398887336	0.692164000
C	-0.704809140	1.140654268	1.058568478
O	-0.695843502	2.102801023	1.811175174
N	0.445399662	0.639984706	0.517707958
C	1.746458295	1.212000217	0.796517638
H	1.568294251	2.222136429	1.184159678
H	2.254927780	0.654016467	1.600585818
C	2.623098174	1.249887062	-0.441840553
H	2.120771335	1.839222962	-1.223251203
H	2.745147770	0.234363312	-0.852059166
C	3.999699793	1.840156460	-0.155058863
H	3.858097120	2.844253191	0.294852008
H	4.501184457	1.250492548	0.631197088
C	4.872363767	1.914364213	-1.355115052
H	4.400815527	2.079670753	-2.325380755
H	0.400560293	-0.165602184	-0.089500486
C	6.351191458	2.000070512	-1.263486910
H	6.687697637	1.956913718	-0.220794061
H	6.849069097	1.178775135	-1.802264787
H	6.744334436	2.930304712	-1.700101650

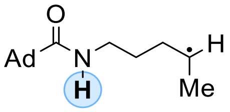

Conformer 7

C	-2.517333172	-2.405236729	-0.077696534
C	-2.620394624	-1.613473808	-1.384199201
C	-3.836537772	-0.684941641	-1.324638657
C	-3.681533143	0.289405361	-0.153292959
C	-3.575829891	-0.502429959	1.153497975
C	-2.361008596	-1.433583806	1.094895809
H	-1.657938738	-3.091832450	-0.117271264
H	-3.416927712	-3.023971190	0.058252523
H	-2.724361313	-2.306595882	-2.231270012
H	-4.755188376	-1.278736801	-1.205137242
H	-3.934416112	-0.129100204	-2.269564152
H	-4.552609220	0.958692810	-0.111354455
H	-4.493017099	-1.088982672	1.314371071
H	-3.480327944	0.187031046	2.005273421
H	-2.281279095	-1.997478639	2.035233173
C	-1.094352087	-0.599586735	0.900205044
H	-0.206665353	-1.253586457	0.872576498
H	-0.950075268	0.100197522	1.733896076
C	-1.350790266	-0.775744296	-1.576850298
H	-0.477204971	-1.446758625	-1.637398295
H	-1.423478807	-0.228367076	-2.531503864
C	-2.413519525	1.123404934	-0.347318408
H	-2.280470089	1.837941006	0.476236250
H	-2.481115538	1.710274349	-1.277397105
C	-1.176978412	0.207471090	-0.410045477
C	0.065436843	1.094282160	-0.476514841
O	0.346408534	1.838098353	0.453935980
N	0.824805038	1.016722286	-1.606437069
C	2.123158228	1.653894247	-1.714778281
H	2.169533818	2.245712323	-2.638491454
H	2.196182688	2.344654513	-0.866316829
C	3.255202133	0.634774054	-1.668794366
H	4.209436214	1.166958907	-1.798096659
H	3.170031377	-0.054054641	-2.523425781
C	3.277020804	-0.151090128	-0.357674769
H	2.316334705	-0.680727407	-0.232063264
H	4.037012114	-0.950593352	-0.433521175
C	3.525557742	0.717530159	0.824385457
H	4.320671566	1.460794300	0.747761807
H	0.583632506	0.327598526	-2.303525462
C	2.866281803	0.468702620	2.128626589
H	1.775251482	0.595489890	2.038792252
H	3.039817800	-0.558084909	2.492551123
H	3.212221178	1.157246699	2.905660513

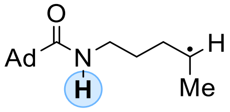

Conformer 8

C	-3.348875432	0.351440305	-1.789720580
C	-3.502974292	0.598228059	-0.285562053
C	-3.900602686	-0.708622575	0.406615891
C	-2.816795207	-1.764016676	0.168732031
C	-2.666596028	-2.011298262	-1.334978005

C	-2.266664559	-0.706402175	-2.029121079
H	-3.079168123	1.288184125	-2.299320814
H	-4.305579016	0.014994162	-2.217048110
H	-4.278066968	1.358434050	-0.112769743
H	-4.864688641	-1.067447706	0.016036456
H	-4.034242150	-0.539765913	1.485993254
H	-3.094162786	-2.700757862	0.673025264
H	-3.613767527	-2.387424419	-1.749719554
H	-1.905921979	-2.785603155	-1.517833981
H	-2.152265243	-0.880915108	-3.108472581
C	-0.937970478	-0.211004219	-1.456008625
H	-0.616881412	0.718680196	-1.944435101
H	-0.144116731	-0.957628016	-1.631308259
C	-2.173775397	1.094213458	0.286906281
H	-1.866187254	2.036875128	-0.184497740
H	-2.272591148	1.290225319	1.366631694
C	-1.483635771	-1.265063338	0.740121681
H	-0.710663948	-2.036402912	0.580224218
H	-1.588792894	-1.112798434	1.827219137
C	-1.068436455	0.046274553	0.057989096
C	0.255824773	0.645368495	0.529932400
O	0.695007100	1.663246716	0.011802417
N	0.908790024	-0.006000535	1.535547095
C	2.248520019	0.362155049	1.949578214
H	2.449678227	1.331104163	1.479404157
H	2.276000749	0.504821013	3.038255037
C	3.287731147	-0.668622330	1.519363103
H	3.137279181	-1.608071585	2.072901214
H	4.280830103	-0.296905166	1.812125782
C	3.259506149	-0.945050481	0.016081126
H	4.023235968	-1.711926247	-0.213317315
H	2.296114246	-1.413510180	-0.251105456
C	3.447456531	0.284784276	-0.797775906
H	4.182485745	1.013560248	-0.448084421
H	0.518189075	-0.863983471	1.895452805
C	2.852506374	0.476003448	-2.141139995
H	3.594100410	0.416142253	-2.954216872
H	2.084106338	-0.281210003	-2.347155170
H	2.368276916	1.459269046	-2.224481241

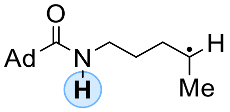

Conformer 9

C	-4.074411867	-0.475167820	1.565985916
C	-3.210043650	-1.582724103	0.955043176
C	-3.791249586	-1.993374723	-0.400900624
C	-3.809297043	-0.781826832	-1.337391440
C	-4.674816450	0.323654807	-0.725964427
C	-4.094201848	0.736998386	0.629440903
H	-3.675269103	-0.185058680	2.549049806
H	-5.098889961	-0.842746369	1.728908769
H	-3.191956605	-2.451701366	1.628246652
H	-4.811919067	-2.384409644	-0.274202063
H	-3.189400980	-2.803112079	-0.841029179
H	-4.218266065	-1.074793412	-2.315037658
H	-5.708211615	-0.033995401	-0.603576829
H	-4.713247347	1.191557264	-1.401855647
H	-4.711548387	1.533722513	1.068518188
C	-2.667289552	1.253730528	0.436297423
H	-2.226694776	1.567411177	1.391853295
H	-2.666789961	2.135348069	-0.224681257
C	-1.783743909	-1.065185881	0.762021698
H	-1.336219470	-0.770838150	1.720433966
H	-1.144550956	-1.855717395	0.336680659
C	-2.378999786	-0.262897378	-1.528560406
H	-2.393808519	0.595941764	-2.220073562
H	-1.764612867	-1.055214881	-1.987991050
C	-1.779181384	0.155051534	-0.178148706
C	-0.349987672	0.690232898	-0.234012722
O	0.230948847	1.036915917	0.786616820
N	0.245592538	0.770515167	-1.456284024
C	1.610519965	1.245219220	-1.581555325
H	1.753799871	1.629275606	-2.600414950
H	1.738170795	2.089672143	-0.891580979
C	2.646787506	0.176421983	-1.262257055
H	2.582032074	-0.637263411	-1.998126898
H	2.399233280	-0.255509271	-0.282506792
C	4.055806658	0.758807231	-1.217304181
H	4.784604145	-0.067541478	-1.114344163
H	4.301347219	1.227805184	-2.187877032
C	4.229319084	1.752493830	-0.124181423
H	3.584106638	1.677610979	0.752642362
H	-0.243630213	0.448736063	-2.276199455
C	5.292167665	2.787061307	-0.175689307
H	5.603562905	3.109059791	0.824016700
H	6.183110470	2.419937929	-0.705478027
H	4.965589703	3.693937054	-0.713348813

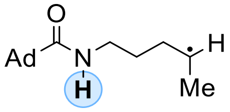

Conformer 10

C	-3.910461177	0.478184360	-1.280380088
C	-3.698658764	0.642977432	0.227922283
C	-3.909353804	-0.705333686	0.922624674
C	-2.907269623	-1.724988000	0.374128628
C	-3.121539638	-1.889734603	-1.133023188
C	-2.910355051	-0.543332976	-1.830783100
H	-3.777976864	1.445404505	-1.787190193
H	-4.939705924	0.145322905	-1.483000198
H	-4.413840393	1.378261074	0.623579299
H	-4.936601431	-1.062547215	0.755255469
H	-3.780679716	-0.595088048	2.010165367
H	-3.049351474	-2.692377025	0.876891738
H	-4.137890896	-2.263328790	-1.328677034
H	-2.421864128	-2.638230602	-1.535395954
H	-3.059076605	-0.660702639	-2.913721200

C	-1.486209586	-0.051711380	-1.568203436
H	-1.301655426	0.910372581	-2.064167256
H	-0.753442208	-0.769108730	-1.971932181
C	-2.273868683	1.133639642	0.490591129
H	-2.094704195	2.105722343	0.012896172
H	-2.110589425	1.270278743	1.571647738
C	-1.478849060	-1.230734653	0.634646935
H	-0.764148300	-1.979178145	0.253065545
H	-1.318063186	-1.134407881	1.721207421
C	-1.249460194	0.121374638	-0.055808146
C	0.141923412	0.724376739	0.123418635
O	0.421272332	1.810250365	-0.367544049
N	1.050959806	0.011074760	0.844377257
C	2.403144734	0.500884232	1.033753808
H	2.346728520	1.587015647	1.182566620
H	2.810626155	0.061708548	1.954718967
C	3.320049632	0.199727187	-0.143109594
H	2.891520687	0.659434830	-1.042462616
H	3.345309475	-0.887302626	-0.317602657
C	4.736828390	0.713846936	0.105042683
H	5.324809712	0.590415357	-0.823878764
H	4.705920065	1.801325770	0.282854595
C	5.408367592	0.043297687	1.251798982
H	5.268386143	-1.033903545	1.366888835
H	0.801484844	-0.894057864	1.210478344
C	6.388140007	0.727709014	2.133790063
H	5.997706525	0.878907137	3.153647753
H	7.319255394	0.155309519	2.246783424
H	6.649841699	1.718101647	1.742518089

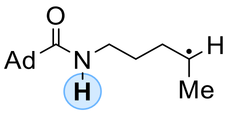

Conformer 11

C	-4.444461228	-0.378838138	-0.584583002
C	-3.754625832	-0.093730775	0.753194617
C	-3.667775086	-1.387351966	1.567919120
C	-2.860726002	-2.429485881	0.788030325
C	-3.553206957	-2.715111578	-0.547725631
C	-3.639994512	-1.423126267	-1.365106495
H	-4.526601136	0.548242445	-1.170849654
H	-5.468306469	-0.743171936	-0.410962243
H	-4.329371553	0.657908152	1.313093001
H	-4.677017134	-1.773298910	1.775934545
H	-3.192397771	-1.190630549	2.541016273
H	-2.788405655	-3.356800117	1.374236516
H	-4.560743483	-3.119539208	-0.368535587
H	-2.994702221	-3.480476319	-1.107848424
H	-4.132555206	-1.626379296	-2.326724411
C	-2.230205444	-0.889183135	-1.623930569
H	-2.258754809	0.033451249	-2.218341402
H	-1.643122337	-1.624336327	-2.197515087
C	-2.345348752	0.440652542	0.493464121
H	-2.376455119	1.374915850	-0.082545567
H	-1.840072030	0.665538042	1.446685518
C	-1.448062481	-1.890886512	0.527380930
H	-0.861837703	-2.651627156	-0.014354924
H	-0.949374098	-1.705670770	1.493553246
C	-1.515909778	-0.593657754	-0.291549376
C	-0.170969843	0.051035371	-0.619872530
O	-0.115443237	1.077374276	-1.284458684
N	0.953706717	-0.556589624	-0.149784295
C	2.265560363	0.011161740	-0.396628897
H	3.012525933	-0.792255558	-0.339257203
H	2.275120326	0.396534440	-1.424580748
C	2.624283369	1.134662446	0.565514394
H	2.585024959	0.757403192	1.599341169
H	1.862310822	1.919285283	0.480288124
C	4.013565352	1.697252603	0.275008512
H	4.048155883	2.070355629	-0.762059725
H	4.173570505	2.591942665	0.906291395
C	5.101959026	0.703089742	0.479665835
H	5.013907242	0.016858830	1.325198529
H	0.875895195	-1.382151897	0.422813313
C	6.388319554	0.748148080	-0.260848753
H	7.257058285	0.792065966	0.411606671
H	6.537258218	-0.143054150	-0.891477545
H	6.434624000	1.621397784	-0.922235881

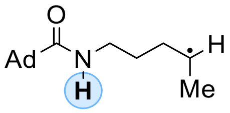

Conformer 12

C	-4.664452540	-0.081038727	0.102541181
C	-4.138029037	-0.934791129	-1.054890917
C	-4.253597210	-0.148641177	-2.363511079
C	-3.422431900	1.132366461	-2.260582083
C	-3.942621669	1.986836378	-1.100208440
C	-3.834170647	1.201018757	0.210363030
H	-4.608797095	-0.647393149	1.044104475
H	-5.723455048	0.169869559	-0.060777723
H	-4.726772766	-1.860210297	-1.127342634
H	-5.305661478	0.101678904	-2.567239182
H	-3.899466972	-0.762259863	-3.205443350
H	-3.495523299	1.700251759	-3.199297773
H	-4.989664617	2.270940570	-1.283075654
H	-3.365511856	2.921992324	-1.030286542
H	-4.206004563	1.816255995	1.042340760
C	-2.367722392	0.842003675	0.467239995
H	-2.266327466	0.283107622	1.410287608
H	-1.770801387	1.760915112	0.576568652
C	-2.672407103	-1.289912573	-0.800419191
H	-2.563730551	-1.874551817	0.122308385
H	-2.279000017	-1.921429981	-1.609877578
C	-1.954722917	0.776373036	-2.004371278
H	-1.368022873	1.707892639	-1.967689017

H	-1.551109248	0.181309771	-2.837835314
C	-1.820579282	-0.017769243	-0.691046660
C	-0.372323744	-0.415828389	-0.392442477
O	-0.055821539	-1.555972713	-0.090443509
N	0.541589486	0.599578188	-0.455047778
C	1.948855675	0.408031551	-0.173667850
H	2.100178576	-0.671635226	-0.059105117
H	2.551301333	0.730447734	-1.037908422
C	2.407055580	1.142342656	1.078072734
H	1.851457007	0.748663675	1.942063219
H	2.147598455	2.208978030	1.000876095
C	3.910200657	0.987092534	1.305327123
H	4.458298417	1.406389466	0.444788376
H	4.202289316	1.616933615	2.167088441
C	4.322125572	-0.428142447	1.507099418
H	3.681004787	-1.061103816	2.123973771
H	0.227592089	1.524509986	-0.710023319
C	5.653002627	-0.949126536	1.110769713
H	6.337620947	-1.056028453	1.968512050
H	5.582753807	-1.945963119	0.653406902
H	6.141828791	-0.283436842	0.389071717

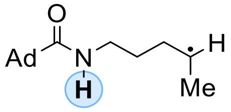

Conformer 13

C	-3.083382790	-1.043373362	-0.846224166
C	-3.250064944	0.373163951	-0.288969441
C	-3.774820731	0.298879140	1.148209547
C	-2.783086781	-0.478019193	2.019093990
C	-2.620162944	-1.894476677	1.460380906
C	-2.092284165	-1.820048962	0.024742688
H	-2.720478516	-1.000218157	-1.885094094
H	-4.052960315	-1.563362160	-0.865161055
H	-3.959157348	0.935976918	-0.912765400
H	-4.758555410	-0.194332172	1.166927328
H	-3.914789130	1.313256782	1.550054231
H	-3.157440155	-0.528185446	3.051591597
H	-3.585349753	-2.422565315	1.478522574
H	-1.925805361	-2.472750807	2.088977650
H	-1.963701303	-2.836045124	-0.375547688
C	-0.735015304	-1.106622734	0.020823265
H	-0.334056271	-1.078546716	-1.005581436
H	-0.031688612	-1.686049043	0.642334543
C	-1.896444508	1.083710051	-0.298662529
H	-1.511056287	1.155290973	-1.328368056
H	-1.981844724	2.109492054	0.082645601
C	-1.429449739	0.235901001	2.008926945
H	-0.707458813	-0.303181517	2.643486615
H	-1.518158231	1.253887651	2.412468428
C	-0.880660033	0.320230145	0.570466699
C	0.408892865	1.138339185	0.618824357
O	0.383592211	2.329747309	0.895087983
N	1.574412817	0.468695371	0.381041501
C	2.849446832	1.153174722	0.295491436
H	2.712728229	2.115745936	0.801819676
H	3.598765143	0.583849400	0.862146233
C	3.328600463	1.369287691	-1.134836659
H	3.411354691	0.397534784	-1.647436873
H	4.350425334	1.771371625	-1.093962636
C	2.447656447	2.324636488	-1.950619097
H	2.338199767	3.262466385	-1.389390792
H	3.002811140	2.580217449	-2.873405809
C	1.097742344	1.801772181	-2.314769238
H	0.237876558	2.451967047	-2.165538679
H	1.529859807	-0.489508275	0.067599560
C	0.947994242	0.599415580	-3.174335932
H	1.560597022	-0.245547295	-2.822029264
H	-0.092165554	0.250942649	-3.222061679
H	1.269962202	0.788133673	-4.213642944

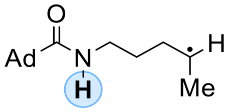

Conformer 14

C	-2.926241820	-1.685582682	-0.450830748
C	-2.681603574	-1.063661581	-1.828621172
C	-3.340723393	0.316793027	-1.889681432
C	-2.734042180	1.217739614	-0.810949797
C	-2.974880567	0.595747692	0.568594995
C	-2.320011175	-0.788017577	0.631452792
H	-2.476295450	-2.688660293	-0.405321614
H	-4.005591220	-1.808620992	-0.275088179
H	-3.109583508	-1.711958977	-2.606464574
H	-4.427137269	0.227185440	-1.738567388
H	-3.189421074	0.763897446	-2.883622844
H	-3.199043205	2.213213032	-0.853158584
H	-4.055037279	0.508938381	0.759207233
H	-2.561889088	1.246835841	1.354679351
H	-2.490477863	-1.233759330	1.622124727
C	-0.813345313	-0.647579136	0.395565824
H	-0.320484646	-1.631700335	0.449777755
H	-0.367447662	-0.025468540	1.189461864
C	-1.177092610	-0.923310929	-2.062139552
H	-0.683888622	-1.904376152	-2.044593213
H	-0.974148320	-0.498309999	-3.055392275
C	-1.226829085	1.358593907	-1.045834550
H	-0.802270328	2.034147010	-0.287401090
H	-1.031573250	1.823121920	-2.024447371
C	-0.548337634	-0.023368315	-0.989909597
C	0.957812952	0.067300855	-1.240801885
O	1.526713040	-0.609968310	-2.084258183
N	1.635971232	0.967476549	-0.465036466
C	3.083607206	1.050050572	-0.518454107
H	3.366925856	0.822193837	-1.551002586
H	3.380502936	2.088099186	-0.310776002
C	3.799693346	0.100377341	0.434311495

H	4.871759192	0.118288158	0.194795135
H	3.451689178	-0.923683057	0.232837559
C	3.602184694	0.444798501	1.911395871
H	4.345322065	-0.119653917	2.506704725
H	3.854487884	1.506295245	2.078860206
C	2.232336829	0.170163048	2.425422989
H	1.784390800	-0.788533733	2.155563665
H	1.174810751	1.349832163	0.349846803
C	1.679950842	0.905081093	3.591810144
H	0.596650779	0.767877641	3.690732475
H	2.128447461	0.573694480	4.545303202
H	1.884073990	1.982735991	3.519025900

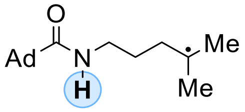

Conformer 1

C	-2.423085276	1.355130004	-1.838302952
C	-2.554402424	-0.002937455	-1.143237800
C	-2.819632583	-1.088385192	-2.191448890
C	-1.658476807	-1.134428888	-3.189881664
C	-1.526117717	0.225452367	-3.881604925
C	-1.259845220	1.309573885	-2.833065970
H	-2.253076842	2.147059067	-1.092914995
H	-3.357096818	1.604165313	-2.363883299
H	-3.385847520	0.028277759	-0.424675846
H	-3.763043367	-0.880141091	-2.718383505
H	-2.932839210	-2.066233952	-1.700563585
H	-1.846513679	-1.914913419	-3.940810715
H	-2.445962972	0.460476920	-4.437749319
H	-0.705526570	0.198555117	-4.614688070
H	-1.155670656	2.286013915	-3.327350006
C	0.038274981	0.987977670	-2.082717313
H	0.228037241	1.780384163	-1.337940364
H	0.881464496	0.983957193	-2.792896885
C	-1.257468859	-0.324416639	-0.399277667
H	-1.056370404	0.438445896	0.371720655
H	-1.326235041	-1.291330650	0.116275252
C	-0.360774494	-1.453377512	-2.445665859
H	0.482765418	-1.503033283	-3.152719502
H	-0.418995854	-2.429588443	-1.946326060
C	-0.074004444	-0.375173003	-1.385249012
C	1.153963440	-0.803972265	-0.587667907
O	1.178457247	-1.877297344	-0.000428801
N	2.208065493	0.059516305	-0.546642241
C	3.318835829	-0.175758574	0.359946288
H	3.614195975	-1.225970475	0.261036735
H	4.161641491	0.444165000	0.026621679
C	2.964285517	0.130229533	1.809422392
H	3.842212057	-0.074461738	2.439116076
H	2.179323729	-0.571475984	2.120474680
C	2.503690880	1.567900326	2.016901124
H	3.255802875	2.260763368	1.597408705
H	1.593715356	1.734489528	1.397780251
C	2.211452534	1.957712914	3.428491776
H	2.114691659	0.971826134	-0.965531344
C	2.048521967	3.411141922	3.709940674
H	1.064336151	3.787201073	3.373849102
H	2.117969973	3.629141565	4.782858566
H	2.804185353	4.013958585	3.188506929
C	1.588028089	0.972717372	4.355933254
H	0.551842149	0.726568919	4.057857948
H	2.130726123	0.019632452	4.387525594
H	1.539689714	1.366905177	5.377928491

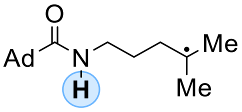

Conformer 2

C	-3.705055481	-1.114175609	0.446016964
C	-2.346120864	-1.752628061	0.142771779
C	-2.278123823	-2.125166249	-1.340470136
C	-2.452392530	-0.865018905	-2.191971446
C	-3.812181577	-0.229592973	-1.889085397
C	-3.884437547	0.145740598	-0.406436168
H	-3.771271151	-0.859114097	1.513985163
H	-4.513265374	-1.830771185	0.234831969
H	-2.216094566	-2.654668592	0.757597873
H	-3.064364268	-2.854778255	-1.586064820
H	-1.313564039	-2.604761451	-1.566398590
H	-2.395020967	-1.126755416	-3.258240912
H	-4.618564051	-0.934373767	-2.141748454
H	-3.959919839	0.664520915	-2.513721206
H	-4.859037506	0.605047516	-0.187749049
C	-2.771755933	1.143174096	-0.079419722
H	-2.803184659	1.437363594	0.978317862
H	-2.893879542	2.061920332	-0.675202352
C	-1.233939319	-0.755392465	0.469174349
H	-1.249297025	-0.476467066	1.530552043
H	-0.246766759	-1.205391839	0.269665884
C	-1.337533055	0.134732158	-1.861604780
H	-1.456517621	1.029763073	-2.494761675
H	-0.359652858	-0.318955004	-2.097747974
C	-1.393385541	0.522123931	-0.377112308
C	-0.326052489	1.510212314	0.086534443
O	-0.256179778	1.842133927	1.261724519
N	0.529030308	1.997612156	-0.860237950
C	1.696945614	2.777368708	-0.495885483
H	1.767618434	3.658897253	-1.146580818
H	1.515779823	3.131730534	0.525235570
C	2.981523320	1.958618182	-0.556496630
H	3.825912303	2.623366253	-0.325628468
H	3.147122279	1.602773850	-1.586744209
C	2.962952378	0.769584518	0.397211096
H	2.827840791	1.154469426	1.429625032
H	2.050877017	0.175470022	0.213116044
C	4.146498123	-0.136083355	0.325943782
H	0.497053141	1.607169477	-1.790433109

C	4.032274140	-1.466091225	0.987529754
H	3.031119255	-1.899884853	0.859000711
H	4.768547531	-2.180790835	0.596809216
H	4.204326345	-1.405126468	2.078088512
C	5.506484493	0.408638159	0.057426285
H	5.908726727	0.968868920	0.922267548
H	6.221466977	-0.395351289	-0.156108796
H	5.523200749	1.105522186	-0.792098324

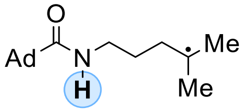

Conformer 3

C	-3.705055635	-1.114175473	0.446016896
C	-2.346121003	-1.752628008	0.142771951
C	-2.278123790	-2.125166331	-1.340469920
C	-2.452392336	-0.865019060	-2.191971375
C	-3.812181396	-0.229593043	-1.889085564
C	-3.884437542	0.145740660	-0.406436377
H	-3.771271438	-0.859113855	1.513985061
H	-4.513265523	-1.830771043	0.234831866
H	-2.216094827	-2.654668490	0.757598142
H	-3.064364238	-2.854778326	-1.586064625
H	-1.313564002	-2.604761602	-1.566398210
H	-2.395020646	-1.126755676	-3.258240808
H	-4.618563869	-0.934373821	-2.141748672
H	-3.959919534	0.664520799	-2.513721468
H	-4.859037512	0.605047636	-0.187749429
C	-2.771755928	1.143174139	-0.079419876
H	-2.803184768	1.437363725	0.978317677
H	-2.893879444	2.061920331	-0.675202595
C	-1.233939448	-0.755392436	0.469174576
H	-1.249297278	-0.476466957	1.530552248
H	-0.246766892	-1.205391912	0.269666280
C	-1.337532848	0.134731979	-1.861604658
H	-1.456517234	1.029762837	-2.494761664
H	-0.359652669	-0.318955318	-2.097747666
C	-1.393385510	0.522123914	-0.377112231
C	-0.326052515	1.510212329	0.086534584
O	-0.256179842	1.842133869	1.261724681
N	0.529030273	1.997612270	-0.860237775
C	1.696945583	2.777368799	-0.495885268
H	1.767618363	3.658897405	-1.146580523
H	1.515779830	3.131730524	0.525235825
C	2.981523279	1.958618288	-0.556496580
H	3.825912285	2.623366342	-0.325628458
H	3.147122132	1.602774018	-1.586744198
C	2.962952426	0.769584567	0.397211076
H	2.827841015	1.154469423	1.429625052
H	2.050877022	0.175470120	0.213116117
C	4.146498119	-0.136083368	0.325943524
H	0.497053159	1.607169591	-1.790432937
C	4.032274090	-1.466091330	0.987529273
H	3.031119244	-1.899884979	0.858999983
H	4.768547579	-2.180790853	0.596808770
H	4.204326077	-1.405126733	2.078088076
C	5.506484480	0.408638158	0.057426025
H	5.908726618	0.968869136	0.922267192
H	6.221467030	-0.395351289	-0.156108827
H	5.523200767	1.105522007	-0.792098723

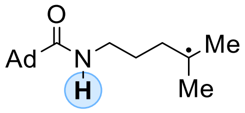

Conformer 4

C	-3.585378919	-2.019632813	-0.413475936
C	-3.191113907	-0.925528425	-1.409466064
C	-4.033874523	0.326950509	-1.150110888
C	-3.792287278	0.823835556	0.278320036
C	-4.185972195	-0.272429970	1.272129803
C	-3.340782295	-1.523225379	1.014395217
H	-3.000180239	-2.932439199	-0.602584110
H	-4.645423351	-2.285414632	-0.541563795
H	-3.362216292	-1.280575324	-2.435742409
H	-5.100994972	0.100481595	-1.295137440
H	-3.770257862	1.114061651	-1.871798157
H	-4.394003731	1.725089673	0.463824103
H	-5.254393216	-0.513850118	1.167641427
H	-4.035886668	0.080419601	2.303924819
H	-3.613832996	-2.309518508	1.732674516
C	-1.856042916	-1.180180076	1.189170333
H	-1.252000318	-2.088581582	1.026414147
H	-1.683229583	-0.840792944	2.224027685
C	-1.710358894	-0.586164557	-1.234034134
H	-1.089375676	-1.474512384	-1.432362978
H	-1.395264859	0.190733021	-1.942900643
C	-2.311101010	1.162701100	0.452860952
H	-2.121678562	1.535599269	1.472439299
H	-2.001839863	1.954708977	-0.242016721
C	-1.442201016	-0.084119809	0.196604278
C	0.018790523	0.353553959	0.272377473
O	0.466028650	1.183449479	-0.507998237
N	0.789456792	-0.196973194	1.251916880
C	2.192659967	0.143853161	1.380662199
H	2.295644102	1.209782424	1.141271003
H	2.485657000	0.011278338	2.431429664
C	3.096022710	-0.672439155	0.469285493
H	2.780656466	-0.497122310	-0.568087800
H	2.955118022	-1.745970452	0.669048675
C	4.564489523	-0.298919746	0.656339464
H	4.670560827	0.781012709	0.421497060
H	4.828554403	-0.388118221	1.722749851
C	5.522747210	-1.099852872	-0.163119505
H	0.408435323	-0.922388384	1.838832423
C	6.890316127	-1.362574186	0.357675648
H	6.889431260	-1.510697659	1.445508691
H	7.339549046	-2.251591092	-0.105179304
H	7.589278615	-0.529077411	0.158792691

C	5.224615074	-1.308928402	-1.608000306
H	6.050006383	-1.811215482	-2.125303936
H	4.320172708	-1.915584230	-1.767945378
H	5.038140960	-0.355031115	-2.132376643

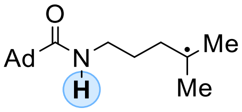

Conformer 5

C	-3.262869147	0.481680722	-0.608109951
C	-2.890239475	0.635604937	0.869115907
C	-3.678935339	-0.377490372	1.702381924
C	-3.338927131	-1.795412849	1.236535370
C	-3.708220460	-1.949562803	-0.241888891
C	-2.922069842	-0.935134052	-1.078127141
H	-2.720213795	1.222945466	-1.214838114
H	-4.336532114	0.678321955	-0.746569158
H	-3.123738052	1.655819288	1.205669763
H	-4.758184712	-0.190347009	1.597237977
H	-3.435406781	-0.261293067	2.769380718
H	-3.901958442	-2.525583221	1.835169882
H	-4.788835619	-1.792948229	-0.379302284
H	-3.485236030	-2.972022590	-0.580628322
H	-3.184168782	-1.046434720	-2.139932295
C	-1.423568850	-1.185891118	-0.899418256
H	-0.841597938	-0.469696538	-1.505016679
H	-1.149766614	-2.194437838	-1.238779380
C	-1.387546860	0.384385783	1.041420071
H	-0.838953039	1.131050564	0.441293588
H	-1.100384019	0.527864801	2.095406107
C	-1.841223329	-2.044504128	1.415915664
H	-1.565770450	-3.060010866	1.104696130
H	-1.563285377	-1.948791187	2.477478871
C	-1.028729241	-1.037427760	0.584332286
C	0.448689048	-1.408900265	0.688153829
O	0.824172746	-2.559236970	0.502053436
N	1.325566460	-0.404783431	0.957386493
C	2.762061353	-0.622735852	0.919926891
H	3.231969859	0.286634073	1.317804466
H	3.011759171	-1.447614373	1.598810026
C	3.297625671	-0.926642673	-0.475594705
H	2.994989189	-1.940740402	-0.761801780
H	4.396603097	-0.919569044	-0.425369328
C	2.814387513	0.070393466	-1.539604775
H	3.320285257	-0.184224544	-2.486358650
H	1.740230667	-0.090646757	-1.713468874
C	3.066792103	1.500532095	-1.190611424
H	0.985533649	0.540123735	1.050743324
C	4.471222578	1.967896946	-1.027593945
H	4.836457951	1.865695690	0.011806142
H	5.164783154	1.394806777	-1.657033467
H	4.578067837	3.031415060	-1.282626516
C	1.964652271	2.407931373	-0.767972365
H	2.091642967	3.423180980	-1.172912266
H	0.982868407	2.039385214	-1.094396137
H	1.915974557	2.542537841	0.331676886

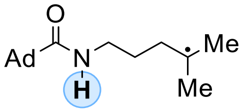

Conformer 6

C	-4.435214204	-0.945630893	1.659899614
C	-3.236045617	-1.727583881	1.118071442
C	-3.396456715	-1.926646155	-0.392513617
C	-3.474699082	-0.563082780	-1.087013953
C	-4.673648893	0.217300085	-0.540854685
C	-4.507423541	0.418290263	0.968256560
H	-4.338531070	-0.813082092	2.747978197
H	-5.362678239	-1.511796627	1.485550402
H	-3.175220300	-2.706406606	1.615357433
H	-4.307014816	-2.508164164	-0.600406240
H	-2.548986489	-2.506457209	-0.790144239
H	-3.587254651	-0.706739834	-2.171298981
H	-5.605316747	-0.329143227	-0.751908152
H	-4.750586045	1.192116018	-1.045068496
H	-5.362151140	0.986666736	1.361496844
C	-3.218828928	1.195872766	1.239539001
H	-3.089003281	1.370671003	2.316968384
H	-3.254004657	2.188941674	0.772203350
C	-1.945834940	-0.947380723	1.389548961
H	-1.798023412	-0.811150567	2.471875538
H	-1.090317831	-1.538077539	1.025927097
C	-2.186749285	0.220065207	-0.815867310
H	-2.219285664	1.200561187	-1.315465784
H	-1.326245433	-0.321941146	-1.237789933
C	-2.002565393	0.430039991	0.700812085
C	-0.741927293	1.255523293	0.969289294
O	-0.782343769	2.383071944	1.435999241
N	0.437525958	0.653954483	0.627258818
C	1.725041676	1.288772773	0.811461867
H	1.518075284	2.326466075	1.098479973
H	2.266083201	0.826192134	1.653500400
C	2.578700886	1.231847897	-0.444022051
H	2.030766633	1.714248412	-1.265278005
H	2.734850834	0.183245798	-0.746674437
C	3.934113893	1.903273877	-0.233633273
H	3.749659103	2.972568935	-0.000640062
H	4.403056463	1.491654507	0.675011238
C	4.883936782	1.781061586	-1.379655582
H	0.422252038	-0.289547069	0.267588752
C	4.386440244	2.052860128	-2.758364313
H	5.172640219	1.907754769	-3.507691741
H	3.541274025	1.406922501	-3.035700328
H	4.023031010	3.090838025	-2.868045779
C	6.345946049	1.827787588	-1.114471169
H	6.610816239	1.283153204	-0.198420811
H	6.920256505	1.401943775	-1.946959099
H	6.718513833	2.860015060	-0.978005169

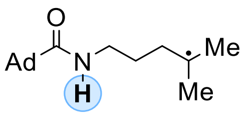

Conformer 7

C	-4.441162481	-0.935144128	1.660023128
C	-3.244463209	-1.724027651	1.122776936
C	-3.403294481	-1.927317475	-0.387426777
C	-3.474359758	-0.565676207	-1.086471637
C	-4.670850048	0.221595505	-0.545005137
C	-4.506423342	0.426746802	0.963738277
H	-4.345705023	-0.799339465	2.747816439
H	-5.370719201	-1.497959901	1.486017393
H	-3.188693675	-2.701456971	1.623362074
H	-4.316116562	-2.505423002	-0.594905992
H	-2.557794990	-2.512185657	-0.781843682
H	-3.585585435	-0.712311794	-2.170490697
H	-5.604474380	-0.321492886	-0.756045730
H	-4.742656401	1.195142453	-1.052435021
H	-5.359434026	1.000018071	1.353590872
C	-3.215020537	1.199671818	1.234842672
H	-3.086220383	1.377329831	2.311910709
H	-3.245134356	2.191420074	0.764335401
C	-1.951312069	-0.948551148	1.393874222
H	-1.804679167	-0.809595868	2.476013419
H	-1.097603049	-1.543815563	1.033459082
C	-2.183488300	0.212752238	-0.815569990
H	-2.210812297	1.191670613	-1.318560142
H	-1.324689020	-0.334510180	-1.234219098
C	-2.001048646	0.426986146	0.700716830
C	-0.737558603	1.247962972	0.969598112
O	-0.774344770	2.375166411	1.437464692
N	0.440162493	0.643000257	0.627369148
C	1.729134109	1.274651553	0.812385677
H	1.523802735	2.310946109	1.105555803
H	2.271152169	0.806703357	1.650788700
C	2.580772517	1.224787644	-0.444823872
H	2.029411068	1.707715205	-1.263450540
H	2.740946786	0.177919552	-0.751294636
C	3.933068756	1.902216529	-0.234041222
H	3.742853269	2.969785144	0.002332756
H	4.405003908	1.490394817	0.672971935
C	4.882672204	1.788757133	-1.381237595
H	0.422237940	-0.299977010	0.266511685
C	6.344567237	1.842547805	-1.116432591
H	6.613472040	1.292173459	-0.204961923
H	6.921302934	1.427253000	-1.952574697
H	6.710857104	2.875910098	-0.971574408
C	4.382593592	2.063215301	-2.758465869
H	5.168137710	1.923100864	-3.509464207
H	3.539115973	1.415482686	-3.036734755
H	4.015580457	3.100331287	-2.864627189

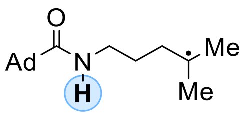

Conformer 8

C	-3.429449557	0.339426365	-1.750140218
C	-3.584315333	0.540273448	-0.239735763
C	-3.941166436	-0.795772772	0.417780925
C	-2.828354774	-1.812159762	0.145795077
C	-2.677908158	-2.013289114	-1.364590837
C	-2.318232726	-0.678919802	-2.023414216
H	-3.189862074	1.297430263	-2.234783983
H	-4.377922748	-0.012644947	-2.183385608
H	-4.380067811	1.273207422	-0.044218895
H	-4.896605801	-1.170900090	0.021329017
H	-4.074130475	-0.660637859	1.501966161
H	-3.075454693	-2.770426562	0.624782570
H	-3.616075810	-2.403691028	-1.786683521
H	-1.897064049	-2.760935001	-1.571532238
H	-2.204569077	-0.820260805	-3.107635645
C	-1.002095894	-0.159518903	-1.443002546
H	-0.712540596	0.792483460	-1.908070406
H	-0.186717902	-0.876058201	-1.641271568
C	-2.267690518	1.058836066	0.340683728
H	-1.990715340	2.023410736	-0.104474301
H	-2.367096735	1.221846277	1.425850466
C	-1.506402606	-1.291063519	0.723176926
H	-0.714520472	-2.035709632	0.532820600
H	-1.605644057	-1.175098241	1.815189972
C	-1.132937640	0.052161149	0.078450208
C	0.176973369	0.673777833	0.562912849
O	0.558147383	1.747075045	0.114377121
N	0.889764480	-0.024031179	1.492469597
C	2.212900070	0.383124777	1.925330087
H	2.390620706	1.358395117	1.456866911
H	2.221750360	0.528522297	3.014284708
C	3.282763016	-0.619604721	1.511946140
H	3.107133250	-1.583188591	2.015238651
H	4.254833611	-0.261896425	1.880201022
C	3.337291672	-0.840080062	-0.003806210
H	4.141386966	-1.573813440	-0.207775123
H	2.402690283	-1.325748101	-0.324539359
C	3.547879163	0.410570155	-0.793508992
H	0.539494140	-0.915539174	1.810027783
C	2.729562728	0.723544437	-1.994763272
H	3.351451979	0.915009741	-2.884157493
H	2.037963112	-0.091944892	-2.242407204
H	2.113458696	1.624109304	-1.837058240
C	4.716446340	1.274252719	-0.465517160
H	4.679690337	1.680279848	0.558120568
H	5.669838764	0.719929240	-0.530865056
H	4.789741445	2.130711880	-1.145626739

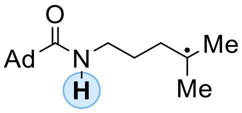

Conformer 9

C	-4.139480809	-0.444849979	1.486289085
C	-3.279858000	-1.559775531	0.882176141
C	-3.830606056	-1.937758109	-0.495764038
C	-3.798186381	-0.711943569	-1.413247260
C	-4.659215301	0.400979119	-0.809035366
C	-4.109534165	0.781483873	0.568650357
H	-3.762717913	-0.177613921	2.484563550
H	-5.174886445	-0.794842515	1.614713131
H	-3.297590941	-2.439184653	1.541634040
H	-4.862003273	-2.309736378	-0.403427361
H	-3.233155814	-2.753019060	-0.931632290
H	-4.184273312	-0.981810216	-2.406651983
H	-5.702390641	0.062145720	-0.721554910
H	-4.661419537	1.279889746	-1.471578330
H	-4.723119663	1.583631723	1.003001239
C	-2.668242712	1.272653869	0.423714888
H	-2.248866151	1.563311335	1.395866387
H	-2.631912311	2.164095785	-0.222891227
C	-1.838673396	-1.068106137	0.737883139
H	-1.413802007	-0.797472250	1.713539429
H	-1.203050387	-1.864733483	0.318553913
C	-2.352701233	-0.219622201	-1.555630447
H	-2.327583246	0.648684989	-2.234529149
H	-1.743162235	-1.019716974	-2.008524099
C	-1.784173122	0.166306215	-0.182539689
C	-0.344553137	0.674501692	-0.189444276
O	0.202824610	1.014798645	0.852426233
N	0.295574624	0.741644081	-1.389438129
C	1.679240700	1.172004989	-1.464096956
H	1.871169588	1.563394429	-2.472816845
H	1.802370008	2.006441112	-0.761173655
C	2.671087169	0.070894579	-1.120151084
H	2.593263842	-0.746266294	-1.851843313
H	2.391287243	-0.348621509	-0.143890242
C	4.105805953	0.605989296	-1.065480461
H	4.776786813	-0.241430244	-0.836683584
H	4.398543725	0.966038017	-2.064847652
C	4.285533844	1.699064793	-0.059660623
H	-0.159880787	0.398464197	-2.220373076
C	3.890749015	1.435300943	1.351782031
H	2.795969188	1.468869423	1.502374127
H	4.206065622	0.430658832	1.673769886
H	4.335070350	2.165126136	2.039268668
C	4.675746930	3.060226189	-0.516318338
H	4.956656330	3.706831551	0.323279557
H	5.525197195	3.028664086	-1.216120866
H	3.862600246	3.576964827	-1.059403446

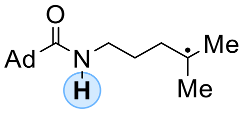

Conformer 10

C	-3.933297584	0.582903624	-1.205571230
C	-3.651381006	0.746822331	0.291055080
C	-3.879371133	-0.590299972	1.001263259
C	-2.939774424	-1.647635692	0.415461630
C	-3.223907598	-1.810793365	-1.079913719
C	-2.994937609	-0.475631161	-1.793219794
H	-3.789346383	1.542780235	-1.722947405
H	-4.981314578	0.285730157	-1.361882044
H	-4.322029973	1.508824813	0.713206285
H	-4.925124255	-0.910768949	0.881426171
H	-3.699194661	-0.480686226	2.081581327
H	-3.093972061	-2.607209167	0.929637662
H	-4.260260277	-2.148734337	-1.229839473
H	-2.569095310	-2.585564426	-1.507205011
H	-3.194919137	-0.591690466	-2.867983124
C	-1.544098893	-0.034773050	-1.595168015
H	-1.346160530	0.917582571	-2.104197995
H	-0.856577937	-0.780199047	-2.026364519
C	-2.200210415	1.187452354	0.488672888
H	-2.007699314	2.150307316	-0.002623947
H	-1.984536434	1.323187430	1.560812236
C	-1.485865289	-1.203197210	0.612878116
H	-0.812977481	-1.978183554	0.208566109
H	-1.278972114	-1.106693568	1.691528526
C	-1.237236932	0.135996909	-0.095348584
C	0.179815711	0.692612629	0.029732895
O	0.496693148	1.731383045	-0.534160439
N	1.060803318	0.001165550	0.806295947
C	2.436199729	0.436188205	0.950053454
H	2.434504930	1.533729315	0.984441371
H	2.818717703	0.074894322	1.915395628
C	3.340018120	-0.038189995	-0.177683464
H	2.941540520	0.335872198	-1.129770180
H	3.312481013	-1.138329076	-0.226152159
C	4.784559811	0.440349873	0.021911791
H	5.380307826	0.115579905	-0.847491930
H	4.795275870	1.540822197	0.008119322
C	5.394233466	-0.063861511	1.290959213
H	0.788662854	-0.885550168	1.200923014
C	5.860332001	-1.474215022	1.371567792
H	5.384749191	-2.107029874	0.610836543
H	6.951085847	-1.560688249	1.218395243
H	5.656116322	-1.917126879	2.357233203
C	5.696419213	0.858974920	2.420702700
H	5.534464437	0.374581740	3.395247714
H	6.747219569	1.199176444	2.421828070
H	5.073400136	1.761882614	2.385361859

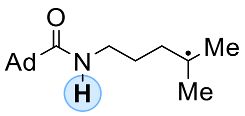

Conformer 11

C	-4.443564143	-0.389682829	-0.715434778
C	-3.827204954	-0.119588905	0.660680759
C	-3.745500586	-1.429748920	1.448826047
C	-2.875653850	-2.432763513	0.686264993
C	-3.495115721	-2.703526951	-0.687843294
C	-3.575433948	-1.394836613	-1.479137696
H	-4.521292492	0.548864527	-1.283636688
H	-5.464638481	-0.783849810	-0.601177501
H	-4.447350071	0.603882955	1.208970289
H	-4.752517442	-1.846616260	1.599716917
H	-3.322175057	-1.244266299	2.447777236
H	-2.807533029	-3.371769639	1.254102897
H	-4.499335785	-3.136682301	-0.567095963
H	-2.890680861	-3.441538729	-1.236940027
H	-4.015001258	-1.587059833	-2.468267026
C	-2.168821940	-0.819860315	-1.656227042
H	-2.193360079	0.116351411	-2.229519395
H	-1.534996983	-1.525872656	-2.216717145
C	-2.421188361	0.454763087	0.483023539
H	-2.446569558	1.401462141	-0.071931383
H	-1.969731277	0.669617727	1.465121398
C	-1.466581634	-1.854933245	0.506222652
H	-0.840374183	-2.589297397	-0.027358398
H	-1.014069863	-1.683189203	1.497328385
C	-1.528534448	-0.539412678	-0.282450685
C	-0.182934541	0.137166433	-0.534495178
O	-0.122542643	1.217049078	-1.107337311
N	0.937148080	-0.518843494	-0.119708342
C	2.253846184	0.061344849	-0.298165853
H	2.990279332	-0.752260614	-0.361838697
H	2.254585426	0.587586808	-1.261724845
C	2.641983580	1.028387133	0.810033283
H	2.606335497	0.504602823	1.778312072
H	1.895768331	1.832363998	0.853074932
C	4.041304826	1.609239126	0.579874625
H	4.036883371	2.169645091	-0.367750137
H	4.246675496	2.349363983	1.372303946
C	5.112865189	0.564763806	0.548360551
H	0.848813125	-1.375365779	0.404455136
C	5.580869757	-0.027335123	1.830758108
H	4.777802100	-0.083465967	2.578021239
H	5.985811338	-1.038825750	1.692263464
H	6.389656662	0.569247546	2.291836269
C	5.908877814	0.348849803	-0.690694378
H	6.746892574	1.063613238	-0.783404551
H	6.354802980	-0.654683313	-0.721183583
H	5.296888637	0.475285575	-1.594612518

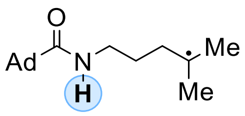

Conformer 12

C	-4.611595214	0.035925440	0.141596474
C	-4.014757381	-1.007034673	-0.807988793
C	-4.147279420	-0.517327272	-2.252509598
C	-3.392214088	0.804555882	-2.413080816
C	-3.983593611	1.849293787	-1.461041062
C	-3.857415367	1.359303665	-0.014887399
H	-4.542150988	-0.316992414	1.181374327
H	-5.679695895	0.183790701	-0.078045179
H	-4.548718286	-1.960681201	-0.691156892
H	-5.207594247	-0.378370298	-2.512216417
H	-3.741805907	-1.269992355	-2.945194368
H	-3.479287363	1.158848621	-3.450282899
H	-5.041109157	2.026726988	-1.707269443
H	-3.461274390	2.811017044	-1.582464015
H	-4.278610817	2.110696736	0.668525239
C	-2.378636094	1.148233961	0.322090391
H	-2.264815765	0.805973688	1.362279602
H	-1.839438043	2.105296677	0.242374736
C	-2.536930379	-1.214273163	-0.473611422
H	-2.414786690	-1.586506519	0.552225530
H	-2.092183041	-1.976241268	-1.129321793
C	-1.912097625	0.596187466	-2.078000007
H	-1.373504078	1.544846428	-2.229566455
H	-1.460399896	-0.135629052	-2.765050109
C	-1.760877165	0.100709857	-0.626859081
C	-0.296719012	-0.138713280	-0.255151791
O	0.098023981	-1.194859869	0.221221688
N	0.553244341	0.904176978	-0.479613513
C	1.959290638	0.812797251	-0.132011918
H	2.318986459	-0.179906093	-0.434161356
H	2.512733817	1.549735734	-0.729438328
C	2.234107350	1.016847441	1.350844078
H	1.587346022	0.335174312	1.919162447
H	1.951962434	2.037317296	1.646168789
C	3.702370326	0.756606156	1.686365937
H	4.334089618	1.488983109	1.157522974
H	3.852123267	0.964041596	2.764247940
C	4.161630483	-0.628844910	1.357370818
H	0.185605323	1.781451906	-0.814781641
C	3.340626695	-1.782333728	1.827341717
H	2.331638893	-1.794113695	1.387740797
H	3.819937298	-2.737598790	1.584270390
H	3.198554652	-1.758809987	2.922827747
C	5.577470341	-0.857545693	0.960404140
H	6.236133024	-1.028766767	1.832127631
H	5.680874950	-1.747493712	0.324233547
H	5.994666408	-0.000491885	0.416439882

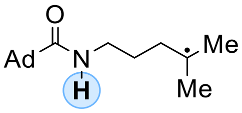

Conformer 13

C	-3.097306563	-1.204523150	-0.752256773
C	-3.279095478	0.251478009	-0.315520385
C	-3.800067703	0.294894664	1.124098987
C	-2.795813094	-0.393399997	2.053717860
C	-2.619025748	-1.850577500	1.616727927
C	-2.095604693	-1.893616975	0.178310870
H	-2.736413272	-1.244341094	-1.792104748
H	-4.061149453	-1.734704280	-0.725562718
H	-3.996048457	0.750473770	-0.983030076
H	-4.777719232	-0.206383194	1.188075691
H	-3.950960290	1.338386319	1.437477435
H	-3.165177414	-0.360131451	3.088603980
H	-3.578747999	-2.384780021	1.682699797
H	-1.917270435	-2.366455186	2.289716023
H	-1.957037676	-2.938625012	-0.134204734
C	-0.746921258	-1.167575627	0.108003547
H	-0.348928655	-1.225492918	-0.919203866
H	-0.034970452	-1.682276054	0.774807212
C	-1.934293434	0.975375302	-0.391313961
H	-1.553667360	0.966917485	-1.425537525
H	-2.032274843	2.028974987	-0.098440498
C	-1.450458627	0.331957420	1.976566592
H	-0.720056752	-0.142492606	2.651796126
H	-1.549374048	1.379691773	2.292100382
C	-0.906918221	0.300394547	0.534014366
C	0.374494729	1.133946328	0.509187924
O	0.339288795	2.342423859	0.702128541
N	1.544735599	0.462512693	0.310698921
C	2.824180579	1.140610950	0.230782078
H	2.679957045	2.124049906	0.693483223
H	3.553445689	0.591407644	0.842077337
C	3.347783055	1.301144002	-1.192966169
H	3.405805377	0.314743101	-1.679555867
H	4.384566126	1.661442739	-1.133987251
C	2.534805888	2.275303267	-2.047117322
H	2.463980923	3.231670111	-1.505631291
H	3.119538826	2.499959278	-2.962193013
C	1.153646106	1.850387858	-2.437370886
H	1.512802299	-0.518804186	0.079806620
C	0.146170895	2.939927176	-2.590463035
H	0.476924967	3.703447012	-3.317220128
H	-0.824212253	2.562859051	-2.936937394
H	-0.012319347	3.458293090	-1.632538173
C	0.945000983	0.563845978	-3.163641257
H	1.633537982	-0.226603571	-2.836242562
H	-0.081004777	0.186172481	-3.030478815
H	1.088589680	0.673616027	-4.254297254

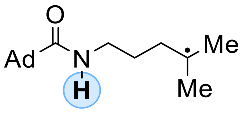

Conformer 14

C	-2.946896078	-1.712938370	-0.720104867
C	-2.743655614	-0.832790678	-1.957197295
C	-3.363547089	0.545336271	-1.711517088
C	-2.684440863	1.200453762	-0.506171167
C	-2.884226874	0.321003153	0.732968363
C	-2.269351433	-1.061006359	0.488899780
H	-2.523552842	-2.713081441	-0.896205931
H	-4.021033383	-1.847523924	-0.522278205
H	-3.223943084	-1.303958401	-2.826577545
H	-4.444587029	0.450775146	-1.528708253
H	-3.241993920	1.178310623	-2.603396822
H	-3.121163113	2.193650654	-0.327772738
H	-3.957210785	0.222306977	0.955443650
H	-2.416616687	0.793373626	1.611572736
H	-2.409120298	-1.691405837	1.379115894
C	-0.771713628	-0.905357013	0.212606514
H	-0.301151350	-1.888472108	0.050698227
H	-0.270404131	-0.452439039	1.082254661
C	-1.247413921	-0.677543378	-2.231581746
H	-0.779945409	-1.650001727	-2.432997955
H	-1.078771673	-0.064794912	-3.129227906
C	-1.184429464	1.354865046	-0.778518259
H	-0.720664903	1.853473242	0.087832392
H	-1.014142831	2.007598265	-1.648121482
C	-0.547272852	-0.024559913	-1.032596761
C	0.955019985	0.047116077	-1.309587548
O	1.492668064	-0.604136730	-2.193011981
N	1.665662866	0.868951392	-0.481583940
C	3.112715312	0.924245387	-0.527356651
H	3.395900994	0.672179832	-1.554401784
H	3.431974883	1.959735221	-0.335274688
C	3.795601506	-0.023051488	0.450570752
H	4.870051009	-0.044941614	0.220786431
H	3.417941321	-1.040779748	0.269975860
C	3.606106844	0.371598242	1.921681906
H	4.310826473	-0.224426630	2.529306630
H	3.917741555	1.421523891	2.044019882
C	2.217432371	0.183750429	2.451901499
H	1.200375951	1.271123956	0.319161815
C	1.479997122	1.334535469	3.046396989
H	0.391846526	1.171369866	3.041107975
H	1.758145688	1.519118479	4.099959167
H	1.687079517	2.274009142	2.511723655
C	1.780676109	-1.208253230	2.753426881
H	1.998290311	-1.893421971	1.920616914
H	2.306112590	-1.617686656	3.634814322
H	0.705561738	-1.271945819	2.963344865

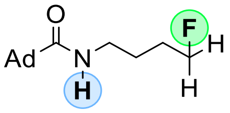

Conformer 1

C	-2.523796793	1.236929177	-1.909700702
C	-2.676747266	-0.126945377	-1.231675621
C	-2.874658204	-1.208088821	-2.298430737
C	-1.663239034	-1.235886239	-3.235345546
C	-1.511063002	0.130237272	-3.910177205
C	-1.310784440	1.210279118	-2.843426126
H	-2.401186555	2.025265942	-1.151425607
H	-3.431693815	1.480787814	-2.481401379
H	-3.544021353	-0.109621354	-0.556484684
H	-3.791341986	-1.006869006	-2.873078537
H	-3.003244357	-2.190123502	-1.819905107
H	-1.804801894	-2.012600565	-4.000167577
H	-2.404453010	0.358912777	-4.510336559
H	-0.654783983	0.117711326	-4.601621100
H	-1.192335117	2.190953341	-3.325864541
C	-0.048970216	0.896673140	-2.030917897
H	0.097391547	1.685929474	-1.273577455
H	0.826824727	0.904759359	-2.700868835
C	-1.416586073	-0.439358011	-0.424436162
H	-1.263686705	0.321684567	0.359057314
H	-1.501688997	-1.409388875	0.082506843
C	-0.401966908	-1.547246797	-2.427609592
H	0.477103895	-1.583349507	-3.090795582
H	-0.476735549	-2.527217994	-1.937640610
C	-0.183179235	-0.472212041	-1.347756192
C	1.006465742	-0.893977412	-0.491583379
O	0.998789328	-1.953023292	0.121387102
N	2.069886335	-0.042161992	-0.430044494
C	3.158565353	-0.289359830	0.497386681
H	3.439457228	-1.344808191	0.414450174
H	4.017065006	0.314847073	0.176504855
C	2.784534200	0.035205160	1.938569604
H	3.651320146	-0.176098239	2.582478904
H	1.986114551	-0.653759333	2.248192033
C	2.334551232	1.475885376	2.123836350
H	3.115528313	2.172995626	1.783354247
H	1.441975708	1.675667407	1.511638126
C	2.000507620	1.782535984	3.561889047
H	2.879141521	1.638648123	4.209347133
H	1.204155966	1.115660495	3.925981429
F	1.575639688	3.085260019	3.680353798
H	2.013694847	0.856147636	-0.883904984

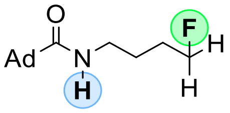

Conformer 2

C	-3.751187819	-1.114284230	0.532272468
C	-2.431155430	-1.750141021	0.085561263
C	-2.485804508	-2.033268674	-1.418440821
C	-2.704399048	-0.721625055	-2.177995784
C	-4.024247850	-0.087186361	-1.731396590
C	-3.972737343	0.196825250	-0.227814393
H	-3.729648148	-0.923003366	1.615245094
H	-4.585728122	-1.806066179	0.342673317
H	-2.268328424	-2.689163693	0.633321847
H	-3.301718349	-2.736528774	-1.642593710
H	-1.550620741	-2.511042363	-1.748099517
H	-2.734285815	-0.919398608	-3.259026376
H	-4.861199755	-0.762979501	-1.962402829
H	-4.202708070	0.846201980	-2.286706403
H	-4.918124187	0.655810706	0.094647524
C	-2.820948275	1.157833718	0.068258026
H	-2.763929907	1.391217608	1.139333531
H	-2.974686474	2.111850441	-0.460737436
C	-1.278833493	-0.788640115	0.382655972
H	-1.208928685	-0.575285917	1.457787442
H	-0.318986672	-1.238405365	0.079088060
C	-1.546903924	0.238452481	-1.877885784
H	-1.684030870	1.172249862	-2.447430978
H	-0.605517525	-0.226689248	-2.215883936
C	-1.482099819	0.539748517	-0.373234507
C	-0.360789403	1.479427727	0.061634287
O	-0.281947445	1.865331607	1.219370892
N	0.551480507	1.843154769	-0.888047440
C	1.719823890	2.635632347	-0.559537175
H	1.853954557	3.414141274	-1.321442903
H	1.495998064	3.132244317	0.391322813
C	2.985267091	1.796121892	-0.432427593
H	3.829294300	2.469816748	-0.219114136
H	3.212551012	1.324465623	-1.402875575
C	2.885752776	0.726081912	0.643835304
H	2.637075256	1.179349059	1.612885049
H	2.066563493	0.030464124	0.411225987
C	4.166057927	-0.061720771	0.763426857
H	5.003637959	0.589264589	1.059215731
H	4.430618102	-0.524700658	-0.200848797
H	0.464550440	1.476627495	-1.823677302
F	4.028104274	-1.054584112	1.703381148

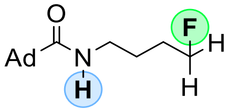

Conformer 3

C	-3.442330827	1.041564356	-2.212730548
C	-2.463614031	1.930030117	-1.440691689
C	-2.882249875	1.994182929	0.030873516
C	-2.869159227	0.584245802	0.628267305
C	-3.848433679	-0.304590756	-0.144580044
C	-3.431471506	-0.368536425	-1.616943393
H	-3.162349912	1.005606476	-3.276604842
H	-4.455430780	1.467656434	-2.162093620
H	-2.462005766	2.941822556	-1.870910019

H	-3.887739713	2.432700381	0.115611625
H	-2.197579139	2.649563515	0.590431486
H	-3.164388851	0.626696615	1.686129196
H	-4.869862534	0.095828000	-0.058713452
H	-3.860601876	-1.316287805	0.287137526
H	-4.131608135	-1.009382106	-2.171350773
C	-2.023107803	-0.955652890	-1.722914230
H	-1.715209641	-1.021613017	-2.778660453
H	-1.986340815	-1.973523569	-1.313016881
C	-1.052182185	1.340148737	-1.544218960
H	-0.744415831	1.316779708	-2.602812981
H	-0.347783594	1.994640440	-1.003940450
C	-1.460376057	-0.003184210	0.522717045
H	-1.420242287	-1.013341074	0.950874036
H	-0.743037559	0.615253289	1.087097862
C	-1.020742667	-0.077250995	-0.952620364
C	0.338558403	-0.772347636	-0.988399021
O	0.469973161	-1.912999680	-0.567316888
N	1.388227042	-0.063465354	-1.498410814
C	2.736395468	-0.595891942	-1.511799602
H	2.637458628	-1.679002223	-1.377398948
H	3.185767045	-0.419536749	-2.497403986
C	3.613048652	-0.006230799	-0.414746835
H	3.714588518	1.080714534	-0.569383031
H	4.626875310	-0.422203031	-0.517164977
C	3.074049163	-0.274801303	0.982032201
H	2.078820232	0.177038830	1.096705154
H	2.940363346	-1.353620242	1.138445115
C	3.982908097	0.285433747	2.046666473
H	4.135517908	1.366844626	1.900105959
H	4.972798725	-0.196058795	2.014163039
H	1.239481593	0.883355739	-1.812507380
F	3.436763433	0.083988457	3.291587441

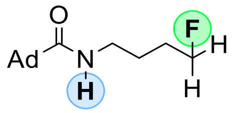

Conformer 4

C	-3.621755795	-1.977514970	-0.525324243
C	-3.192224792	-0.888386033	-1.511791988
C	-4.024943056	0.374437786	-1.270344499
C	-3.808749321	0.866953976	0.163773908
C	-4.237234158	-0.224658705	1.148374267
C	-3.402416565	-1.485572315	0.907963254
H	-3.044204546	-2.897650616	-0.702347564
H	-4.681825036	-2.230237394	-0.676702640
H	-3.344449156	-1.240800950	-2.541906648
H	-5.091090962	0.160721094	-1.439499634
H	-3.735540704	1.158763506	-1.985184222
H	-4.402780334	1.775582260	0.336851367
H	-5.305921963	-0.453370465	1.021312296
H	-4.104588249	0.125614780	2.183373506
H	-3.700625736	-2.269346020	1.619000538
C	-1.917692554	-1.162511734	1.114496157
H	-1.323029806	-2.078442241	0.960184866
H	-1.759033296	-0.829006282	2.153738420
C	-1.711477785	-0.568308205	-1.303392412
H	-1.096954210	-1.464454646	-1.487236772
H	-1.371158777	0.205036029	-2.004739439
C	-2.327767425	1.186868532	0.371672394
H	-2.157171612	1.556716974	1.395518711
H	-1.992598735	1.975455163	-0.314807056
C	-1.469458375	-0.069966796	0.133898809
C	-0.003934676	0.342028353	0.236996881
O	0.460593510	1.203837928	-0.497014866
N	0.764165806	-0.287436537	1.170663586
C	2.165630954	0.048594333	1.326804685
H	2.266972875	1.132647051	1.189603981
H	2.467850375	-0.186009223	2.356150598
C	3.062558513	-0.671697236	0.331364174
H	2.731627415	-0.401702776	-0.681588106
H	2.921696180	-1.759283929	0.431660606
C	4.526634395	-0.312393562	0.525181445
H	4.667221296	0.773798463	0.431577030
H	4.859353531	-0.585587319	1.537510235
C	5.413084594	-1.006462726	-0.478233327
H	5.141598046	-0.716693767	-1.504696700
H	5.308880537	-2.100043238	-0.400814971
H	0.359427284	-0.998291746	1.759320013
F	6.728570696	-0.673319469	-0.257687155

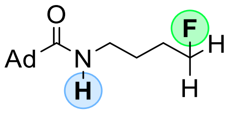

Conformer 5

C	-3.301709282	0.782429610	-0.012420921
C	-3.096840279	0.316906715	1.433071111
C	-3.852557213	-0.995487217	1.657541551
C	-3.319033796	-2.060269510	0.695595880
C	-3.526764275	-1.595814867	-0.749112683
C	-2.772428742	-0.282889646	-0.977960354
H	-2.781895377	1.738236411	-0.180987748
H	-4.370497021	0.962669968	-0.200752115
H	-3.470672933	1.084773156	2.125411854
H	-4.929825429	-0.840506484	1.495879801
H	-3.728061303	-1.330416838	2.698253954
H	-3.853313267	-3.006831625	0.859045863
H	-4.598788325	-1.453558311	-0.952339845
H	-3.165764790	-2.365190204	-1.447776055
H	-2.915916257	0.053256719	-2.014717777
C	-1.278089579	-0.501050631	-0.723734531
H	-0.723279702	0.431631964	-0.914239713
H	-0.874928390	-1.257722364	-1.414939161
C	-1.600812313	0.101828915	1.686803651
H	-1.079780519	1.063046004	1.550238966
H	-1.426749551	-0.208212461	2.728585647
C	-1.827175518	-2.275443135	0.951091514
H	-1.422225395	-3.051483749	0.288619058

H	-1.655131691	-2.629798368	1.977732565
C	-1.050510069	-0.970717623	0.728572607
C	0.439556410	-1.241530260	0.935216599
O	0.900394008	-2.369170817	1.039212654
N	1.249046767	-0.142734313	0.956796514
C	2.690432429	-0.268205772	1.082488968
H	3.088200863	0.733627135	1.289020590
H	2.915269334	-0.890347976	1.957984585
C	3.361088360	-0.880044323	-0.147914586
H	3.103237862	-1.943755625	-0.182816289
H	4.450485651	-0.819881030	-0.005043500
C	2.972189815	-0.228210977	-1.467237194
H	3.502175077	-0.717909838	-2.293874455
H	1.898595579	-0.370764484	-1.658183787
C	3.287997230	1.246261300	-1.520822622
H	4.349271415	1.428001036	-1.285638299
H	2.685714062	1.816258191	-0.795498395
H	0.840208382	0.777112847	0.894543526
F	3.021942486	1.738062808	-2.776753179

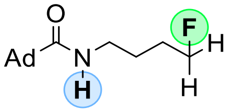

Conformer 6

C	-4.482667239	-0.859546660	1.642300363
C	-3.295333203	-1.694331457	1.154491414
C	-3.419404041	-1.920287202	-0.355750122
C	-3.430877001	-0.570219072	-1.080106187
C	-4.618537069	0.261854959	-0.589356567
C	-4.489865219	0.490521736	0.918937356
H	-4.413443155	-0.704266720	2.729261056
H	-5.423537115	-1.397858015	1.452741813
H	-3.283561288	-2.663421763	1.673713191
H	-4.343335945	-2.474357959	-0.578694454
H	-2.581653877	-2.537008252	-0.716737202
H	-3.512851081	-0.732963627	-2.164355574
H	-5.561187569	-0.257918797	-0.817556820
H	-4.647225751	1.227291889	-1.116555047
H	-5.336362198	1.094833229	1.274512473
C	-3.185344214	1.231964107	1.212601334
H	-3.077870542	1.428987605	2.287176825
H	-3.176944778	2.214863549	0.719687460
C	-1.990300905	-0.950308458	1.451879354
H	-1.877026471	-0.786246731	2.534491278
H	-1.130577930	-1.559187085	1.130294138
C	-2.123203161	0.171155254	-0.784115453
H	-2.097655558	1.135076379	-1.315310874
H	-1.283109411	-0.426277168	-1.172558765
C	-1.980384707	0.412577135	0.729909209
C	-0.698621578	1.165970554	1.089324711
O	-0.693905781	2.144794524	1.819882650
N	0.456810832	0.662383739	0.559042367
C	1.749490418	1.258970056	0.820028385
H	1.565883398	2.296971439	1.121500835
H	2.244862221	0.770011127	1.675065951
C	2.647211075	1.196816963	-0.402405040
H	2.152230413	1.713483129	-1.238661078
H	2.771480172	0.147278765	-0.718639254
C	4.012899768	1.810138794	-0.138072404
H	3.905380829	2.860859291	0.165495572
H	4.509453354	1.292726567	0.695038671
C	4.904946635	1.741526800	-1.352989507
H	4.456229661	2.284702295	-2.198922950
H	5.054978029	0.697394271	-1.669121647
H	0.419104356	-0.153774434	-0.034150626
F	6.129429033	2.296799229	-1.071400214

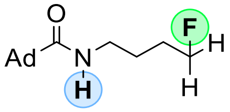

Conformer 7

C	-2.531222388	-2.343813858	-0.056712096
C	-2.681795202	-1.536137589	-1.348943556
C	-3.888263762	-0.600582877	-1.230605306
C	-3.678546922	0.357293198	-0.054195781
C	-3.523863037	-0.449868123	1.238334879
C	-2.318481606	-1.388121711	1.120252684
H	-1.681156233	-3.037890622	-0.140593615
H	-3.430117188	-2.955945958	0.109644368
H	-2.824288937	-2.218993762	-2.198698717
H	-4.805449929	-1.190019759	-1.082699063
H	-4.019164380	-0.031699208	-2.163732867
H	-4.541767031	1.032358543	0.031798610
H	-4.436855711	-1.033207842	1.430760007
H	-3.389767890	0.230035835	2.092485504
H	-2.205425643	-1.963970287	2.049612882
C	-1.053567528	-0.561303715	0.884949849
H	-0.172846068	-1.220424131	0.810606316
H	-0.870407859	0.126413618	1.720848062
C	-1.412972861	-0.706944559	-1.582432945
H	-0.551304646	-1.388562227	-1.681080984
H	-1.511658428	-0.145907802	-2.526215294
C	-2.413297954	1.184852441	-0.289737696
H	-2.241835508	1.889409355	0.534530772
H	-2.513888231	1.782074640	-1.210186359
C	-1.186612915	0.260437843	-0.411532161
C	0.054591475	1.141019589	-0.517417634
O	0.326030656	1.939732729	0.371000412
N	0.837131735	0.997722951	-1.625008216
C	2.090086991	1.711082289	-1.781101758
H	2.131358308	2.150111646	-2.786652059
H	2.060800024	2.540441587	-1.066140683
C	3.310409265	0.821969892	-1.557868803
H	4.212347234	1.431947929	-1.717385840
H	3.332681388	0.044499051	-2.335553738
C	3.373749195	0.159078025	-0.188944171
H	2.484908466	-0.468584180	-0.027796434
H	4.241706372	-0.511850757	-0.146652496

C	3.495379035	1.145213089	0.951524408
H	2.567674570	1.717480895	1.086685324
H	4.320472596	1.851613907	0.761454965
H	0.594990244	0.298734019	-2.310891270
F	3.765888286	0.457730336	2.114867359

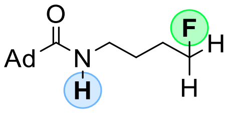

Conformer 8

C	-3.444933117	0.511932206	-1.702700164
C	-3.606084684	0.607166153	-0.182349154
C	-3.895374583	-0.785020487	0.386316006
C	-2.735179034	-1.726522243	0.050348943
C	-2.576724569	-1.821287420	-1.469334638
C	-2.286010738	-0.431228543	-2.040533774
H	-3.253519119	1.509645422	-2.124377138
H	-4.376373393	0.142868711	-2.157910296
H	-4.436045148	1.285810934	0.060550456
H	-4.831710816	-1.179996382	-0.035292205
H	-4.033241509	-0.727890612	1.476860729
H	-2.935213850	-2.724552631	0.465632226
H	-3.494266359	-2.231750146	-1.916679869
H	-1.759035533	-2.513063454	-1.722545045
H	-2.167461821	-0.495941873	-3.131268309
C	-0.996448383	0.113688167	-1.426200715
H	-0.758538892	1.108304101	-1.824198686
H	-0.143502273	-0.540433496	-1.673612429
C	-2.314805142	1.151207273	0.432710321
H	-2.086691112	2.154408422	0.048301869
H	-2.419734836	1.239724018	1.526069639
C	-1.441324445	-1.179833045	0.665184610
H	-0.609116652	-1.867814265	0.439180269
H	-1.555951353	-1.136652130	1.761596602
C	-1.135273935	0.216621688	0.105654565
C	0.144743319	0.861505495	0.626944436
O	0.523567026	1.939990294	0.186308039
N	0.847089511	0.179754514	1.576866133
C	2.154230154	0.612353149	2.028744301
H	2.302357530	1.611642919	1.605474964
H	2.156711893	0.720405637	3.121602648
C	3.261116383	-0.350016152	1.600197306
H	3.157571052	-1.292113424	2.158158716
H	4.226159311	0.077281858	1.910941310
C	3.285146789	-0.661435355	0.109093843
H	4.096442925	-1.369281357	-0.105138456
H	2.350848559	-1.158476762	-0.191642136
C	3.490168053	0.556849744	-0.762629822
H	4.385553967	1.114191223	-0.441572198
H	2.628976618	1.239300749	-0.723608740
H	0.496992699	-0.708663497	1.903464600
F	3.669346515	0.148313467	-2.066562451

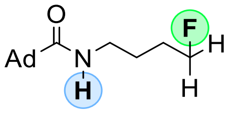

Conformer 9

C	-4.156080086	-0.524000180	1.562607947
C	-3.236249829	-1.617211971	1.011152243
C	-3.747552316	-2.071502495	-0.358718208
C	-3.760360264	-0.879401442	-1.319478115
C	-4.681355961	0.212140861	-0.767255444
C	-4.171523732	0.669134531	0.602361344
H	-3.807389091	-0.204205929	2.555634761
H	-5.175275424	-0.918861532	1.690636797
H	-3.222174252	-2.471685084	1.702723798
H	-4.761033677	-2.489702736	-0.266581644
H	-3.105081404	-2.871893033	-0.756577072
H	-4.119028508	-1.202593174	-2.307223813
H	-5.708430396	-0.173064695	-0.680988460
H	-4.716442104	1.064708933	-1.462413738
H	-4.829928050	1.455041126	0.998798870
C	-2.753003620	1.224139351	0.459042856
H	-2.363414009	1.568014164	1.426570820
H	-2.748901888	2.092653740	-0.218773985
C	-1.818567250	-1.062916722	0.867628231
H	-1.418832387	-0.738916338	1.837502631
H	-1.141348273	-1.843012596	0.484257288
C	-2.339243616	-0.321473661	-1.461102642
H	-2.352580204	0.523690243	-2.169516668
H	-1.681956082	-1.102331736	-1.878893422
C	-1.809371799	0.139942906	-0.095025006
C	-0.395830247	0.715474872	-0.104095287
O	0.141136933	1.081853632	0.932606570
N	0.236651808	0.805705528	-1.308232922
C	1.586839933	1.320970234	-1.404261736
H	1.710222247	1.821348420	-2.375103997
H	1.690976508	2.081589307	-0.621757800
C	2.635585573	0.228412035	-1.229223011
H	2.423301835	-0.584782578	-1.937004610
H	2.516505225	-0.196805856	-0.221909320
C	4.061172822	0.720779767	-1.433806141
H	4.763984538	-0.118793023	-1.359738174
H	4.181381407	1.140036616	-2.443818785
C	4.478277579	1.761041030	-0.419986579
H	4.319269290	1.389770074	0.604330162
H	3.903875414	2.692635616	-0.529266130
H	-0.219393828	0.468389231	-2.141401388
F	5.811583981	2.055514053	-0.587522285

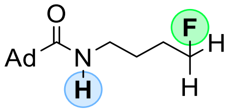

Conformer 10

C	-3.905158409	0.526450663	-1.374410872

C	-3.694256103	0.677482725	0.135499974
C	-3.928102777	-0.672707697	0.819667729
C	-2.942066341	-1.704388858	0.265062304
C	-3.153916322	-1.854896006	-1.243715706
C	-2.920028639	-0.506587102	-1.930503801
H	-3.757116567	1.494897392	-1.874455779
H	-4.938941143	0.210898627	-1.581267581
H	-4.397700700	1.421361543	0.535468045
H	-4.960569122	-1.012102366	0.647978387
H	-3.799487752	-0.573715117	1.908279070
H	-3.100999477	-2.672940128	0.760569734
H	-4.174952820	-2.212006492	-1.444891615
H	-2.464286371	-2.611049640	-1.649174173
H	-3.067853033	-0.613388333	-3.014669140
C	-1.488551700	-0.040369484	-1.661313446
H	-1.286418678	0.921778947	-2.149756844
H	-0.766966593	-0.767172867	-2.068573183
C	-2.262008285	1.143576214	0.404474283
H	-2.067049096	2.115963184	-0.066761744
H	-2.099788734	1.271125329	1.486796212
C	-1.507644627	-1.235030876	0.533788477
H	-0.800884697	-1.990903984	0.152384552
H	-1.352043741	-1.147031711	1.622073861
C	-1.253778382	0.117582823	-0.146791449
C	0.147104050	0.691979163	0.042748974
O	0.462133833	1.762548782	-0.458935848
N	1.029955831	-0.032548056	0.787644651
C	2.386900379	0.434935590	0.991991881
H	2.350721824	1.524949681	1.120018211
H	2.754129283	0.002163788	1.931353961
C	3.304162040	0.094078678	-0.177633190
H	2.828074796	0.472294440	-1.090264989
H	3.367906141	-1.000450360	-0.284150559
C	4.698759477	0.690312461	-0.045006684
H	5.285545170	0.477734110	-0.947599594
H	4.636856102	1.784596107	0.037738204
C	5.463556067	0.148939468	1.140091100
H	5.492371999	-0.952591470	1.111211369
H	5.002463453	0.446082106	2.094078868
H	0.752606526	-0.921752847	1.172258845
F	6.752486715	0.625797055	1.119999984

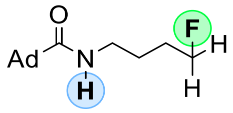

Conformer 11

C	-4.330125386	-1.047194600	-1.189236642
C	-4.152761017	-0.176590202	0.058154970
C	-4.084703903	-1.070026415	1.299454465
C	-2.894788720	-2.024847715	1.173280469
C	-3.068643366	-2.895105636	-0.075838373
C	-3.141653715	-2.003538147	-1.319585652
H	-4.403217424	-0.412130517	-2.084647716
H	-5.267608956	-1.619719955	-1.123346703
H	-5.001180090	0.516471269	0.147711519
H	-5.017106791	-1.644239231	1.407693604
H	-3.979784652	-0.452575397	2.204177357
H	-2.835396390	-2.665508980	2.064798936
H	-3.985628734	-3.496884223	0.010092896
H	-2.229414374	-3.602294742	-0.165239721
H	-3.263612567	-2.628203725	-2.216131967
C	-1.846861335	-1.195267649	-1.443484925
H	-1.876098729	-0.553465874	-2.337378190
H	-0.989640258	-1.875757584	-1.567325236
C	-2.858097039	0.627358765	-0.063552659
H	-2.889088926	1.293766270	-0.935292870
H	-2.719798112	1.276842234	0.812981404
C	-1.596904370	-1.219834605	1.049526563
H	-0.751696894	-1.924017502	0.992164757
H	-1.436852658	-0.605086762	1.948587304
C	-1.651522185	-0.312606903	-0.193702310
C	-0.388053084	0.531554026	-0.366651653
O	-0.423416544	1.729730368	-0.601062488
N	0.796672414	-0.143191694	-0.266054106
C	2.075881763	0.518880721	-0.409213086
H	2.411219193	0.486583444	-1.458542084
H	1.918948013	1.577557496	-0.167375030
C	3.114281924	-0.102652708	0.511594403
H	3.284953828	-1.152412558	0.219033935
H	2.703558985	-0.130233214	1.529676054
C	4.442238736	0.641893402	0.520645339
H	4.295455651	1.676495301	0.860860967
H	5.128050104	0.170682137	1.236216411
C	5.126712141	0.657834526	-0.826701635
H	4.572242595	1.260545089	-1.560542182
H	5.212823457	-0.364852866	-1.228533204
F	6.386882717	1.190340439	-0.695133721
H	0.792077386	-1.136447704	-0.084914709

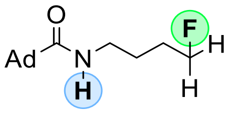

Conformer 12

C	-4.655068750	-0.064288886	0.115994835
C	-4.174397121	-0.898471416	-1.074362189
C	-4.300521134	-0.075292750	-2.359633820
C	-3.439805301	1.185830858	-2.245159323
C	-3.914586388	2.019660633	-1.050813388
C	-3.792867395	1.195366772	0.235023252
H	-4.590413969	-0.655126641	1.041955251
H	-5.711610522	0.214124250	-0.015243393
H	-4.784944451	-1.808589680	-1.157969749
H	-5.351403530	0.201645763	-2.532484694
H	-3.978687469	-0.674551612	-3.224336065
H	-3.524333479	1.779138897	-3.166941047
H	-4.959186301	2.330675238	-1.200494972
H	-3.315633142	2.940046793	-0.969370623
H	-4.128037864	1.796227478	1.092614694

C	-2.327092968	0.800148614	0.443841653
H	-2.211944463	0.224734752	1.374976557
H	-1.726269054	1.716309231	0.561275025
C	-2.711875175	-1.292111185	-0.864292232
H	-2.599732883	-1.902730218	0.043129856
H	-2.348142214	-1.911016047	-1.695158256
C	-1.975653307	0.789350359	-2.037535232
H	-1.348477194	1.692952386	-1.985350817
H	-1.610971571	0.197790029	-2.891283139
C	-1.829574287	-0.041914690	-0.746132169
C	-0.372684954	-0.477567789	-0.581263880
O	-0.006400095	-1.631974701	-0.738268974
N	0.509439401	0.517822914	-0.268156070
C	1.922017667	0.267919361	-0.084397430
H	2.020150885	-0.798503023	0.149984137
H	2.479980616	0.433923613	-1.021934675
C	2.493730585	1.145229570	1.019189547
H	2.006627427	0.887019443	1.971678903
H	2.234728219	2.195774534	0.818062370
C	4.005503565	1.031697895	1.153937346
H	4.496052894	1.335309513	0.218066188
H	4.365195923	1.716300696	1.932494680
C	4.462057308	-0.362979012	1.517176536
H	4.261435492	-1.082088922	0.709627716
H	3.939391099	-0.718572063	2.419585451
H	0.174141674	1.463713614	-0.154813291
F	5.814703815	-0.353879264	1.761452132

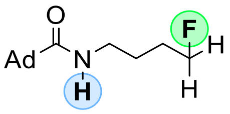

Conformer 13

C	-3.523416222	-0.671808747	-0.602613420
C	-3.597939230	0.456775588	0.429687583
C	-3.600170916	-0.139872781	1.839651324
C	-2.312809824	-0.939491853	2.055624758
C	-2.231808211	-2.066779154	1.020916455
C	-2.235344099	-1.472272305	-0.391206042
H	-3.546412105	-0.254978162	-1.620412938
H	-4.396698599	-1.334272646	-0.505857238
H	-4.516903393	1.039129107	0.273879731
H	-4.475222609	-0.792984275	1.975888131
H	-3.676965988	0.661773068	2.589421053
H	-2.305403516	-1.366476256	3.068787511
H	-3.084422587	-2.751112704	1.142820355
H	-1.319186585	-2.661966050	1.179948740
H	-2.173269070	-2.280509152	-1.133810812
C	-1.024189619	-0.548468031	-0.555713026
H	-1.003052880	-0.120423394	-1.570677921
H	-0.097196466	-1.132548373	-0.437727921
C	-2.387141595	1.376796061	0.266945243
H	-2.373952114	1.834445189	-0.731347420
H	-2.430374335	2.208670381	0.983751882
C	-1.100617883	-0.016910293	1.893719693
H	-0.182213940	-0.594014032	2.086628321
H	-1.128298390	0.790613652	2.641210067
C	-1.083617979	0.594168963	0.478808399
C	0.099789052	1.542825976	0.287311596
O	-0.036295814	2.698415757	-0.087859852
N	1.332012630	1.004352251	0.534023993
C	2.567877726	1.758799983	0.430826185
H	2.276340600	2.792397647	0.208710041
H	3.066197076	1.763090837	1.410296033
C	3.524238383	1.218980968	-0.629364188
H	3.565921537	0.120402988	-0.547001707
H	4.536020627	1.572022061	-0.390369731
C	3.203056540	1.618245684	-2.064414579
H	3.254341558	2.711191825	-2.169292964
H	3.967691133	1.204775378	-2.735711195
C	1.860337923	1.144733550	-2.573501455
H	1.030745109	1.689421458	-2.103138863
H	1.726870982	0.066857144	-2.380687768
H	1.390300809	0.043701128	0.841348371
F	1.803101855	1.358339768	-3.934172974

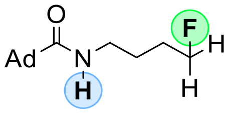

Conformer 14

C	-2.956675726	-1.652250085	-0.362148379
C	-2.624919893	-1.179970113	-1.780626831
C	-3.307894749	0.165180844	-2.043834296
C	-2.802842745	1.199251521	-1.034154028
C	-3.129179964	0.727441441	0.386341824
C	-2.451123018	-0.620862143	0.650224173
H	-2.489496969	-2.629793814	-0.170651873
H	-4.042945117	-1.786997283	-0.249384849
H	-2.978906022	-1.922496671	-2.509584750
H	-4.400191790	0.061051266	-1.960546684
H	-3.093930647	0.503221921	-3.068811498
H	-3.285640727	2.169121097	-1.221587448
H	-4.218075273	0.631329391	0.510161634
H	-2.788472710	1.473276929	1.121137125
H	-2.681253777	-0.957169154	1.671204018
C	-0.934696545	-0.460371893	0.507022586
H	-0.426308596	-1.415925163	0.711415986
H	-0.573014094	0.259493652	1.259421184
C	-1.110596086	-1.019594971	-1.922379041
H	-0.598840668	-1.977934595	-1.758502776
H	-0.844123662	-0.701900632	-2.939845419
C	-1.286729376	1.359512599	-1.178512402
H	-0.932664977	2.128886942	-0.474120655
H	-1.031257634	1.714855602	-2.188574065
C	-0.583058659	0.013159279	-0.917034705
C	0.929669571	0.143132508	-1.088484183
O	1.565301003	-0.506004595	-1.905822509
N	1.550152315	1.043827048	-0.268019389

C	2.983753775	1.271443754	-0.359419791
H	3.237497593	1.360519977	-1.422801675
H	3.199151269	2.238122100	0.115338328
C	3.838427630	0.171932274	0.260684950
H	4.873594927	0.333894574	-0.068005389
H	3.528737143	-0.787413661	-0.176660010
C	3.820248761	0.099052158	1.782769018
H	4.588949433	-0.608759468	2.120704011
H	4.087773867	1.073409924	2.218575775
C	2.510243643	-0.367080739	2.377089864
H	2.169157996	-1.292613349	1.885717971
H	1.711423097	0.379342179	2.262335676
H	1.005545008	1.572876224	0.395405778
F	2.680759578	-0.610174875	3.720986577

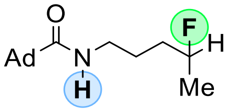

Conformer 1

C	-2.440880793	1.305625699	-1.965315597
C	-2.623805904	-0.005035664	-1.195009941
C	-2.902393351	-1.142502204	-2.182826819
C	-1.726194289	-1.279908050	-3.154986920
C	-1.542774215	0.032933101	-3.922032421
C	-1.262478592	1.168807792	-2.933672154
H	-2.260849147	2.135056001	-1.264309550
H	-3.358071677	1.549595388	-2.521888399
H	-3.465932514	0.091662261	-0.494897531
H	-3.830146656	-0.939309472	-2.738690436
H	-3.052581659	-2.085982721	-1.637644233
H	-1.924982667	-2.097157017	-3.862736850
H	-2.446236611	0.260896579	-4.507242480
H	-0.711522651	-0.059879598	-4.637463550
H	-1.121260233	2.111154520	-3.482033279
C	0.012771922	0.855147492	-2.141573027
H	0.209703737	1.684678226	-1.440211130
H	0.869284799	0.787951649	-2.832192771
C	-1.349410503	-0.319048323	-0.410045705
H	-1.139625710	0.482964289	0.317872742
H	-1.455967515	-1.251155007	0.160555155
C	-0.450768901	-1.591903238	-2.370172882
H	0.402370179	-1.706137618	-3.057812910
H	-0.545611713	-2.534980065	-1.816186390
C	-0.150865016	-0.461616867	-1.369066969
C	1.048896882	-0.878829975	-0.523725719
O	1.040279150	-1.929386020	0.103709176
N	2.114755836	-0.030578638	-0.482927835
C	3.206803339	-0.259210462	0.449318553
H	3.509040967	-1.308933362	0.364832473
H	4.054050536	0.360494992	0.127854021
C	2.825940282	0.054045714	1.889773476
H	3.692334289	-0.120718455	2.539491485
H	2.045252959	-0.654065716	2.201658647
C	2.333475058	1.479550132	2.080309344
H	3.100212684	2.201120961	1.750525471
H	1.442936721	1.658325330	1.455909396
C	1.968351896	1.788133632	3.513945088
H	2.055717645	0.860711097	-0.950323541
C	1.506796190	3.207698558	3.724647361
H	0.600905238	3.413618996	3.143710271
H	1.293719531	3.393970006	4.780698968
H	2.288782816	3.906534995	3.402526087
H	1.200423491	1.071090523	3.854805459
F	3.093203753	1.577564347	4.304256300

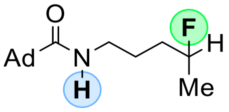

Conformer 2

C	-3.867072036	-1.030546136	0.492957856
C	-2.544170878	-1.711825323	0.128355273
C	-2.529563557	-2.029005447	-1.369605849
C	-2.673830536	-0.730234159	-2.167665479
C	-3.996895352	-0.051139084	-1.803511188
C	-4.014053996	0.268235666	-0.306103844
H	-3.894992793	-0.814932911	1.571173158
H	-4.709753958	-1.704241899	0.276135125
H	-2.435693948	-2.641672660	0.704759448
H	-3.351013452	-2.716618094	-1.620573690
H	-1.591440491	-2.537594975	-1.639816592
H	-2.654216750	-0.951803723	-3.244282633
H	-4.839619518	-0.710075506	-2.060970095
H	-4.121585974	0.872544870	-2.388951815
H	-4.962007322	0.759113722	-0.043698739
C	-2.852421310	1.205810460	0.027640030
H	-2.843427583	1.461189659	1.095484713
H	-2.952517589	2.150956519	-0.529739800
C	-1.382871766	-0.774068524	0.462164137
H	-1.359539177	-0.536951259	1.533742599
H	-0.421731725	-1.256191658	0.216350872
C	-1.509439929	0.208557198	-1.829721120
H	-1.602936450	1.132001957	-2.425138581
H	-0.559810417	-0.278301504	-2.109268207
C	-1.510283743	0.541462380	-0.331019978
C	-0.387814433	1.463725525	0.139055081
O	-0.296119472	1.781930327	1.316685034
N	0.500738679	1.895204011	-0.802169540
C	1.665681856	2.687555288	-0.453835017
H	1.764506950	3.516040371	-1.166930648
H	1.458849245	3.119240447	0.532034306
C	2.942897834	1.860124805	-0.415737739
H	3.796325686	2.521070522	-0.219191004
H	3.123810906	1.414721967	-1.408590955
C	2.903383159	0.760957469	0.634245980
H	2.768912266	1.198959031	1.634705773
H	2.031137059	0.110400205	0.466246096
C	4.146601131	-0.101119444	0.627809118

H	0.407717977	1.573394687	-1.753670295
C	4.161454930	-1.139535188	1.723014303
H	3.316828211	-1.828939508	1.617970815
H	5.090087736	-1.717320668	1.697258216
H	4.086017067	-0.651345900	2.701707385
F	5.256914038	0.719968800	0.791172686
H	4.252509757	-0.581389929	-0.360647575

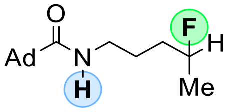

Conformer 3

C	-3.409394885	1.000248880	-2.248070350
C	-2.443932096	1.903982706	-1.476431699
C	-2.884881622	1.988723069	-0.012448530
C	-2.874525788	0.587877068	0.605686438
C	-3.838351963	-0.318142457	-0.167172011
C	-3.401328680	-0.401935486	-1.633004657
H	-3.116010386	0.950273909	-3.307752509
H	-4.424703231	1.423156499	-2.216248359
H	-2.440459077	2.909109076	-1.921660168
H	-3.893443748	2.423991746	0.050711969
H	-2.211871302	2.655110756	0.548249089
H	-3.185128561	0.645649950	1.658633617
H	-4.862768665	0.078253971	-0.100799784
H	-3.851288536	-1.323074852	0.279924661
H	-4.091162940	-1.053249159	-2.188438803
C	-1.988632853	-0.982842886	-1.709843783
H	-1.665400545	-1.062749015	-2.760170203
H	-1.952044801	-1.994136251	-1.284111452
C	-1.027651890	1.319692304	-1.550956415
H	-0.703172460	1.283952575	-2.604172837
H	-0.335357193	1.983919554	-1.006660435
C	-1.461350735	0.007419177	0.528818397
H	-1.420056647	-0.994570273	0.975692470
H	-0.757211965	0.642676543	1.091577493
C	-1.000211938	-0.088191171	-0.939017441
C	0.359442209	-0.782790345	-0.942959687
O	0.486750626	-1.903742739	-0.469022271
N	1.409006525	-0.099145132	-1.483403110
C	2.759991115	-0.628364560	-1.462835087
H	2.662688641	-1.708178952	-1.302031501
H	3.218731325	-0.477483707	-2.448537539
C	3.622761978	-0.003537541	-0.374387517
H	3.734653910	1.075700803	-0.553036639
H	4.634840883	-0.431176728	-0.441500723
C	3.065668239	-0.215777112	1.024568545
H	2.067216046	0.242788506	1.102426836
H	2.926826423	-1.287597606	1.225232969
C	3.938817457	0.388849161	2.100736627
H	4.960307452	-0.023925189	2.033753064
H	1.265472246	0.840000168	-1.822566164
F	4.048372476	1.754806775	1.853840686
C	3.388560326	0.205181791	3.493244862
H	2.376639720	0.622360240	3.553028751
H	3.338941003	-0.857041652	3.755524007
H	4.016101301	0.717564734	4.228910729

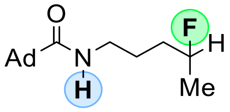

Conformer 4

C	-3.545426061	-2.014278345	-0.652349768
C	-3.187721865	-0.853027547	-1.584181397
C	-4.081500481	0.350044241	-1.266738423
C	-3.869080180	0.777949842	0.188788071
C	-4.226411142	-0.385171594	1.118655082
C	-3.331009886	-1.586671037	0.802352694
H	-2.922445044	-2.891337180	-0.884899942
H	-4.593024344	-2.314968911	-0.803712985
H	-3.336471674	-1.158354633	-2.629738069
H	-5.138018793	0.090473767	-1.432837823
H	-3.843560752	1.184318444	-1.942969483
H	-4.507361614	1.643380418	0.417541611
H	-5.283794177	-0.661561289	0.991115869
H	-4.097842450	-0.083327458	2.169405834
H	-3.578617062	-2.420663413	1.474673232
C	-1.862573935	-1.195514075	1.010536911
H	-1.217774639	-2.066432579	0.807678734
H	-1.716534621	-0.905661754	2.064847025
C	-1.722659949	-0.466446323	-1.375657106
H	-1.065069788	-1.317542867	-1.614682090
H	-1.431314184	0.358743995	-2.038193689
C	-2.403515009	1.164666516	0.395572038
H	-2.236965476	1.490421476	1.435133805
H	-2.121015332	2.003924012	-0.254309159
C	-1.484884426	-0.031823062	0.082757716
C	-0.040557051	0.452592814	0.190927243
O	0.388992098	1.319322153	-0.557602239
N	0.740775742	-0.109885369	1.155951872
C	2.131405451	0.269800509	1.309806649
H	2.213381132	1.331727897	1.046811330
H	2.407877176	0.167667450	2.368632877
C	3.063717896	-0.553438489	0.434407488
H	2.764007382	-0.433288682	-0.613728748
H	2.944901814	-1.621544629	0.677724006
C	4.517379703	-0.144725925	0.603663393
H	4.657133223	0.898184776	0.277738844
H	4.803358627	-0.178099255	1.667363236
C	5.469450670	-1.025129158	-0.176324014
H	5.356581370	-2.070870304	0.159784405
H	0.360907062	-0.826959299	1.753500561
F	5.091951725	-0.993511932	-1.512599977
C	6.912377033	-0.594564132	-0.084600850
H	7.263543442	-0.632073454	0.952747309
H	7.550821898	-1.243623756	-0.690752577
H	7.020736474	0.434056987	-0.448023025

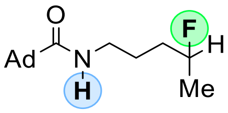

Conformer 5

C	-3.246058795	0.753921750	-0.050831980
C	-3.039087367	0.268969866	1.388041459
C	-3.815864820	-1.032425744	1.603526090
C	-3.307351020	-2.095797204	0.626391041
C	-3.516731490	-1.611900971	-0.811678969
C	-2.741550404	-0.309434274	-1.031742934
H	-2.711268034	1.702738504	-0.212383454
H	-4.312954870	0.954270265	-0.229778216
H	-3.395240537	1.035720877	2.090903284
H	-4.891403653	-0.856862254	1.451245647
H	-3.689772143	-1.380657798	2.639634686
H	-3.857442524	-3.034437253	0.782861856
H	-4.587488463	-1.448577609	-1.005952652
H	-3.174047360	-2.379919263	-1.521023504
H	-2.887486145	0.040073368	-2.063971774
C	-1.249675634	-0.555955223	-0.789709952
H	-0.678491862	0.370011101	-0.970205368
H	-0.863564633	-1.312082440	-1.491511497
C	-1.545031444	0.024855142	1.628750154
H	-1.011015640	0.979660603	1.496991371
H	-1.368560007	-0.297258987	2.666333883
C	-1.817488507	-2.339888668	0.868188046
H	-1.429694132	-3.114173963	0.193990055
H	-1.644555402	-2.709174824	1.889571737
C	-1.018999664	-1.046526650	0.655692022
C	0.469431186	-1.348609309	0.836308086
O	0.917289319	-2.486249718	0.823532024
N	1.287000578	-0.265391484	0.962324600
C	2.732235223	-0.402526479	1.050004623
H	3.136241179	0.580026709	1.321471819
H	2.973154593	-1.092760993	1.868384749
C	3.387389918	-0.904952667	-0.233458690
H	3.152400947	-1.967991152	-0.354289002
H	4.474102937	-0.821946624	-0.106821615
C	2.964817323	-0.154245690	-1.489916096
H	3.543394920	-0.523819171	-2.348472721
H	1.907988369	-0.365057046	-1.714522117
C	3.125836874	1.348953622	-1.396467215
H	2.410496594	1.754900954	-0.659152677
H	0.883773939	0.658642891	0.982882495
F	4.396228964	1.645275712	-0.920290466
C	2.936665692	2.054440078	-2.716258359
H	3.673462941	1.696727945	-3.444361509
H	1.935346104	1.860283144	-3.117432824
H	3.061139158	3.135316411	-2.598651668

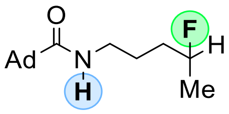

Conformer 6

C	-4.519085004	-0.763819732	1.584811729
C	-3.331476812	-1.657374797	1.216601291
C	-3.377868150	-1.969651540	-0.282731252
C	-3.308540144	-0.665568618	-1.084107593
C	-4.495658885	0.225681239	-0.712676094
C	-4.444182219	0.539533183	0.784582292
H	-4.507516342	-0.545019089	2.662977051
H	-5.463581602	-1.286767985	1.371751151
H	-3.378211917	-2.593426849	1.791402162
H	-4.304386361	-2.510938949	-0.525626401
H	-2.540281943	-2.628660592	-0.559042616
H	-3.334450126	-0.890750142	-2.159957518
H	-5.439728703	-0.280400960	-0.964798294
H	-4.464484273	1.158672020	-1.295390850
H	-5.290987160	1.185981890	1.054670312
C	-3.137891743	1.263575212	1.110547766
H	-3.085421910	1.521431576	2.176243355
H	-3.073221619	2.214743513	0.562569947
C	-2.023679420	-0.930903878	1.545501026
H	-1.968080467	-0.704838889	2.621490388
H	-1.165254280	-1.579810779	1.311193598
C	-1.998584485	0.058027240	-0.756294943
H	-1.914773721	0.987359559	-1.340354800
H	-1.156526472	-0.583499020	-1.060411436
C	-1.932705437	0.386033672	0.746971655
C	-0.650329964	1.126681353	1.130556721
O	-0.654915851	2.125420628	1.834004555
N	0.508623711	0.585046691	0.650288752
C	1.811601364	1.150839311	0.927417994
H	1.631915226	2.138076154	1.369113581
H	2.343376900	0.555217838	1.687903936
C	2.651400713	1.257120505	-0.333237461
H	2.128672897	1.896981162	-1.060042794
H	2.752669929	0.269392846	-0.806799238
C	4.041468906	1.807808603	-0.059848974
H	3.974115517	2.800371365	0.409886389
H	4.572623197	1.157915181	0.653658666
C	4.879113900	1.925166221	-1.313973206
H	0.467474227	-0.255181679	0.091514067
F	4.976996857	0.662733223	-1.885845241
C	6.275281003	2.435796649	-1.060814123
H	6.784248939	1.777072290	-0.347551734
H	6.856469757	2.451962211	-1.987329010
H	6.255150698	3.449005080	-0.644858395
H	4.360725548	2.567423194	-2.045584049

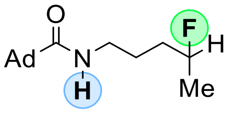

Conformer 7

C	-2.604779718	-2.329395383	-0.152405150
C	-2.749354025	-1.459235107	-1.403876281
C	-3.924591609	-0.494613404	-1.222800890
C	-3.669764577	0.401661910	-0.007724559
C	-3.523106875	-0.468884523	1.244149194
C	-2.348110496	-1.435205932	1.063715869
H	-1.776156746	-3.042500755	-0.280148178
H	-3.518489507	-2.923249010	-0.000960263
H	-2.924796555	-2.097099028	-2.281850213
H	-4.857466743	-1.061899665	-1.086847210
H	-4.052388226	0.120203951	-2.126781138
H	-4.511075264	1.097296526	0.121328196
H	-4.451032564	-1.033169792	1.421477508
H	-3.357289178	0.165469640	2.127287955
H	-2.239114716	-2.057038539	1.963638033
C	-1.062421915	-0.635127011	0.846702379
H	-0.203549316	-1.316957781	0.729284263
H	-0.846362157	0.008804043	1.709053038
C	-1.460905154	-0.656186761	-1.619543724
H	-0.619109873	-1.353484824	-1.768583141
H	-1.563495795	-0.049366818	-2.534596786
C	-2.383994203	1.201869664	-0.224954955
H	-2.180004275	1.862031253	0.628612815
H	-2.479783980	1.844044874	-1.115192624
C	-1.187580066	0.248917641	-0.409476168
C	0.078287434	1.097436569	-0.498611872
O	0.422696619	1.798436518	0.445881031
N	0.792005657	1.035423169	-1.657622866
C	2.060032000	1.723496987	-1.823811878
H	2.098796189	2.155565758	-2.831517167
H	2.056493939	2.557899205	-1.114546165
C	3.262838107	0.812177128	-1.596072562
H	4.181189861	1.398987636	-1.733125752
H	3.273866519	0.029860696	-2.369115858
C	3.286251786	0.166276147	-0.217701543
H	2.341001235	-0.366349495	-0.033110441
H	4.083947490	-0.589257876	-0.172117375
C	3.522607246	1.149425079	0.910002075
H	0.497439510	0.395693659	-2.379844947
F	4.800633610	1.680523170	0.743308359
C	3.433693028	0.510378229	2.272533736
H	2.404493537	0.185165559	2.464553498
H	4.096981331	-0.360922729	2.339321549
H	3.719748314	1.225522798	3.049639355
H	2.807568511	1.982628977	0.849017765

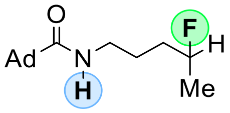

Conformer 8

C	-3.404259097	0.409275955	-1.768710641
C	-3.589106002	0.674802892	-0.271390590
C	-3.993375639	-0.624293243	0.431129237
C	-2.900453598	-1.677570675	0.227435463
C	-2.719331885	-1.943289365	-1.269631274
C	-2.312262381	-0.645631400	-1.973189351
H	-3.129816497	1.340806541	-2.285298651
H	-4.350549228	0.061977189	-2.210095519
H	-4.370257338	1.433939271	-0.123683373
H	-4.948519593	-0.992868545	0.028155187
H	-4.147411726	-0.441292367	1.505456447
H	-3.183103562	-2.608994259	0.738408093
H	-3.656312306	-2.329076436	-1.698252772
H	-1.952087690	-2.716665890	-1.427057588
H	-2.176616585	-0.834133223	-3.047683776
C	-0.997332890	-0.133979385	-1.382142730
H	-0.676165733	0.793175326	-1.874715452
H	-0.194931530	-0.876078000	-1.534466583
C	-2.274169695	1.184691525	0.320377811
H	-1.962803926	2.123336236	-0.156717654
H	-2.394985830	1.393923035	1.395297960
C	-1.580329228	-1.165509033	0.816680790
H	-0.801503342	-1.934787393	0.677794727
H	-1.703853408	-1.001379100	1.900038747
C	-1.159749055	0.139862544	0.126629232
C	0.151347549	0.748951649	0.615821694
O	0.577119308	1.782004719	0.112549590
N	0.816886573	0.090634144	1.606025973
C	2.154042739	0.467662765	2.028631406
H	2.322309023	1.483752192	1.656926708
H	2.190499270	0.512940075	3.124693990
C	3.219308435	-0.491288998	1.506275688
H	3.061706549	-1.485575909	1.950738711
H	4.200377378	-0.146820630	1.856524730
C	3.246077028	-0.618304131	-0.011769444
H	3.990602397	-1.372831959	-0.303344997
H	2.273028936	-0.981380674	-0.380372700
C	3.576745447	0.676176109	-0.728056948
H	0.447755930	-0.788068338	1.938338120
F	4.778990200	1.154271466	-0.209310334
C	3.738373532	0.495742375	-2.216617786
H	4.500548160	-0.260529639	-2.439576380
H	2.789106598	0.174076851	-2.660853861
H	4.036063211	1.437438981	-2.688355556
H	2.805468117	1.433836937	-0.526943461

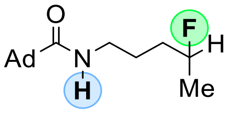

Conformer 9

C	-4.150796482	-0.537743951	1.618261403
C	-3.260099945	-1.627546210	1.013322402
C	-3.807120642	-2.028511217	-0.359699680
C	-3.819513224	-0.806195720	-1.282071220
C	-4.711198057	0.281744787	-0.676886326
C	-4.165381525	0.685233569	0.695767879
H	-3.776185124	-0.254499739	2.612878744
H	-5.173919995	-0.919497228	1.754848002
H	-3.245988499	-2.504349766	1.676356304
H	-4.825623031	-2.432690574	-0.259698436
H	-3.186519198	-2.826003892	-0.796210763
H	-4.203465935	-1.092440011	-2.271774056
H	-5.742775543	-0.089104866	-0.581808973
H	-4.744706537	1.157347216	-1.343045518
H	-4.801648782	1.469174072	1.130811719
C	-2.741256271	1.221010826	0.539815902
H	-2.324805008	1.528284667	1.508091197
H	-2.736688915	2.110505344	-0.110525951
C	-1.836241533	-1.090941906	0.857702810
H	-1.413057898	-0.802845862	1.828992267
H	-1.178402350	-1.868623023	0.437125005
C	-2.391607186	-0.268514007	-1.435906855
H	-2.400358871	0.597860381	-2.117998500
H	-1.759530528	-1.049640900	-1.890853037
C	-1.826550385	0.140558735	-0.067930337
C	-0.403619442	0.695124880	-0.082304978
O	0.138764451	1.053918870	0.953162748
N	0.225830148	0.779432518	-1.289398152
C	1.591931328	1.252832922	-1.394023893
H	1.712441222	1.766457183	-2.359394911
H	1.732063556	1.993624846	-0.598072730
C	2.615013598	0.132629128	-1.251524731
H	2.333017389	-0.697617003	-1.914411430
H	2.565625074	-0.248414599	-0.223684422
C	4.038199095	0.572591974	-1.573341424
H	4.720301877	-0.282708430	-1.466979674
H	4.105282933	0.904028104	-2.621111504
C	4.545393855	1.695497182	-0.690011587
H	-0.230452818	0.426376730	-2.115981873
F	4.384238571	1.314933955	0.632143367
C	6.001482575	2.024047139	-0.918744203
H	6.332699261	2.808165180	-0.231360969
H	6.622920229	1.136901493	-0.753617867
H	6.162909664	2.373410703	-1.944932811
H	3.936494670	2.602564718	-0.840230422

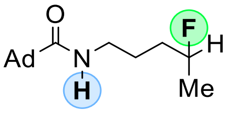

Conformer 10

C	-3.892397820	0.438551137	-1.398442410
C	-3.695666642	0.646209543	0.106593285
C	-3.916862557	-0.681209409	0.837455717
C	-2.912191178	-1.718664408	0.328297768
C	-3.110980705	-1.925925806	-1.175611801
C	-2.889299784	-0.600433124	-1.909250422
H	-3.752438122	1.390637606	-1.931253219
H	-4.920261905	0.102083351	-1.602118192
H	-4.412802541	1.394288323	0.473632165
H	-4.943434631	-1.040471675	0.670404513
H	-3.798372590	-0.540133022	1.922584688
H	-3.061988201	-2.670961349	0.856984774
H	-4.126015845	-2.303042872	-1.371309257
H	-2.408676880	-2.687029245	-1.548766350
H	-3.027526853	-0.748396145	-2.989831301
C	-1.466564306	-0.105096012	-1.646287000
H	-1.273773532	0.841782329	-2.167666598
H	-0.732298495	-0.836090916	-2.021859616
C	-2.272213355	1.140965153	0.369253982
H	-2.086033620	2.098439551	-0.134874582
H	-2.118784475	1.308568309	1.447425987
C	-1.485671400	-1.220262203	0.589830632
H	-0.767187758	-1.980493098	0.240036130
H	-1.339052232	-1.091994601	1.675089470
C	-1.244996362	0.110526369	-0.137405900
C	0.145189670	0.716393971	0.043398969
O	0.440368855	1.777307952	-0.491824813
N	1.032096049	0.038096923	0.821728573
C	2.377551328	0.537314084	1.039474962
H	2.315483272	1.622688925	1.192700300
H	2.767795511	0.098745899	1.961899719
C	3.298842258	0.250332395	-0.141659468
H	2.825066720	0.675006071	-1.035711166
H	3.360982030	-0.837692541	-0.303561362
C	4.700304291	0.828636752	0.014123394
H	5.228961878	0.775442042	-0.947164607
H	4.644001950	1.897200029	0.276782323
C	5.562553953	0.139178593	1.050056277
H	0.769354815	-0.847168769	1.224748075
F	5.009898228	0.348731370	2.312856935
C	6.981224274	0.649977050	1.085499652
H	7.535636829	0.187411570	1.907561774
H	7.500808970	0.427594837	0.147194116
H	6.984990955	1.735972122	1.234540742
H	5.548536734	-0.950433810	0.877540841

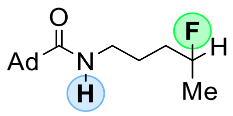

Conformer 11

C	-4.256473173	-1.104875394	-1.279856850
C	-4.152365931	-0.197308803	-0.050597998
C	-4.134228459	-1.055725115	1.217174167
C	-2.926217226	-1.995148476	1.178411471
C	-3.024747630	-2.901777181	-0.052868178
C	-3.049286214	-2.045848456	-1.323176003
H	-4.293600227	-0.496700706	-2.195940970
H	-5.187904062	-1.689794111	-1.243231442
H	-5.013894749	0.484765834	-0.022203666
H	-5.062756394	-1.641249473	1.294724595
H	-4.083640828	-0.412067078	2.108191709
H	-2.903978001	-2.610180897	2.089565976
H	-3.935738523	-3.516035118	0.004269707
H	-2.171463530	-3.597142746	-0.080729958
H	-3.116532723	-2.697187766	-2.206480812
C	-1.761300086	-1.222142711	-1.404463287
H	-1.753892351	-0.607414292	-2.317620863
H	-0.893534284	-1.896878995	-1.469361971
C	-2.863948503	0.623104558	-0.129670772
H	-2.860533511	1.264334074	-1.021114376
H	-2.778702398	1.299289973	0.732757581
C	-1.636187755	-1.172390391	1.098552383
H	-0.773289011	-1.857138787	1.105859335
H	-1.535192792	-0.527906854	1.985256692
C	-1.639880077	-0.301576396	-0.171721750
C	-0.374973499	0.551678147	-0.283079382
O	-0.406374761	1.762921898	-0.438320954
N	0.802781212	-0.136542259	-0.213292076
C	2.093514869	0.509397920	-0.331297272
H	2.464895864	0.429399780	-1.365113504
H	1.933093124	1.576125938	-0.130833244
C	3.096513669	-0.094032877	0.638235711
H	3.260572651	-1.150906445	0.382737112
H	2.666112263	-0.075315422	1.648440740
C	4.443782886	0.617730104	0.646401698
H	4.327225345	1.652855088	1.001331934
H	5.115061492	0.115230899	1.357738282
C	5.126461371	0.656907902	-0.707890215
F	5.120378508	-0.628318672	-1.237957526
H	0.785968684	-1.140505290	-0.104691971
C	6.554873524	1.138866886	-0.643744991
H	7.145577658	0.484427523	0.007515799
H	7.010237757	1.139584857	-1.638532448
H	6.599917474	2.157205417	-0.241032037
H	4.551072590	1.290809036	-1.402901678

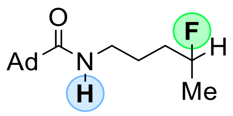

Conformer 12

C	-4.668258067	-0.273910822	-0.029832601
C	-3.984860922	-0.952676281	-1.221125056
C	-4.094654227	-0.050206492	-2.453370591
C	-3.412190952	1.290228105	-2.167191949
C	-4.097612310	1.968684188	-0.977792541
C	-3.988593505	1.068749633	0.256410964
H	-4.609820914	-0.921927428	0.857034704
H	-5.735889191	-0.117823609	-0.246240108
H	-4.469919974	-1.917830161	-1.425364676
H	-5.151667083	0.114685615	-2.710656100
H	-3.624409315	-0.535884352	-3.321927640
H	-3.480606264	1.937614405	-3.053180278
H	-5.154406613	2.162414723	-1.215074392
H	-3.629251367	2.944384657	-0.777292671
H	-4.476600378	1.555177932	1.113060556
C	-2.514021191	0.830405685	0.585174002
H	-2.404399977	0.192945798	1.472267785
H	-2.012082329	1.785250271	0.810303357
C	-2.509681281	-1.189547630	-0.893270681
H	-2.397915650	-1.843736409	-0.018501590
H	-2.006958703	-1.690066540	-1.736450768
C	-1.934144508	1.050124858	-1.835953659
H	-1.441222505	2.019784143	-1.653845718
H	-1.440393871	0.579909912	-2.702573183
C	-1.804504728	0.148244425	-0.599925300
C	-0.376030749	-0.170512214	-0.162874447
O	-0.163204359	-0.867904384	0.820589784
N	0.641929678	0.346114508	-0.905898267
C	2.025906355	0.082277688	-0.554551342
H	2.104389639	-0.966478041	-0.253539754
H	2.646258373	0.207928356	-1.451747431
C	2.512876475	0.991805595	0.568281814
H	1.906521779	0.778658082	1.460557053
H	2.304986119	2.035685500	0.292648221
C	3.992700198	0.843760871	0.898466746
H	4.614318191	1.098412097	0.025188727
H	4.260312003	1.559366800	1.688663559
C	4.405816287	-0.541563754	1.354268633
H	0.438023440	0.938999751	-1.694745414
F	4.367255623	-1.398675994	0.256778428
C	5.804541503	-0.589786635	1.918066015
H	5.879483956	0.003246826	2.835555738
H	6.088448347	-1.621226470	2.147253210
H	6.518846237	-0.190695006	1.187755988
H	3.676220705	-0.927578827	2.086324429

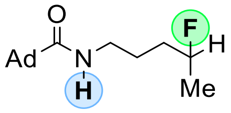

Conformer 13

C	-3.909845533	-0.191537819	-0.279987801
C	-3.522429699	0.391621528	1.082300580
C	-3.035677230	-0.734613142	1.998562306
C	-1.820341533	-1.419302105	1.367798993
C	-2.213319900	-2.004713555	0.009096827
C	-2.695528776	-0.879788549	-0.910272910
H	-4.275641900	0.607990853	-0.941255274
H	-4.732039579	-0.913530692	-0.161606031
H	-4.393754628	0.886466768	1.534634462
H	-3.839218450	-1.469607599	2.157054296
H	-2.771239556	-0.330228820	2.987566681
H	-1.462934401	-2.222872111	2.027692964
H	-3.007649638	-2.755180928	0.138994377
H	-1.353017165	-2.520619542	-0.443306187
H	-2.973085480	-1.295938668	-1.889419168
C	-1.571811253	0.140605650	-1.097696406
H	-1.883890401	0.953827719	-1.766655307
H	-0.690244401	-0.343409138	-1.549162304
C	-2.401791885	1.415609134	0.894897744
H	-2.724866341	2.242078511	0.248133685
H	-2.121362327	1.855376561	1.865566051
C	-0.696003573	-0.394358732	1.175232157
H	0.177010212	-0.895636522	0.726279102
H	-0.394000108	0.004331828	2.157870754
C	-1.168044741	0.746649263	0.262253852
C	-0.132300410	1.833326925	-0.017821922
O	-0.401290538	2.779626025	-0.748964511
N	1.097121005	1.675553586	0.544391533
C	2.225717078	2.530908088	0.234153390
H	1.861246427	3.276478648	-0.483640065
H	2.536204783	3.077305422	1.135984497
C	3.406844686	1.742979194	-0.326349666
H	3.561971422	0.842459559	0.286120478
H	4.317205704	2.344868825	-0.207375997
C	3.287634099	1.337728524	-1.793223173
H	3.302081335	2.236300107	-2.428073093
H	4.170596247	0.743569814	-2.074343760
C	2.042638160	0.553155328	-2.156837963
H	1.288040333	0.826125891	1.055181278
F	1.897133168	-0.508910832	-1.257688226
C	2.095092505	-0.028506937	-3.547551028
H	2.955104003	-0.701687271	-3.645314305
H	1.184489526	-0.597405454	-3.761878796
H	2.188305673	0.765708972	-4.296098854
H	1.152034015	1.190162768	-2.049860982

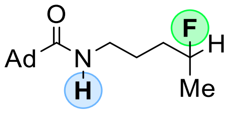

Conformer 14

C	-2.854520696	-1.629620632	-0.098261407
C	-2.538214652	-1.313085040	-1.562702978
C	-3.268078864	-0.032848692	-1.978913492
C	-2.801858567	1.128867278	-1.097646446
C	-3.115890335	0.812625731	0.368254153
C	-2.388737013	-0.469786651	0.786052213
H	-2.350343429	-2.559730881	0.203427571
H	-3.935306328	-1.790770444	0.032557853
H	-2.864974341	-2.147252432	-2.199664626
H	-4.356284875	-0.165071100	-1.881268096
H	-3.064406060	0.191052718	-3.036807294
H	-3.319215264	2.052365994	-1.395640319
H	-4.201314989	0.691584997	0.502290766
H	-2.805805514	1.651144238	1.011242088
H	-2.610589949	-0.694032431	1.839465038
C	-0.879021589	-0.270809662	0.622889978
H	-0.321371172	-1.161377155	0.947226809
H	-0.557890131	0.555392686	1.279292323
C	-1.030544527	-1.116287579	-1.725755158
H	-0.485227137	-2.030058234	-1.451969299
H	-0.773122069	-0.909043085	-2.773358051
C	-1.291693559	1.324869989	-1.264140830
H	-0.962524592	2.182442860	-0.656028837
H	-1.048975814	1.567776913	-2.310152573
C	-0.541052308	0.043867832	-0.848621498
C	0.964649731	0.211632897	-1.051150047
O	1.600436556	-0.423716495	-1.878510265
N	1.573822891	1.136916826	-0.251624949
C	3.006443461	1.370330015	-0.334810360
H	3.266192198	1.516331369	-1.391285054
H	3.221050954	2.311371426	0.190271063
C	3.858189640	0.236887399	0.225497983
H	4.898959549	0.429560877	-0.068480719
H	3.562546989	-0.693211188	-0.272673142
C	3.813148320	0.050270973	1.737767201
H	4.575586964	-0.687936598	2.029198699
H	4.081365697	0.988189863	2.250178703
C	2.483085325	-0.415032702	2.295856456
H	1.022285800	1.666729164	0.405344160
F	2.051368995	-1.513835625	1.562347850
C	2.562058538	-0.821237105	3.746986221
H	1.587288158	-1.161586975	4.109037865
H	3.280812139	-1.640177080	3.867693276
H	2.889444610	0.019106071	4.369794273
H	1.719739548	0.367105701	2.164283783

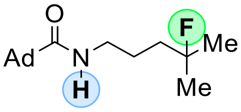

Conformer 1

C	-2.283764122	1.385773229	-1.508949809
C	-2.386003257	0.063739905	-0.743240437
C	-2.844663008	-1.042999499	-1.698773126
C	-1.834202407	-1.190310477	-2.841247759
C	-1.732562501	0.133810657	-3.604046677
C	-1.272341354	1.239167668	-2.649614202
H	-1.972778868	2.193468138	-0.828786746
H	-3.267569124	1.667052461	-1.913648726
H	-3.108887005	0.167122583	0.078439502
H	-3.839396157	-0.803217288	-2.104285718
H	-2.938713267	-1.994339976	-1.154622156
H	-2.162499465	-1.986504685	-3.524572840
H	-2.707736745	0.398713374	-4.039606328
H	-1.023036981	0.035729790	-4.439834401
H	-1.188891329	2.189723907	-3.195946802
C	0.097872045	0.877962017	-2.063730602
H	0.407754402	1.689139057	-1.381953620
H	0.846710852	0.811796728	-2.869789339
C	-1.018330207	-0.305414904	-0.166162852
H	-0.669897624	0.455674961	0.551703127
H	-1.070929995	-1.255519360	0.382554787
C	-0.462263818	-1.550958903	-2.268184077
H	0.270437303	-1.669553558	-3.082234978
H	-0.495256750	-2.504637145	-1.725873570
C	0.015968842	-0.453673335	-1.303774707
C	1.305520284	-0.906300988	-0.630195453
O	1.390233970	-2.007069132	-0.104275409
N	2.332406786	-0.007403044	-0.574873469
C	3.412398455	-0.203608305	0.375578043
H	3.764757060	-1.236084116	0.281434639
H	4.238386038	0.462386732	0.093620339
C	2.952307726	0.063126238	1.804671157
H	3.766401245	-0.190483196	2.497638767
H	2.121199953	-0.622030743	2.019889506
C	2.493839884	1.499067555	2.011893991
H	3.358167357	2.178802430	2.074418898
H	1.899744624	1.824246653	1.141705363
C	1.610239321	1.716420387	3.232192234
F	0.451131148	0.956402523	3.032542845
H	2.200400256	0.909407074	-0.972699762
C	1.169963495	3.161767274	3.337936384
H	0.683372119	3.484442900	2.410294575
H	0.456429270	3.278875397	4.160553651
H	2.027330830	3.818125396	3.526351699
C	2.241247864	1.229037685	4.519493194
H	1.576091502	1.427120068	5.366915542
H	2.427688359	0.151058234	4.479560750
H	3.194055651	1.741972862	4.698353825

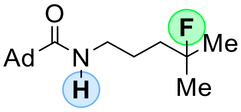

Conformer 2

C	-3.746863360	-1.106990879	0.498722284
C	-2.390448529	-1.731438623	0.156757469
C	-2.344668003	-2.065259357	-1.336968743
C	-2.537903198	-0.783717224	-2.152253816
C	-3.894652898	-0.161379868	-1.810874542
C	-3.943390593	0.174334227	-0.317729340
H	-3.796932885	-0.880038864	1.573881902
H	-4.555451226	-1.821103303	0.281123337
H	-2.246651118	-2.648741983	0.745416888
H	-3.132192901	-2.791571415	-1.587966978
H	-1.382038268	-2.534458764	-1.591026863
H	-2.495943957	-1.017271432	-3.225670978
H	-4.703519704	-0.861114989	-2.069471651
H	-4.054689225	0.748834801	-2.408673358
H	-4.915489876	0.624756488	-0.071350569
C	-2.828906394	1.167353738	0.016811597
H	-2.843842928	1.436372373	1.081268798
H	-2.965129548	2.100100563	-0.553777988
C	-1.276277381	-0.738454754	0.492460327
H	-1.277621105	-0.487723977	1.561302600
H	-0.291310854	-1.179521177	0.263858133
C	-1.419882666	0.209653032	-1.813131249
H	-1.545924688	1.120959610	-2.421094809
H	-0.447126248	-0.239540681	-2.075142243
C	-1.454081310	0.560434168	-0.318704733
C	-0.378427229	1.536440688	0.151631129
O	-0.321626646	1.883753694	1.323341627
N	0.513693210	1.978913300	-0.781456270
C	1.659179739	2.791800962	-0.420376958
H	1.758472076	3.616045268	-1.138376794
H	1.428319215	3.227933536	0.558219593
C	2.951324168	1.984984883	-0.357918002
H	3.777814790	2.677338534	-0.143680493
H	3.162273336	1.541820424	-1.342695730
C	2.897153265	0.874259169	0.679750924
H	2.831203991	1.300008533	1.692067660
H	1.976108028	0.290563549	0.534355802
C	4.057903050	-0.108637855	0.621231080
H	0.459093022	1.622702713	-1.723785112
C	3.875398986	-1.221728377	1.632041319
H	2.906495215	-1.712795616	1.487184524
H	4.664281593	-1.973332682	1.518125266
H	3.915086915	-0.828161504	2.654190770
F	4.026035248	-0.704824230	-0.646735542
C	5.413391995	0.553180616	0.760264012
H	5.474307119	1.114140416	1.700239272
H	6.208758350	-0.199580782	0.752161771
H	5.594243350	1.244520986	-0.069226186

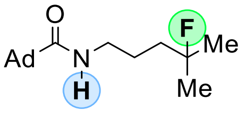

Conformer 3

C	-3.356080931	1.207564805	-2.159902985
C	-2.355537409	2.000331068	-1.314143781
C	-2.794612862	1.975104463	0.152386633
C	-2.843150988	0.527070771	0.646867728
C	-3.841791557	-0.268012193	-0.200165444
C	-3.405481599	-0.242148364	-1.668530034
H	-3.063576753	1.238298931	-3.220532867
H	-4.353459430	1.666729378	-2.088440837
H	-2.309966505	3.039586657	-1.669684021
H	-3.784168190	2.444973719	0.255804544
H	-2.095343997	2.561895853	0.767337962
H	-3.153300370	0.506363056	1.701357027
H	-4.849167581	0.162997090	-0.098013483
H	-3.897003292	-1.306499727	0.158181198
H	-4.119958673	-0.814566064	-2.277089512
C	-2.017009948	-0.871557707	-1.796954618
H	-1.694901040	-0.873595315	-2.850691300
H	-2.023516364	-1.916623656	-1.460001598
C	-0.964226295	1.367530902	-1.441261174
H	-0.642559592	1.409676960	-2.495286118
H	-0.244505069	1.952686326	-0.844427978
C	-1.454856841	-0.101363629	0.518198412
H	-1.454300828	-1.139059915	0.876232785
H	-0.726933617	0.453728743	1.133431497
C	-0.994698441	-0.087863509	-0.952763110
C	0.337411110	-0.831888471	-1.016758067
O	0.425775174	-1.985286805	-0.617987865
N	1.408391144	-0.153392291	-1.521049259
C	2.740851260	-0.726397757	-1.540751560
H	2.609747793	-1.807290259	-1.415512720
H	3.190291354	-0.557199444	-2.527871035
C	3.641712816	-0.164236759	-0.446521525
H	3.763131798	0.920626180	-0.581648913
H	4.640698891	-0.607007469	-0.567282337
C	3.100145493	-0.428144364	0.950352737
H	2.057833416	-0.078510458	1.001626246
H	3.065514781	-1.510167709	1.147609534
C	3.859920846	0.268557352	2.071944934
H	1.296142278	0.808145122	-1.804708689
F	3.783921920	1.646562927	1.818783906
C	5.332666019	-0.087994002	2.112849781
H	5.821032950	0.398966833	2.963879330
H	5.463882100	-1.172331441	2.210194769
H	5.838588950	0.243085746	1.200268268
C	3.191851951	0.034919765	3.411057534
H	3.687363839	0.624966474	4.189529777
H	2.136556997	0.327798970	3.372175104
H	3.244827152	-1.024080127	3.689135127

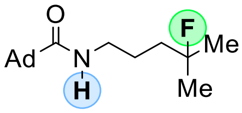

Conformer 4

C	-3.603426738	-2.019709038	-0.586026471
C	-3.186028764	-0.918914085	-1.565225839
C	-4.043371416	0.328045712	-1.326281945
C	-3.846661101	0.819230462	0.111219019
C	-4.262895548	-0.283537536	1.088701435
C	-3.403837411	-1.528703065	0.850730906
H	-3.007612761	-2.928355641	-0.761789107
H	-4.657674213	-2.290954997	-0.746160005
H	-3.323992427	-1.270349361	-2.597611281
H	-5.104431272	0.096475423	-1.503891422
H	-3.762257923	1.119834143	-2.036125231
H	-4.458598034	1.716225136	0.283198226
H	-5.326491029	-0.530271983	0.952595715
H	-4.144471971	0.065627566	2.125877159
H	-3.693946738	-2.319770598	1.557060118
C	-1.926758347	-1.179256059	1.070753773
H	-1.313322515	-2.083227562	0.919720846
H	-1.785330314	-0.845533938	2.112421262
C	-1.712779012	-0.572529282	-1.344027058
H	-1.080422130	-1.456272984	-1.526839889
H	-1.380607784	0.209317710	-2.039355227
C	-2.373427759	1.165205361	0.331638238
H	-2.217085468	1.534821986	1.357948850
H	-2.047280522	1.961876602	-0.349953649
C	-1.490776738	-0.075179743	0.096956252
C	-0.033869132	0.367329600	0.213734973
O	0.424895169	1.218523412	-0.535327650
N	0.725884557	-0.216046527	1.183984429
C	2.124981857	0.128866392	1.344867014
H	2.230574956	1.196917685	1.117260556
H	2.399444925	-0.014332193	2.399370015
C	3.041538769	-0.683069705	0.441426933
H	2.761749413	-0.497247146	-0.603509558
H	2.875930467	-1.753190333	0.636371299
C	4.500452894	-0.315473610	0.653729767
H	4.617540623	0.773790057	0.543691736
H	4.809639767	-0.559018313	1.683156238
C	5.474401754	-0.977873188	-0.311658170
H	0.324916957	-0.926266143	1.775897870
F	5.109330679	-0.575225277	-1.600177576
C	6.890144670	-0.487226127	-0.084534464
H	7.257437468	-0.796593311	0.900883565
H	7.557758073	-0.901610168	-0.847525737
H	6.933631230	0.605695117	-0.144561833
C	5.401503499	-2.491978024	-0.287739505
H	4.413209956	-2.840109042	-0.604078820
H	6.144396922	-2.916230653	-0.971768350
H	5.600820260	-2.873986448	0.720662284

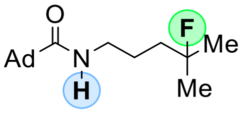

Conformer 5

C	-3.027465028	0.971049840	0.025272313
C	-2.975897115	0.366515051	1.431827987
C	-3.832554939	-0.901897902	1.472316467
C	-3.293794566	-1.914614437	0.457350616
C	-3.352772198	-1.308737508	-0.947961677
C	-2.494900571	-0.041217092	-0.993423586
H	-2.429996230	1.895118830	-0.011654029
H	-4.061756700	1.248781144	-0.227556118
H	-3.357247857	1.096073288	2.160821847
H	-4.880740808	-0.655151542	1.244780270
H	-3.815987352	-1.335806262	2.483302851
H	-3.902103738	-2.829657175	0.489965449
H	-4.392664038	-1.067855942	-1.215972441
H	-2.989640447	-2.036973551	-1.688812035
H	-2.529465263	0.395864600	-2.001888320
C	-1.044651990	-0.390952635	-0.647392689
H	-0.421278801	0.516979287	-0.717488768
H	-0.640946055	-1.112791599	-1.376204708
C	-1.525621053	0.018945097	1.779076459
H	-0.916400552	0.935150632	1.780456418
H	-1.462372554	-0.403439600	2.793546230
C	-1.844272183	-2.260029220	0.802318931
H	-1.440529002	-3.001849370	0.099291418
H	-1.777659593	-2.718464049	1.798732104
C	-0.968676759	-1.001534938	0.766873702
C	0.473774123	-1.380272379	1.102974058
O	0.826784092	-2.530270989	1.325031453
N	1.350626012	-0.337525567	1.140779601
C	2.778280107	-0.553422444	1.253080575
H	3.227502423	0.396155301	1.564675265
H	2.951024506	-1.282814466	2.052201007
C	3.413461188	-1.067708434	-0.039064754
H	3.138606290	-2.124454044	-0.150417891
H	4.507362919	-1.044378814	0.070434748
C	2.983502037	-0.344672262	-1.313772866
H	3.517081786	-0.790257584	-2.165980168
H	1.914790985	-0.532547453	-1.495555762
C	3.200625973	1.161255658	-1.386319514
H	1.073813512	0.565356846	0.782429957
C	2.773267287	1.700670031	-2.735154696
H	3.404596243	1.301346131	-3.536365281
H	1.733877425	1.419422744	-2.943274108
H	2.843266571	2.793353647	-2.747739849
C	4.611110624	1.582230550	-1.035564260
H	4.875339553	1.278326322	-0.017664365
H	5.329701829	1.127813586	-1.727797955
H	4.706994342	2.671189501	-1.101545930
F	2.348243662	1.777771430	-0.442358982

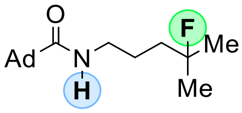

Conformer 6

C	-4.425307508	-0.964037209	1.595026539
C	-3.213310556	-1.732782457	1.061672350
C	-3.341749247	-1.896291836	-0.456242004
C	-3.408819252	-0.516872300	-1.120167522
C	-4.620950700	0.248442650	-0.583049293
C	-4.486348591	0.415684830	0.933052288
H	-4.352370129	-0.855435294	2.687480293
H	-5.347880071	-1.527017845	1.387890285
H	-3.162052925	-2.722846490	1.537223113
H	-4.246750736	-2.473804896	-0.696912388
H	-2.485276198	-2.465315862	-0.850099891
H	-3.496400690	-0.635089600	-2.209769585
H	-5.546310043	-0.295307100	-0.826286258
H	-4.689862723	1.234246158	-1.066938168
H	-5.350893210	0.972645752	1.321185837
C	-3.205993668	1.190498785	1.249245394
H	-3.096454919	1.343446735	2.331262630
H	-3.236658998	2.193674811	0.801191869
C	-1.933099061	-0.955852340	1.381486397
H	-1.815868640	-0.837433550	2.469723249
H	-1.058421258	-1.524127821	1.028440279
C	-2.127117226	0.260112941	-0.802387633
H	-2.146075074	1.248619662	-1.286391546
H	-1.264603165	-0.280952556	-1.223006946
C	-1.977696779	0.437638825	0.720529572
C	-0.717490089	1.228738977	1.076505740
O	-0.742741049	2.236254121	1.766551164
N	0.450252902	0.725467569	0.575969893
C	1.736406621	1.342500459	0.822129135
H	1.534018248	2.367050865	1.156933717
H	2.260630711	0.839230987	1.651380226
C	2.605595350	1.330051582	-0.423381474
H	2.069550960	1.846279914	-1.232301404
H	2.768183399	0.294187504	-0.759053410
C	3.958809593	1.976038124	-0.171629893
H	3.836129236	3.054074797	0.011815071
H	4.400487636	1.552611628	0.744089465
C	4.970343341	1.781463787	-1.291727316
H	0.430274526	-0.122023798	0.026974456
F	5.185571855	0.401521870	-1.410432182
C	4.469649119	2.265118596	-2.637550019
H	5.243011343	2.129383831	-3.400961878
H	3.584527637	1.701670363	-2.949915498
H	4.207126015	3.328981615	-2.594137343
C	6.304598135	2.402920834	-0.935799889
H	6.666372138	2.004341431	0.018240084
H	7.046691780	2.170745807	-1.707271871
H	6.220009449	3.492904549	-0.852279852

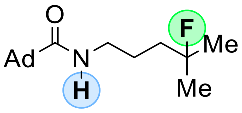

Conformer 7

C	-2.617405391	-2.483910228	-0.322370654
C	-2.883247785	-1.489022663	-1.455863602
C	-4.081542179	-0.608158075	-1.091791761
C	-3.777343094	0.159362333	0.197839408
C	-3.508036142	-0.834779729	1.332107926
C	-2.310681810	-1.718492368	0.968133353
H	-1.772855268	-3.139696740	-0.583919082
H	-3.494691392	-3.132400009	-0.179259182
H	-3.093344991	-2.035506869	-2.386443880
H	-4.978462834	-1.232141503	-0.961673279
H	-4.295807472	0.096455314	-1.909624395
H	-4.634482248	0.795633236	0.460363835
H	-4.397112994	-1.459611986	1.506118165
H	-3.305907053	-0.292857458	2.267717837
H	-2.114022727	-2.430163592	1.782726808
C	-1.076667708	-0.838769678	0.758454871
H	-0.201276170	-1.461285794	0.507019349
H	-0.826510833	-0.284293289	1.673500686
C	-1.643152502	-0.610434679	-1.661859335
H	-0.794259020	-1.260781944	-1.933966322
H	-1.814496398	0.085235894	-2.499145508
C	-2.543181706	1.038389053	-0.012486043
H	-2.304910033	1.612406854	0.891977427
H	-2.727033799	1.768079088	-0.817004840
C	-1.324700625	0.171939174	-0.379418504
C	-0.097409402	1.080136218	-0.447060769
O	0.191483481	1.803587758	0.498270649
N	0.656887911	1.025595055	-1.581044932
C	1.904444084	1.757111479	-1.721867001
H	1.947915917	2.186390271	-2.730974334
H	1.873772184	2.578736430	-1.002177910
C	3.129172553	0.881401689	-1.466247805
H	4.030783390	1.450575296	-1.732364613
H	3.097312765	0.019456542	-2.149361858
C	3.214103366	0.386606381	-0.025584315
H	2.207117247	0.111996761	0.317939859
H	3.812736908	-0.533981047	0.023938000
C	3.832490337	1.392160672	0.954701521
H	0.413437817	0.355830002	-2.295416934
F	3.546686212	2.692681014	0.517859932
C	3.223482971	1.249728235	2.331855688
H	2.149594770	1.459388057	2.270334075
H	3.363738791	0.228380873	2.706905127
H	3.686724196	1.947337327	3.038753867
C	5.344501956	1.289882292	0.976452296
H	5.766921416	2.092170215	1.590888717
H	5.659461774	0.326744565	1.394566219
H	5.757758326	1.378147649	-0.035624854

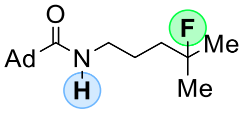

Conformer 8

C	-3.374106039	0.581256189	-1.693359372
C	-3.654126916	0.476927472	-0.190442465
C	-3.812018244	-0.996055664	0.197108584
C	-2.524235400	-1.752930806	-0.139495141
C	-2.248307883	-1.649300991	-1.642334794
C	-2.086202497	-0.177189008	-2.031732810
H	-3.276072455	1.637264964	-1.985020274
H	-4.218009561	0.164906612	-2.264366037
H	-4.576460076	1.023744500	0.052364372
H	-4.660390769	-1.443727781	-0.342172859
H	-4.031998125	-1.082983986	1.272046099
H	-2.629753069	-2.809680457	0.145788285
H	-3.076091188	-2.103285392	-2.207734193
H	-1.337115529	-2.211227945	-1.899332660
H	-1.884127616	-0.100113170	-3.109769301
C	-0.918846265	0.433862915	-1.255161900
H	-0.776979425	1.490255641	-1.519695561
H	0.028199414	-0.077975023	-1.491896893
C	-2.486413115	1.083817122	0.589218745
H	-2.353574362	2.145436232	0.345009936
H	-2.680950182	1.025559559	1.672059834
C	-1.349365573	-1.141358909	0.633229652
H	-0.434340524	-1.704768906	0.381161502
H	-1.520627200	-1.246785658	1.717284054
C	-1.180854181	0.339335586	0.263091142
C	-0.017947818	1.061535846	0.938309119
O	0.111350373	2.271922454	0.835244836
N	0.892180313	0.289828858	1.601214765
C	2.197402551	0.802688908	1.961950446
H	2.319276537	1.739022404	1.407824569
H	2.244748807	1.053898690	3.032098725
C	3.296370842	-0.202837313	1.632342512
H	3.201867766	-1.069006702	2.305961560
H	4.266756727	0.249250210	1.880481468
C	3.306940115	-0.727780348	0.198400723
H	4.109388750	-1.474038303	0.104177036
H	2.368186786	-1.264179143	-0.012714535
C	3.484051657	0.294042627	-0.919204610
H	0.732685795	-0.703709790	1.678028740
F	2.332920480	1.092175384	-0.955263593
C	4.661767235	1.223319868	-0.706591219
H	4.525577313	1.834818077	0.190844406
H	5.590525213	0.649670245	-0.601761572
H	4.767361919	1.900470428	-1.560965691
C	3.559894678	-0.398068713	-2.264440940
H	4.449917667	-1.034328153	-2.328985596
H	2.672884967	-1.023640618	-2.422729285
H	3.603429336	0.346335261	-3.066724366

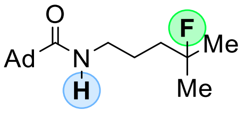

Conformer 9

C	-3.999604508	-0.547940519	1.721459220
C	-3.185653968	-1.640446020	1.020692737
C	-3.838533687	-1.980097392	-0.322247826
C	-3.879165230	-0.727413692	-1.202235139
C	-4.694661875	0.362832388	-0.501152211
C	-4.042547728	0.705316092	0.841408298
H	-3.548567733	-0.308764478	2.695761925
H	-5.021533960	-0.906077308	1.917731943
H	-3.150911172	-2.539140771	1.653012386
H	-4.858966152	-2.359811503	-0.163328076
H	-3.273572489	-2.778895376	-0.826595090
H	-4.339165013	-0.969349623	-2.171042867
H	-5.727202758	0.016324723	-0.344478992
H	-4.750031416	1.260917892	-1.135051633
H	-4.623552269	1.491130582	1.344946413
C	-2.617684704	1.207854759	0.602361055
H	-2.126887050	1.471197616	1.548635367
H	-2.632520967	2.118234759	-0.018120595
C	-1.761054613	-1.137455420	0.781748036
H	-1.263046835	-0.894001123	1.729415072
H	-1.157625939	-1.918508747	0.291404968
C	-2.450269732	-0.222945747	-1.439342149
H	-2.482780359	0.666464519	-2.090289469
H	-1.872568673	-1.003104540	-1.962845180
C	-1.779470349	0.123781446	-0.102370831
C	-0.347041173	0.640834804	-0.204512095
O	0.288176663	0.924510948	0.805168485
N	0.186225341	0.780655635	-1.447710305
C	1.552722673	1.244807147	-1.613077505
H	1.669831751	1.638386276	-2.631023629
H	1.711422186	2.083716199	-0.930062798
C	2.565465722	0.137186356	-1.344924501
H	2.391868998	-0.681974545	-2.057688268
H	2.361639450	-0.276934523	-0.348491000
C	4.019083503	0.581254845	-1.446076565
H	4.670946557	-0.286376033	-1.268127739
H	4.239143635	0.929316362	-2.467457144
C	4.456594767	1.693482969	-0.496939485
H	-0.340844522	0.488652654	-2.255483429
F	3.865224596	2.884662800	-0.949016617
C	5.953770456	1.914452915	-0.583289461
H	6.233915860	2.800109153	-0.002568666
H	6.501309351	1.051805036	-0.187620855
H	6.258963186	2.074853881	-1.623790546
C	4.005312149	1.483788570	0.934138723
H	2.912989151	1.448217650	1.017221266
H	4.411668122	0.543190947	1.326613671
H	4.371222162	2.303496755	1.562309627

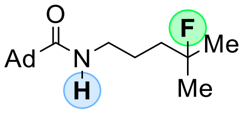

Conformer 10

C	-3.867482756	0.477526698	-1.404131141
C	-3.690696727	0.537753512	0.116144481
C	-3.886740255	-0.861471419	0.706187127
C	-2.853264715	-1.819270557	0.107515142
C	-3.032128867	-1.878710119	-1.411911526
C	-2.835384788	-0.481212877	-2.005473728
H	-3.745964836	1.481240539	-1.837389855
H	-4.885223445	0.140038077	-1.652158774
H	-4.429777949	1.227844289	0.547680347
H	-4.902980665	-1.224859405	0.491638104
H	-3.782403229	-0.828557498	1.801451839
H	-2.985148156	-2.822925570	0.536441882
H	-4.035795620	-2.257458314	-1.656835481
H	-2.307932681	-2.581771018	-1.851064908
H	-2.959812528	-0.521985141	-3.097003150
C	-1.428093682	0.018278540	-1.676653351
H	-1.253502155	1.017190216	-2.097555898
H	-0.671937991	-0.653213543	-2.114682152
C	-2.282673370	1.036911756	0.444503226
H	-2.115241130	2.044248013	0.040848323
H	-2.144004930	1.098594268	1.535889497
C	-1.442602301	-1.316489650	0.435836223
H	-0.701181030	-2.019606677	0.021110672
H	-1.310385977	-1.294936288	1.530398924
C	-1.227113984	0.085792442	-0.150836214
C	0.147258317	0.698700663	0.104020360
O	0.429370037	1.807993436	-0.330631603
N	1.038221527	-0.030334726	0.831510117
C	2.377638786	0.468192901	1.083496908
H	2.300211337	1.548986724	1.262957915
H	2.752120902	-0.010914989	1.995422143
C	3.314097225	0.197855381	-0.086101158
H	2.836450439	0.595875085	-0.990586043
H	3.408485493	-0.887554201	-0.220521830
C	4.704610342	0.811164890	0.031392807
H	5.253150945	0.567279398	-0.888875381
H	4.630420779	1.909021180	0.057884446
C	5.533826421	0.358367488	1.240687800
H	0.792198742	-0.953632837	1.151744644
F	5.119154311	-0.932059989	1.600661462
C	5.336694893	1.244944351	2.456827795
H	4.280140327	1.354564027	2.719435266
H	5.863640289	0.819207217	3.317939746
H	5.742351367	2.244796917	2.263747656
C	7.003337191	0.258257911	0.887347735
H	7.593530807	-0.040742329	1.760590109
H	7.150586144	-0.484079661	0.095153576
H	7.377019260	1.224625418	0.528480077

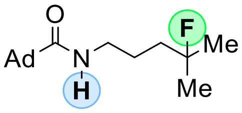

Conformer 11

C	-4.362457695	-1.017232660	-1.152676413
C	-4.157215088	-0.182258838	0.114352942
C	-4.054370070	-1.111866316	1.326491736
C	-2.863638455	-2.056201681	1.143301541
C	-3.063556454	-2.889901559	-0.126759749
C	-3.172719420	-1.962082178	-1.341224367
H	-4.461954388	-0.356947878	-2.027083885
H	-5.294879706	-1.597278477	-1.079624143
H	-5.006649399	0.502953275	0.245844274
H	-4.981399302	-1.693867569	1.440786388
H	-3.929485145	-0.520921027	2.246318341
H	-2.780402137	-2.723031662	2.013705404
H	-3.974619272	-3.500137821	-0.036393638
H	-2.222450764	-3.588432992	-0.257561115
H	-3.314482121	-2.560763877	-2.252550249
C	-1.885270747	-1.143341078	-1.473339107
H	-1.939991845	-0.477138812	-2.347949108
H	-1.032324125	-1.818974655	-1.642902696
C	-2.869125800	0.631679211	-0.015840788
H	-2.926116314	1.323642112	-0.866603591
H	-2.710583672	1.256389394	0.874569737
C	-1.574097859	-1.239512759	1.012742613
H	-0.721761392	-1.930390321	0.917643639
H	-1.399512645	-0.646926098	1.923988174
C	-1.661706959	-0.296867644	-0.203558737
C	-0.399843471	0.557390284	-0.354424927
O	-0.435430789	1.776306442	-0.417556132
N	0.773422707	-0.139833274	-0.421921688
C	2.065654715	0.499954610	-0.572244898
H	2.458890872	0.357177888	-1.587017640
H	1.896265652	1.576316225	-0.453206307
C	3.047643067	-0.010804287	0.474066891
H	3.206940754	-1.093336846	0.340810839
H	2.577161101	0.099434304	1.460974996
C	4.387171263	0.715865028	0.494961780
H	4.215509007	1.787222372	0.678139962
H	4.981112103	0.347167157	1.344227671
C	5.256049296	0.610571024	-0.753807593
F	4.590186613	1.283353652	-1.788312035
H	0.745795379	-1.148698236	-0.386069592
C	5.468239414	-0.815052353	-1.221667140
H	5.926171217	-1.420183142	-0.430189316
H	4.521005297	-1.282123828	-1.510539666
H	6.129903127	-0.828825506	-2.094646016
C	6.574087291	1.332003761	-0.552704572
H	7.187632184	0.825296861	0.201327750
H	7.133080358	1.360382661	-1.493863185
H	6.400450212	2.362719678	-0.224014374

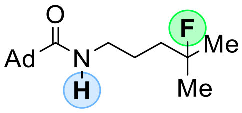

Conformer 12

C	-4.694616098	0.012443596	0.092592946
C	-4.221201340	-0.848709491	-1.081553848
C	-4.312302032	-0.038789185	-2.378262744
C	-3.420682593	1.201285153	-2.269356142
C	-3.888459003	2.062193934	-1.091374941
C	-3.803040034	1.251686219	0.206245697
H	-4.655218703	-0.568062727	1.026571947
H	-5.742216279	0.315775276	-0.054384113
H	-4.853800705	-1.744099291	-1.161153580
H	-5.353876585	0.262434694	-2.566661473
H	-3.996057783	-0.656951903	-3.231665331
H	-3.479396982	1.784441702	-3.199662817
H	-4.923034196	2.397595007	-1.257724862
H	-3.267212055	2.968058030	-1.013863847
H	-4.134105227	1.871802823	1.051846706
C	-2.350645991	0.822510327	0.438503638
H	-2.261948590	0.256531964	1.378593538
H	-1.727315191	1.723503834	0.552308004
C	-2.771510668	-1.276221957	-0.848414155
H	-2.685331626	-1.878328386	0.067320227
H	-2.412978853	-1.914252521	-1.666829608
C	-1.969748852	0.769507750	-2.038188656
H	-1.319855959	1.657360680	-1.991740862
H	-1.610527010	0.157176818	-2.879506195
C	-1.859458376	-0.047231335	-0.734249714
C	-0.416013911	-0.518914663	-0.539230350
O	-0.092944281	-1.693803335	-0.618694225
N	0.494868999	0.467406671	-0.285633104
C	1.905361136	0.201853819	-0.088703156
H	2.003986128	-0.870116091	0.104054795
H	2.479803753	0.398114508	-1.008552944
C	2.452763752	1.041414385	1.057446817
H	1.936175033	0.763248455	1.988015020
H	2.192580681	2.097013020	0.878970013
C	3.963762324	0.960222082	1.234072991
H	4.462681724	1.302328531	0.314273508
H	4.270355959	1.654931469	2.029799202
C	4.539424546	-0.413662013	1.562892071
H	0.181553573	1.426598276	-0.238598377
F	4.365022777	-1.220817294	0.430592454
C	3.835150663	-1.102277641	2.713376279
H	2.784484501	-1.295081090	2.474444437
H	4.314162545	-2.063665078	2.927667049
H	3.879037444	-0.484093381	3.618093397
C	6.033769656	-0.328121909	1.798723082
H	6.252106818	0.247181056	2.705979527
H	6.455356978	-1.332696092	1.917332453
H	6.529452668	0.155734879	0.950576619

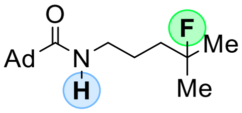

Conformer 13

C	-3.079369406	-1.128706097	-0.645154947
C	-3.183725478	0.384041441	-0.434567229
C	-3.808458206	0.666073564	0.935107450
C	-2.934374822	0.056183868	2.035036153
C	-2.830164120	-1.456260521	1.820648959
C	-2.202961097	-1.736990554	0.452616877
H	-2.648308508	-1.343234480	-1.634727397
H	-4.079906423	-1.586641722	-0.622829943
H	-3.809476416	0.824764974	-1.224006593
H	-4.823232503	0.242421077	0.982748676
H	-3.904321097	1.751133154	1.089129003
H	-3.380333954	0.260689563	3.019074147
H	-3.827887321	-1.916981254	1.878310900
H	-2.220406630	-1.910371565	2.616758709
H	-2.117442718	-2.822567425	0.300068556
C	-0.803549310	-1.113618200	0.396907251
H	-0.339117611	-1.334504602	-0.577498766
H	-0.179843277	-1.569700089	1.183112705
C	-1.786089583	1.001663109	-0.495754197
H	-1.321701976	0.808863706	-1.474502848
H	-1.831106846	2.090794880	-0.361094129
C	-1.537995656	0.677819814	1.976026159
H	-0.898546170	0.260017289	2.770566632
H	-1.584006968	1.763817775	2.135502955
C	-0.888511689	0.406081203	0.604235315
C	0.446849194	1.147307790	0.584784402
O	0.478592971	2.372559727	0.608998408
N	1.578596078	0.387646852	0.599306413
C	2.899252431	0.971535126	0.465279783
H	2.837661332	1.981582122	0.885449351
H	3.600502859	0.397786787	1.085595863
C	3.403292925	1.032121576	-0.975897475
H	3.432318429	0.012439627	-1.388602097
H	4.446620702	1.373186202	-0.949548379
C	2.565330365	1.956542157	-1.870685807
H	1.965243983	2.621091444	-1.235055118
H	3.219224753	2.606790376	-2.468749756
C	1.634250511	1.256030653	-2.857131768
H	1.485339532	-0.587864134	0.358074498
F	0.954123785	0.223253154	-2.178963362
C	0.574299976	2.210806241	-3.362262334
H	1.034664282	3.050945767	-3.895077438
H	-0.111296969	1.698879291	-4.047297093
H	-0.000489440	2.610618899	-2.518067550
C	2.380268001	0.588992688	-3.994639746
H	1.682520023	0.024751655	-4.623078818
H	2.878248439	1.340249404	-4.617859077
H	3.140197535	-0.105404295	-3.619619019

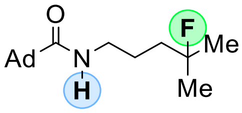

Conformer 14

C	-3.056694830	-1.752733561	-0.617135926
C	-2.777763912	-1.060051115	-1.953901704
C	-3.437172233	0.321878747	-1.958521243
C	-2.860411286	1.168343713	-0.820550905
C	-3.134053481	0.474859586	0.517748644
C	-2.479054182	-0.909985216	0.523076646
H	-2.606093278	-2.756755433	-0.610596208
H	-4.139970629	-1.885207136	-0.474917903
H	-3.185255895	-1.666089895	-2.775647487
H	-4.526905662	0.223974616	-1.839431030
H	-3.262432079	0.819239629	-2.924269627
H	-3.328587381	2.163215191	-0.823770726
H	-4.218473438	0.378156982	0.677178190
H	-2.740512381	1.082464609	1.347092028
H	-2.670697543	-1.405765699	1.485781453
C	-0.967186074	-0.754468254	0.331716445
H	-0.480680345	-1.743352726	0.347335179
H	-0.551094918	-0.181361200	1.176587001
C	-1.268334861	-0.903514809	-2.143632330
H	-0.773990356	-1.885129449	-2.165653156
H	-1.041465302	-0.427224842	-3.106789504
C	-1.349115346	1.324090082	-1.012425794
H	-0.932978590	1.954419283	-0.211952530
H	-1.132506403	1.833623116	-1.964027959
C	-0.668348470	-0.059210717	-1.011638039
C	0.835044255	0.099639148	-1.251900660
O	1.383967904	-0.292450351	-2.272311637
N	1.521905265	0.740544720	-0.262630448
C	2.942400908	0.997594717	-0.370723749
H	3.165689792	1.029906533	-1.442027872
H	3.153957497	1.991861774	0.048587390
C	3.834348904	-0.049170166	0.292728861
H	4.843329317	0.059317165	-0.128849542
H	3.491584766	-1.047027359	-0.016425895
C	3.973580050	0.050531193	1.810876847
H	4.766172376	-0.639578958	2.136480975
H	4.325130226	1.060448014	2.076034616
C	2.760537206	-0.241638823	2.685820677
H	1.076643936	0.961878636	0.616355355
F	1.822338895	0.801725788	2.497952115
C	2.065603799	-1.539731983	2.341188215
H	2.754480231	-2.382702200	2.471296325
H	1.201856039	-1.693987778	2.998220338
H	1.713633494	-1.536744853	1.304925091
C	3.133119131	-0.184130303	4.153161366
H	2.235468236	-0.281967863	4.773047945
H	3.822941661	-0.995224830	4.411348137
H	3.614902341	0.770905452	4.390201175

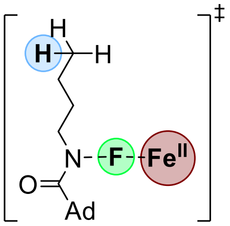

Fe	-1.174990427	0.010671362	-1.754486825
O	0.593484843	-0.803379238	-1.711983653
S	1.694593892	-0.484904022	-0.692763123
O	2.192594642	0.878893680	-0.845716050
O	1.389248018	-0.981143539	0.637418454
C	3.040693357	-1.556290172	-1.335537903
F	3.319771049	-1.233302342	-2.591311693
F	2.683192611	-2.827593307	-1.277255189
F	4.115648409	-1.368114610	-0.588933931
O	-2.325304709	-1.605514243	-0.865941597
S	-3.573642926	-0.786103928	-0.733102443
O	-4.164338691	-0.654646752	0.582157682
O	-3.278301615	0.495730215	-1.468161082
C	-4.836143363	-1.661282237	-1.749760469
F	-5.269168664	-2.710212919	-1.079158752
F	-4.314625182	-2.053586421	-2.895365608
F	-5.839971431	-0.830511349	-1.982156711
O	-0.888224637	0.949694373	0.164012795
C	-1.137083466	2.346582359	0.065918949
H	-2.210314954	2.510892590	-0.117926145
H	-0.851508463	2.843217973	1.003531122
C	-0.274400701	2.848699161	-1.058607196
H	-0.504655126	3.897149108	-1.297685168
H	0.785863599	2.749201712	-0.788611341
O	-0.533881647	2.031079318	-2.200433830
C	-1.228872945	0.382211247	1.434342135
H	-2.283961835	0.574703171	1.664717699
H	-1.045534981	-0.690467184	1.360860513
H	-0.571066275	0.810295020	2.199159475
C	0.445956082	2.223764551	-3.224828160
H	1.443614849	2.000085628	-2.828363700
H	0.195798397	1.537054035	-4.034352480
H	0.395069669	3.259259093	-3.587712139
F	-1.502929975	-0.088179900	-3.631545839
C	1.344502423	-3.208840680	-4.234378112
C	1.547891718	-2.296802384	-5.448259528
C	1.661203221	-3.143017105	-6.716891874
C	0.378898912	-3.959547403	-6.896033484
C	0.186313279	-4.884657460	-5.690778495
C	0.074136594	-4.043153600	-4.417007474
H	1.254306757	-2.603391331	-3.324215148
H	2.219119725	-3.864715926	-4.104852990
H	2.461785113	-1.701351129	-5.309064898
H	2.530605679	-3.814149251	-6.647035522
H	1.822981672	-2.496237360	-7.593140738
H	0.445937518	-4.557302410	-7.816396961
H	1.035530743	-5.579833684	-5.609017413
H	-0.719252150	-5.496028941	-5.824681985
H	-0.064015968	-4.698914217	-3.546028003
C	-1.128219711	-3.100658343	-4.519251701
H	-1.236671166	-2.514269474	-3.595763662
H	-2.060404444	-3.674748406	-4.642117256
C	0.354004536	-1.343179610	-5.562325181
H	0.298200991	-0.723715350	-4.660063256
H	0.477154596	-0.673278783	-6.429568643
C	-0.817818652	-3.013268616	-7.006721153
H	-1.745334042	-3.583464848	-7.160847027
H	-0.711108240	-2.352927348	-7.879303822
C	-0.947204161	-2.161435200	-5.730681888
C	-2.166940686	-1.279684030	-5.889737853
O	-2.803629063	-1.169566092	-6.929347465
N	-2.790344723	-0.746139503	-4.767816526
C	-3.730913168	0.339046558	-4.813839878
H	-4.680083884	-0.157250552	-5.082758456
H	-3.866658555	0.651739794	-3.768099428
C	-3.457386966	1.522537011	-5.725508621
H	-4.296308780	2.223753850	-5.599378328
H	-3.474756726	1.196333430	-6.772804422
C	-2.150565895	2.237802998	-5.422495862
H	-2.110358411	2.483051702	-4.349641665
H	-1.315751787	1.543641732	-5.605064427
C	-1.969469296	3.490359007	-6.261636306
H	-2.769677707	4.215912654	-6.068112452
H	-1.993315961	3.255350642	-7.332816900
H	-1.014441779	3.987472756	-6.054070358

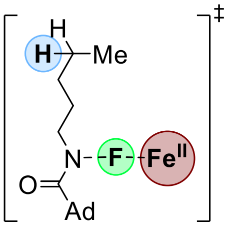

C	10.897220000	-2.090878000	-9.951441000
C	11.843691000	-2.698159700	-8.912233000
C	13.240331000	-2.848458000	-9.523448000
C	13.765422000	-1.474554700	-9.951807000
C	12.816558000	-0.867457800	-10.988826000
C	11.420158000	-0.716430600	-10.378537000
H	9.883226000	-1.996997400	-9.532884000
H	10.821943000	-2.752509600	-10.827304000
H	11.467792500	-3.682857800	-8.599606000
H	13.200526000	-3.524458600	-10.390922000
H	13.925029000	-3.302320200	-8.792020000
H	14.769471000	-1.581343300	-10.386504000

H	12.767475000	-1.509231300	-11.881248000
H	13.193382000	0.112676110	-11.318859000
H	10.737447000	-0.272689000	-11.117110000
C	11.494422000	0.201232660	-9.152492000
H	10.481158000	0.309739740	-8.727365500
H	11.840088000	1.201834900	-9.459849000
C	11.922087000	-1.779343600	-7.691775300
H	10.925625000	-1.674303300	-7.229463600
H	12.589951000	-2.195917800	-6.925780300
C	13.840109000	-0.554792340	-8.732250000
H	14.228779000	0.434178170	-9.023692000
H	14.523969000	-0.955950600	-7.972880400
C	12.445721000	-0.388687850	-8.101117000
C	12.599730000	0.433956060	-6.825046000
O	13.375327000	0.092929110	-5.941621300
N	11.824418000	1.547822700	-6.697387700
C	11.808930000	2.300305000	-5.454756700
H	12.841805000	2.545518400	-5.179205000
H	11.285851000	3.245644800	-5.653373700
C	11.134901000	1.563548400	-4.304451000
H	11.035006500	2.240384600	-3.446120700
H	11.775380000	0.728403870	-3.990299500
C	9.747789000	1.034521500	-4.652880700
H	9.105992000	1.825099500	-5.072288500
H	9.836476000	0.273663820	-5.453896000
C	9.079876000	0.412645730	-3.493295400
H	8.029116000	0.136882300	-3.566141000
H	9.674442000	-0.201614340	-2.817490800
H	11.166139000	1.786457800	-7.422291800
C	5.034860000	-0.162102430	0.021093596
C	4.577560400	1.201024000	0.548185900
C	4.815490700	1.266376900	2.060490100
C	6.305345500	1.071393800	2.356772400
C	6.756784400	-0.291823200	1.828356400
C	6.525690000	-0.347916540	0.316544300
H	4.854960000	-0.227356060	-1.063438400
H	4.451655000	-0.967306260	0.493578200
H	3.507809000	1.342790400	0.336151660
H	4.224315600	0.489352730	2.568939000
H	4.476718000	2.237099200	2.453165500
H	6.476940600	1.126270200	3.441882600
H	6.197009600	-1.097961300	2.326813700
H	7.822386700	-0.450898560	2.053833000
H	6.859290600	-1.322074000	-0.071531800
C	7.322647000	0.759872140	-0.379633430
H	7.152636000	0.706932100	-1.462661900
H	8.400920000	0.622812030	-0.221372700
C	5.376332800	2.310595300	-0.139873500
H	5.199887800	2.288444800	-1.228331100
H	5.059317000	3.299179600	0.217958200
C	7.105993300	2.176228800	1.667872200
H	8.181597000	2.057421200	1.874839300
H	6.808389000	3.164504300	2.043106800
C	6.881298500	2.134846000	0.136261570
C	7.607383700	3.360731100	-0.409229800
O	7.232790000	4.483017400	-0.071877480
N	8.793377000	3.264797700	-1.100023300
C	9.268306000	4.401100000	-1.861067200
H	9.569711000	5.153703000	-1.117294500
H	10.189012000	4.074113400	-2.363558500
C	8.292644000	5.025365400	-2.852566500
H	8.791965500	5.894844000	-3.308435200
H	7.429951700	5.418604000	-2.299412000
C	7.819700700	4.089358300	-3.956130700
H	8.699003000	3.726780400	-4.514169000
H	7.363817700	3.192757800	-3.509011300
C	6.844249200	4.767985300	-4.904030300
H	7.301409000	5.642528500	-5.385943000
H	6.502255000	4.091730000	-5.696982400
H	5.956352000	5.122302500	-4.364899600
F	8.909740000	1.953829400	-2.229780200

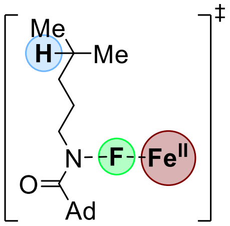

Fe	-1.181651180	0.002722561	-1.712809105
O	0.604013889	-0.781211486	-1.707214169
S	1.714958087	-0.454838409	-0.703150116
O	2.158995104	0.931054143	-0.818664754
O	1.461172542	-1.006627908	0.615884935
C	3.085351309	-1.449376141	-1.413859146
F	3.309495083	-1.084058809	-2.669335052
F	2.786139256	-2.736559666	-1.377823751
F	4.174915899	-1.232498519	-0.696702043
O	-2.290949057	-1.671776126	-0.880703631
S	-3.555908575	-0.886516621	-0.709120901
O	-4.133441210	-0.811303103	0.616611772
O	-3.300966268	0.425087115	-1.404593932
C	-4.812812317	-1.757366935	-1.736697386
F	-5.221182218	-2.830682618	-1.089330962
F	-4.297926646	-2.112089011	-2.897615384
F	-5.833031317	-0.938537996	-1.938369268
O	-0.903056710	0.851638278	0.247222560
C	-1.189149820	2.244183486	0.225180032
H	-2.270125040	2.389036437	0.071911457
H	-0.895965046	2.701232809	1.180525283
C	-0.364359805	2.826228286	-0.890480839
H	-0.635748984	3.875434669	-1.078165132
H	0.703705990	2.751847967	-0.644278464
O	-0.616152086	2.053093355	-2.064849544
C	-1.197692553	0.211978281	1.494639295
H	-2.249086755	0.372775094	1.763232619

H	-0.998320899	-0.851834470	1.359548131
H	-0.526295842	0.611535268	2.263157036
C	0.334752579	2.328110133	-3.097348589
H	1.345331788	2.095821854	-2.740149946
H	0.071455903	1.694835828	-3.946038012
H	0.261597384	3.384805406	-3.388612279
F	-1.502031458	-0.029218441	-3.598018571
C	1.363940829	-3.108829446	-4.289100401
C	1.521373487	-2.185927446	-5.501605050
C	1.614012539	-3.020663193	-6.779804605
C	0.339907607	-3.855913895	-6.929880051
C	0.193561080	-4.792378598	-5.726975582
C	0.102961009	-3.962276464	-4.444173994
H	1.287171956	-2.510872810	-3.372955678
H	2.251669525	-3.751217243	-4.186402296
H	2.428679549	-1.577049790	-5.381965615
H	2.495430677	-3.677919576	-6.739072806
H	1.741268022	-2.364646280	-7.654443069
H	0.390009549	-4.446197104	-7.855950182
H	1.054612503	-5.475181839	-5.672935701
H	-0.705990411	-5.416204565	-5.841519301
H	-0.001280313	-4.626203569	-3.574925124
C	-1.115800938	-3.037995125	-4.505571386
H	-1.206016425	-2.459990210	-3.575082686
H	-2.042705768	-3.624422934	-4.607618707
C	0.310474396	-1.249546051	-5.576021759
H	0.271930933	-0.636174342	-4.668784510
H	0.400241731	-0.571698336	-6.441187971
C	-0.874662347	-2.928895266	-6.999841480
H	-1.796333529	-3.513522063	-7.131175180
H	-0.804412082	-2.261190390	-7.870342436
C	-0.981630554	-2.087411522	-5.715186390
C	-2.226281997	-1.234679395	-5.830639939
O	-2.924488545	-1.166897109	-6.833928087
N	-2.806373203	-0.680407269	-4.696575183
C	-3.749855649	0.403606996	-4.716444401
H	-4.719108429	-0.096423857	-4.887219128
H	-3.802547445	0.759395005	-3.677268644
C	-3.546260150	1.541226778	-5.703082311
H	-4.365722913	2.253361464	-5.531212010
H	-3.664574482	1.161131601	-6.725749411
C	-2.202583561	2.238125625	-5.566896946
H	-2.115716596	2.687975218	-4.561310714
H	-1.404036398	1.480549664	-5.628724151
C	-1.944225015	3.306009587	-6.627358650
H	-2.049391495	2.824134256	-7.613991141
C	-0.524426343	3.841921811	-6.505014305
H	-0.394662509	4.369957670	-5.548269791
H	0.219965548	3.036527045	-6.546830477
H	-0.293201602	4.556044693	-7.302973852
C	-2.947820270	4.449624151	-6.543455745
H	-2.724221545	5.228806001	-7.281586718
H	-3.977242287	4.117216686	-6.717405825
H	-2.911791198	4.917144497	-5.547994034

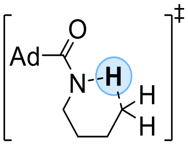

C	-3.779440008	0.507543225	-1.832419535
C	-2.831012398	1.697871544	-1.659219065
C	-2.892328566	2.194560348	-0.212298496
C	-2.473181141	1.064611701	0.732299364
C	-3.425737060	-0.121929107	0.559922898
C	-3.363298357	-0.622683481	-0.885791458
H	-3.755186912	0.154879999	-2.874198355
H	-4.814205968	0.816158610	-1.619737867
H	-3.124689815	2.507958820	-2.342340961
H	-3.911976352	2.529438483	0.030909019
H	-2.228313634	3.062493488	-0.082190684
H	-2.509124676	1.419722979	1.772293207
H	-4.453261279	0.180528274	0.812847404
H	-3.149922583	-0.931822254	1.252576543
H	-4.042630863	-1.478090163	-1.010686352
C	-1.934539781	-1.061641054	-1.212833005
H	-1.862129358	-1.436256116	-2.241971960
H	-1.630221788	-1.887176561	-0.549675764
C	-1.402553889	1.259486597	-1.985433299
H	-1.328960512	0.906244655	-3.023576805
H	-0.704948743	2.105147282	-1.877375602
C	-1.042070083	0.624087043	0.404060508
H	-0.738604576	-0.174752391	1.099390473
H	-0.346116457	1.464792115	0.547381894
C	-0.962380755	0.117717716	-1.041431577
C	0.424276758	-0.306103243	-1.507136507
O	0.569945261	-0.916774596	-2.561109928
N	1.517440610	0.160044867	-0.809627039
C	2.813957568	-0.361053228	-1.204611869
H	2.752417968	-1.428491107	-1.472608875
H	3.125070251	0.166217205	-2.118341937
C	3.830791600	-0.135251631	-0.095523847
H	3.939206277	0.943421854	0.090681478
H	4.810024709	-0.509832009	-0.415248023
C	3.371653312	-0.834515534	1.185615114
H	3.277456667	-1.912542559	0.992136604
H	4.133637070	-0.721973657	1.971750772
C	2.051245974	-0.269912840	1.633092696
H	1.496663414	0.020207552	0.505469546
H	1.381557225	-0.941691731	2.174305412
H	2.102936870	0.722301468	2.093825236

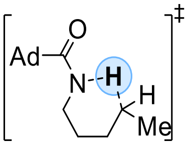

C	-3.679762294	-0.137373037	-1.386254578
C	-3.130201134	1.246052609	-1.745510523

C	-3.176871617	2.145560228	-0.507366039
C	-2.313926662	1.532289189	0.598526449
C	-2.849615077	0.142312965	0.956323896
C	-2.819853784	-0.758347830	-0.282337175
H	-3.679085640	-0.786675418	-2.274491545
H	-4.724043314	-0.054530244	-1.048746118
H	-3.739970448	1.687586230	-2.546697457
H	-4.214846941	2.258670496	-0.159161880
H	-2.809139287	3.152246857	-0.756568860
H	-2.339048119	2.176898089	1.489017692
H	-3.877702273	0.220185550	1.340934194
H	-2.238442562	-0.300682439	1.759598550
H	-3.207294861	-1.754933819	-0.025453909
C	-1.377190087	-0.891548645	-0.775505096
H	-1.330322672	-1.534579241	-1.667811110
H	-0.760363414	-1.369240514	0.000772641
C	-1.685191797	1.110899719	-2.229942183
H	-1.629085108	0.482675675	-3.128344595
H	-1.280249544	2.093617319	-2.514102158
C	-0.867869851	1.404639075	0.112069643
H	-0.239819133	1.000840792	0.918248950
H	-0.455350327	2.394017252	-0.136747503
C	-0.805311377	0.499506651	-1.128966137
C	0.612532234	0.316122109	-1.655065316
O	0.861839942	0.240781702	-2.853034077
N	1.624994011	0.287297097	-0.720667717
C	2.919400512	-0.195997199	-1.158779952
H	2.821210905	-1.047364174	-1.853093121
H	3.406885077	0.608144649	-1.729213144
C	3.763959483	-0.568087240	0.051160551
H	3.933781432	0.326288776	0.667642315
H	4.748248593	-0.917473567	-0.281490573
C	3.063340434	-1.651152675	0.876103753
H	2.947629882	-2.549074529	0.253154197
H	3.694046849	-1.938638299	1.732806682
C	1.708883386	-1.182833253	1.362106216
H	1.366812601	-0.431339681	0.391601992
H	0.953645545	-1.975772215	1.413090093
C	1.702322081	-0.269136304	2.558799563
H	0.685352588	0.032987458	2.834211642
H	2.148370066	-0.765116060	3.433584584
H	2.280638134	0.644030921	2.367853429

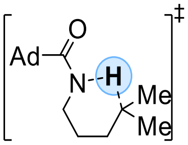

C	-3.707183207	0.134797472	-1.584139739
C	-2.960034403	1.470599278	-1.639141868
C	-2.977930636	2.123541475	-0.254932786
C	-2.292893926	1.197550385	0.752523861
C	-3.041666541	-0.137587158	0.808195395
C	-3.025324529	-0.794259211	-0.575276907

H	-3.717036369	-0.332716494	-2.579899122
H	-4.755822557	0.300198351	-1.294848936
H	-3.445058590	2.134897664	-2.369076417
H	-4.013798857	2.322347913	0.059225308
H	-2.459886716	3.093654699	-0.288416559
H	-2.297100034	1.664905512	1.747907381
H	-4.079502021	0.024783706	1.136333765
H	-2.572458908	-0.803949263	1.549218118
H	-3.559649856	-1.754480303	-0.535662354
C	-1.578494341	-1.039493316	-1.009165944
H	-1.540576849	-1.512696082	-2.000441173
H	-1.081159852	-1.726111332	-0.303481230
C	-1.512841463	1.225509247	-2.065869525
H	-1.466095148	0.770527941	-3.063576862
H	-0.964362292	2.179309506	-2.121530478
C	-0.843368290	0.944788574	0.324787189
H	-0.367418160	0.285458715	1.065064651
H	-0.267652785	1.882313431	0.315765670
C	-0.805054830	0.292859333	-1.062360895
C	0.590760841	0.010136924	-1.604374447
O	0.744106113	-0.368436548	-2.762290801
N	1.671832801	0.282809342	-0.795528918
C	2.961495495	-0.182091937	-1.274863760
H	2.870750144	-1.143097419	-1.807365690
H	3.328027158	0.543214951	-2.015997722
C	3.940075496	-0.292228744	-0.115551268
H	4.090125722	0.698330090	0.337282160
H	4.915908024	-0.621111844	-0.491631213
C	3.407464697	-1.276337715	0.923871125
H	3.302847016	-2.265226600	0.451301433
H	4.128193987	-1.396235371	1.749373696
C	2.063907227	-0.837043118	1.480308501
H	1.589542656	-0.251739887	0.467459151
C	2.153171570	0.261576922	2.512739547
H	1.162443795	0.639351827	2.791223670
H	2.631770261	-0.118789887	3.428773193
H	2.746752821	1.110666259	2.154103633
C	1.151406812	-1.975442190	1.861946289
H	0.971170565	-2.648320778	1.012911299
H	1.611893750	-2.577095122	2.662085412
H	0.181796133	-1.624081126	2.236610136

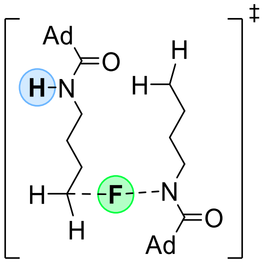

C	10.880108715	-2.099358057	-9.923441079
C	11.827680637	-2.701599610	-8.882302915

C	13.221890158	-2.860948007	-9.496237420
C	13.749921568	-1.492254779	-9.936736686
C	12.799968751	-0.890167779	-10.975646048
C	11.405998421	-0.730070610	-10.362627240
H	9.867745909	-1.999027368	-9.502823647
H	10.800223319	-2.767279520	-10.793923722
H	11.449732345	-3.682577737	-8.561014701
H	13.177506281	-3.543318658	-10.358301521
H	13.907338506	-3.311270321	-8.763340456
H	14.752200723	-1.605603264	-10.373454433
H	12.746294783	-1.538491415	-11.862888802
H	13.178781149	0.086076100	-11.314329359
H	10.722556373	-0.289859026	-11.102610423
C	11.486842509	0.196642652	-9.144051094
H	10.475278629	0.311699754	-8.716894813
H	11.834667340	1.193604953	-9.460189933
C	11.912556749	-1.773853663	-7.669335265
H	10.917864657	-1.662213230	-7.205093724
H	12.581290085	-2.186597768	-6.902070564
C	13.831148995	-0.563562380	-8.724650496
H	14.221958917	0.421788183	-9.024831516
H	14.515937557	-0.961080612	-7.964200346
C	12.439320508	-0.388217871	-8.090946826
C	12.599368590	0.443106065	-6.821566209
O	13.374056872	0.104409111	-5.936511599
N	11.830087783	1.561982705	-6.701447900
C	11.812359818	2.317104736	-5.460756112
H	12.844754729	2.556958544	-5.180924887
H	11.295329956	3.264734853	-5.663144349
C	11.129200955	1.585218400	-4.313490664
H	11.027796871	2.262314452	-3.455880570
H	11.764010107	0.747633848	-3.996379431
C	9.742499099	1.062611407	-4.668420896
H	9.104882016	1.856729394	-5.085618293
H	9.831746519	0.305293795	-5.472586035
C	9.069005523	0.437615710	-3.514825255
H	8.018855707	0.164172303	-3.593150910
H	9.659292431	-0.179714334	-2.839270871
H	11.169598474	1.796127765	-7.425821918
C	5.066820082	-0.192182419	-0.000346403
C	4.589850425	1.166843998	0.518685889
C	4.810980784	1.237376955	2.032900200
C	6.299235675	1.058403908	2.345372543
C	6.770214566	-0.300763221	1.825076289
C	6.556069613	-0.361916544	0.311484307
H	4.899210080	-0.261476056	-1.086288321
H	4.486684891	-1.002176252	0.467468194
H	3.521158854	1.296710311	0.295061645
H	4.222305406	0.455412725	2.536448897
H	4.458447834	2.205099150	2.420005516
H	6.458590525	1.117390162	3.431822639
H	6.213410093	-1.111591181	2.318993522
H	7.834726688	-0.448128560	2.062333081
H	6.903580020	-1.333034082	-0.071151800
C	7.348347197	0.752692185	-0.378783439
H	7.189045684	0.695032106	-1.462561883
H	8.426499539	0.627922007	-0.209882712
C	5.384262406	2.283465422	-0.162953505
H	5.219698105	2.257534640	-1.252921130
H	5.053266773	3.269129509	0.189668194
C	7.095643592	2.169918853	1.662912157
H	8.170006296	2.062551348	1.881369195
H	6.784499214	3.155514402	2.033176867
C	6.887668439	2.123875740	0.129471567
C	7.608083623	3.355850964	-0.409919757
O	7.228419564	4.474507322	-0.068067481
N	8.794416262	3.266287641	-1.100393194
C	9.270585755	4.409840167	-1.849677173
H	9.568770671	5.154713286	-1.097804489
H	10.193130997	4.088763018	-2.351588569
C	8.300543405	5.044715317	-2.838806340
H	8.801945724	5.920594104	-3.279425251
H	7.433771449	5.429113882	-2.286551855
C	7.836560825	4.124618102	-3.958130955
H	8.719963807	3.766430488	-4.512189369
H	7.373398049	3.224927827	-3.526791280
C	6.873369204	4.817825141	-4.906240481
H	7.338448310	5.695318569	-5.374112960
H	6.535855420	4.152679876	-5.709382685
H	5.982462239	5.169422406	-4.370849446
F	8.896380587	1.973499423	-2.245470398

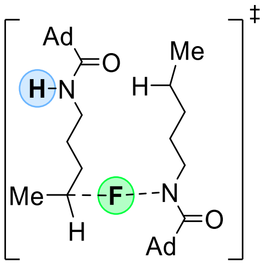

C	8.405465427	-1.554385081	-10.636447006
C	9.809914957	-1.952166810	-10.175766247
C	10.795431531	-1.776343979	-11.335077216
C	10.801515688	-0.313134916	-11.788625299
C	9.394954849	0.082585033	-12.247477931
C	8.410607510	-0.091250649	-11.087192366

H	7.683849557	-1.696262413	-9.817514376
H	8.080197517	-2.201466545	-11.464914678
H	9.808407993	-3.001085581	-9.846778732
H	10.510784353	-2.430174065	-12.173379942
H	11.805292161	-2.077416717	-11.019981086
H	11.510585578	-0.186180479	-12.618692232
H	9.083363287	-0.541619118	-13.098539369
H	9.389184465	1.126630280	-12.595686014
H	7.401325394	0.201284780	-11.409766795
C	8.839298464	0.799955299	-9.915217698
H	8.117454335	0.679996498	-9.088557637
H	8.815531011	1.855803935	-10.231779909
C	10.237289759	-1.059939169	-9.009714021
H	9.547262980	-1.184216353	-8.157882379
H	11.239408995	-1.329662164	-8.651869607
C	11.227612739	0.580102624	-10.621166913
H	11.249504783	1.635493932	-10.936478758
H	12.238920905	0.326086070	-10.276534503
C	10.249807262	0.418902826	-9.442528140
C	10.785200533	1.226425628	-8.263152366
O	11.889186934	0.992629444	-7.790261546
N	9.983788177	2.205629351	-7.753970038
C	10.346114093	2.894355728	-6.526345083
H	11.396646795	3.195199752	-6.604854778
H	9.739897008	3.809191602	-6.465602232
C	10.144889420	2.031626121	-5.286081593
H	10.401936614	2.615807611	-4.390258511
H	10.851276572	1.192407253	-5.336764265
C	8.716532047	1.517997849	-5.155587263
H	8.001511502	2.354409103	-5.225887118
H	8.493515428	0.866952964	-6.026384205
C	8.439809048	0.740617418	-3.921026100
H	7.388825175	0.551179382	-3.702712341
H	9.050647392	2.323147110	-8.117780281
C	4.502787619	0.439888120	-0.104883757
C	4.465357893	1.706130126	0.754628760
C	4.916737083	1.367152136	2.178750533
C	6.342966504	0.809499858	2.148057567
C	6.380077342	-0.452598422	1.284116238
C	5.932771633	-0.106343344	-0.137746082
H	4.162030094	0.666622870	-1.126661539
H	3.816712976	-0.317506114	0.303319132
H	3.443251610	2.110477376	0.777934178
H	4.232909807	0.629561417	2.625231330
H	4.877596250	2.266807899	2.810783771
H	6.670238693	0.571608700	3.170345134
H	5.721879715	-1.224379840	1.710806153
H	7.398384597	-0.869960977	1.268880413
H	5.968552018	-1.009099601	-0.765442973
C	6.872196416	0.946060502	-0.735197819
H	6.561321750	1.184846315	-1.761311376
H	7.896425378	0.554133009	-0.795249530
C	5.405357708	2.757098661	0.160664405
H	5.084388563	3.023476728	-0.859552486
H	5.384343624	3.679095213	0.755105262
C	7.283492774	1.859137250	1.555636383
H	8.318516090	1.482917618	1.533980976
H	7.280720965	2.771867299	2.166451178
C	6.848268301	2.220724112	0.114744934
C	7.762514232	3.369311785	-0.301292229
O	7.660732404	4.457548822	0.256155200
N	8.862636594	3.178831269	-1.113527977
C	9.555767126	4.320022955	-1.677045893
H	10.051034689	4.813742603	-0.829797077
H	10.352225564	3.906107014	-2.308557954
C	8.709544555	5.332148168	-2.438258413
H	9.383572934	6.132057936	-2.780594232
H	8.006392275	5.799445645	-1.737887406
C	7.952899046	4.767901704	-3.630100237
H	8.666393108	4.304945392	-4.333487167
H	7.293870606	3.948461997	-3.300426513
C	7.135041822	5.823914094	-4.357672229
H	7.797161468	6.643123330	-4.677903575
H	6.421111765	6.275928568	-3.653069381
F	8.626973459	2.163414159	-2.388234022
C	9.427053408	-0.225918386	-3.384016279
H	9.644015228	-1.020468277	-4.117380252
H	10.380837910	0.265618345	-3.156932086
H	9.064318002	-0.702221634	-2.468198236
C	6.391598084	5.260552007	-5.557492429
H	5.786446288	6.020199149	-6.064016855
H	7.091019018	4.848040976	-6.297450109
H	5.718870220	4.446764342	-5.256355517

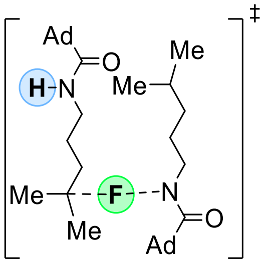

C	11.483454395	-1.038714981	-10.250025878
C	12.030568869	-2.073578923	-9.263380938
C	13.462366713	-2.447444140	-9.658677584
C	14.347129420	-1.197373160	-9.631123575
C	13.796143893	-0.162537913	-10.616192807
C	12.365231594	0.212515448	-10.220067410
H	10.446103740	-0.777961339	-9.989761050

H	11.461949154	-1.458604599	-
	11.266844698		
H	11.395976931	-2.971231847	-9.278643278
H	13.470527246	-2.893954429	-
	10.664500358		
H	13.858451739	-3.205327865	-8.966778238
H	15.375365216	-1.465376111	-9.912577004
H	13.807045848	-0.568258345	-
	11.638958056		
H	14.435028114	0.733698985	-
	10.620412958		
H	11.970462254	0.961891985	-
	10.920906007		
C	12.364757493	0.801810282	-8.804241234
H	11.332233646	1.080556312	-8.530445954
H	12.976935309	1.718504895	-8.789673185
C	12.033487550	-1.482824406	-7.852851626
H	11.006339379	-1.222062161	-7.545915462
H	12.412535284	-2.208062311	-7.120735000
C	14.348278761	-0.605960930	-8.220365937
H	14.992544315	0.286879751	-8.180923970
H	14.747132187	-1.322742849	-7.490231729
C	12.917301283	-0.221423669	-7.801152354
C	12.957354670	0.245923901	-6.348534943
O	13.369011049	-0.489291667	-5.460842682
N	12.513813611	1.506692689	-6.081951280
C	12.366110908	1.970077339	-4.711708152
H	13.310257456	1.794032940	-4.183373362
H	12.202913017	3.057133852	-4.752213833
C	11.221555050	1.286565692	-3.970919808
H	11.100036058	1.752249938	-2.983231513
H	11.496904244	0.237467906	-3.804031813
C	9.907375484	1.376761179	-4.732584715
H	9.734997247	2.407627523	-5.083891334
H	9.989740343	0.770969029	-5.660056621
C	8.690474089	0.911989706	-4.009220580
H	12.106181772	2.056547981	-6.822464877
C	4.816307402	0.716559022	-1.604028629
C	4.465147444	2.170372259	-1.937618008
C	3.502883404	2.708470625	-0.875443993
C	4.175099873	2.643358894	0.499681018
C	4.523611587	1.189004069	0.828338447
C	5.487495263	0.646828488	-0.229547942
H	5.486351289	0.295865577	-2.370495314
H	3.903777137	0.101173471	-1.611534546
H	3.992278142	2.217389865	-2.930147323
H	2.574113013	2.117908626	-0.871045948
H	3.223465260	3.746397233	-1.110779717

H	3.490227025	3.040762650	1.262446773
H	3.609223418	0.576884285	0.857729707
H	4.984033127	1.127570013	1.825857715
H	5.746714458	-0.397014479	0.000945494
C	6.766874654	1.487629453	-0.244826586
H	7.478012352	1.096731732	-0.980893246
H	7.263317998	1.441423869	0.736766394
C	5.735274104	3.025649298	-1.948039787
H	6.433333046	2.687217597	-2.722011248
H	5.484805822	4.075903763	-2.167564540
C	5.451985654	3.486172177	0.486707524
H	5.935740832	3.467453003	1.474551990
H	5.222825190	4.538788269	0.274864124
C	6.429946558	2.952721294	-0.572633739
C	7.658305786	3.846847930	-0.595182153
O	7.662618870	4.970028822	-0.106185547
N	8.861877822	3.335487814	-1.051720508
C	9.959266996	4.225690585	-1.355041988
H	10.250044375	4.649634421	-0.383638546
H	10.795383477	3.588816525	-1.676569168
C	9.719497832	5.348240750	-2.356019482
H	10.625837903	5.970966825	-2.347078207
H	8.898935066	5.983823927	-1.998161084
C	9.446604279	4.858840206	-3.768740298
H	10.279427427	4.202007411	-4.078587844
H	8.551527844	4.216822051	-3.762440819
C	9.284088623	5.952109426	-4.821885017
H	8.421822011	6.578315623	-4.536545305
F	8.691969048	2.340426823	-2.366963889
C	8.775780600	-0.303407816	-3.157675432
H	9.006105729	-1.191108991	-3.772172258
H	9.568885524	-0.218343439	-2.406767936
H	7.829822229	-0.496368682	-2.637533164
C	8.995649609	5.318928488	-6.177350525
H	8.834566913	6.074818008	-6.955433401
H	9.848866764	4.697479069	-6.493781527
H	8.108460586	4.673755941	-6.145511811
C	7.399538385	1.208810455	-4.685866438
H	7.343265640	2.257248113	-5.007257660
H	7.298948393	0.590825708	-5.595863534
H	6.533127429	0.987703912	-4.052229725
C	10.515886547	6.843863564	-4.920592010
H	11.411897981	6.234064560	-5.114567491
H	10.421243800	7.561545856	-5.744483080
H	10.694954428	7.417498586	-4.004679759

References

1. A. B. Pangborn, M. A. Giardello, R. H. Grubbs, R. K. Rosen and F. J. Timmers, *Organometallics*, 1996, **15**, 1518-1520.
2. C. P. Rosenau, B. J. Jelier, A. D. Gossert and A. Togni, *Angew. Chem. Int. Ed.*, 2018, **57**, 9528-9533.
3. T. T. Nguyen and K. L. Hull, *ACS Catal.*, 2016, **6**, 8214-8218.
4. A. P. Haering, P. Biallas and S. F. Kirsch, *Eur. J. Org. Chem.*, 2017, **2017**, 1526-1539.
5. D. H. R. Barton and J. A. Ferreira, *Tetrahedron*, 1996, **52**, 9347-9366.
6. K. Wang, Y. Lu and K. Ishihara, *Chem. Commun*, 2018, **54**, 5410-5413.
7. I.-H. Kim, Y.-K. Park, B. D. Hammock and K. Nishi, *J. Med. Chem.*, 2011, **54**, 1752-1761.
8. J. Castilla, R. Risquez, D. Cruz, K. Higaki, E. Nanba, K. Ohno, Y. Suzuki, Y. Diaz, C. Ortiz Mellet, J. M. Garcia Fernandez and S. Castillon, *J. Med. Chem.*, 2012, **55**, 6857-6865.
9. G. R. Newkome, K. K. Kotta and C. N. Moorefield, *Chem. - Eur. J.*, 2006, **12**, 3726-3734.
10. W. S. Bechara, G. Pelletier and A. B. Charette, *Nature Chemistry*, 2012, **4**, 228-234.
11. A. D. Bochevarov, E. Harder, T. F. Hughes, J. R. Greenwood, D. A. Braden, D. M. Philipp, D. Rinaldo, M. D. Halls, J. Zhang and R. A. Friesner, *Int. J. Quantum Chem.*, 2013, **113**, 2110-2142.
12. Y. Zhao and D. G. Truhlar, *Theor. Chem. Acc.*, 2008, **120**, 215-241.
13. W. R. Wadt and P. J. Hay, *J. Chem. Phys.*, 1985, **82**, 284-298.
14. T. H. Dunning, *J. Chem. Phys.*, 1989, **90**, 1007-1023.
15. (*a*) B. Marten, K. Kim, C. Cortis, R. A. Friesner, R. B. Murphy, M. N. Ringnalda, D. Sitkoff and B. Honig, *J. Phys. Chem.*, 1996, **100**, 11775-11788; (*b*) M. Friedrichs, R. Zhou, S. R. Edinger and R. A. Friesner, *J. Phys. Chem. B*, 1999, **103**, 3057-3061; (*c*) S. R. Edinger, C. Cortis, P. S. Shenkin and R. A. Friesner, *J. Phys. Chem. B*, 1997, **101**, 1190-1197.

NMR Spectra

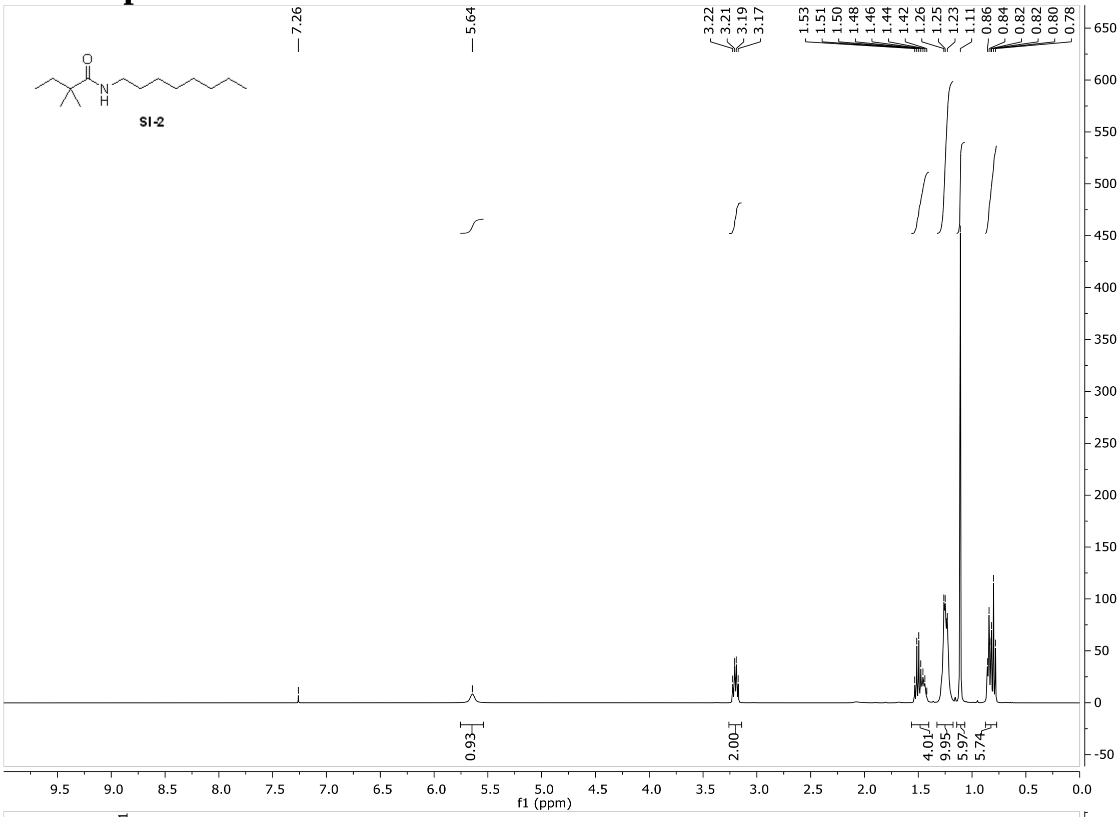
SI-2
7.26
5.64
3.22
3.21
3.19
3.17
1.53
1.51
1.50
1.48
1.46
1.44
1.42
1.26
1.25
1.23
1.11
0.86
0.84
0.82
0.82
0.80
0.78
0.93
2.00
4.01
9.95
5.97
5.74
f1 (ppm)

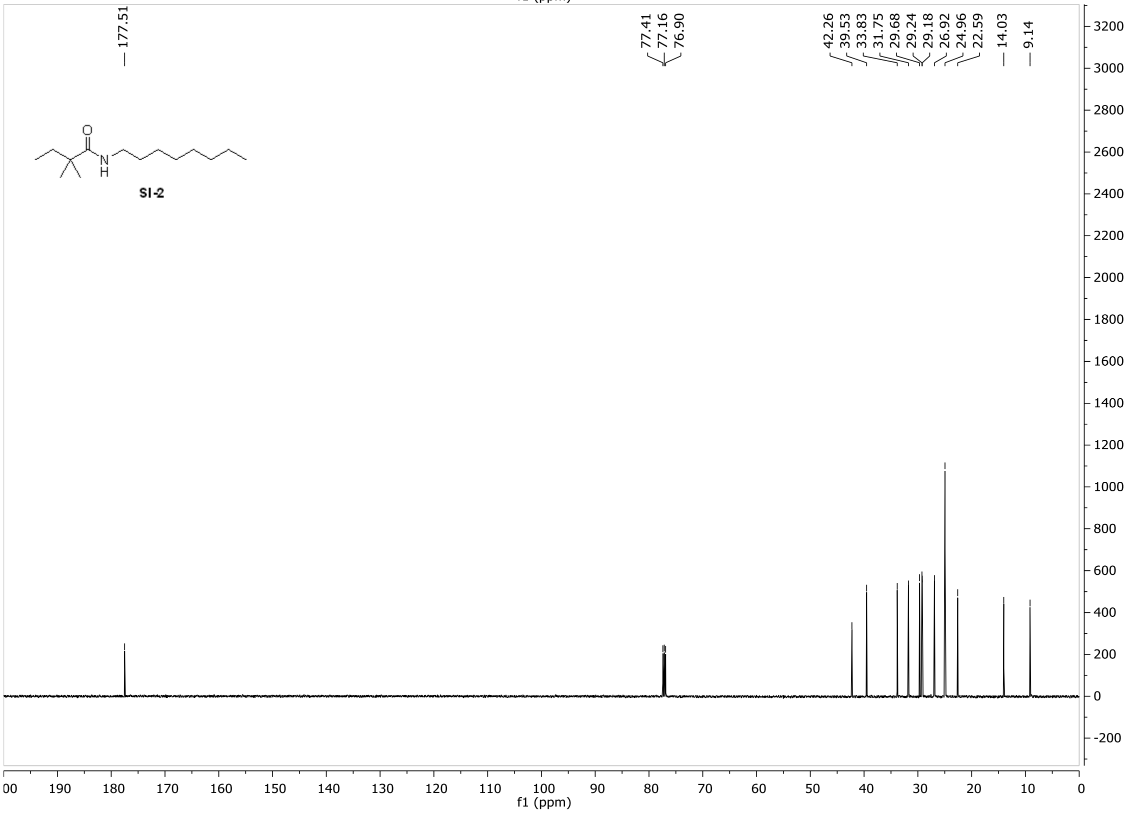
SI-2
177.51
77.41
77.16
76.90
42.26
39.53
33.83
31.75
29.68
29.24
29.18
26.92
24.96
22.59
14.03
9.14
f1 (ppm)

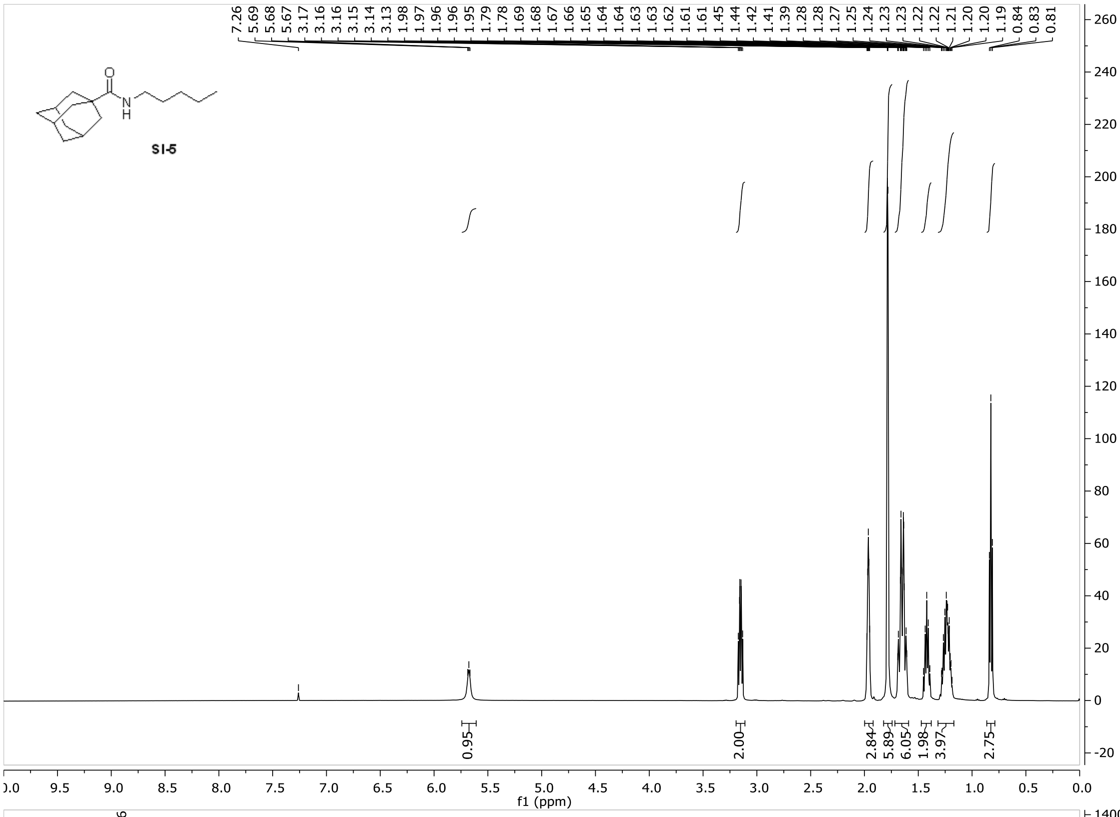
SI-5
7.26 5.69 5.68 5.67 3.17 3.16 3.16 3.15 3.14 3.13 1.98 1.97 1.96 1.96 1.95 1.79 1.78 1.69 1.68 1.67 1.66 1.65 1.64 1.64 1.63 1.63 1.62 1.61 1.61 1.45 1.44 1.42 1.41 1.39 1.28 1.28 1.27 1.25 1.24 1.23 1.23 1.22 1.22 1.21 1.20 1.20 1.19 0.84 0.83 0.81
0.95 2.00 2.84 5.89 6.05 1.98 3.97 2.75
f1 (ppm)

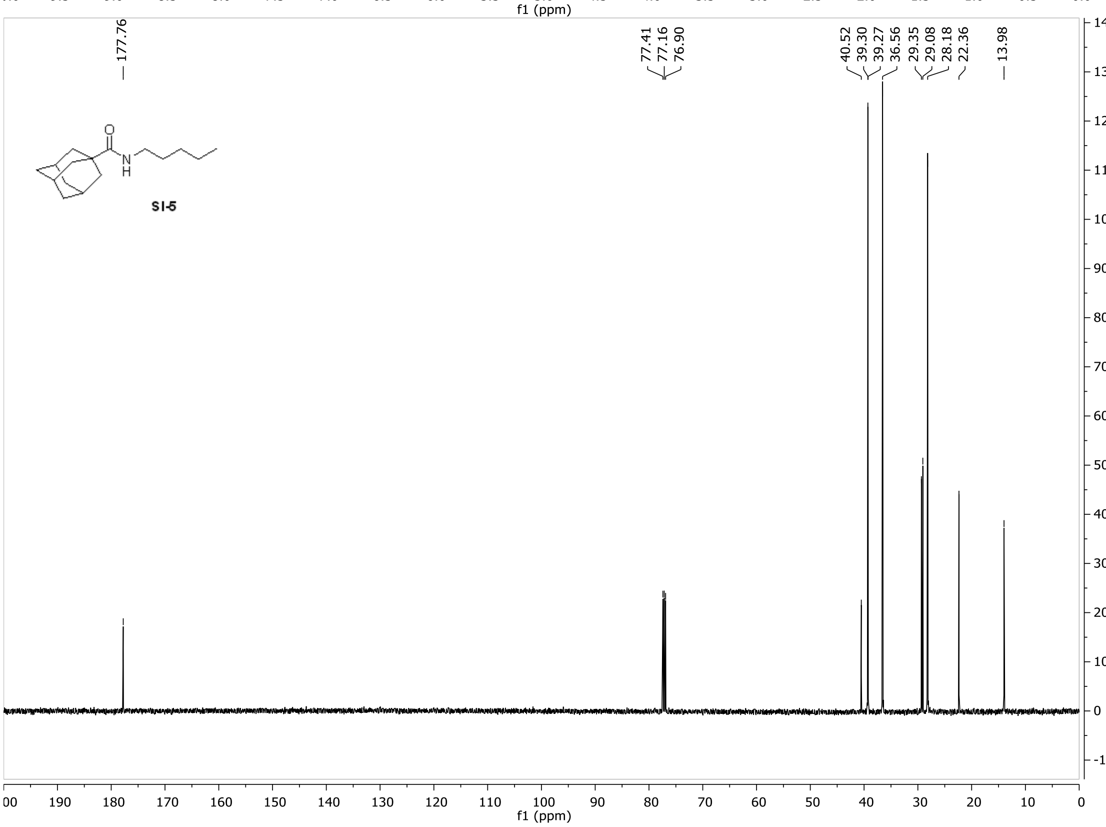
SI-5
177.76
77.41 77.16 76.90
40.52 39.30 39.27 36.56 29.35 29.08 28.18 22.36
13.98
f1 (ppm)

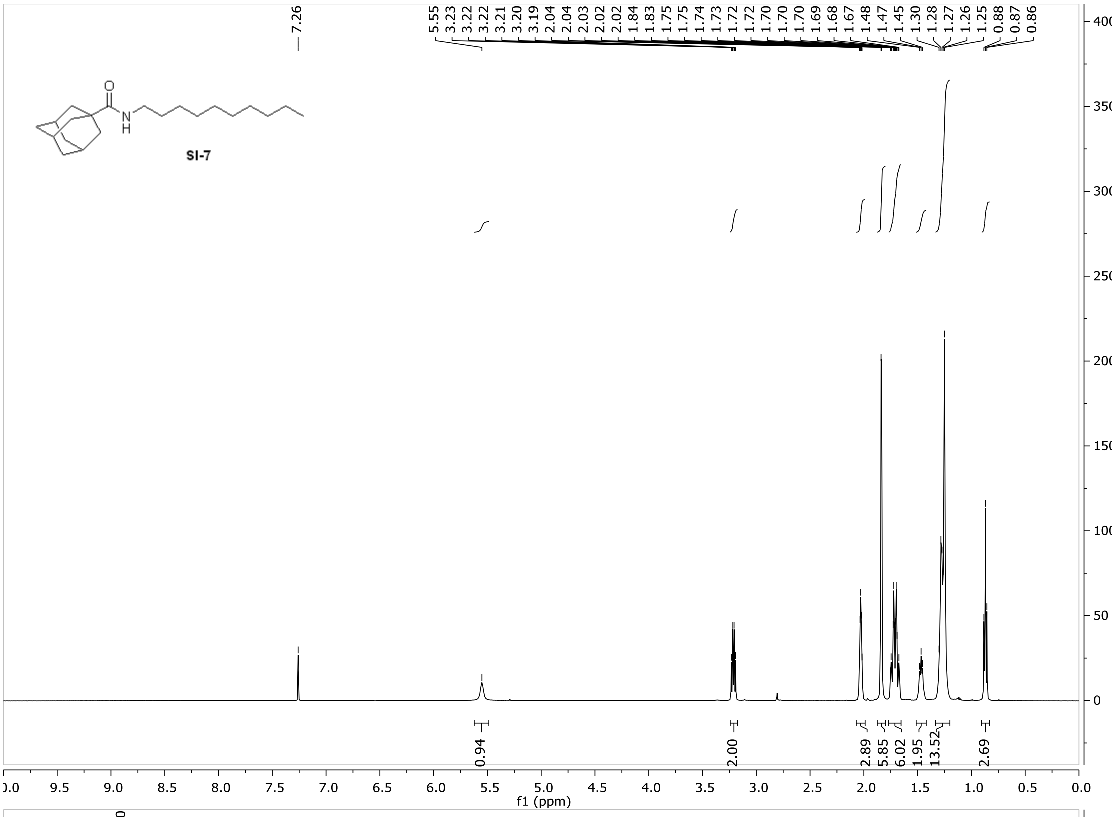
SI-7
7.26
5.55 3.23 3.22 3.22 3.21 3.20 3.19 2.04 2.04 2.03 2.02 2.02 1.84 1.83 1.75 1.75 1.74 1.73 1.72 1.72 1.70 1.70 1.70 1.69 1.68 1.67 1.48 1.47 1.45 1.30 1.28 1.27 1.26 1.25 0.88 0.87 0.86
0.94
2.00
2.89
5.85
6.02
1.95
13.52
2.69
f1 (ppm)

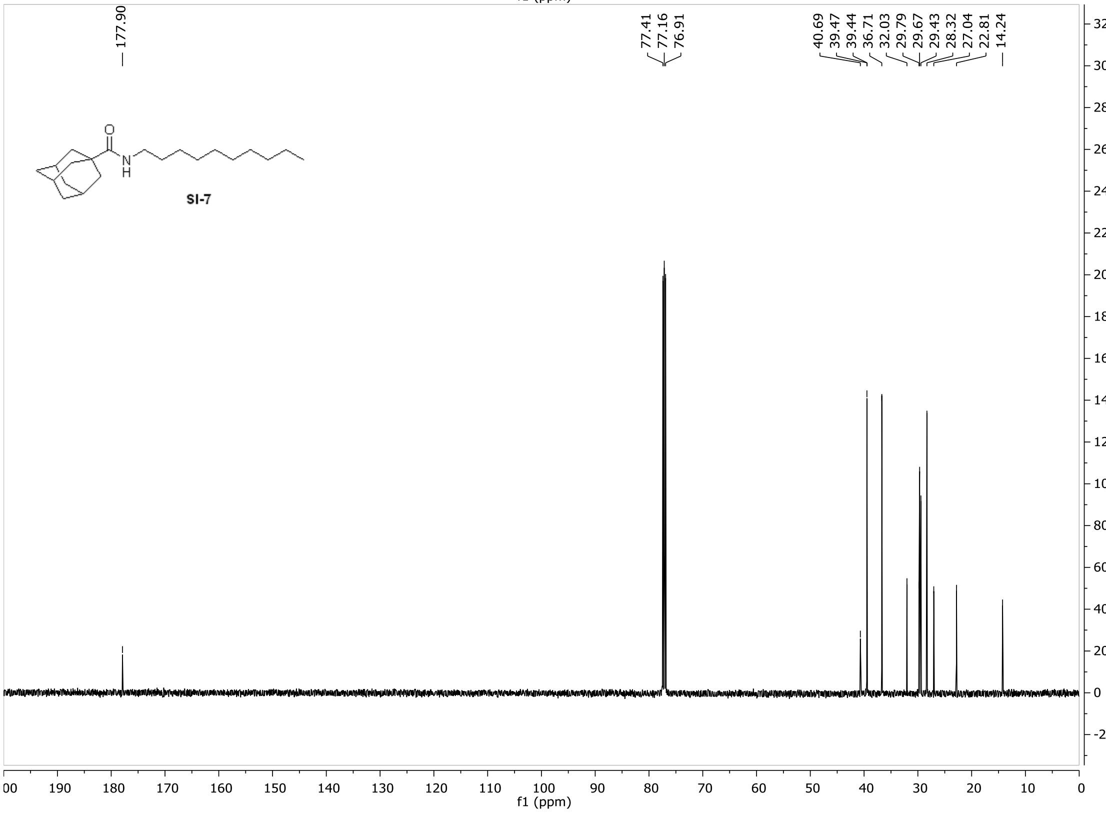
SI-7
177.90
77.41 77.16 76.91
40.69 39.47 39.44 36.71 32.03 29.79 29.67 29.43 28.32 27.04 22.81
14.24
f1 (ppm)

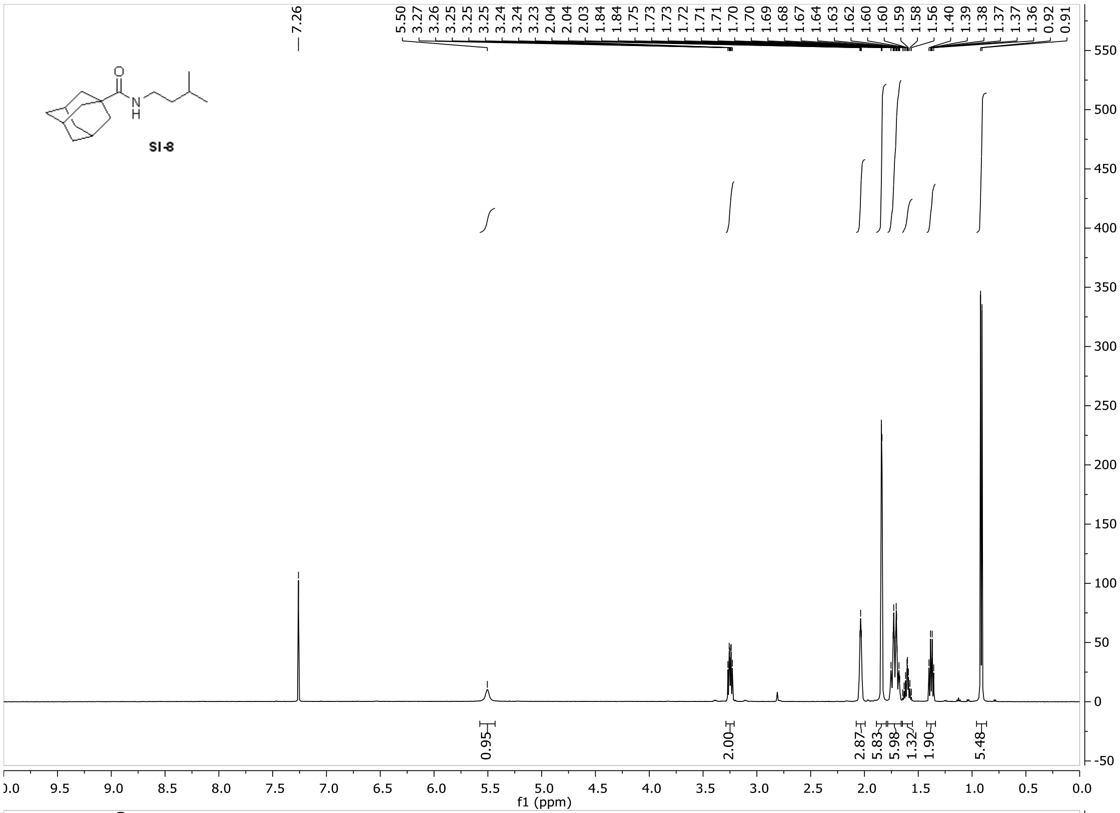
SI-8
7.26
5.50
3.27
3.26
3.25
3.25
3.25
3.24
3.24
3.23
2.04
2.04
2.03
1.84
1.84
1.75
1.73
1.73
1.72
1.71
1.71
1.70
1.70
1.69
1.68
1.67
1.64
1.63
1.62
1.60
1.60
1.59
1.58
1.56
1.40
1.39
1.38
1.37
1.37
1.36
0.92
0.91
0.95
2.00
2.87
5.83
5.98
1.32
1.90
5.48
f1 (ppm)

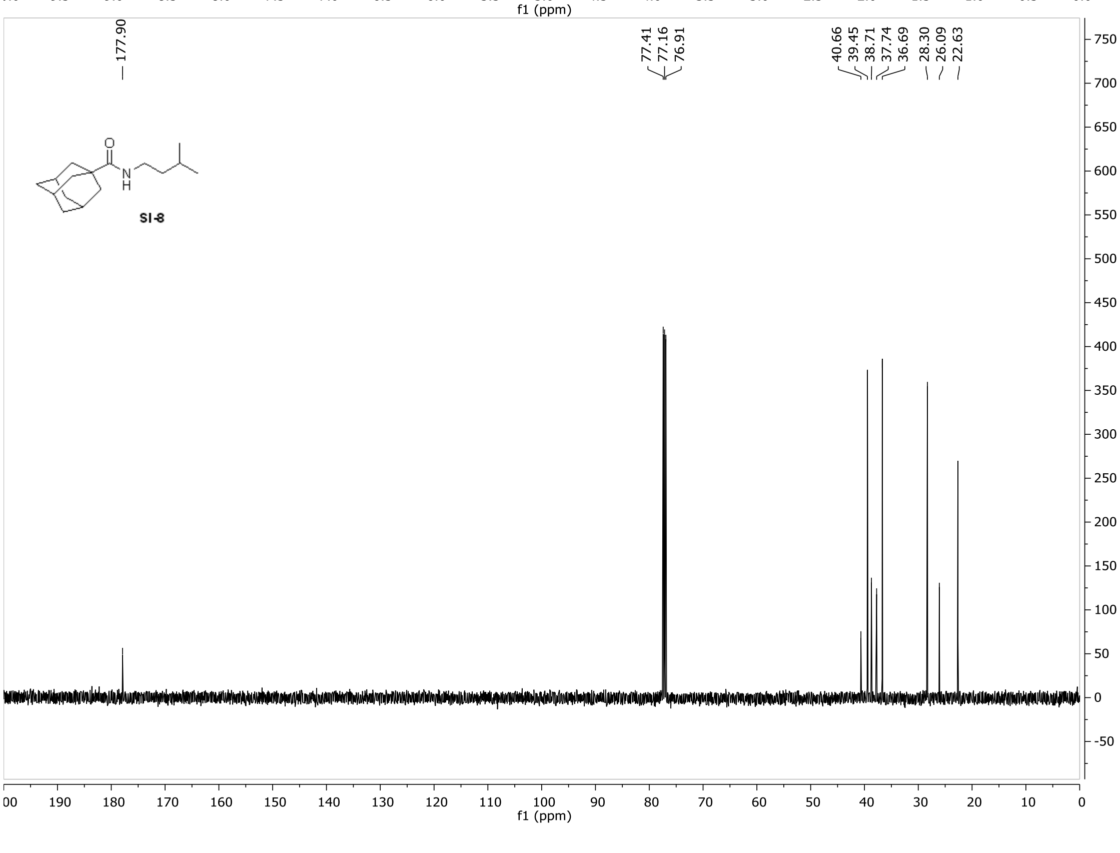
SI-8
177.90
77.41
77.16
76.91
40.66
39.45
38.71
37.74
36.69
28.30
26.09
22.63
f1 (ppm)

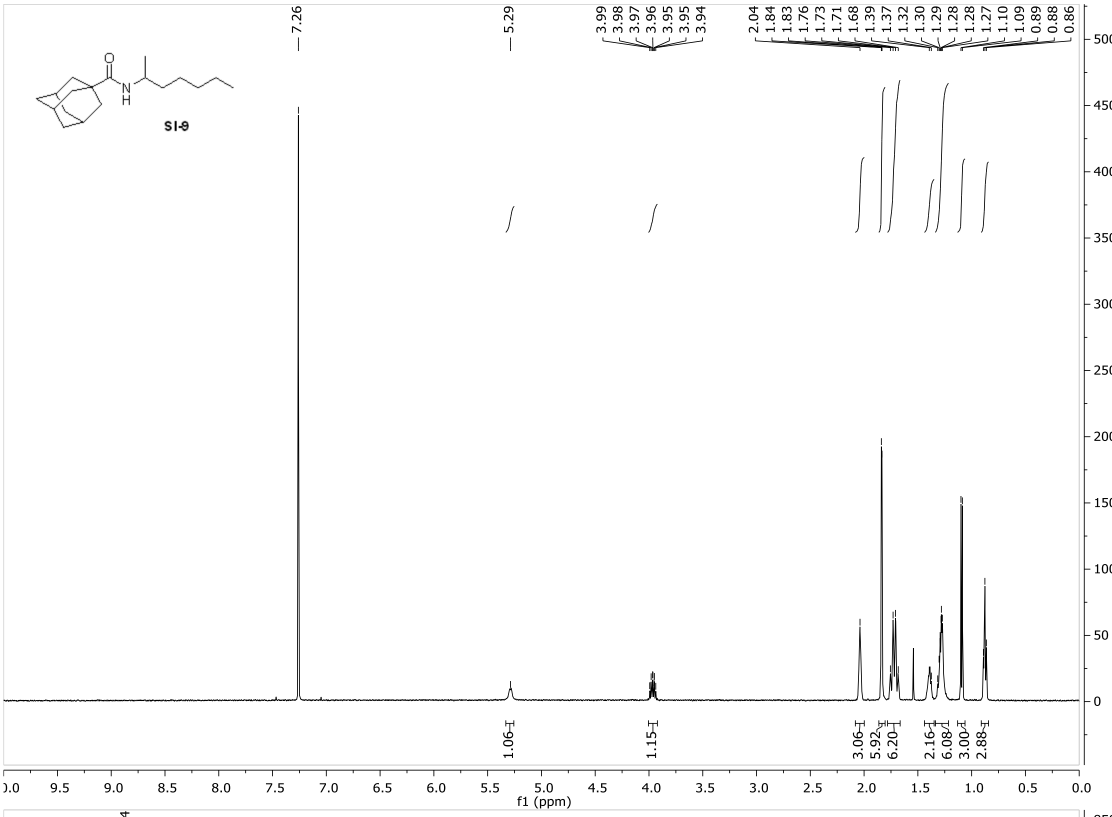
SI-9
7.26
5.29
3.99 3.98 3.97 3.96 3.95 3.95 3.94
2.04 1.84 1.83 1.76 1.73 1.71 1.68 1.39 1.37 1.32 1.30 1.29 1.28 1.28 1.27 1.10 1.09 0.89 0.88 0.86
1.06 1.15 3.06 5.92 6.20 2.16 6.08 3.00 2.88
f1 (ppm)

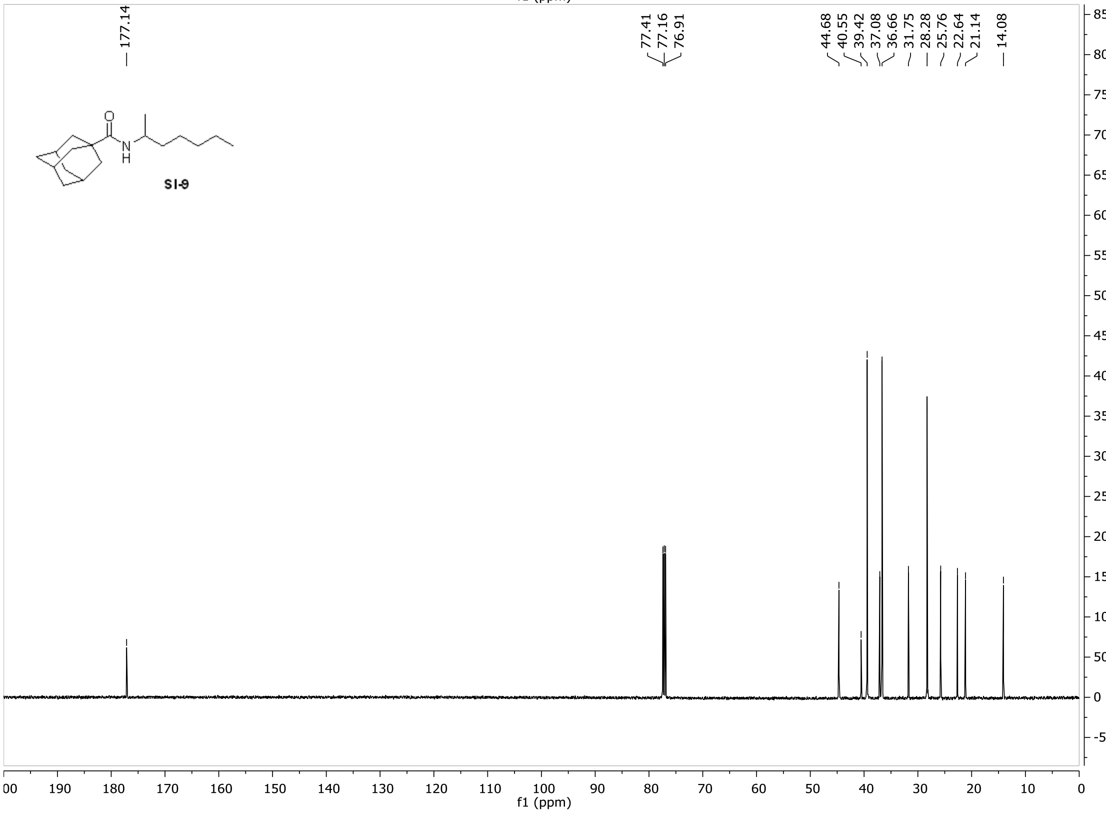
SI-9
177.14
77.41 77.16 76.91
44.68 40.55 39.42 37.08 36.66 31.75 28.28 25.76 22.64 21.14 14.08
f1 (ppm)

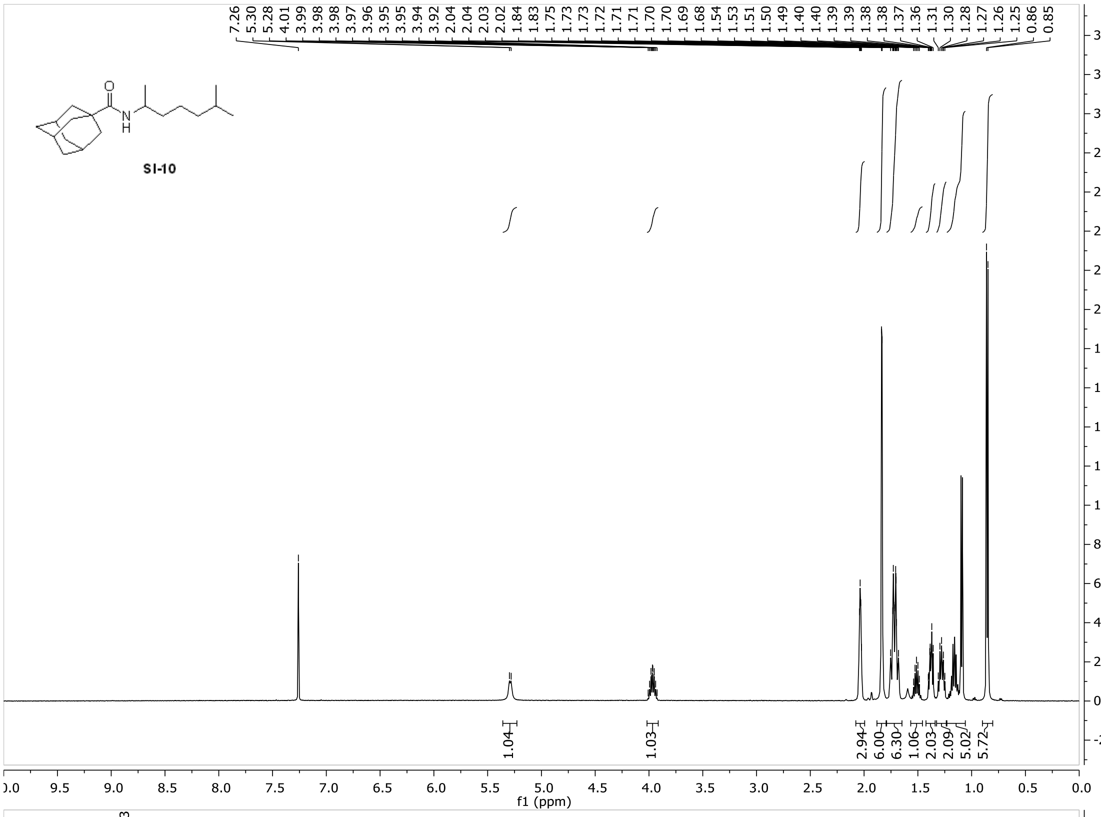
7.26 5.30 5.28 4.01 3.99 3.98 3.98 3.97 3.96 3.95 3.95 3.94 3.92 2.04 2.04 2.03 2.02 1.84 1.83 1.75 1.73 1.73 1.72 1.71 1.71 1.70 1.70 1.69 1.68 1.54 1.53 1.51 1.50 1.49 1.40 1.40 1.39 1.39 1.38 1.38 1.37 1.36 1.31 1.30 1.28 1.27 1.26 1.25 0.86 0.85
SI-10
1.04 1.03 2.94 6.00 6.30 1.06 2.03 2.09 5.02 5.72
f1 (ppm)

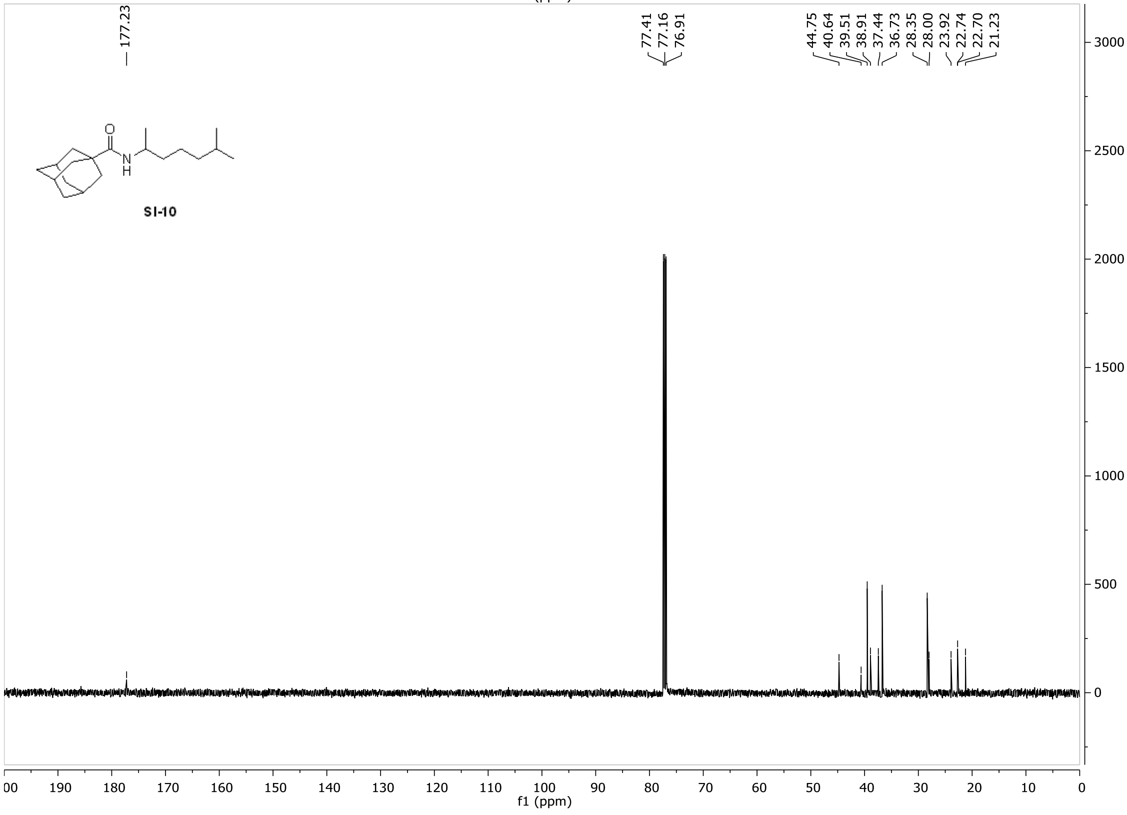
177.23
77.41 77.16 76.91
44.75 40.64 39.51 38.91 37.44 36.73 28.35 28.00 23.92 22.74 22.70 21.23
SI-10
f1 (ppm)

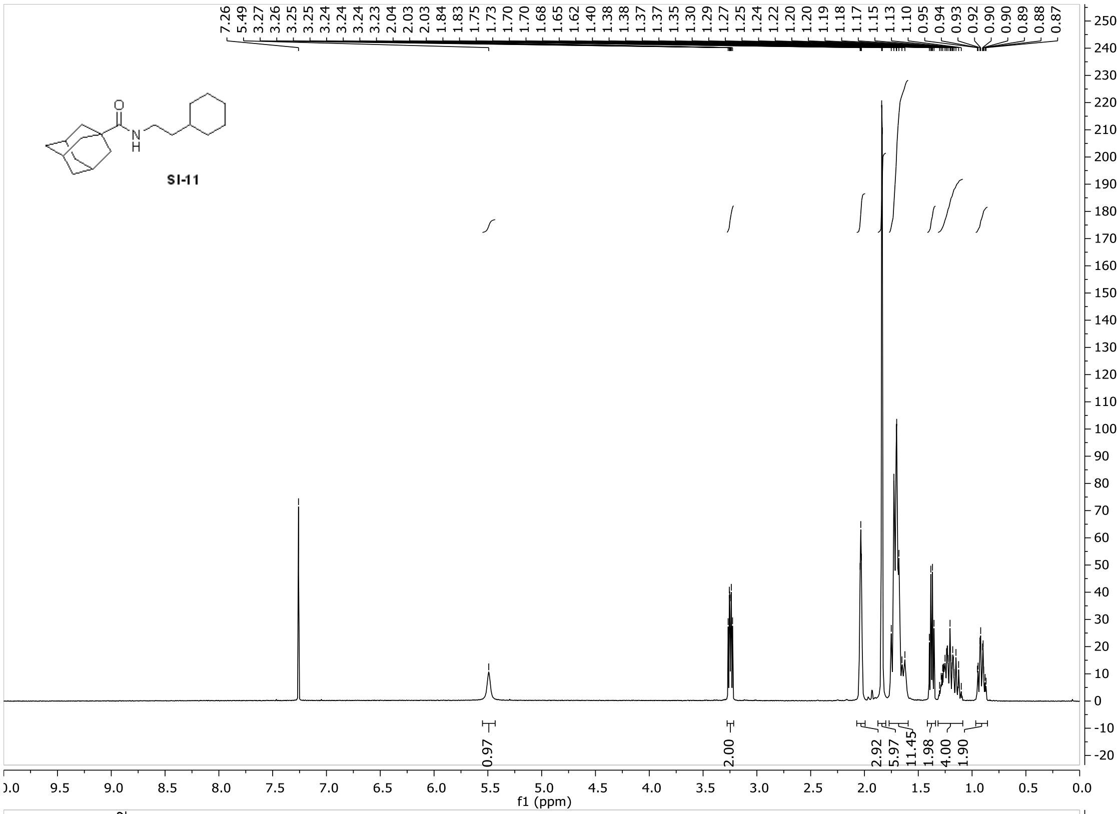

7.26
5.49
3.27
3.26
3.25
3.25
3.24
3.24
3.24
3.23
2.04
2.03
2.03
1.84
1.83
1.75
1.73
1.70
1.70
1.68
1.65
1.62
1.40
1.38
1.38
1.37
1.37
1.35
1.30
1.29
1.27
1.25
1.24
1.22
1.20
1.20
1.19
1.18
1.17
1.15
1.13
1.10
0.95
0.94
0.93
0.92
0.90
0.90
0.89
0.88
0.87
O
N
H
SI-11
0.97
2.00
2.92
5.97
11.45
1.98
4.00
1.90
f1 (ppm)

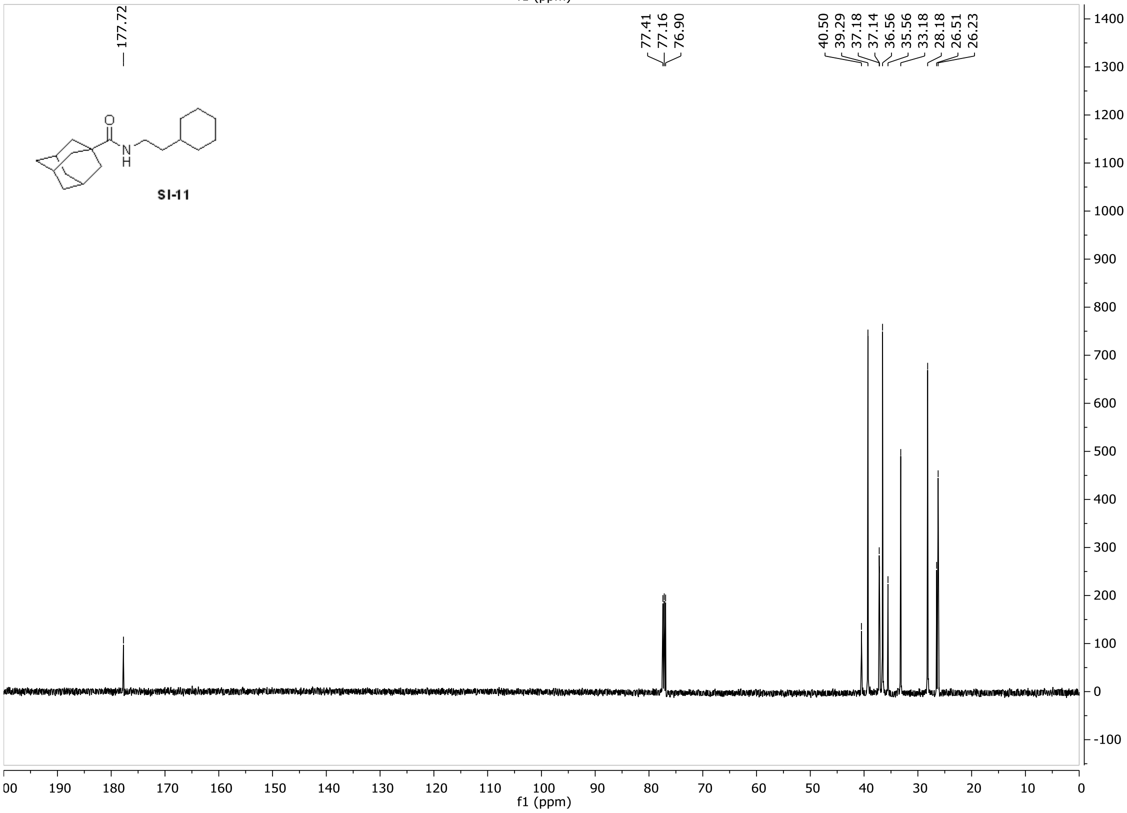

177.72
77.41
77.16
76.90
40.50
39.29
37.18
37.14
36.56
35.56
33.18
28.18
26.51
26.23
O
N
H
SI-11
f1 (ppm)

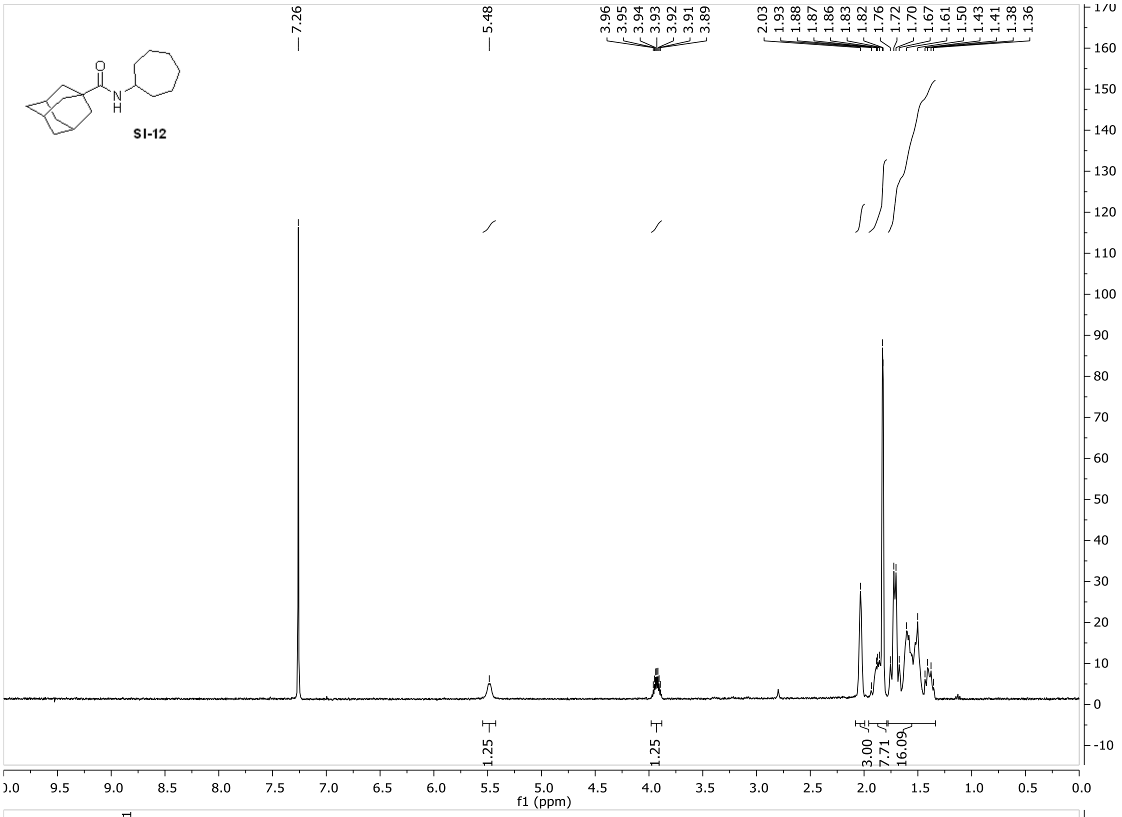
7.26
5.48
3.96 3.95 3.94 3.93 3.92 3.91 3.89
2.03 1.93 1.88 1.87 1.86 1.83 1.82 1.76 1.72 1.70 1.67 1.61 1.50 1.43 1.41 1.38 1.36
SI-12
1.25
1.25
3.00
7.71
16.09
f1 (ppm)

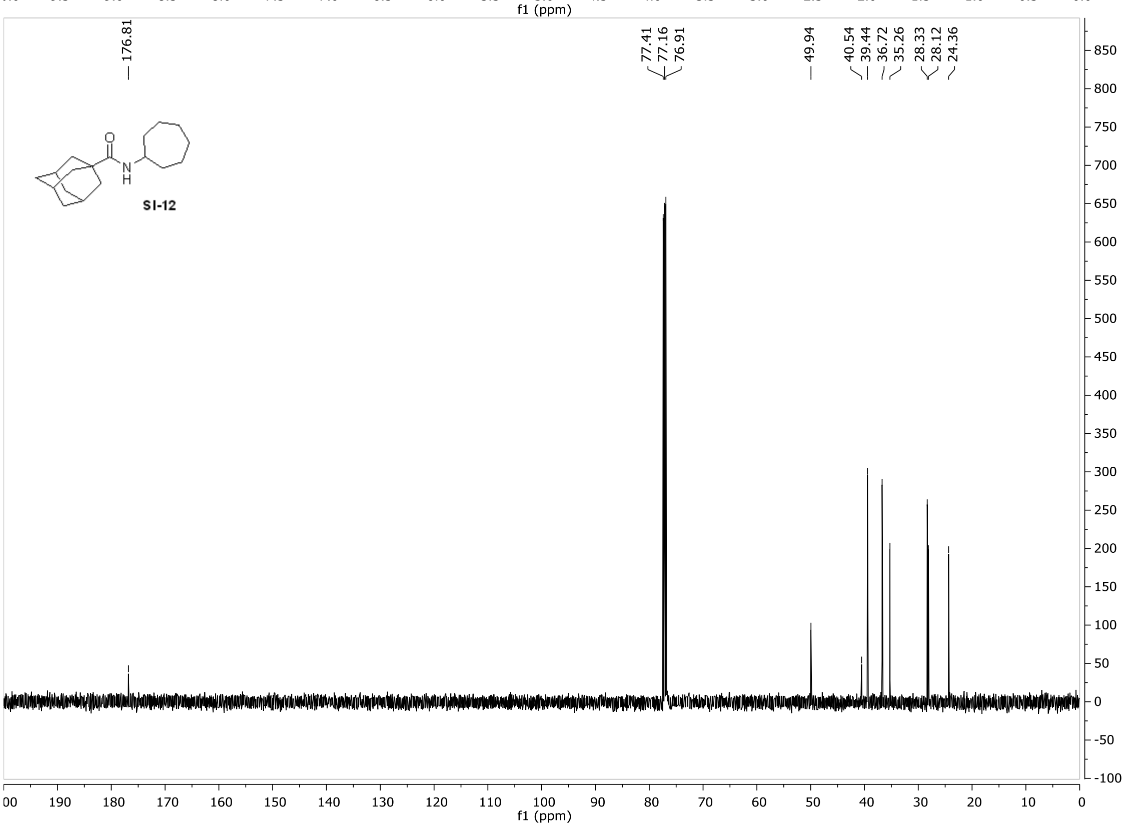
176.81
77.41 77.16 76.91
49.94
40.54 39.44 36.72 35.26 28.33 28.12 24.36
SI-12
f1 (ppm)

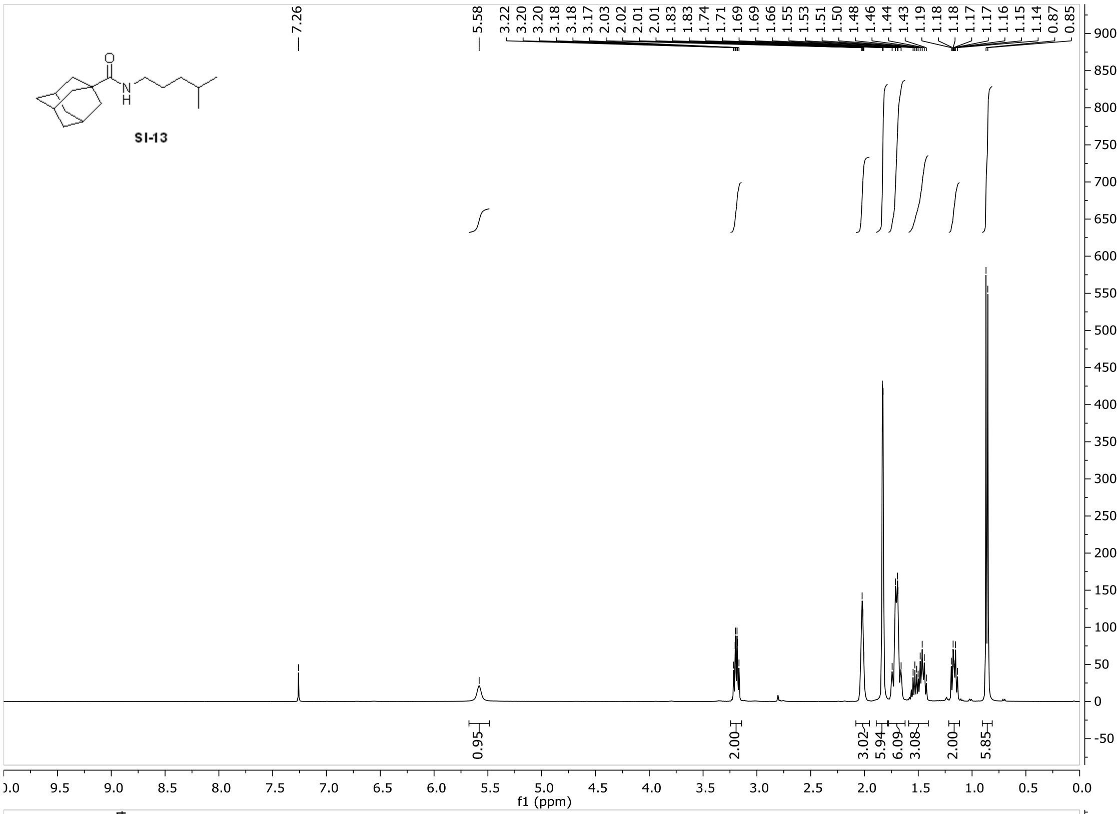
SI-13
f1 (ppm)

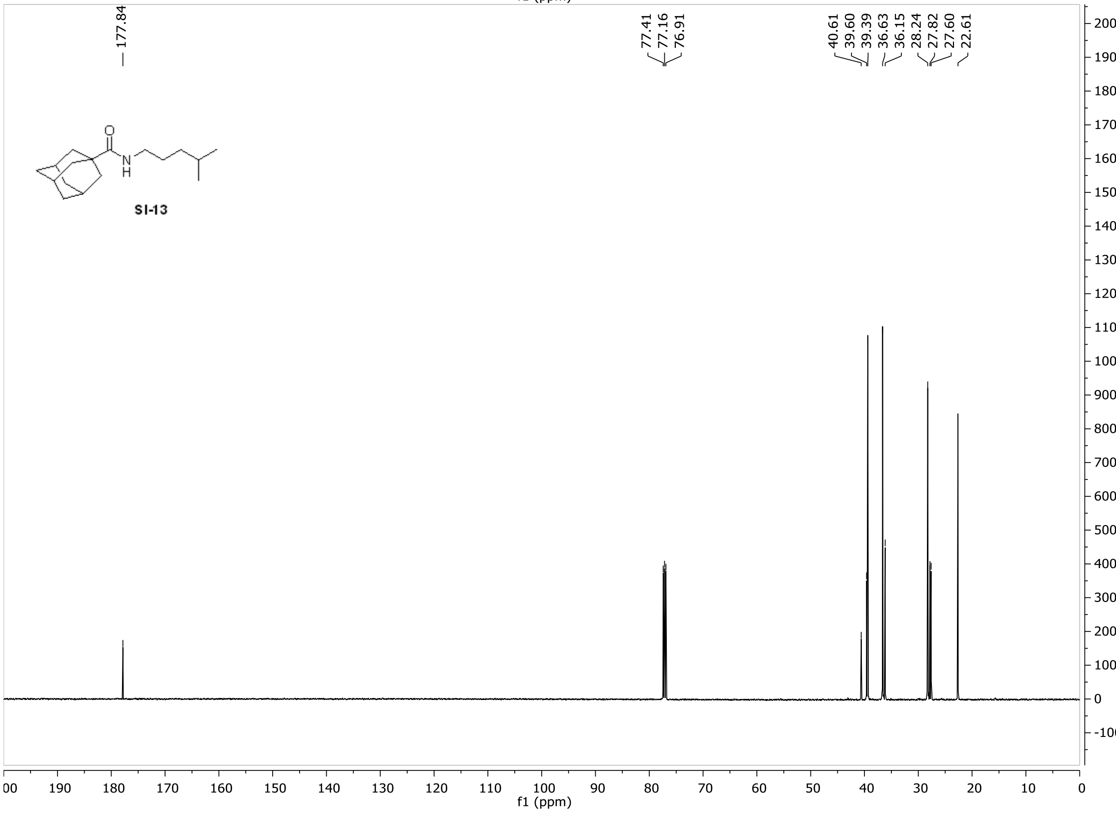
SI-13
f1 (ppm)

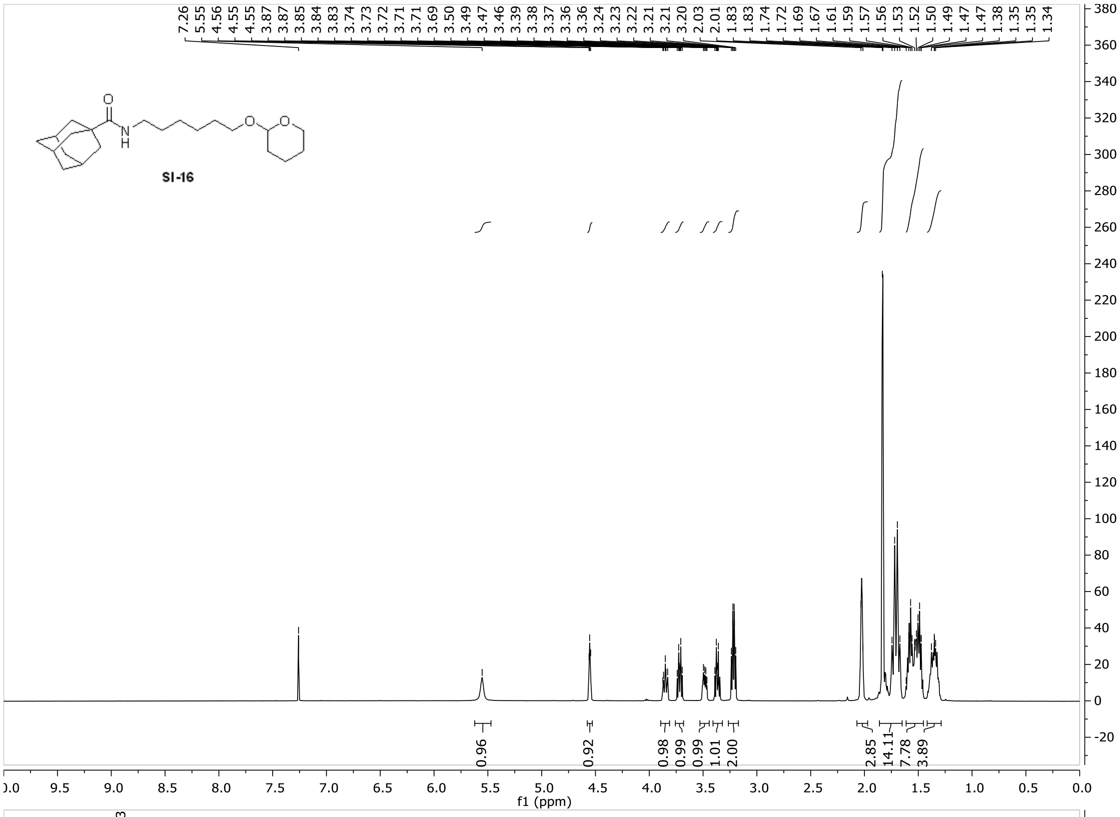
7.26 5.55 4.56 4.55 4.55 3.87 3.85 3.84 3.83 3.74 3.73 3.72 3.71 3.69 3.50 3.49 3.47 3.46 3.39 3.38 3.37 3.36 3.36 3.24 3.23 3.22 3.21 3.21 3.20 2.03 2.01 1.83 1.83 1.74 1.72 1.69 1.67 1.61 1.59 1.57 1.56 1.53 1.52 1.50 1.49 1.47 1.47 1.38 1.35 1.35 1.34
SI-16
0.96 0.92 0.98 0.99 0.99 1.01 2.00 2.85 14.11 7.78 3.89
f1 (ppm)

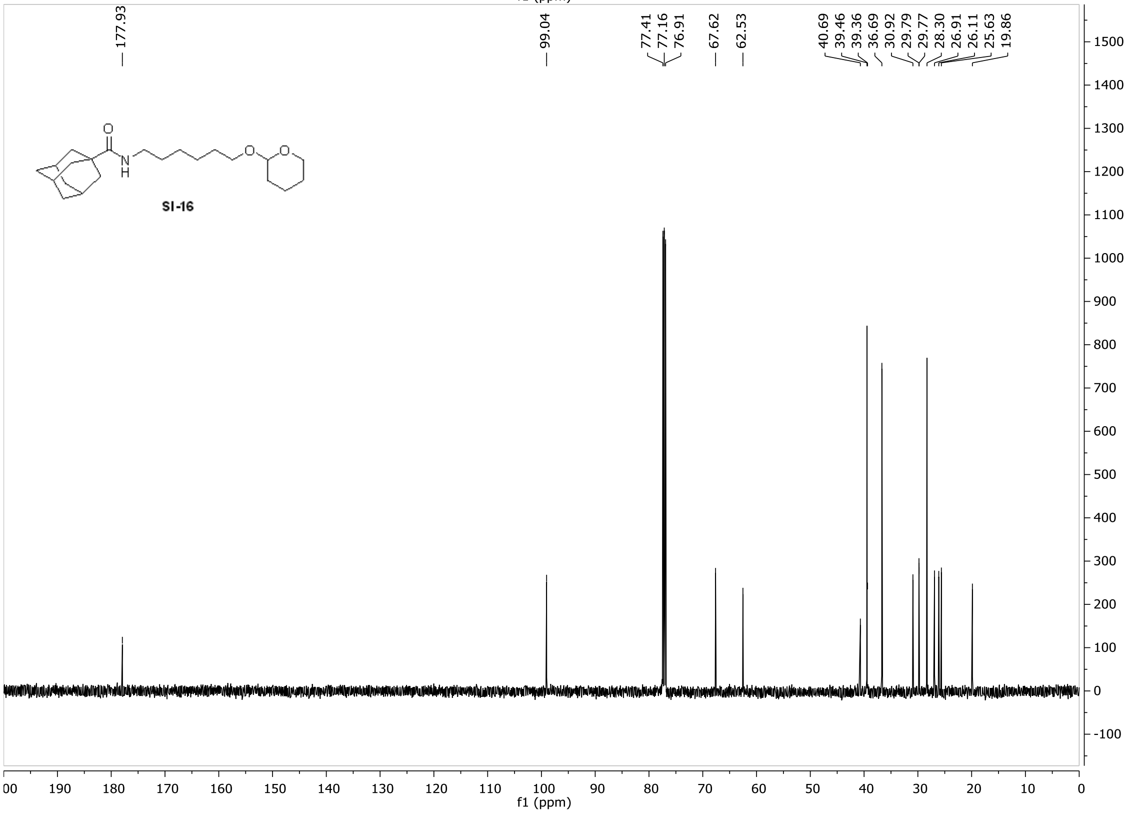
177.93 99.04 77.41 77.16 76.91 67.62 62.53 40.69 39.46 39.36 36.69 30.92 29.79 29.77 28.30 26.91 26.11 25.63 19.86
SI-16
f1 (ppm)

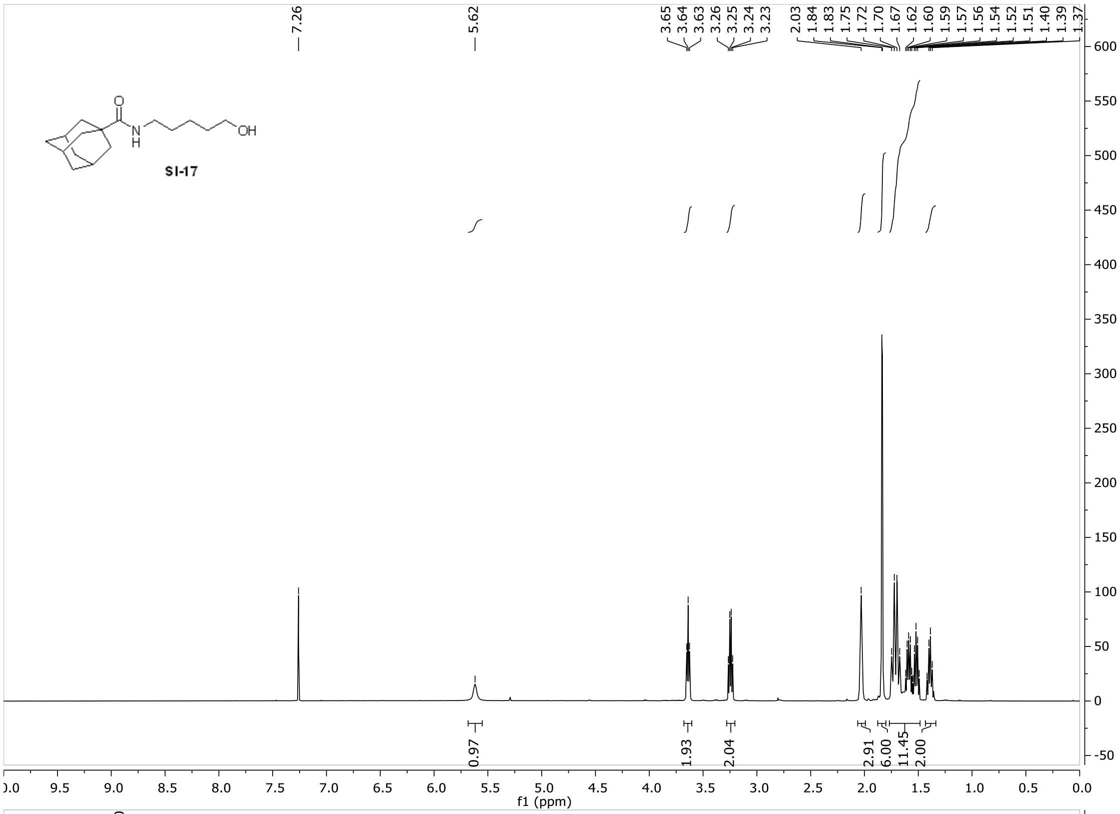
7.26
5.62
3.65
3.64
3.63
3.26
3.25
3.24
3.23
2.03
1.84
1.83
1.75
1.72
1.70
1.67
1.62
1.60
1.59
1.57
1.56
1.54
1.52
1.51
1.40
1.39
1.37
O
N
H
OH
SI-17
0.97
1.93
2.04
2.91
6.00
11.45
2.00
f1 (ppm)

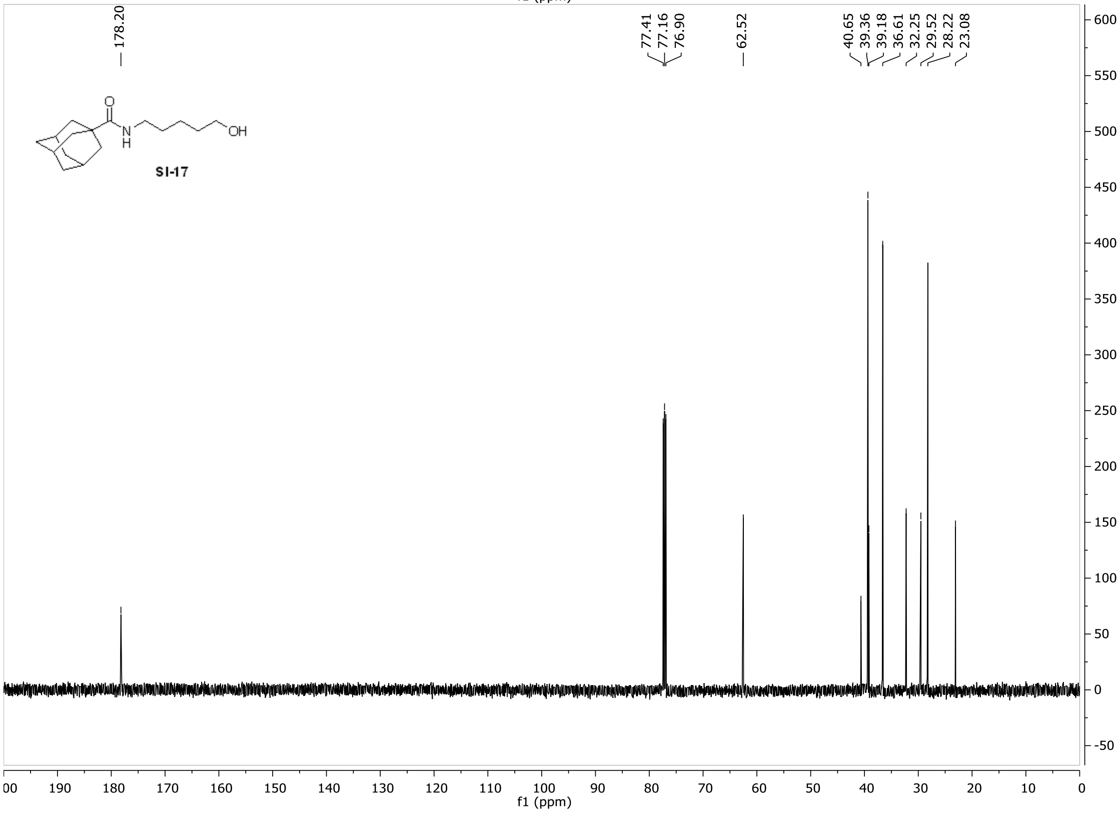
178.20
77.41
77.16
76.90
62.52
40.65
39.36
39.18
36.61
32.25
29.52
28.22
23.08
O
N
H
OH
SI-17
f1 (ppm)

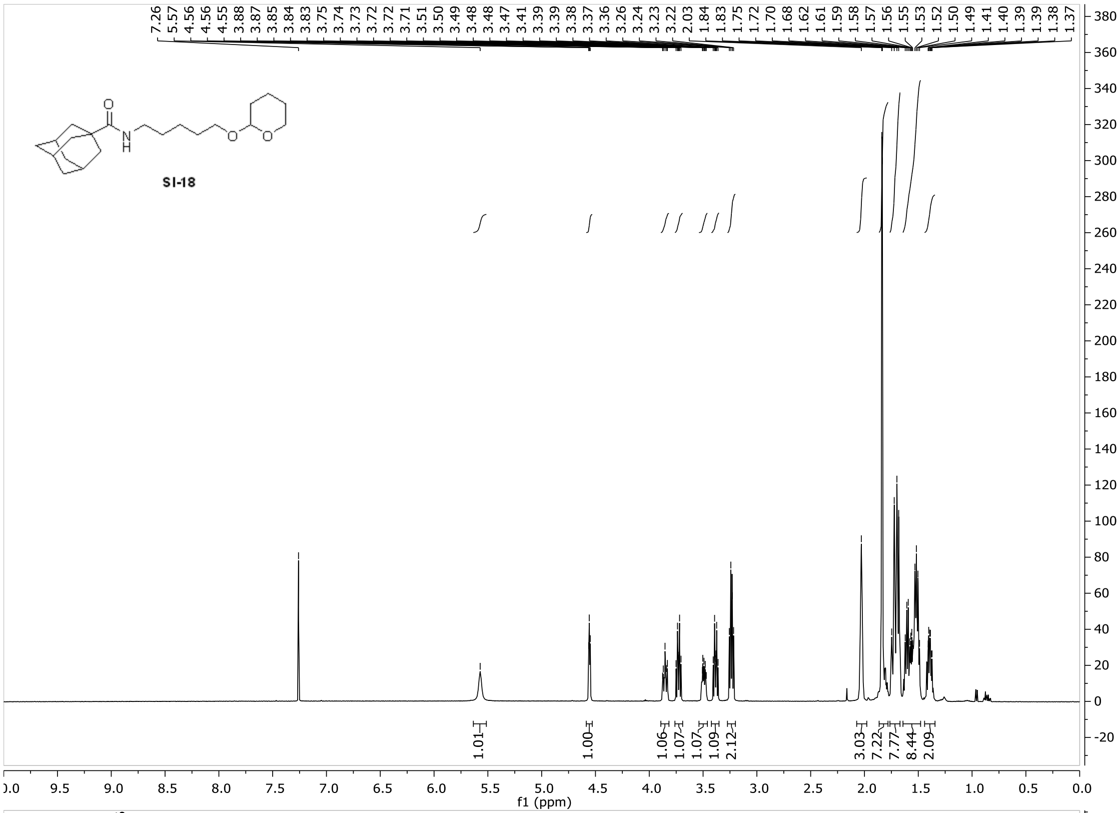
7.26 5.57 4.56 4.56 4.55 3.88 3.87 3.85 3.84 3.83 3.75 3.74 3.73 3.72 3.72 3.71 3.51 3.50 3.49 3.48 3.48 3.47 3.41 3.39 3.39 3.38 3.37 3.36 3.26 3.24 3.23 3.22 3.22 2.03 1.84 1.83 1.75 1.72 1.70 1.68 1.62 1.61 1.59 1.58 1.57 1.56 1.55 1.53 1.52 1.50 1.49 1.41 1.40 1.39 1.39 1.38 1.37
O
N
H
O
O
SI-18
1.01
1.00
1.06
1.07
1.07
1.09
2.12
3.03
7.22
7.77
8.44
2.09
f1 (ppm)
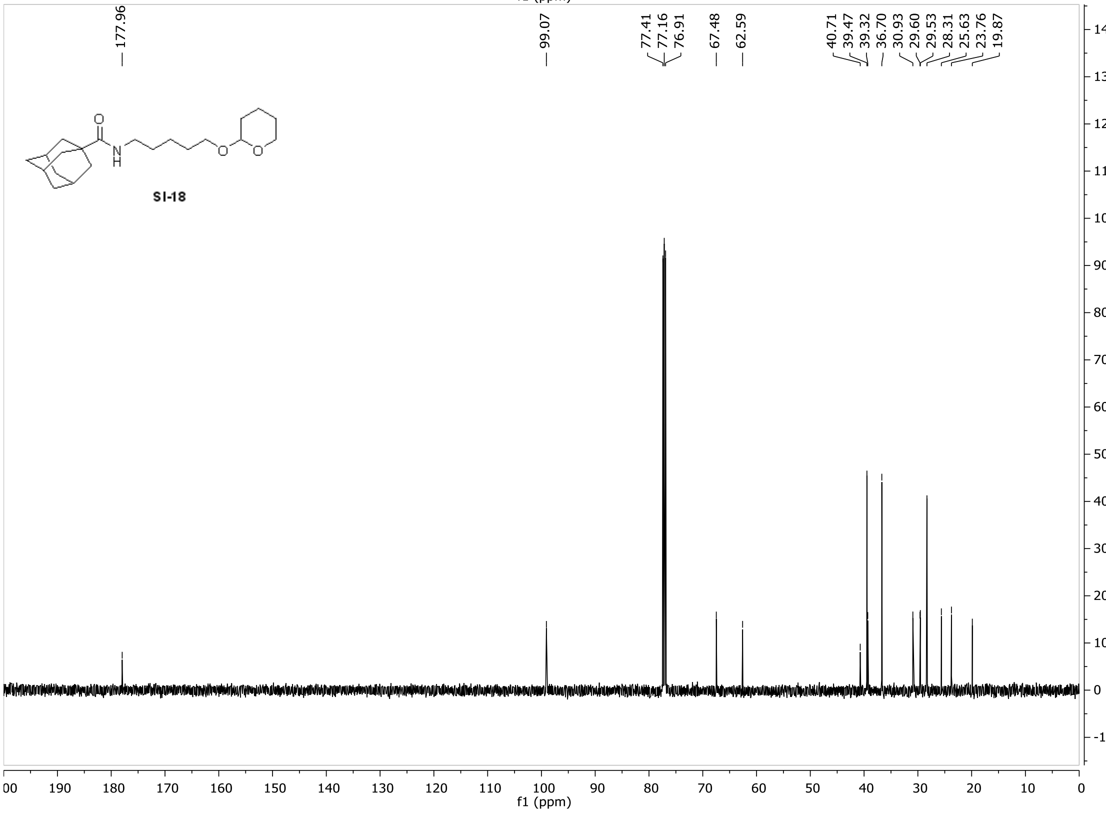
177.96
99.07
77.41 77.16 76.91
67.48
62.59
40.71 39.47 39.32 36.70 30.93 29.60 29.53 28.31 25.63 23.76 19.87
O
N
H
O
O
SI-18
f1 (ppm)

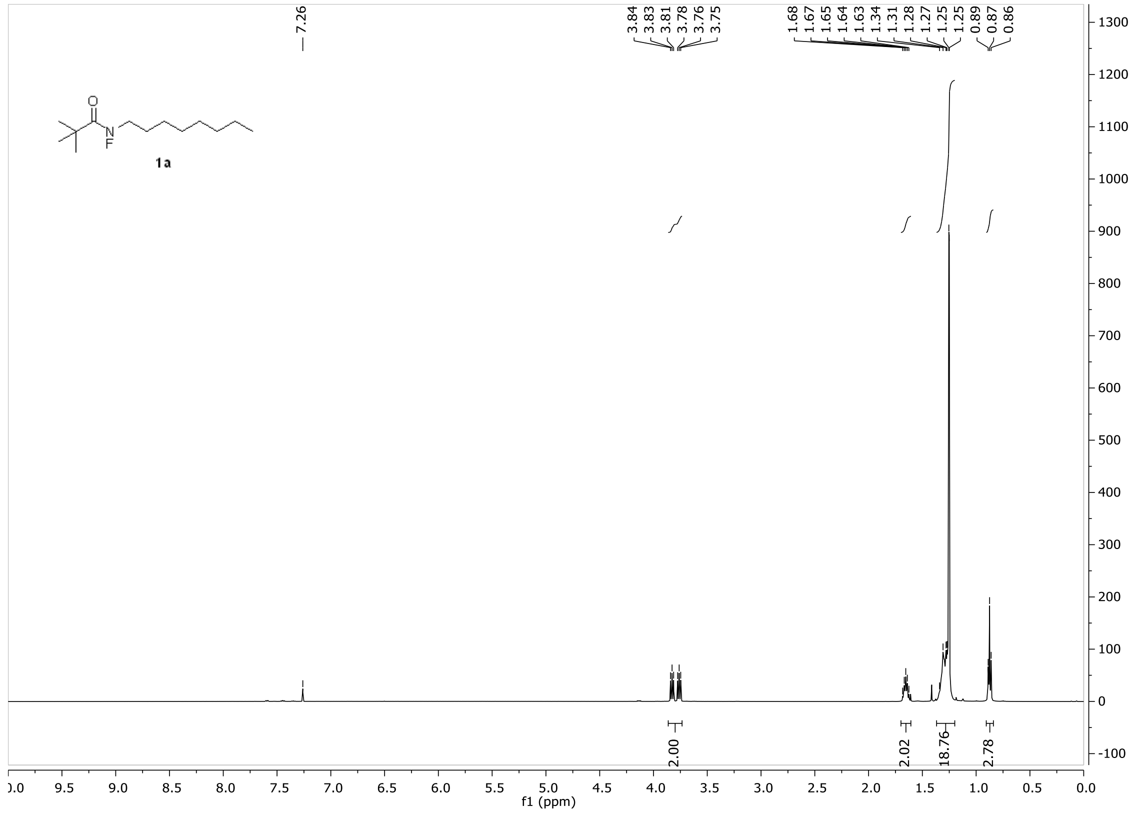
7.26
3.84
3.83
3.81
3.78
3.76
3.75
1.68
1.67
1.65
1.64
1.63
1.34
1.31
1.28
1.27
1.25
1.25
0.89
0.87
0.86
1a
2.00
2.02
18.76
2.78
f1 (ppm)

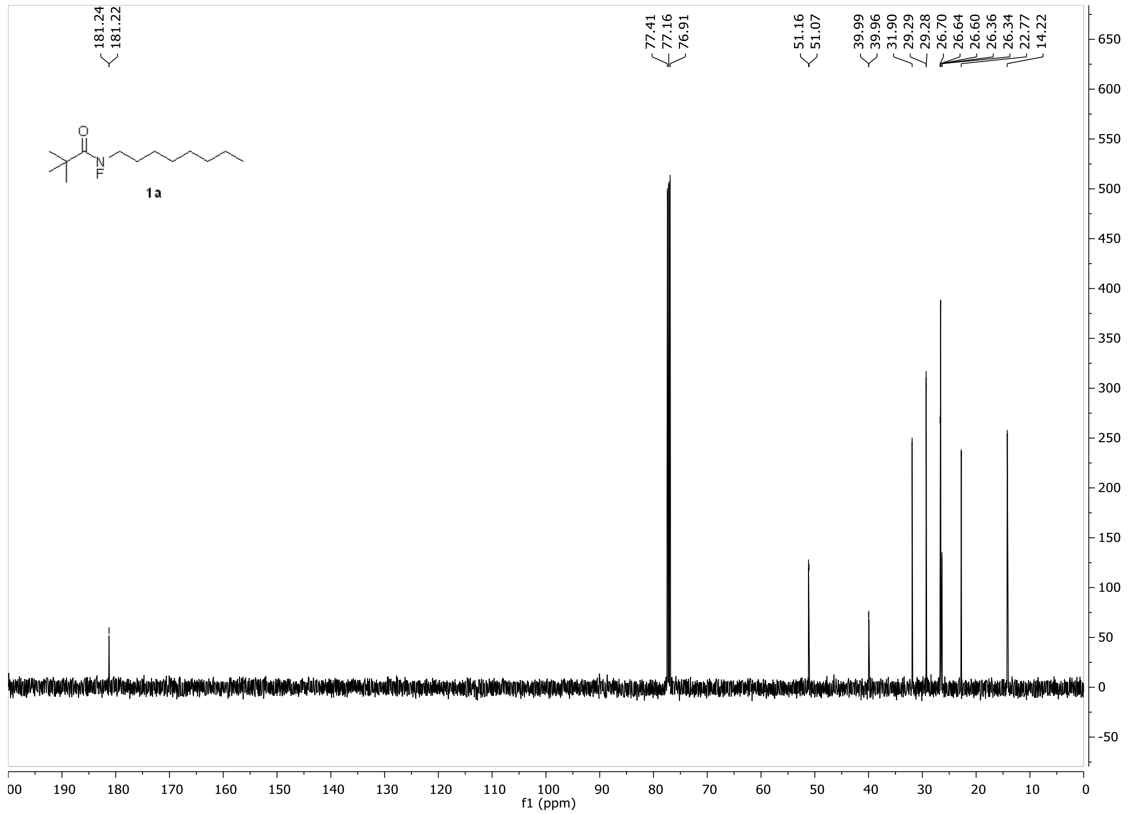
181.24
181.22
77.41
77.16
76.91
51.16
51.07
39.99
39.96
31.90
29.29
29.28
26.70
26.64
26.60
26.36
26.34
22.77
14.22
1a
f1 (ppm)

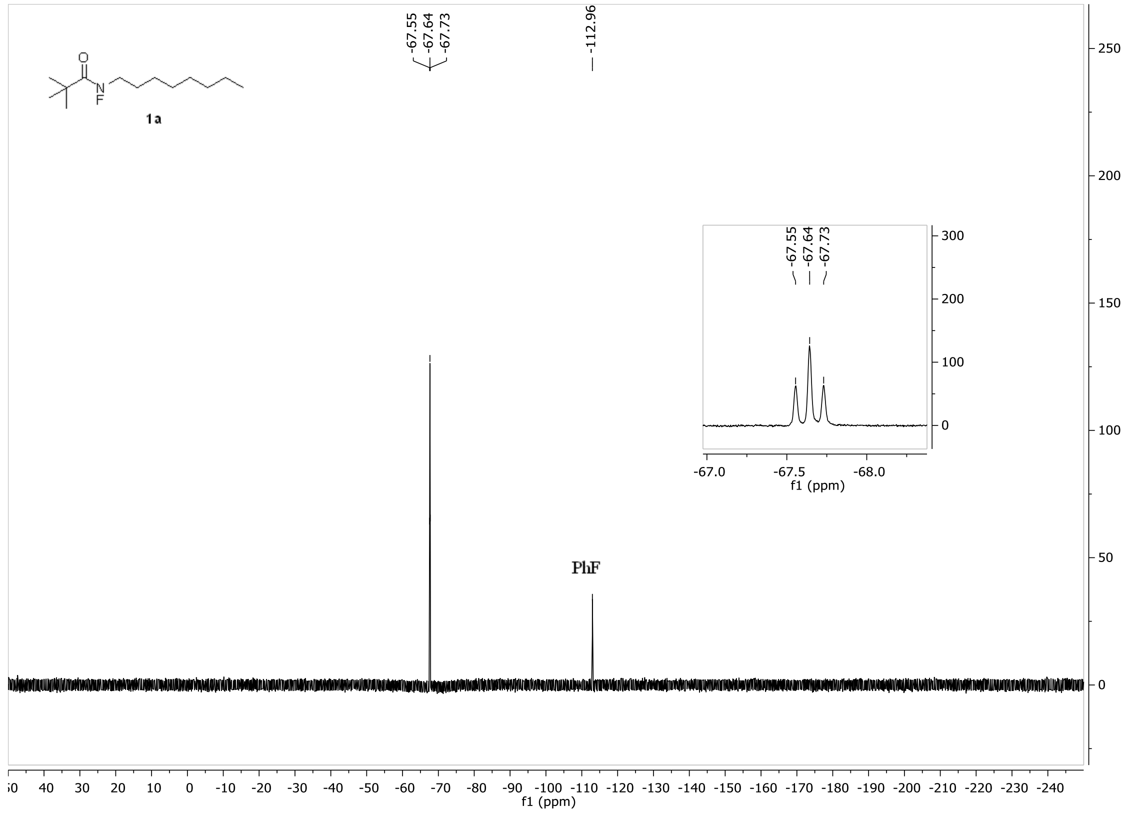
-67.55
-67.64
-67.73
-112.96
1a
PhF
f1 (ppm)

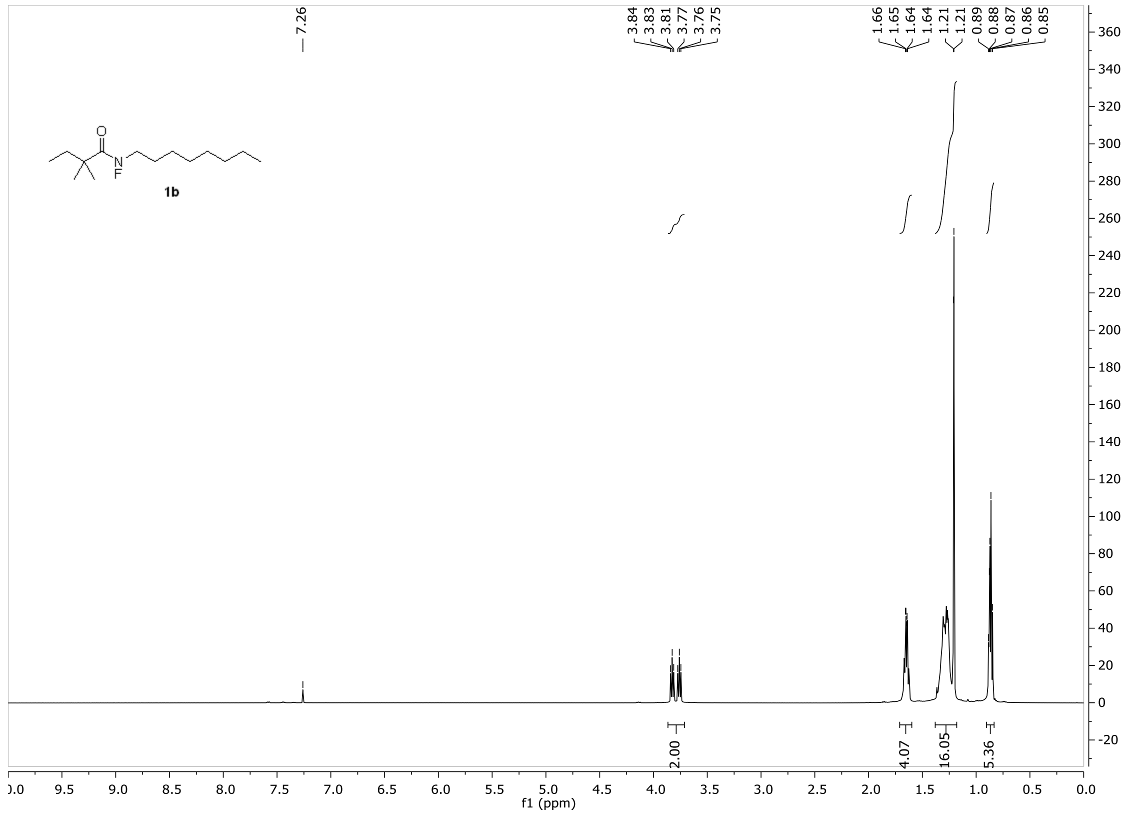
7.26
1b
2.00
4.07
16.05
5.36
f1 (ppm)

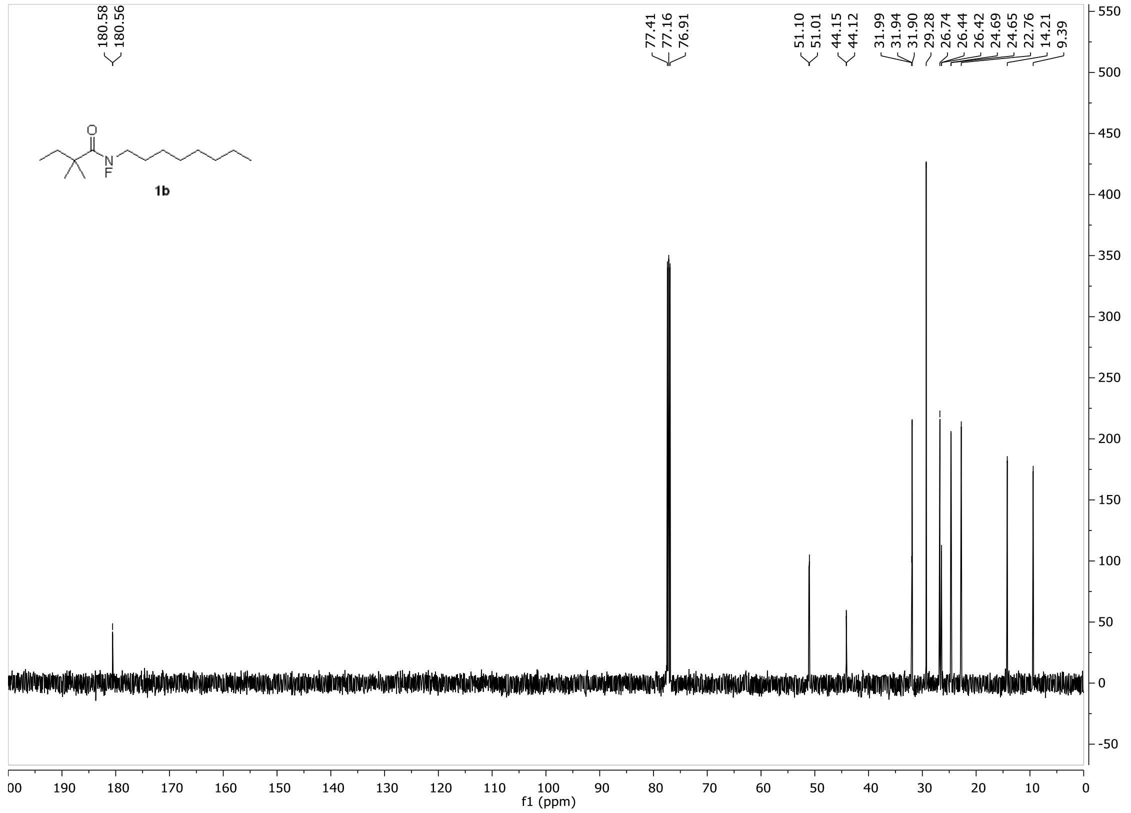
180.58
180.56
77.41
77.16
76.91
51.10
51.01
44.15
44.12
31.99
31.94
31.90
29.28
26.74
26.44
26.42
24.69
24.65
22.76
14.21
9.39
O
N
F
1b
f1 (ppm)

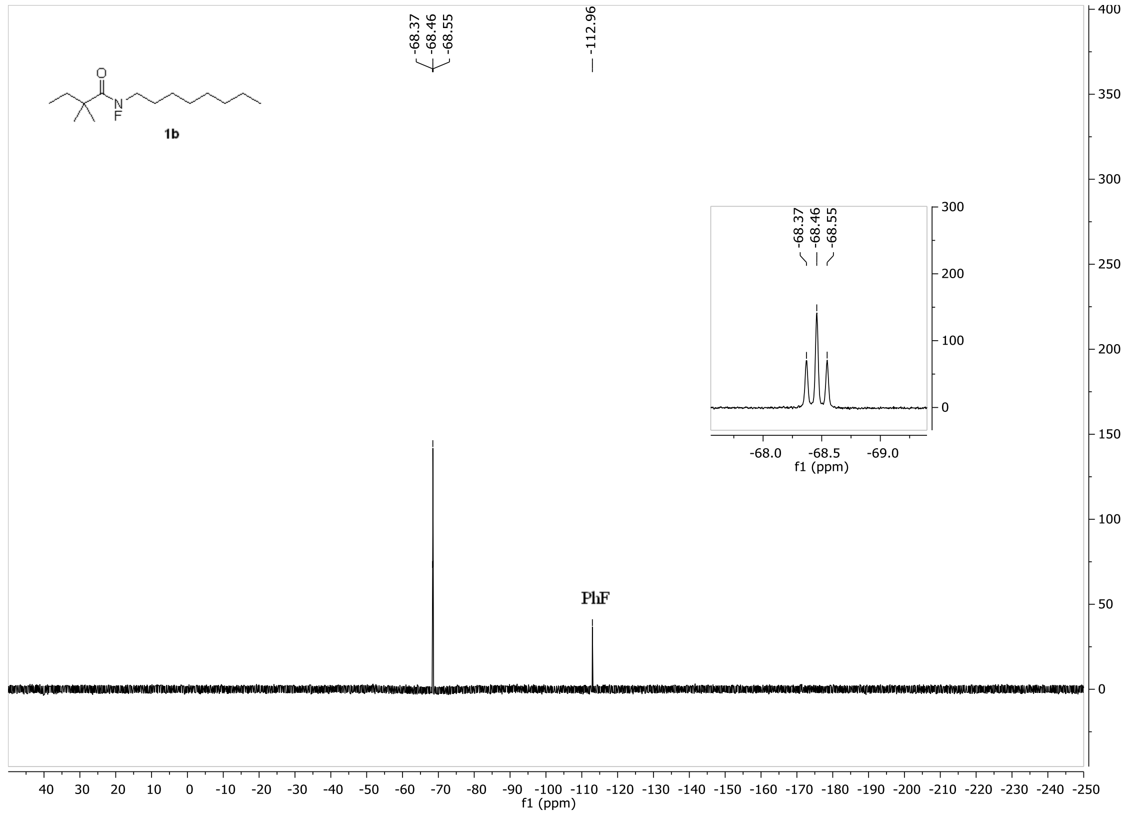
-68.37
-68.46
-68.55
-112.96
O
N
F
1b
PhF
-68.37
-68.46
-68.55
-68.0
-68.5
-69.0
f1 (ppm)
f1 (ppm)

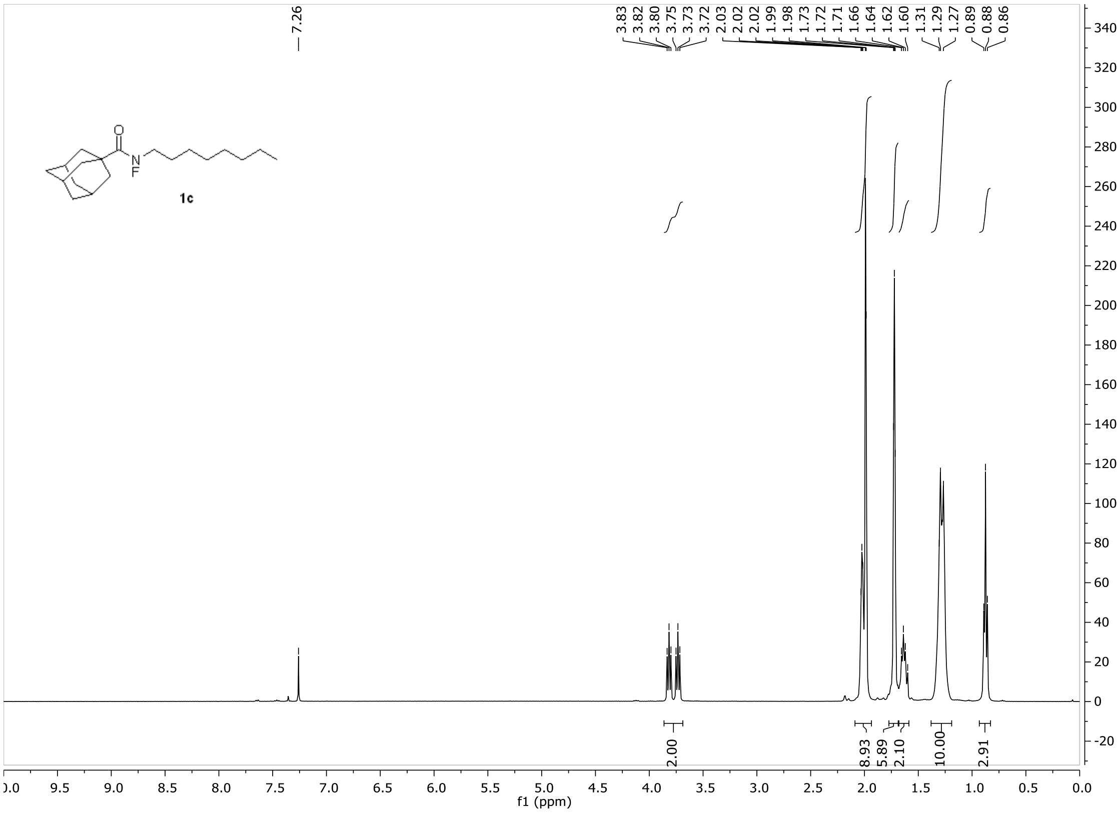

7.26
3.83
3.82
3.80
3.75
3.73
3.72
2.03
2.02
2.02
1.99
1.98
1.73
1.72
1.71
1.66
1.64
1.62
1.60
1.31
1.29
1.27
0.89
0.88
0.86
1c
2.00
8.93
5.89
2.10
10.00
2.91
f1 (ppm)

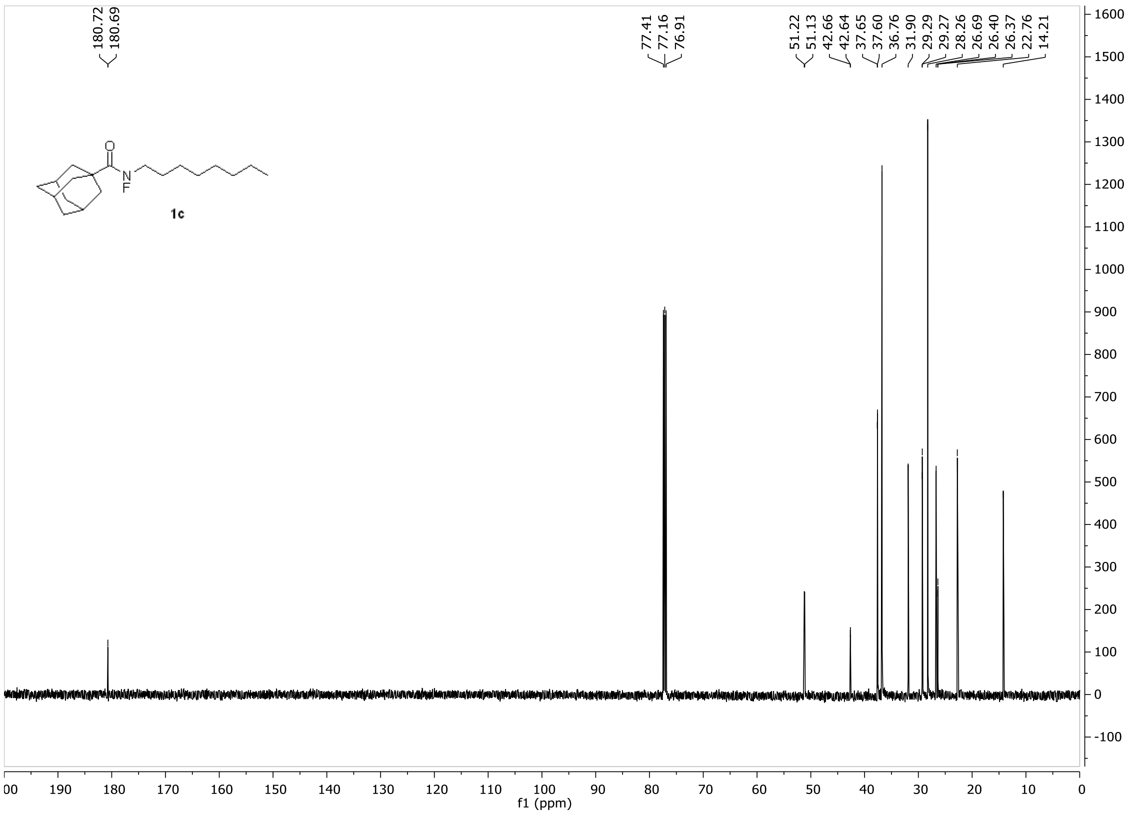

180.72
180.69
77.41
77.16
76.91
51.22
51.13
42.66
42.64
37.65
37.60
36.76
31.90
29.29
29.27
28.26
26.69
26.40
26.37
22.76
14.21
1c
f1 (ppm)

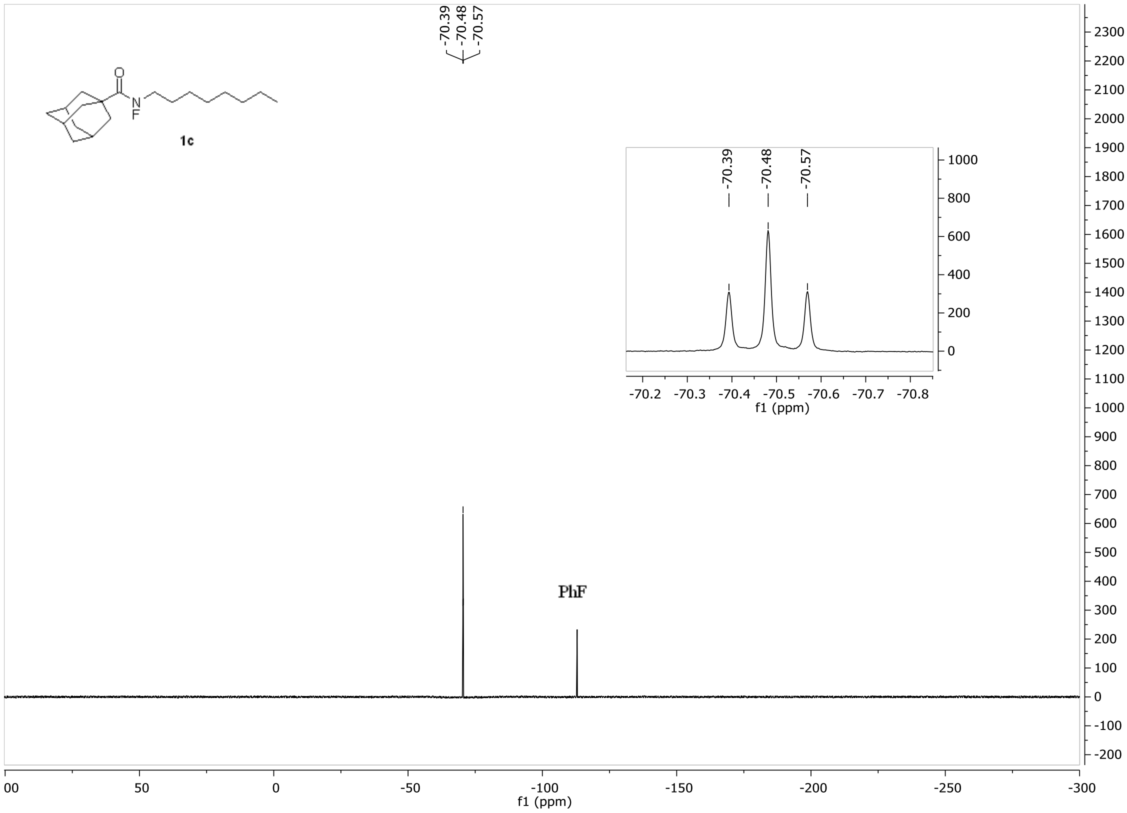

-70.39
-70.48
-70.57
1c
PhF
f1 (ppm)

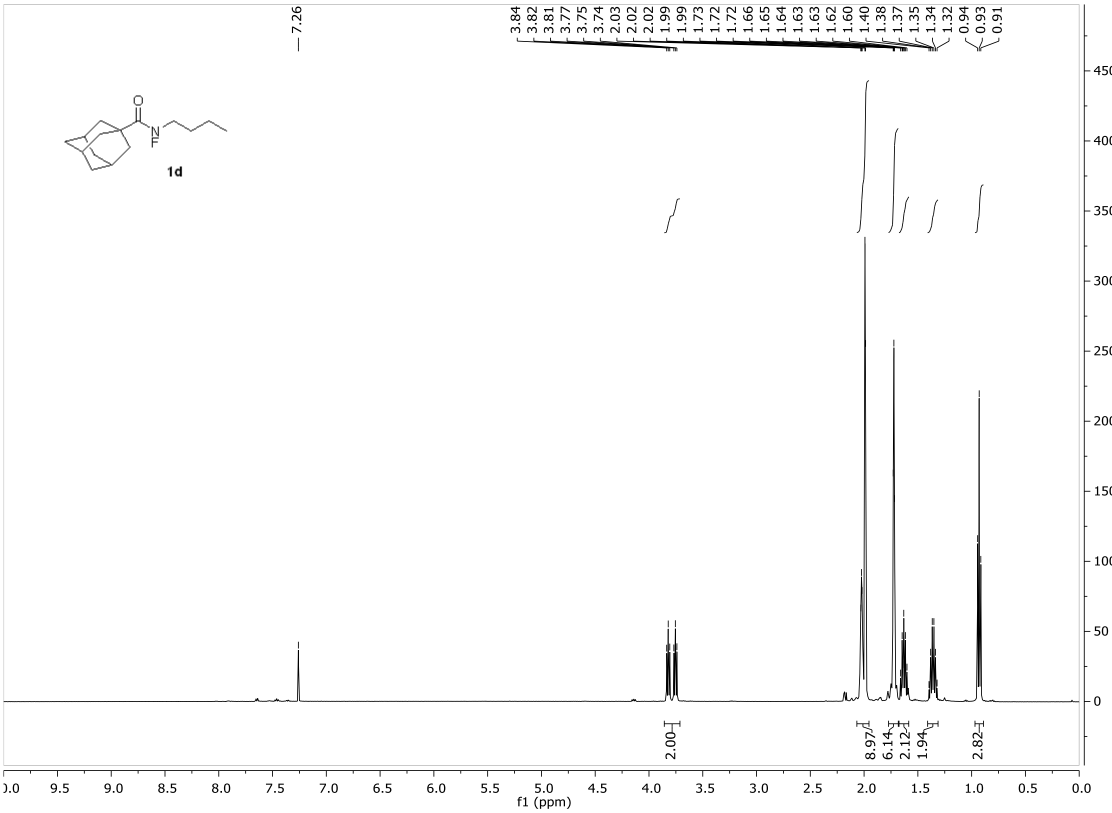

7.26
3.84
3.82
3.81
3.77
3.75
3.74
2.03
2.02
2.02
1.99
1.99
1.73
1.72
1.72
1.66
1.65
1.64
1.63
1.63
1.62
1.60
1.40
1.38
1.37
1.35
1.34
1.32
0.94
0.93
0.91
1d
2.00
8.97
6.14
2.12
1.94
2.82
f1 (ppm)

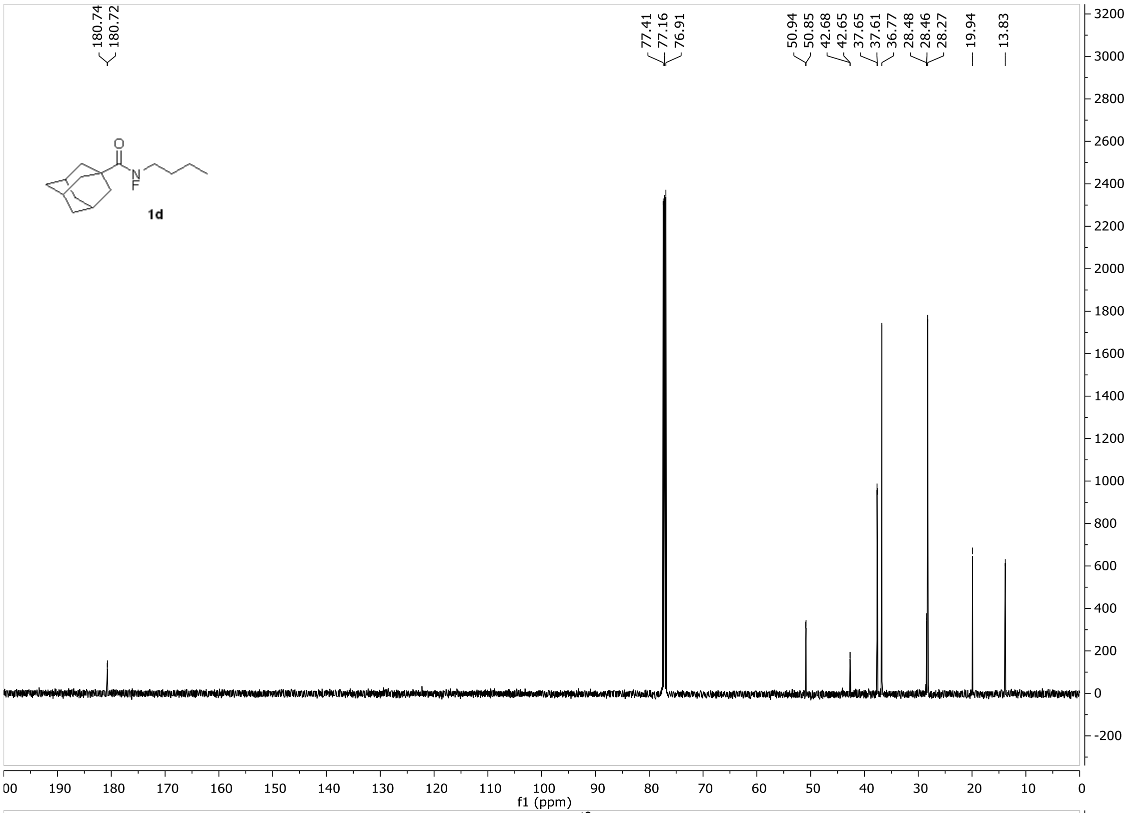
180.74
180.72
77.41
77.16
76.91
50.94
50.85
42.68
42.65
37.65
37.61
36.77
28.48
28.46
28.27
19.94
13.83
O
N
F
1d
f1 (ppm)

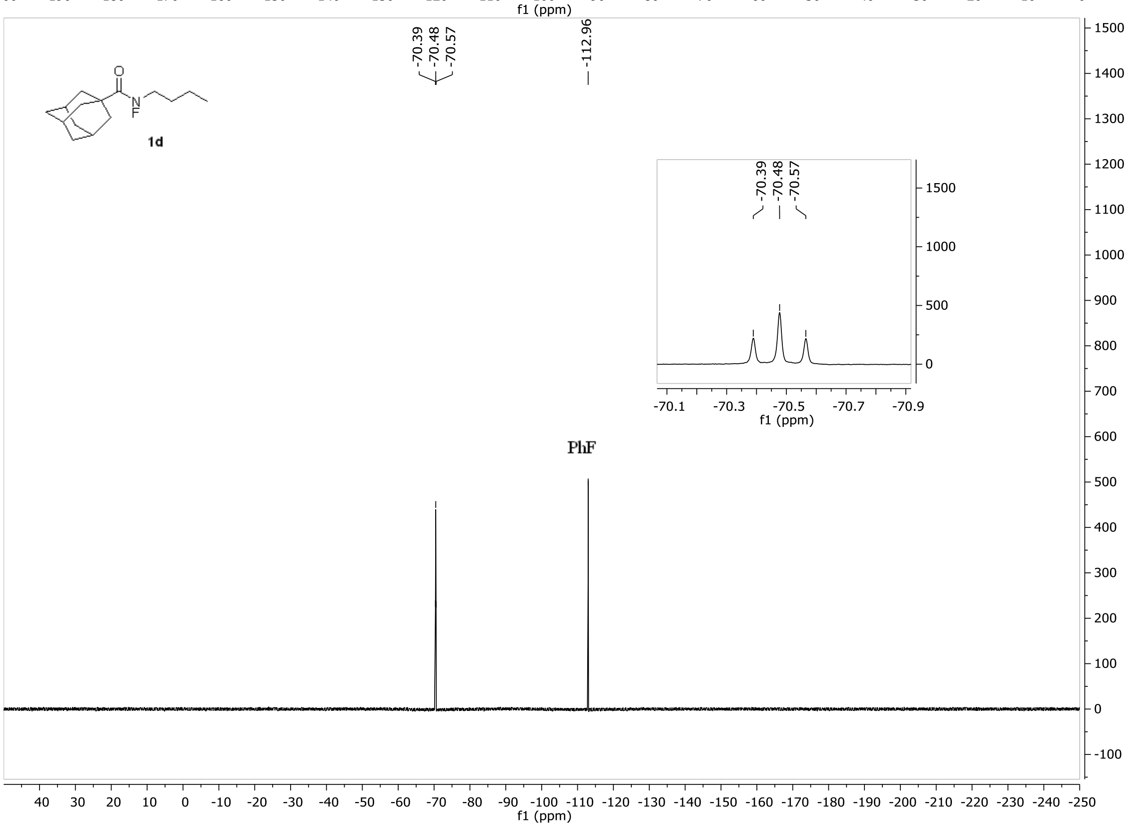
-70.39
-70.48
-70.57
-112.96
O
N
F
1d
PhF
f1 (ppm)

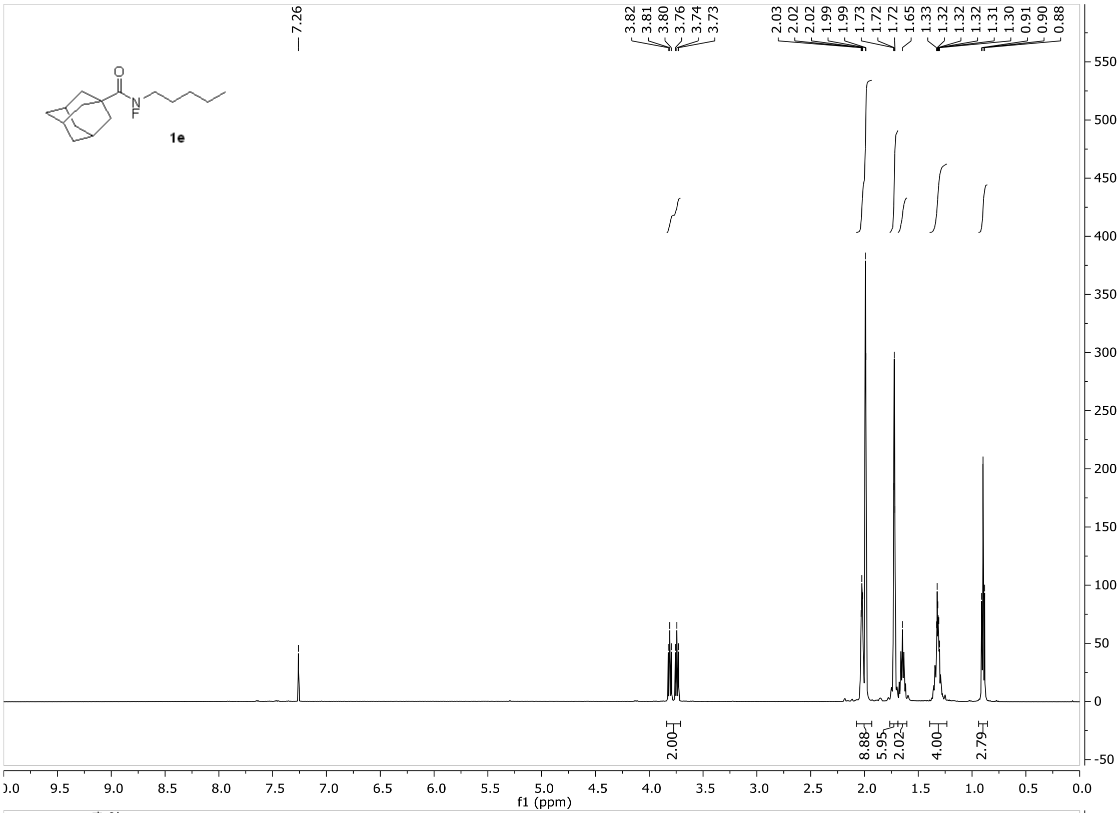
7.26
3.82
3.81
3.80
3.76
3.74
3.73
2.03
2.02
2.02
1.99
1.99
1.73
1.72
1.72
1.65
1.33
1.32
1.32
1.32
1.31
1.30
0.91
0.90
0.88
1e
2.00
8.88
5.95
2.02
4.00
2.79
f1 (ppm)

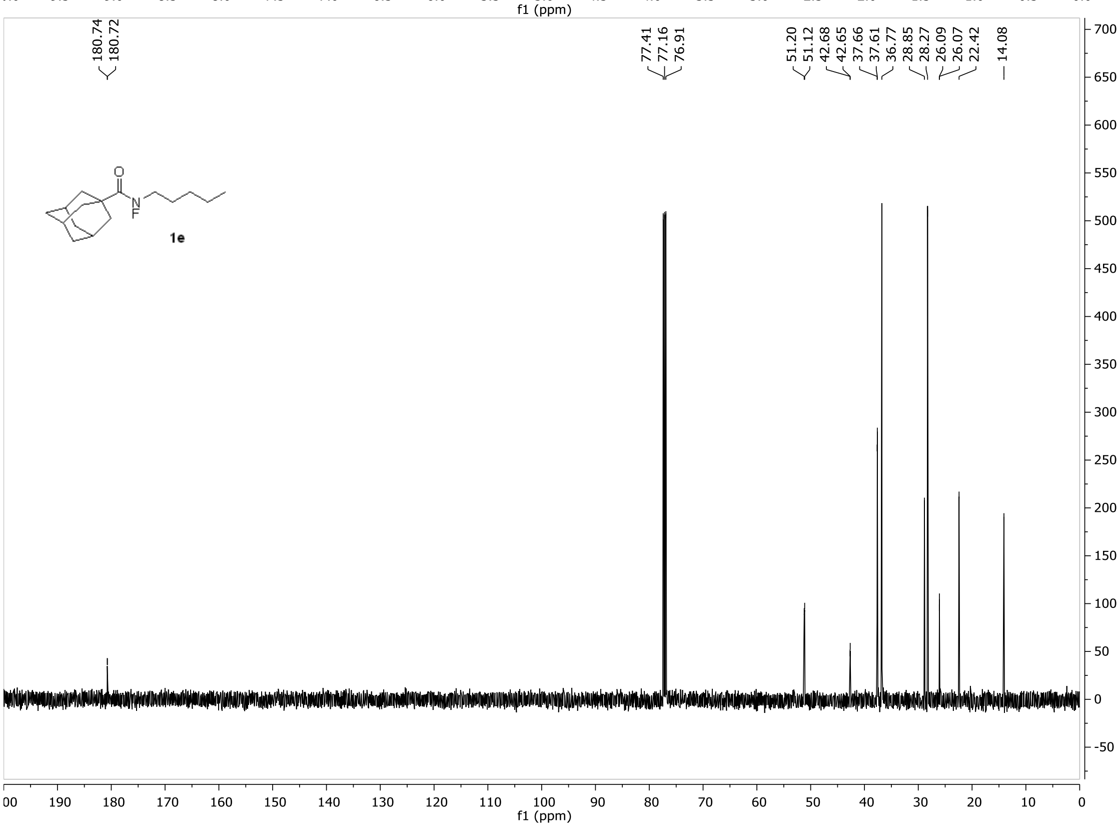
180.74
180.72
77.41
77.16
76.91
51.20
51.12
42.68
42.65
37.66
37.61
36.77
28.85
28.27
26.09
26.07
22.42
14.08
1e
f1 (ppm)

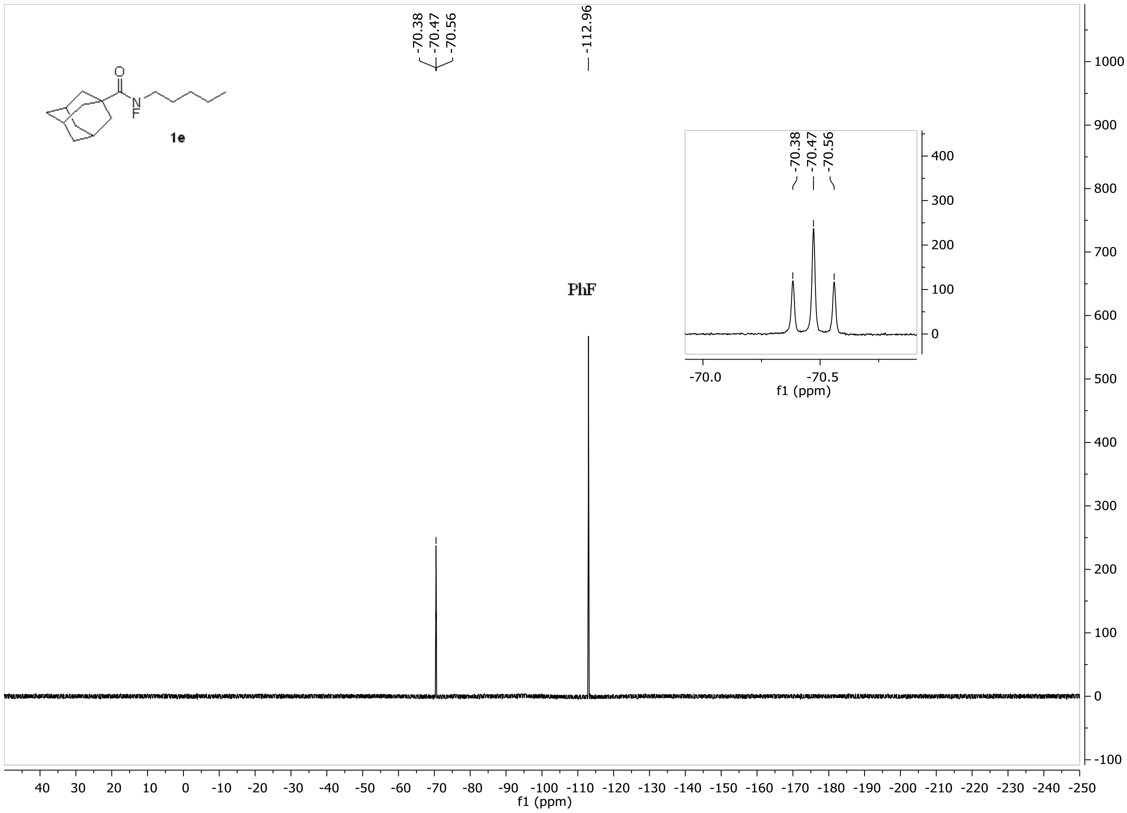
1e
PhF
-70.38
-70.47
-70.56
-112.96
f1 (ppm)

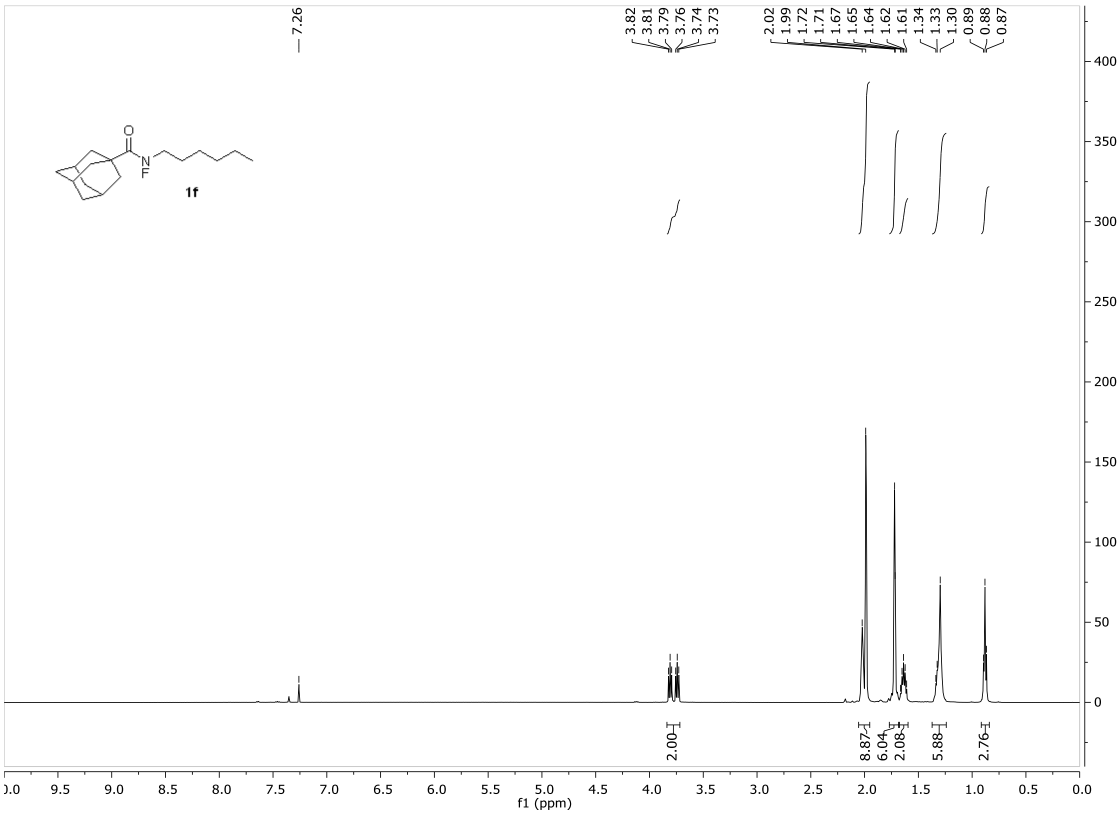
1f
7.26
3.82
3.81
3.79
3.76
3.74
3.73
2.02
1.99
1.72
1.71
1.67
1.65
1.64
1.62
1.61
1.34
1.33
1.30
0.89
0.88
0.87
2.00
8.87
6.04
2.08
5.88
2.76
f1 (ppm)

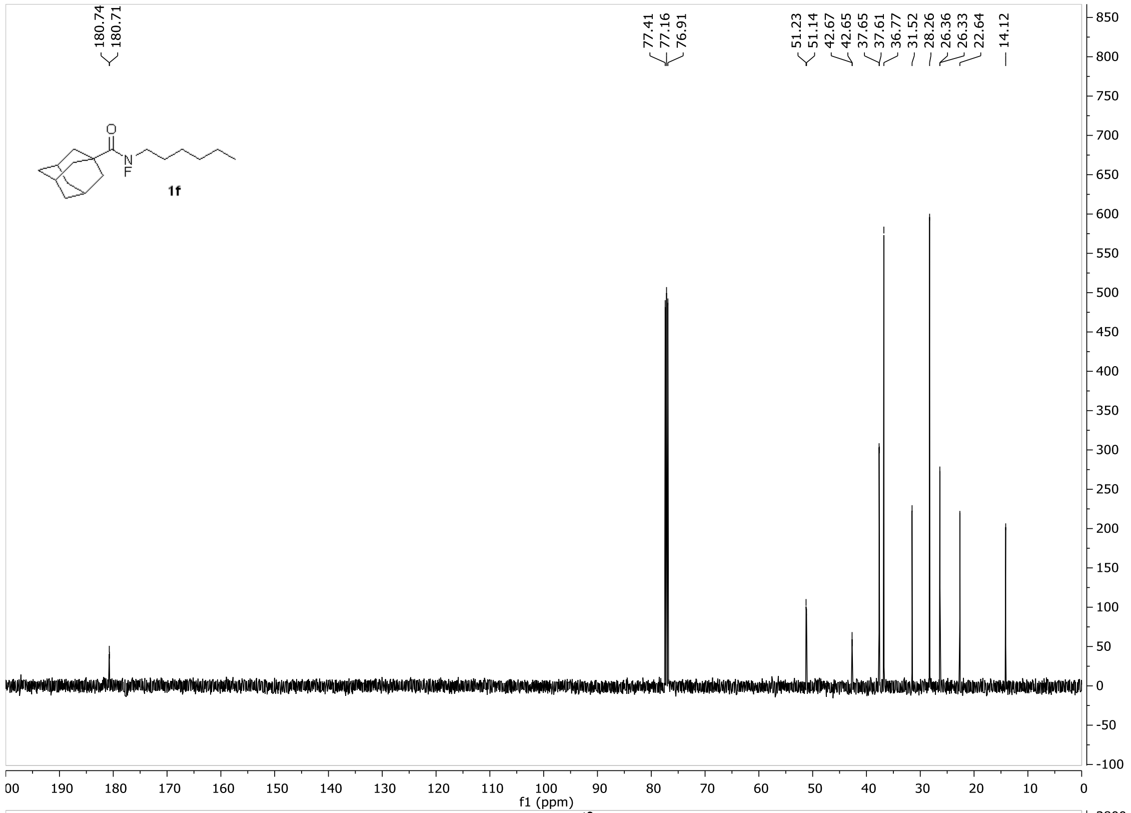
180.74
180.71
77.41
77.16
76.91
51.23
51.14
42.67
42.65
37.65
37.61
36.77
31.52
28.26
26.36
26.33
22.64
14.12
1f
f1 (ppm)

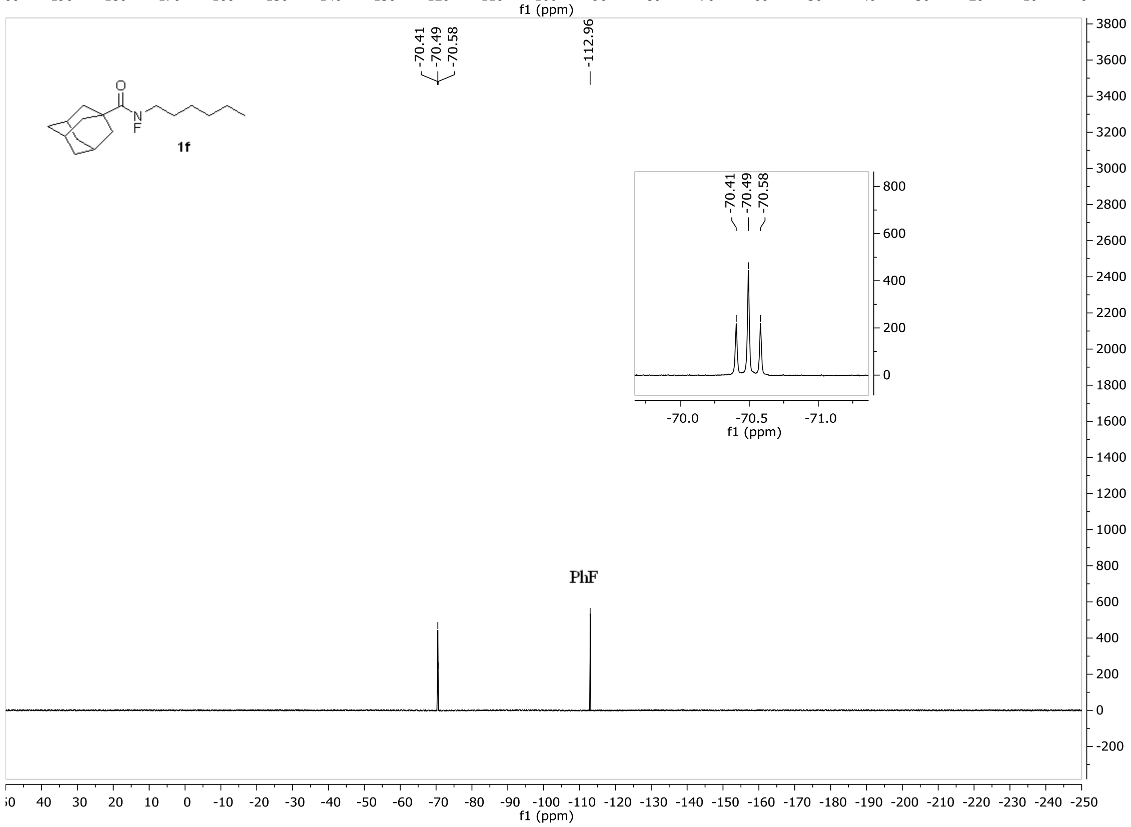
-70.41
-70.49
-70.58
-112.96
1f
PhF
f1 (ppm)

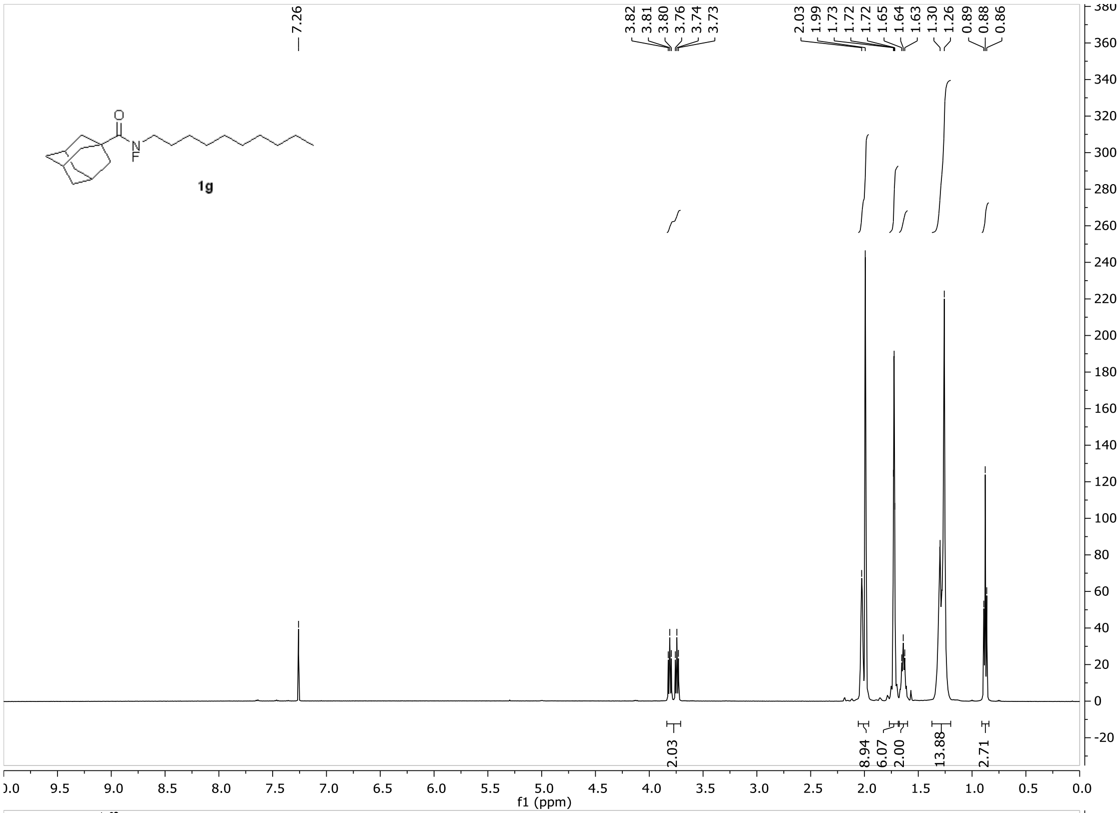
7.26
3.82
3.81
3.80
3.76
3.74
3.73
2.03
1.99
1.73
1.72
1.72
1.65
1.64
1.63
1.30
1.26
0.89
0.88
0.86
1g
2.03
8.94
6.07
2.00
13.88
2.71
f1 (ppm)

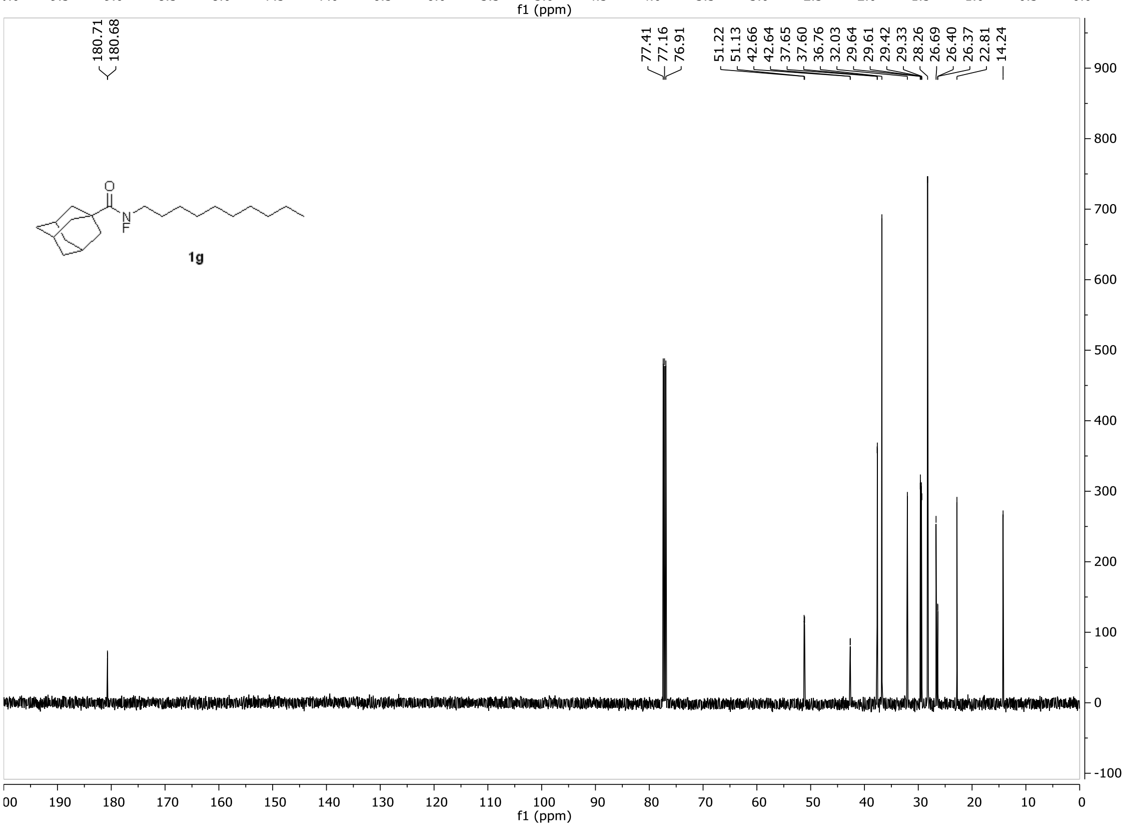
180.71
180.68
77.41
77.16
76.91
51.22
51.13
42.66
42.64
37.65
37.60
36.76
32.03
29.64
29.61
29.42
29.33
28.26
26.69
26.40
26.37
22.81
14.24
1g
f1 (ppm)

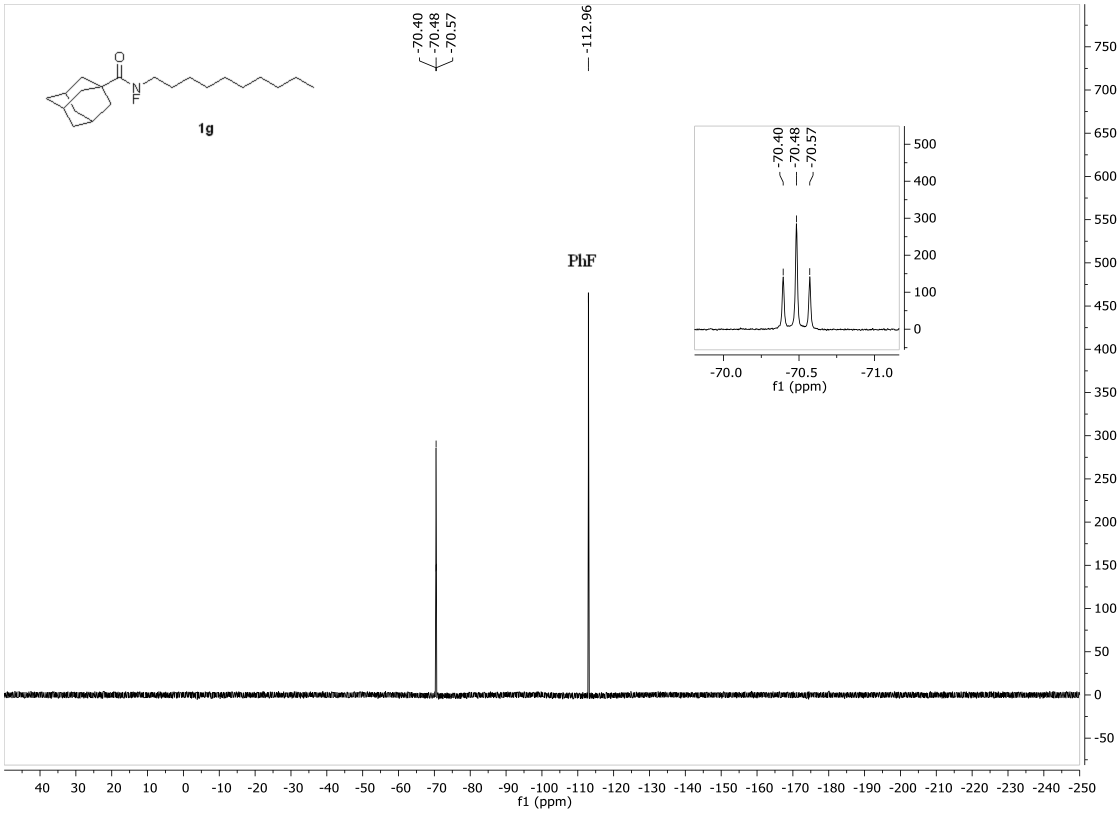
1g
-70.40
-70.48
-70.57
-112.96
PhF
f1 (ppm)

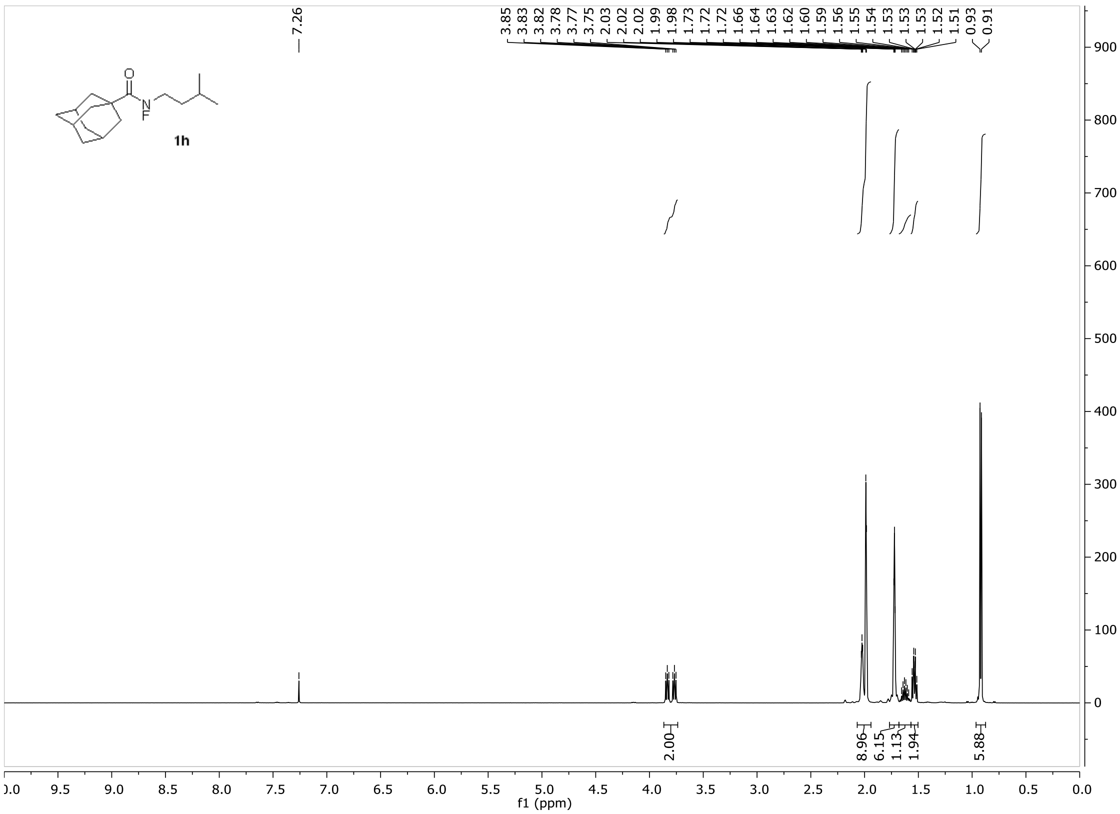
1h
7.26
3.85
3.83
3.82
3.78
3.77
3.75
2.03
2.02
2.02
1.99
1.98
1.73
1.72
1.72
1.66
1.64
1.63
1.62
1.60
1.59
1.56
1.55
1.54
1.53
1.53
1.53
1.52
1.51
0.93
0.91
2.00
8.96
6.15
1.13
1.94
5.88
f1 (ppm)

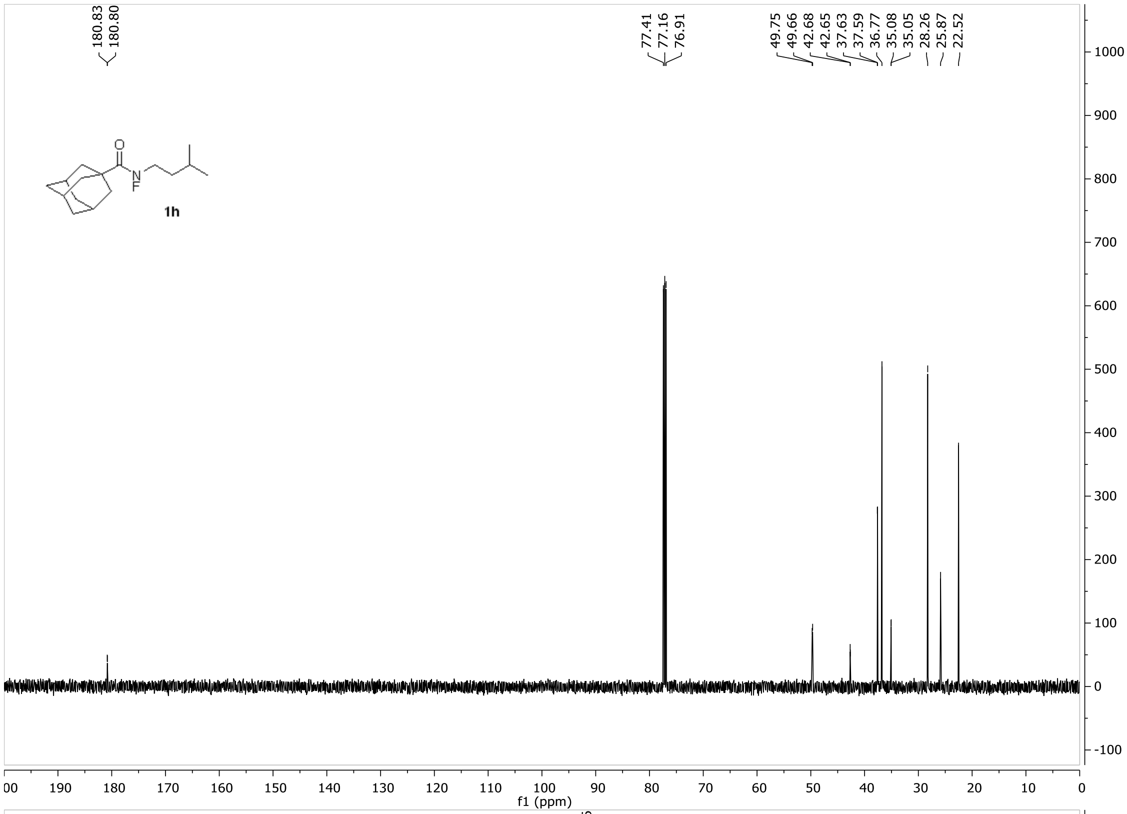
180.83
180.80
77.41
77.16
76.91
49.75
49.66
42.68
42.65
37.63
37.59
36.77
35.08
35.05
28.26
25.87
22.52
O
N
F
1h
f1 (ppm)

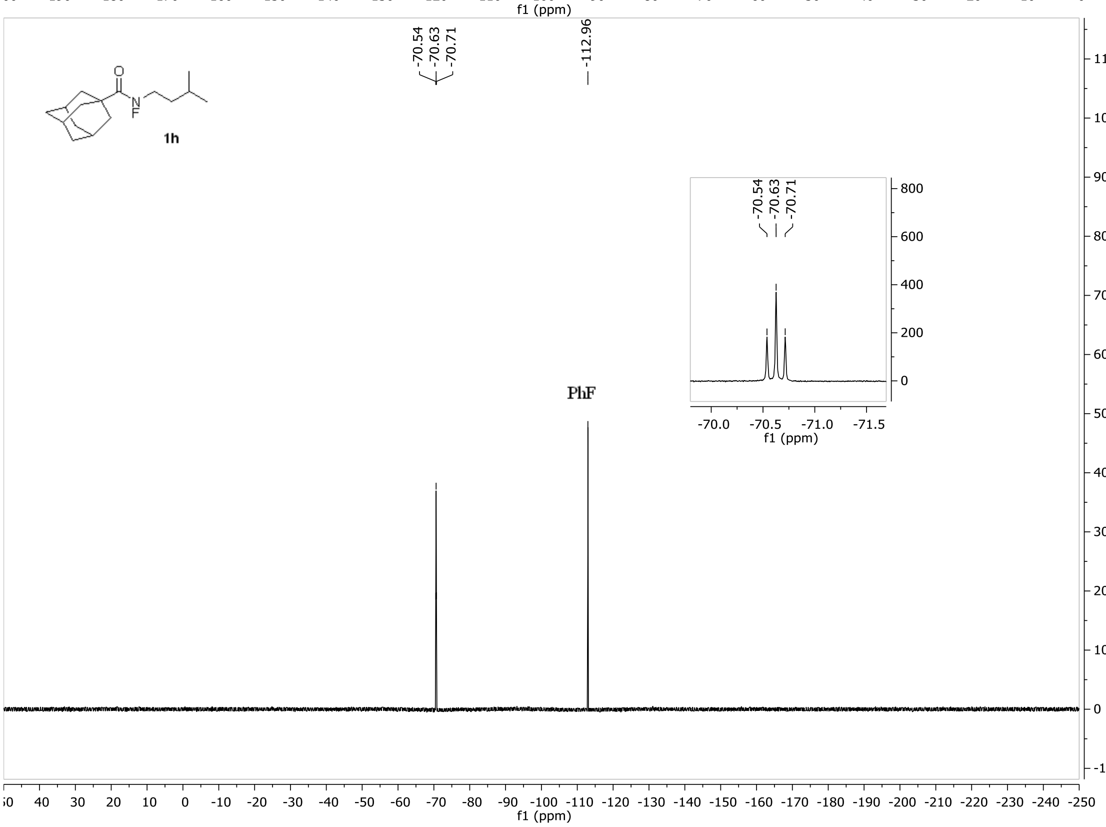
-70.54
-70.63
-70.71
-112.96
O
N
F
1h
PhF
f1 (ppm)

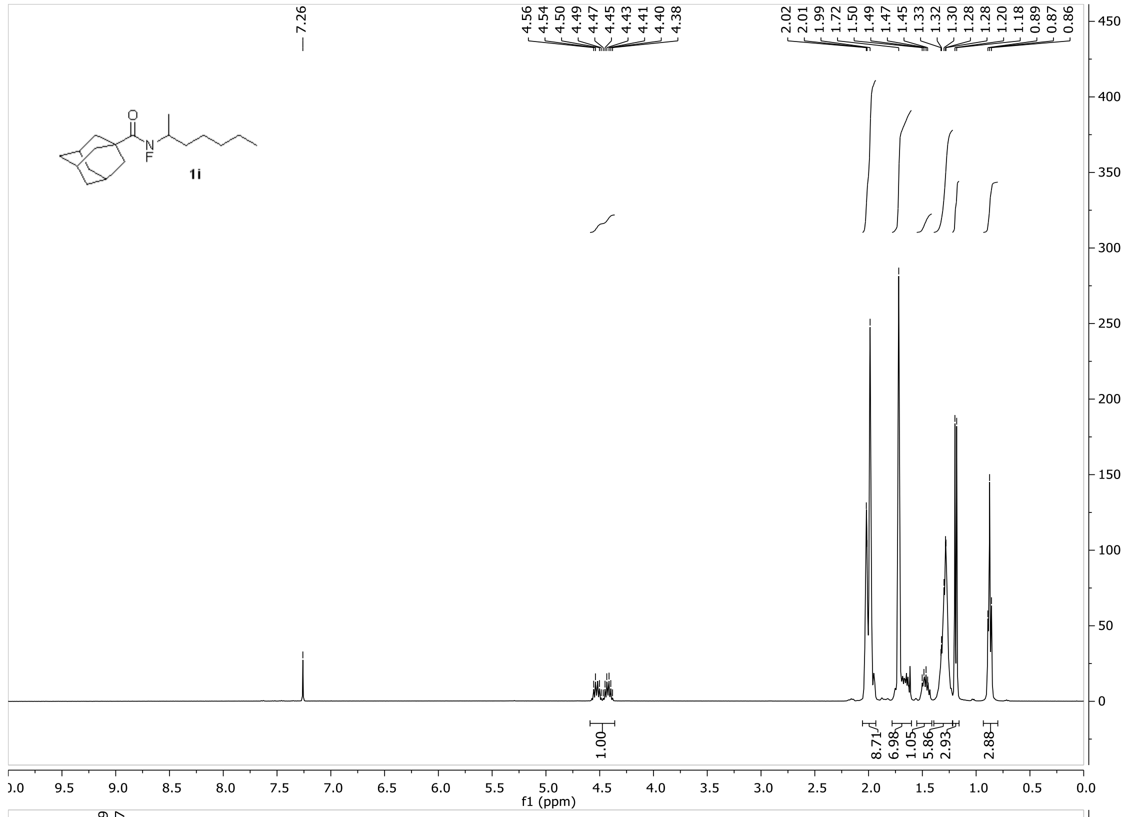
7.26
4.56
4.54
4.50
4.49
4.47
4.45
4.43
4.41
4.40
4.38
2.02
2.01
1.99
1.72
1.50
1.49
1.47
1.45
1.33
1.32
1.30
1.28
1.28
1.20
1.18
0.89
0.87
0.86
1i
1.00
8.71
6.98
1.05
5.86
2.93
2.88
f1 (ppm)

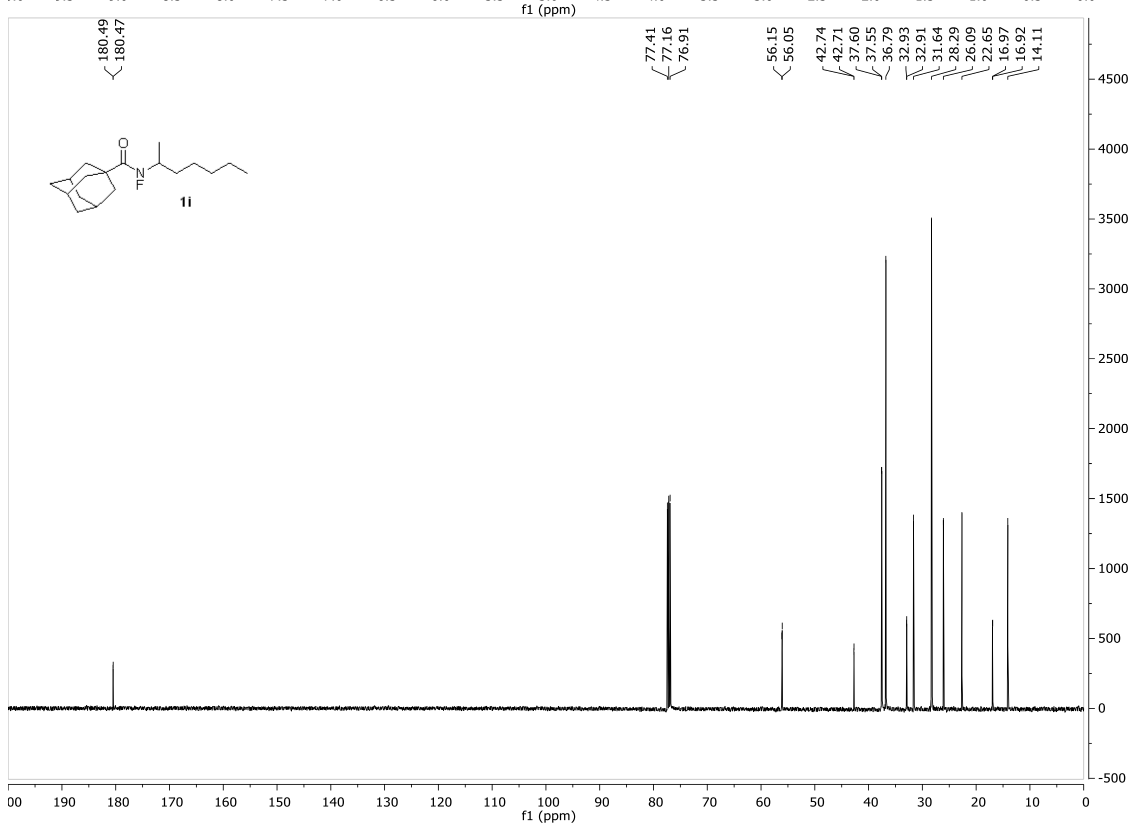
180.49
180.47
77.41
77.16
76.91
56.15
56.05
42.74
42.71
37.60
37.55
36.79
32.93
32.91
31.64
28.29
26.09
22.65
16.97
16.92
14.11
1i
f1 (ppm)

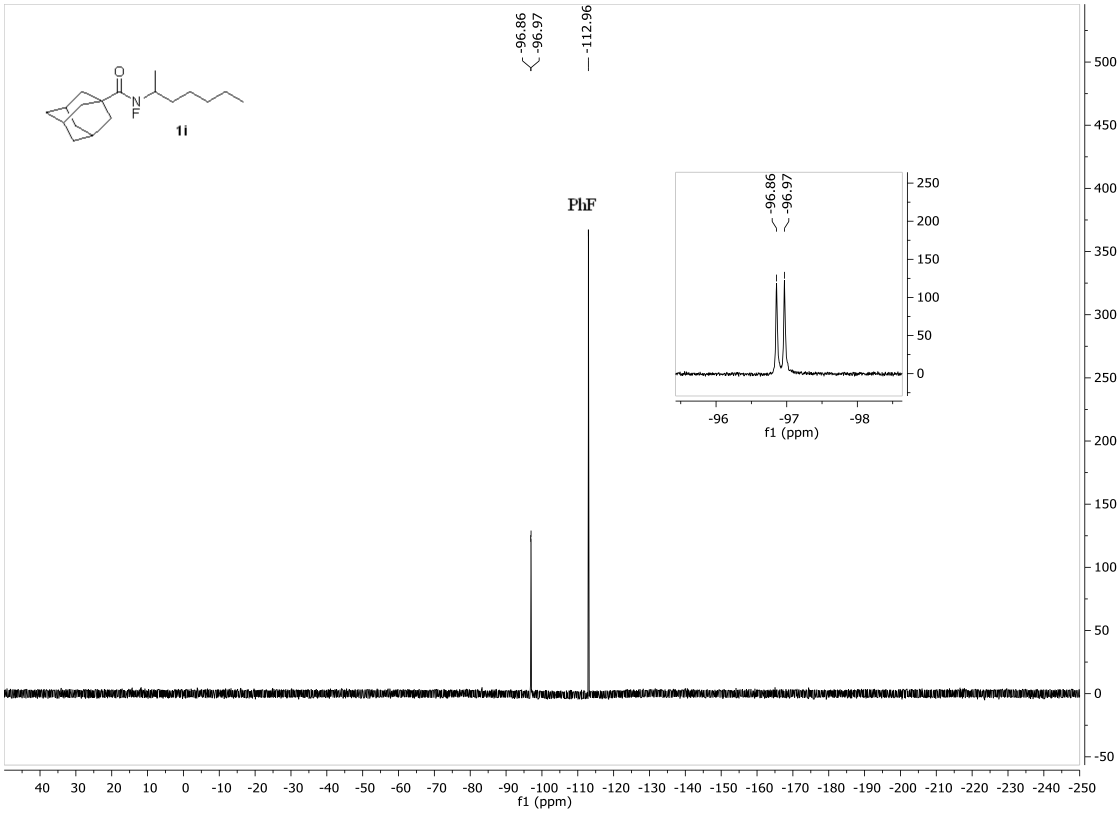
-96.86
-96.97
-112.96
1i
PhF
f1 (ppm)

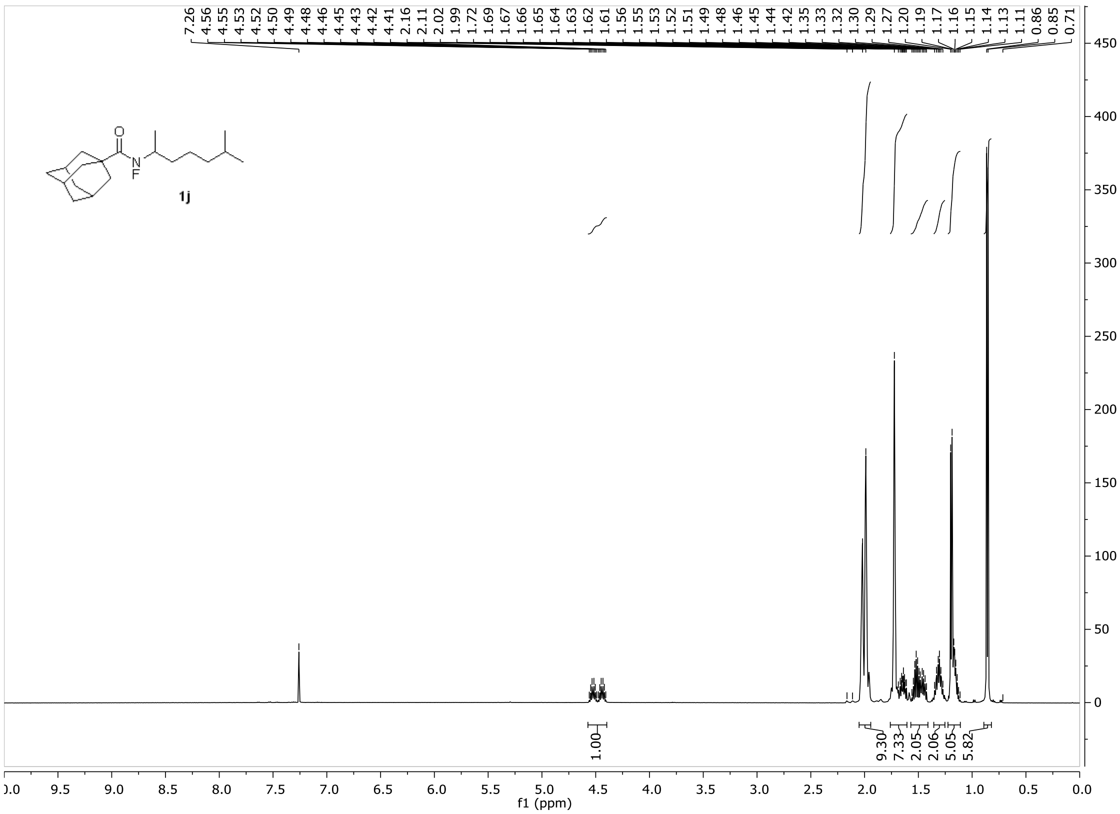
7.26 4.56 4.55 4.53 4.52 4.50 4.49 4.48 4.46 4.45 4.43 4.42 4.41 2.16 2.11 2.02 1.99 1.72 1.69 1.67 1.66 1.65 1.64 1.63 1.62 1.61 1.56 1.55 1.52 1.51 1.49 1.48 1.46 1.45 1.44 1.42 1.35 1.33 1.32 1.30 1.29 1.27 1.20 1.19 1.17 1.16 1.15 1.14 1.13 1.11 0.86 0.85 0.71
1j
1.00
9.30
7.33
2.05
2.06
5.05
5.82
f1 (ppm)

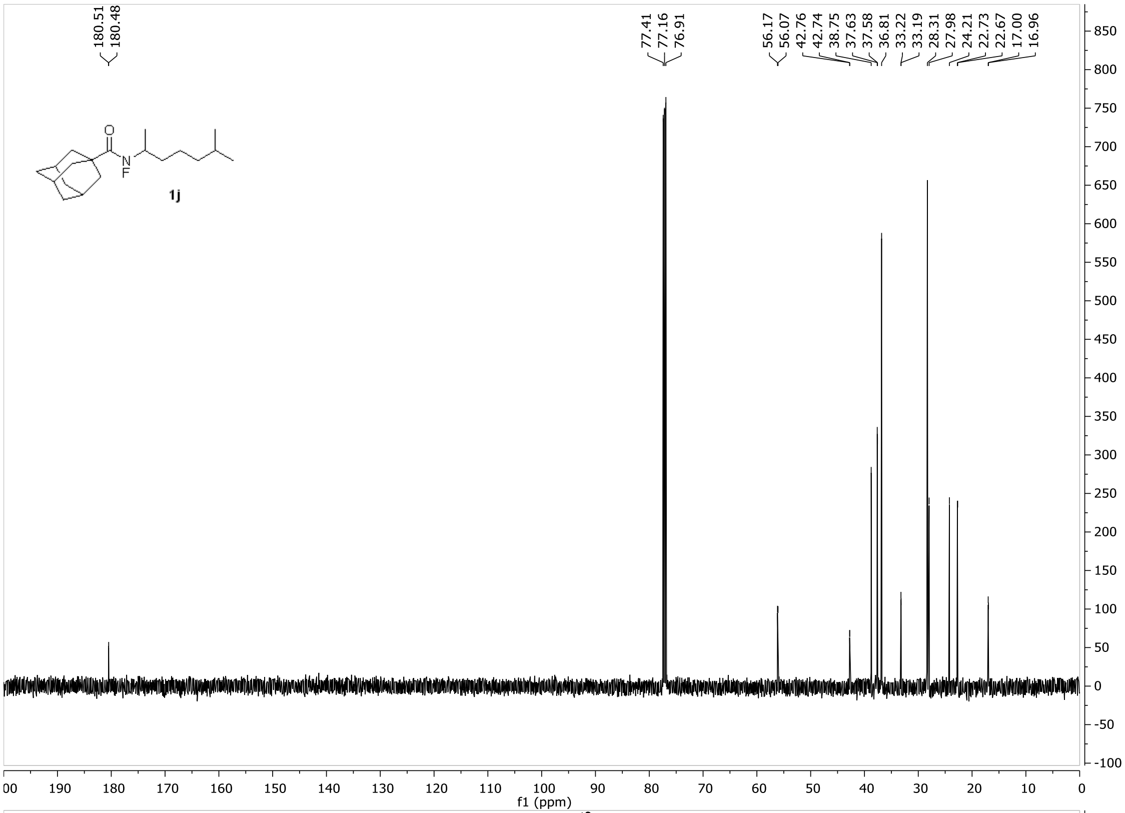
180.51
180.48
77.41
77.16
76.91
56.17
56.07
42.76
42.74
38.75
37.63
37.58
36.81
33.22
33.19
28.31
27.98
24.21
22.73
22.67
17.00
16.96
1j
f1 (ppm)

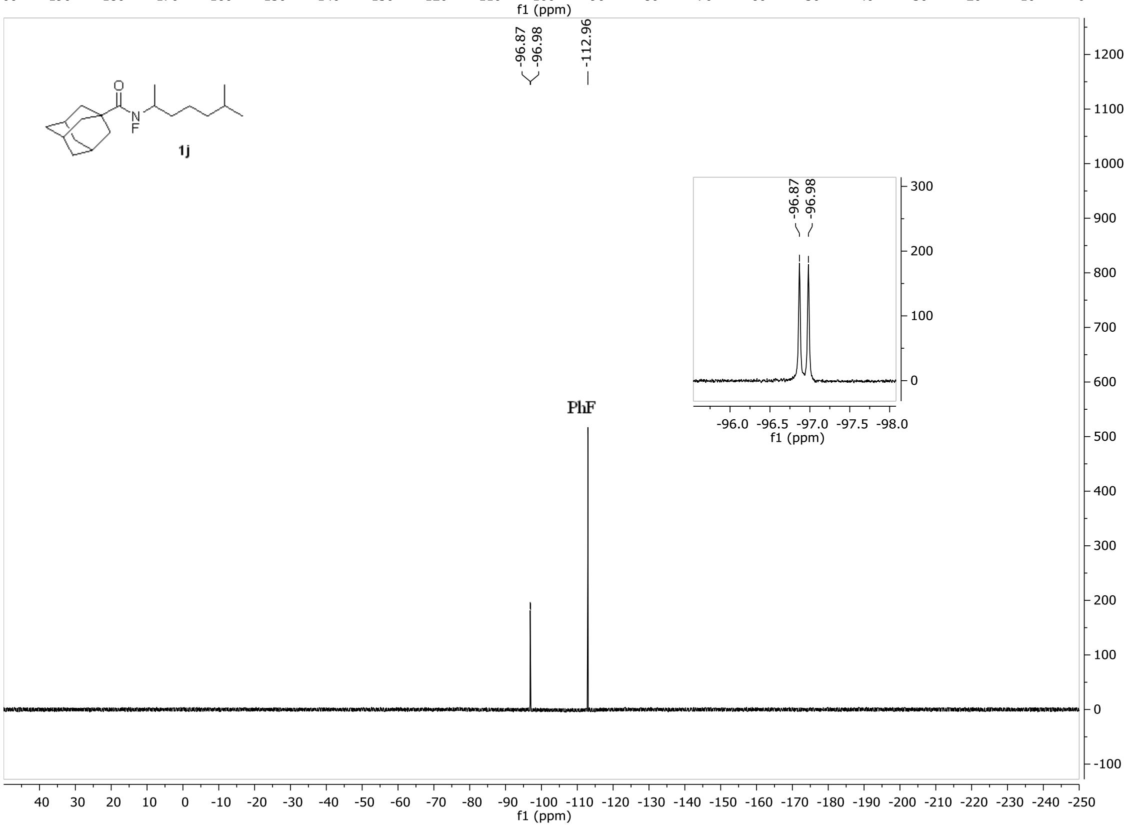
-96.87
-96.98
-112.96
1j
PhF
f1 (ppm)
f1 (ppm)

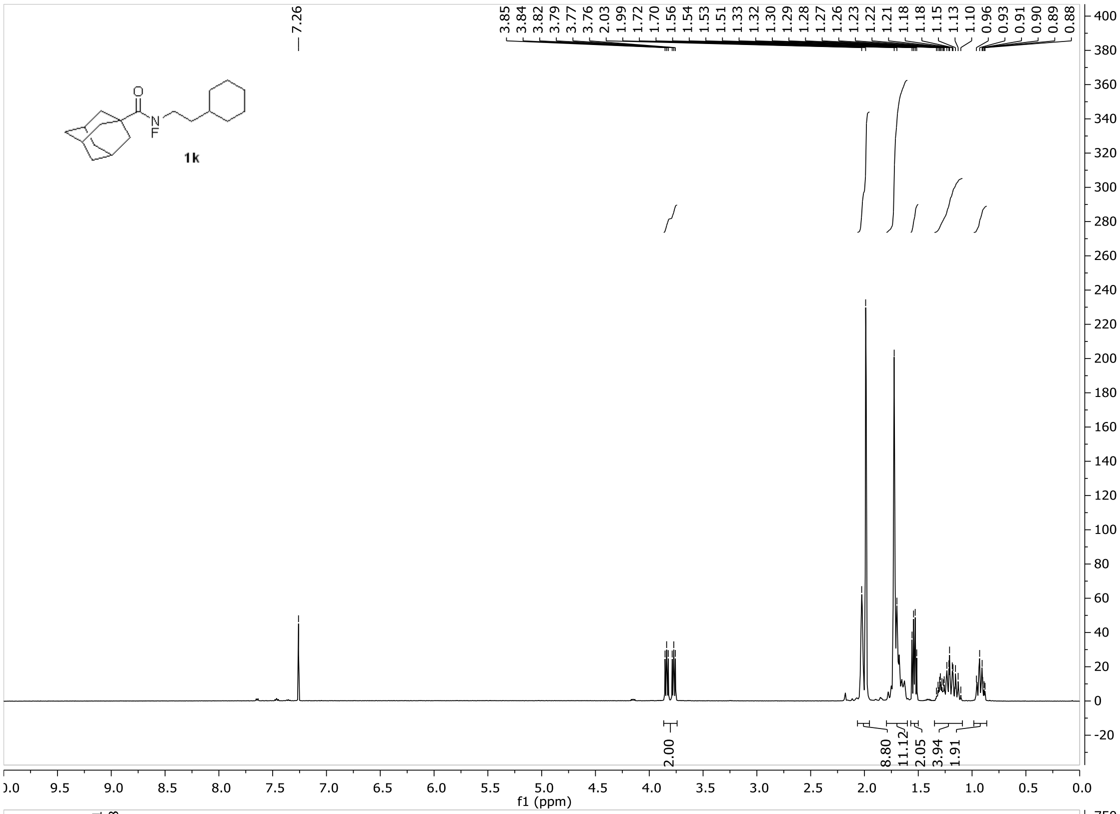
7.26
3.85 3.84 3.82 3.79 3.77 3.76 2.03 1.99 1.72 1.70 1.56 1.54 1.53 1.51 1.33 1.32 1.30 1.29 1.28 1.27 1.26 1.23 1.22 1.21 1.18 1.18 1.15 1.13 1.10 0.96 0.93 0.91 0.90 0.89 0.88
O
N
F
1k
2.00
8.80
11.12
2.05
3.94
1.91
f1 (ppm)

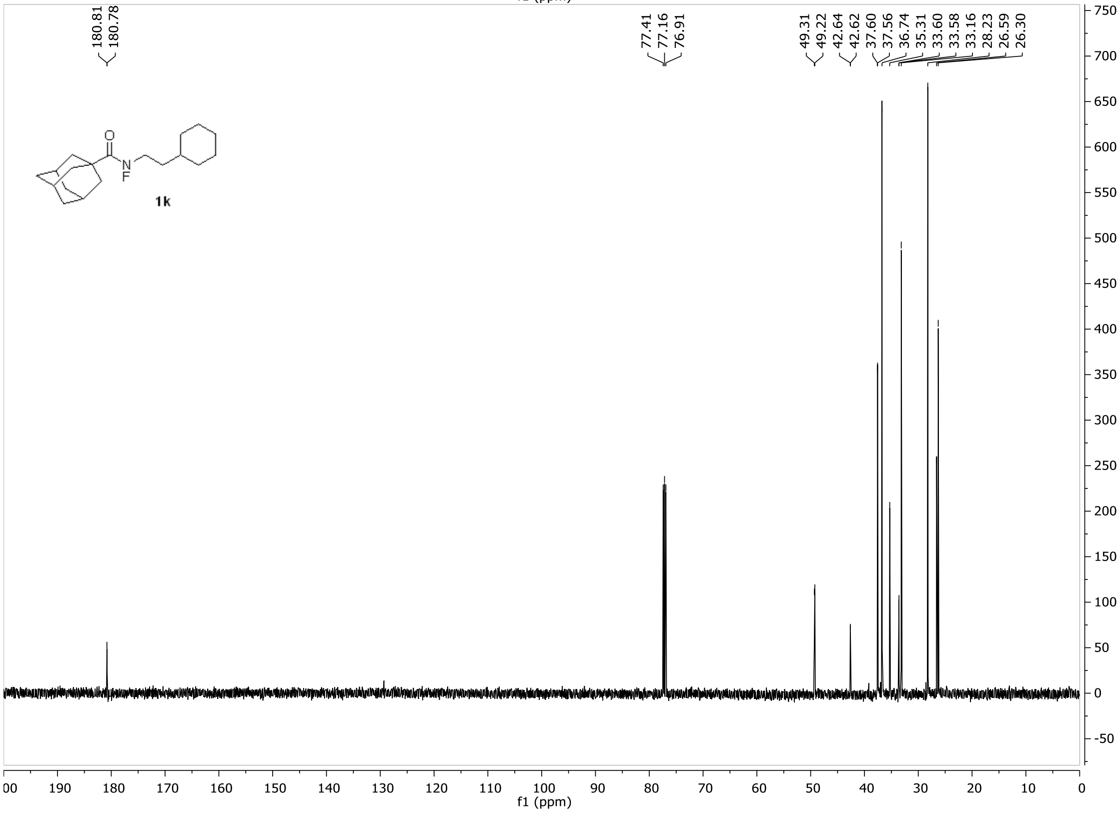
180.81
180.78
77.41
77.16
76.91
49.31 49.22 42.64 42.62 37.60 37.56 36.74 35.31 33.60 33.58 33.16 28.23 26.59 26.30
O
N
F
1k
f1 (ppm)

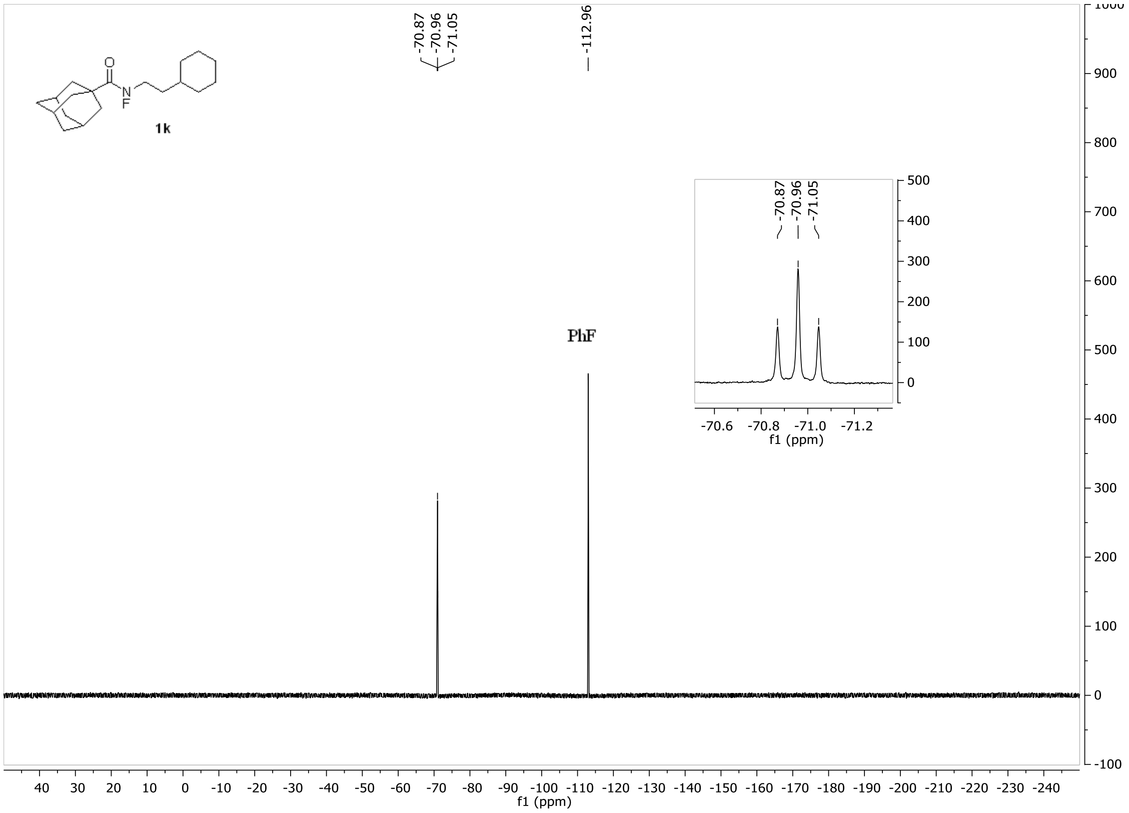
1k
-70.87
-70.96
-71.05
-112.96
PhF
f1 (ppm)

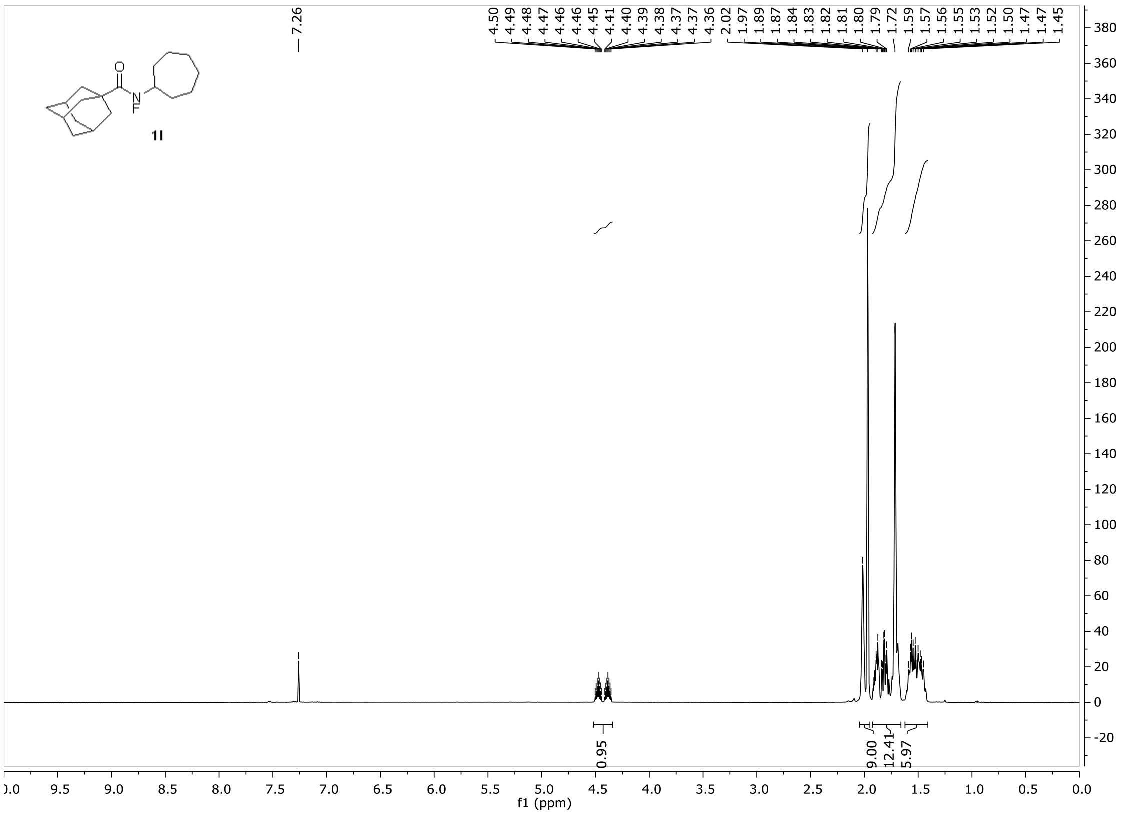
1l
7.26
0.95
9.00
12.41
5.97
f1 (ppm)

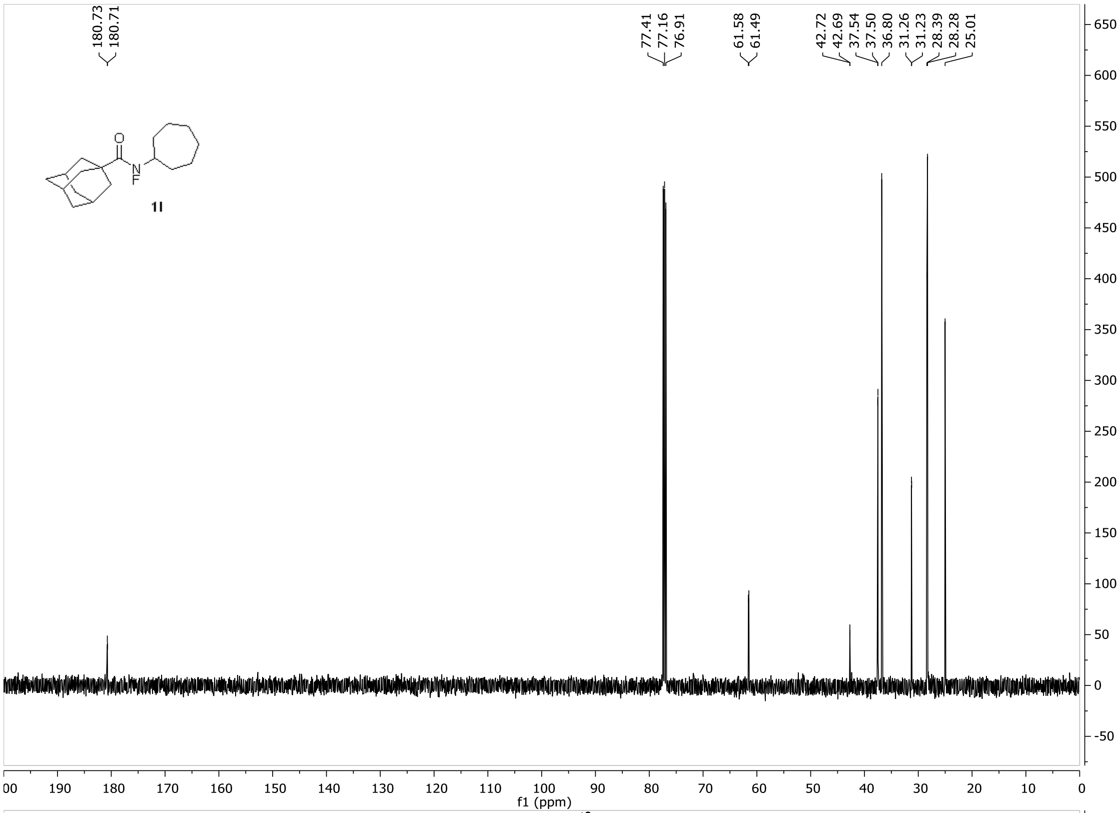
180.73
180.71
77.41
77.16
76.91
61.58
61.49
42.72
42.69
37.54
37.50
36.80
31.26
31.23
28.39
28.28
25.01
1l
f1 (ppm)

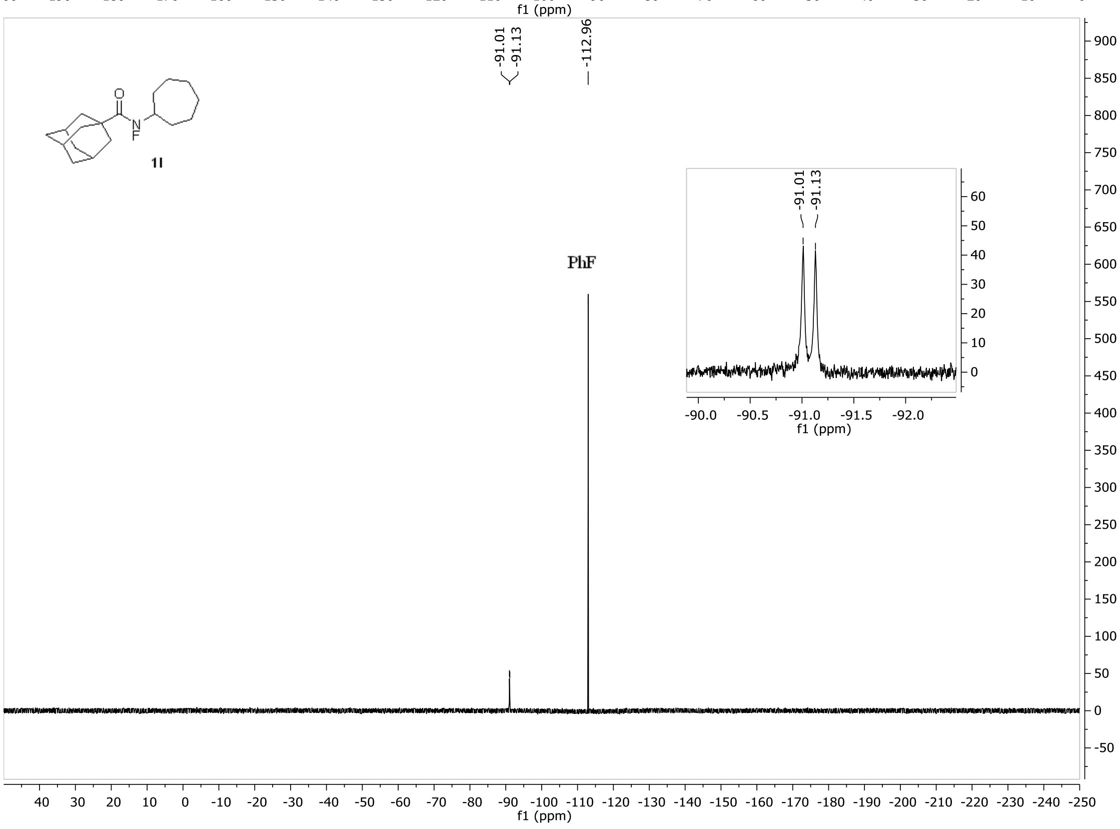
-91.01
-91.13
-112.96
1l
PhF
f1 (ppm)

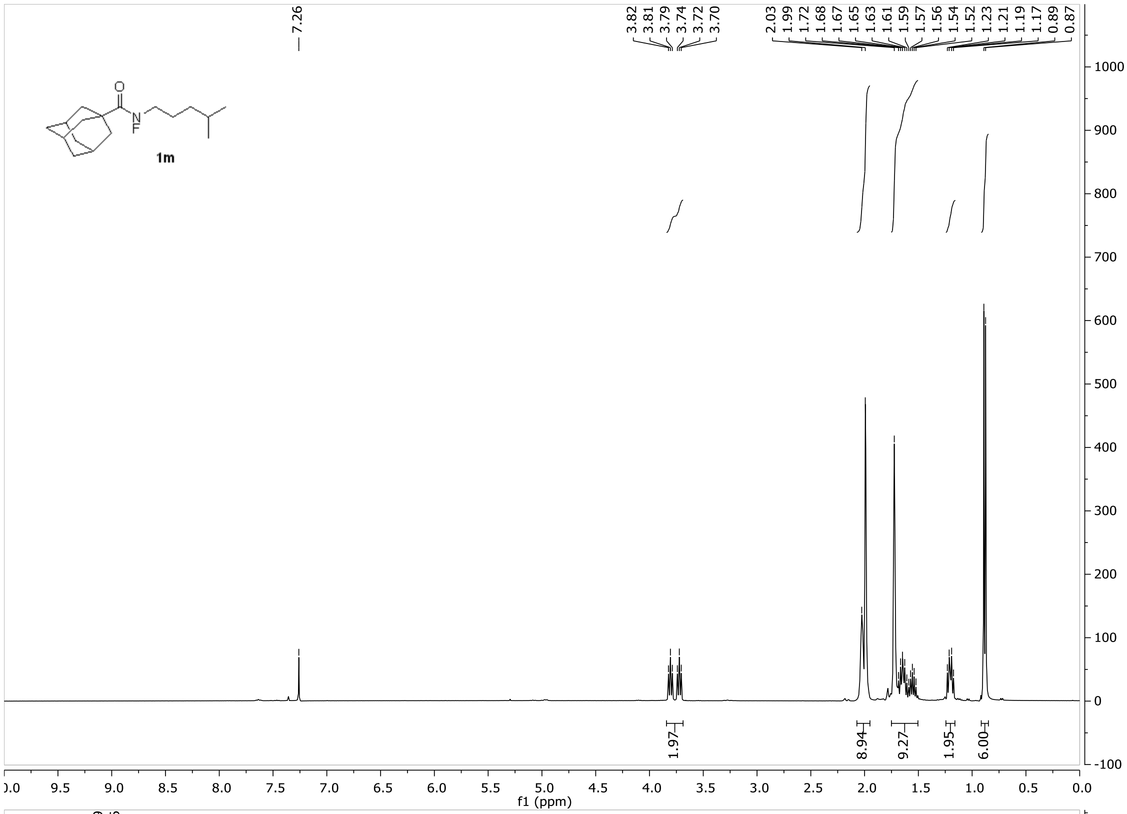

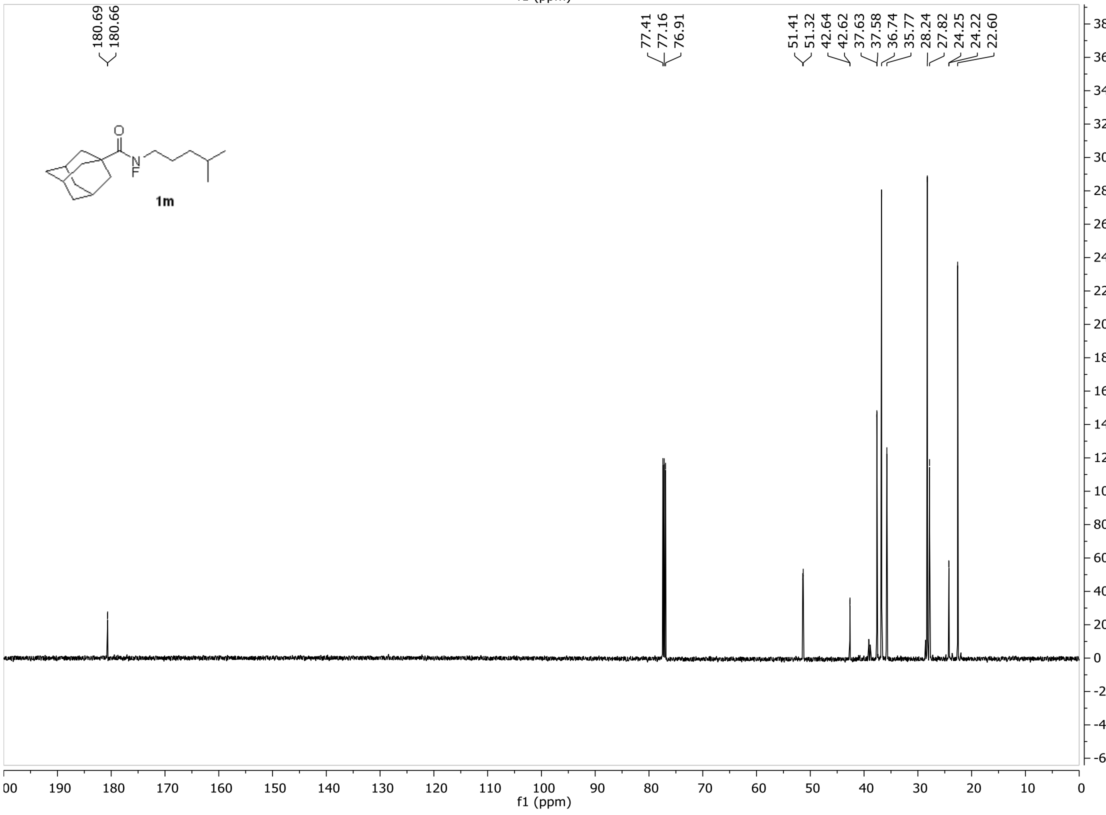

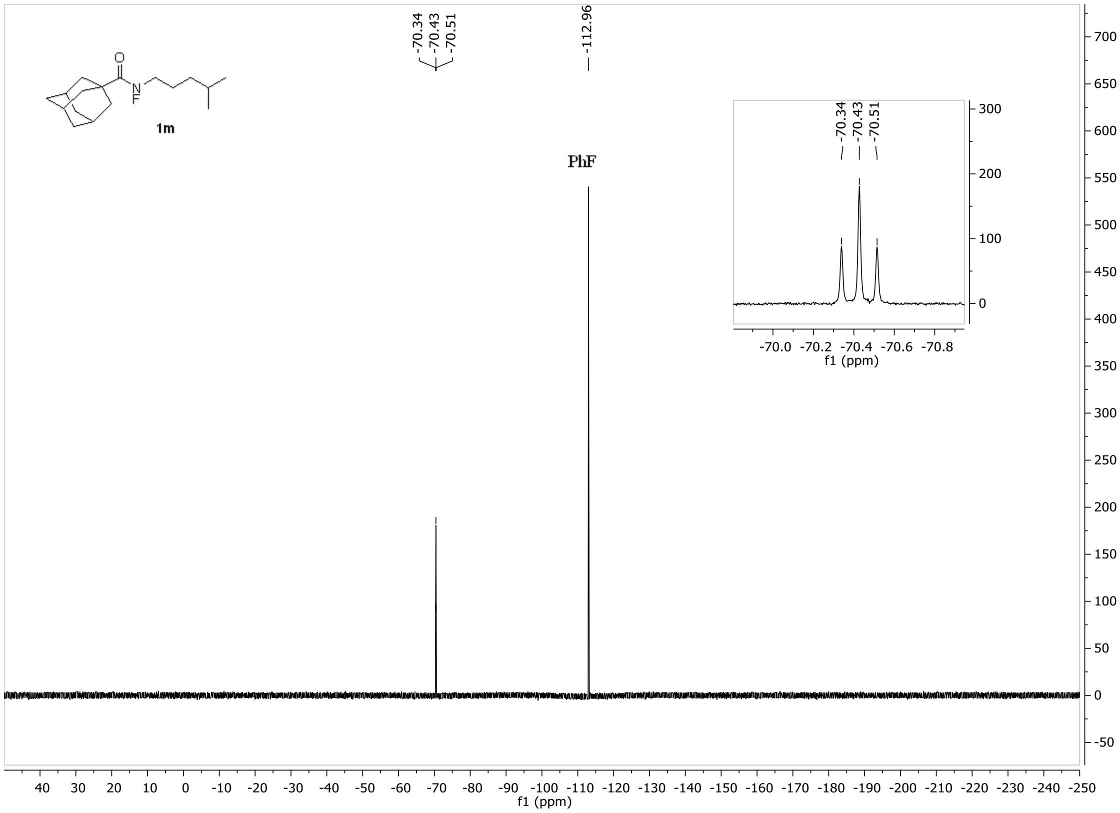

-70.34
-70.43
-70.51
-112.96
1m
PhF
f1 (ppm)

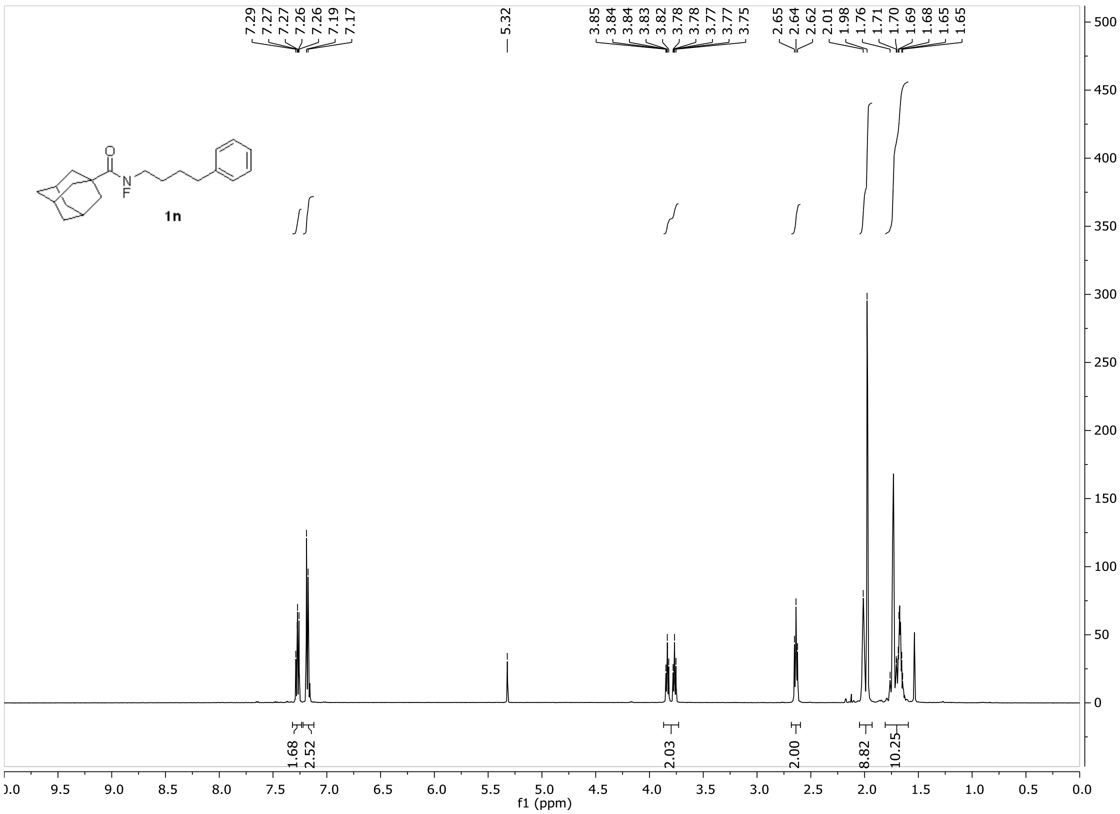

1n
1.68
2.52
2.03
2.00
8.82
10.25
f1 (ppm)

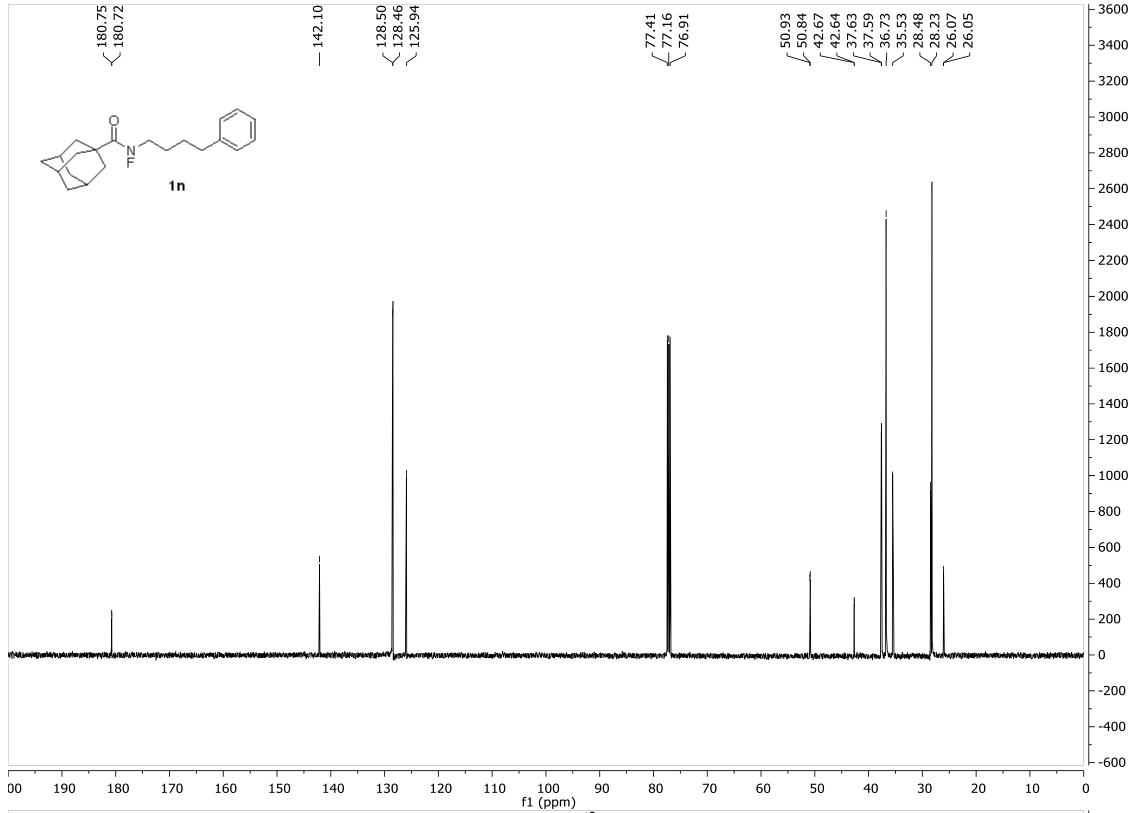
180.75
180.72
142.10
128.50
128.46
125.94
77.41
77.16
76.91
50.93
50.84
42.67
42.64
37.63
37.59
36.73
35.53
28.48
28.23
26.07
26.05
1n
f1 (ppm)

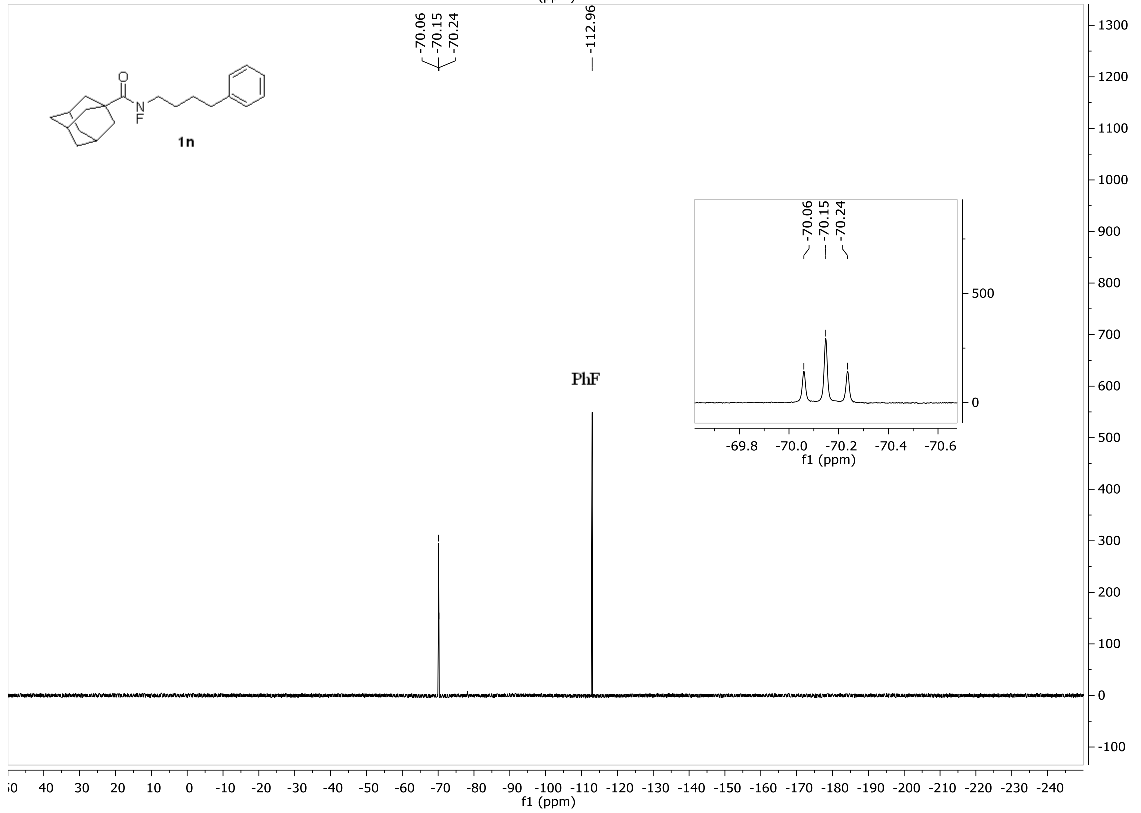
-70.06
-70.15
-70.24
-112.96
1n
PhF
f1 (ppm)

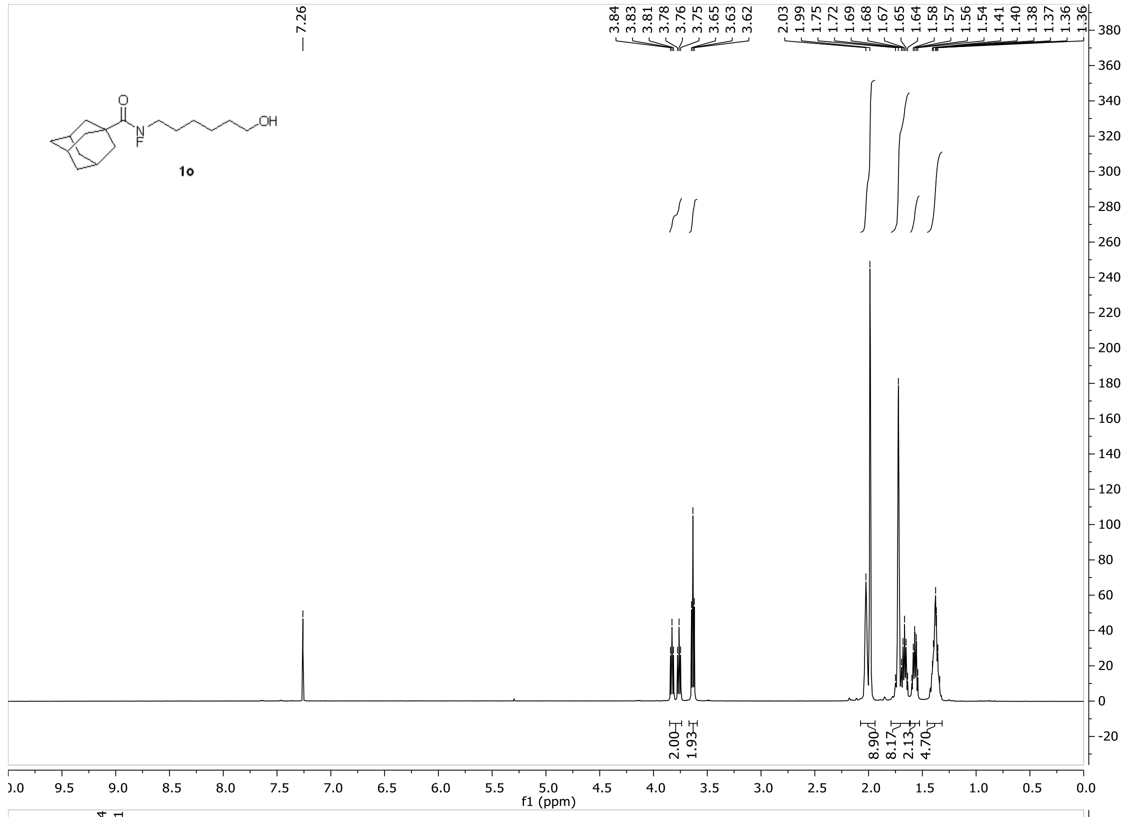
7.26
3.84
3.83
3.81
3.78
3.76
3.75
3.65
3.63
3.62
2.03
1.99
1.75
1.72
1.69
1.68
1.67
1.65
1.64
1.58
1.57
1.56
1.54
1.41
1.40
1.38
1.37
1.36
1.36
OH
N
F
O
1o
2.00
1.93
8.90
8.17
2.13
4.70
f1 (ppm)

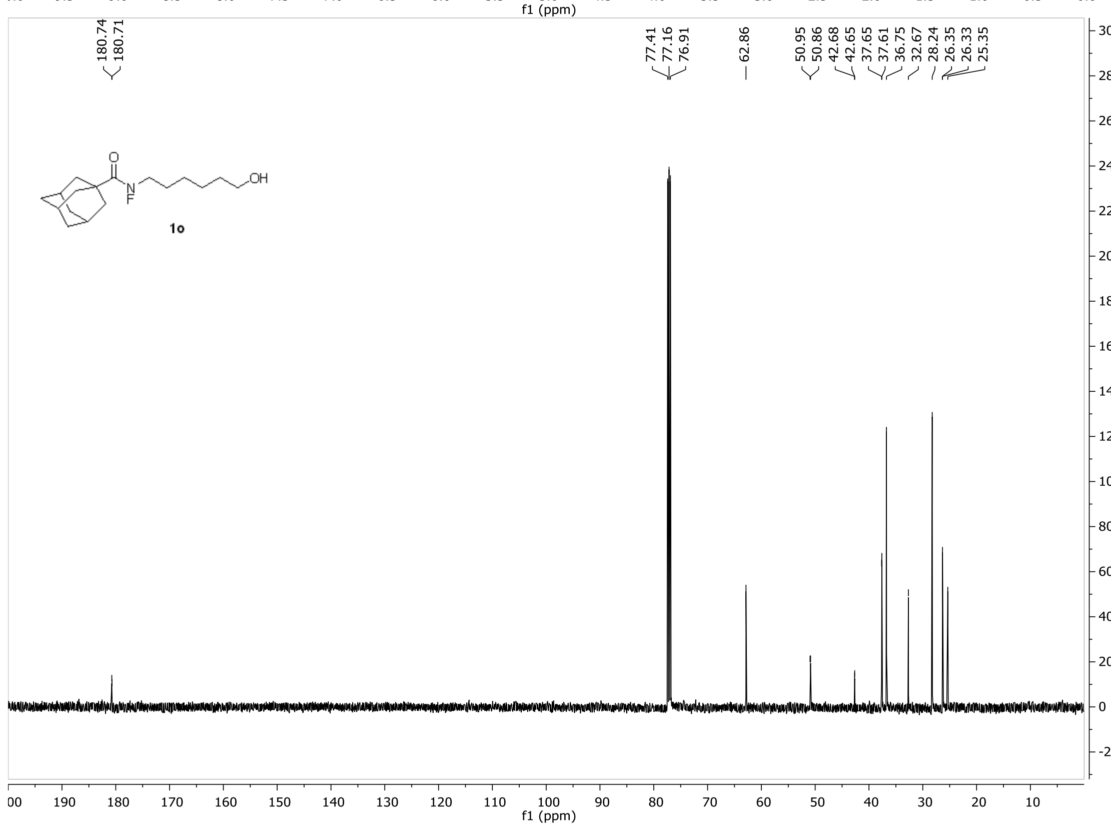
180.74
180.71
77.41
77.16
76.91
62.86
50.95
50.86
42.68
42.65
37.65
37.61
36.75
32.67
28.24
26.35
26.33
25.35
OH
N
F
O
1o
f1 (ppm)

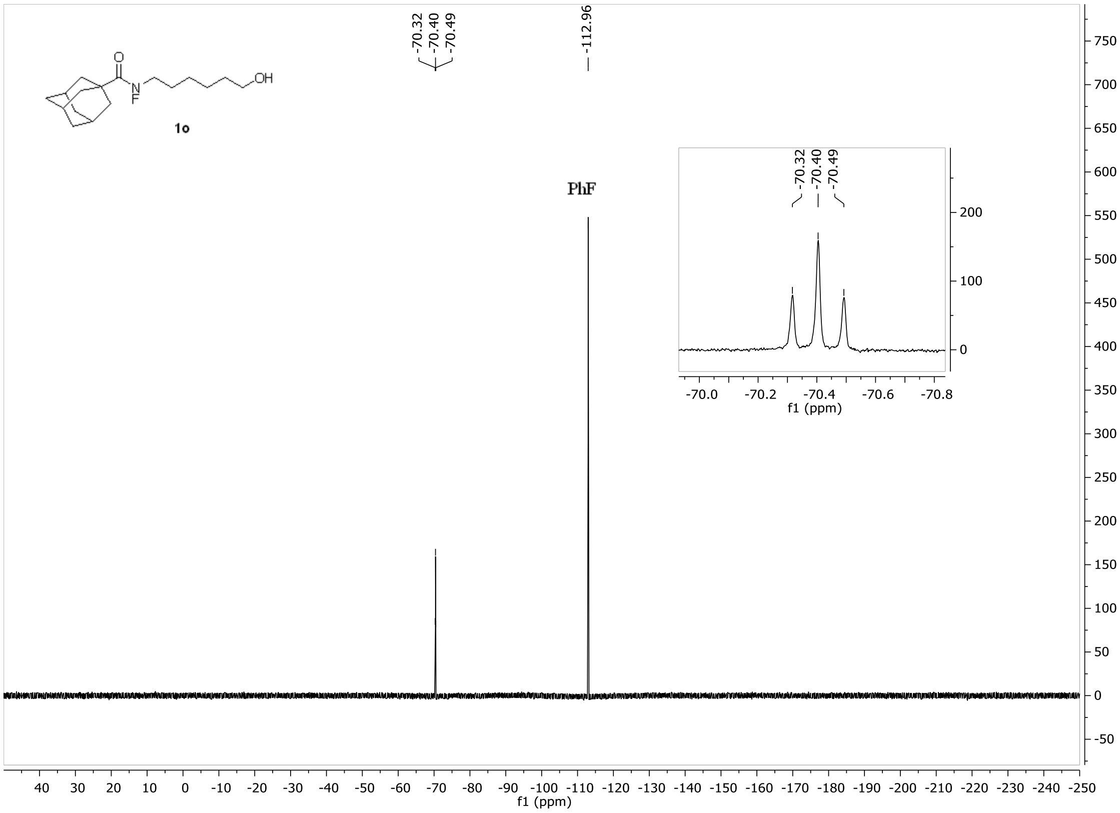
-70.32
-70.40
-70.49
-112.96
PhF
1o
f1 (ppm)

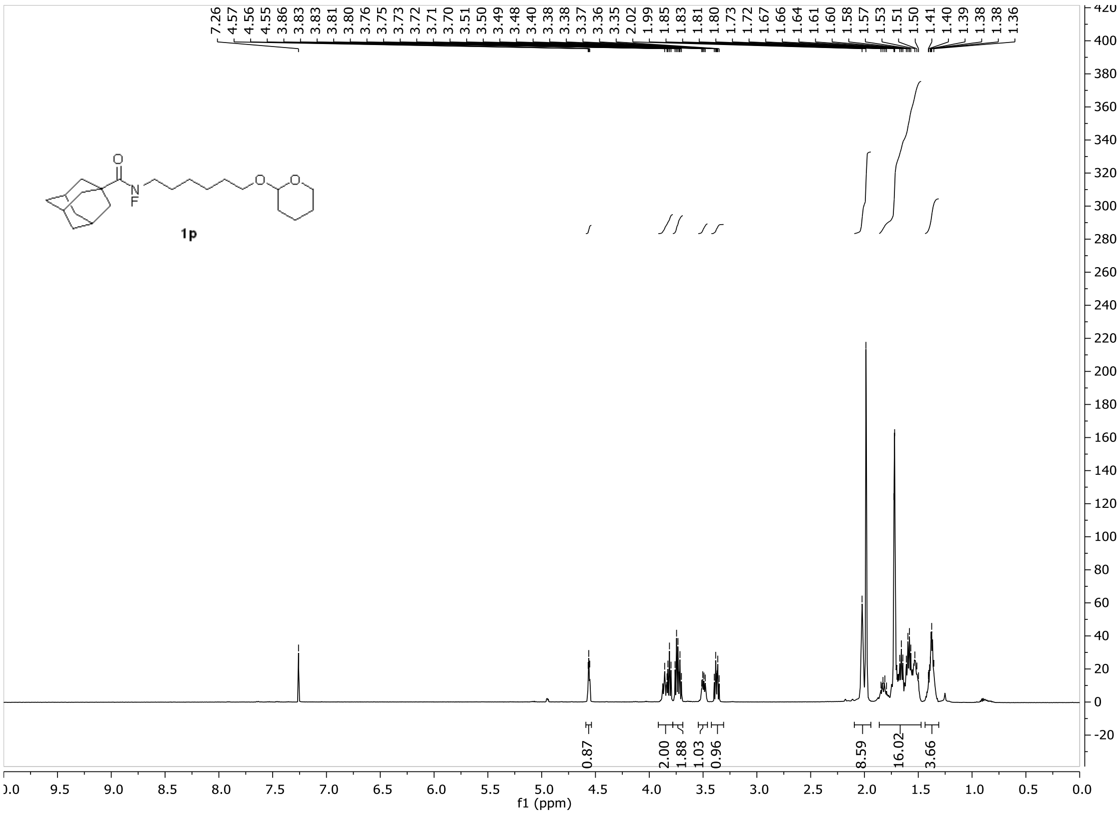
1p
0.87
2.00
1.88
1.03
0.96
8.59
16.02
3.66
f1 (ppm)

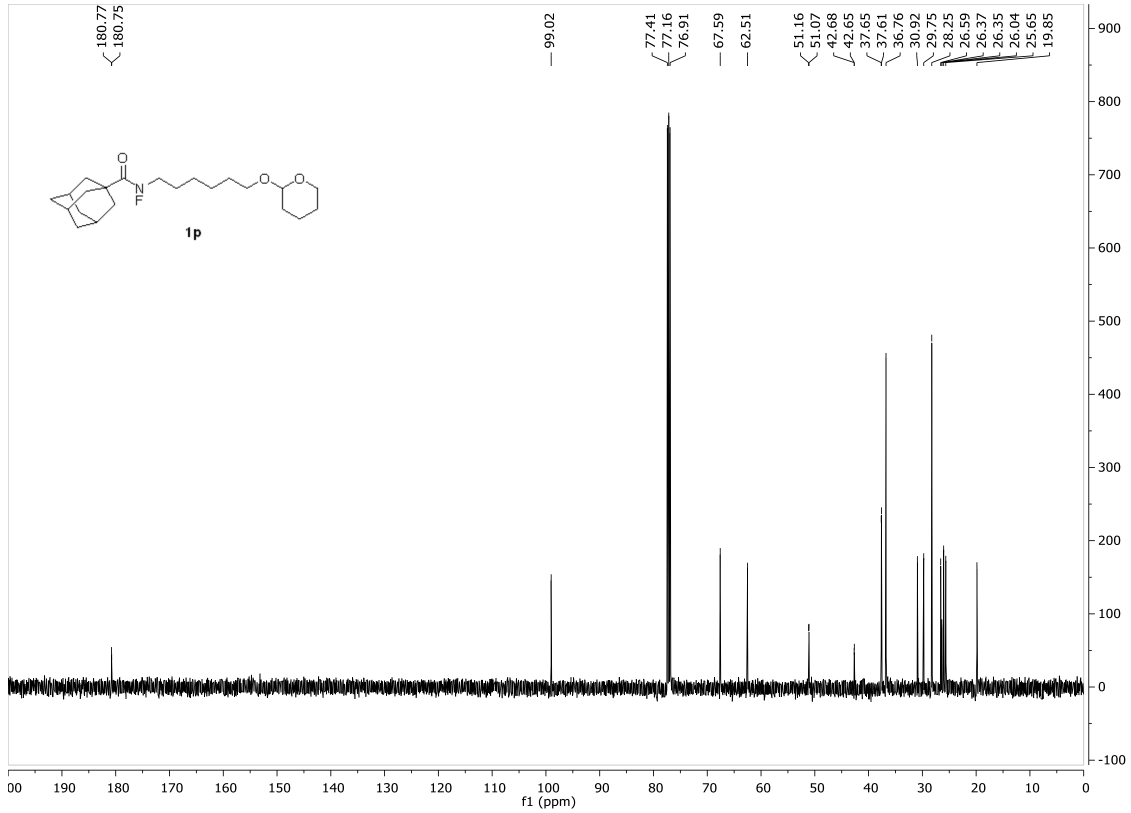
180.77
180.75
99.02
77.41
77.16
76.91
67.59
62.51
51.16
51.07
42.68
42.65
37.65
37.61
36.76
30.92
29.75
28.25
26.59
26.37
26.35
26.04
25.65
19.85
1p
f1 (ppm)

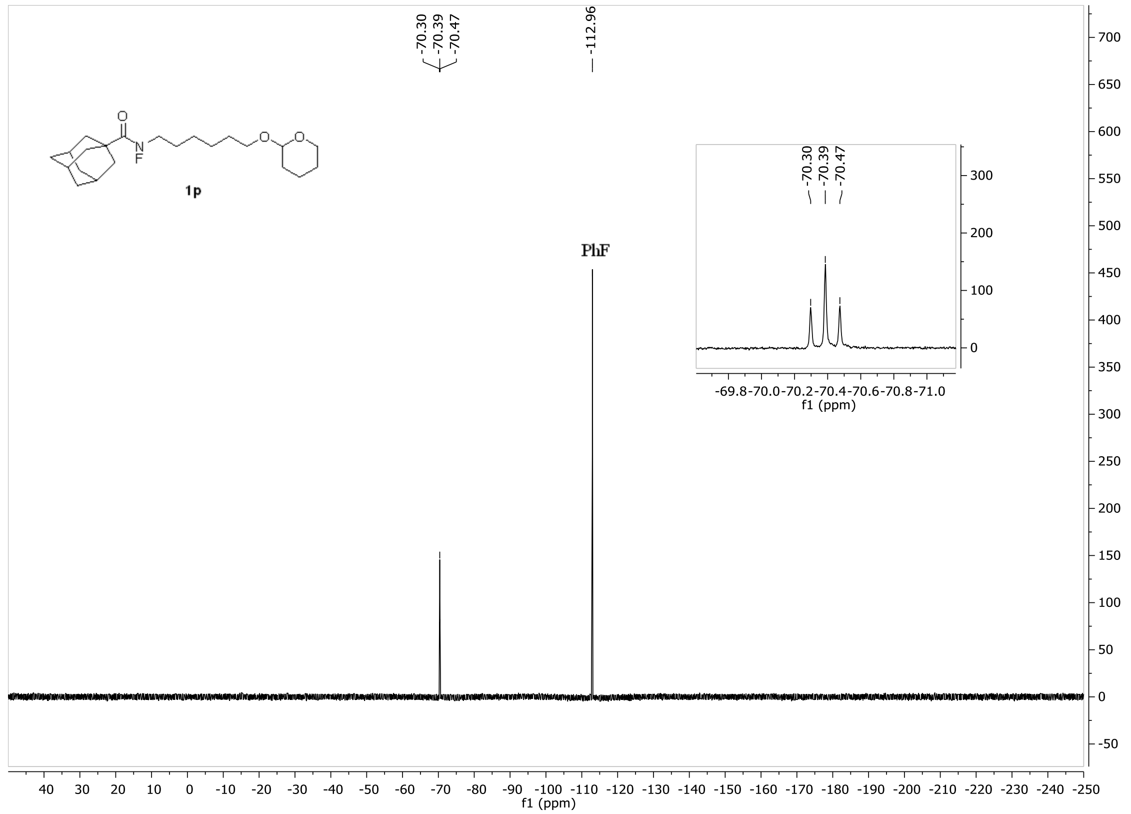
-70.30
-70.39
-70.47
-112.96
1p
PhF
f1 (ppm)

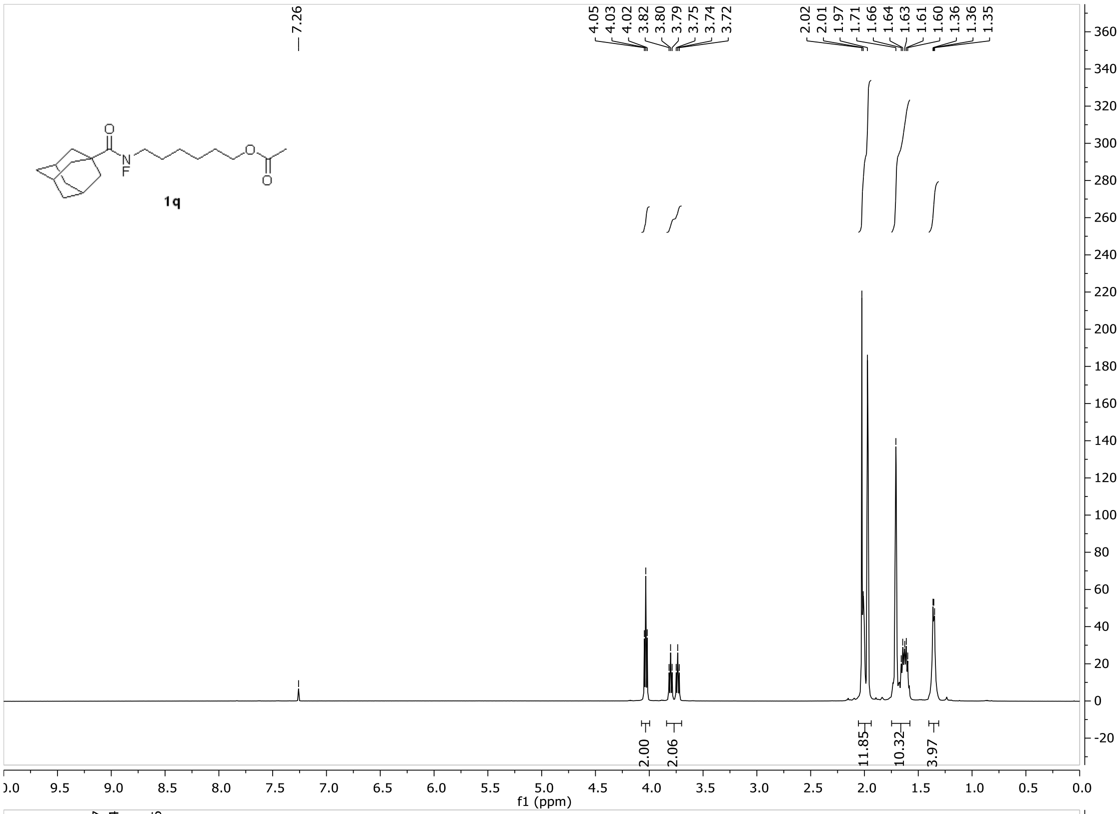
7.26
4.05
4.03
4.02
3.82
3.80
3.79
3.75
3.74
3.72
2.02
2.01
1.97
1.71
1.66
1.64
1.63
1.61
1.60
1.36
1.36
1.35
1q
2.00
2.06
11.85
10.32
3.97
f1 (ppm)

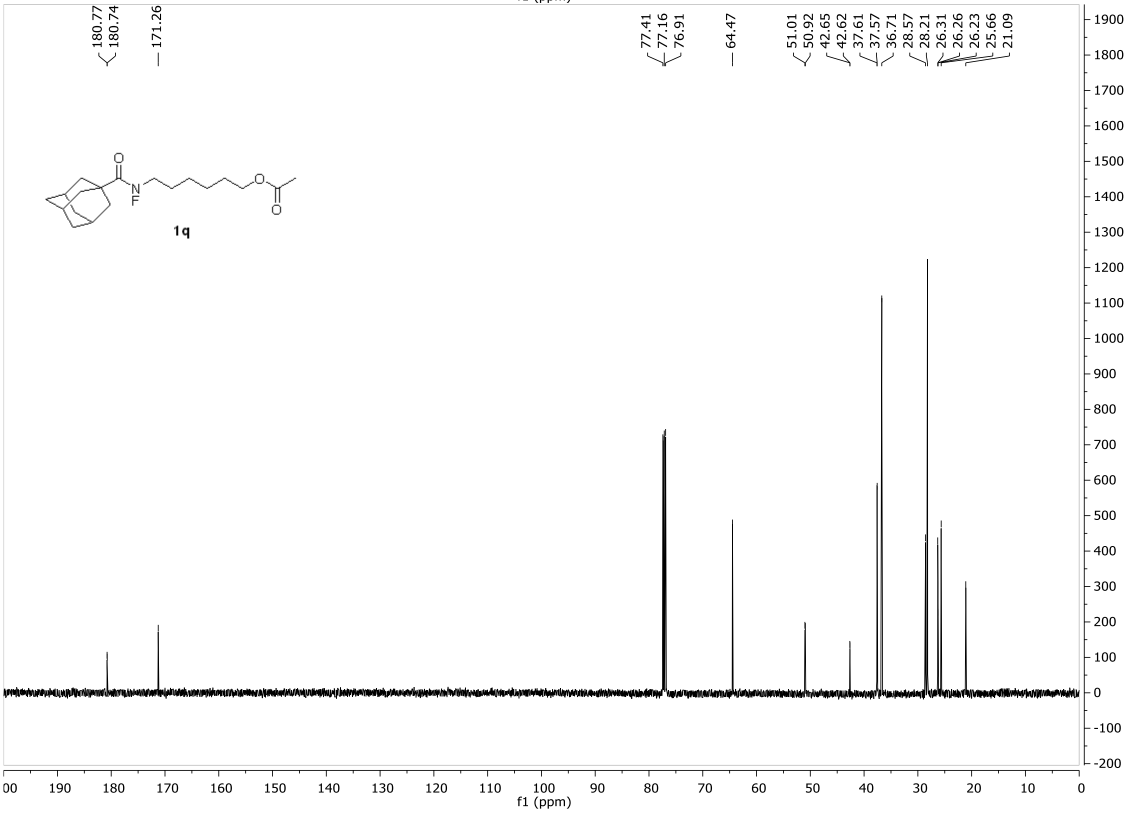
180.77
180.74
171.26
77.41
77.16
76.91
64.47
51.01
50.92
42.65
42.62
37.61
37.57
36.71
28.57
28.21
26.31
26.26
26.23
25.66
21.09
1q
f1 (ppm)

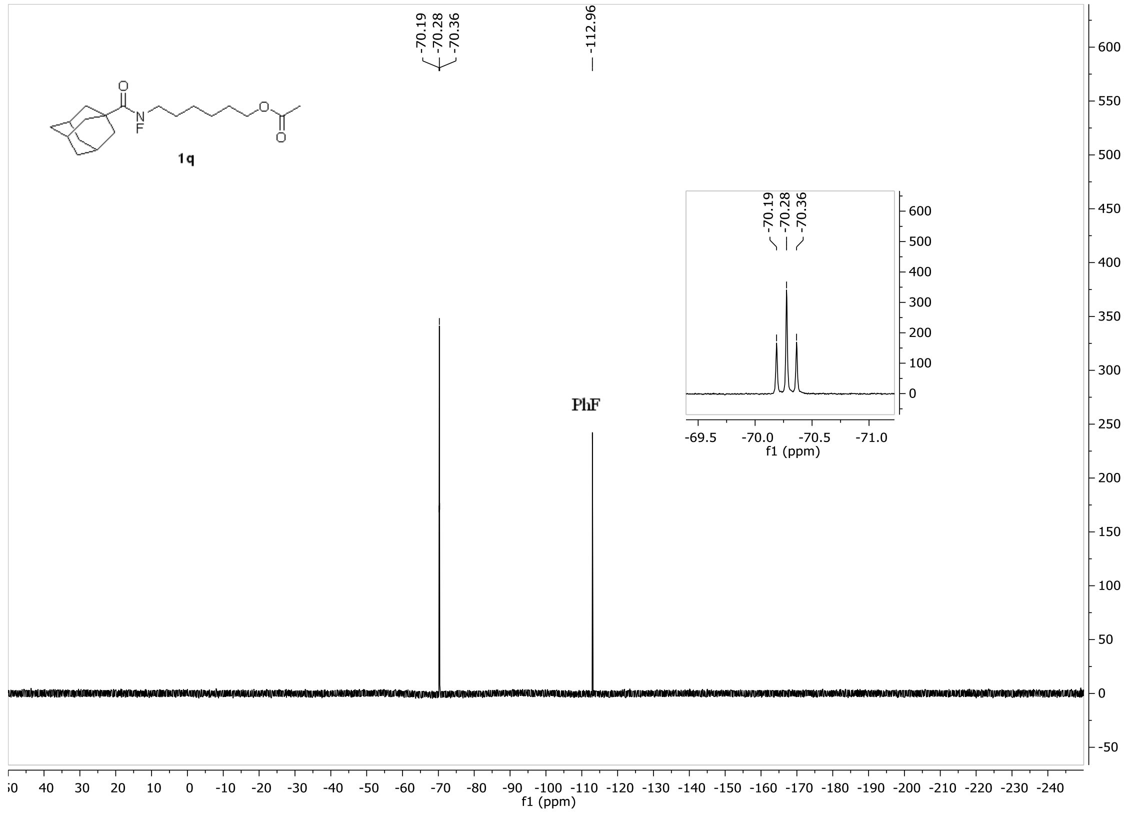
-70.19
-70.28
-70.36
-112.96
1q
PhF
f1 (ppm)

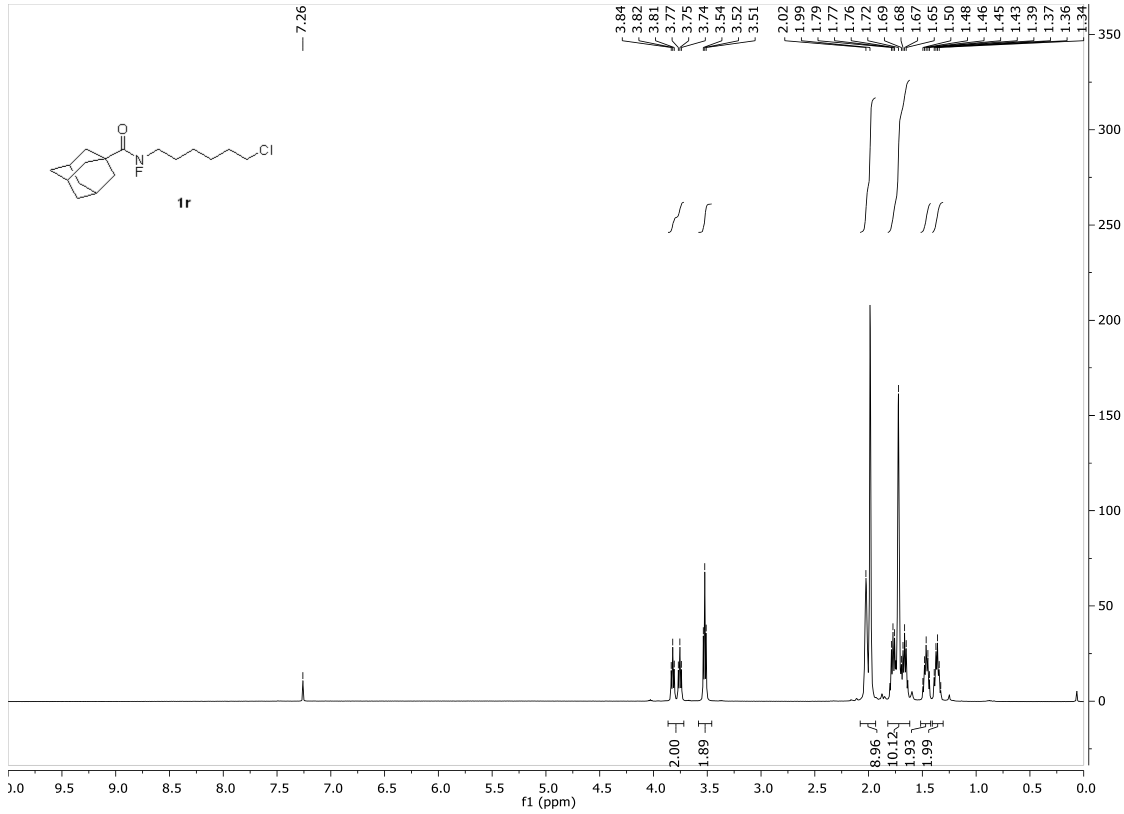
7.26
1r
2.00
1.89
8.96
10.12
1.93
1.99
f1 (ppm)

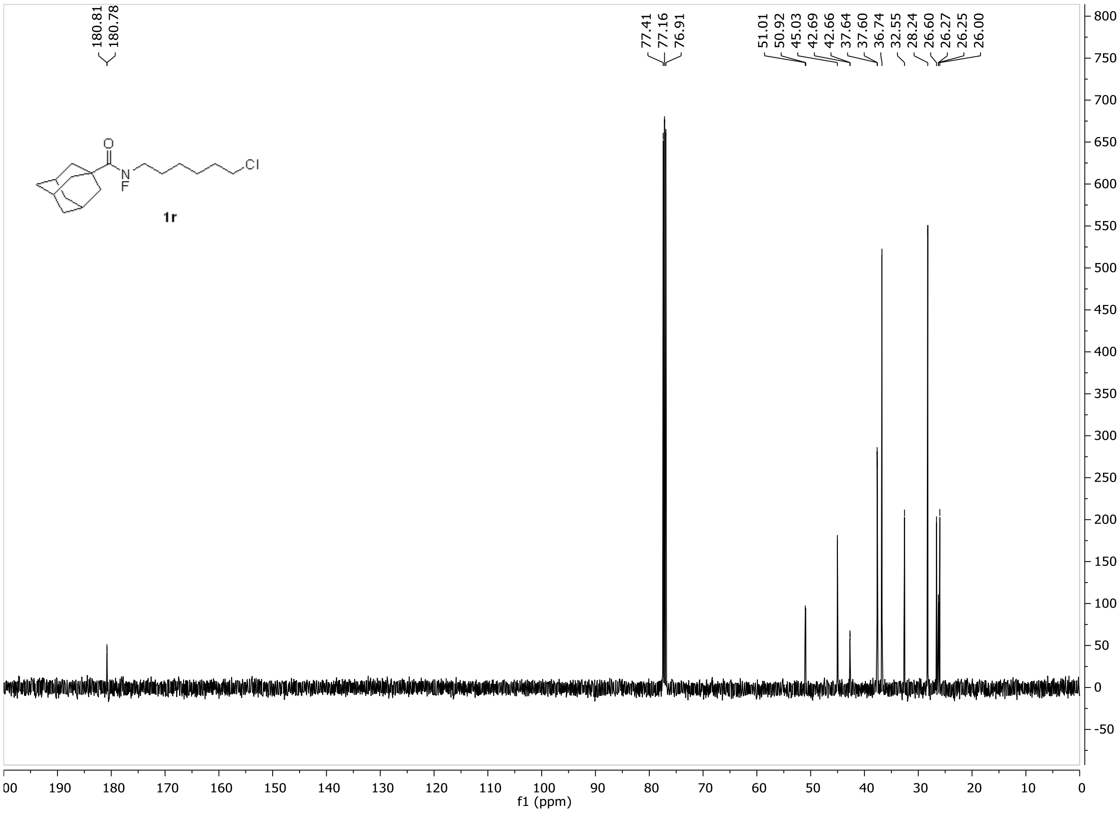
180.81
180.78
77.41
77.16
76.91
51.01
50.92
45.03
42.69
42.66
37.64
37.60
36.74
32.55
28.24
26.60
26.27
26.25
26.00
1r
f1 (ppm)

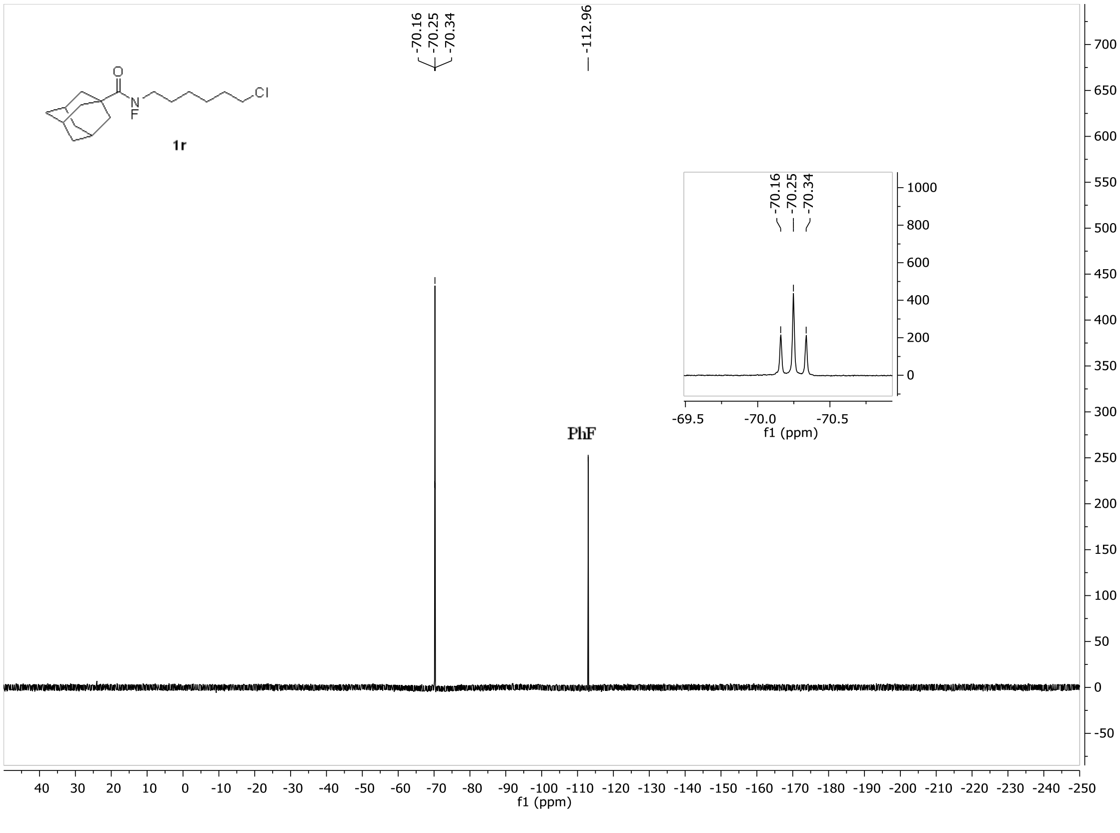
-70.16
-70.25
-70.34
-112.96
1r
PhF
f1 (ppm)

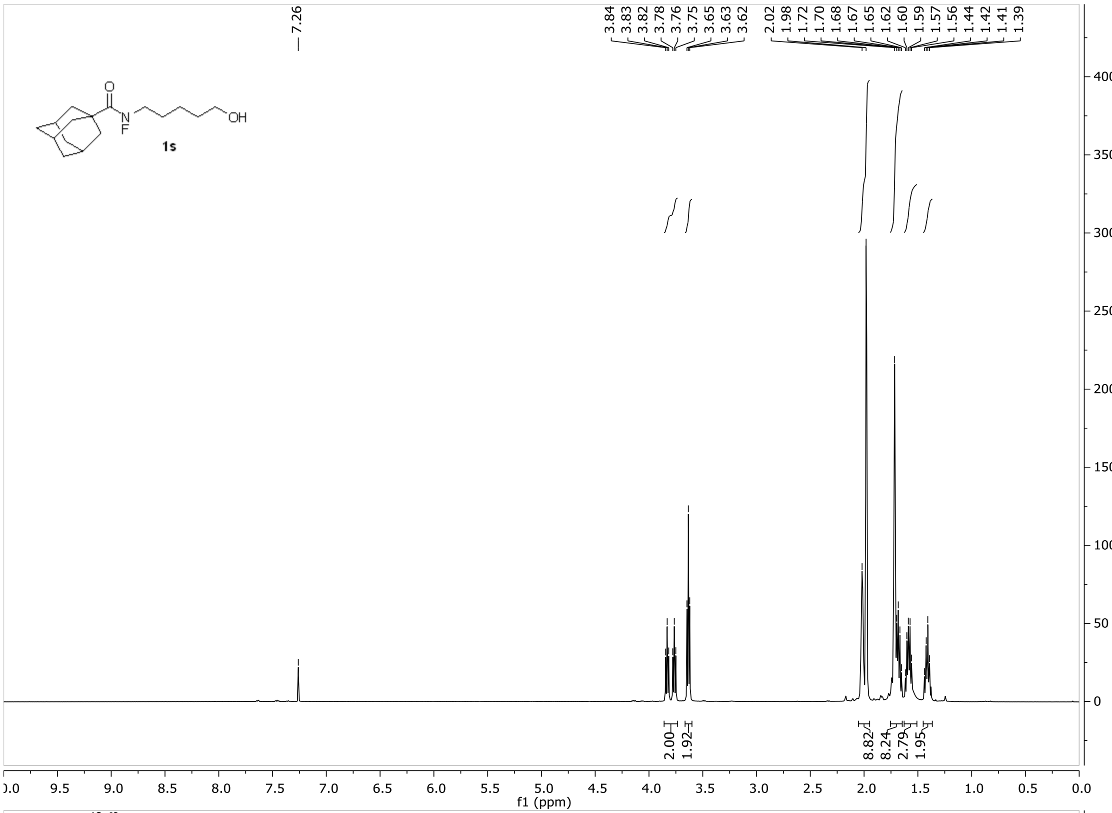

1s
OH
N
F
O
7.26
3.84
3.83
3.82
3.78
3.76
3.75
3.65
3.63
3.62
2.02
1.98
1.72
1.70
1.68
1.67
1.65
1.62
1.60
1.59
1.57
1.56
1.44
1.42
1.41
1.39
2.00
1.92
8.82
8.24
2.79
1.95
f1 (ppm)

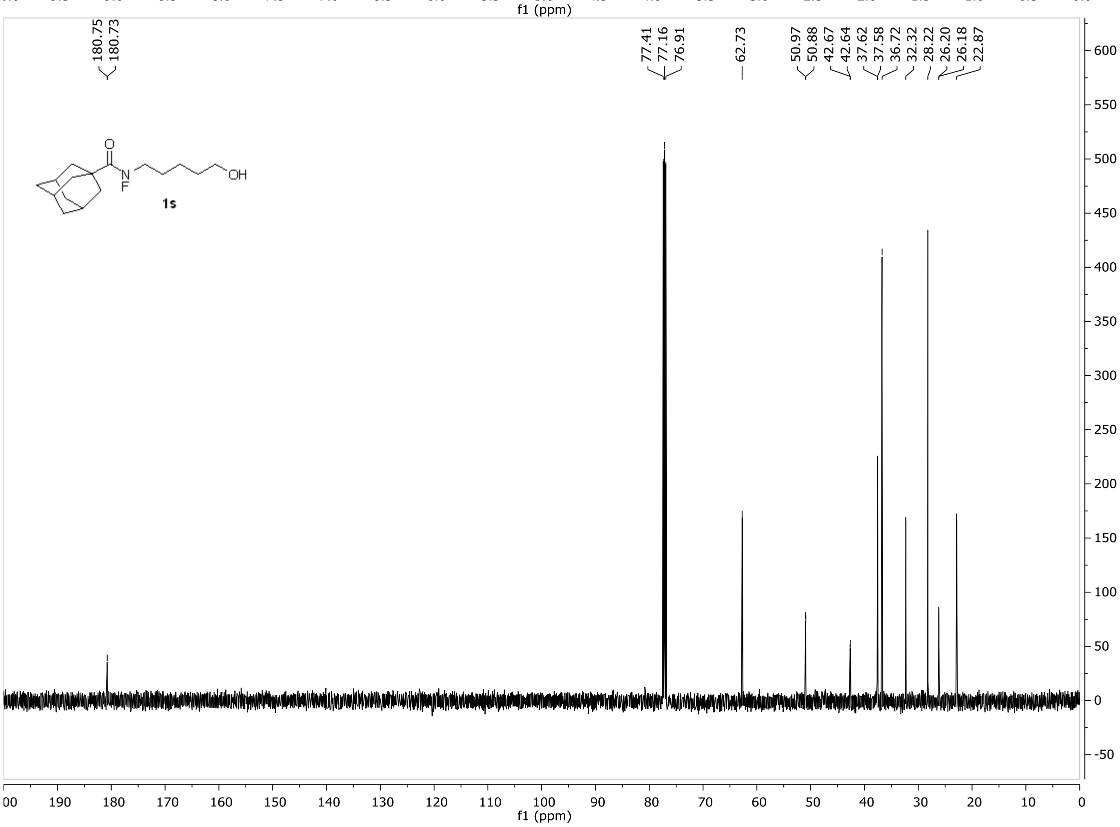

1s
OH
N
F
O
180.75
180.73
77.41
77.16
76.91
62.73
50.97
50.88
42.67
42.64
37.62
37.58
36.72
32.32
28.22
26.20
26.18
22.87
f1 (ppm)

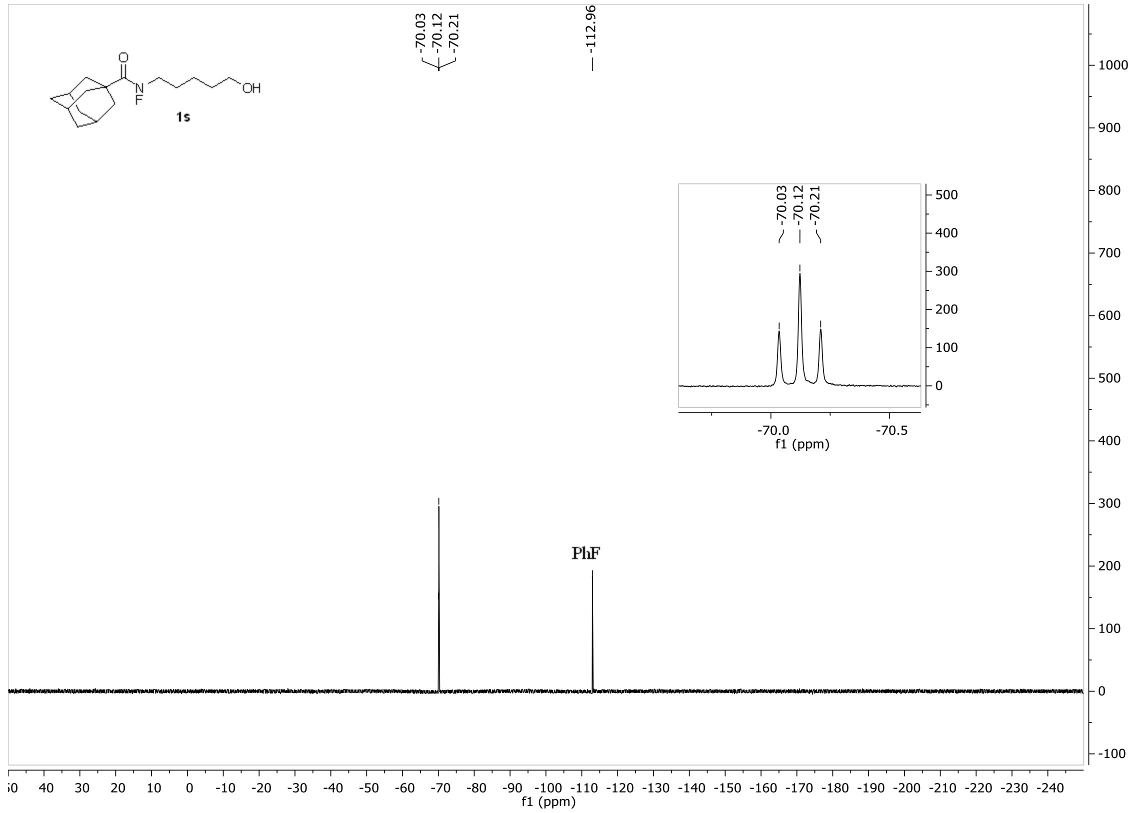
-70.03
-70.12
-70.21
-112.96
O
N
F
OH
1s
-70.03
-70.12
-70.21
-70.0
-70.5
f1 (ppm)
PhF
f1 (ppm)

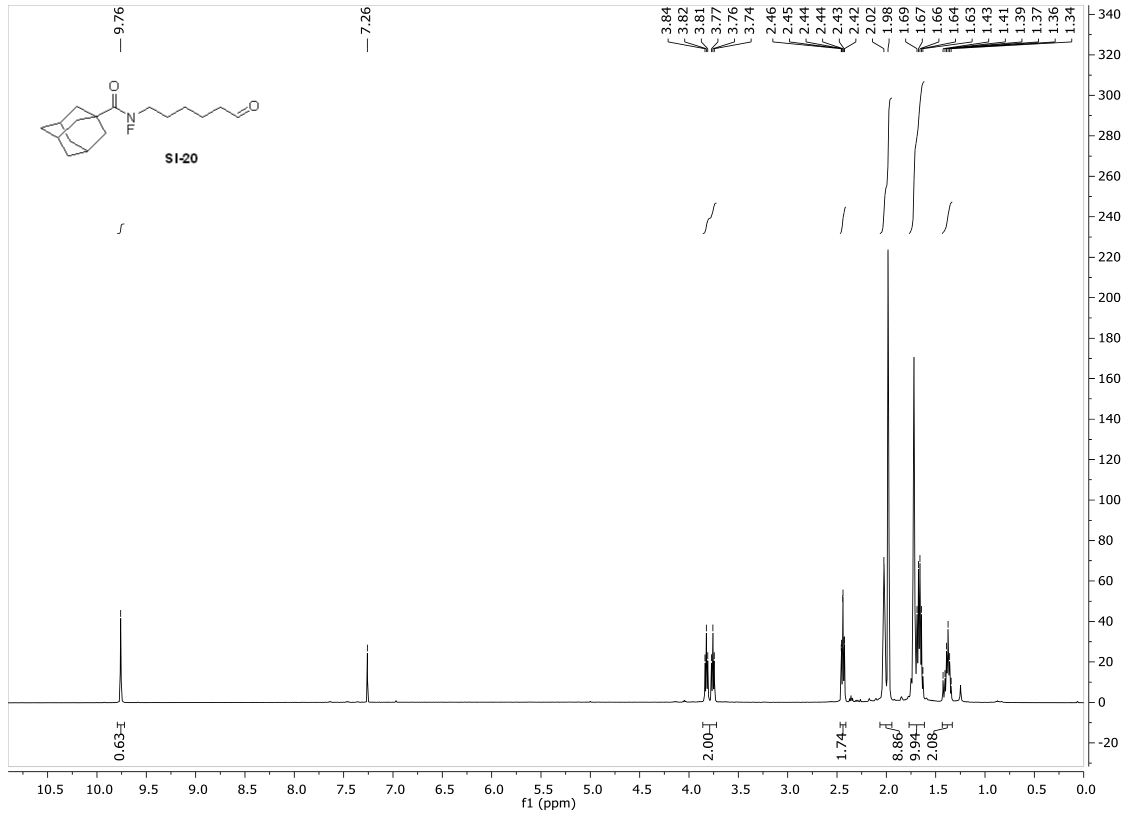
9.76
7.26
3.84
3.82
3.81
3.77
3.76
3.74
2.46
2.45
2.44
2.44
2.43
2.42
2.02
1.98
1.69
1.67
1.66
1.64
1.63
1.43
1.41
1.39
1.37
1.36
1.34
O
N
F
O
SI-20
0.63
2.00
1.74
8.86
9.94
2.08
f1 (ppm)

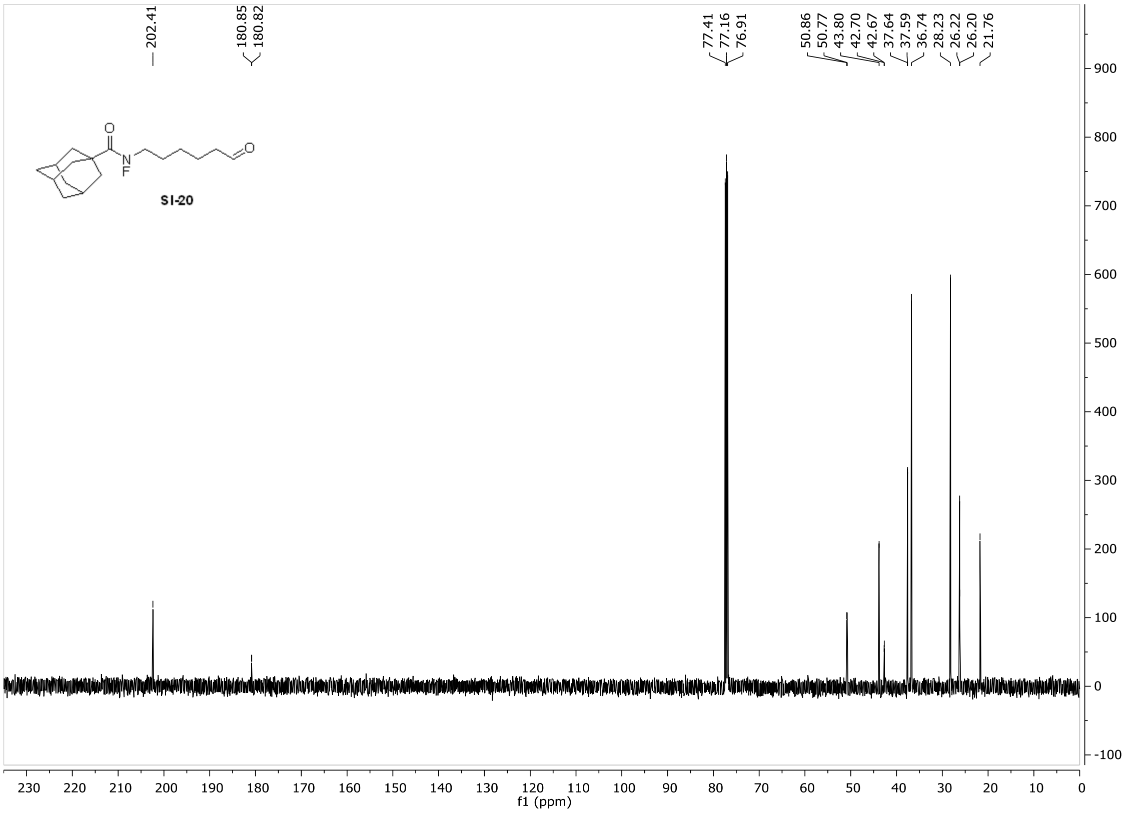
202.41
180.85
180.82
77.41
77.16
76.91
50.86
50.77
43.80
42.70
42.67
37.64
37.59
36.74
28.23
26.22
26.20
21.76
SI-20
f1 (ppm)

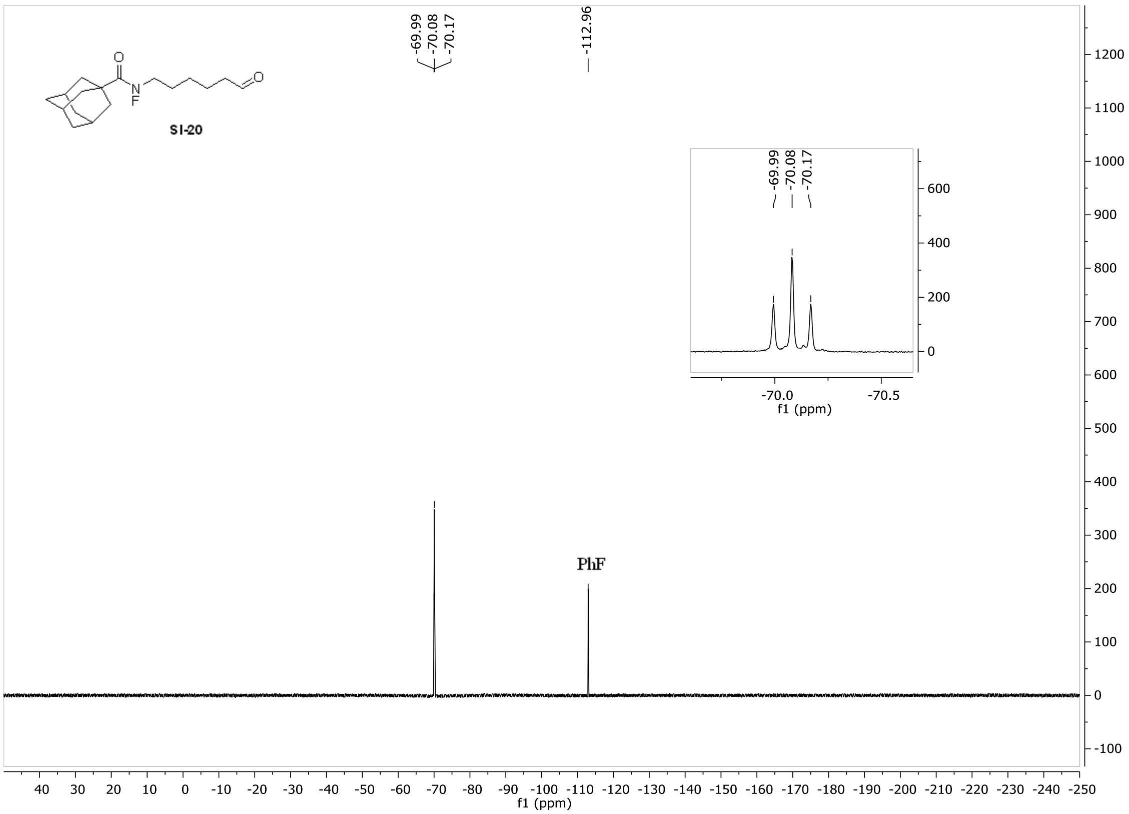
-69.99
-70.08
-70.17
-112.96
SI-20
PhF
f1 (ppm)

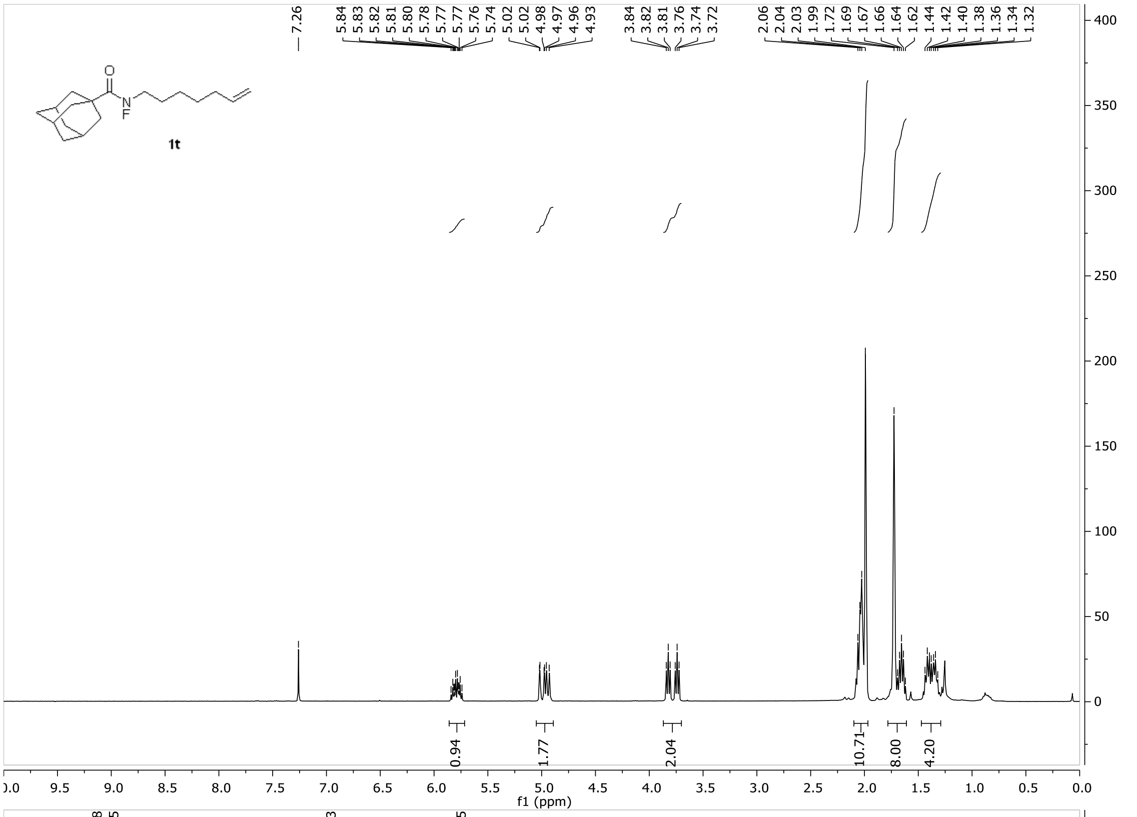
7.26
5.84
5.83
5.82
5.81
5.80
5.78
5.77
5.77
5.76
5.74
5.02
5.02
4.98
4.97
4.96
4.93
3.84
3.82
3.81
3.76
3.74
3.72
2.06
2.04
2.03
1.99
1.72
1.69
1.67
1.66
1.64
1.62
1.44
1.42
1.40
1.38
1.36
1.34
1.32
O
N
F
1t
0.94
1.77
2.04
10.71
8.00
4.20
f1 (ppm)

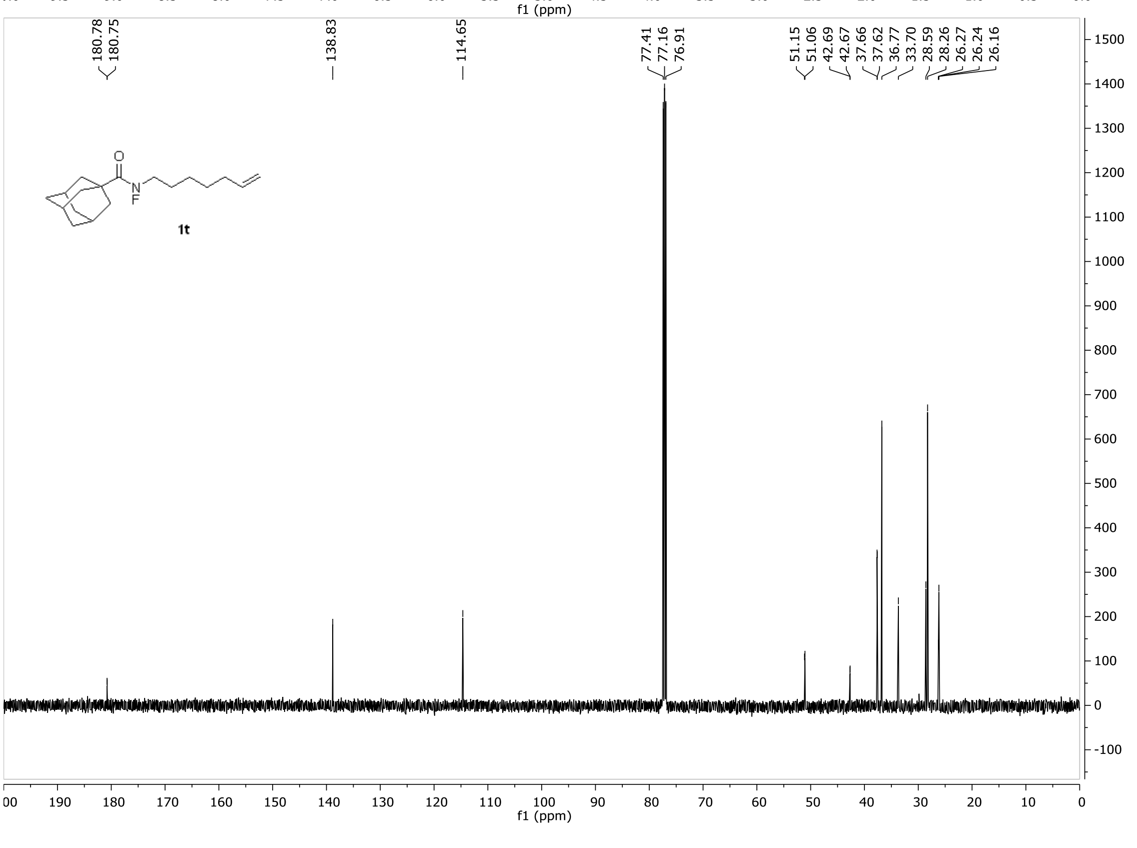
180.78
180.75
138.83
114.65
77.41
77.16
76.91
51.15
51.06
42.69
42.67
37.66
37.62
36.77
33.70
28.59
28.26
26.27
26.24
26.16
O
N
F
1t
f1 (ppm)

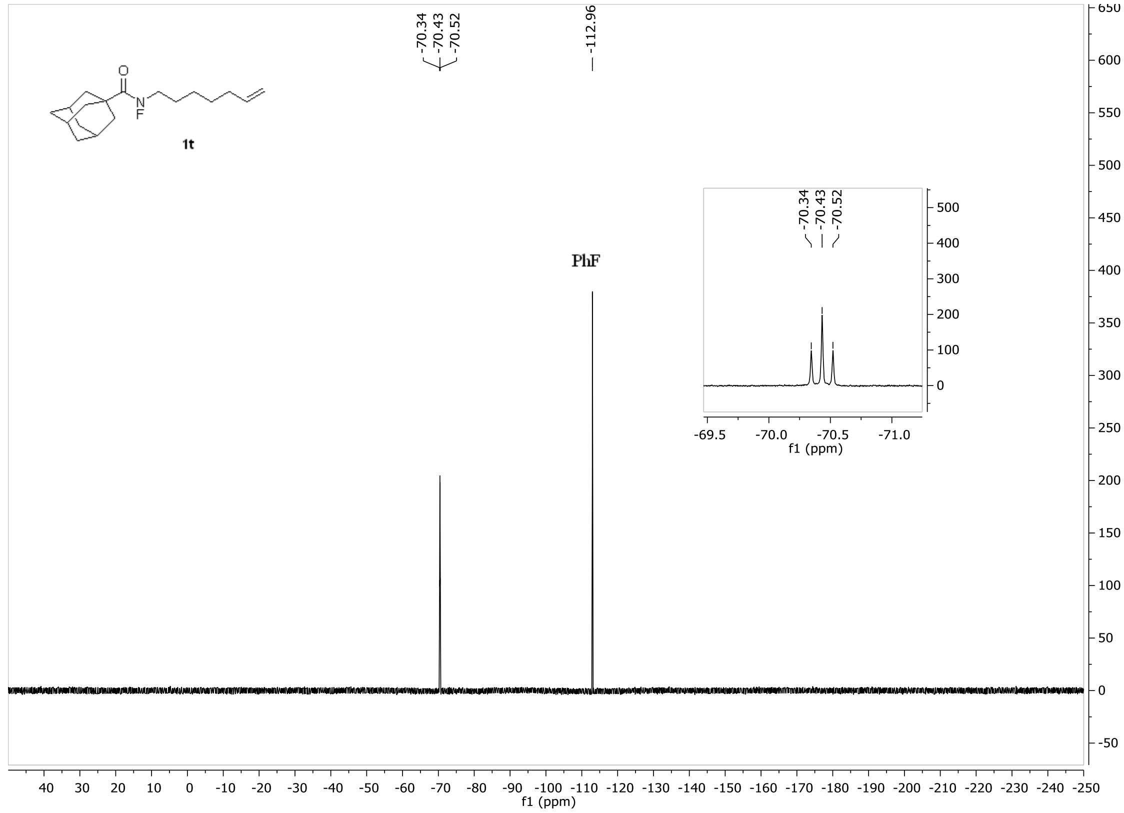

1t
PhF
-70.34
-70.43
-70.52
-112.96
f1 (ppm)

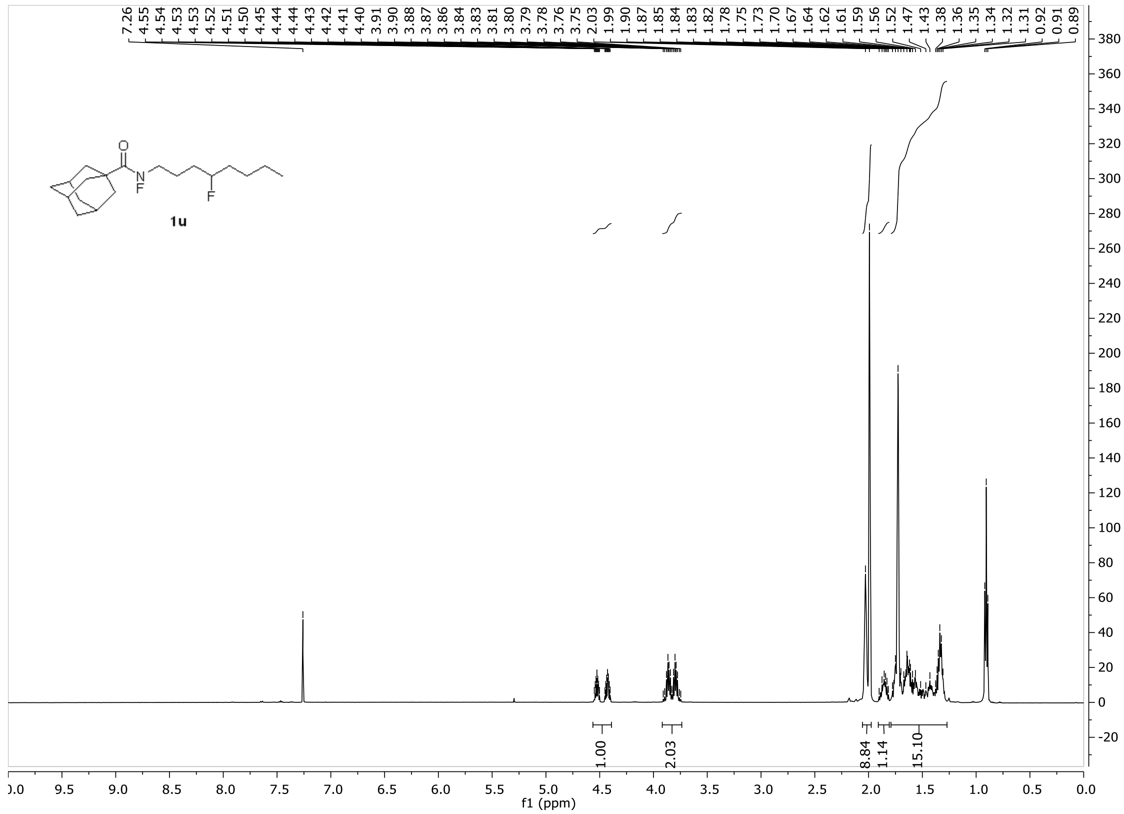

1u
1.00
2.03
8.84
1.14
15.10
f1 (ppm)

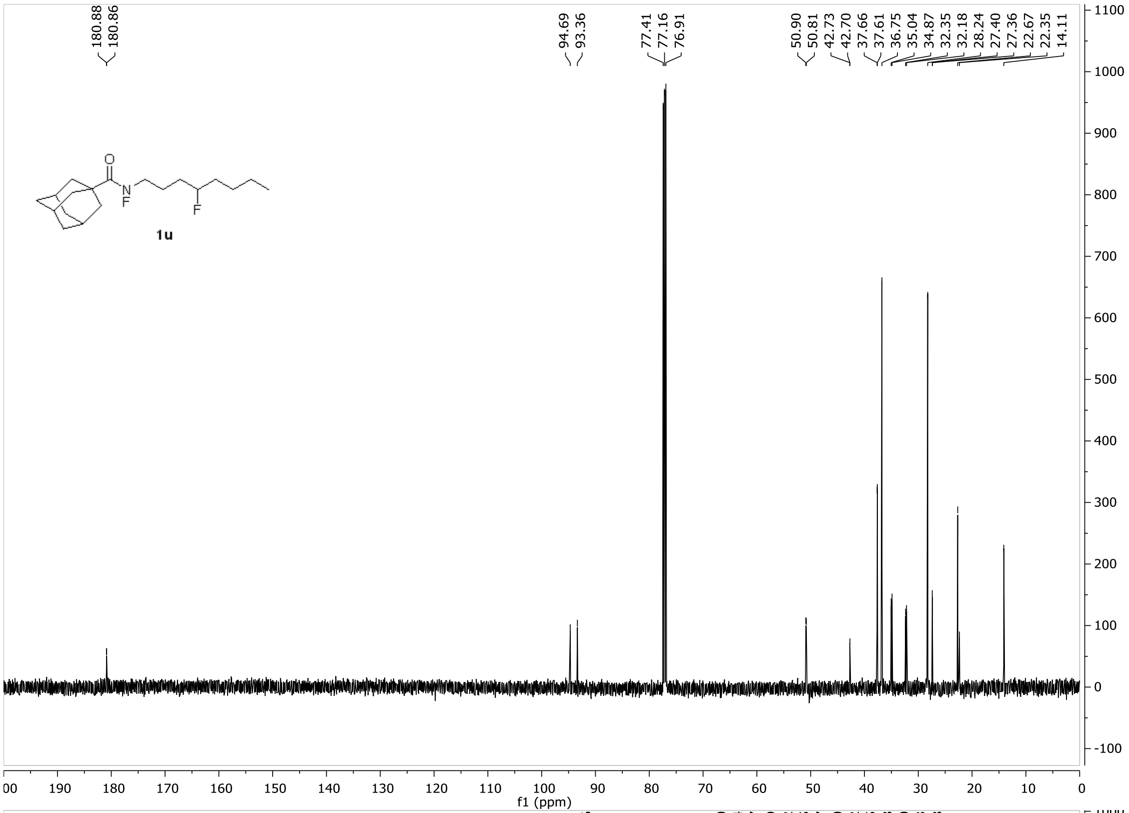

180.88
180.86
94.69
93.36
77.41
77.16
76.91
50.90
50.81
42.73
42.70
37.66
37.61
36.75
35.04
34.87
32.35
32.18
28.24
27.40
27.36
22.67
22.35
14.11
1u
f1 (ppm)

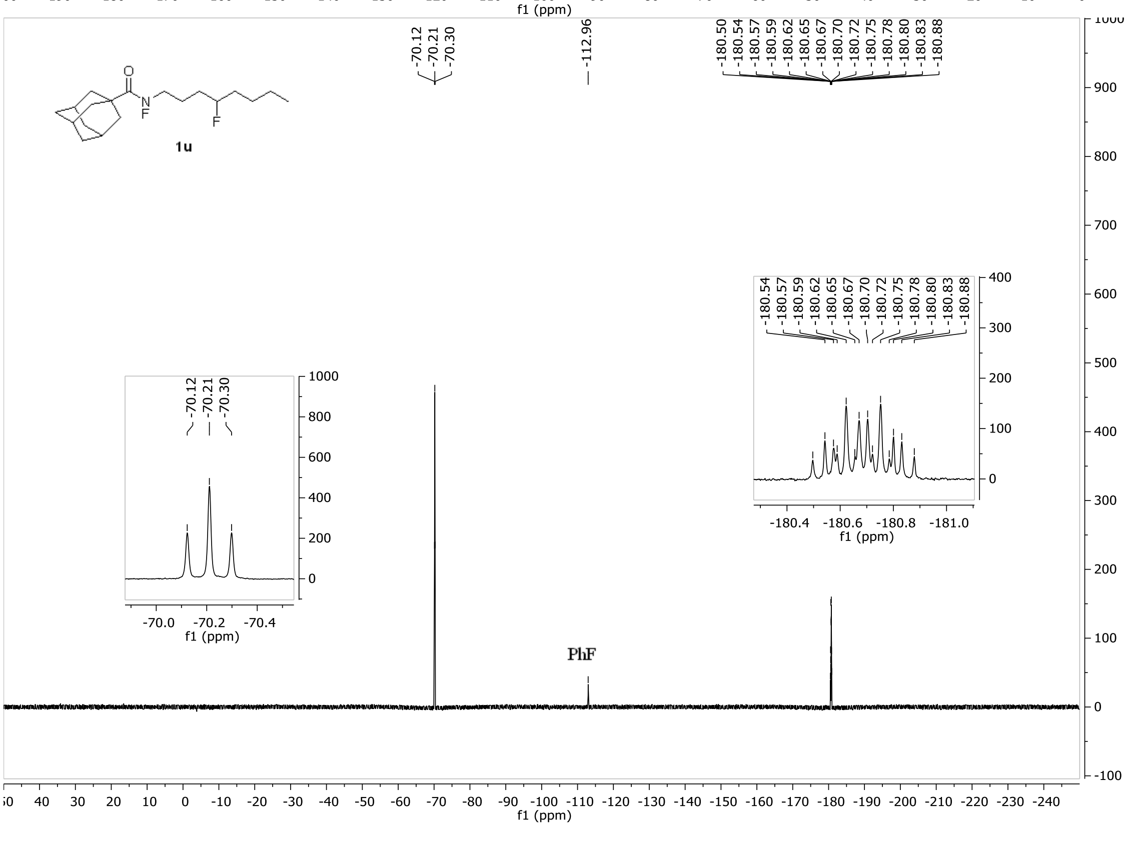

-70.12
-70.21
-70.30
-112.96
-180.50
-180.54
-180.57
-180.59
-180.62
-180.65
-180.67
-180.70
-180.72
-180.75
-180.78
-180.80
-180.83
-180.88
1u
PhF
f1 (ppm)

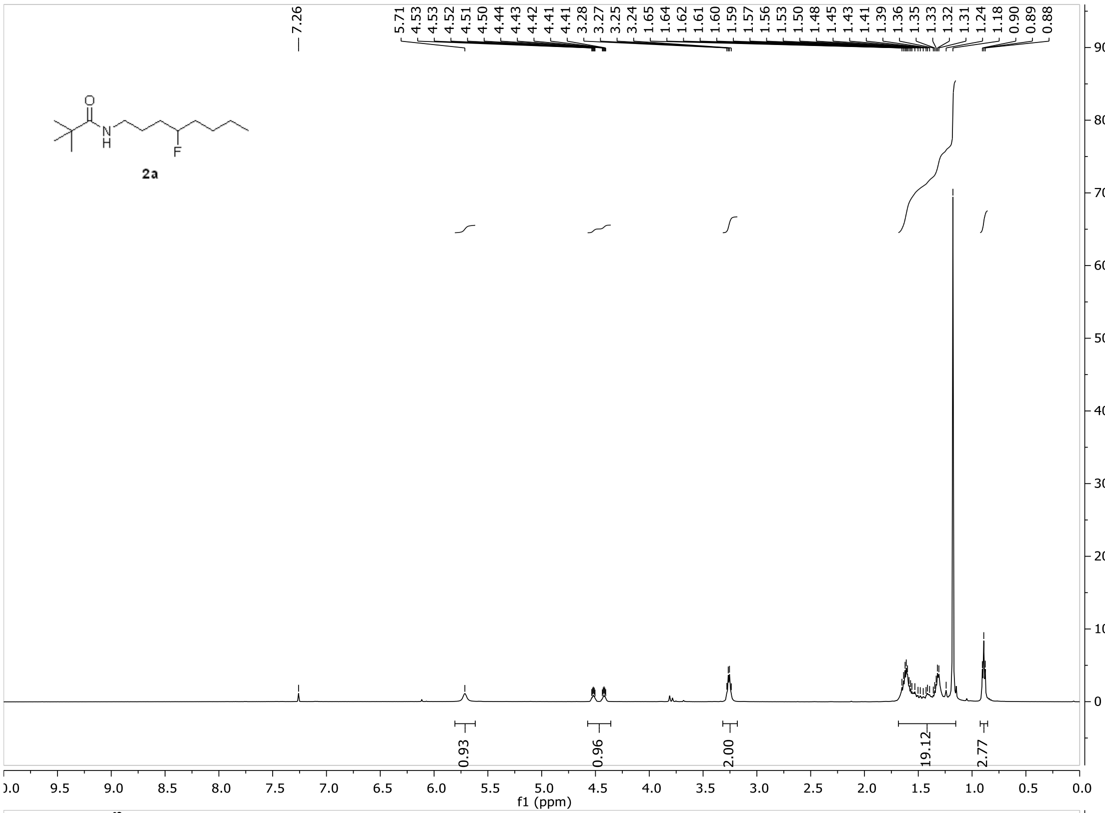

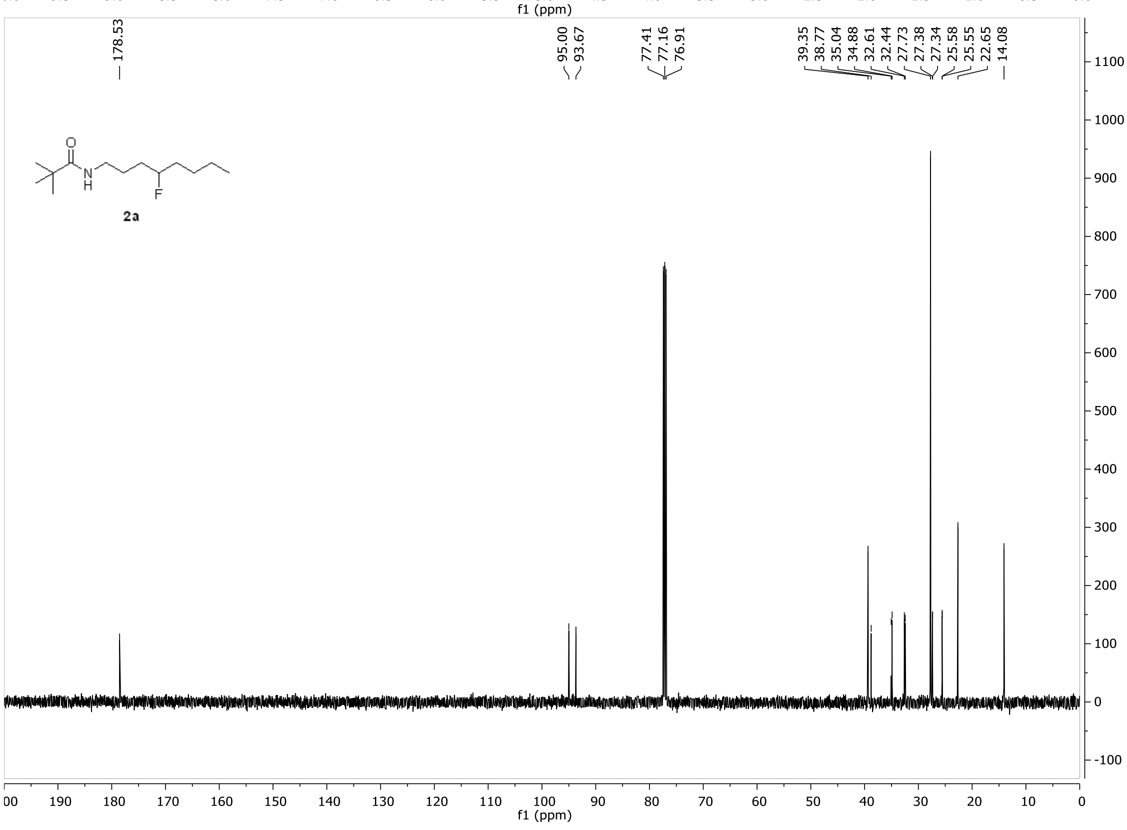

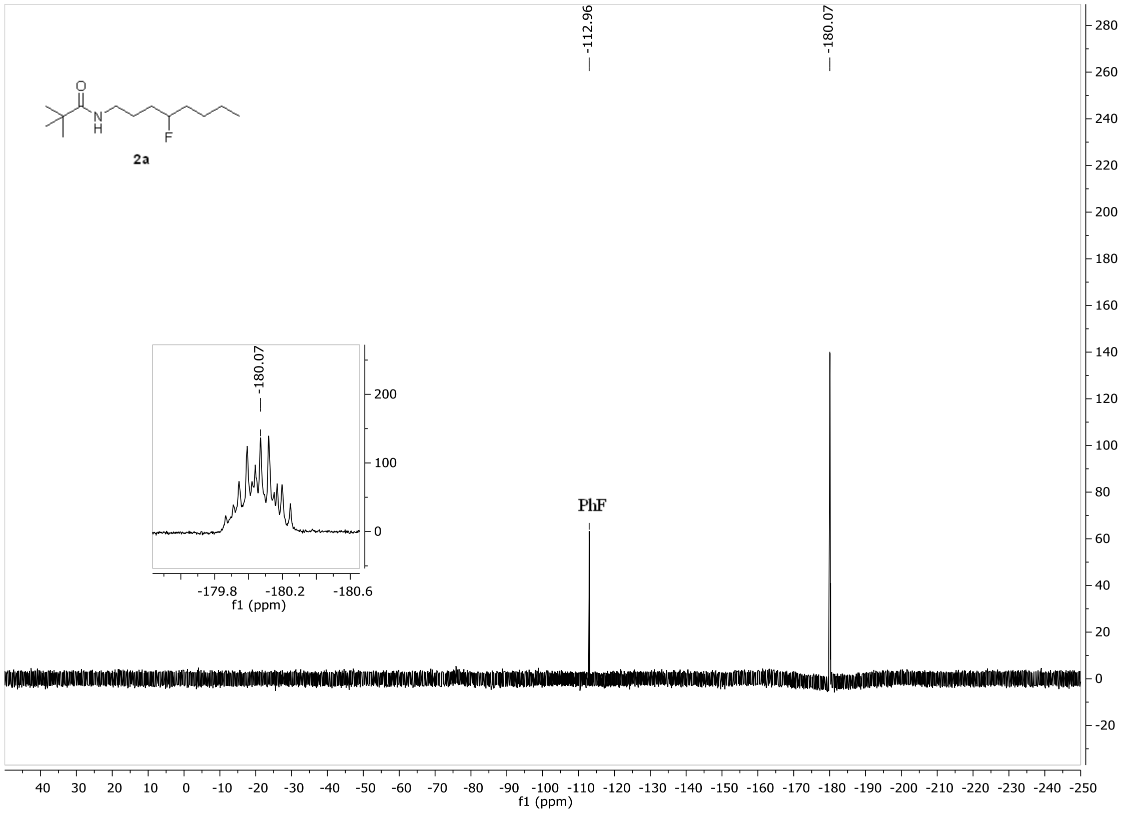
2a
PhF
-112.96
-180.07
f1 (ppm)

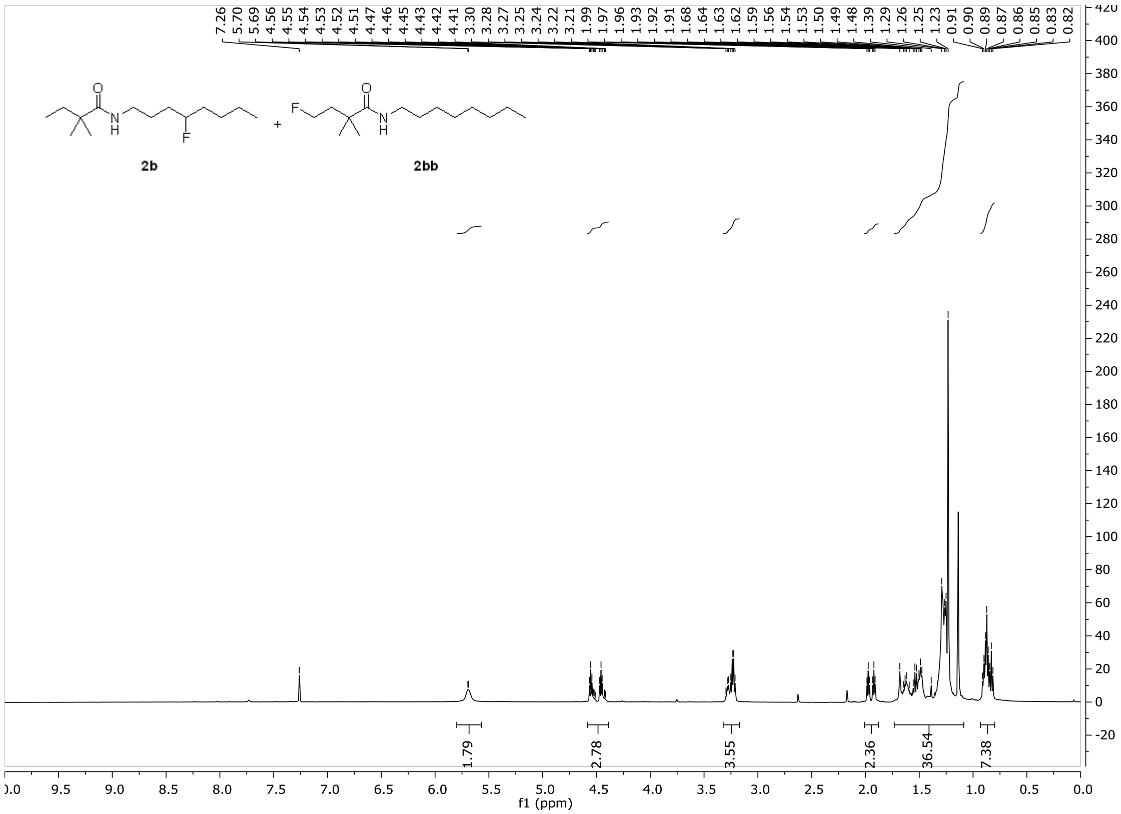
2b
2bb
f1 (ppm)
1.79
2.78
3.55
2.36
36.54
7.38

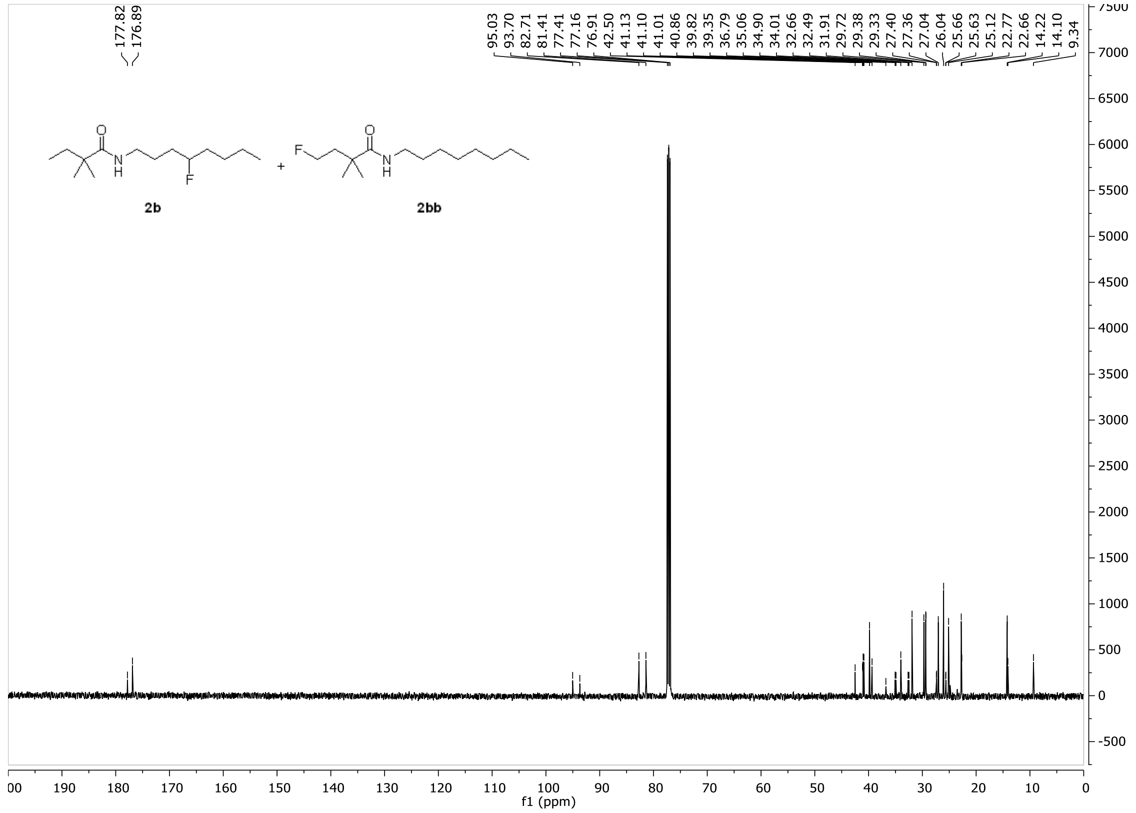
2b
2bb
f1 (ppm)

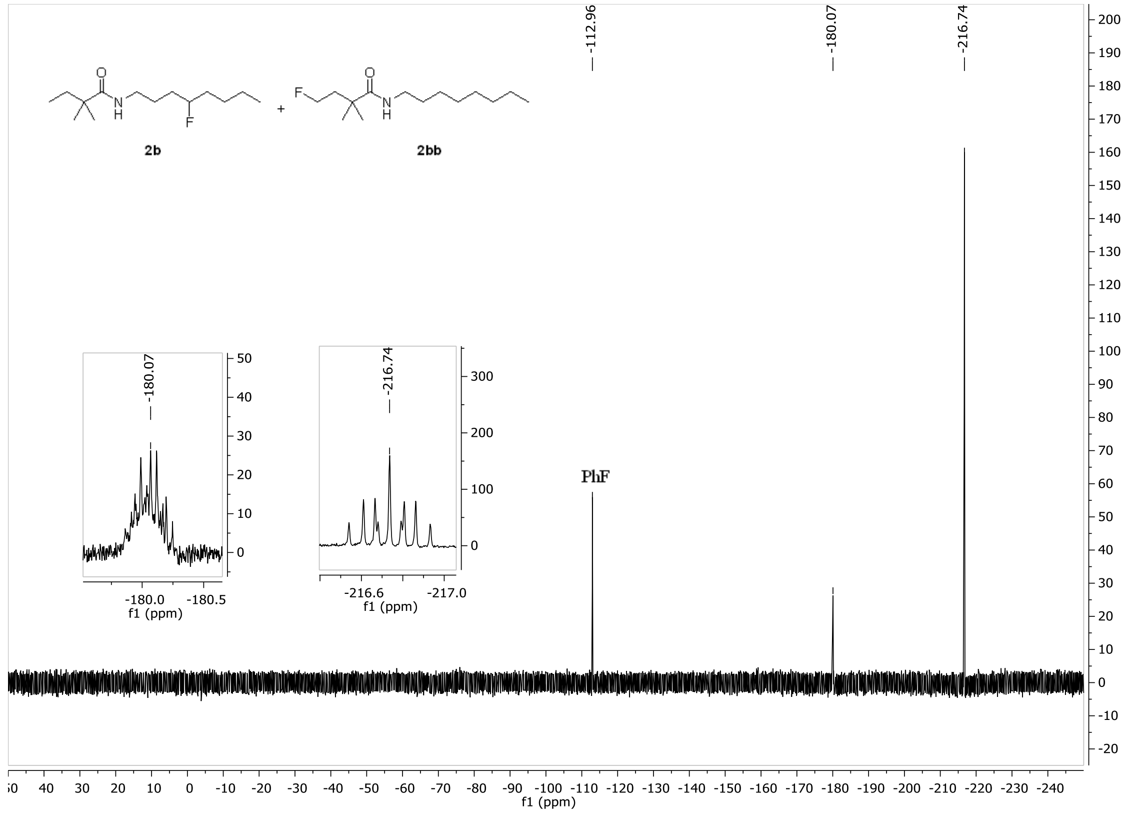
2b
2bb
PhF
-112.96
-180.07
-216.74
f1 (ppm)

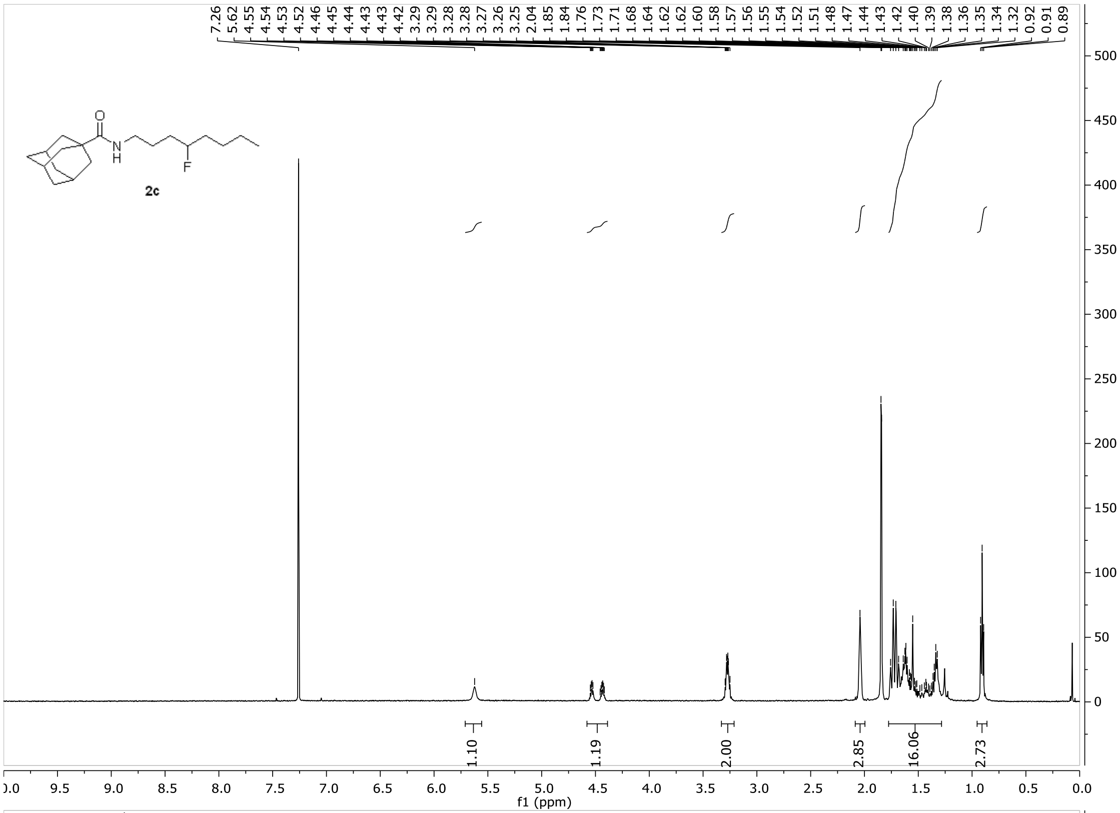
7.26 5.62 4.55 4.54 4.53 4.52 4.46 4.45 4.44 4.43 4.43 4.42 3.29 3.29 3.28 3.27 3.26 3.25 2.04 1.85 1.84 1.76 1.73 1.71 1.68 1.64 1.62 1.62 1.60 1.58 1.57 1.56 1.55 1.54 1.52 1.51 1.48 1.47 1.44 1.43 1.42 1.40 1.39 1.38 1.36 1.35 1.34 1.32 0.92 0.91 0.89
2c
1.10
1.19
2.00
2.85
16.06
2.73
f1 (ppm)

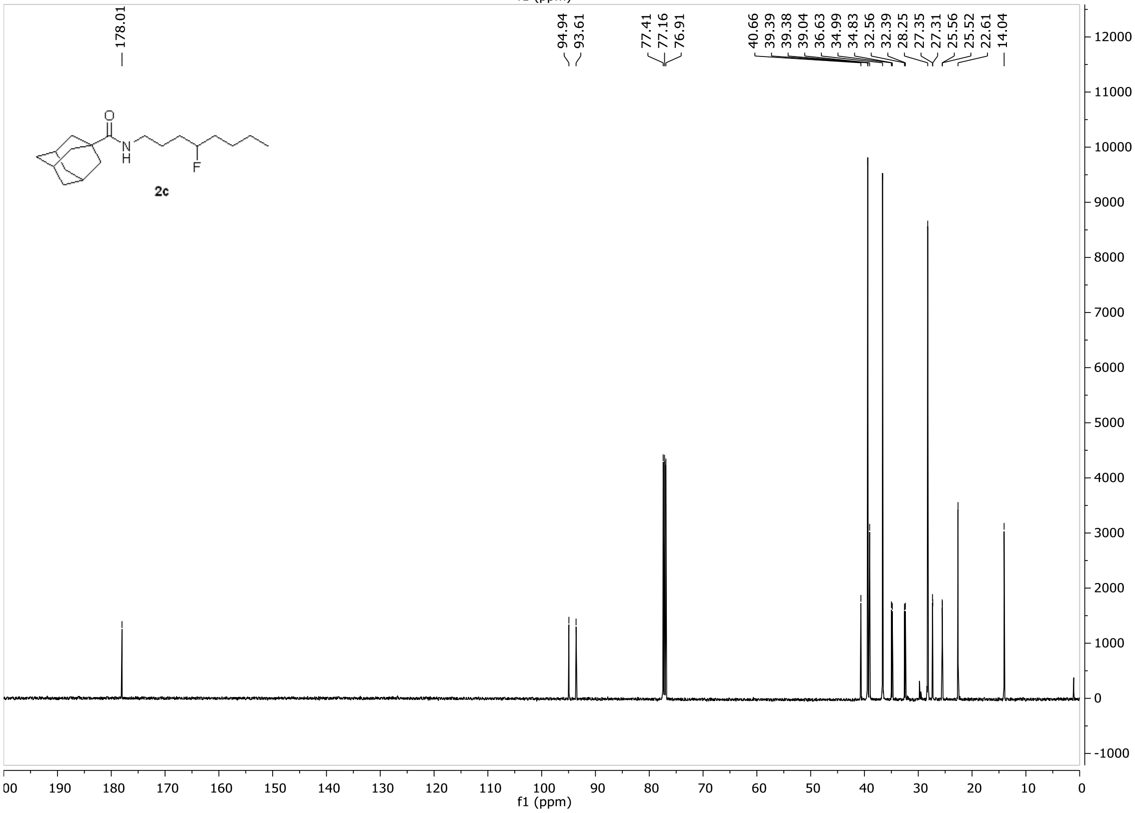
178.01
94.94 93.61
77.41 77.16 76.91
40.66 39.39 39.38 39.04 36.63 34.99 34.83 32.56 32.39 28.25 27.35 27.31 25.56 25.52 22.61
14.04
2c
f1 (ppm)

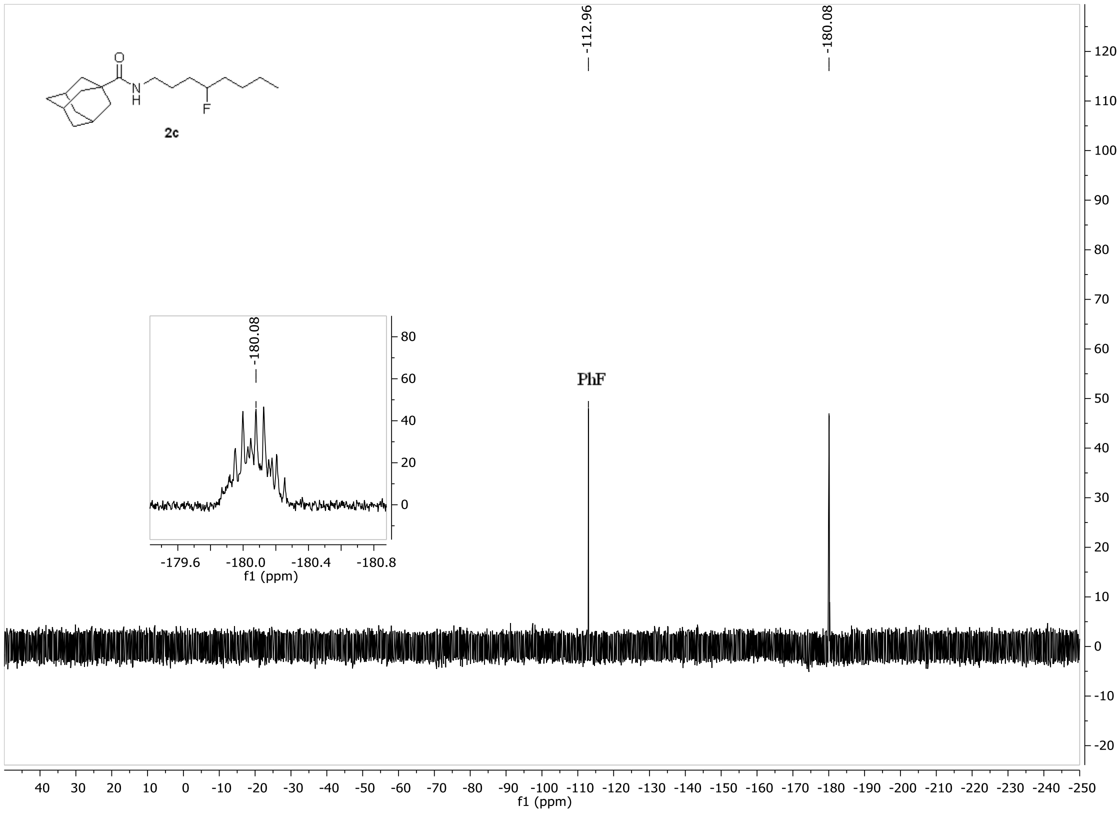
2c
PhF
-112.96
-180.08
f1 (ppm)

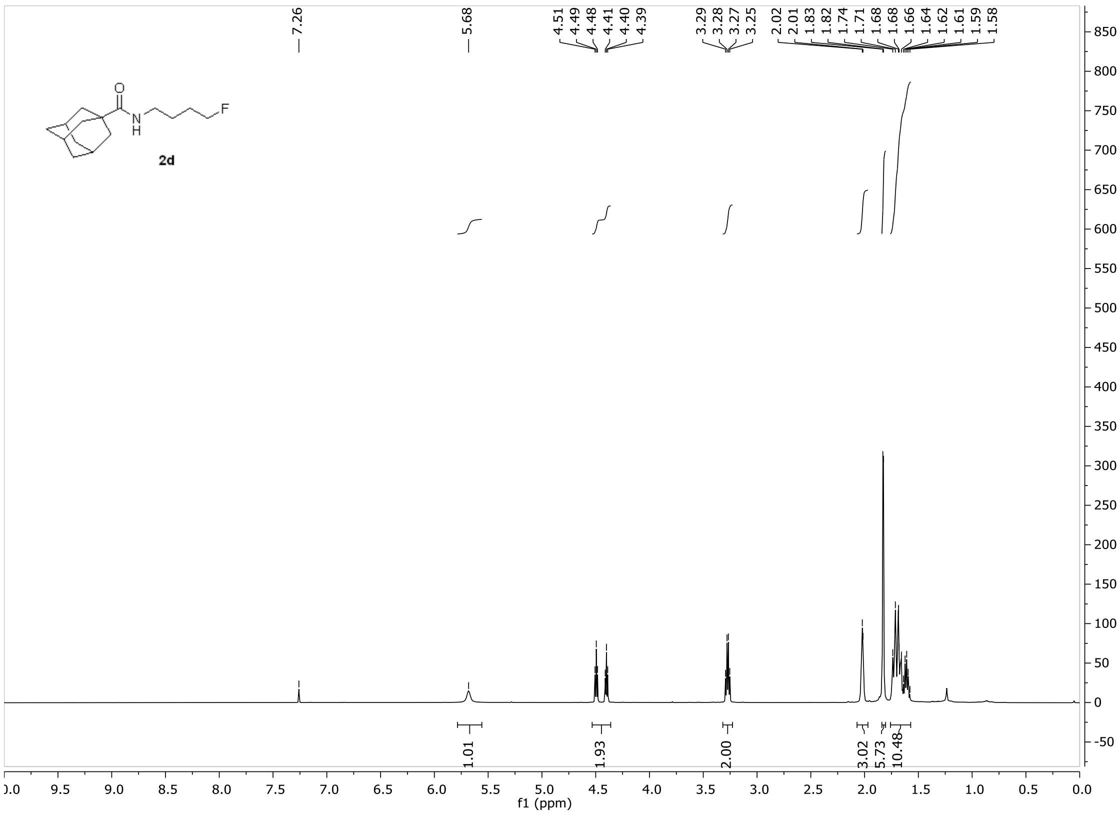
2d
7.26
5.68
f1 (ppm)

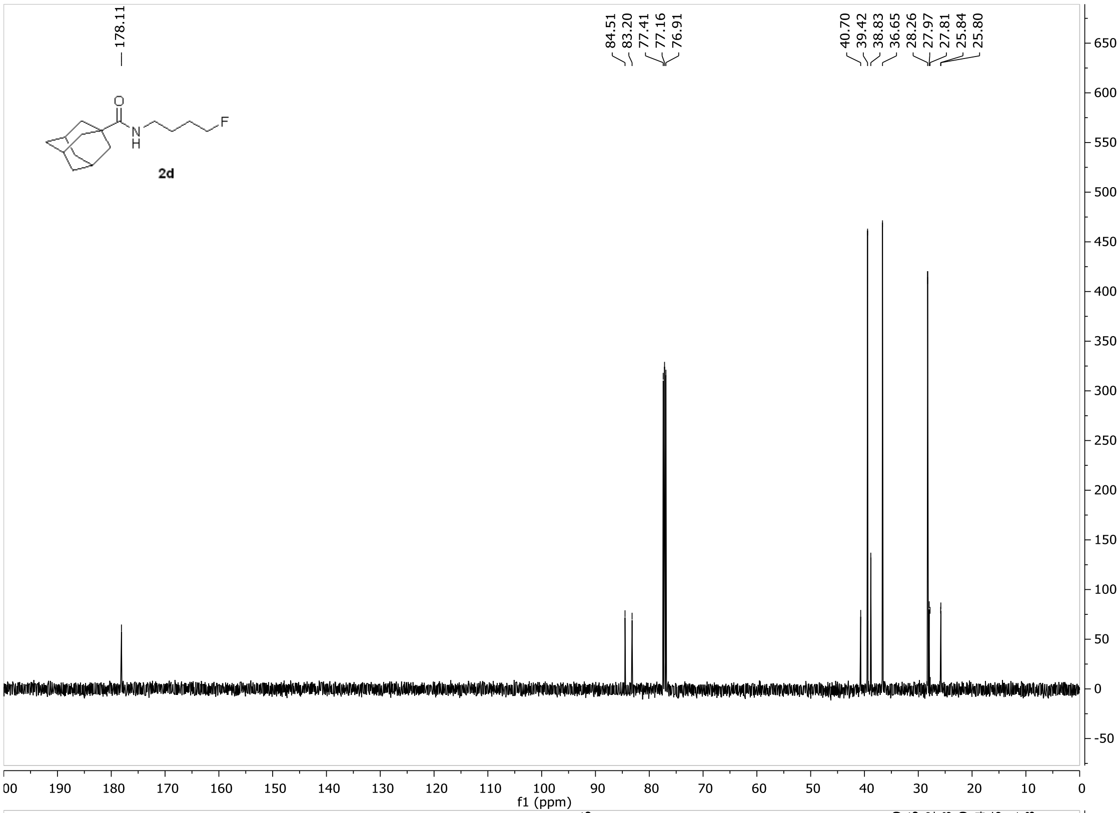
178.11
84.51
83.20
77.41
77.16
76.91
40.70
39.42
38.83
36.65
28.26
27.97
27.81
25.84
25.80
O
N
H
F
2d
f1 (ppm)

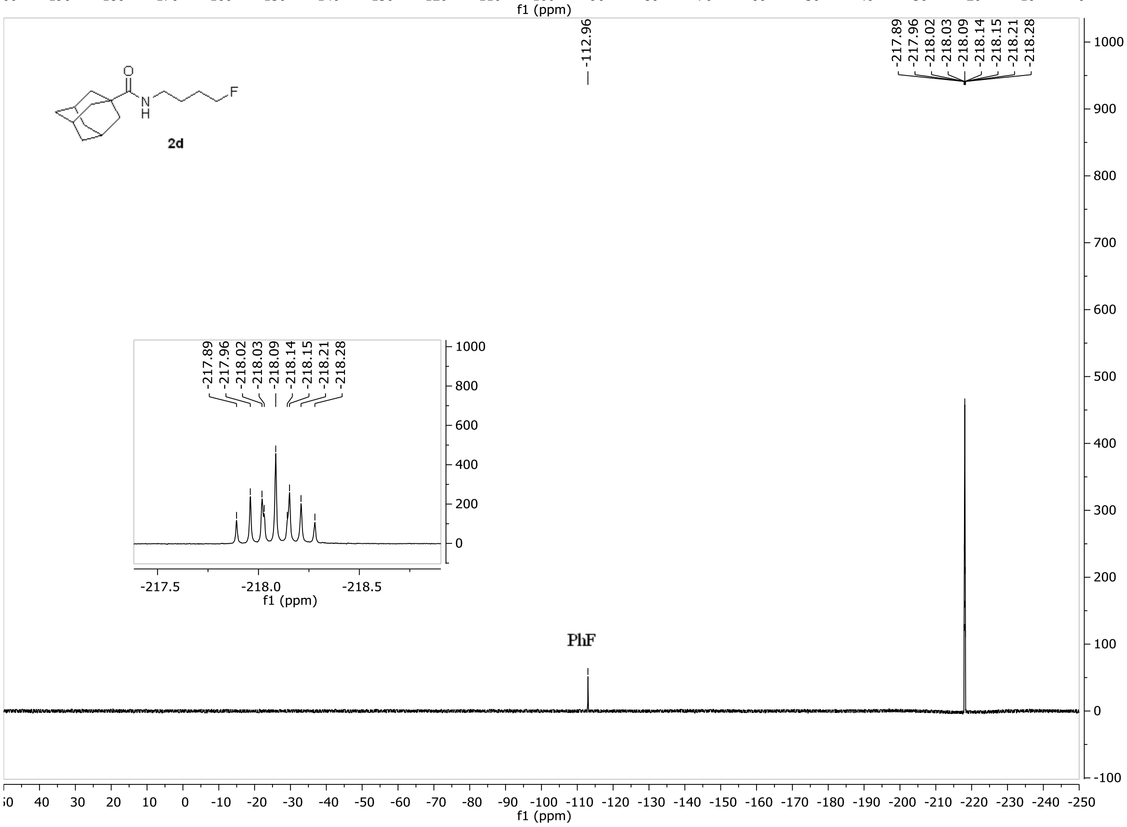
-112.96
-217.89
-217.96
-218.02
-218.03
-218.09
-218.14
-218.15
-218.21
-218.28
O
N
H
F
2d
PhF
f1 (ppm)

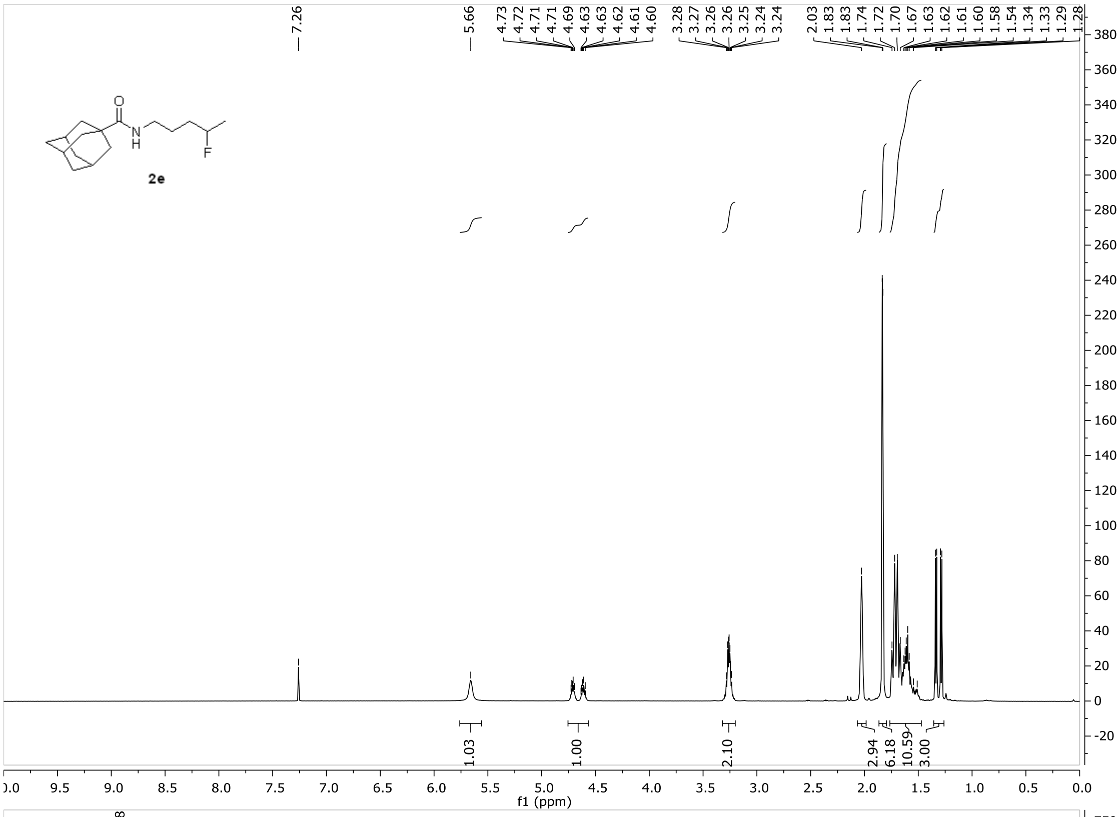
7.26
5.66
4.73
4.72
4.71
4.69
4.63
4.63
4.62
4.61
4.60
3.28
3.27
3.26
3.26
3.25
3.24
3.24
2.03
1.83
1.83
1.74
1.72
1.70
1.67
1.63
1.62
1.61
1.60
1.58
1.54
1.34
1.33
1.29
1.28
2e
1.03
1.00
2.10
2.94
6.18
10.59
3.00
f1 (ppm)

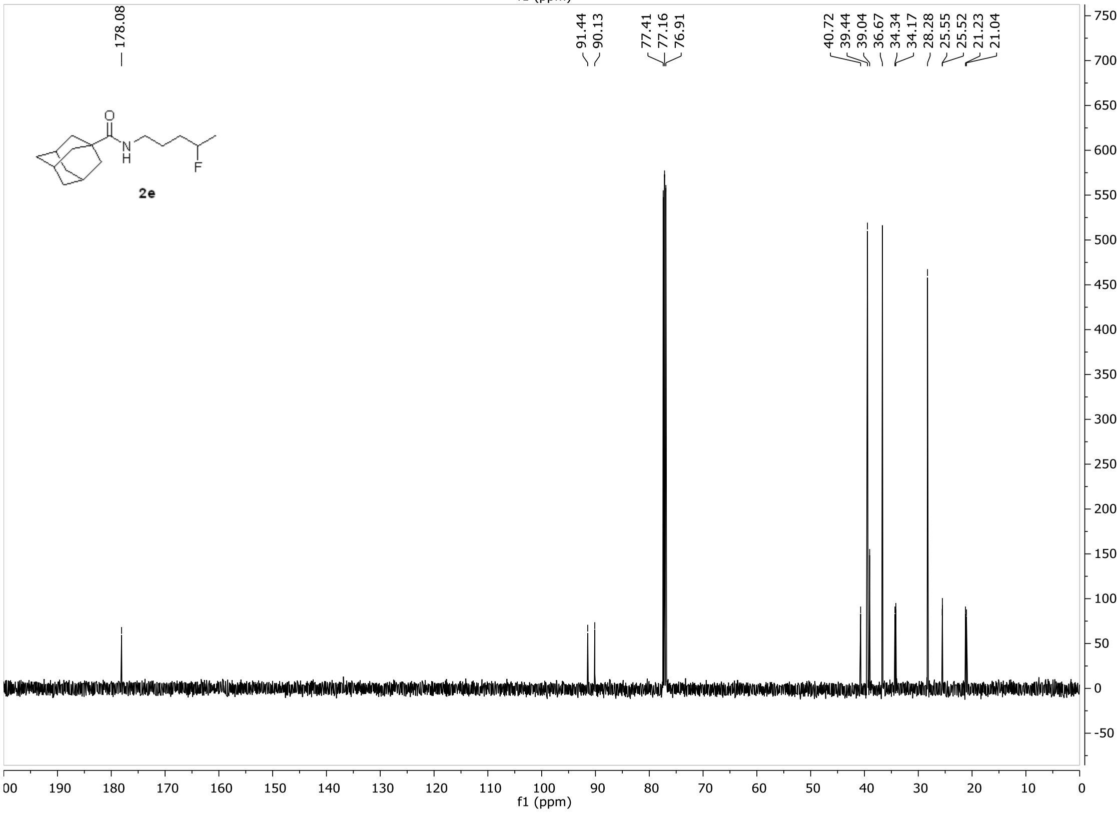
178.08
91.44
90.13
77.41
77.16
76.91
40.72
39.44
39.04
36.67
34.34
34.17
28.28
25.55
25.52
21.23
21.04
2e
f1 (ppm)

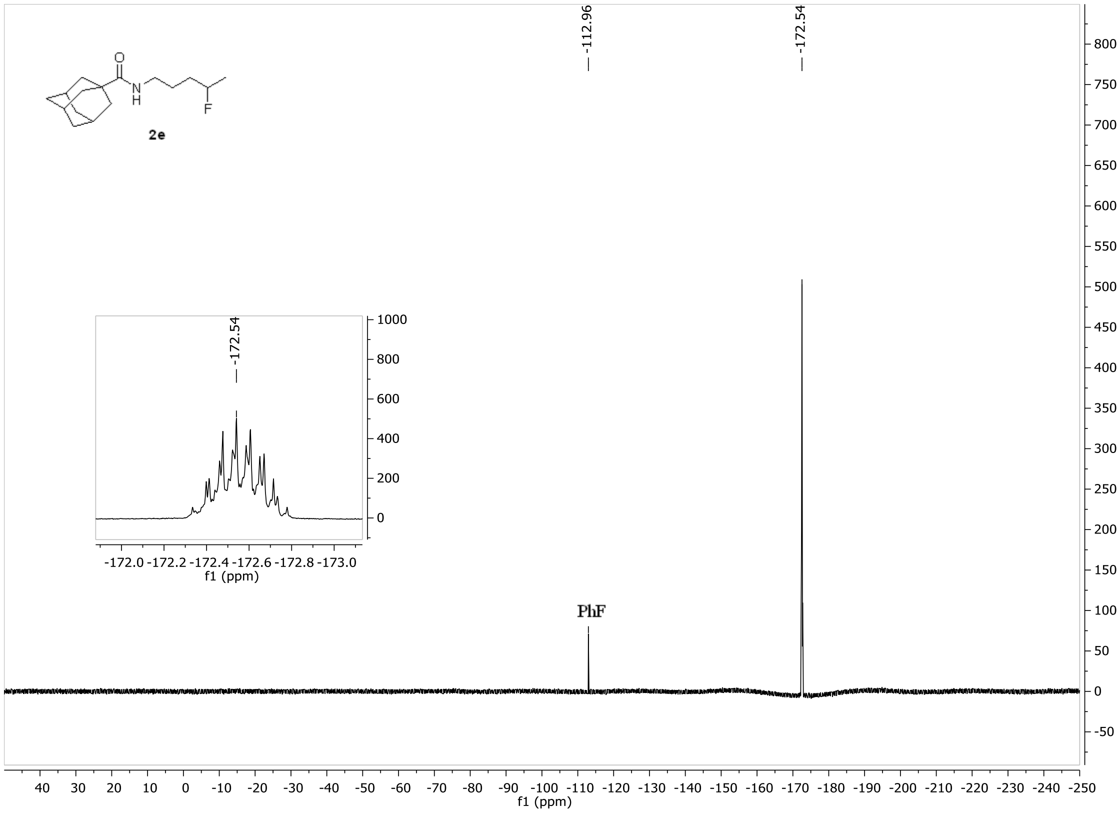
2e
-112.96
-172.54
PhF
f1 (ppm)

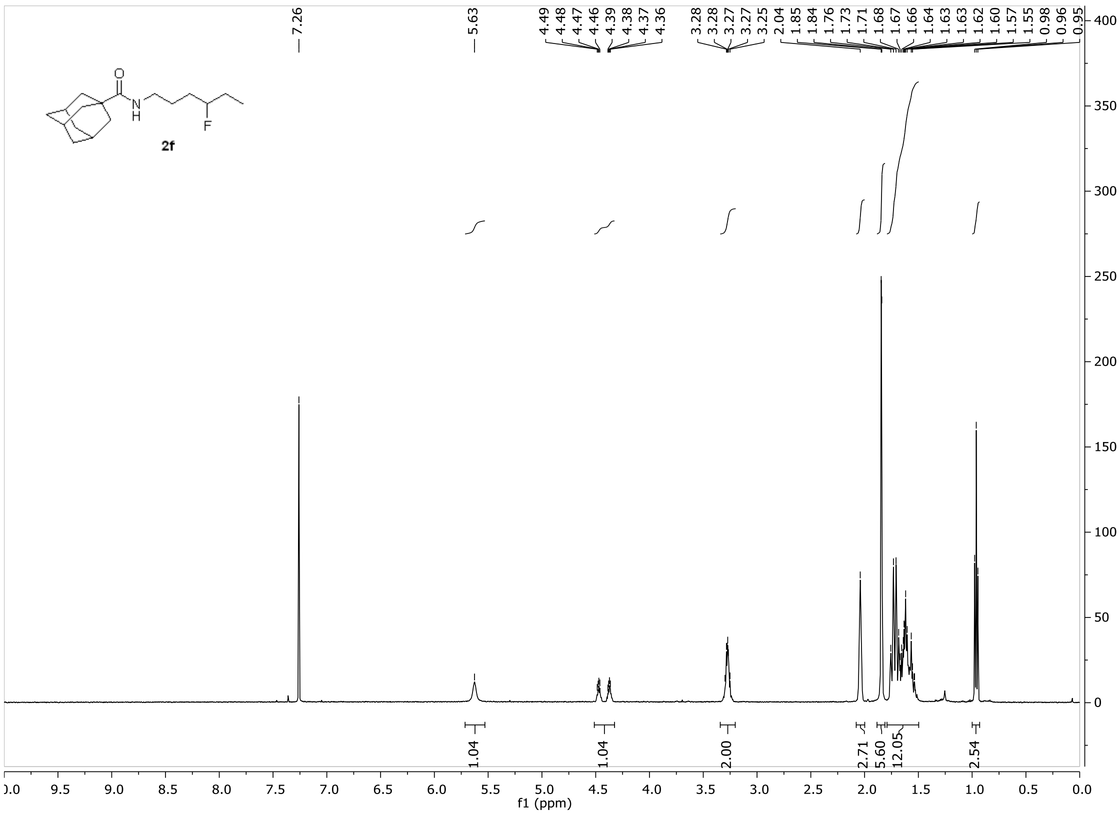
2f
7.26
5.63
4.49 4.48 4.47 4.46 4.39 4.38 4.37 4.36
3.28 3.28 3.27 3.27 3.25
2.04 1.85 1.84 1.76 1.73 1.71 1.68 1.67 1.66 1.64 1.63 1.63 1.62 1.60 1.57 1.55 0.98 0.96 0.95
1.04
1.04
2.00
2.71
5.60
12.05
2.54
f1 (ppm)

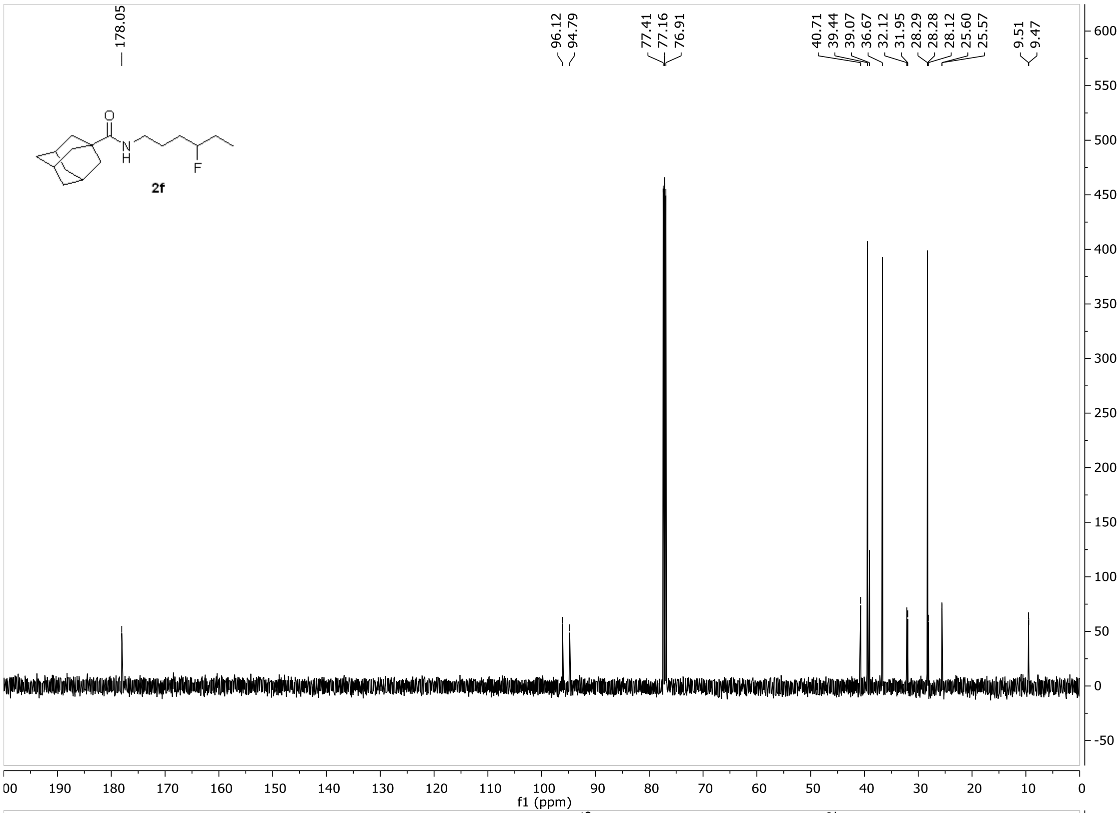
178.05
96.12
94.79
77.41
77.16
76.91
40.71
39.44
39.07
36.67
32.12
31.95
28.29
28.28
28.12
25.60
25.57
9.51
9.47
O
N
H
F
2f
f1 (ppm)

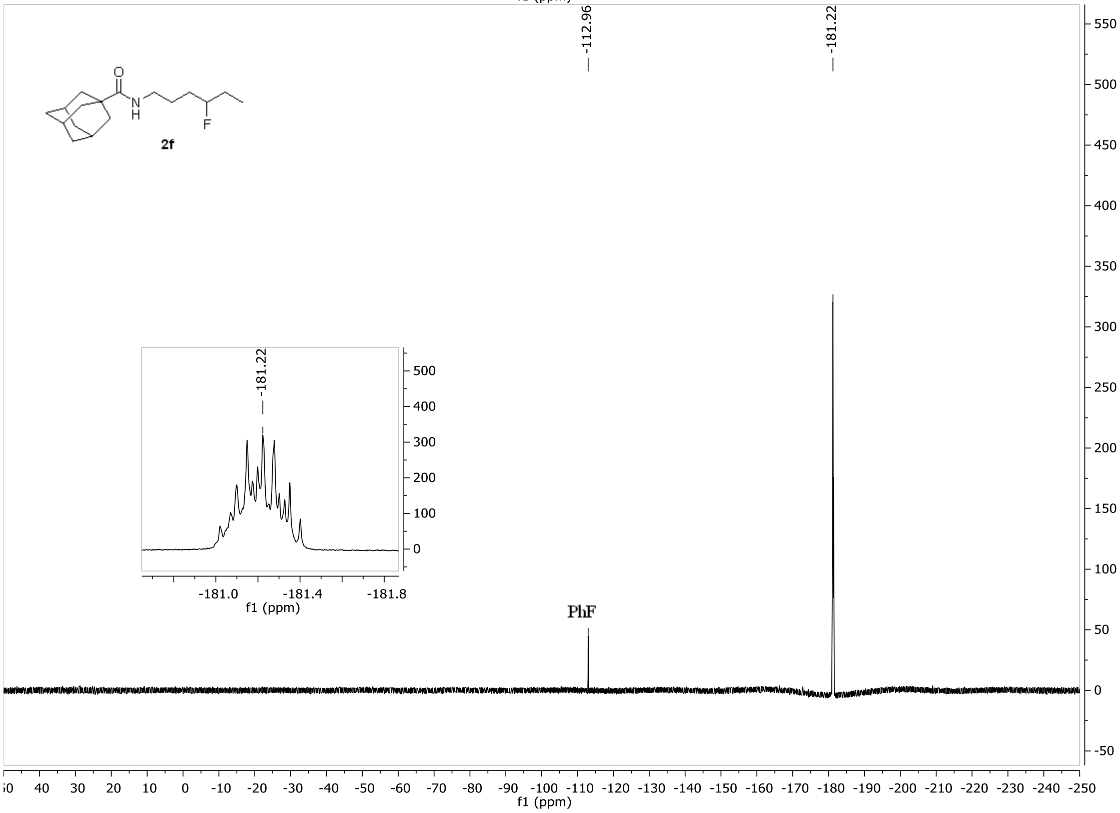
-112.96
-181.22
O
N
H
F
2f
PhF
f1 (ppm)

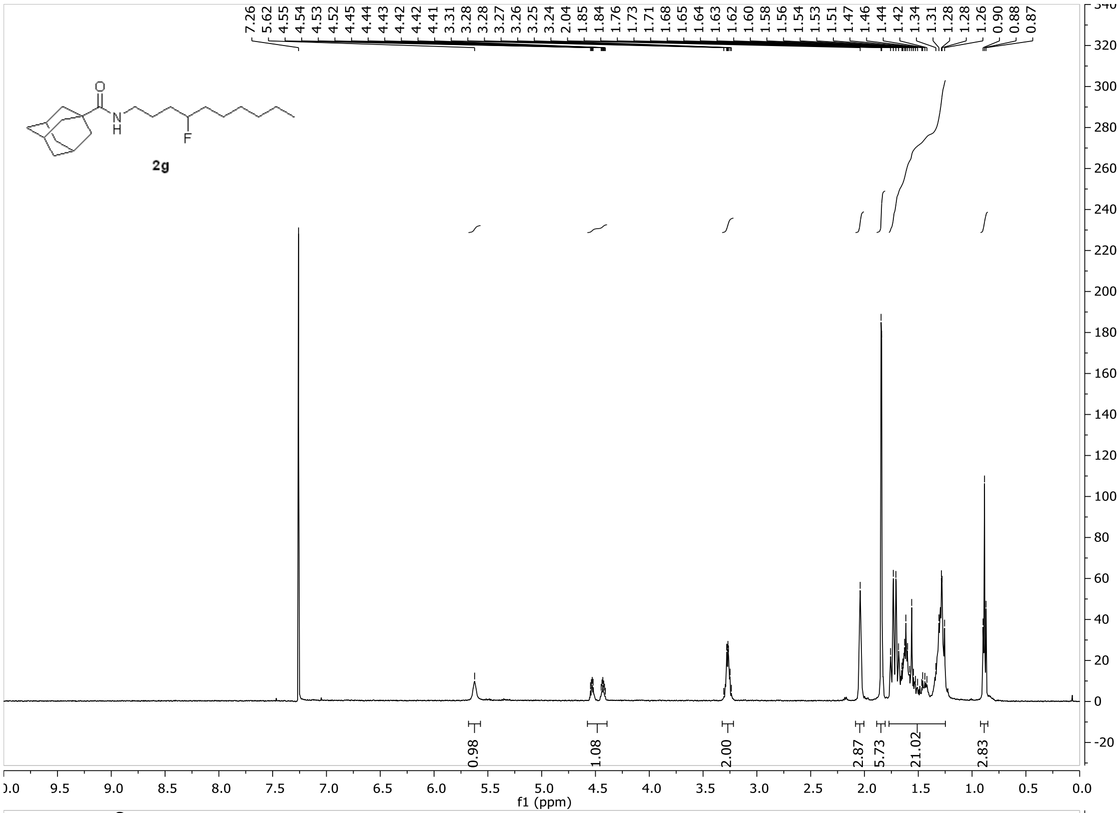
7.26
5.62
4.55
4.54
4.53
4.52
4.45
4.44
4.43
4.42
4.42
4.41
3.31
3.28
3.28
3.27
3.26
3.25
3.24
2.04
1.85
1.84
1.76
1.73
1.71
1.68
1.65
1.64
1.63
1.62
1.60
1.58
1.56
1.54
1.53
1.51
1.47
1.46
1.44
1.42
1.34
1.31
1.28
1.28
1.26
0.90
0.88
0.87
O
N
H
F
2g
0.98
1.08
2.00
2.87
5.73
21.02
2.83
f1 (ppm)

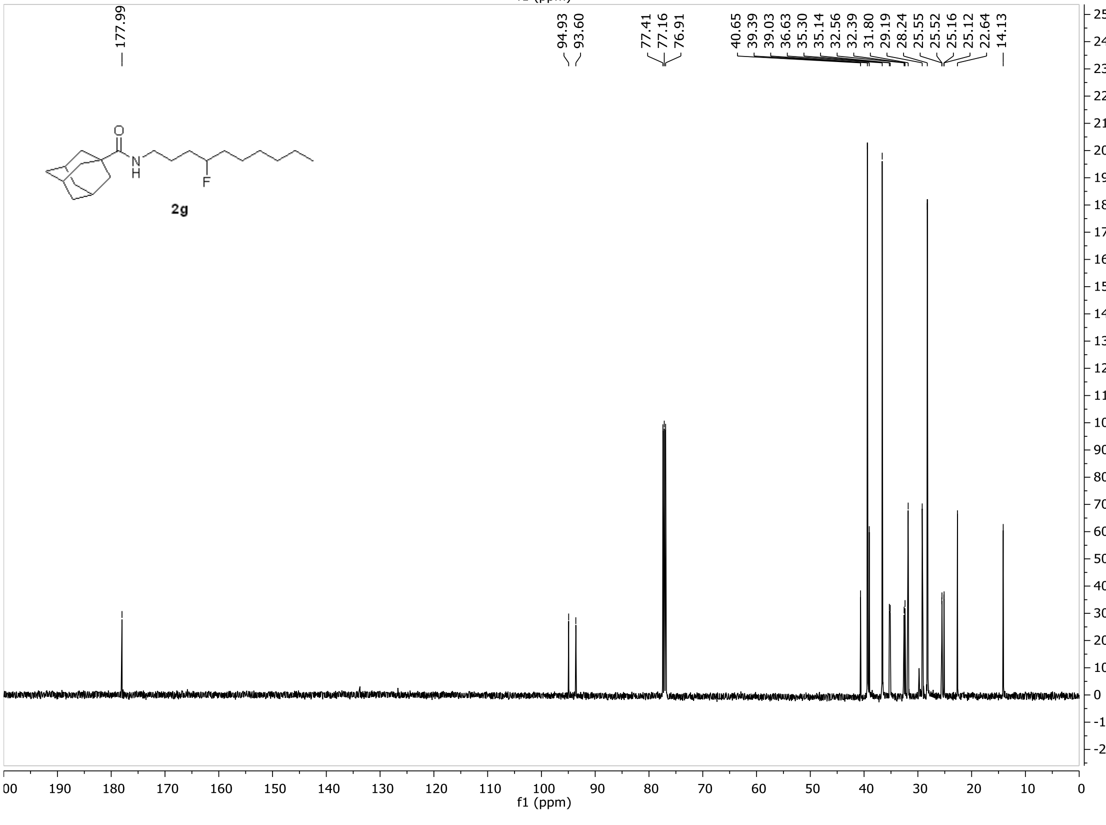
177.99
94.93
93.60
77.41
77.16
76.91
40.65
39.39
39.03
36.63
35.30
35.14
32.56
32.39
31.80
29.19
28.24
25.55
25.52
25.16
25.12
22.64
14.13
O
N
H
F
2g
f1 (ppm)

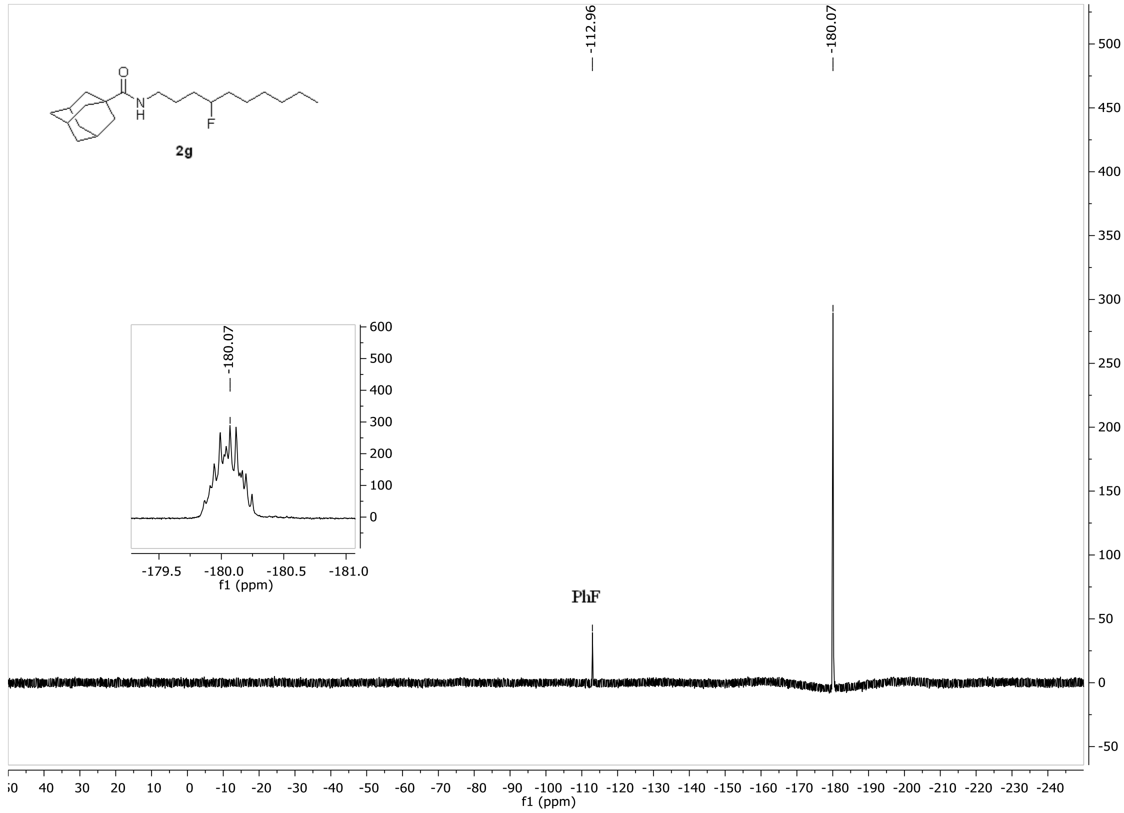
2g
-112.96
-180.07
PhF
f1 (ppm)

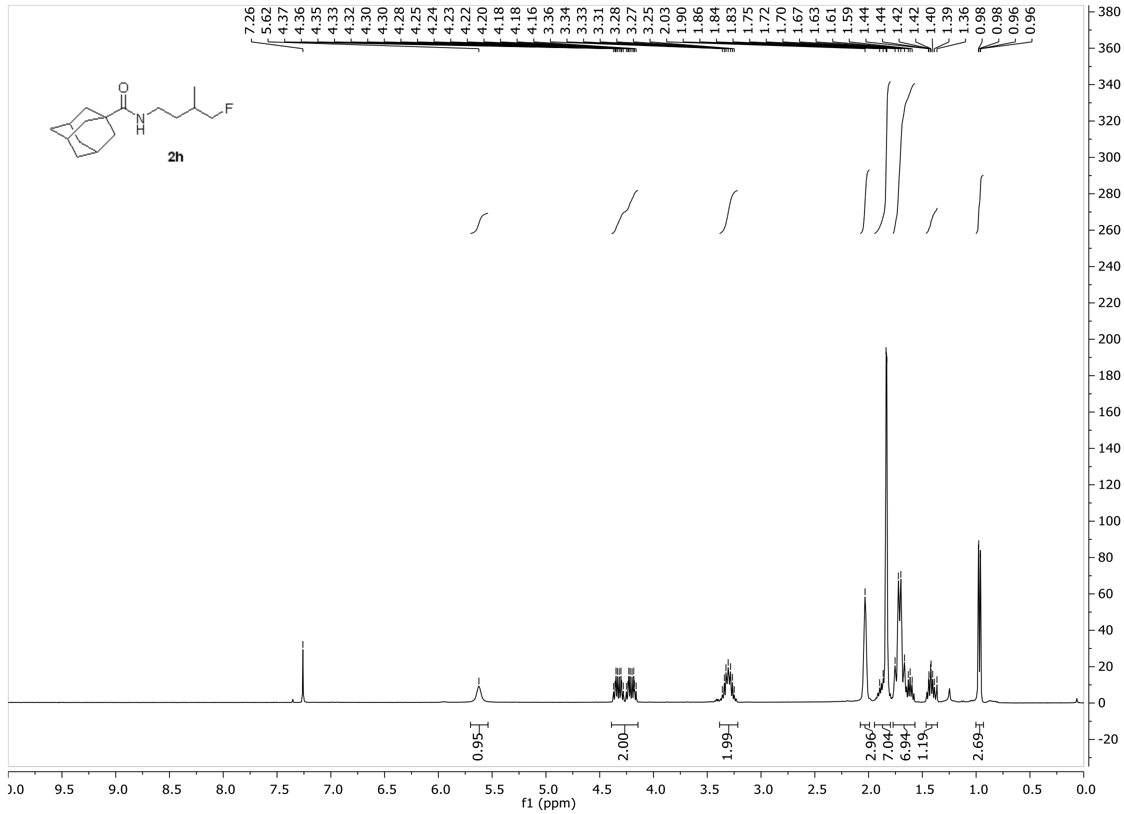
2h
f1 (ppm)

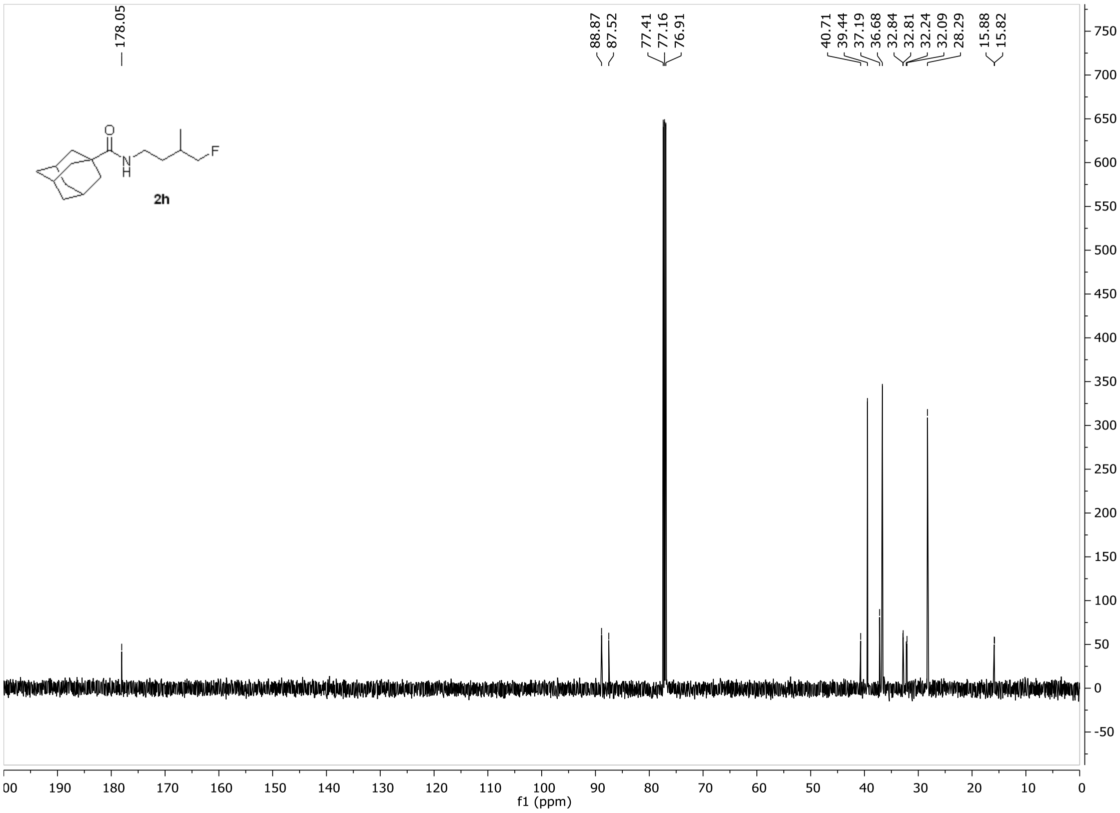
178.05
88.87
87.52
77.41
77.16
76.91
40.71
39.44
37.19
36.68
32.84
32.81
32.24
32.09
28.29
15.88
15.82
O
N
H
F
2h
f1 (ppm)

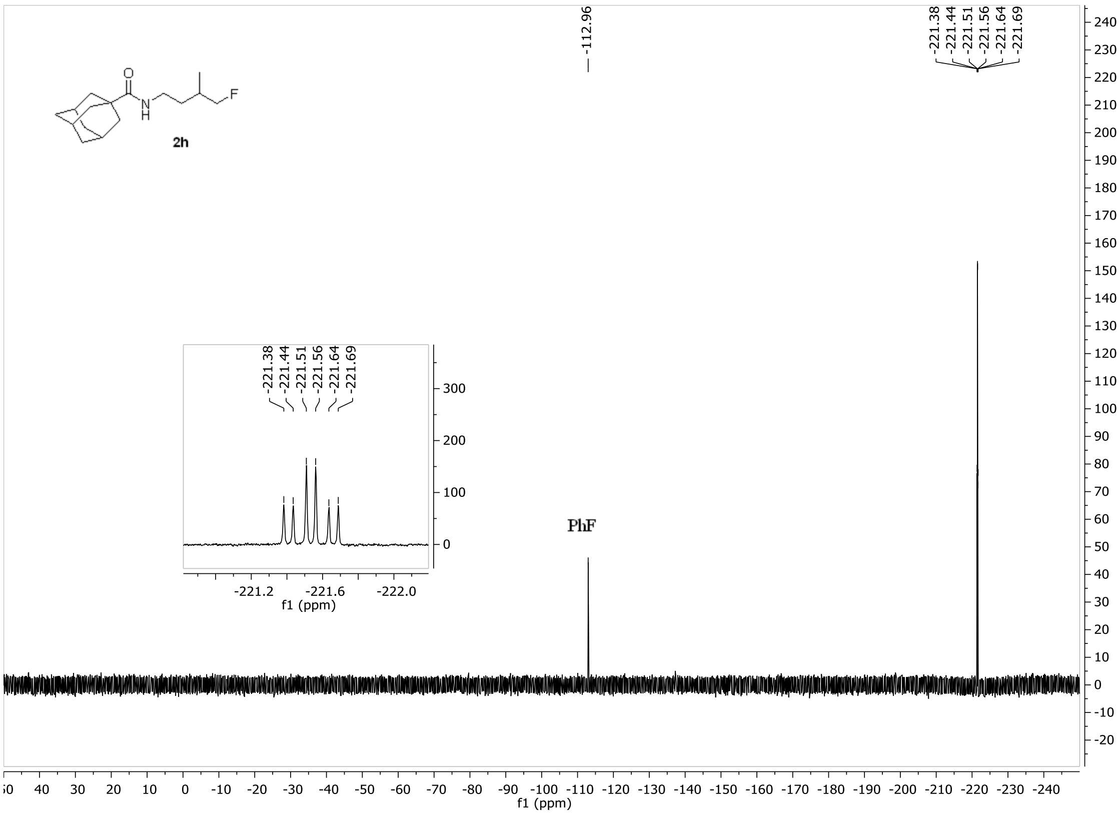
-112.96
-221.38
-221.44
-221.51
-221.56
-221.64
-221.69
O
N
H
F
2h
PhF
f1 (ppm)

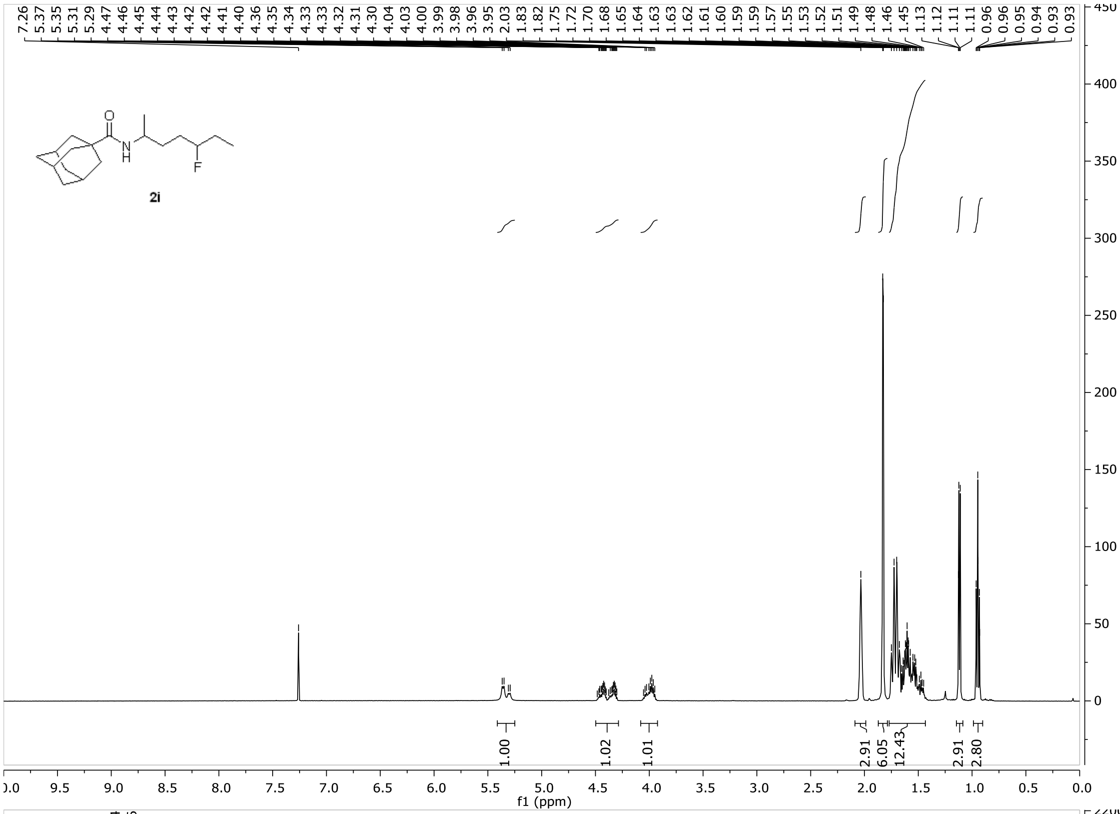

7.26 5.37 5.35 5.31 5.29 4.47 4.46 4.45 4.44 4.43 4.42 4.42 4.41 4.40 4.36 4.35 4.34 4.33 4.33 4.32 4.31 4.30 4.04 4.03 4.00 3.99 3.98 3.96 3.95 2.03 1.83 1.82 1.75 1.70 1.68 1.65 1.64 1.63 1.63 1.62 1.61 1.60 1.59 1.59 1.57 1.55 1.53 1.52 1.51 1.49 1.48 1.46 1.45 1.13 1.12 1.11 1.11 0.96 0.96 0.95 0.94 0.93 0.93
O
N
H
F
2i
1.00
1.02
1.01
2.91
6.05
12.43
2.91
2.80
f1 (ppm)

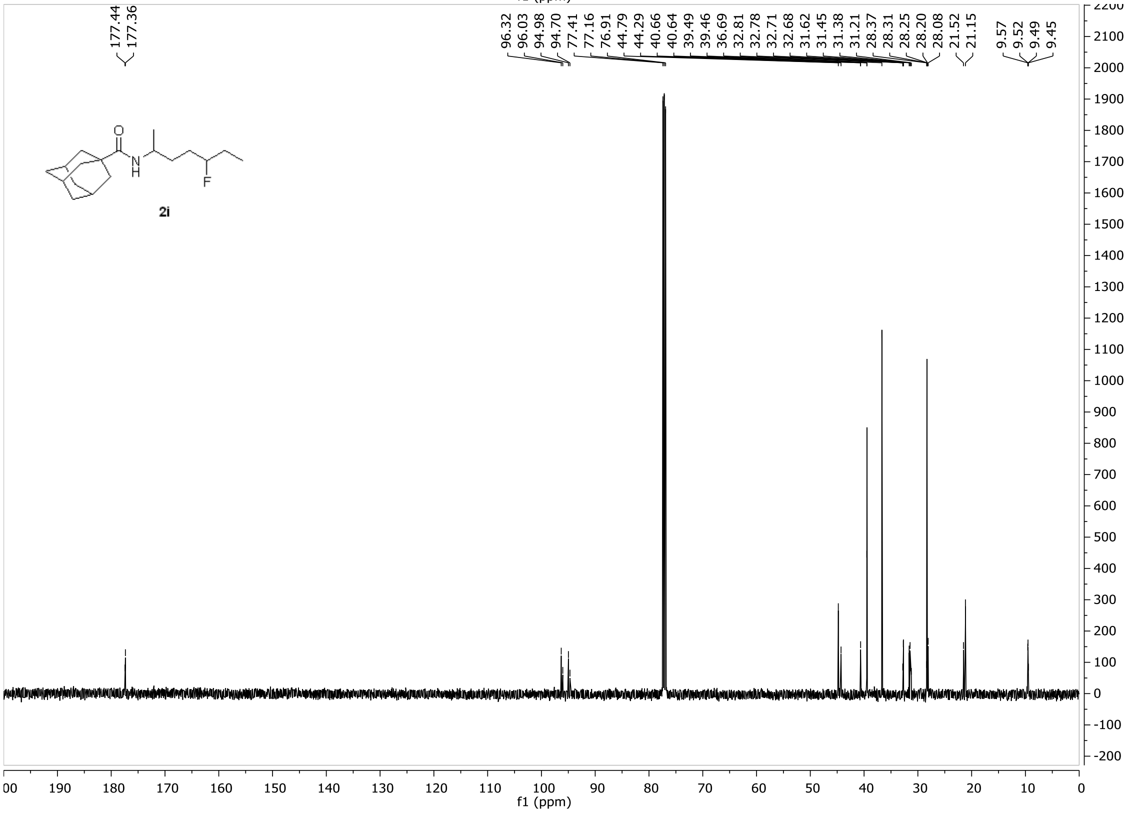

177.44 177.36
96.32 96.03 94.98 94.70 77.41 77.16 76.91 44.79 44.29 40.66 40.64 39.49 39.46 36.69 32.81 32.78 32.71 32.68 31.62 31.45 31.38 31.21 28.37 28.31 28.25 28.20 28.08 21.52 21.15
9.57 9.52 9.49 9.45
O
N
H
F
2i
f1 (ppm)

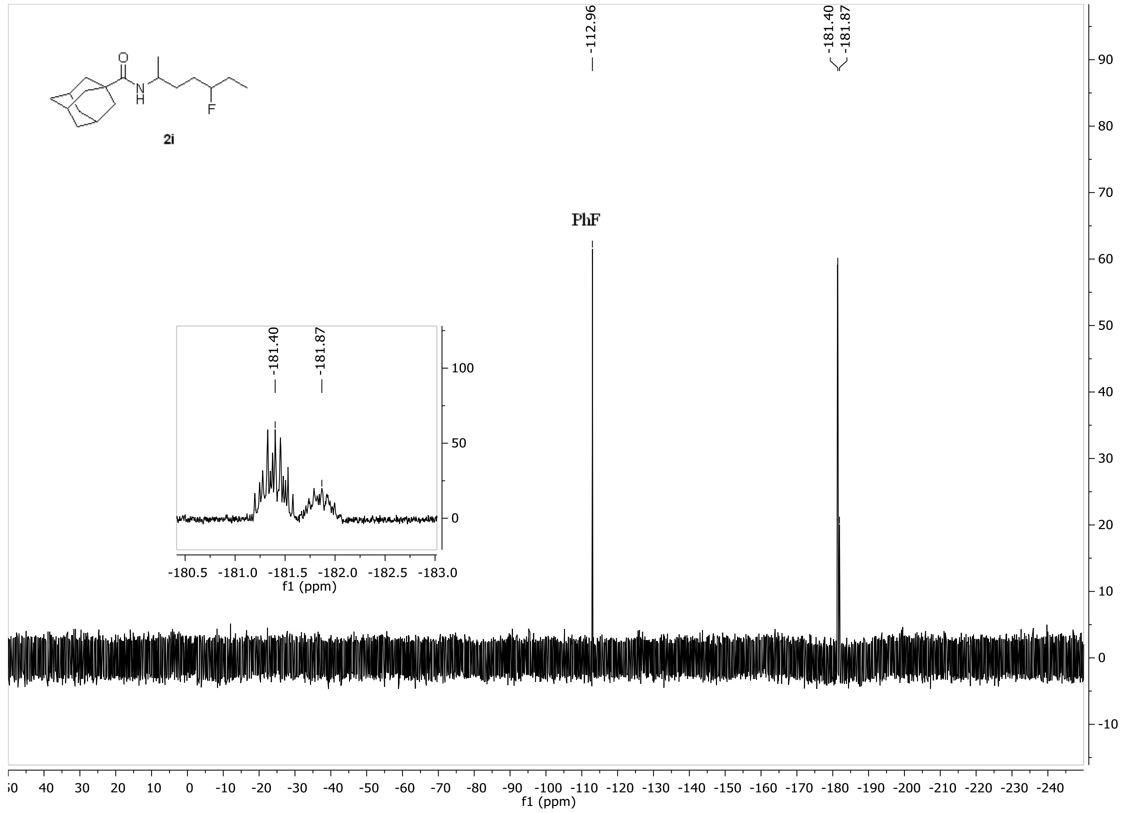
2i
PhF
-112.96
-181.40
-181.87
f1 (ppm)

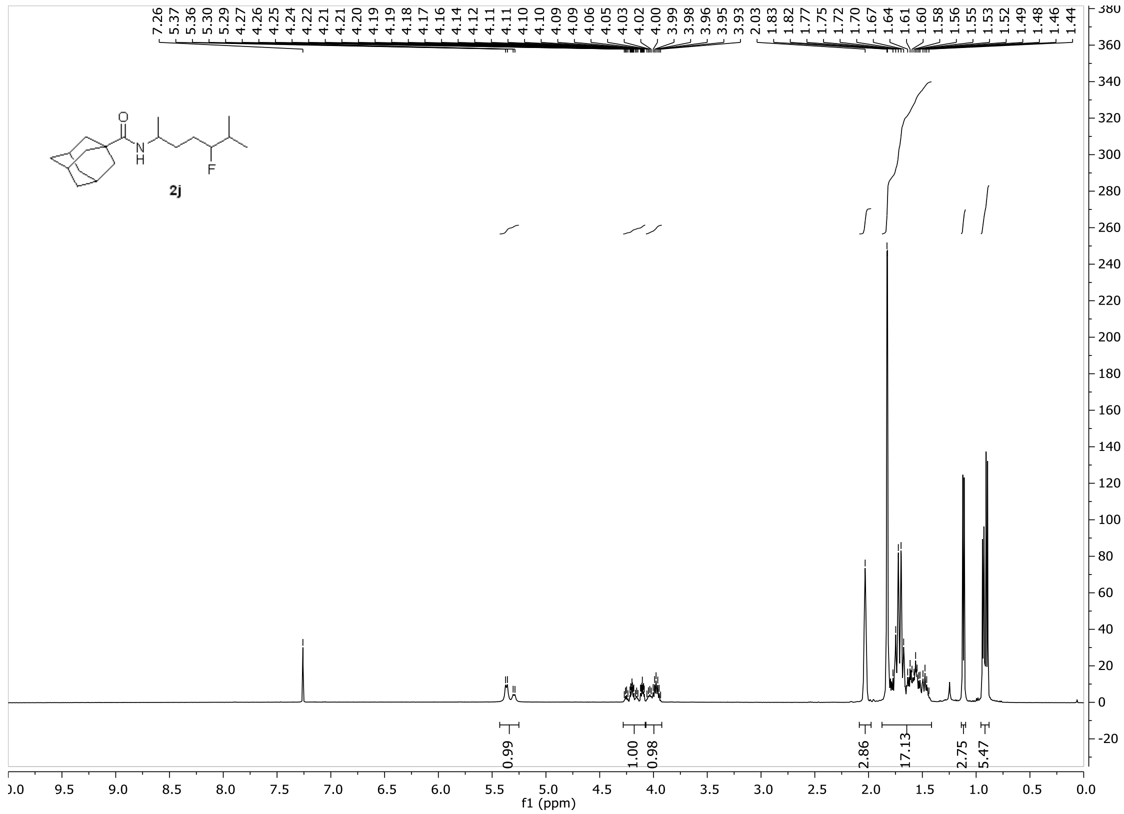
2j
7.26
5.37
5.36
5.30
5.29
4.27
4.26
4.25
4.24
4.22
4.21
4.21
4.20
4.19
4.19
4.18
4.17
4.16
4.14
4.12
4.11
4.11
4.10
4.10
4.09
4.09
4.06
4.05
4.03
4.02
4.00
3.99
3.98
3.96
3.95
3.93
2.03
1.83
1.82
1.77
1.75
1.72
1.70
1.67
1.64
1.61
1.60
1.58
1.56
1.55
1.53
1.52
1.49
1.48
1.46
1.44
0.99
1.00
0.98
2.86
17.13
2.75
5.47
f1 (ppm)

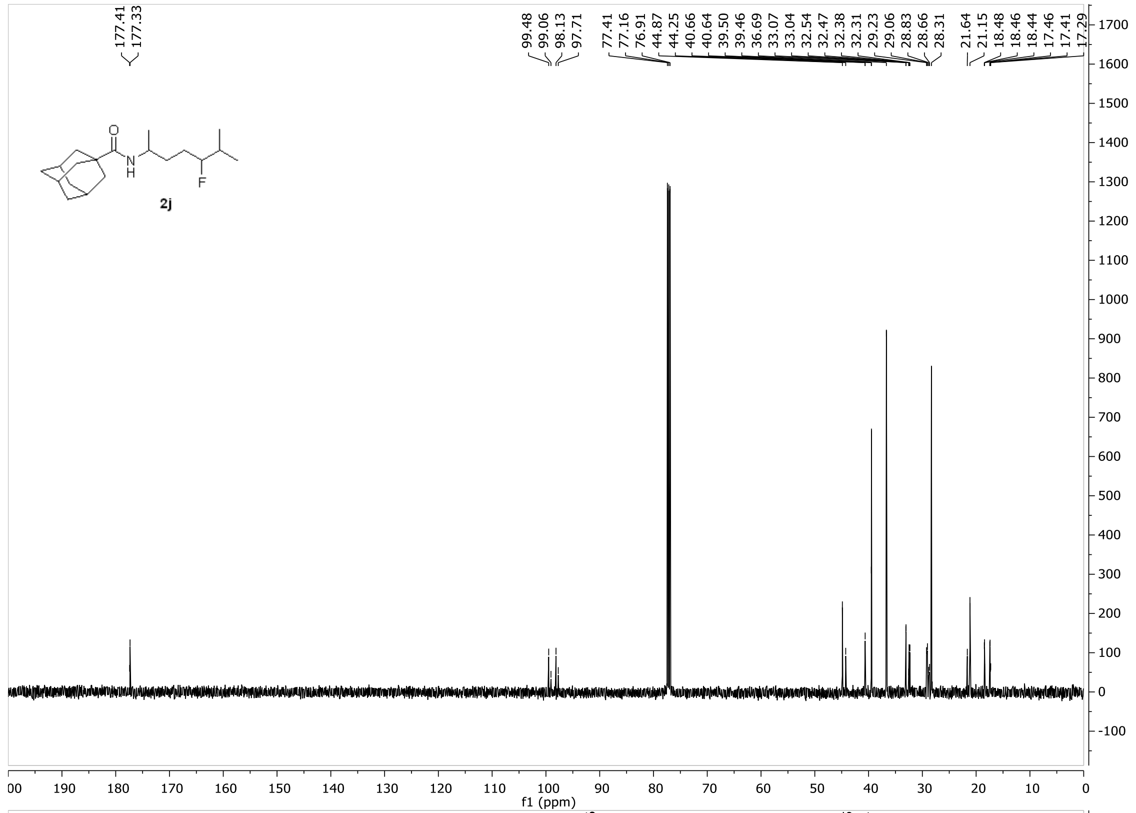
177.41
177.33
99.48
99.06
98.13
97.71
77.41
77.16
76.91
44.87
44.25
40.66
40.64
39.50
39.46
36.69
33.07
33.04
32.54
32.47
32.38
32.31
29.23
29.06
28.83
28.66
28.31
21.64
21.15
18.48
18.46
18.44
17.46
17.41
17.29
2j
f1 (ppm)

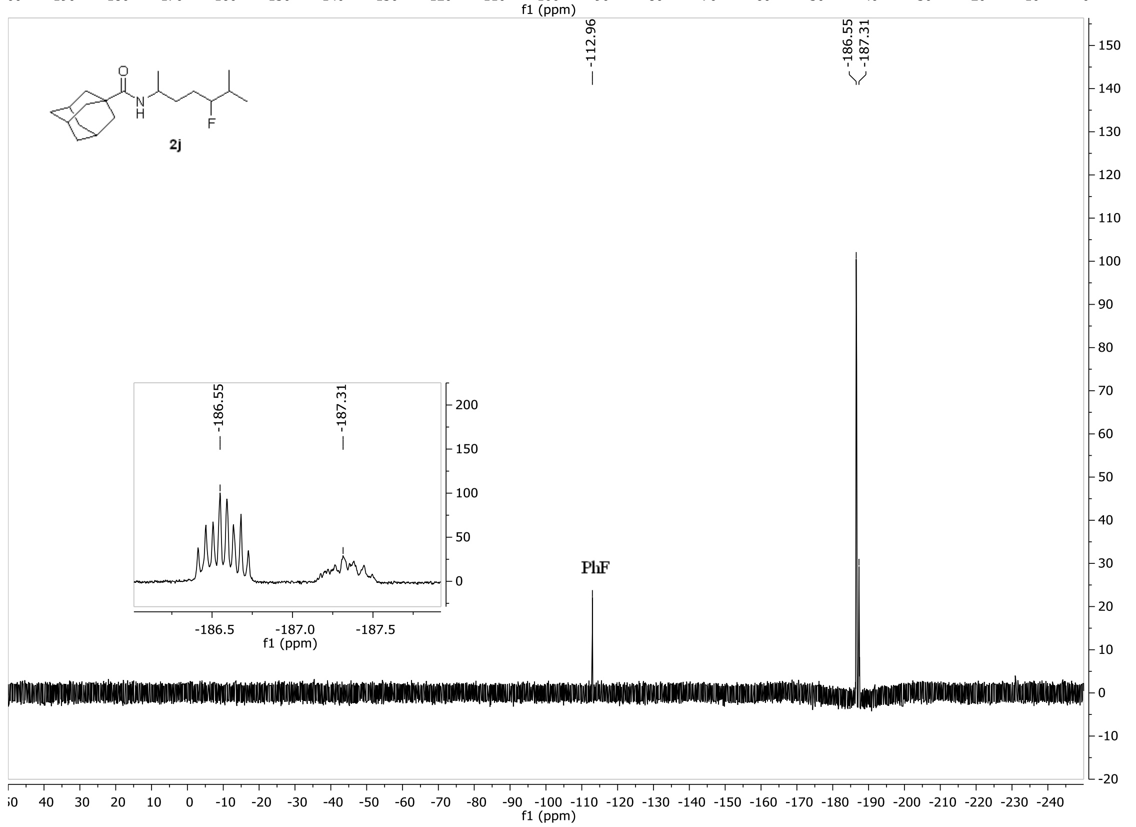
-112.96
-186.55
-187.31
2j
PhF
f1 (ppm)

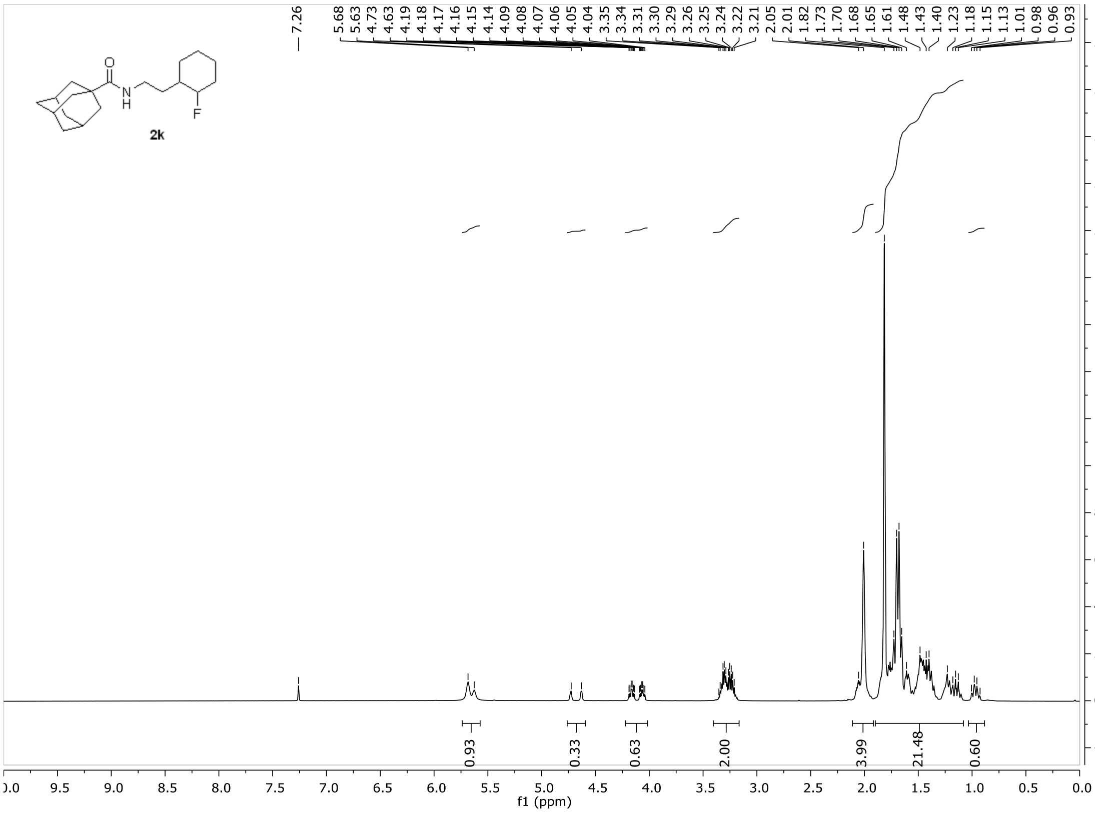

7.26
5.68
5.63
4.73
4.63
4.19
4.18
4.17
4.16
4.15
4.14
4.09
4.08
4.07
4.06
4.05
4.04
3.35
3.34
3.31
3.30
3.29
3.26
3.25
3.24
3.22
3.21
2.05
2.01
1.82
1.73
1.70
1.68
1.65
1.61
1.48
1.43
1.40
1.23
1.18
1.15
1.13
1.01
0.98
0.96
0.93
2k
0.93
0.33
0.63
2.00
3.99
21.48
0.60
f1 (ppm)

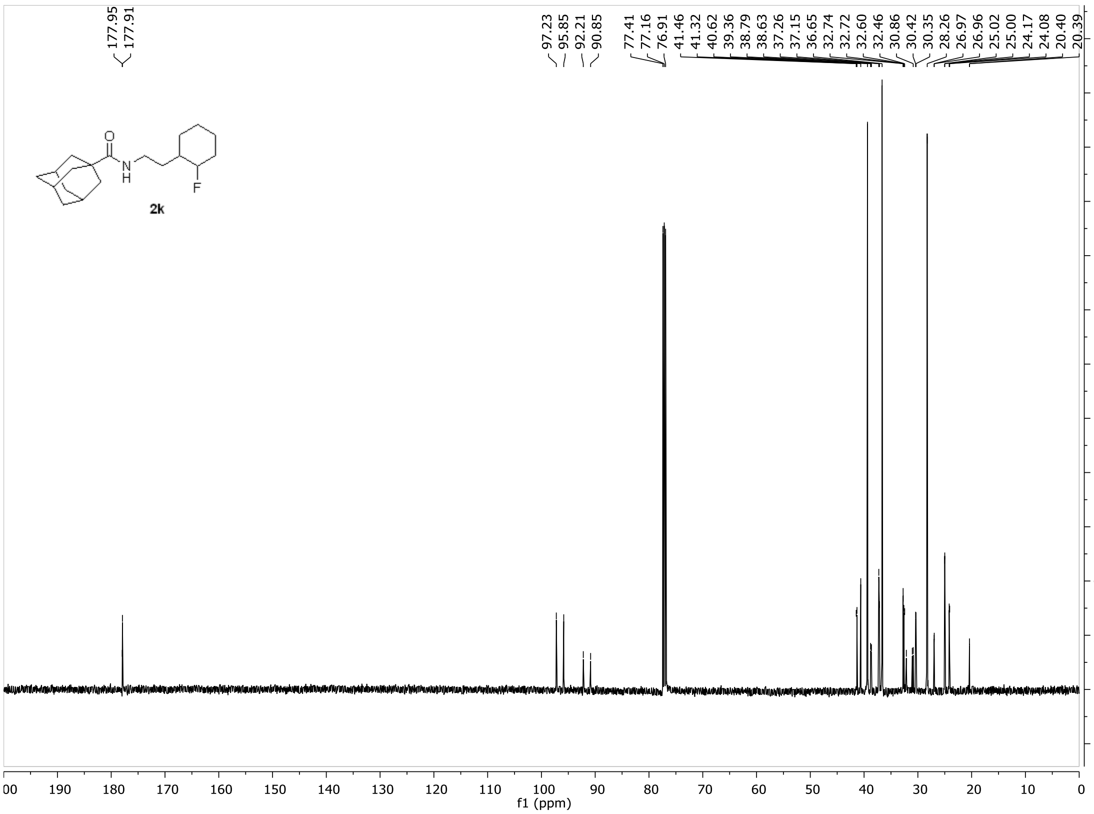

177.95
177.91
97.23
95.85
92.21
90.85
77.41
77.16
76.91
41.46
41.32
40.62
39.36
38.79
38.63
37.26
37.15
36.65
32.74
32.72
32.60
32.46
30.86
30.42
30.35
28.26
26.97
26.96
25.02
25.00
24.17
24.08
20.40
20.39
2k
f1 (ppm)

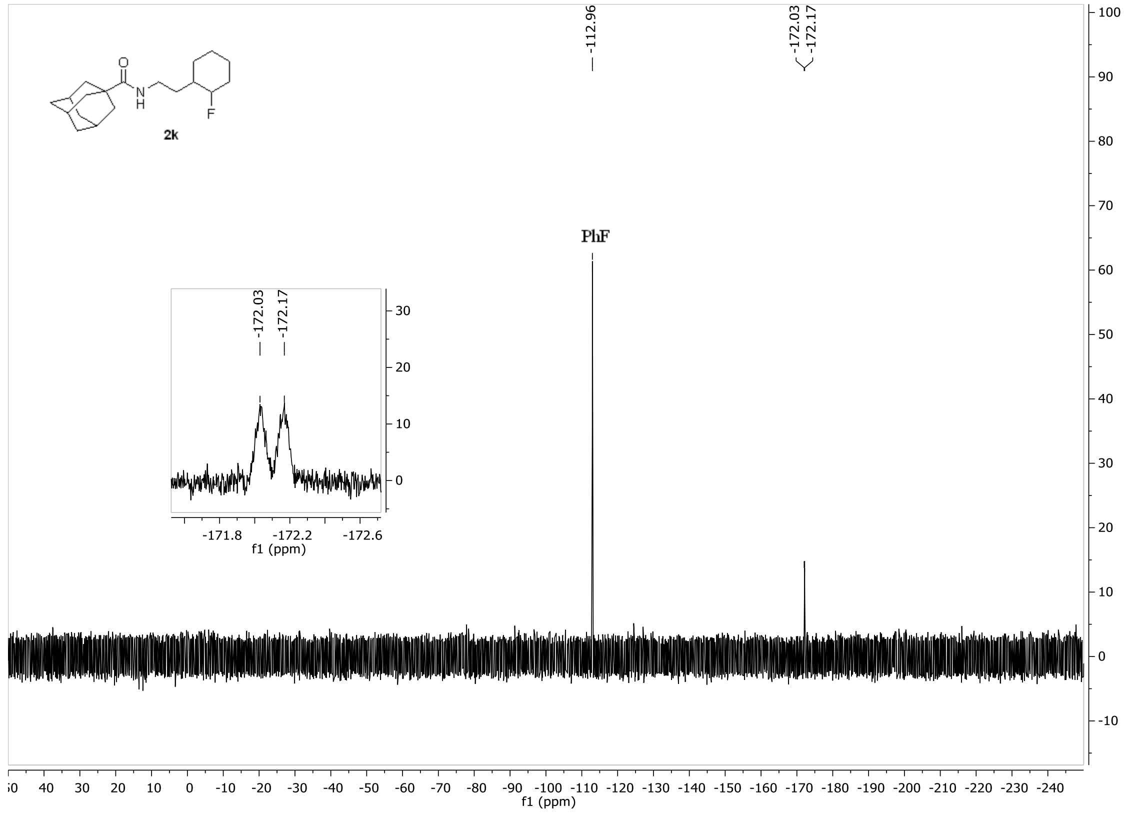

2k
PhF
-112.96
-172.03
-172.17
f1 (ppm)

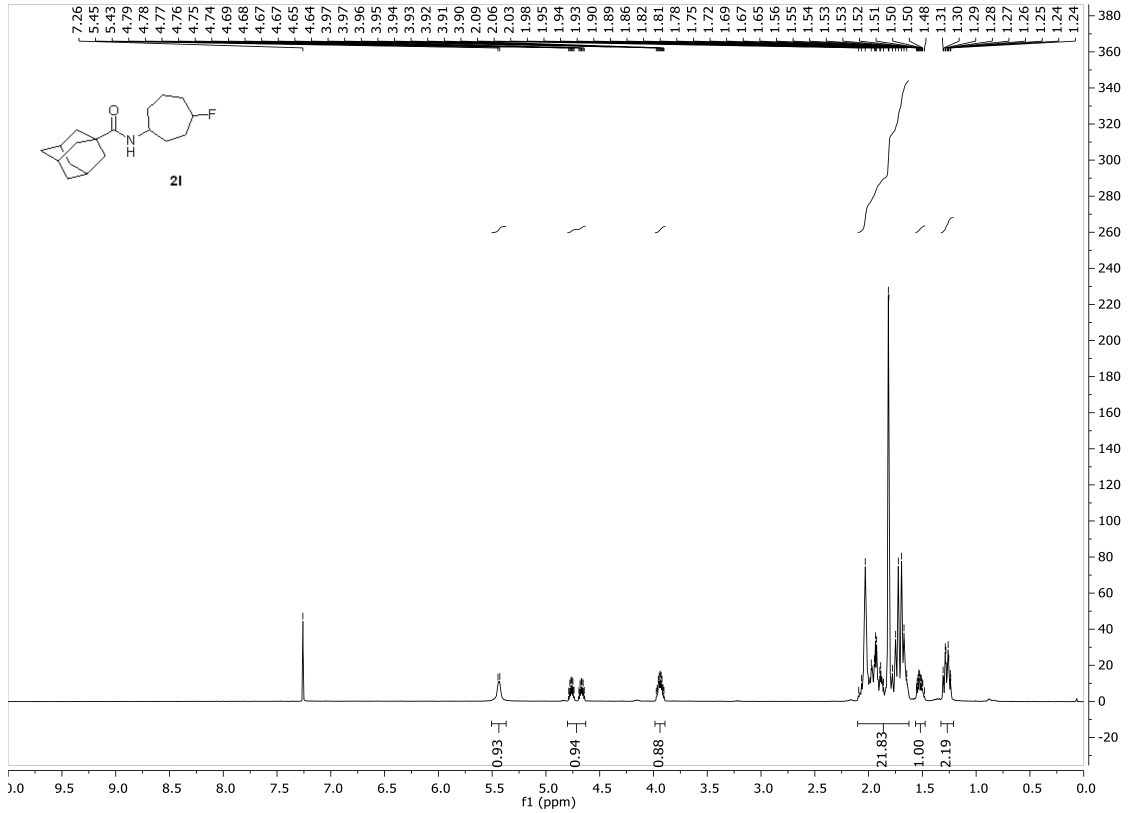

2l
0.93
0.94
0.88
21.83
1.00
2.19
f1 (ppm)

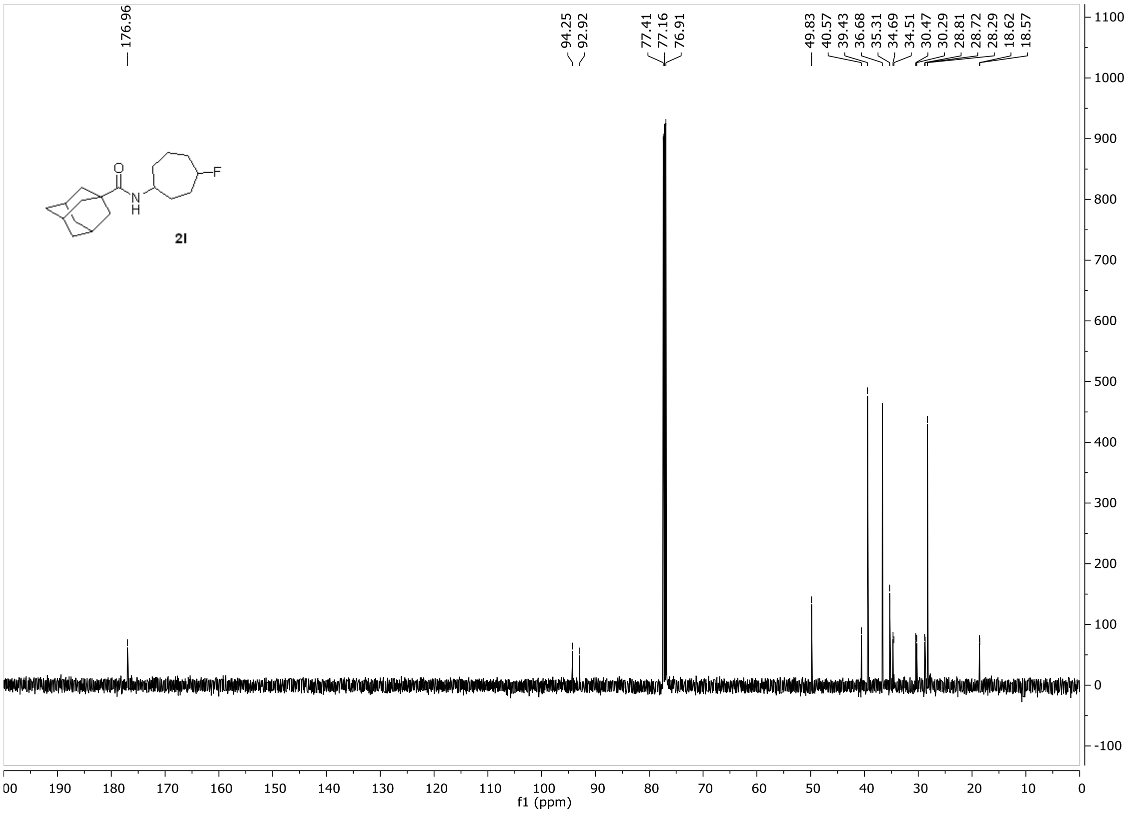
176.96
94.25
92.92
77.41
77.16
76.91
49.83
40.57
39.43
36.68
35.31
34.69
34.51
30.47
30.29
28.81
28.72
28.29
18.62
18.57
O
N
H
F
2l
f1 (ppm)

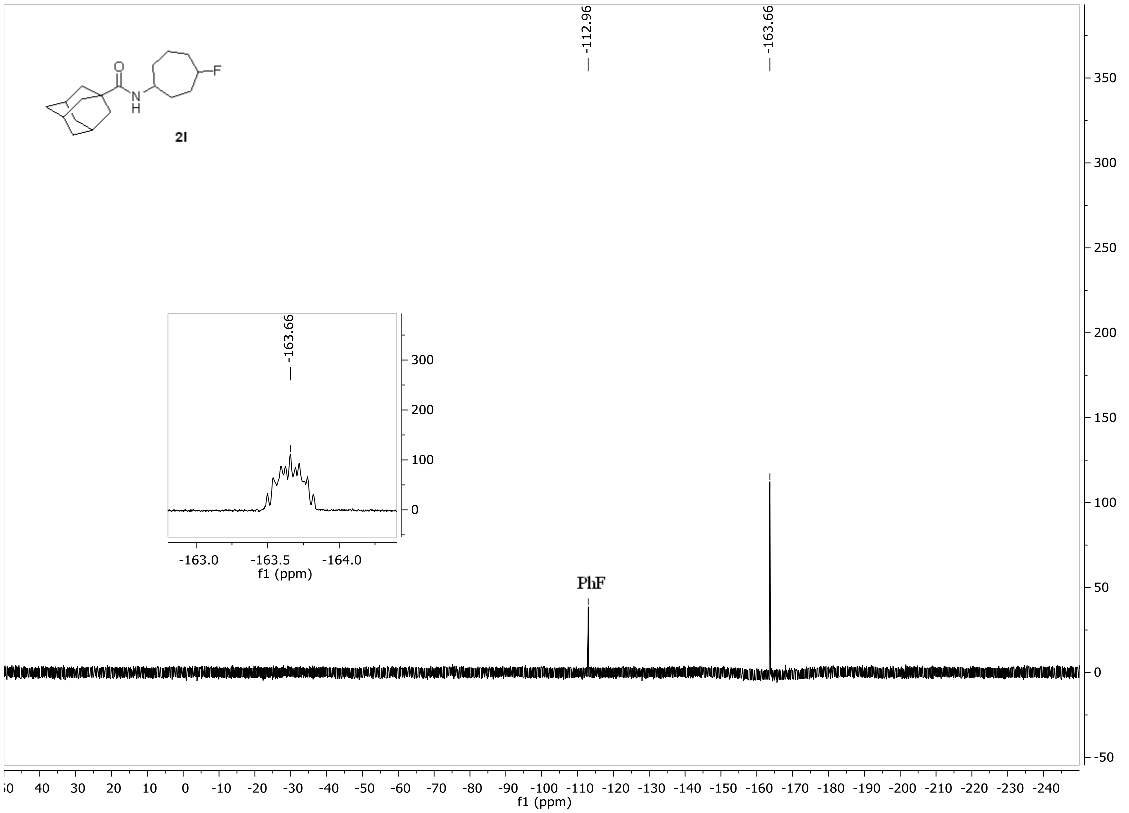
-112.96
-163.66
O
N
H
F
2l
-163.66
-163.0
-163.5
-164.0
f1 (ppm)
PhF
f1 (ppm)

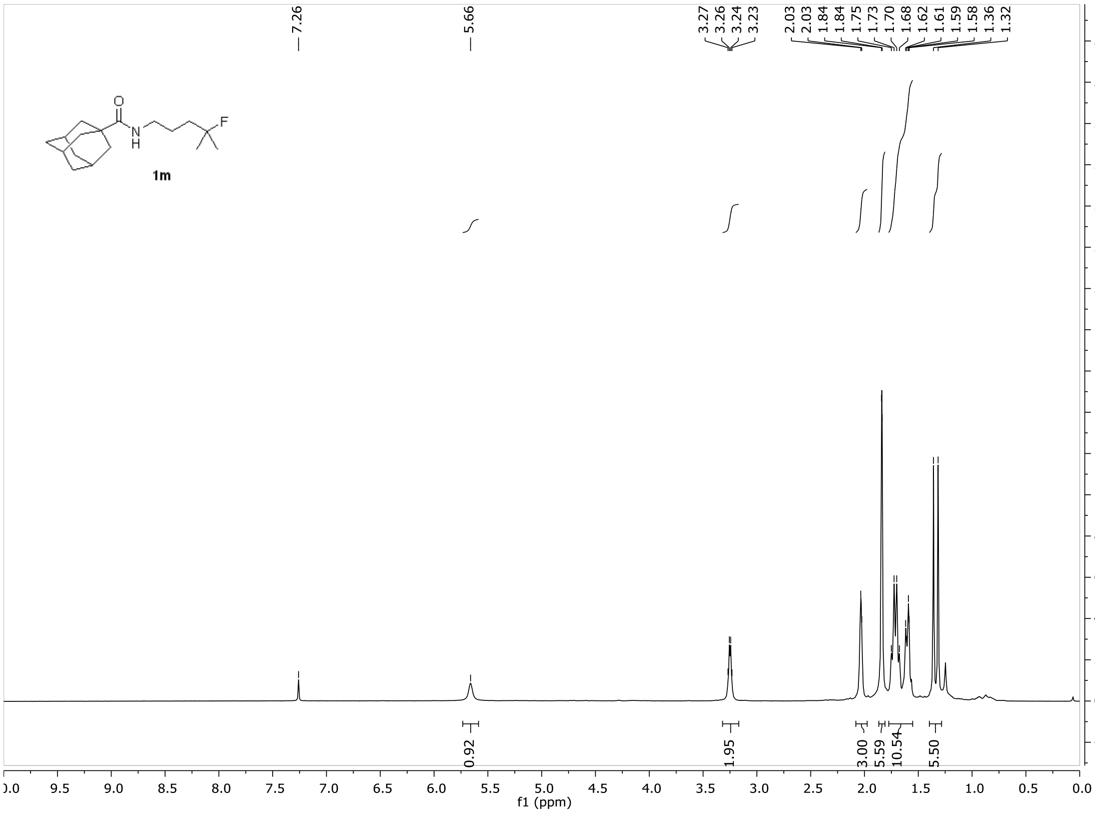

7.26
5.66
3.27
3.26
3.24
3.23
2.03
2.03
1.84
1.84
1.75
1.73
1.70
1.68
1.62
1.61
1.59
1.58
1.36
1.32
O
N
H
F
1m
0.92
1.95
3.00
5.59
10.54
5.50
f1 (ppm)

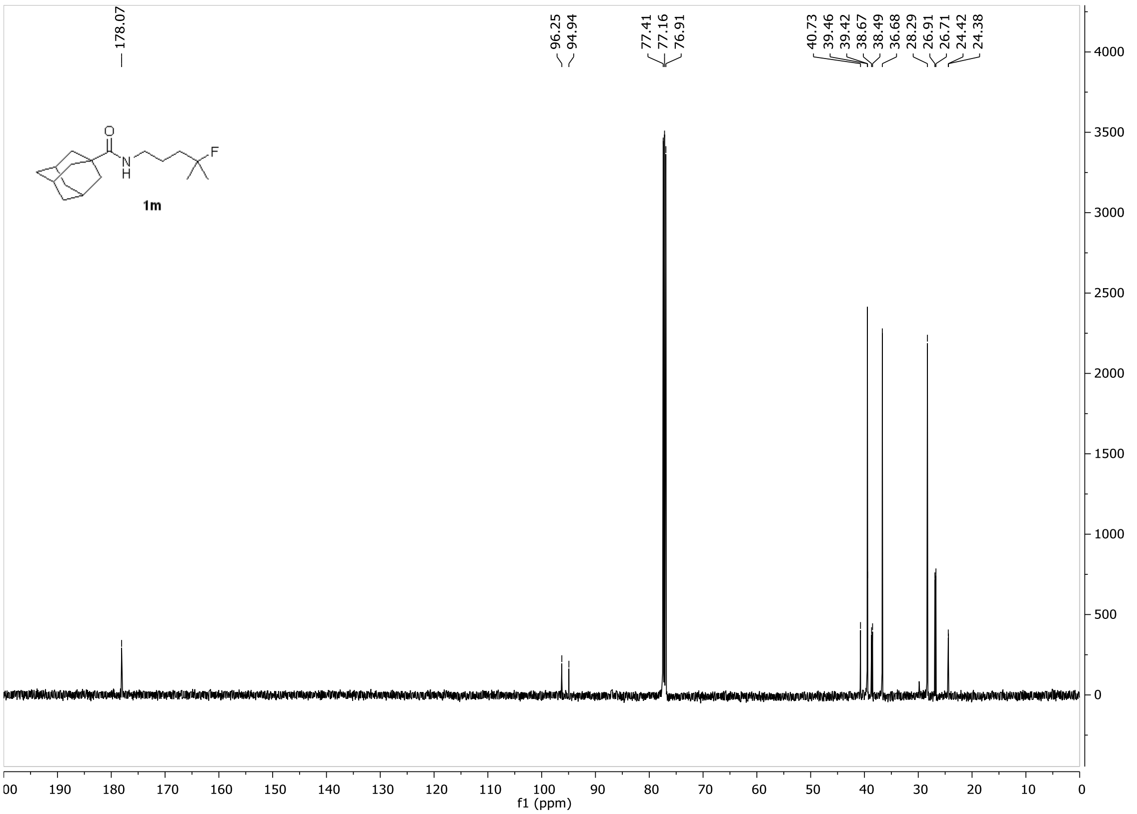

178.07
96.25
94.94
77.41
77.16
76.91
40.73
39.46
39.42
38.67
38.49
36.68
28.29
26.91
26.71
24.42
24.38
O
N
H
F
1m
f1 (ppm)

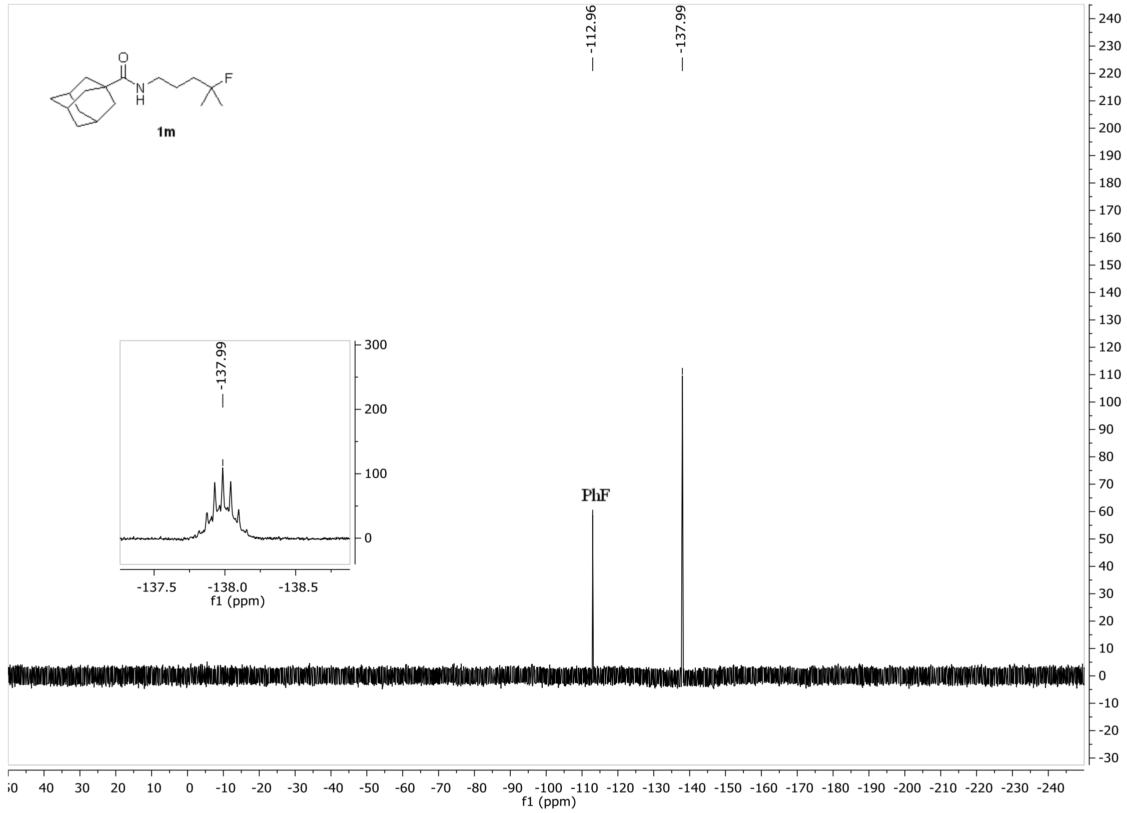
-112.96
-137.99
1m
PhF
-137.99
f1 (ppm)
f1 (ppm)

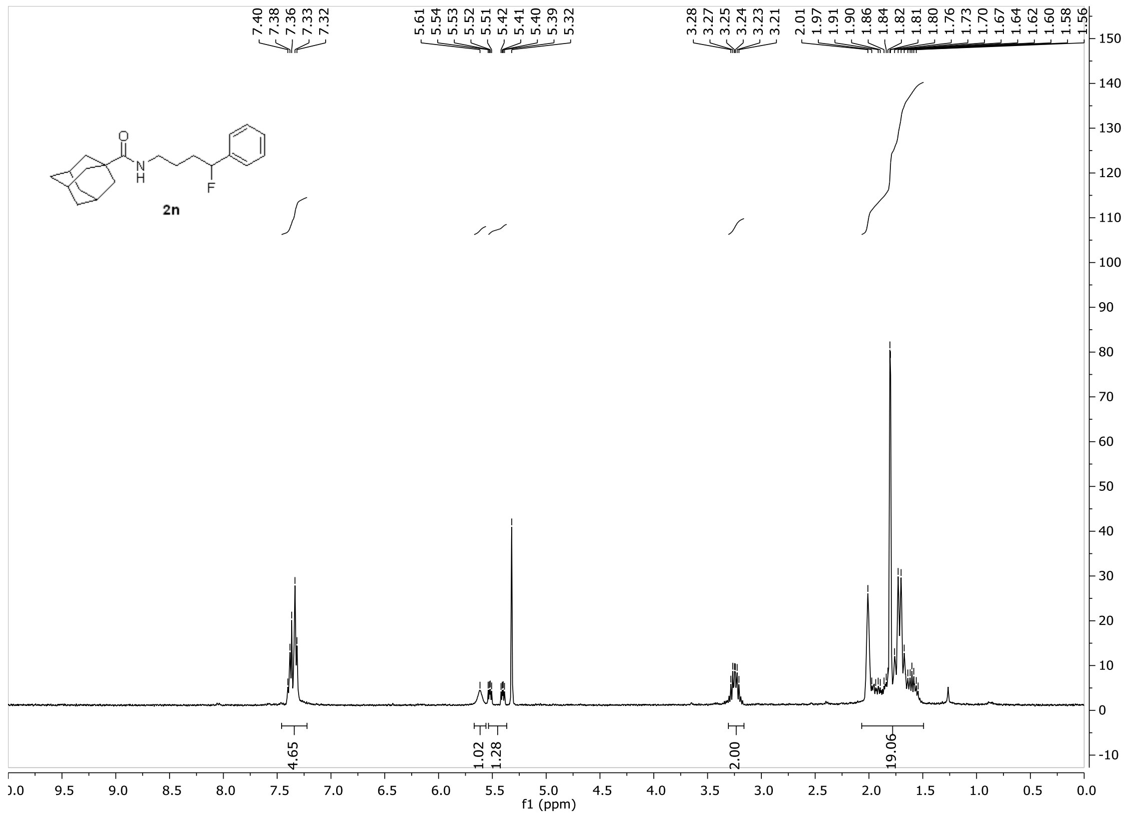
7.40
7.38
7.36
7.33
7.32
5.61
5.54
5.53
5.52
5.51
5.42
5.41
5.40
5.39
5.32
3.28
3.27
3.25
3.24
3.23
3.21
2.01
1.97
1.91
1.90
1.86
1.84
1.82
1.81
1.80
1.76
1.73
1.70
1.67
1.64
1.62
1.60
1.58
1.56
2n
4.65
1.02
1.28
2.00
19.06
f1 (ppm)

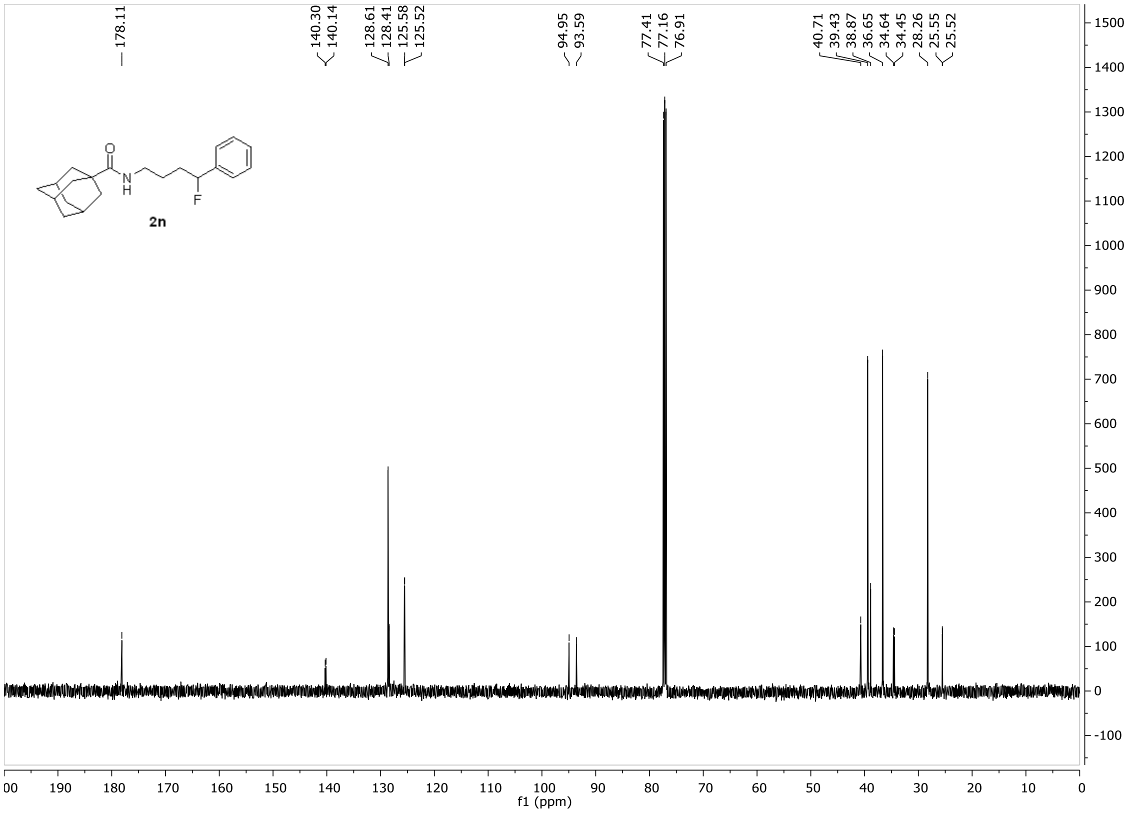
178.11
140.30
140.14
128.61
128.41
125.58
125.52
94.95
93.59
77.41
77.16
76.91
40.71
39.43
38.87
36.65
34.64
34.45
28.26
25.55
25.52
2n
f1 (ppm)

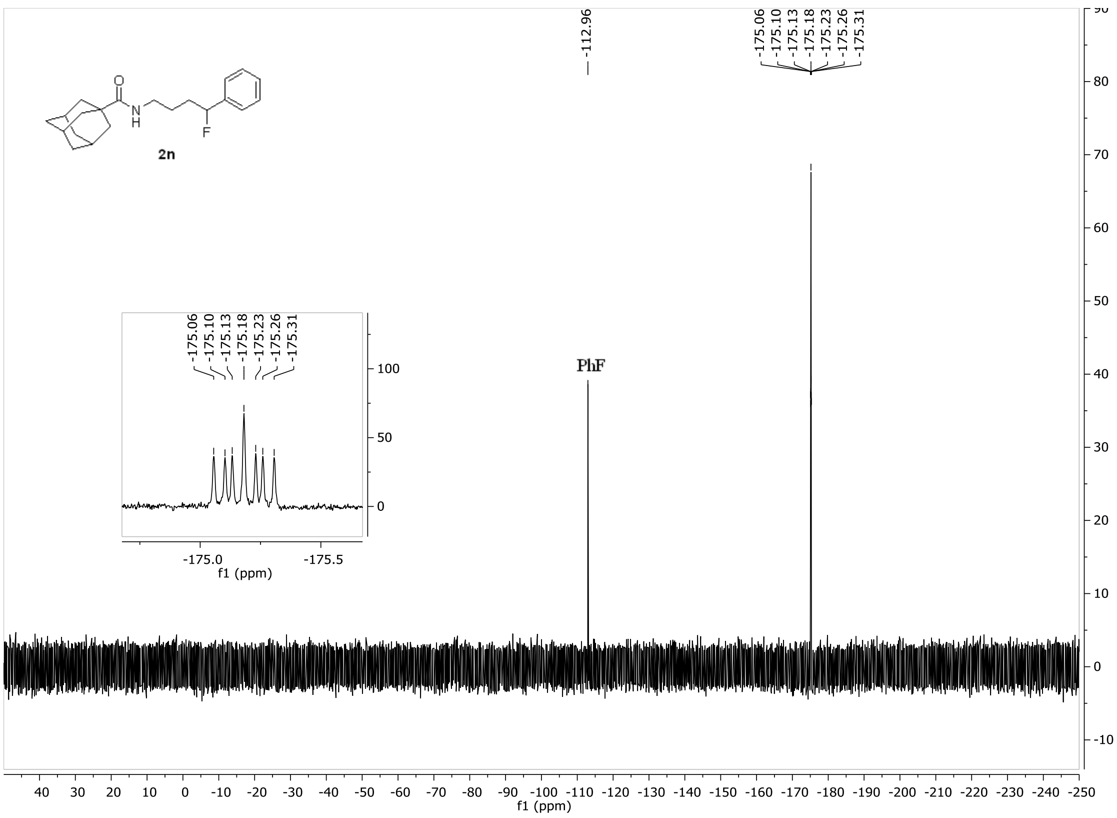
-112.96
-175.06
-175.10
-175.13
-175.18
-175.23
-175.26
-175.31
2n
PhF
f1 (ppm)

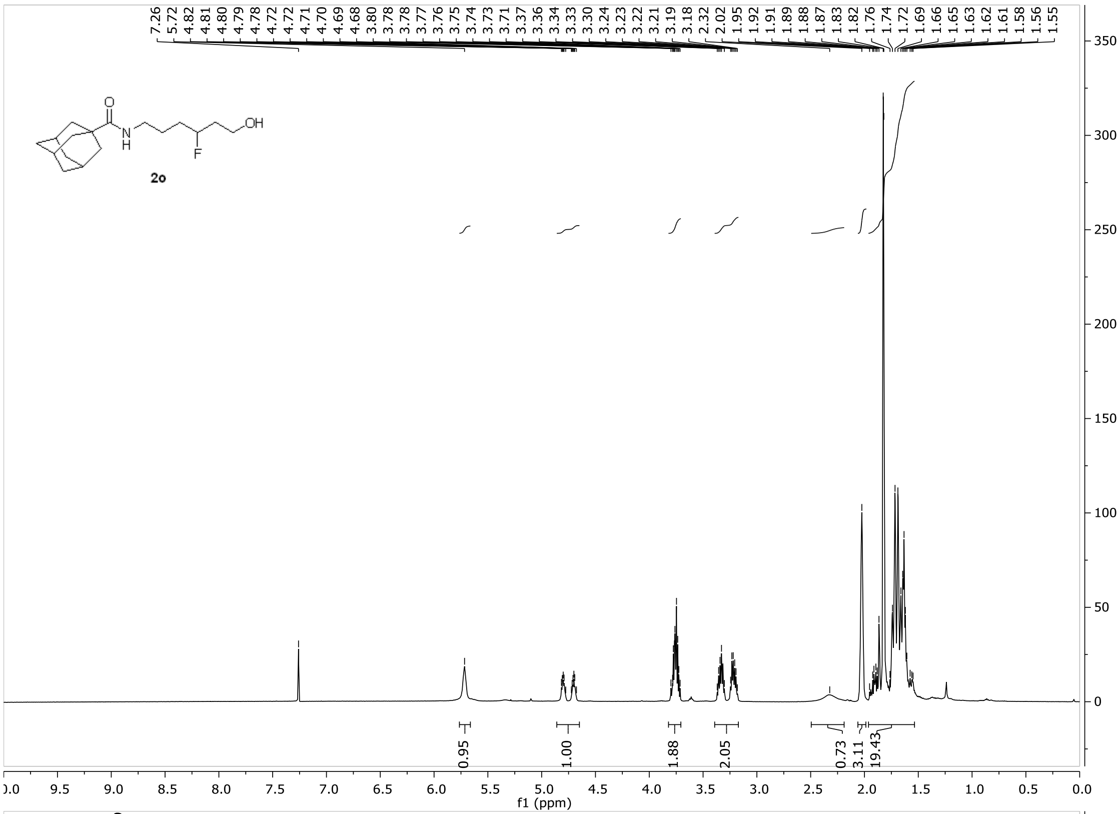
7.26
5.72
4.81
4.80
4.79
4.78
4.72
4.72
4.71
4.70
4.69
4.68
3.80
3.78
3.78
3.77
3.76
3.75
3.74
3.73
3.71
3.37
3.36
3.34
3.33
3.30
3.24
3.23
3.22
3.21
3.19
3.18
2.32
2.02
1.95
1.92
1.91
1.89
1.88
1.87
1.83
1.82
1.76
1.74
1.72
1.69
1.66
1.65
1.63
1.62
1.61
1.58
1.56
1.55
O
N
H
F
OH
2o
0.95
1.00
1.88
2.05
0.73
3.11
19.43
f1 (ppm)

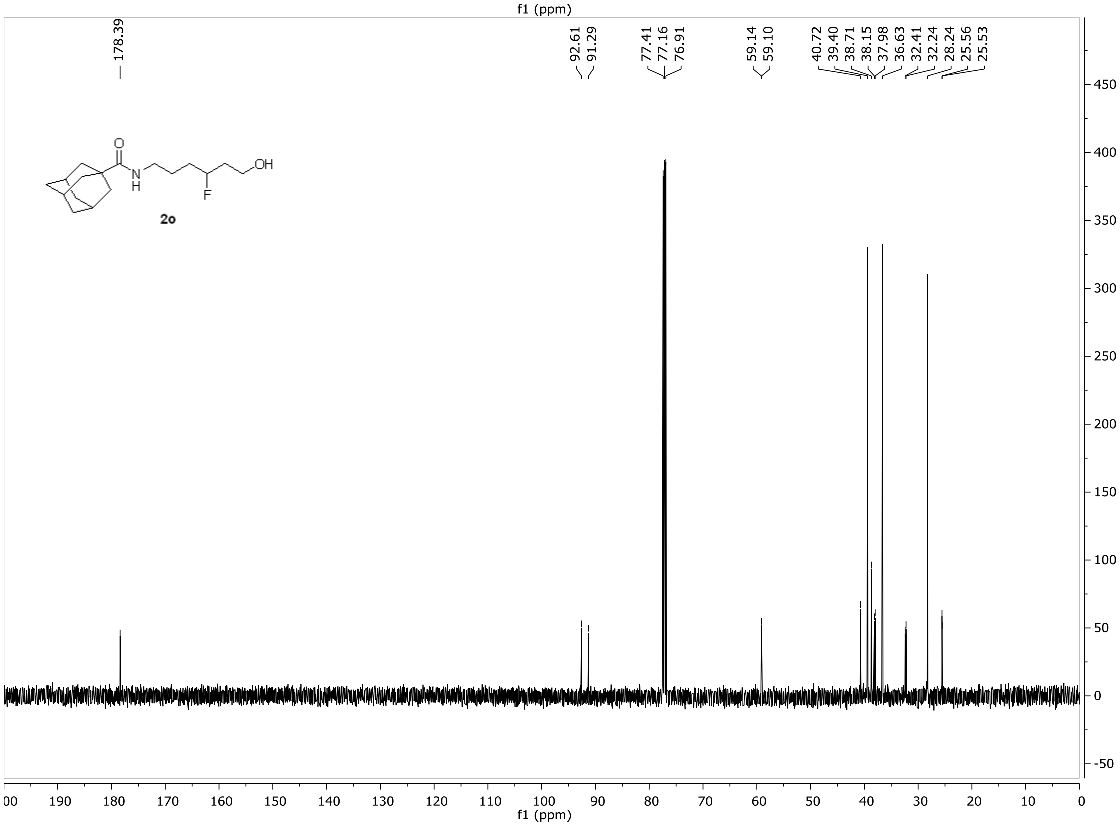
178.39
92.61
91.29
77.41
77.16
76.91
59.14
59.10
40.72
39.40
38.71
38.15
37.98
36.63
32.41
32.24
28.24
25.56
25.53
O
N
H
F
OH
2o
f1 (ppm)

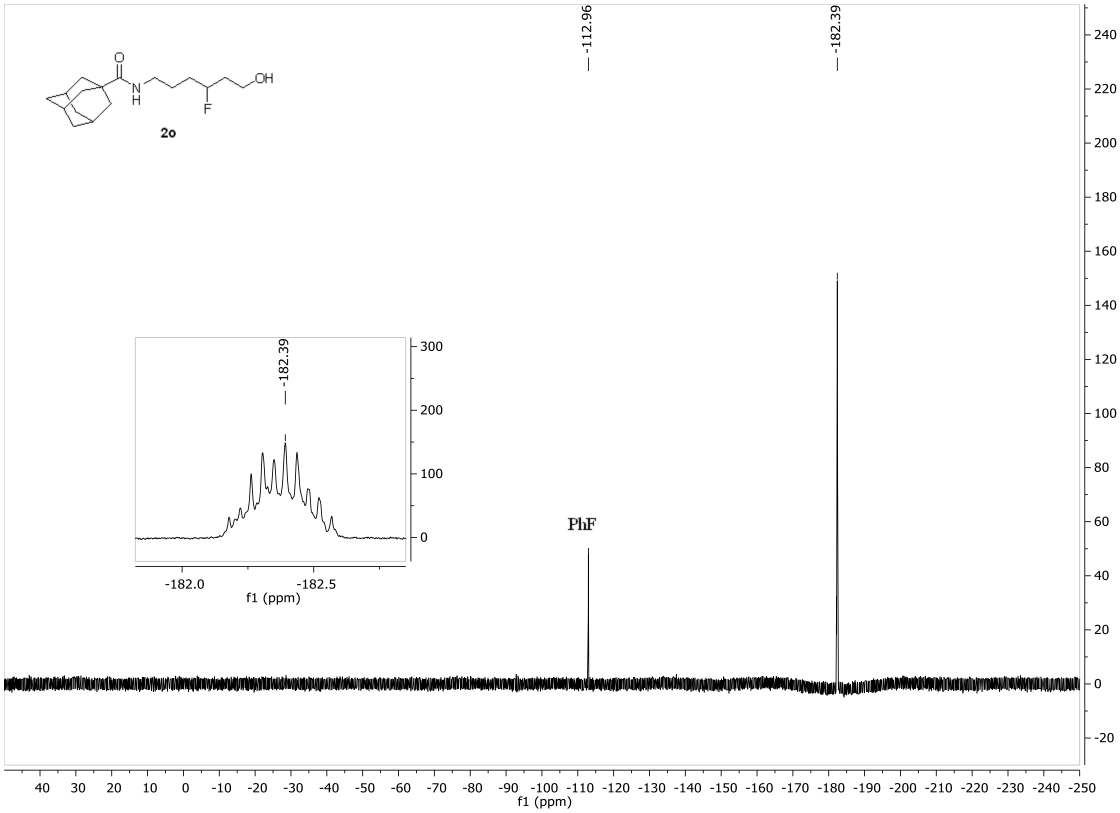
-112.96
-182.39
2o
PhF
f1 (ppm)

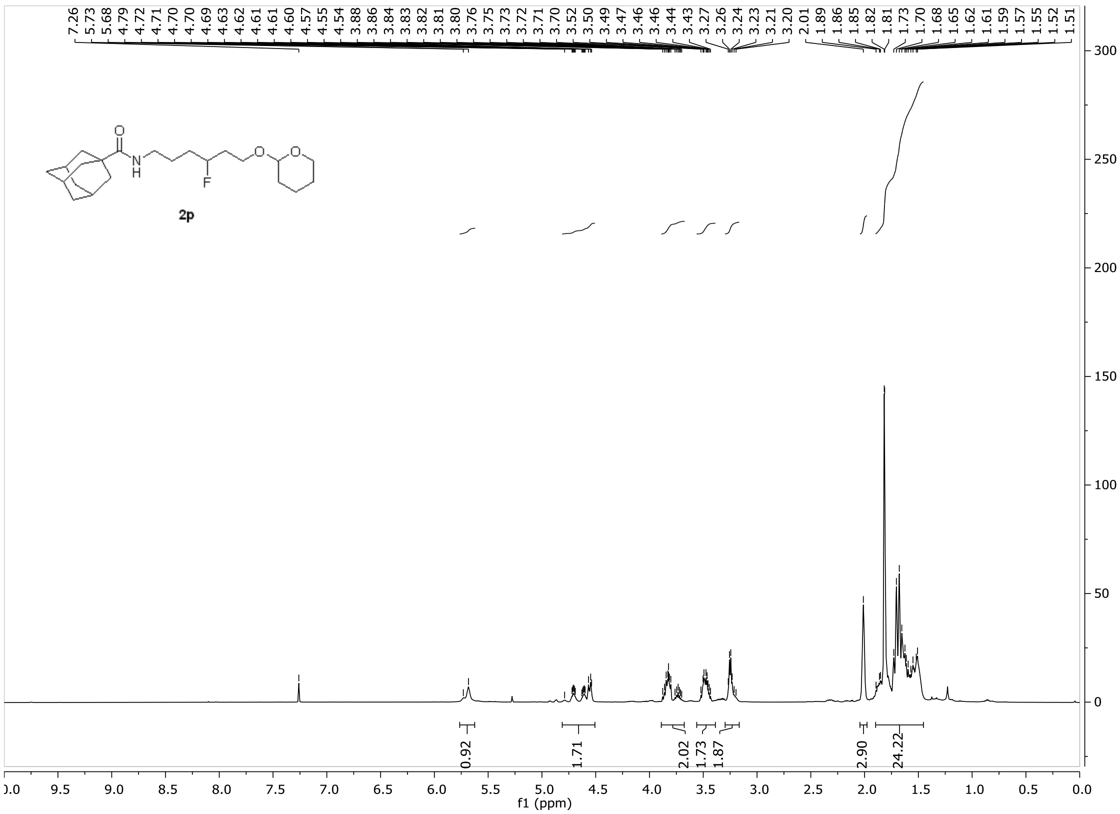
2p
f1 (ppm)

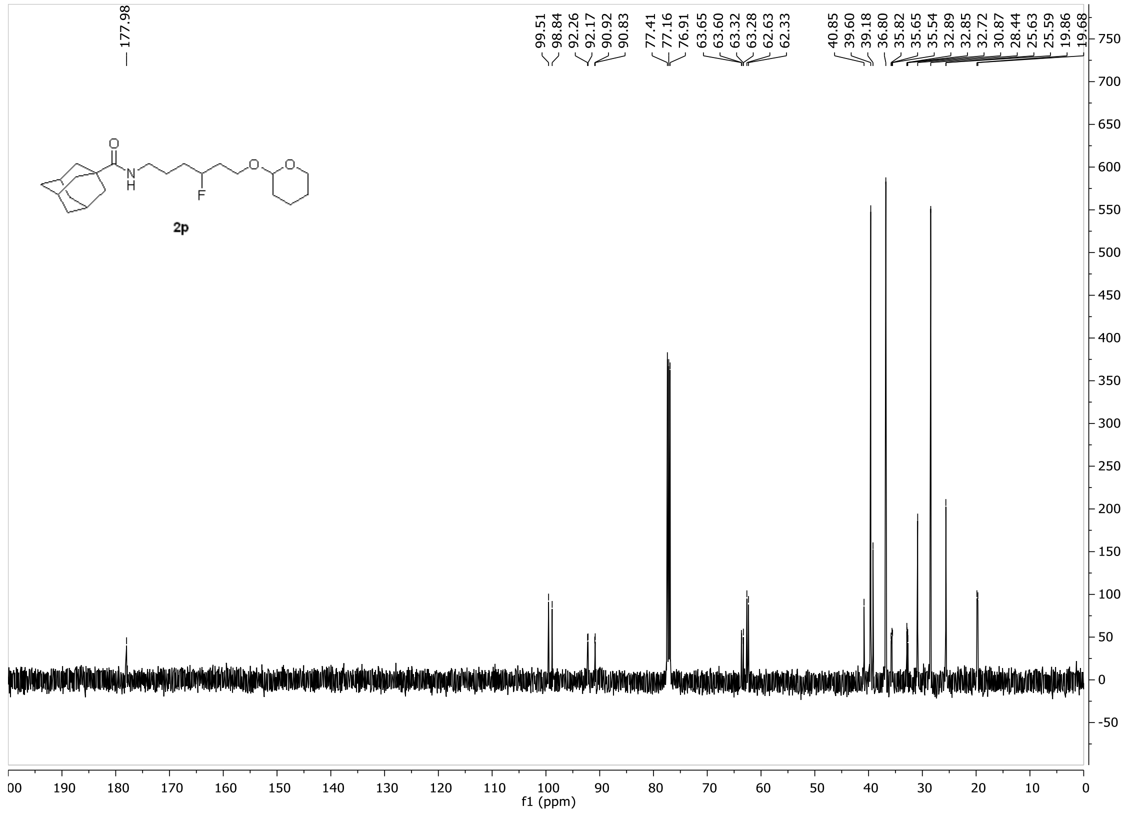
177.98
99.51
98.84
92.26
92.17
90.92
90.83
77.41
77.16
76.91
63.65
63.60
63.32
63.28
62.63
62.33
40.85
39.60
39.18
36.80
35.82
35.65
35.54
32.89
32.85
32.72
30.87
28.44
25.63
25.59
19.86
19.68
2p
f1 (ppm)

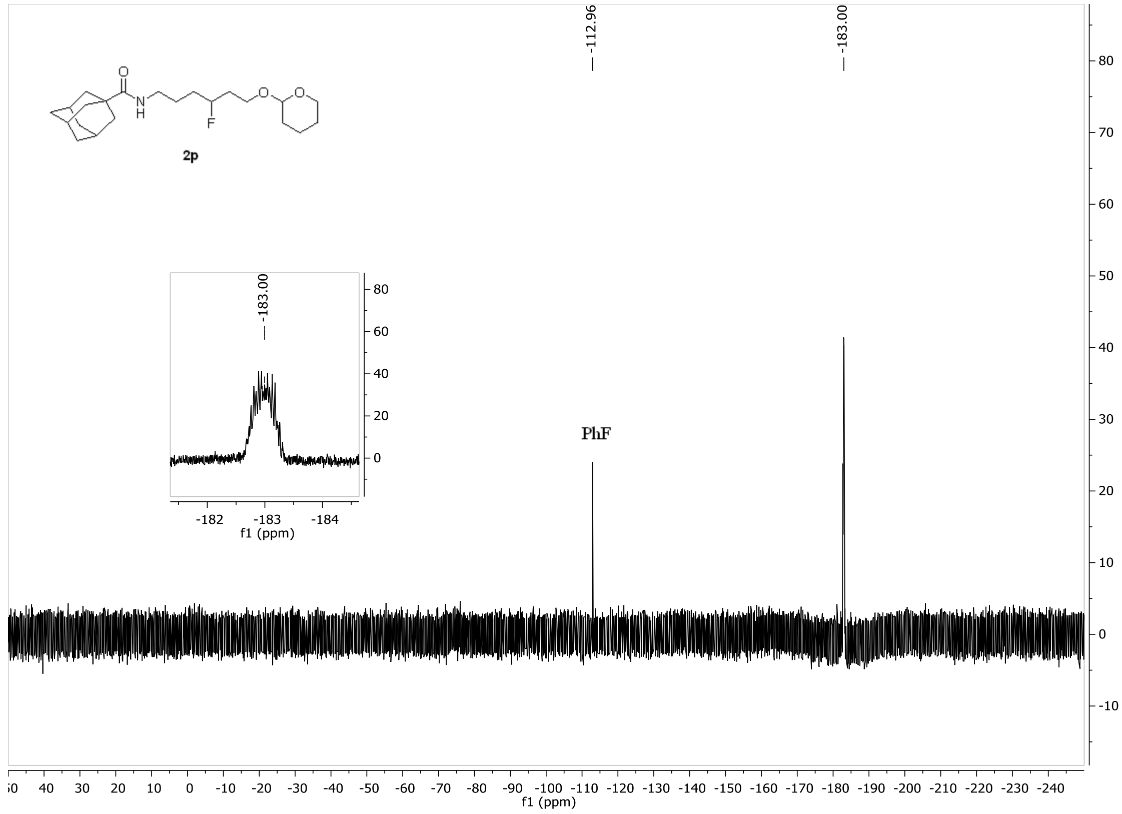
-112.96
-183.00
2p
PhF
f1 (ppm)

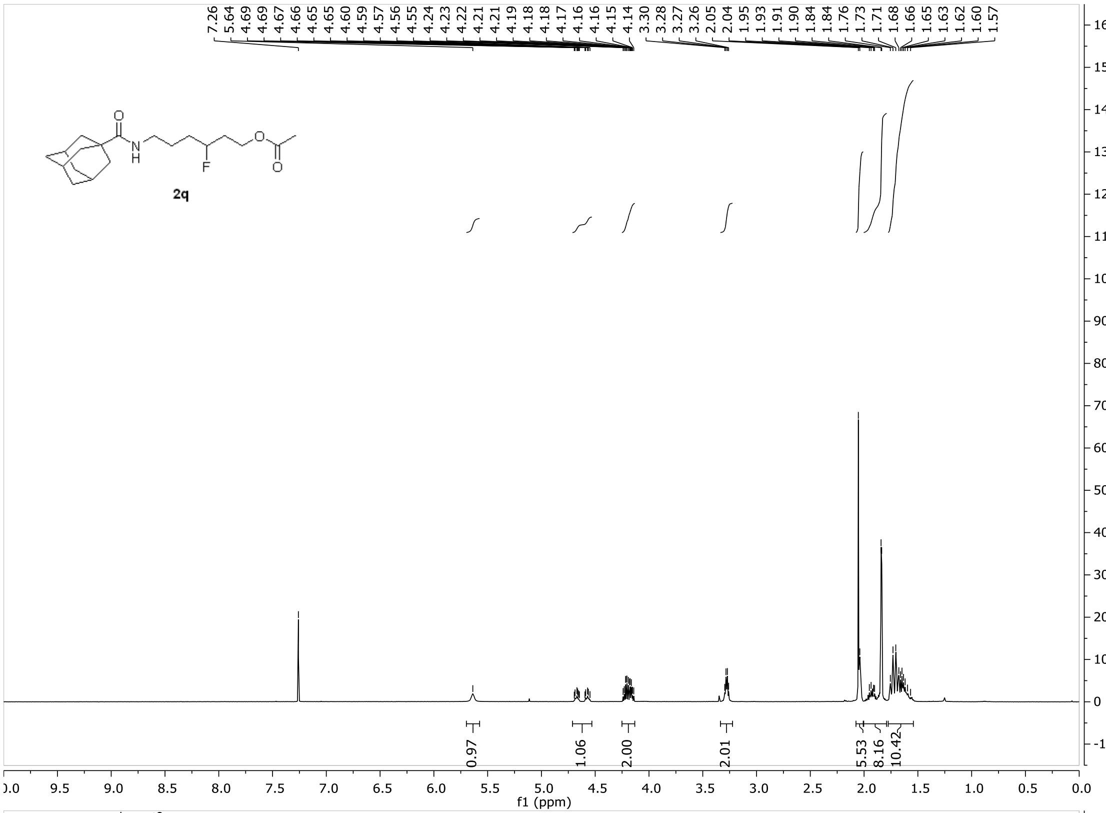
7.26
5.64
4.69
4.69
4.67
4.66
4.65
4.65
4.60
4.59
4.57
4.56
4.55
4.24
4.23
4.22
4.21
4.21
4.19
4.18
4.18
4.17
4.16
4.16
4.15
4.14
3.30
3.28
3.27
3.26
2.05
2.04
1.95
1.93
1.91
1.90
1.84
1.84
1.76
1.73
1.71
1.68
1.66
1.65
1.63
1.62
1.60
1.57
2q
0.97
1.06
2.00
2.01
5.53
8.16
10.42
f1 (ppm)

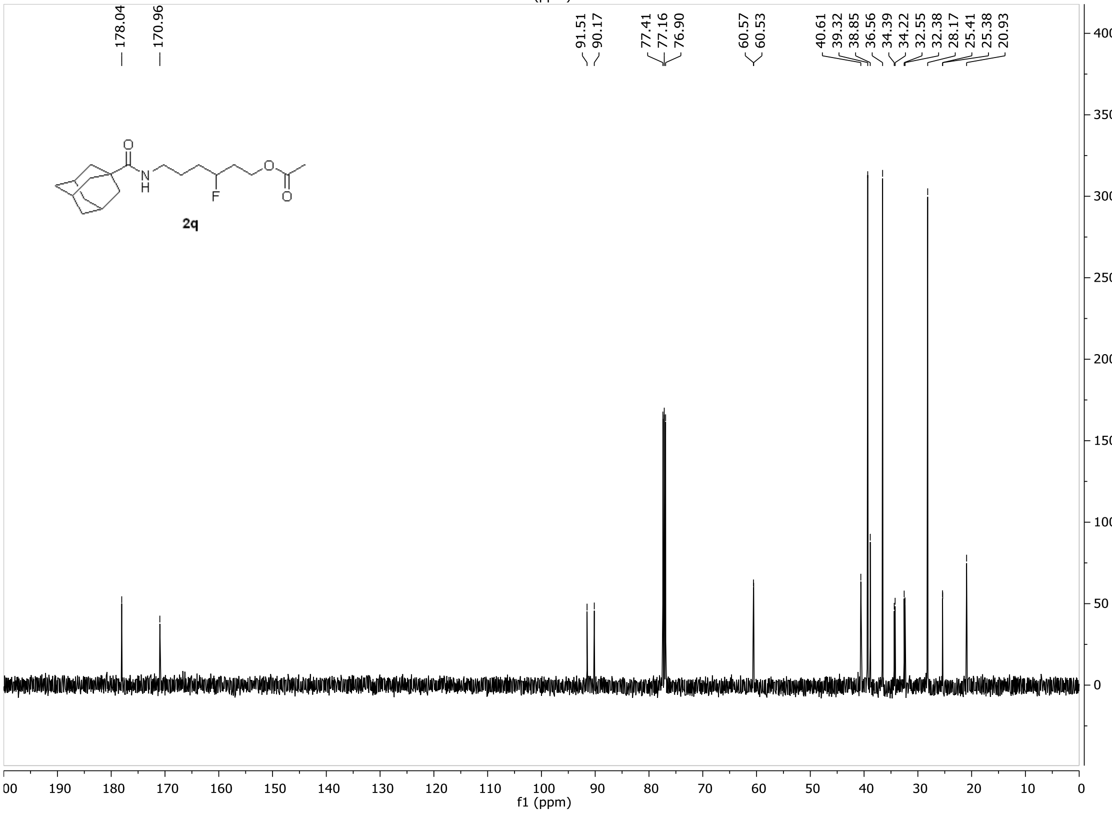
178.04
170.96
91.51
90.17
77.41
77.16
76.90
60.57
60.53
40.61
39.32
38.85
36.56
34.39
34.22
32.55
32.38
28.17
25.41
25.38
20.93
2q
f1 (ppm)

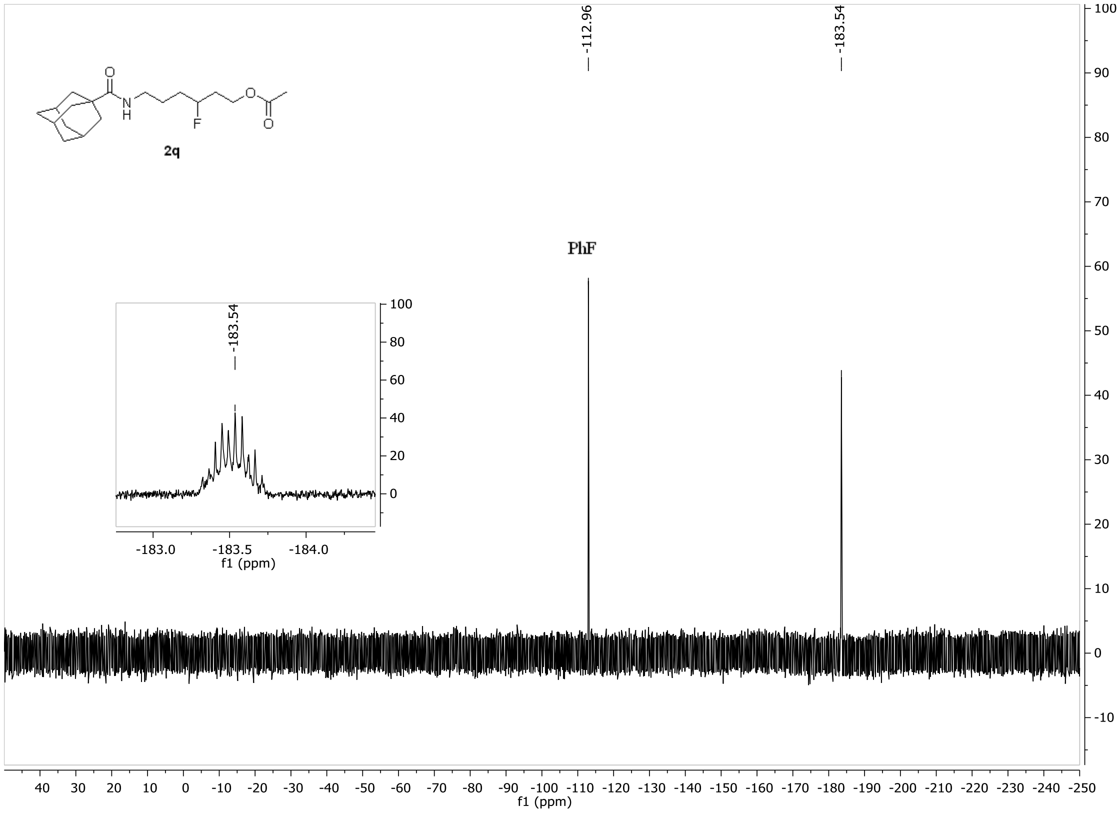

2q
PhF
-112.96
-183.54
f1 (ppm)

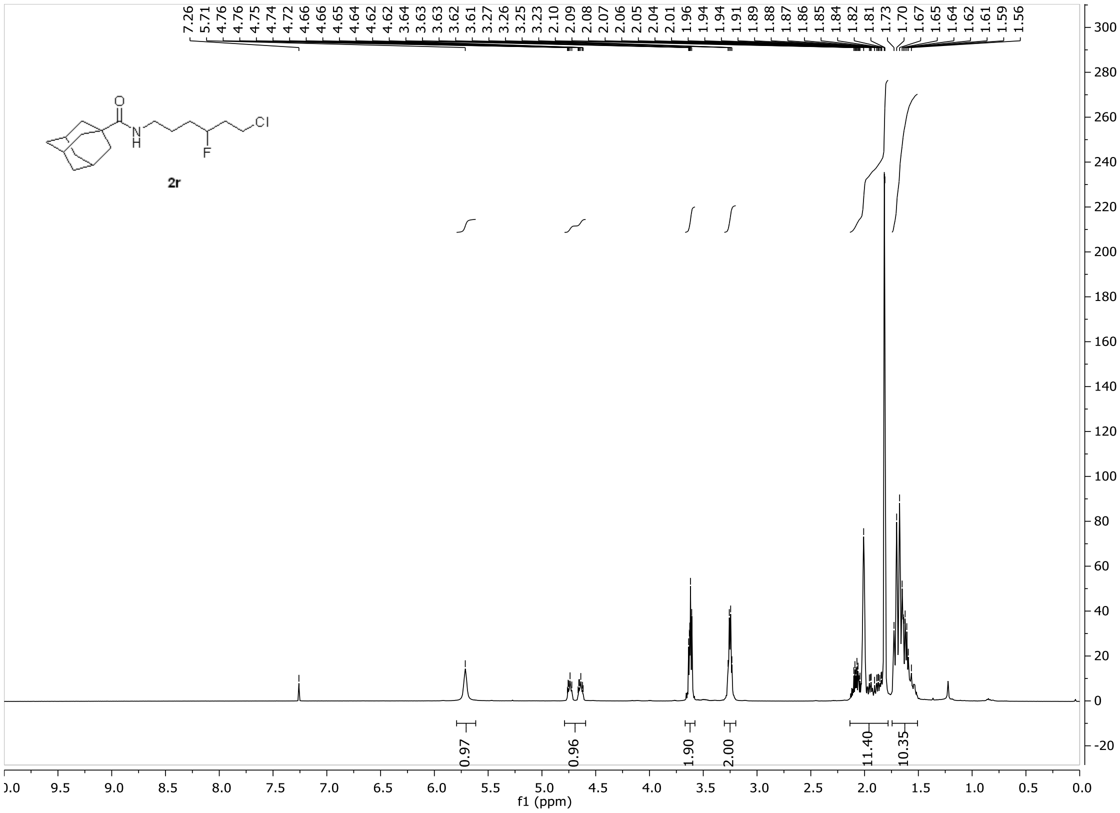

2r
f1 (ppm)
0.97
0.96
1.90
2.00
11.40
10.35

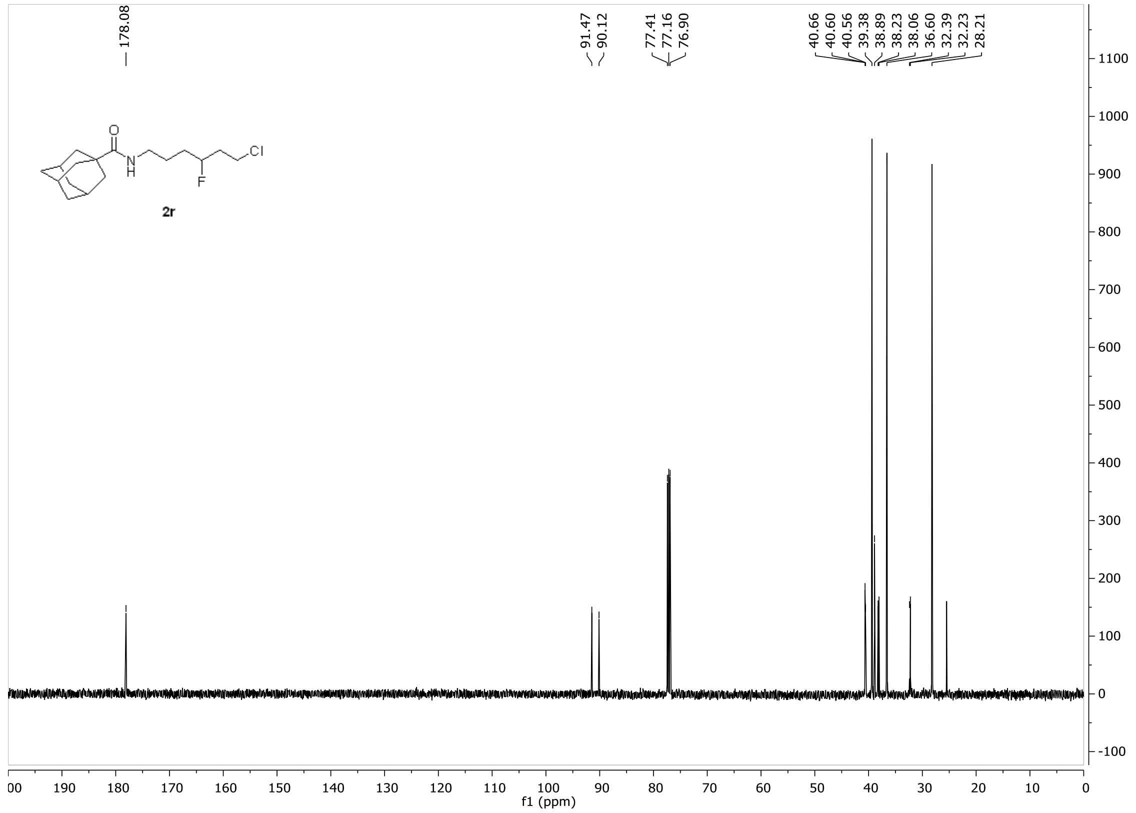
178.08
91.47
90.12
77.41
77.16
76.90
40.66
40.60
40.56
39.38
38.89
38.23
38.06
36.60
32.39
32.23
28.21
2r
f1 (ppm)

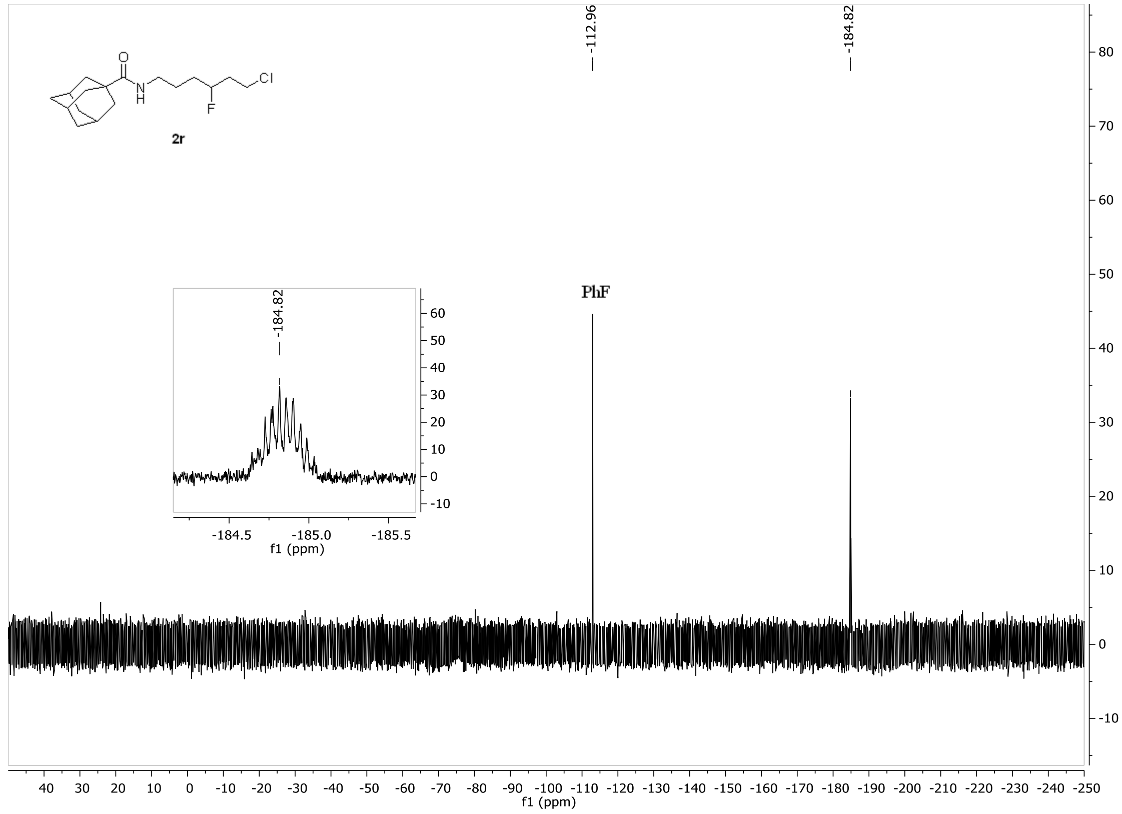
-112.96
-184.82
2r
PhF
-184.82
f1 (ppm)
f1 (ppm)

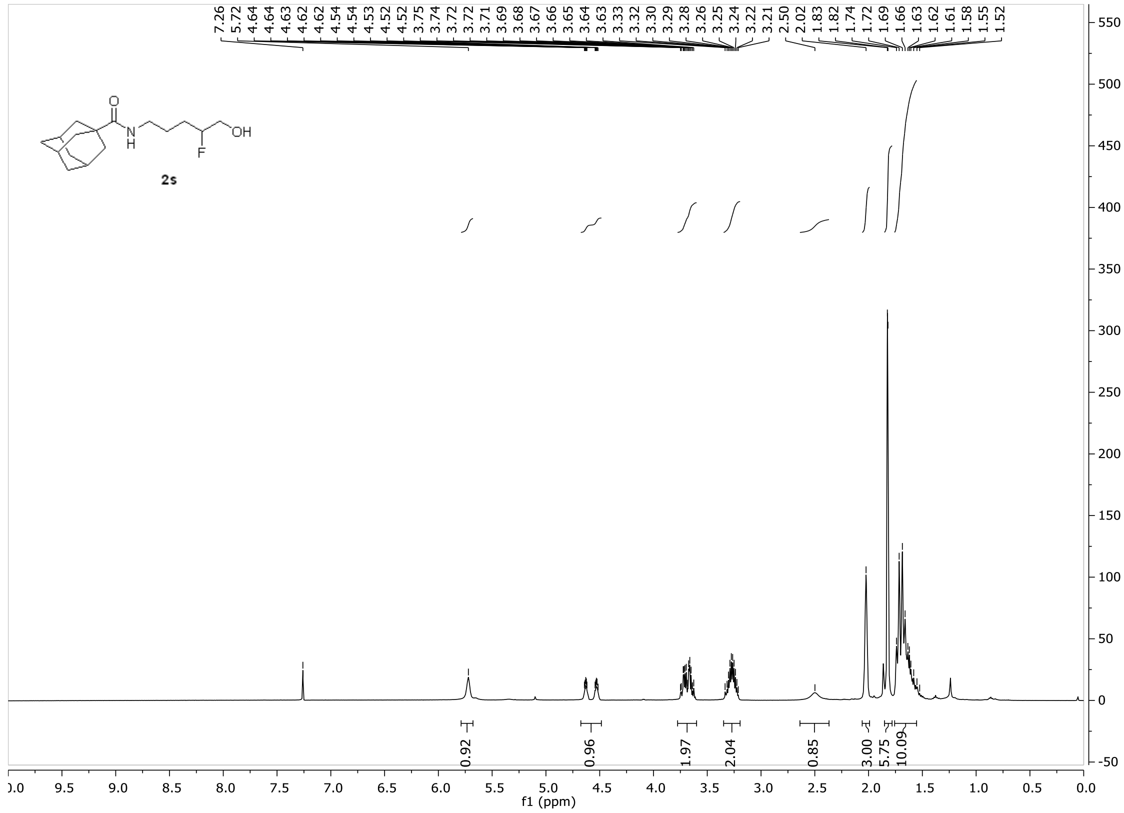

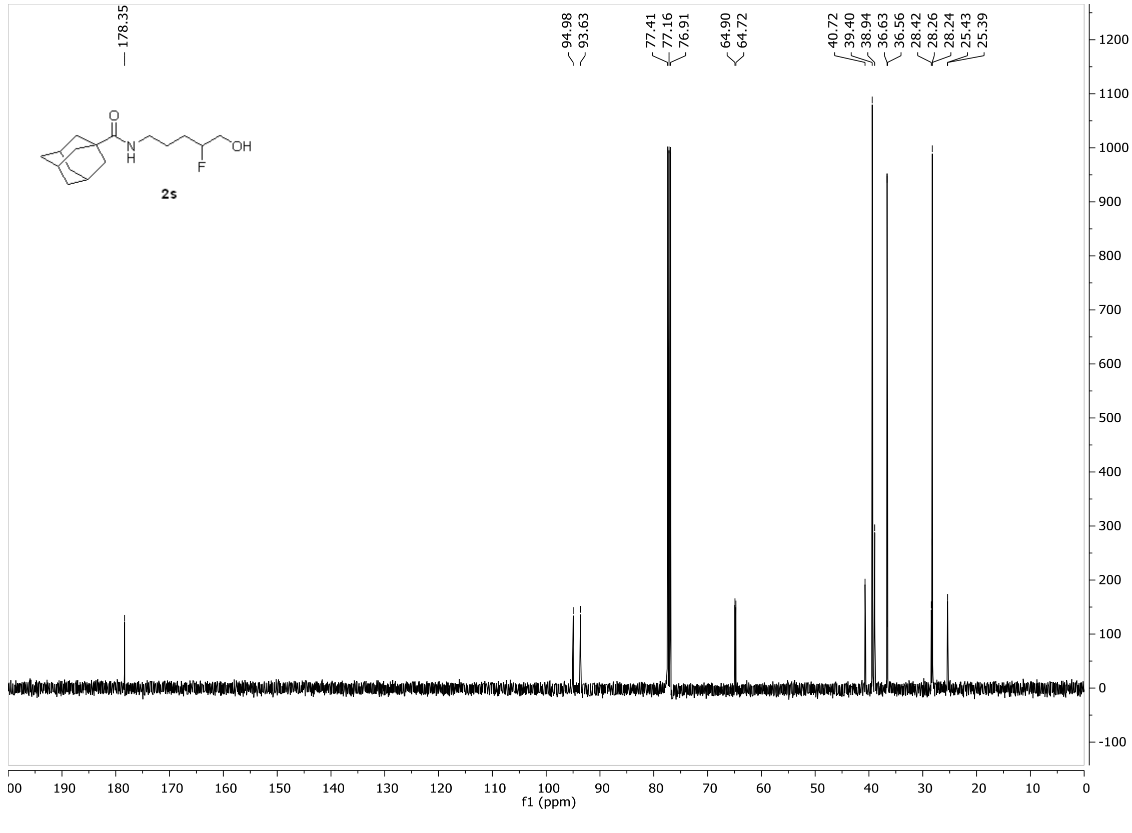

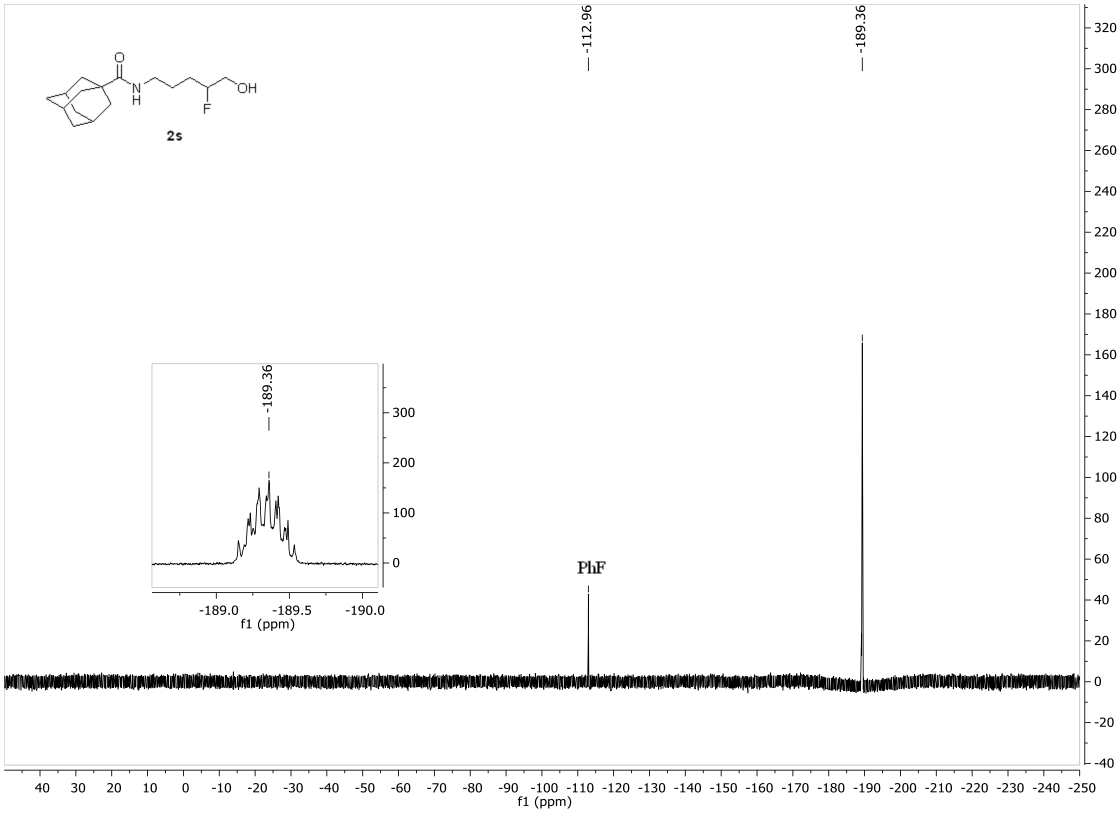
2s
-112.96
-189.36
PhF
f1 (ppm)

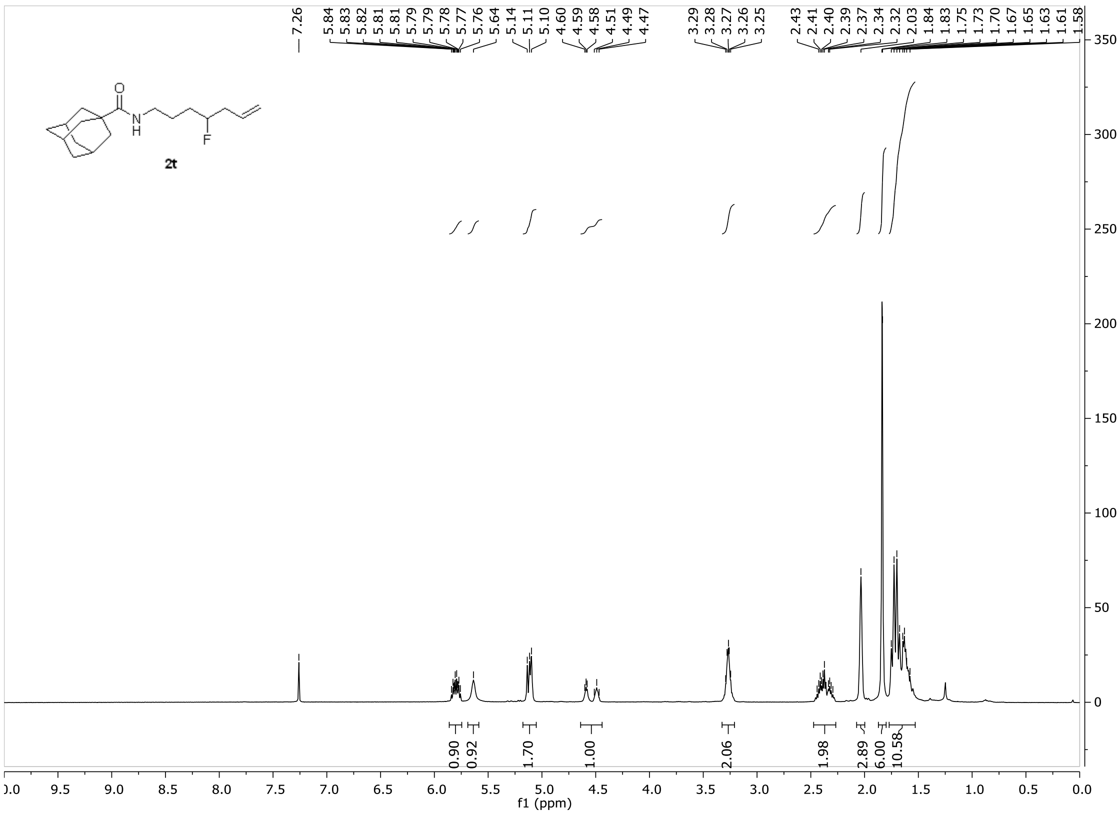
2t
7.26
5.84
5.83
5.82
5.81
5.81
5.79
5.79
5.78
5.77
5.76
5.64
5.14
5.11
5.10
4.60
4.59
4.58
4.51
4.49
4.47
3.29
3.28
3.27
3.26
3.25
2.43
2.41
2.40
2.39
2.37
2.34
2.32
2.03
1.84
1.83
1.75
1.73
1.70
1.67
1.65
1.63
1.61
1.58
0.90
0.92
1.70
1.00
2.06
1.98
2.89
6.00
10.58
f1 (ppm)

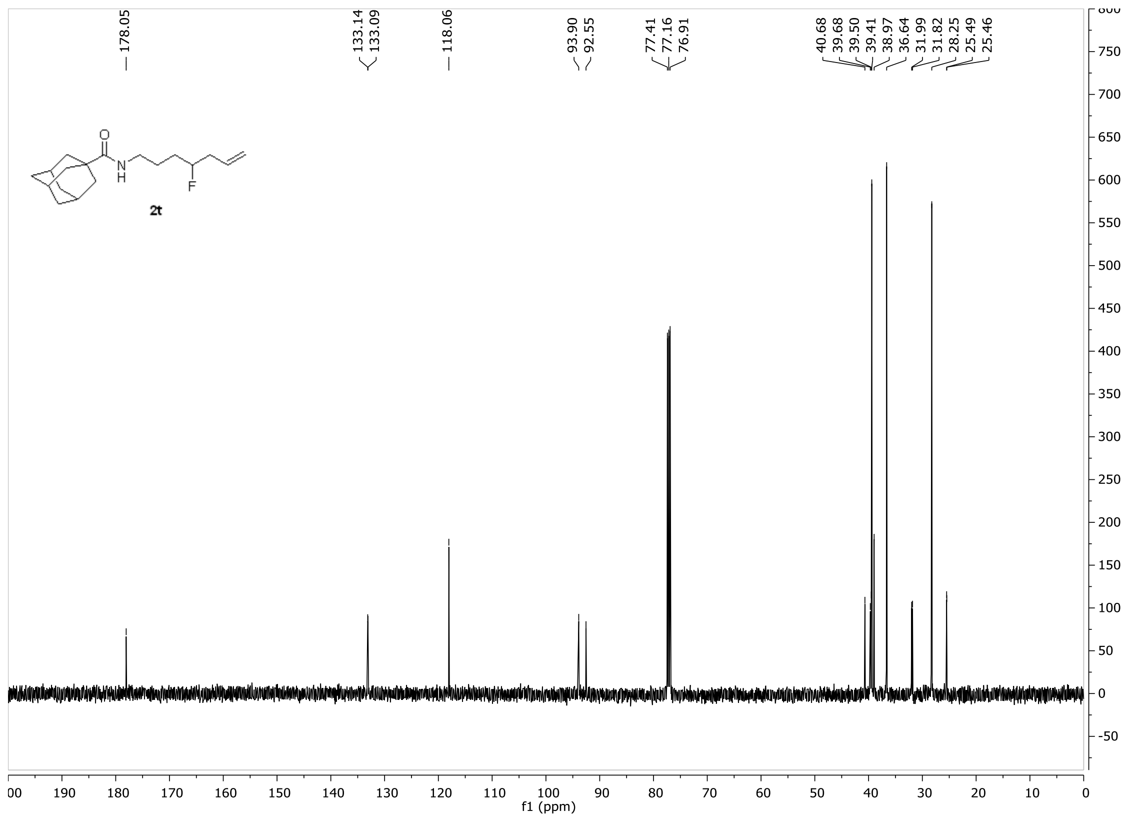
178.05
133.14
133.09
118.06
93.90
92.55
77.41
77.16
76.91
40.68
39.68
39.50
39.41
38.97
36.64
31.99
31.82
28.25
25.49
25.46
O
N
H
F
2t
f1 (ppm)

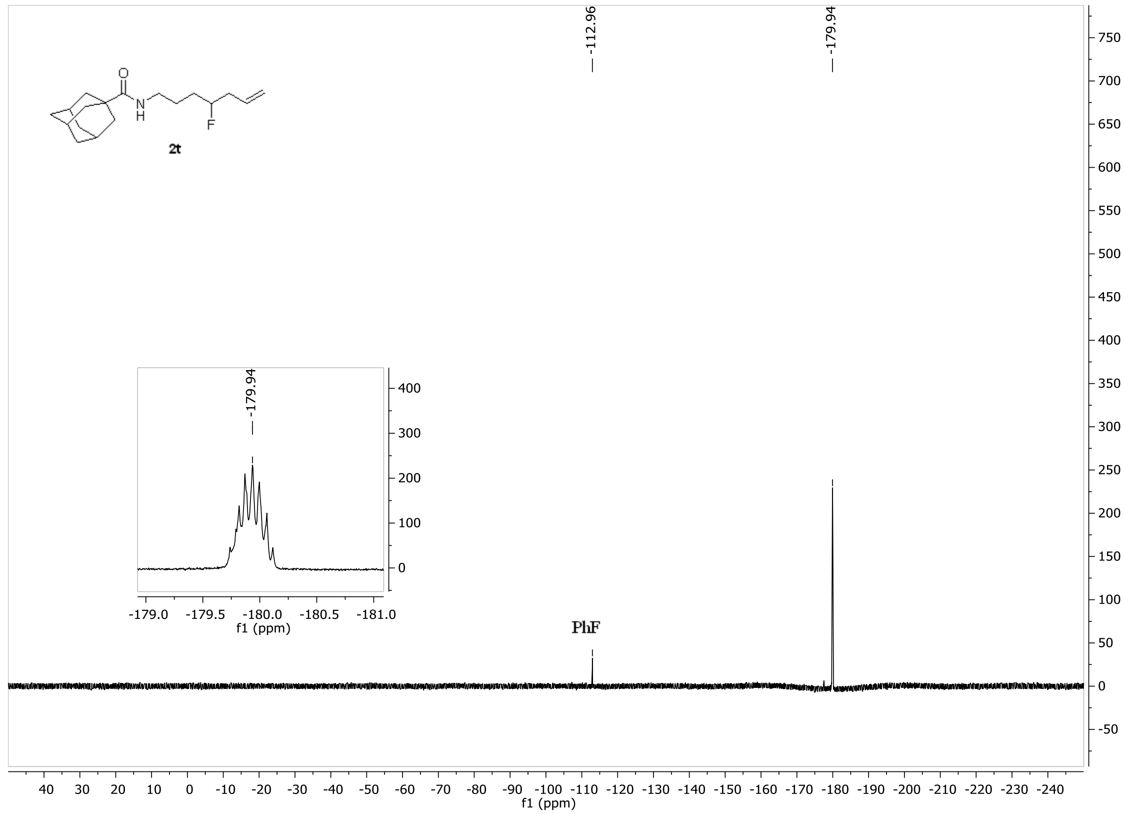
-112.96
-179.94
O
N
H
F
2t
-179.94
f1 (ppm)
PhF
f1 (ppm)

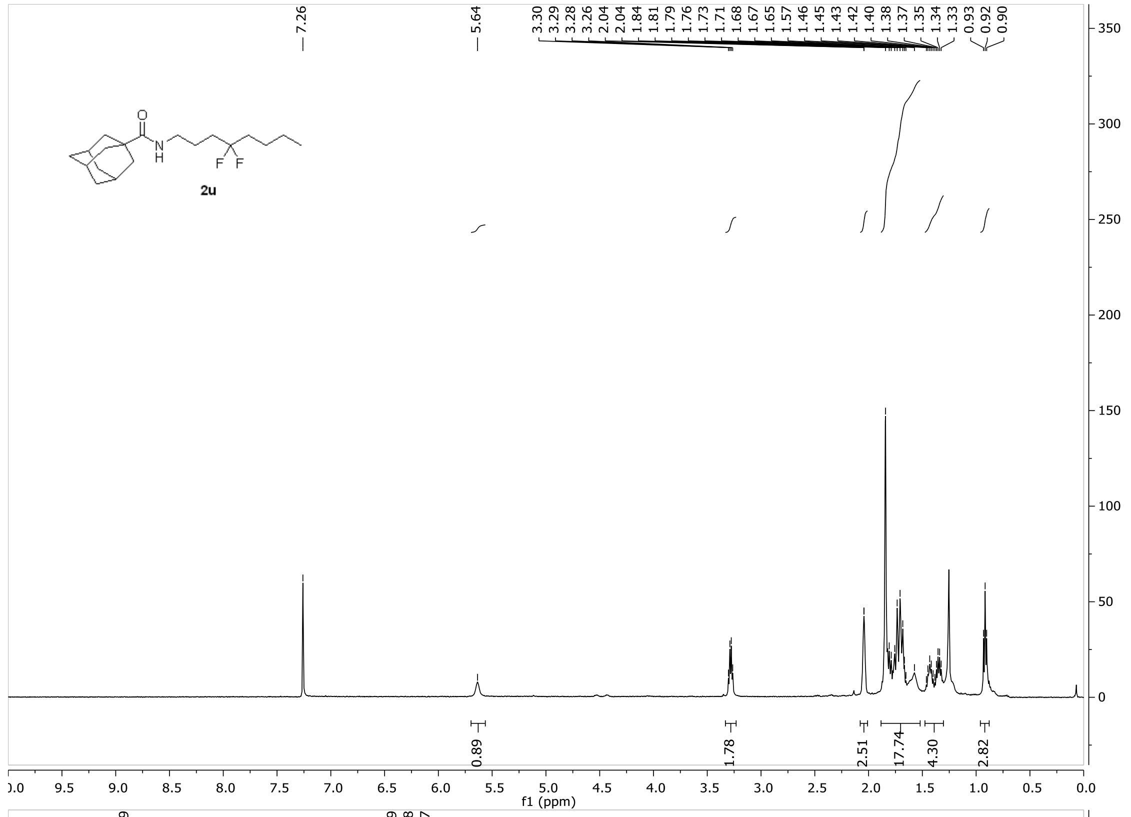

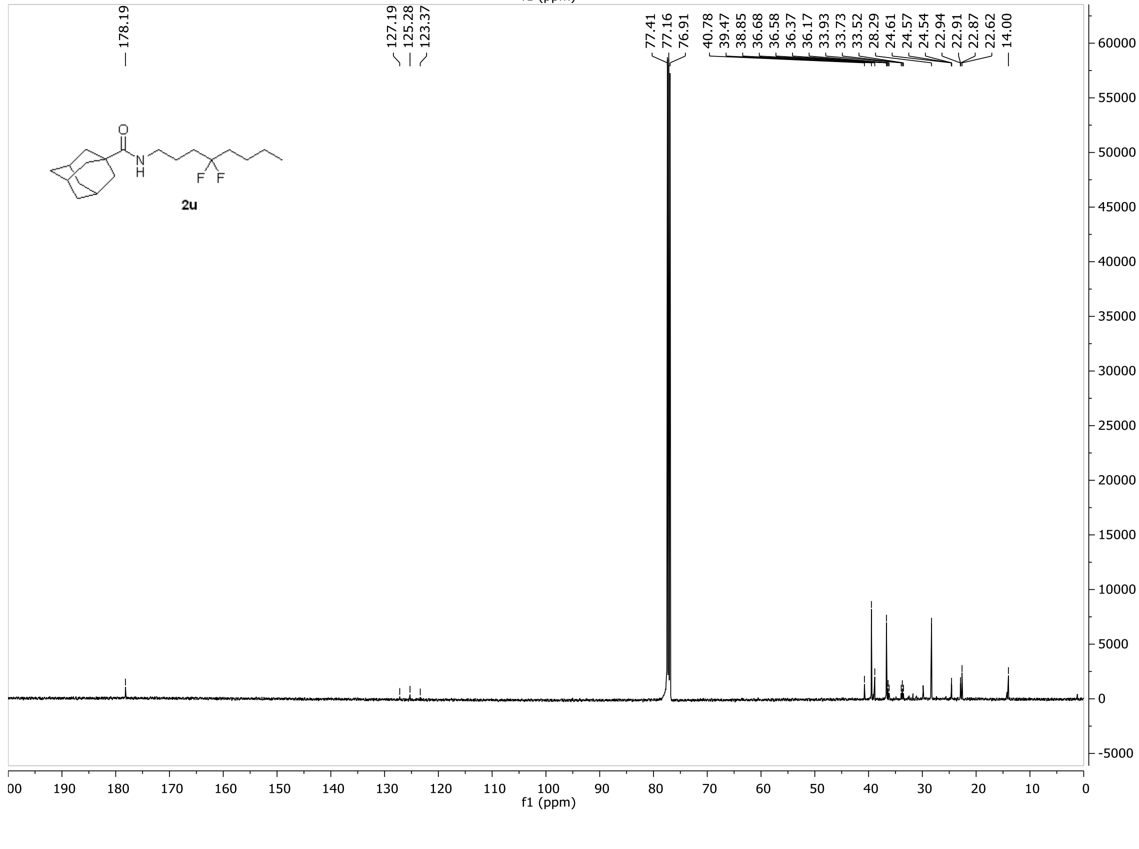

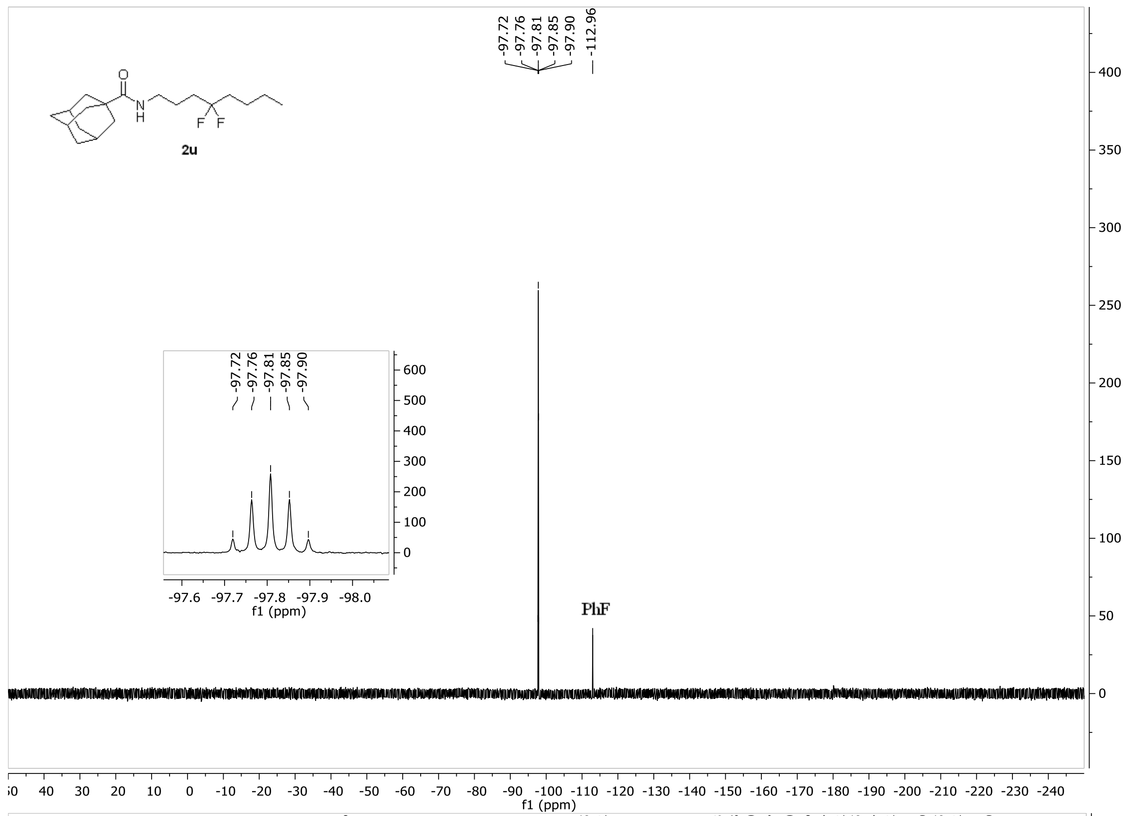
-97.72
-97.76
-97.81
-97.85
-97.90
-112.96
2u
PhF
f1 (ppm)

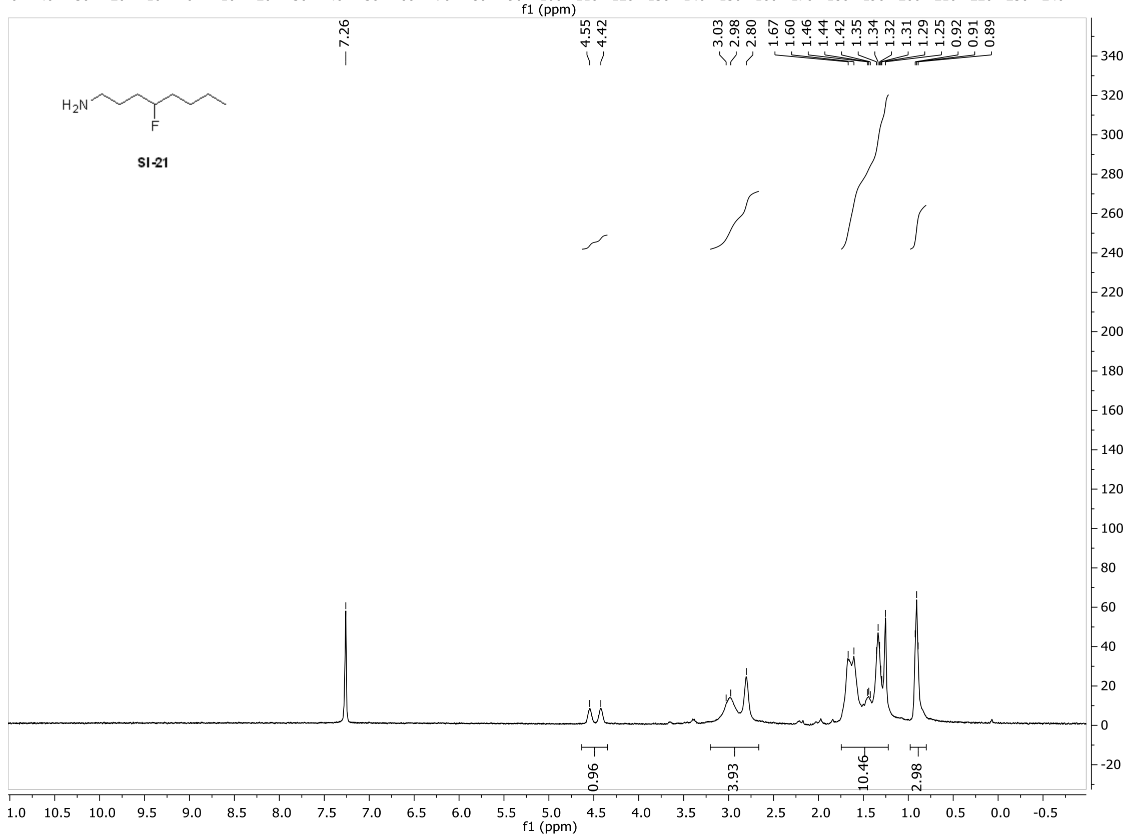
7.26
4.55
4.42
3.03
2.98
2.80
1.67
1.60
1.46
1.44
1.42
1.35
1.34
1.32
1.31
1.29
1.25
0.92
0.91
0.89
SI-21
0.96
3.93
10.46
2.98
f1 (ppm)

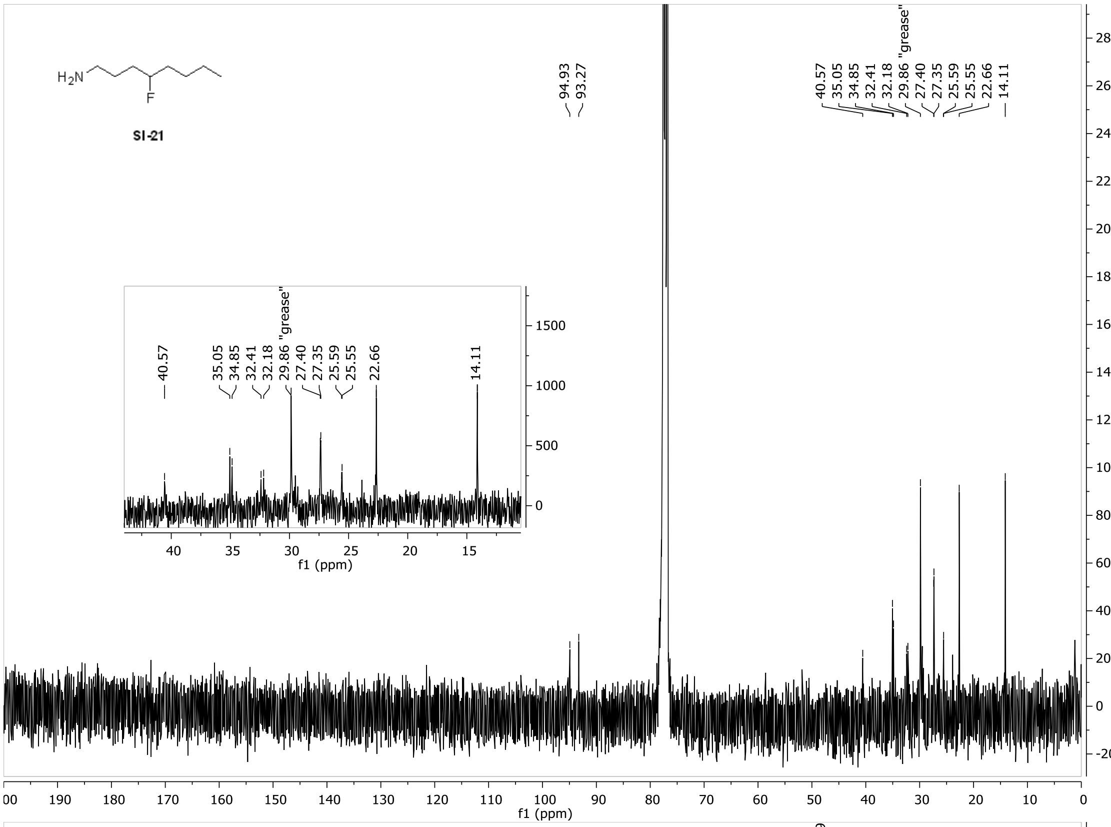
H2N
F
SI-21
94.93
93.27
40.57
35.05
34.85
32.41
32.18
29.86 "grease"
27.40
27.35
25.59
25.55
22.66
14.11
f1 (ppm)

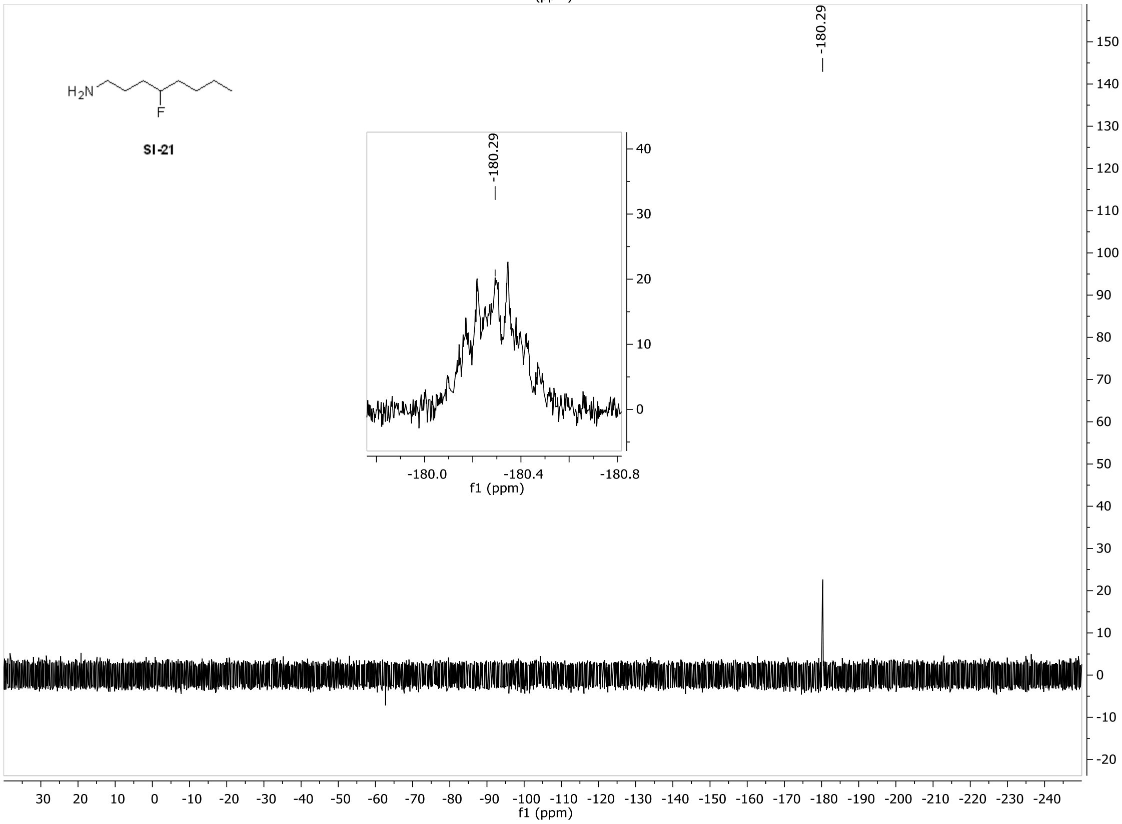
H2N
F
SI-21
-180.29
f1 (ppm)
